# Enantioselective Syntheses of 3,4-Dihydropyrans Employing Isochalcogenourea-Catalyzed Formal (4+2)-Cycloadditions of Allenoates

**DOI:** 10.1002/adsc.202400038

**Published:** 2024-03-04

**Authors:** Magdalena Piringer, Mario Hofer, Lukas S. Vogl, Peter Mayer, Mario Waser

**Affiliations:** a Institute of Organic Chemistry, Johannes Kepler University Linz, Altenbergerstr. 69, 4040 Linz, Austria + 43 732 2468 5411; b Department Chemie, Ludwig-Maximilians-Universität München, Butenandtstraße 5–13, 81377 München, Germany

**Keywords:** Organocatalysis, Lewis Bases, Isochal-cogenoureas, Cycloadditions, Allenoates

## Abstract

We herein successfully demonstrate the use of chiral isochalcogenoureas as Lewis Base catalysts for a variety of (4+2)-cycloaddition reactions of allenoates and different Michael acceptors. In all cases the same structural key-motive, a dihydropyran with a (Z)-configurated exocyclic double bond could be accessed as the major regio- and diastereoisomer in an enantioselective manner. Furthermore, these chiral dihydropyrans were successfully engaged in different follow-up transformations.

## Introduction

**Scheme 1:**
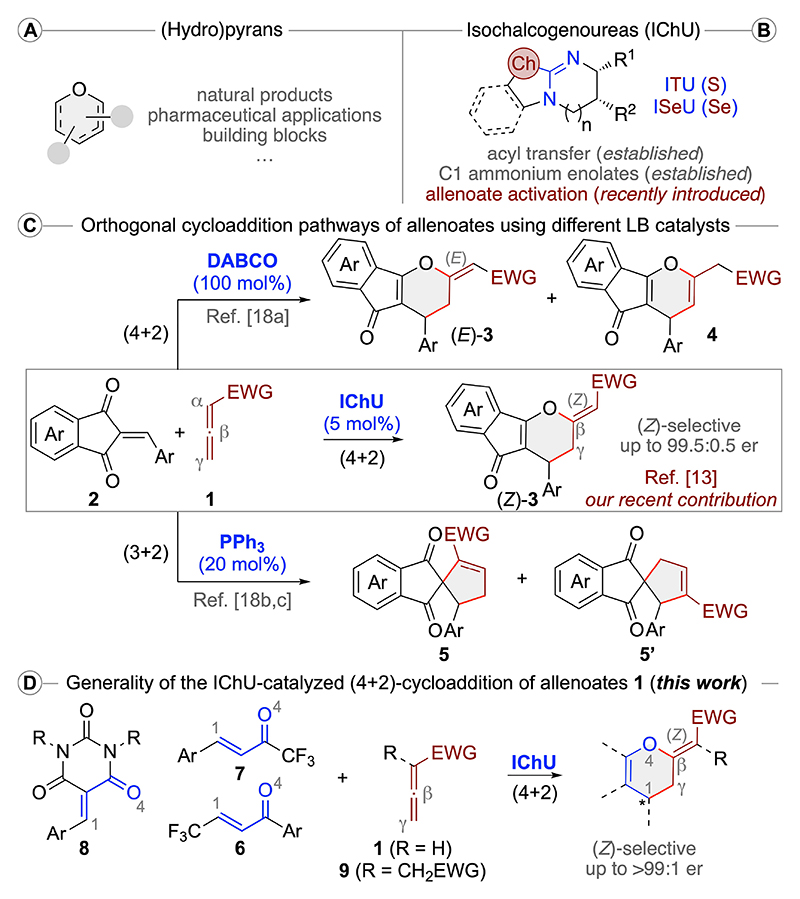
(Hydro)pyrans (A), isochalcogenoureas (B), our recently developed IChU-catalysed (4+2)-cycloaddition of allenoates 1 with acceptors 2 and the orthogonal reaction pathways accessible with different Lewis base catalysts (C), and the herein investigated generality of this process using different acceptors and allenoates (D).

Oxygen-containing 6-ring heterocycles like pyrans and (partially) hydrogenated derivatives thereof are frequently encountered motives in biologically active (natural) products or serve as valuable building blocks and synthesis intermediates en route to pharmaceutically relevant targets (Scheme 1A).^[1–5]^ Accordingly, the development of synthesis approaches to access structurally diverse 6-ring O-heterocycles in an asymmetric manner is an important task.^[2,3]^ (Formal) (4+2)-cycloadditions provide direct access to 6-ring motives and have been successfully used to access chiral partially hydrogenated pyran derivatives via formal (4+2)-cycloadditions of enones.^[3]^ Allenoates **1** are easily accessible versatile reagents which can undergo a multitude of different cyclization reactions.^[6]^ Remarkably, when activated with (chiral) Lewis base catalysts^[7]^ they can enter different (orthogonal) reaction pathways depending on the used catalysts and reaction partners,^[8–13]^ thus giving access to a diverse chemical space relying on simple starting materials and catalysts. Numerous applications utilizing allenoates for formal cycloadditions have been reported but unfortunately the identification of suited chiral catalysts that allow for high reactivities and high enantioselectivities can sometimes be tedious.^[14]^

Our group has a longstanding interest in allenoate chemistry and their utilization to access chiral O-heterocycles.^[14]^ Recently we initiated a program focusing on the use of easily accessible bench stable isochalcogenoureas (IChUs) as catalysts for allenoate reactions.^[13]^ Noteworthy, while these catalysts (Scheme 1B) have successfully been established for acyl-transfer reactions and C1 ammonium enolate transformations,^[15]^ they have, to the best of our knowledge, until our recent study not been utilized for allenoate activation. After thorough investigations we realized that these organocatalysts hold indeed much promise for allenoate reactions, as they allowed for highly enantioselective unprecedented formal (4+2)-cycloadditions of allenoates **1** with acceptors **2**.^[13]^ More specifically, this protocol gave the (Z)-configured dihydropyrans **3** with high levels of diastereo- and enantioselectivities either using established isothioureas (ITUs) or the very recently introduced isoselenoureas (ISeU) as catalysts (Scheme 1C).^[16,17]^ Remarkably, this outcome was in sharp contrast to reported studies on the reaction of **1** and **2** in the presence of tert. phosphines, which resulted in (3 + 2)-cyclizations, and tert. amines which gave rac-(E)-**3** and the double bond isomers **4**.^[18,19]^ Based on these encouraging previous results, which demonstrated that ITUs and ISeUs allow for novel highly enantio- and diastereoselective (4+2)-cycloadditions of allenoates in an unprecedented manner, we were wondering if this concept can be applied more generally to other classes of acceptors and allenoates.

As outlined in this contribution, the IChU-catalyzed formal (4+2)-cycloaddition of allenoates and different enone-type acceptors **6**–**8** is indeed a pretty general process, always delivering the corresponding 3,4-dihydropyrans with a (Z)-configurated exocyclic double bond in good to excellent enantioselectivities. Furthermore, the use of α-branched allenoates **9** was equally well tolerated, underscoring the generality of this protocol (Scheme 1D).

## Results and Discussion

**Figure 1:**
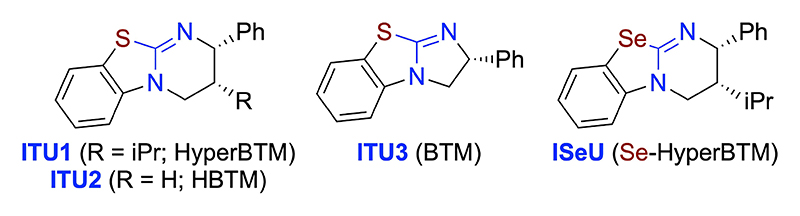
Chiral IChU used in these investigations.

We started by investigating the reaction of the β-CF_3_-containing enone **6 a** with allenoates **1 a** and **9 a** (Table 1 gives an overview of the most significant results obtained using the well-established catalysts **ITU1**–**3** and **ISeU** depicted in Figure 1).

This transformation was chosen because it was previously shown by Smith’s group^[20]^ that the reaction of compounds **6** with allenoates **1** in the presence of Cinchona alkaloids as chiral tert. amine Lewis base catalysts allows for the enantioselective syntheses of dihydropyrans **10** with (E)-configuration of the exocyclic double bond.^[19]^ Keeping in mind our recently established orthogonal (Z)-selective IChU-catalysed allenoate protocol using enones **2** (please compare with Scheme 1C),^[13]^ we were thus hoping for a similar (Z)-preference combined with a high enantioselectivity when employing the CF_3_-containing acceptors **6**.

First attempts using 20 mol% HyperBTM (**ITU1**) supported our proposal by yielding the expected (4+2)-product **10 a** in 74% with excellent enantioselectivity (> 99:1) and high Z-selectivity (> 95%) when carrying out the reaction under base-free conditions in toluene at 80 °C for approximately one day (entry 1). Testing other ITUs next (entries 1–3) showed the same trend as observed in our previous study,^[13]^ with **ITU1** being more active than **ITU2** and the 5-ring-based **ITU3** showing no reactivity at all (this can be rationalized by the lower nucleophilicity of **ITU3** as compared to the 6-ring derivatives **ITU1**-**2**^[21]^ and, in analogy to our recent study, supports again a mechanistic scenario where catalyst-addition to the allenoate is the rate-limiting step). Also very similarly to our previous observations, **ISeU** allowed for a slightly higher yield by maintaining the excellent levels of stereocontrol (entry 4). Further fine-tuning of reaction conditions (entries 5–11) showed that toluene is the solvent of choice and that addition of an external base has no positive effect on the yield (entry 9). Furthermore, the transformation is finished within 2 h when carried out at 80 °C (entries 10 and 11). Unfortunately however, lower catalyst loadings gave slightly lower yields, but without effecting the excellent enantioselectivities (entries 12 and 13).

Having established suited highly enantioselective (Z)-selective (4+2)-cycloaddition conditions for the parent allenoate **1 a**, we next tested the α-branched allenoate **9 a** (entries 14–20). Recently we found that such allenoates can serve as unique C1 synthons for formal (4+1)-cycloadditions with quinone methides under organophosphine catalysis.^[14b,c]^ In these applications, the catalyst adds to the allenoate’s sp-carbon in the established manner but after a subsequent proton transfer the CH_2_-group of the α-substituent becomes nucleophilic and then adds to the vinylogous acceptor. Interestingly, back-cyclization (and catalyst release) then occurs via the same carbon,^[14b,c]^ thus allowing for reaction pathways that differ significantly from established transformations of classical allenoates **1**. Based on these observations, we were thus wondering if a similar reactivity can also be observed under ITU catalysis or if **9 a** reacts in the same manner as established for **1 a**. Right from the first experiments we realized that **9 a** reacts indeed in the same highly enantio- and diastereo-selective (4+2)-fashion as **1 a** in the presence of either **ITU1** or **ISeU** (entries 14 and 15). However, conversion was incomplete after 2 h and thus the use of **9 a** required longer reaction times (entry 16) and also benefitted from a slightly larger excess of 1.6 equiv. of the allenoate (entries 17 and 18). Lower catalyst loadings were again detrimental (entries 19 and 20) but overall, it can be stated that **9 a** reacts like a decorated derivative of **1 a** without “active” participation of the sidechain under IChU catalysis.

**Scheme 2:**
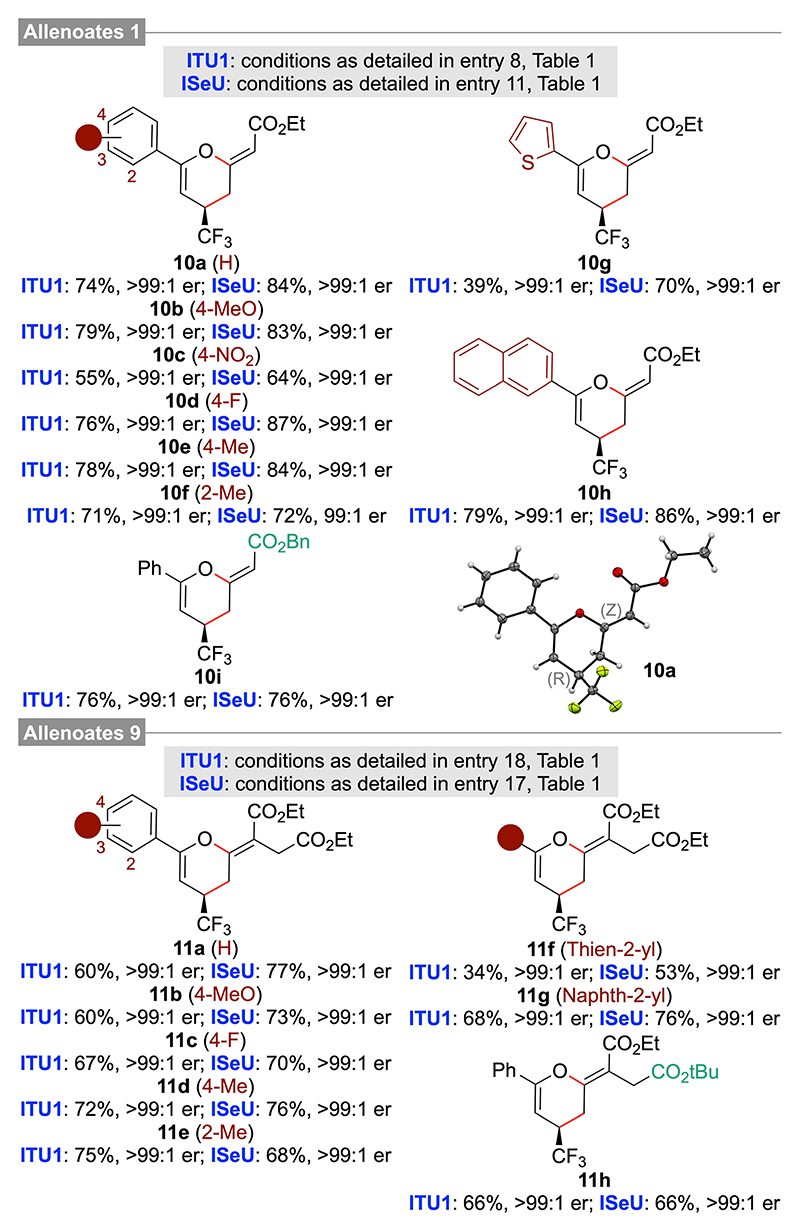
Application scope of the IChU-catalysed (4+2)-cycloaddition of allenoates 1 and 9 with acceptors 6 (the (R)-configuration of 10 a could be assigned by single crystal X-ray diffraction analysis^[22,23]^ and all other derivatives were assigned in analogy).

With suited conditions for the IChU-catalyzed (4+2)-cycloadditions of allenoates **1 a** and **9 a** with acceptor **6 a** established, we next investigated the application scope of these transformations by using differently decorated acceptors **6**, as well as varying the allenoate’s ester groups. As detailed in Scheme 2, these cycloadditions were found to be rather independent from the electronic properties of the acceptors (e. g. **10 b** vs **10 c**) and the steric effects (**10 e** vs **10 f** and **11 d** vs **11 e**), giving access to various products **10** and **11** in good isolated yields and with excellent enantioselectivities. Furthermore, the minor (E)-diastereomers were also only detected in trace amounts, thus underscoring the generality of this process.

**Scheme 3:**
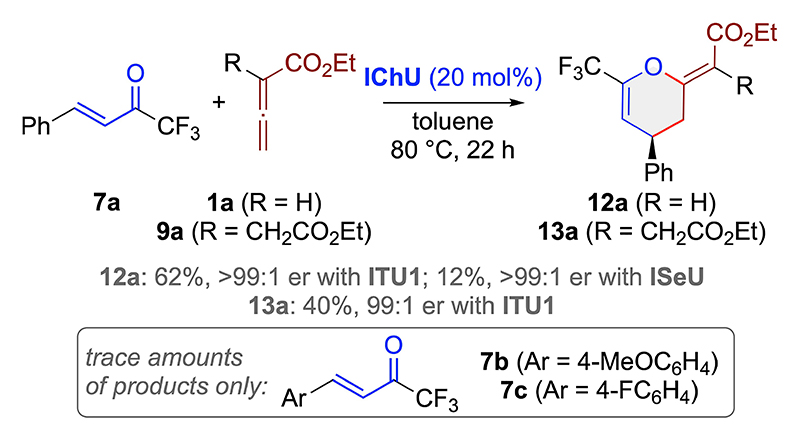
Use of acceptors 7.

Based on the excellent results obtained for acceptors **6**, we focused on the isomeric CF_3_-containing enones **7** next. Interestingly, in this case we were again able to access the targeted (Z)-configurated (4+2)-cycloaddition products **12 a** and **13 a** with excellent enantioselectivities when using the parent starting material **7 a** in the presence of **ITU1** (Scheme 3). Unfortunately, the yields were limited to around 40–60% and even after a detailed screening of different conditions and catalysts we were not able to overcome this limitation. Even more surprising, **ISeU** resulted in a significantly reduced yield for **12 a** only and did not provide **13 a** at all. Analysis of the reaction mixtures showed that acceptor **7 a** undergoes some oligomerization in the presence of IChUs, which accounts not only for the reduced yields, but also explains why the usually more reactive **ISeU** leads to even lower yields because of an increased rate of this oligomerization attributed to this catalyst’s higher nucleophilicity. Based on these less encouraging results, we did not explore the full application scope for different enones **7**, but we quickly realized that electronic alterations of these acceptors, as outlined for **7 b** and **7 c**, are not well tolerated as only trace amounts of products were formed in these cases (Scheme 3).

Besides CF_3_-enones **6** and **7**, we also investigated the suitability of barbiturate-based acceptors **8** for these IChU-catalyzed (4+2)-cycloadditions.^[24]^ First reactions of derivative **8 a** with allenoates **1 a** and **9 a** showed that these compounds can be successfully engaged in our (4+2)-cycloaddition protocol, delivering the dihydropyrans **14 a** and **15 a** with (Z)-configuration again (Table 2). A detailed screening of different catalysts and conditions for the reaction of **1 a** showed that again **ITU1** and **ISeU** deliver the best results with respect to yield and enantioselectivity (entires 1–8). Noteworthy, hereby **ITU3** also allowed for good enantioselectivities (entry 3) but with clearly reduced conversion rates as compared to **ITU1** (entry 1) and **ISeU** (entry 4). Unfortunately, the obtained enantioselectivities were limited to 86:14 er and thus clearly not as excellent as compared to **6** and **7** (vide supra). Further variations of catalyst loading, reaction temperature and time, and H_2_O content (entries 9–18) showed that elevated temperatures of 120 °C in combination with 10 mol% of catalyst allow for fast reactions yielding **14 a** in around 90% yield within one hour (entries 15 and 16), but with 86:14 er only. Interestingly, addition of molecular sieves or spiking of additional H_2_O did not really influence the reaction (entries 17 and 18), underscoring the general robustness of this protocol. With the results for allenoate **1 a** at hand, we then again tested the α-branched **9 a** (entries 19–24). In this case reaction rate and er were lower and we found slightly adapted conditions (as compared to **1 a**) with reduced temperature (80 °C), prolonged reaction time (22 h), and a larger excess of allenoate (1.6 equiv.; entries 22–24) being beneficial to obtain **15 a** in reasonable yields and enantioselectivities in the presence of 10 mol% **ISeU** (entry 22). In this case larger catalyst loadings turned out to be not beneficial (entry 24) and **ITU1** gave **15 a** with comparable enantioselectivity but in lower yield only (entry 23).

**Scheme 4:**
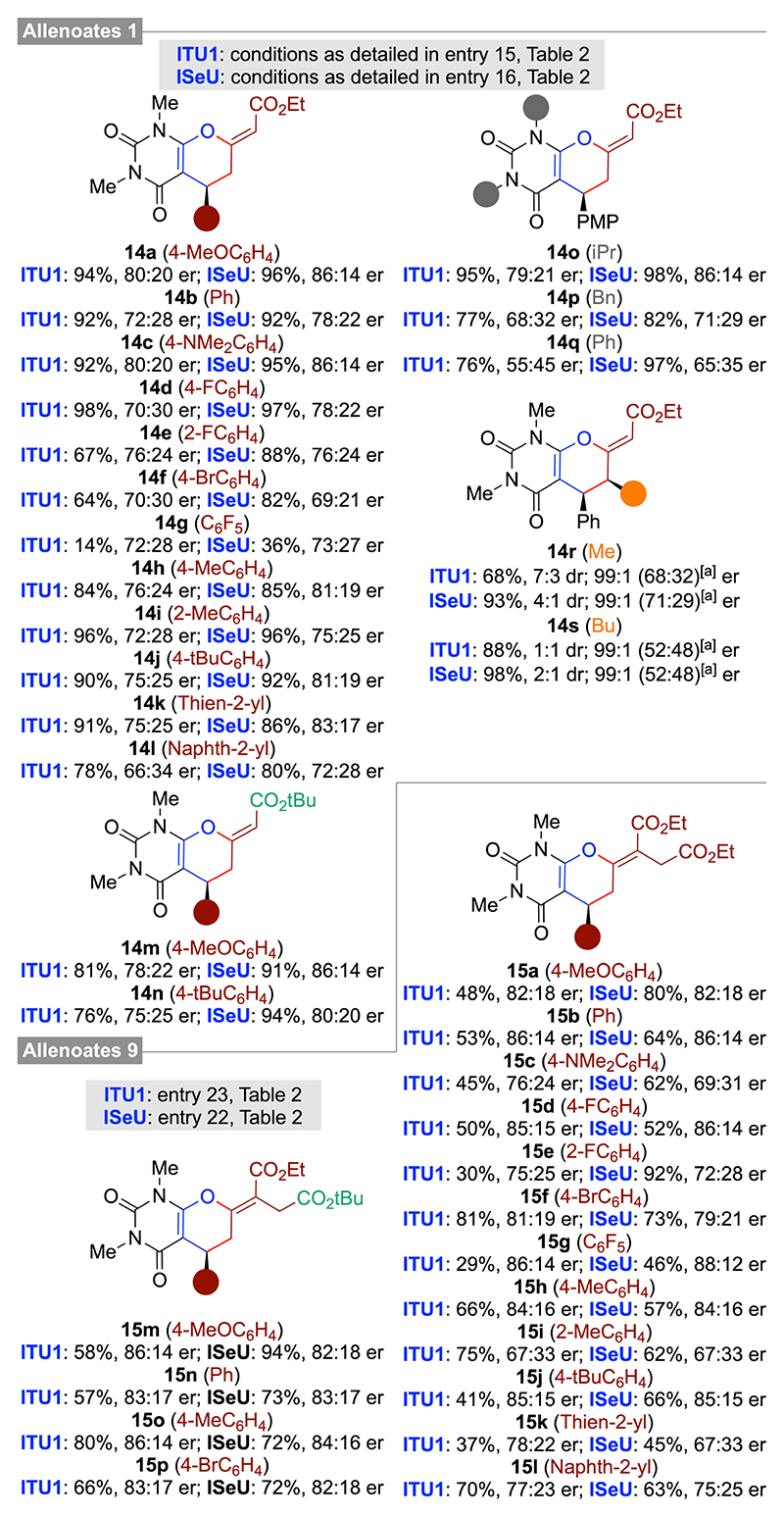
Application scope of the IChU-catalysed (4+2)-cycloaddition of allenoates 1 and 9 with acceptors 8 ([a] er of the minor trans diastereoisomer).

With robust and moderately enantioselective conditions for the IChU-catalyzed (4+2)-cycloadditions of allenoates **1 a** and **9 a** with the parent acceptor **8 a** at hand, we investigated the application scope for different allenoates and barbiturate acceptors **8**. As summarized in Scheme 4, these cycloadditions were again rather robust, i. e. when using **ISeU** as the catalyst, delivering the majority of products **14** and **15** in comparable yields, with high (Z)-selectivity, and with moderate to good enantioselectivities. Noteworthy less reactive are electron poor substrates as illustrated for the low-yielding syntheses of products **14 g** and **15 g**. In addition, the N-substituent of acceptors **8** has a pronounced effect on the observed outcome as well, as illustrated by the reduced enantioselectivities obtained for product **14 q**. We hereby also tested some γ-substituted allenoates and the corresponding products **14 r** and **14 s** could be accessed in high yields as well. Interestingly, the cis diastereoisomers (relative configuration assigned by NMR analysis) were always obtained with significantly higher enantioselectivities as compared to the trans diastereoisomers, a result which is in line with previous observations made for acceptors **2**.^[13]^

**Scheme 5:**
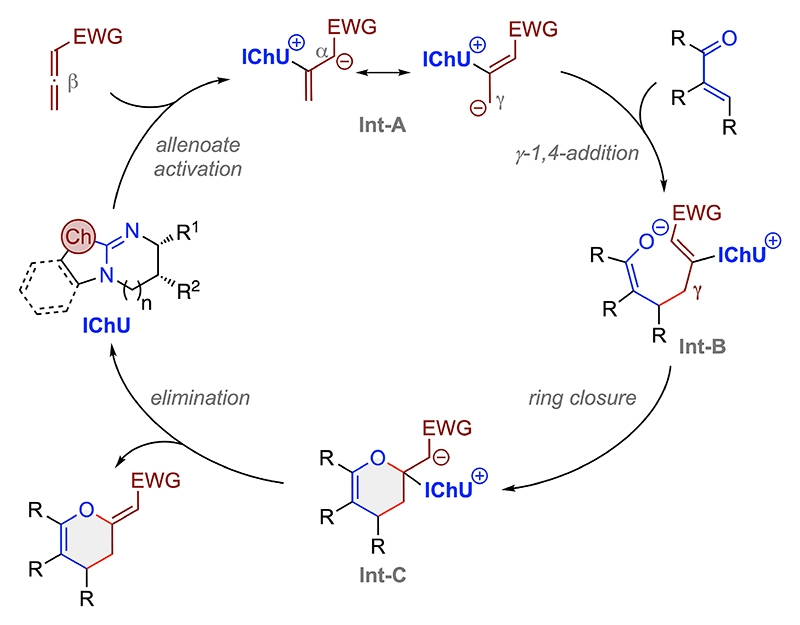
Proposed mechanistic scenario.

We have carried out detailed (computational) mechanistic investigations during our previous studies using acceptors **2** (Scheme 1C).^[13]^ Based on the results obtained therein and the conceptually analogous experimental outcome obtained in the present study we propose a mechanistic scenario as outlined in Scheme 5. First the catalyst adds to the β-position of the allenoate giving the resonance-stabilized **Int-A** (we have previously detected these intermediates by MS analysis and found this step being the rate-determining one^[13]^). This intermediate then undergoes the stereo-defining 1,4-addition to the Michael acceptor with its γ-carbon (giving **Int-B**). Cyclization then gives **Int-C** which finally undergoes catalyst elimination, which sets the double bond configuration and gives the herein accessed dihydropyran products with their (Z)-configurated exocylic double bonds. It should be noted that it may also be possible that the catalyst undergoes 1,4-addition to the Michael acceptor first, which can be an explanation for the partial decomposition of acceptor **7 a** (compare with Scheme 3). However this seems to be operational for selected very reactive compounds only, as we did not observe any reaction of the catalysts and acceptors **6** and **8** when carrying out control experiments (neither did we using compounds 2 before).

**Scheme 6:**
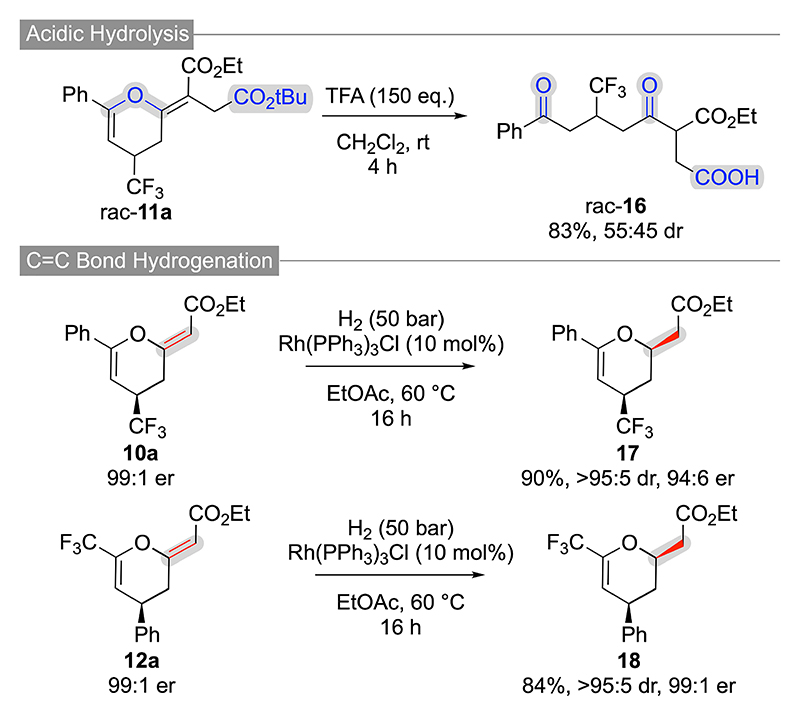
Further manipulations of CF_3_-dihydropyrans 10–12.

**Scheme 7:**
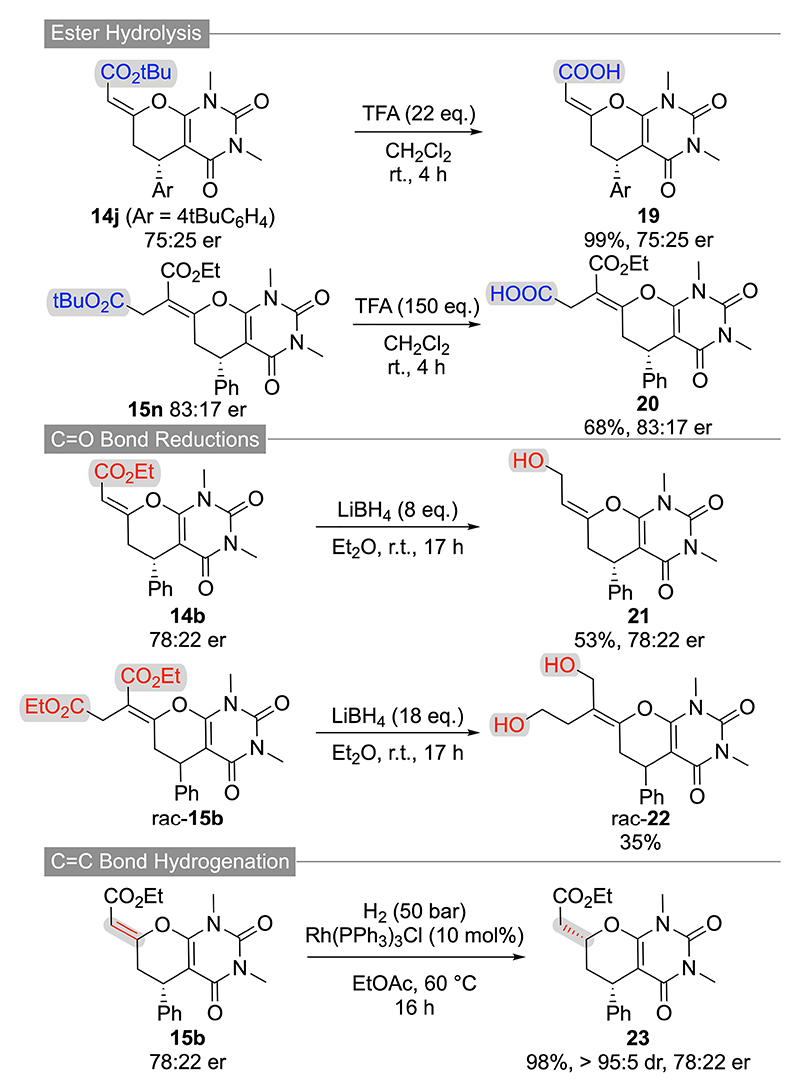
Further manipulations of barbiturate-based dihydropyrans 14 and 15.

Finally, we also explored the suitability of the herein accessed dihydropyrans for further manipulations (Schemes 6 and 7). First, the t-butyl ester-containing products **11 a, 14 j** and **15 n** could be successfully engaged in acidic hydrolysis reactions. Interestingly however, while **14 j** and **15 n** showed the expected t-butyl ester hydrolysis only (Scheme 7), compound **11 a** underwent a simultaneous ester-hydrolysis/dihydropyran-hydrolysis process giving compound **16** as a mixture of diastereoisomers (Scheme 6).

Besides these acidic hydrolysis reactions, we also succeeded in carrying out a variety of C=O and C=C doublebond reductions. For example, compounds **10 a** and **12 a** can be partially hydrogenated under homogeneous transition metal-catalysed conditions giving the dihydropyrans **17** and **18** in high yields, with excellent diastereoselectivities, and with no or only little erosion of enantiopurity (Scheme 6). Noteworthy, these compounds were also successfully accessed by Smith’s group starting from the analogous (E)-configurated starting materials^[20]^ and comparison of our analytical details with their reported data further confirms the absolute configuration of compounds **10 a** and **12 a**. Gratifyingly, an analogous homogenous C=C bond hydrogenation of **15 b** was possible as well (relative configuration of **23** was assigned by NOE correlations). We also tried to carry out heterogenous Pd/C-catalyzed hydrogenations which however were not possible to work selectively as they gave mainly several (unidentified) side products. In addition, it was also possible to reduce the ester functionalities of compounds **14 b** and **15 b** (giving products **21** and **22**) by using an excess of LiBH_4_ (Scheme 6).

## Conclusion

We herein successfully demonstrate the generality of our recently introduced isochalcogenourea-catalysed stereoselective allenoate (4+2)-cycloaddition protocol.^[13]^ Using CF_3_-based enones **6** and **7** in combination with allenoates **1** and **9** allows for enantioselective syntheses of CF_3_-containing dihydropyrans **10**–**12** (usually > 99:1 er). In addition, barbiturate-based acceptors **8** could be successfully employed as well, albeit with reduced enantioselectivities only. In all these cases, independent of the used starting materials, the targeted allenoate γ-addition-based (4+2)-cycloaddition products were obtained almost exclusively and with very high (Z)-selectivity for the exocyclic double bond, thus underscoring the generality of this IChU-catalysed allenoate-activation protocol. Furthermore, there herein access chiral dihydropyrans could be successfully engaged in various follow-up manipulations.

## Experimental Section^[23]^

### General Procedure for the Syntheses of Products 10 and 11

Acceptors **6** (0.1 mmol, 1 eq.) and Lewis base catalyst (**ITU1** or **ISeU**, 20 mol%) were transferred to a Schlenk flask and dissolved in PhMe (2.5 mL, 0.04 M) under N_2_ atmosphere at rt. Then, the respective allenoate (0.13 mmol, 1.3 eq. for allenoates **1** and 0.16 mmol, 1.6 eq. for allenoates **9**) was added and the mixture was stirred at 80 °C for 2 h (when using **1**) or for 22 h (using **9**). The mixture was cooled to rt., filtered over a Na_2_SO_4_ plug (2 cm) and evaporated to dryness to obtain the crude products **10** or **11**, respectively. The residue was purified by preparative TLC (heptanes/EtOAc, 2/1 for products **10**, 5/1 for products **11**) giving the cyclic products in the reported yields and enantiopurities.

### General Procedures for the Syntheses of Barbiturate-Based-Products 14 and 15

The barbiturate acceptors **8** (0.1 mmol, 1 eq.) and the Lewis base catalyst (**ITU1** or **ISeU**, 10 mol%) were transferred to a Schlenk flask and dissolved in toluene (5 mL, 0.02 M) under a N_2_ atmosphere at rt. Then, the respective allenoate (0.13 mmol, 1.3 eq. for allenoates **1** and 0.16 mmol, 1.6 eq. for allenoates **9**) was added in one portion and the mixture was stirred at 120 °C for 1 h (when using **1**) or at 80 °C for 22 h (using **9**). Afterwards, the mixture was cooled to rt., filtered over a Na_2_SO_4_ plug (2 cm) and evaporated to dryness to obtain the crude products **14** or **15**, respectively. The residues were purified by preparative TLC (heptanes/EtOAc, 1/1) giving the cyclic products in the reported yields and enantiopurities.

## Supplementary Material

Supporting information

## Figures and Tables

**Table 1:** Optimization of the (4+2)-cycloaddition of starting material 6 a.^[a]^

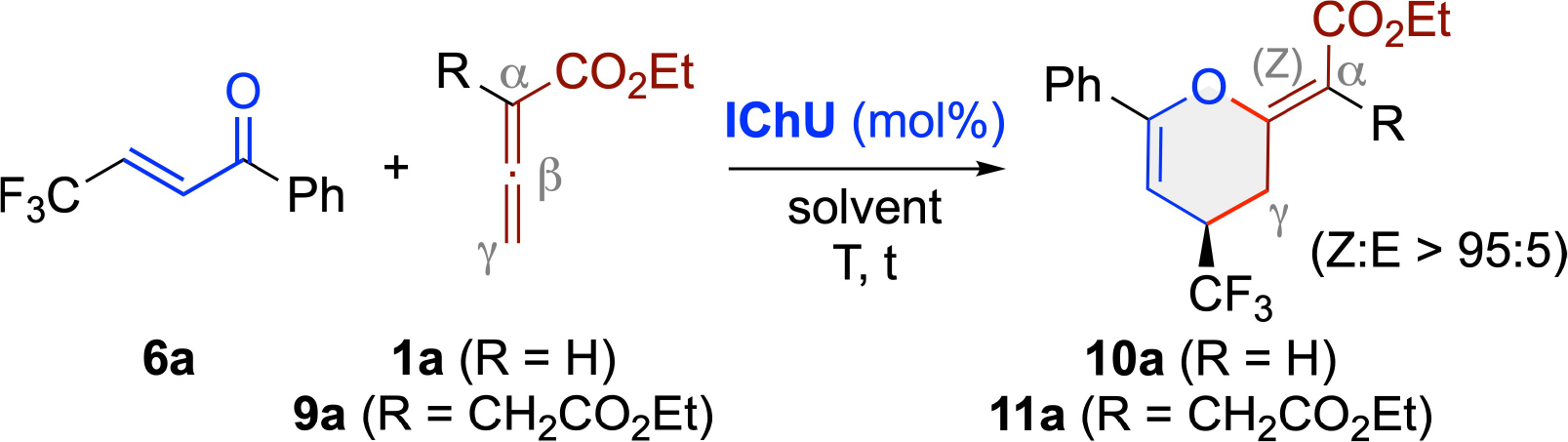						
Entry	IChU (mol%)	Solv.	T [°C]	t [h]	yield [%]^[b]^	er^[c]^
**1 a–**>**10 a**						
1	**ITU1** (20)	PhMe	80	22	74	> 99:1
2	**ITU2** (20)	PhMe	80	22	59	> 99:1
3	**ITU3** (20)	PhMe	80	22	< 10	n.d.
4	**ISeU** (20)	PhMe	80	22	84	> 99:1
5	**ITU1**(20)	PhMe	60	22	48	> 99:1
6	**ITU1** (20)	CHCl_3_	80	22	61	> 99:1
7	**ITU1** (20)	AcN	80	22	n.d.^[d]^	n.d.
8	**ITU1** (20)	PhMe	80	2	74	> 99:1
9	**ITU1** (20)^[e]^	PhMe	80	2	70	> 99:1
10	**ITU1** (20)	PhMe	100	2	72	> 99:1
11	**ISeU** (20)	PhMe	80	2	84	> 99:1
12	**ITU1** (10)	PhMe	80	2	52	> 99:1
13	**ISeU** (10)	PhMe	80	2	78	> 99:1
**9 a–**>**11 a**						
14	**ISeU** (20)	PhMe	80	2	64	> 99:1
15	**ITU1** (20)	PhMe	80	2	48	> 99:1
16	**ISeU** (20)	PhMe	80	22	71	> 99:1
17	**ISeU** (20)^[f]^	PhMe	80	22	77	> 99:1
18	**ITU1** (20)^[f]^	PhMe	80	22	60	> 99:1
19	**ITU1** (10)^[f]^	PhMe	80	22	43	> 99:1
20	**ISeU** (10)^[f]^	PhMe	80	22	68	> 99:1

[a] All reactions were run for the indicated time at the given temperature by using 0.1 mmol **6 a** and the respective allenoate (1.3 eq.) in the stated solvent (0.04 M based on **6 a**) unless otherwise noted.

[b] Isolated yields.

[c] Determined by HPLC using a chiral stationary phase. The absolute configuration of **10 a** was assigned by single crystal X-ray analysis.^[22,23]^

[d] Decomposition of starting materials.

[e] Addition of 1 equiv. K_2_CO_3_.

[f] Using 1.6 eq. **9 a**.

**Table 2:** Optimization of the (4+2)-cycloaddition of starting material 8 a.^[a]^

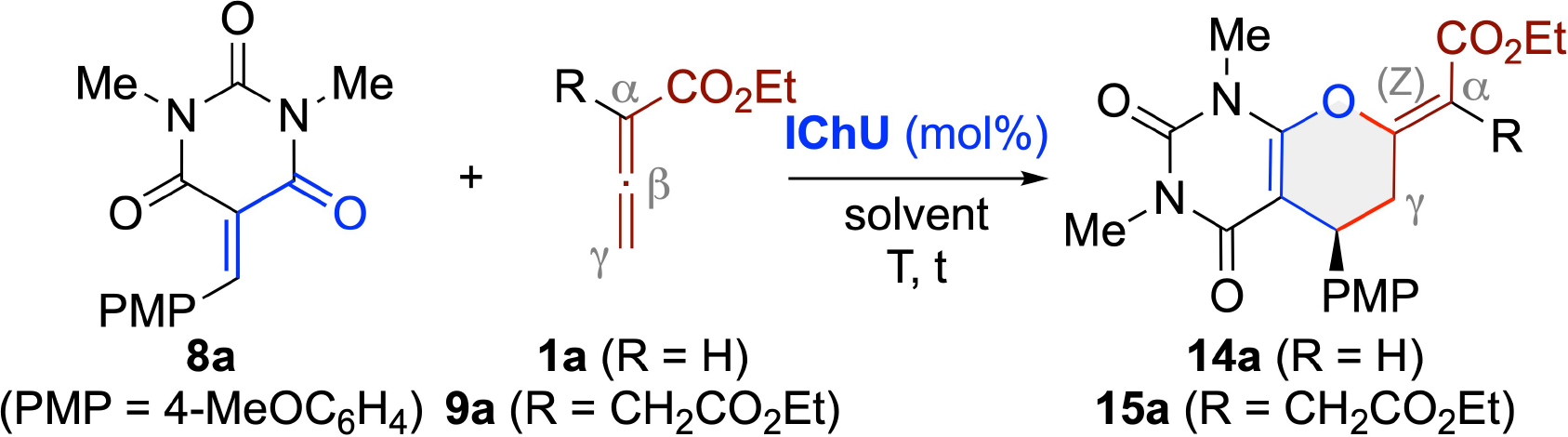						
Entry	IChU (mol%)	Solv.	T [°C]	t [h]	yield [%]^[b]^	er^[c]^
**1 a–**>**14 a**						
1	**ITU1** (20)	PhMe	80	22	75	80:20
2	**ITU2** (20)	PhMe	80	22	74	78:22
3	**ITU3** (20)	PhMe	80	22	26	86:14
4	**ISeU**(20)	PhMe	80	22	74	86:14
5	**ITU1** (20)	EtOAc	80	22	47	78:22
6	**ITU1** (20)	CHCl_3_	80	22	76	79:21
7	**ITU1** (20)	MeCN	80	22	n.d.^[d]^	80:20
8	**ITU1** (20)	THF	66	22	29	80:20
9	**ITU1** (20)	PhMe^[e]^	80	22	67	80:20
10	**ITU1** (10)	PhMe	80	22	75	80:20
11	**ITU1** (5)	PhMe	80	22	62	80:20
12	**ITU1** (10)	PhMe	60	22	72	80:20
13	**ITU1** (10)	PhMe	100	22	81	80:20
14	**ITU1** (10)	PhMe	120	22	94	80:20
15	**ITU1** (10)	PhMe	120	1	92	80:20
16	**ISeU** (10)	PhMe	120	1	96	86:14
17	**ISeU** (10)^[f]^	PhMe	120	1	83	86:14
18	**ISeU**(10)^[g]^	PhMe	120	1	87	86:14
**9 a–**>**15 a**						
19	**ISeU** (10)	PhMe	120	1	63	74:26
20	**ISeU** (10)	PhMe	120	22	67	72:28
21	**ISeU** (10)	PhMe	80	22	75	79:21
22	**ISeU** (10)^[h]^	PhMe	80	22	80	82:18
23	**ITU1** (10)^[h]^	PhMe	80	22	48	82:18
24	**ISeU**(20)^[h]^	PhMe	80	22	85	74:26

[a] All reactions were run for the indicated time at the given temperature by using 0.1 mmol **8 a** and the respective allenoate (1.3 eq.) in the stated solvent (0.02 M based on **8 a**) unless otherwise noted.

[b] Isolated yields.

[c] Determined by HPLC using a chiral stationary phase.

[d] Decomposition of starting materials.

[e] 0.04 M.

[f] Addition of 1 equiv. MS 4 Å.

[g] Addition of 2 equiv. H_2_O.

[h] Using 1.6 eq. **9 a**.
