# Supplementary material for: Enantioselective Syntheses of 3,4-Dihydropyrans Employing Isochalcogenourea-Catalyzed Formal (4+2)-Cycloadditions of Allenoates
Source: Adv Synth Catal. Author manuscript; Available in PMC 2024 Jun 5. (PMC7616061; doi:10.1002/adsc.202400038)
Supplement: Supporting information [file EMS196529-supplement-Supporting_information.pdf]

Advanced Synthesis & Catalysis

Supporting Information

Enantioselective Syntheses of 3,4-Dihydropyrans Employing Isochalcogenourea-Catalyzed Formal (4+2)-Cycloadditions of Allenoates

Magdalena Piringer, Mario Hofer, Lukas S. Vogl, Peter Mayer, and Mario Waser*© 2024 The Authors. Advanced Synthesis & Catalysis published by Wiley-VCH GmbH. This is an open access article under the terms of the Creative Commons Attribution License, which permits use, distribution and reproduction in any medium, provided the original work is properly cited.

Supporting Information

Enantioselective Syntheses of 3,4-Dihydropyrans Employing Isochalcogenourea-Catalyzed Formal (4+2)-Cycloadditions of Allenoates

Magdalena Piringer,[a] Mario Hofer,[a] Lukas S. Vogl,[a] Peter Mayer,[b] and Mario Waser[a,*]

[a] Institute of Organic Chemistry, Johannes Kepler University Linz, Altenbergerstr. 69, 4040 Linz, Austria
+43 732 2468 5411; mario.waser@jku.at

[b] Department Chemie, Ludwig-Maximilians-Universität München, Butenandtstraße 5–13, 81377 München, Germany

Contents

1. General information 3

2. Synthesis of 1,3-disubstituted barbiturate Michael acceptors 4

2.1 General procedure A for 1,3-dimethyl barbiturate Michael acceptors 4

2.2 Synthesis of 1,3-diphenyl/diisopropyl/dibenzyl barbiturate acceptors 4

Overview 4

3. Synthesis of trifluoromethyl enone Michael acceptors 5

4. Synthesis of 6-membered heterocycles 6

4.1 General information 6

4.2 General procedure C for the synthesis of 6-membered heterocycles 14 and 15 6

4.3 General procedure D for the synthesis of 6-membered heterocycles 10 and 11 6

4.4 General procedure E for the synthesis of 6-membered heterocycles 12 and 13 6

5. Characterization of novel starting materials 7

6. Product characterization: 6-membered heterocycles 9

7. Product diversification 44

8. Crystallographic structure of 10a 50

10. References 53

11. Appendix: NMR spectra and HPLC chromatograms 54

11.1 NMR spectra of the novel starting materials - barbiturates 54

11.2 NMR spectra of the 4+2 cyclization products 60

11.3 NMR spectra of the product diversification 129

11.4 HPLC chromatograms of the cyclization products 139

11.5 HPLC chromatograms of the product diversification 250

1. General information

NMR spectra were recorded on a Bruker Avance III 300 MHz spectrometer with a broad band observe probe and a sample changer for 16 samples, a Bruker Avance DRX 500 MHz spectrometer, and a Bruker Avance III 700 MHz spectrometer with an Ascend magnet which are all property of the Austro Czech NMR Research Center "RERI uasb". All NMR spectra were referenced on the solvent residual peak ($CDCl_3$: δ 7.26 ppm for ^1H NMR and δ 77.16 ppm for ^{13}C NMR). NMR data are reported as follows: chemical shift (δ ppm), multiplicity (s = singlet, d = doublet, t = triplet, q = quartet, m = multiplet, bs = broad singlet), coupling constants (Hz), and integrals.

High resolution mass spectra were obtained using an Agilent QTOF 6520 with ESI source. Optical rotations were measured on a Schmidt+Haensch Unipol L 100 polarimeter ($[\alpha]_D$ values are listed in $deg \cdot cm^3 \cdot g^{-1} \cdot dm^{-1}$; concentration c is given in g/100 mL).

Preparative column chromatography was carried out using Davisil LC 60A 70– 200 MICRON silica gel. (Preparative) thin layer chromatography was performed on Macherey-Nagel pre-coated TLC plates (silica gel, 60 F254, 0.20 mm, ALUGRAM® Xtra SIL, respectively, 0.50 mm, SIL G-50). TLC plates were visualized under 254 nm UV lamp. Semipreparative HPLC was carried out using a Dionex Ultimate 3000 system with variable wavelength detection and a Grace Alltima Silica 10 µm 250x10 mm column.

Enantiomeric ratios (*e.r.*) were determined by HPLC analysis using a Dionex Summit, a Dionix Ultimate 3000 or a Shimadzu Prominence HPLC system with a CHIRAL ART Amylose-SA (4.6 mm × 250 mm, 5 µm), a CHIRAL ART Cellulose-SB (4.6 mm × 250 mm, 5 µm), a CHIRALCEL® OD-H (4.6 mm × 250 mm, 5 µm) or a CHIRALPAK® AD-H (4.6 mm × 250 mm, 5 µm) chiral stationary phase.

Dry solvents were taken from an mBRAUN SPS solvent purifier. All reactions were run under an inert atmosphere (argon or nitrogen) unless otherwise stated. All chemicals were purchased from commercial suppliers and used without further purification unless otherwise stated. Technical grade solvents for extraction or chromatography (EtOAc, heptanes and DCM) were distilled prior to use.

Catalyst **ITU1** (HyperBTM) was purchased from commercial sources and selenium catalyst **ISeU** (Se-HyperBTM) was prepared in-house following established procedures [1]. Allenoates **1** [2] and **9** [3] were synthesized according to known literature procedures.

Melting points were determined by a BÜCHI melting point apparatus M-560.

2. Synthesis of 1,3-disubstituted barbiturate Michael acceptors

The 1,3-dimethyl barbituric acid-based acceptors were synthesized from commercially available 1,3-dimethyl barbituric acid following a well-known one-step procedure [4].

2.1 General procedure A for 1,3-dimethyl barbiturate Michael acceptors

In analogy to a known procedure [4], the respective aldehyde (1 eq.) was added to a homogenous solution of 1,3-dimethyl barbituric acid (1 eq.) in deionized water (0.5 mol L^{-1}) and vigorously stirred at rt. until a precipitate was formed. Then, stirring was continued for approximately 30 min. The reaction was terminated and the precipitated was filtered off, washed with warm water and Et_2O and dried under high vacuum to give the desired acceptors **8** as powdery solids. Analytical data are in accordance with the literature.

2.2 Synthesis of 1,3-diphenyl/diisopropyl/dibenzyl barbiturate acceptors

Overview

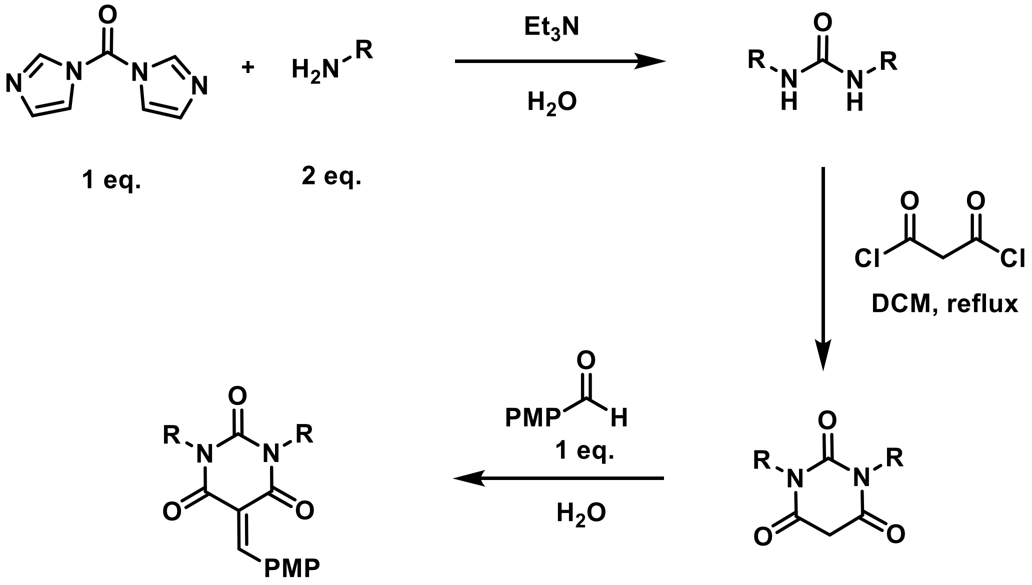

R = phenyl, isopropyl, benzyl

Synthesis of 1,3-diphenyl urea

The respective urea was prepared analogously to an already described method [5] and directly used in the next step without further purification. Analytical data are in accordance with the literature.

Synthesis of 1,3-diisopropyl/dibenzyl urea

Adapting literature procedure [5], carbonyldiimidazol (12 mmol, 1.94 g) was added in four portions to an aqueous solution (20 mL) of the respective primary amine (2 eq.) and Et_3N (2 eq.). Stirring at rt. was continued for 6 h. After completion of the reaction time, the precipitate was filtered, washed thoroughly with water and dried properly under high vacuum to give the desired urea as white to slightly beige solid (30-45% yield).

Synthesis of 1,3-disubstituted barbituric acids

Adapting literature procedure [5], malonyl chloride (1.3 eq.) was added to a vigorously stirred suspension of the respective urea in anhydrous DCM (0.1 M). Subsequently, the reaction mixture was refluxed and conversion was followed by TLC (DCM). After completion (~6.5 h), the reaction was quenched by the addition of water and the phases were separated. The aqueous phase was extracted with DCM (3×) and the combined organic phases were dried with Na_2SO_4, filtered, and evaporated to dryness to give the crude product as beige to slightly yellowish solids.

Purification was either done by recrystallization from EtOH or by column chromatography (heptanes/EtOAc, 10/1 to 5/1, 85-95% yield).

General Procedure B for 1,3- diphenyl/diisopropyl/dibenzyl barbiturate Michael acceptors

According to literature [5], *p*-anisaldehyde (1 eq.) was added to a suspension of the respective barbituric acid (1 eq.) in deionized water (0.5 mol L^{-1}). Then, the mixture was refluxed for approximately 4 h. Afterwards, the reaction was cooled to rt. and the formed precipitated was filtered off, washed with Et_2O and dried under high vacuum. The crude acceptor was purified by column chromatography (heptanes/EtOAc, 5/1 to 1/1) to give the desired compound as powdery yellow solid.

3. Synthesis of trifluoromethyl enone Michael acceptors

Acceptors **6a-6h** [6] and **7a** [7] were synthesized according to well-known procedures with spectroscopic data in accordance to literature.

4. Synthesis of 6-membered heterocycles

4.1 General information

In general, the products were obtained with high preference for the (Z)-diastereomer in all cases. Stereoisomeric ratio and conversion were determined from crude ^{1}H-NMR spectra. Traces of eventually formed (E)-diastereomers could be removed chormatographically. Racemic samples were obtained by performing the reaction using Okamoto's achiral catalyst (DHPB) for the barbiturates **8** or, in the case of the trifluoromethyl acceptors **6**, with racemic **ITU1** (HyperBTM).

4.2 General procedure C for the synthesis of 6-membered heterocycles 14 and 15

Adapting the literature procedure [2], barbiturate acceptor **8** (0.1 mmol, 1 eq.) and catalyst (**ITU1/ISeU**, 10 mol%) were dissolved in PhMe (5 mL, 0.02 mol L^{-1}) in a Schlenk flask under N_2 at rt. Then, the respective allenoate (0.13 mmol, 1.3 eq. for allenoates **1** and 0.16 mmol, 1.6 eq. for allenoates **9**) was added in one portion and the mixture was stirred at 120 °C for 1 h or at 80 °C for 22 h. Afterwards, the mixture was cooled to rt., filtered over a Na_2SO_4 plug (2 cm) and evaporated to dryness to obtain crude products **14** or **15**, respectively. The residue was purified by preparative TLC (heptanes/EtOAc, 1/1) to give the cyclic product in high purity and yield.

4.3 General procedure D for the synthesis of 6-membered heterocycles 10 and 11

Adapting the literature procedure [2], the respective (*E*)-4,4,4-trifluoro-but-2-en-1-one **6** (0.1 mmol, 1 eq.) and catalyst (**ITU1/ISeU**, 20 mol%) were dissolved in PhMe (2.5 mL, 0.04 mol L^{-1}) in a Schlenk flask under N_2 at rt. Then, the respective allenoate (0.13 mmol, 1.3 eq. for allenoates **1** and 0.16 mmol, 1.6 eq. for allenoates **9**) was added and the mixture was stirred at 80 °C for 2 h or for 22 h. The mixture was cooled to rt., filtered over a Na_2SO_4 plug (2 cm) and evaporated to dryness to obtain crude products **10** or **11**, respectively. The residue was purified by preparative TLC (heptanes/EtOAc, 2/1 for products **10**, 5/1 for products **11**) to give the cyclic product in high purity and good yield.

4.4 General procedure E for the synthesis of 6-membered heterocycles 12 and 13

Further adapting the literature procedure [2], (*E*)-1,1,1-trifluoro-but-3-en-2-one **7a** (0.1 mmol, 1 eq.) and catalyst (**ITU1/ISeU**, 20 mol%) were dissolved in PhMe (5 mL, 0.02 mol L^{-1}) in a Schlenk flask under N_2 at rt. Then, the respective allenoate **1** (0.13 mmol, 1.3 eq) was added and the mixture was stirred at 80 °C for 4 h. The mixture was cooled to rt., filtered over a Na_2SO_4 plug (2 cm) and evaporated to dryness. The residue was purified by preparative TLC (heptanes/EtOAc, 2/1) to give the cyclic product in high purity and moderate yield.

5. Characterization of novel starting materials

1,3-Dimethyl-5-((perfluorophenyl)methylene)pyrimidine-2,4,6(1*H*,3*H*,5*H*)-trione (8b)

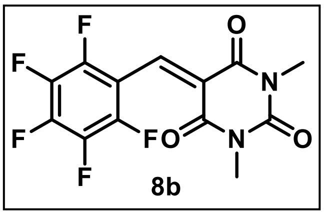

8b

Barbiturate **8b** was synthesized according to general procedure **A** and obtained in a yield of 70% as a slightly yellowish solid after column chromatography on silica (DCM as eluent).

^{1}H-NMR (300 MHz, $CDCl_3$, 298 K) δ / ppm = 8.17-8.16 (m, 1 H, -C=C**H**), 3.40 (s, 3 H, -NC**H$_3$**), 3.30 (s, 3 H, -NC**H$_3$**).

^{13}C-NMR (75 MHz, $CDCl_3$, 298 K) δ / ppm = 160.3, 159.0, 150.8, 140.0, 124.6, 109.7, 109.6, 29.2, 28.7.

^{19}F-NMR (282 MHz, CDCl3, 298 K) δ / ppm = -134.8 (2 F, Ar-**F**), -150.2 (1 F, Ar-**F**), -161.8 (2 F, Ar-**F**).

Melting point: 74.9-77.4 °C

HRMS (ESI-TOF): *m/z:* $[M+H]^+$ calcd for $C_{13}H_8F_5N_2O_3^+$: 335.0450, found 335.0451.

5-(4-(*tert*-Butyl)benzylidene)-1,3-dimethylpyrimidine-2,4,6(1*H*,3*H*,5*H*)-trione (8c)

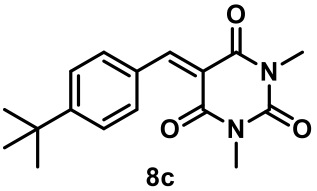

8c

Barbiturate **8c** was synthesized according to general procedure **A** and obtained as a white solid in a yield of 60%.

^{1}H-NMR (300 MHz, $CDCl_3$, 298 K) δ / ppm = 8.56 (s, 1 H, -C=C**H**), 8.11-8.08 (m, 2 H, Ar-**H**), 7.51-7.48 (m, 2 H, Ar-**H**), 3.42 (s, 3 H, -NC**H$_3$**), 3.39 (s, 3 H, -NC**H$_3$**), 1.35 (s, 9 H, -(C**H$_3$**)$_3$).

^{13}C-NMR (75 MHz, $CDCl_3$, 298 K) δ / ppm = 162.9, 160.7, 159.5, 157.7, 151.5, 134.3 (2 C), 130.1, 125.5 (2 C), 116.6, 35.5, 31.1 (3 C), 29.2, 28.6.

Melting point: 132.6-134.4 °C

HRMS (ESI-TOF): *m/z:* $[M+H]^+$ calcd for $C_{17}H_{21}N_2O_3^+$: 301.1547, found 301.1548.

1,3-Diisopropyl-5-(4-methoxybenzylidene)pyrimidine-2,4,6(1*H*,3*H*,5*H*)-trione (8d)

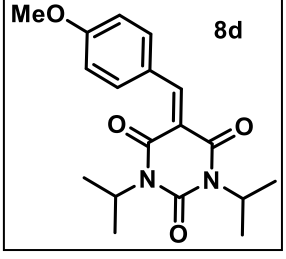

8d

Barbiturate **8d** was synthesized according to general procedure **B** and obtained as a yellowish solid in a yield of 64% after column chromatography on silica (heptanes/EtOAc, 5/1 to 1/1, as eluent).

^{1}H-NMR (300 MHz, $CDCl_3$, 298 K) δ / ppm = 8.43 (s, 1 H, -C=C**H**), 8.24-8.20 (m, 2 H, Ar-**H**), 6.99-6.94 (m, 2 H, Ar-**H**), 5.15 (sep, *J* = 6.9 Hz, 2 H, -NC**H**), 3.89 (s, 3 H, -OC**H$_3$**), 1.49 (d, *J* = 6.9 Hz 6 H, -(C**H**$_3$)$_2$), 1.48 (d, *J* = 6.9 Hz 6 H, -(C**H**$_3$)$_2$).

^{13}C-NMR (75 MHz, $CDCl_3$, 298 K) δ / ppm = 164.0, 163.2, 161.3, 157.9, 150.6, 137.5 (2 C), 125.9, 116.0, 114.0 (2 C), 55.7, 47.3, 46.6, 20.0 (2 C), 19.9 (2 C).

Melting point: 116.8-118.4 °C

HRMS (ESI-TOF): *m/z:* $[M+H]^+$ calcd for $C_{18}H_{23}N_2O_4^+$: 331.1652, found 331.1651.

1,3-Dibenzyl-5-(4-methoxybenzylidene)pyrimidine-2,4,6(1*H*,3*H*,5*H*)-trione (8e)

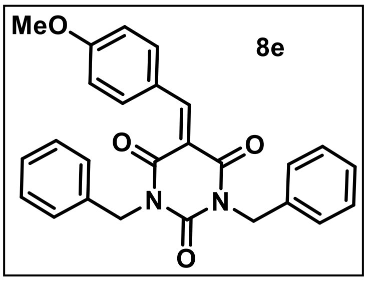

Barbiturate **8e** was synthesized according to general procedure **B** and obtained as a slightly yellowish solid in a yield of 61% after column chromatography on silica (heptanes/EtOAc, 1/1, as eluent).

^{1}H-NMR (300 MHz, $CDCl_3$, 298 K) δ / ppm = 8.54 (s, 1 H, -C=C**H**), 8.34-8.29 (m, 2 H, Ar-**H**), 7.47-7.45 (m, 4 H, Ar-**H**), 7.35-7.24 (m, 6 H, Ar-**H**), 7.00-6.95 (m, 2 H, Ar-**H**), 5.16 (s, 4 H, 2x -C**H$_2$**), 3.90 (s, 3 H, -OC**H$_3$**).

^{13}C-NMR (75 MHz, $CDCl_3$, 298 K) δ / ppm = 164.6, 163.0, 160.8, 159.7, 151.3, 138.3 (2 C), 136.9, 136.8, 129.0 (2 C), 128.9 (2 C), 128.7 (2 C), 128.6 (2 C), 127.9, 127.8, 125.7, 114.4, 114.2 (2 C), 55.8, 45.8, 45.0.

Melting point: 219.7-223.7 °C

HRMS (ESI-TOF): *m/z:* $[M+H]^+$ calcd for $C_{26}H_{23}N_2O_4^+$: 427.1652, found 427.1654.

5-(4-Methoxybenzylidene)-1,3-diphenylpyrimidine-2,4,6(1*H*,3*H*,5*H*)-trione (8f)

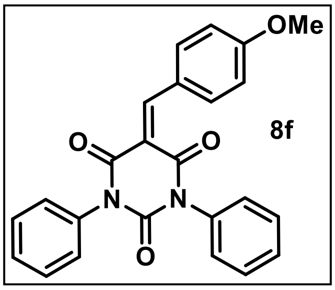

Barbiturate **8f** was synthesized according to general procedure **B** and obtained as a yellowish solid in a yield of 70% after column chromatography on silica (heptanes/EtOAc, 10/1, as eluent).

^{1}H-NMR (300 MHz, $CDCl_3$, 298 K) δ / ppm = 8.66 (s, 1 H, -C=C**H**), 8.39 (d, *J* = 8.9 Hz, 2 H, Ar-**H**), 7.53-7.44 (m, 6 H, Ar-**H**), 7.34-7.31 (m, 4 H, Ar-**H**), 6.95 (d, *J* = 8.9 Hz, 2 H, Ar-**H**), 7.00-6.95 (m, 2 H, Ar-**H**), 3.89 (s, 3 H, -OC**H$_3$**).

^{13}C-NMR (75 MHz, $CDCl_3$, 298 K) δ / ppm = 165.0, 163.3, 161.2, 160.7, 150.9, 139.0 (2 C), 135.2, 134.9, 129.5 (2 C), 129.4 (2 C), 129.1, 129.0, 128.8 (2 C), 128.6 (2 C), 125.6, 114.3 (2 C), 114.2, 55.8.

Melting point: 219.7-222.7 °C

HRMS (ESI-TOF): *m/z:* $[M+H]^+$ calcd for $C_{24}H_{19}N_2O_4^+$: 399.1339, found 399.1340.

6. Product characterization: 6-membered heterocycles

Ethyl (*R,Z*)-2-(6-phenyl-4-(trifluoromethyl)-3,4-dihydro-2*H*-pyran-2-ylidene)acetate (10a)

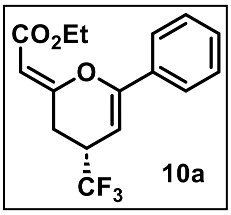

Product **10a** was synthesized according to general procedure **D** on a scale of 0.1 mmol. The compound was obtained as a colorless viscous residue in an isolated yield of 74% (23.1 mg with **ITU1**) and 84% (26.2 mg with **ISeU**) after preparative TLC (heptanes/EtOAc, 2/1, as eluent). The determined *e.r.* were >99:1 (**ITU1**) and >99:1 (**ISeU**).

^{1}H-NMR (300 MHz, $CDCl_3$, 298 K) δ / ppm = 7.83-7.80 (m, 2 H, Ar-**H**), 7.44-7.37 (m, 3 H, Ar-**H**), 5.57 (d, J = 4.0 Hz, 1 H, -C=C**H**), 5.19 (s, 1 H, -C=C**H**), 4.23 (q, J = 7.1 Hz, 2 H, -C**H₂**), 3.27-3.19 (m, 1 H, -C**H**), 2.80-2.67 (m, 2 H, -C**H₂**), 1.32 (t, J = 7.1 Hz, 3 H, -C**H₃**).

^{13}C-NMR (75 MHz, $CDCl_3$, 298 K) δ / ppm = 164.5, 158.4, 152.6, 132.5, 129.7, 128.7, 126.1 (q, J = 279.2 Hz, 1 C), 125.3, 99.8, 91.4 (bs, 1 C), 60.1, 36.0 (q, J = 29.4 Hz, 1 C), 26.5 (bs, 1 C), 14.5.

^{19}F-NMR (282 MHz, $CDCl_3$, 298 K) δ / ppm = -72.7 (3 F, -C**F₃**).

HRMS (ESI-TOF): *m/z:* $[M+H]^+$ calcd for $C_{16}H_{16}F_3O_3^+$: 313.1046, found 313.1045.

$[\alpha]_D^{20}$(c = 1, $CHCl_3$, *e.r.* 99:1): +40.5

HPLC (CHIRALCEL® OD-H, *n*-hexane/IPA = 4/1, flow = 0.5 mL min^{-1}, T_{Column} = 10 °C, l = 220 nm) t_r: 11.9 min (major), 14.1 min (minor).

Ethyl (*R,Z*)-2-(6-(4-methoxyphenyl)-4-(trifluoromethyl)-3,4-dihydro-2*H*-pyran-2-ylidene) acetate (10b)

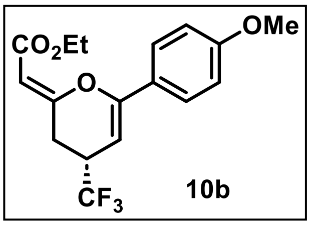

Product **10b** was synthesized according to general procedure **D** on a scale of 0.1 mmol. The compound was obtained as a colorless viscous residue in an isolated yield of 79% (27.0 mg with **ITU1**) and 83% (28.4 mg with **ISeU**) after preparative TLC (heptanes/EtOAc, 2/1, as eluent). The determined *e.r.* were >99:1 (**ITU1**) and >99:1 (**ISeU**).

^{1}H-NMR (300 MHz, $CDCl_3$, 298 K) δ / ppm = 7.78-7.73 (m, 2 H, Ar-**H**), 6.95-6.90 (m, 2 H, Ar-**H**), 5.42 (d, J = 4.0 Hz, 1 H, -C=C**H**), 5.17 (s, 1 H, -C=C**H**), 4.22 (q, J = 7.1 Hz, 2 H, -C**H₂**), 3.82 (s, 3 H, -OC**H₃**), 3.28-3.13 (m, 1 H, -C**H**), 2.78-2.65 (m, 2 H, -C**H₂**), 1.33 (t, J = 7.1 Hz, 3 H, -C**H₃**).

^{13}C-NMR (75 MHz, $CDCl_3$, 298 K) δ / ppm = 164.6, 160.8, 158.7, 152.4, 126.8 (2 C), 126.2 (q, J = 279.5 Hz, 1 C), 125.1, 114.0 (2 C), 99.5, 89.4 (bs, 1 C), 60.0, 55.5, 36.0 (q, J = 29.5 Hz, 1 C), 26.6 (bs, 1 C), 14.5.

^{19}F-NMR (282 MHz, $CDCl_3$, 298 K) δ / ppm = -72.8 (3 F, -C**F₃**).

HRMS (ESI-TOF): *m/z:* $[M+H]^+$ calcd for $C_{17}H_{18}F_3O_4^+$: 343.1152, found 343.1152.

$[\alpha]_D^{20}$(c = 1, $CHCl_3$, *e.r.* 99:1): +19.6

HPLC (CHIRALCEL® OD-H, *n*-hexane/IPA = 4/1, flow = 0.5 mL min^{-1}, T_{Column} = 10 °C, *l* = 220 nm) t_r: 20.8 min (minor), 22.9 min (major).

Ethyl (*R,Z*)-2-(6-(4-nitrophenyl)-4-(trifluoromethyl)-3,4-dihydro-2*H*-pyran-2-ylidene)acetate (10c)

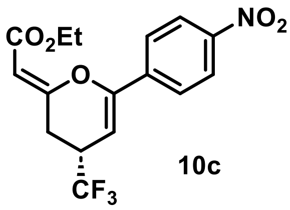

10c

Product **10c** was synthesized according to general procedure **D** on a scale of 0.1 mmol. The compound was obtained as a colorless viscous residue in an isolated yield of 55% (19.6 mg with **ITU1**) and 64% (22.8 mg with **ISeU**) after preparative TLC (heptanes/EtOAc, 2/1, as eluent). The determined *e.r.* were 99:1 (**ITU1**) and >99:1 (**ISeU**).

^{1}**H-NMR** (300 MHz, $CDCl_3$, 298 K) δ / ppm = 8.28-8.24 (m, 2 H, Ar-**H**), 8.02-7.97 (m, 2 H, Ar-**H**), 5.76 (d, *J* = 4.1 Hz, 1 H, -C=C**H**), 5.26 (s, 1 H, -C=C**H**), 4.23 (q, *J* = 7.1 Hz, 2 H, -C**H**$_2$), 3.36-3.22 (m, 1 H, -C**H**), 2.84-2.70 (m, 2 H, -C**H**$_2$), 1.33 (t, *J* = 7.1 Hz, 3 H, -C**H**$_3$).

^{13}**C-NMR** (75 MHz, $CDCl_3$, 298 K) δ / ppm = 164.2, 157.5, 150.8, 148.4, 138.3, 126.2, 125.8 (q, *J* = 279.5 Hz, 1 C), 124.0, 100.6, 95.1 (q, *J* = 2.9 Hz, 1 C), 60.2, 36.2 (q, *J* = 29.7 Hz, 1 C), 26.1 (q, *J* = 2.9 Hz, 1 C), 14.5.

^{19}**F-NMR** (282 MHz, $CDCl_3$, 298 K) δ / ppm = -72.4 (3 F, -C**F**$_3$).

HRMS (ESI-TOF): *m/z:* $[M+H]^+$ calcd for $C_{16}H_{15}F_3NO_5^+$: 358.0897, found 358.0897.

$[\alpha]_D^{20}$(c = 1, $CHCl_3$, *e.r.* 99:1): +20.9

HPLC (CHIRALCEL® OD-H, *n*-hexane/IPA = 2/1, flow = 0.5 mL min^{-1}, T_{Column} = 10 °C, *l* = 220 nm) t_r: 12.9 min (minor), 27.8 min (major).

Ethyl (*R,Z*)-2-(6-(4-fluorophenyl)-4-(trifluoromethyl)-3,4-dihydro-2*H*-pyran-2-ylidene)acetate (10d)

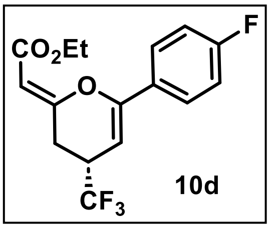

10d

Product **10d** was synthesized according to general procedure **D** on a scale of 0.1 mmol. The compound was obtained as a colorless viscous residue in an isolated yield of 76% (25.1 mg with **ITU1**) and 87% (28.7 mg with **ISeU**) after preparative TLC (heptanes/EtOAc, 2/1, as eluent). The determined *e.r.* were >99:1 (**ITU1**) and >99:1 (**ISeU**).

^{1}**H-NMR** (300 MHz, $CDCl_3$, 298 K) δ / ppm = 7.84-7.77 (m, 2 H, Ar-**H**), 7.13-7.05 (m, 2 H, Ar-**H**), 5.49 (d, *J* = 4.0 Hz, 1 H, -C=C**H**), 5.20 (s, 1 H, -C=C**H**), 4.22 (q, *J* = 7.1 Hz, 2 H, -C**H**$_2$), 3.29-3.15 (m, 1 H, -C**H**), 2.80-2.67 (m, 2 H, -C**H**$_2$), 1.32 (t, *J* = 7.1 Hz, 3 H, -C**H**$_3$).

^{13}C-NMR (75 MHz, $CDCl_3$, 298 K) *δ* / ppm = 165.3, 163.7 (d, *J* = 249.5 Hz, 1 C), 158.3, 151.8, 128.7 (d, *J* = 3.2 Hz, 1 C), 127.3 (d, *J* = 8.3 Hz, 2 C), 126.1 (q, *J* = 279.3 Hz, 1 C), 115.7 (d, *J* = 21.9 Hz, 2 C), 99.9, 91.0 (bs, 1 C), 60.1, 36.0 (q, *J* = 29.7 Hz, 1 C), 26.4 (bs, 1 C), 14.5.

^{19}F-NMR (282 MHz, $CDCl_3$, 298 K) *δ* / ppm = -72.7 (3 F, -C**F_3**), -111.3 (1 F, Ar-**F**).

HRMS (ESI-TOF): *m/z:* $[M+H]^+$ calcd for $C_{16}H_{15}F_4O_3^+$: 331.0952, found 331.0952.

$[\alpha]_D^{20}$(c = 1, $CHCl_3$, *e.r.* 99:1): +38.4

HPLC (CHIRALCEL® OD-H, *n*-hexane/IPA = 4/1, flow = 0.5 mL min^{-1}, T_{Column} = 10 °C, *l* = 220 nm) t_r: 10.9 min (minor), 15.0 min (major).

Ethyl (*R,Z*)-2-(6-(*p*-tolyl)-4-(trifluoromethyl)-3,4-dihydro-2*H*-pyran-2-ylidene)acetate (10e)

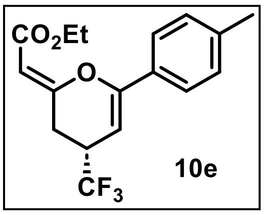

Product **10e** was synthesized according to general procedure **D** on a scale of 0.1 mmol. The compound was obtained as a colorless viscous residue in an isolated yield of 78% (25.4 mg with **ITU1**) and 84% (27.4 mg with **ISeU**) after preparative TLC (heptanes/EtOAc, 2/1, as eluent). The determined *e.r.* were >99:1 (**ITU1**) and >99:1 (**ISeU**).

^{1}H-NMR (300 MHz, $CDCl_3$, 298 K) *δ* / ppm = 7.70 (d, *J* = 8.2 Hz, 2 H, Ar-**H**), 7.21 (d, *J* = 8.2 Hz, 2 H, Ar-**H**), 5.51 (d, *J* = 4.0 Hz, 1 H, -C=C**H**), 5.18 (s, 1 H, -C=C**H**), 4.23 (q, *J* = 7.2 Hz, 2 H, -C**H_2**), 3.28-3.14 (m, 1 H, -C**H**), 2.79-2.65 (m, 2 H, -C**H_2**), 2.37 (s, 3 H, -C**H_3**), 1.33 (t, *J* = 7.2 Hz, 3 H, -C**H_3**).

^{13}C-NMR (75 MHz, $CDCl_3$, 298 K) *δ* / ppm = 164.6, 158.5, 152.7, 139.8, 129.7, 129.2 (2 C), 126.2 (q, *J* = 279.3 Hz, 1 C), 124.9 (2 C), 99.6, 90.5 (q, *J* = 2.8 Hz, 1 C), 60.1, 36.0 (q, *J* = 29.4 Hz, 1 C), 26.6 (q, *J* = 2.8 Hz, 1 C), 21.5, 14.5.

^{19}F-NMR (282 MHz, $CDCl_3$, 298 K) *δ* / ppm = -72.8 (3 F, -C**F_3**).

HRMS (ESI-TOF): *m/z:* $[M+H]^+$ calcd for $C_{17}H_{18}F_3O_3^+$: 327.1203, found 327.1205.

$[\alpha]_D^{20}$(c = 1, $CHCl_3$, *e.r.* 99:1): +25.6

HPLC (YMC-SB, *n*-hexane/IPA = 4/1, flow = 1.0 mL min^{-1}, T_{Column} = 10 °C, *l* = 210 nm) t_r: 6.3 min (major), 7.5 min (minor).

Ethyl (*R,Z*)-2-(6-(o-tolyl)-4-(trifluoromethyl)-3,4-dihydro-2*H*-pyran-2-ylidene)acetate (10f)

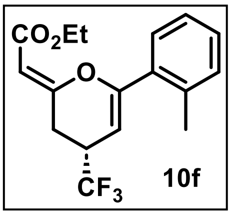

Product **10f** was synthesized according to general procedure **D** on a scale of 0.1 mmol. The compound was obtained as a colorless viscous residue in an isolated yield of 71% (23.1 mg with **ITU1**) and 72% (23.5 mg with **ISeU**) after preparative TLC (heptanes/EtOAc, 2/1, as eluent). The determined *e.r.* were >99:1 (**ITU1**) and 99:1 (**ISeU**).

^{1}H-NMR (300 MHz, $CDCl_3$, 298 K) δ / ppm = 7.45-7.42 (m, 1 H, Ar-**H**), 7.29-7.24 (m, 1 H, Ar-**H**), 7.20-7.16 (m, 2 H, Ar-**H**), 5.15-5.12 (m, 2 H, -C=C**H**, -C=C**H**), 4.13 (q, J = 7.1 Hz, 2 H, -C**H$_2$**), 3.25-3.12 (m, 1 H, -C**H**), 2.80-2.66 (m, 2 H, -C**H$_2$**), 2.43 (s, 3 H, -C**H$_3$**), 1.22 (t, J = 7.1 Hz, 3 H, -C**H$_3$**).

^{13}C-NMR (75 MHz, $CDCl_3$, 298 K) δ / ppm = 164.3, 158.3, 154.5, 137.0, 133.4, 130.8, 129.6, 129.3, 126.1 (q, J = 279.3 Hz, 1 C), 125.8, 99.6, 95.9 (q, J = 3.3 Hz, 1 C), 60.0, 36.0 (q, J = 29.4 Hz, 1 C), 26.7 (q, J = 2.8 Hz, 1 C), 20.6, 14.4.

^{19}F-NMR (282 MHz, $CDCl_3$, 298 K) δ / ppm = -72.8 (3 F, -C**F$_3$**).

HRMS (ESI-TOF): *m/z:* $[M+H]^+$ calcd for $C_{17}H_{18}F_3O_3^+$: 327.1203, found 327.1201.

$[\alpha]_D^{20}$(c = 1, $CHCl_3$, *e.r.* 99:1): +28.4

HPLC (CHIRALCEL® OD-H, *n*-hexane/IPA = 4/1, flow = 0.5 mL min^{-1}, T_{Column} = 10 °C, *l* = 220 nm) t_r: 12.1 min (minor), 13.4 min (major).

Ethyl (*R,Z*)-2-(6-(thiophen-2-yl)-4-(trifluoromethyl)-3,4-dihydro-2*H*-pyran-2-ylidene)acetate (10g)

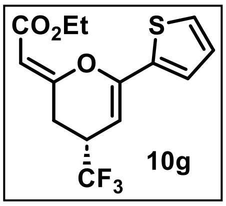

Product **10g** was synthesized according to general procedure **D** on a scale of 0.1 mmol. The compound was obtained as a slightly beige viscous residue in an isolated yield of 39% (12.4 mg with **ITU1**) and 70% (22.3 mg with **ISeU**) after preparative TLC (heptanes/EtOAc, 2/1, as eluent). The determined *e.r.* were >99:1 (**ITU1**) and >99:1 (**ISeU**).

^{1}H-NMR (300 MHz, $CDCl_3$, 298 K) δ / ppm = 7.51 (dd, J_1 = 3.7 Hz, J_2 = 1.0 Hz, 1 H, Ar-**H**), 7.30 (dd, J_1 = 5.0 Hz, J_2 = 1.0 Hz, 1 H, Ar-**H**), 7.04 (dd, J_1 = 5.0 Hz, J_2 = 3.7 Hz, 1 H, Ar-**H**), 5.42 (d, J = 4.2 Hz, 1 H, -C=C**H**), 5.18 (s, 1 H, -C=C**H**), 4.22 (q, J = 7.2 Hz, 2 H, -C**H$_2$**), 3.27-3.13 (m, 1 H, -C**H**), 2.80-2.66 (m, 2 H, -C**H$_2$**), 1.33 (t, J = 7.2 Hz, 3 H, -C**H$_3$**).

^{13}C-NMR (75 MHz, $CDCl_3$, 298 K) δ / ppm = 164.4, 157.8, 148.5, 136.0, 127.7, 126.6, 126.1 (q, J = 279.4 Hz, 1 C), 126.0, 100.1, 90.3 (bs, 1 C), 60.1, 35.9 (q, J = 29.7 Hz, 1 C), 26.5 (bs, 1 C), 14.5.

^{19}F-NMR (282 MHz, $CDCl_3$, 298 K) δ / ppm = -72.7 (3 F, -C**F$_3$**).

HRMS (ESI-TOF): *m/z:* $[M+H]^+$ calcd for $C_{14}H_{14}F_3O_3S^+$: 313.0610, found 313.0612.

$[\alpha]_D^{20}$(c = 1, $CHCl_3$, *e.r.* 99:1): +10.9

HPLC (CHIRALCEL® OD-H, *n*-hexane/IPA = 4/1, flow = 0.5 mL min^{-1}, T_{Column} = 10 °C, *l* = 220 nm) t_r: 13.3 min (minor), 14.2 min (major).

Ethyl (*R,Z*)-2-(6-(naphthalen-2-yl)-4-(trifluoromethyl)-3,4-dihydro-2*H*-pyran-2-ylidene)acetate (10h)

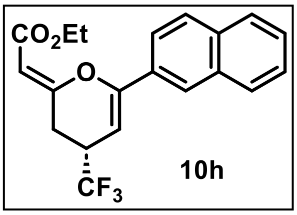

Product **10h** was synthesized according to general procedure **D** on a scale of 0.1 mmol. The compound was obtained as a colorless viscous residue in an isolated yield of 79% (28.6 mg with **ITU1**) and 86% (31.1 mg with **ISeU**) after preparative TLC (heptanes/EtOAc, 2/1, as eluent). The determined *e.r.* were >99:1 (**ITU1**) and >99:1 (**ISeU**).

^{1}H-NMR (300 MHz, $CDCl_3$, 298 K) δ / ppm = 8.38 (bs, 1 H, Ar-**H**), 7.95-7.79 (m, 4 H, Ar-**H**), 7.54-7.48 (m, 2 H, Ar-**H**), 5.70 (d, *J* = 4.0 Hz, 1 H, -C=C**H**), 5.23 (s, 1 H, -C=C**H**), 4.29 (q, *J* = 7.1 Hz, 2 H, -C**H$_2$**), 3.35-3.21 (m, 1 H, -C**H**), 2.84-2.71 (m, 2 H, -C**H$_2$**), 1.38 (t, *J* = 7.1 Hz, 3 H, -C**H$_3$**).

^{13}C-NMR (75 MHz, $CDCl_3$, 298 K) δ / ppm = 164.6, 158.3, 152.6, 133.9, 133.2, 129.7, 129.0, 128.4, 127.8, 126.9, 126.6, 126.2 (q, *J* = 279.5 Hz, 1 C), 125.1, 122.4, 99.9, 92.0 (q, *J* = 3.3 Hz, 1 C), 60.1, 36.2 (q, *J* = 29.2 Hz, 1 C), 26.6 (q, *J* = 2.8 Hz, 1 C), 14.6.

^{19}F-NMR (282 MHz, $CDCl_3$, 298 K) δ / ppm = -72.6 (3 F, -C**F$_3$**).

HRMS (ESI-TOF): *m/z:* $[M+H]^+$ calcd for $C_{20}H_{18}F_3O_3^+$: 363.1203, found 363.1203.

$[\alpha]_D^{20}$(c = 1, $CHCl_3$, *e.r.* 99:1): +25.1

HPLC (CHIRALCEL® OD-H, *n*-hexane/IPA = 4/1, flow = 0.5 mL min^{-1}, T_{Column} = 10 °C, *l* = 272 nm) t_r: 16.2 min (minor), 20.4 min (major).

Benzyl (*R,Z*)-2-(6-phenyl-4-(trifluoromethyl)-3,4-dihydro-2H-pyran-2-ylidene)acetate (10i)

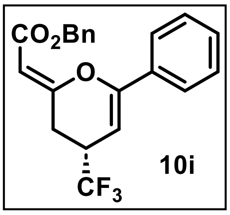

Product **10i** was synthesized according to general procedure **D** on a scale of 0.1 mmol. The compound was obtained as a colorless viscous residue in an isolated yield of 76% (28.3 mg with **ITU1**) and 76% (28.4 mg with **ISeU**) after preparative TLC (heptanes/EtOAc, 2/1, as eluent). The determined *e.r.* were >99:1 (**ITU1**) and >99:1 (**ISeU**).

^{1}H-NMR (300 MHz, $CDCl_3$, 298 K) δ / ppm = 7.76-7.73 (m, 2 H, Ar-**H**), 7.43-7.30 (m, 8 H, Ar-**H**), 5.57 (d, *J* = 4.0 Hz, 1 H, -C=C**H**), 5.26 (s, 1 H, -C=C**H**), 5.23 (s, 2 H, -C**H$_2$**), 3.27-3.19 (m, 1 H, -C**H**), 2.81-2.67 (m, 2 H, -C**H$_2$**).

^{13}C-NMR (75 MHz, $CDCl_3$, 298 K) δ / ppm = 164.3, 158.9, 152.6, 136.3, 132.3, 129.6, 128.7 (2 C), 128.6 (2 C), 128.5 (2 C), 128.3, 126.1 (q, *J* = 279.3 Hz, 1 C), 125.3 (2 C), 99.4, 91.5 (q, *J* = 3.3 Hz, 1 C), 66.0, 36.0 (q, *J* = 29.7 Hz, 1 C), 26.6 (q, *J* = 2.8 Hz, 1 C).

^{19}F-NMR (282 MHz, $CDCl_3$, 298 K) δ / ppm = -72.7 (3 F, -C**F$_3$**).

HRMS (ESI-TOF): *m/z:* $[M+H]^+$ calcd for $C_{21}H_{18}F_3O_3^+$: 375.1203, found 375.1202.

$[\alpha]_D^{20}$(c = 1, $CHCl_3$, *e.r.* 99:1): +29.6

HPLC (CHIRALCEL® OD-H, *n*-hexane/IPA = 4/1, flow = 0.5 mL min^{-1}, T_{Column} = 10 °C, *l* = 220 nm) t_r: 16.0 min (major), 26.7 min (minor).

Diethyl (*R*,*Z*)-2-(6-phenyl-4-(trifluoromethyl)-3,4-dihydro-2*H*-pyran-2-ylidene)succinate (11a)

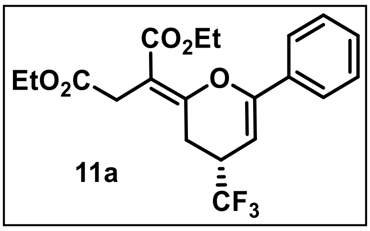

Product **11a** was synthesized according to general procedure **D** on a scale of 0.1 mmol. The compound was obtained as a colorless viscous residue in an isolated yield of 60% (23.8 mg with **ITU1**) and 77% (30.5 mg with **ISeU**) after preparative TLC (heptanes/EtOAc, 5/1, as eluent). The determined *e.r.* were >99:1 (**ITU1**) and >99:1 (**ISeU**).

^{1}H-NMR (300 MHz, $CDCl_3$, 298 K) δ / ppm = 7.81 – 7.75 (m, 2 H, Ar-H), 7.42 – 7.35 (m, 3 H, Ar-H), 5.54 (d, *J* = 3.8 Hz, 1 H, =CH), 4.29 (q, *J* = 7.1 Hz, 2 H, CH_2), 4.17 (q, *J* = 7.1 Hz, 2 H, CH_2), 3.47 (d, *J* = 17.2 Hz, 1 H, CH_2-COO), 3.35 (d, *J* = 17.2 Hz, 1 H, CH_2-COO), 3.29 – 3.21 (m, 1 H, CH-CF_3), 2.87 (dd, J_1 = 15.0 Hz, J_2 = 6.0 Hz, 1 H, =C-CH_2), 2.76 (dd, J_1 = 15.0 Hz, J_2= 8.2 Hz, 1 H, =C-CH_2), 1.33 (t, *J* = 7.1 Hz, 3 H, CH_3), 1.27 (t, *J* = 7.1 Hz, 3 H, CH_3).

^{13}C-NMR (75 MHz, $CDCl_3$, 298 K) δ / ppm = 171.0, 166.1, 155.3, 153.0, 132.7, 129.6, 128.5 (2 C), 126.2 (q, $^1J_{C\text{-}F}$ = 279.4 Hz), 125.4 (2 C), 105.9, 91.1 (q, $^3J_{C\text{-}F}$ = 3.1 Hz), 61.2, 61.0, 36.2 (q, $^2J_{C\text{-}F}$ = 29.3 Hz), 34.0, 23.3 (q, $^3J_{C\text{-}F}$ = 3.0 Hz), 14.4, 14.3.

^{19}F-NMR (282 MHz, $CDCl_3$, 298 K) δ / ppm = -72.8.

HRMS (ESI-TOF): *m/z:* $[M+H]^+$ calcd for $C_{20}H_{22}F_3O_5^+$: 399.1414, found 399.1414.

$[\alpha]_D^{20}$(c = 1, $CHCl_3$, *e.r.* 99:1): +47.1.

HPLC (YMC-SB, *n*-hexane/IPA = 4/1, flow = 1.0 mL min^{-1}, T_{Column} = 20 °C, *l* = 269 nm) t_r: 6.3 min (major), 7.4 min (minor); (YMC-SB, *n*-hexane/IPA = 7/3, flow = 1.0 mL min^{-1}, T_{Column} = 20 °C, *l* = 269 nm) t_r: 5.5 min (major), 6.4 min (minor)

Diethyl (*R*,Z)-2-(6-(4-methoxyphenyl)-4-(trifluoromethyl)-3,4-dihydro-2H-pyran-2-ylidene)succinate (11b)

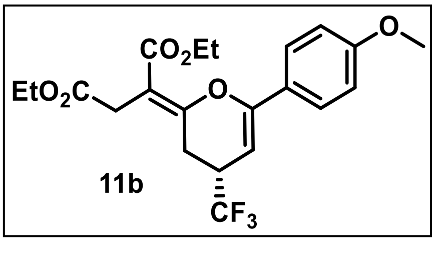

Product **11b** was synthesized according to general procedure **D** on a scale of 0.1 mmol. The compound was obtained as a colorless viscous residue in a yield of 60% (25.7 mg with **ITU1**) and 73% (31.3 mg with **ISeU**) after preparative TLC (heptanes/EtOAc, 5/1, as eluent). The determined *e.r.* were >99:1 (**ITU1**) and >99:1 (**ISeU**).

^{1}H-NMR (300 MHz, $CDCl_3$, 298 K) δ / ppm = 7.74 – 7.69 (m, 2 H, Ar-H), 6.93 – 6.88 (m, 2 H, Ar-H), 5.40 (d, *J* = 3.8 Hz, 1 H, =CH), 4.28 (q, *J* = 7.1 Hz, 2 H, CH_2), 4.16 (q, *J* = 7.1 Hz, 2 H, CH_2), 3.83 (s, 3 H,

O-CH_3), 3.45 (d, J = 17.2 Hz, 1 H, CH_2-COO), 3.33 (d, J = 17.2 Hz, 1 H, CH_2-COO), 3.28 – 3.18 (m, 1 H, CH-CF_3), 2.85 (dd, J_1 = 15.0 Hz, J_2 = 6.0 Hz, 1 H, =C-CH_2), 2.77 (dd, J_1 = 15.0 Hz, J_2 = 8.0 Hz, 1 H, =C-CH_2), 1.33 (t, J = 7.1 Hz, 3 H, CH_3), 1.26 (t, J = 7.1 Hz, 3 H, CH_3).

^{13}C-NMR (75 MHz, $CDCl_3$, 298 K) δ / ppm = 171.1, 166.1, 160.7, 155.6, 152.7, 126.8 (2 C), 126.3 (q, $^1J_{C\text{-}F}$ = 279.5 Hz), 125.4, 113.9 (2 C), 105.6, 89.2 (q, $^3J_{C\text{-}F}$ = 3.1 Hz), 61.2, 60.9, 55.4, 36.1 (q, $^2J_{C\text{-}F}$ = 29.3 Hz), 34.0, 23.3 (q, $^3J_{C\text{-}F}$ = 2.9 Hz), 14.4, 14.3.

^{19}F-NMR (282 MHz, $CDCl_3$, 298 K) δ / ppm = -72.9.

HRMS (ESI-TOF): *m/z:* $[M+H]^+$ calcd for $C_{21}H_{24}F_3O_6^+$: 429.1519, found 429.1520.

$[\alpha]_D^{20}$(c = 1, $CHCl_3$, *e.r.* 99:1): +20.7.

HPLC (YMC-SA, *n*-hexane/IPA = 7/3, flow = 1.0 mL min^{-1}, T_{Column} = 20 °C, l = 269 nm) t_r: 5.5 min (minor), 6.0 min (major).

Diethyl (*Z*)-2-(6-(4-fluorophenyl)-4-(trifluoromethyl)-3,4-dihydro-2*H*-pyran-2-ylidene)succinate (11c)

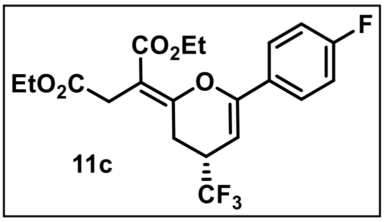

Product **11c** was synthesized according to general procedure **D** on a scale of 0.1 mmol. The compound was obtained as a colorless viscous residue in a yield of 67% (28.2 mg with **ITU1**) and 70% (29.2 mg with **ISeU**) after preparative TLC (heptanes/EtOAc, 5/1, as eluent). The determined *e.r.* were >99:1 (**ITU1**) and >99:1 (**ISeU**).

^{1}H-NMR (300 MHz, $CDCl_3$, 298 K) δ / ppm = 7.81 – 7.74 (m, 2 H, Ar-H), 7.12 – 7.03 (m, 2 H, Ar-H), 5.47 (d, J = 3.8 Hz, 1 H, =CH), 4.27 (q, J = 7.1 Hz, 2 H, CH_2), 4.17 (q, J = 7.1 Hz, 2 H, CH_2), 3.46 (d, J = 17.2 Hz, 1 H, CH_2-COO), 3.33 (d, J = 17.2 Hz, 1 H, CH_2-COO), 3.30 – 3.20 (m, 1 H, CH-CF_3), 2.86 (dd, J_1 = 15.0 Hz, J_2 = 6.0 Hz, 1 H, =C-CH_2), 2.75 (dd, J_1 = 15.0 Hz, J_2 = 8.1 Hz, 1 H, =C-CH_2), 1.32 (t, J = 7.1 Hz, 3 H, CH_3), 1.27 (t, J = 7.1 Hz, 3 H, CH_3).

^{13}C-NMR (75 MHz, $CDCl_3$, 298 K) δ / ppm = 171.0, 165.9, 163.6 (d, $^1J_{C\text{-}F}$ = 249.3 Hz), 155.3, 152.2, 129.0 (d, $^4J_{C\text{-}F}$ = 3.2 Hz), 127.4 (d, $^3J_{C\text{-}F}$ = 8.2 Hz, 2 C), 126.2 (q, $^1J_{C\text{-}F}$ = 279.4 Hz), 115.6 (d, $^2J_{C\text{-}F}$ = 21.7 Hz, 2 C), 106.0, 90.8 (q, $^3J_{C\text{-}F}$ = 1.6 Hz), 61.3, 61.0, 36.1 (q, $^2J_{C\text{-}F}$ = 29.3 Hz), 34.0, 23.2 (q, $^3J_{C\text{-}F}$ = 2.9 Hz), 14.4, 14.3.

^{19}F-NMR (282 MHz, $CDCl_3$, 298 K) δ / ppm = -72.8 (CF_3), -111.5 (ArC-F).

HRMS (ESI-TOF): *m/z:* $[M+H]^+$ calcd for $C_{20}H_{21}F_4O_5^+$: 417.1320, found 417.1320.

$[\alpha]_D^{20}$(c = 1, $CHCl_3$, *e.r.* 99:1): +30.6.

HPLC (YMC-SB, *n*-hexane/IPA = 7/3, flow = 0.5 mL min^{-1}, T_{Column} = 20 °C, l = 269 nm) t_r: 11.2 min (major), 11.7 min (minor).

Diethyl (*R*,*Z*)-2-(6-(*p*-tolyl)-4-(trifluoromethyl)-3,4-dihydro-2*H*-pyran-2-ylidene)succinate (11d)

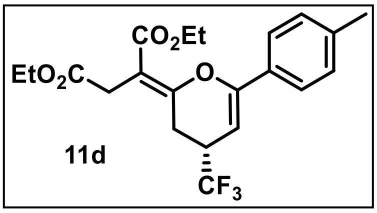

Product **11d** was synthesized according to general procedure **D** on a scale of 0.1 mmol. The compound was obtained as a colorless viscous residue in a yield of 72% (29.7 mg with **ITU1**) and 76% (31.3 mg with **ISeU**) after preparative TLC (heptanes/EtOAc, 5/1, as eluent). The determined *e.r.* were >99:1 (**ITU1**) and >99:1 (**ISeU**).

^{1}H-NMR (300 MHz, $CDCl_3$, 298 K) δ / ppm = 7.66 (d, *J* = 8.2 Hz, 2 H, Ar-H), 7.19 (d, *J* = 8.2 Hz, 2 H, Ar-H), 5.48 (d, *J* = 3.8 Hz, 1 H, =CH), 4.29 (q, *J* = 7.1 Hz, 2 H, CH_2), 4.17 (q, *J* = 7.1 Hz, 2 H, CH_2), 3.46 (d, *J* = 17.2 Hz, 1 H, CH_2-COO), 3.34 (d, *J* = 17.2 Hz, 1 H, CH_2-COO), 3.29 – 3.20 (m, 1 H, CH-CF_3), 2.86 (dd, J_1 = 15.0 Hz, J_2 = 6.0 Hz, 1 H, =C-CH_2), 2.76 (dd, J_1 = 15.0 Hz, J_2 = 8.1 Hz, 1 H, =C-CH_2), 2.37 (s, 3 H, ArC-CH_3), 1.33 (t, *J* = 7.1 Hz, 3 H, CH_3), 1.27 (t, *J* = 7.1 Hz, 3 H, CH_3).

^{13}C-NMR (75 MHz, $CDCl_3$, 298 K) δ / ppm = 171.0, 166.2, 155.4, 153.0, 139.6, 130.0, 129.2 (2 C), 126.3 (q, $^1J_{C\text{-}F}$ = 279.3 Hz), 125.3 (2 C), 105.7, 90.2 (q, $^3J_{C\text{-}F}$ = 3.1 Hz), 61.2, 61.0, 36.1 (q, $^2J_{C\text{-}F}$ = 29.3 Hz), 34.0, 23.3 (q, $^3J_{C\text{-}F}$ = 3.1 Hz), 21.4, 14.4, 14.3.

^{19}F-NMR (282 MHz, $CDCl_3$, 298 K) δ / ppm = -72.8.

HRMS (ESI-TOF): *m/z:* $[M+H]^+$ calcd for $C_{21}H_{24}F_3O_5^+$: 413.1570, found 413.1570.

$[\alpha]_D^{20}$(c = 1, $CHCl_3$, *e.r.* 99:1): +48.6.

HPLC (YMC-SB, *n*-hexane/IPA = 7/3, flow = 1.0 mL min^{-1}, T_{Column} = 20 °C, *l* = 269 nm) t_r: 5.6 min (major), 7.1 min (minor).

Diethyl (*R*,*Z*)-2-(6-(*o*-tolyl)-4-(trifluoromethyl)-3,4-dihydro-2*H*-pyran-2-ylidene)succinate (11e)

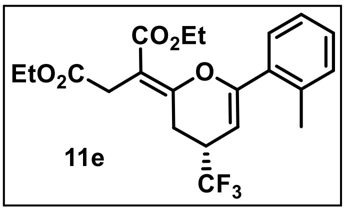

Product **11e** was synthesized according to general procedure **D** on a scale of 0.05 mmol. The compound was obtained as a colorless viscous residue in a yield of 75% (16.0 mg with **ITU1**) and 68% (14.1 mg with **ISeU**) after preparative TLC (heptanes/EtOAc, 5/1, as eluent). The determined *e.r.* were >99:1 (**ITU1**) and >99:1 (**ISeU**).

^{1}H-NMR (300 MHz, $CDCl_3$, 298 K) δ / ppm = 7.47 – 7.42 (m, 1 H, Ar-H), 7.31 – 7.24 (m, 1 H, Ar-H), 7.23 – 7.16 (m, 2 H, Ar-H), 5.11 (d, *J* = 3.7 Hz, 1 H, =CH), 4.17 (q, *J* = 7.1 Hz, 2 H, CH_2), 4.17 (q, *J* = 7.1 Hz, 2 H, CH_2), 3.44 (d, *J* = 17.1 Hz, 1 H, CH_2-COO), 3.32 (d, *J* = 17.1 Hz, 1 H, CH_2-COO), 3.27 – 3.19 (m, 1 H, CH-CF_3), 2.88 (dd, J_1 = 15.1 Hz, J_2 = 6.0 Hz, 1 H, =C-CH_2), 2.77 (dd, J_1 = 15.1 Hz, J_2 = 8.1 Hz, 1 H, =C-CH_2), 2.40 (s, 3 H, ArC-CH_3), 1.27 (t, *J* = 7.1 Hz, 3 H, CH_3), 1.15 (t, *J* = 7.1 Hz, 3 H, CH_3).

^{13}C-NMR (75 MHz, $CDCl_3$, 298 K) δ / ppm = 171.0, 166.3, 154.9, 154.4, 136.7, 133.7, 130.7, 129.4, 129.3, 126.3 (q, $^1J_{C\text{-}F}$ = 279.3 Hz), 125.7, 105.8, 95.5 (q, $^3J_{C\text{-}F}$ = 3.0 Hz), 61.2, 60.9, 36.1 (q, $^2J_{C\text{-}F}$ = 29.2 Hz), 34.0, 23.2 (q, $^3J_{C\text{-}F}$ = 2.9 Hz), 20.5, 14.3, 14.1.

^{19}F-NMR (282 MHz, $CDCl_3$, 298 K) *δ* / ppm = -73.0.

HRMS (ESI-TOF): *m/z:* $[M+H]^+$ calcd for $C_{21}H_{24}F_3O_5^+$: 413.1570, found 413.1570.

$[\alpha]_D^{20}$(c = 1, $CHCl_3$, *e.r.* 99:1): +25.6.

HPLC (YMC-SB, *n*-hexane/IPA = 10/1, flow = 1.0 mL min^{-1}, T_{Column} = 20 °C, *l* = 269 nm) t_r: 6.9 min (major), 7.5 min (minor).

Diethyl (*R*,*Z*)-2-(6-(thiophen-2-yl)-4-(trifluoromethyl)-3,4-dihydro-2*H*-pyran-2-ylidene)succinate (11f)

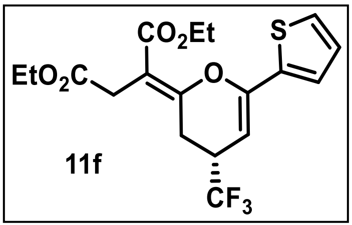

Product **11f** was synthesized according to general procedure **D** on a scale of 0.1 mmol. The compound was obtained as a colorless viscous residue in a yield of 34% (13.8 mg with **ITU1**) and 53% (21.3 mg with **ISeU**) after preparative TLC (heptanes/EtOAc, 5/1, as eluent). The determined *e.r.* were >99:1 (**ITU1**) and >99:1 (**ISeU**).

^{1}H-NMR (300 MHz, $CDCl_3$, 298 K) *δ* / ppm = 7.45 (dd, J_1 = 3.6 Hz, J_2 = 1.0 Hz, 1 H, S-CH), 7.30 (dd, J_1 = 5.0 Hz, J_2 = 1.0 Hz, 1 H, S-C_q=CH), 7.03 (dd, J_1 = 5.0 Hz, J_2 = 3.6 Hz, 1 H, S-CH=C**H**), 5.40 (d, *J* = 4.0 Hz, 1 H, =CH), 4.29 (q, *J* = 7.1 Hz, 2 H, CH_2), 4.16 (q, *J* = 7.1 Hz, 2 H, CH_2), 3.45 (d, *J* = 17.1 Hz, 1 H, CH_2-COO), 3.32 (d, *J* = 17.1 Hz, 1 H, CH_2-COO), 3.27 – 3.20 (m, 1 H, CH-CF_3), 2.84 (dd, J_1 = 15.1 Hz, J_2 = 6.1 Hz, 1 H, =C-CH_2), 2.76 (dd, J_1 = 15.1 Hz, J_2 = 7.7 Hz, 1 H, =C-CH_2), 1.33 (t, *J* = 7.1 Hz, 3 H, CH_3), 1.26 (t, *J* = 7.1 Hz, 3 H, CH_3).

^{13}C-NMR (175 MHz, $CDCl_3$, 298 K) *δ* / ppm = 170.9, 166.0, 154.6, 148.8, 136.3, 127.6, 126.5, 126.1 (q, $^1J_{C-F}$ = 279.5 Hz), 125.8, 106.4, 91.1 (q, $^3J_{C-F}$ = 3.1 Hz), 61.3, 61.1, 36.1 (q, $^2J_{C-F}$ = 29.3 Hz), 34.0, 23.2 (q, $^3J_{C-F}$ = 3.1 Hz), 14.5, 14.3.

^{19}F-NMR (282 MHz, $CDCl_3$, 298 K) *δ* / ppm = -72.8.

HRMS (ESI-TOF): *m/z:* $[M+H]^+$ calcd for $C_{18}H_{20}F_3O_5S^+$: 405.0978, found 405.0975.

$[\alpha]_D^{20}$(c = 1, $CHCl_3$, *e.r.* 99:1): +25.3.

HPLC (YMC-SB, *n*-hexane/IPA = 10/1, flow = 1.0 mL min^{-1}, T_{Column} = 20 °C, *l* = 269 nm) t_r: 8.9 min (major), 10.2 min (minor).

Diethyl (*R*,*Z*)-2-(6-(naphthalen-2-yl)-4-(trifluoromethyl)-3,4-dihydro-2*H*-pyran-2-ylidene)succinate (11g)

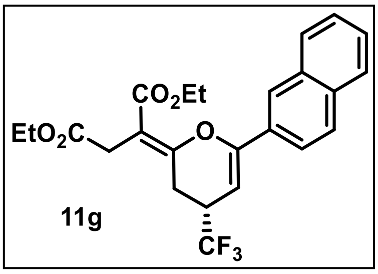

Product **11g** was synthesized according to general procedure **D** on a scale of 0.1 mmol. The compound was obtained as a colorless viscous residue in a yield of 68% (30.4 mg with **ITU1**) and 76% (34.1 mg with **ISeU**) after preparative TLC (heptanes/EtOAc, 5/1, as eluent). The determined *e.r.* were >99:1 (**ITU1**) and >99:1 (**ISeU**).

^{1}H-NMR (300 MHz, $CDCl_3$, 298 K) δ / ppm = 8.33 (broad s, 1 H, Ar-H), 7.91 – 7.76 (m, 4 H, Ar-H), 7.54 – 7.46 (m, 2 H, Ar-H), 5.68 (d, *J* = 3.8 Hz, 1 H, =CH), 4.36 (q, *J* = 7.1 Hz, 2 H, CH_2), 4.19 (q, *J* = 7.1 Hz, 2 H, CH_2), 3.50 (d, *J* = 17.2 Hz, 1 H, CH_2-COO), 3.38 (d, *J* = 17.2 Hz, 1 H, CH_2-COO), 3.37 – 3.27 (m, 1 H, CH-CF_3), 2.92 (dd, J_1 = 15.0 Hz, J_2 = 6.0 Hz, 1 H, =C-CH_2), 2.80 (dd, J_1 = 15.0 Hz, J_2 = 8.3 Hz, 1 H, =C-CH_2), 1.38 (t, *J* = 7.1 Hz, 3 H, CH_3), 1.29 (t, *J* = 7.1 Hz, 3 H, CH_3).

^{13}C-NMR (75 MHz, $CDCl_3$, 298 K) δ / ppm = 171.0, 166.3, 155.2, 152.9, 133.8, 133.2, 129.9, 128.8, 128.2, 127.8, 126.9, 126.6, 126.3 (q, $^1J_{C\text{-}F}$ = 279.4 Hz), 125.1, 122.6, 106.0, 91.7 (q, $^3J_{C\text{-}F}$ = 3.1 Hz), 61.3, 61.1, 36.3 (q, $^2J_{C\text{-}F}$ = 29.3 Hz), 34.1, 23.3 (q, $^3J_{C\text{-}F}$ = 3.1 Hz), 14.5, 14.3.

^{19}F-NMR (282 MHz, $CDCl_3$, 298 K) δ / ppm = -72.7.

HRMS (ESI-TOF): *m/z:* $[M+H]^+$ calcd for $C_{24}H_{24}F_3O_5^+$: 449.1570, found 449.1570.

$[\alpha]_D^{20}$(c = 1, $CHCl_3$, *e.r.* 99:1): +26.9.

HPLC (YMC-SB, *n*-hexane/IPA = 10/1, flow = 1.0 mL min^{-1}, T_{Column} = 20 °C, *l* = 269 nm) t_r: 12.2 min (major), 14.8 min (minor).

4-(*tert*-butyl) 1-ethyl (*R*,*Z*)-2-(6-phenyl-4-(trifluoromethyl)-3,4-dihydro-2*H*-pyran-2-ylidene)succinate (11h)

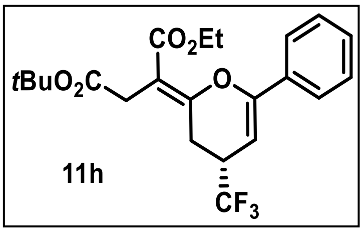

Product **11h** was synthesized according to general procedure **D** on a scale of 0.1 mmol. The compound was obtained as a colorless viscous residue in a yield of 66% (28.0 mg with **ITU1**) and 66% (28.0 mg with **ISeU**) after preparative TLC (heptanes/EtOAc, 5/1, as eluent). The determined *e.r.* were >99:1 (**ITU1**) and >99:1 (**ISeU**).

^{1}H-NMR (300 MHz, $CDCl_3$, 298 K) δ / ppm = 7.79 – 7.75 (m, 2 H, Ar-H), 7.40 – 7.35 (m, 3 H, Ar-H), 5.53 (d, *J* = 3.8 Hz, 1 H, =CH), 4.28 (q, *J* = 7.1 Hz, 2 H, CH_2), 3.38 (d, *J* = 17.2 Hz, 1 H, CH_2-COO), 3.26 (d, *J* = 17.2 Hz, 1 H, CH_2-COO), 3.21 – 3.18 (m, 1 H, CH-CF_3), 2.86 (dd, J_1 = 15.0 Hz, J_2 = 6.0 Hz, 1 H, =C-CH_2), 2.80 (dd, J_1 = 15.0 Hz, J_2= 8.3 Hz, 1 H, =C-CH_2), 1.46 (s, 9 H, $C(CH_3)_3$), 1.33 (t, *J* = 7.1 Hz, 3 H, CH_3).

^{13}C-NMR (75 MHz, $CDCl_3$, 298 K) δ / ppm = 170.1, 166.2, 154.8, 153.0, 132.8, 129.5, 128.5 (2 C), 126.3 (q, $^1J_{C\text{-}F}$ = 279.4 Hz), 125.3 (2 C), 106.4, 91.0 (q, $^3J_{C\text{-}F}$ = 3.1 Hz), 81.4, 60.9, 36.2 (q, $^2J_{C\text{-}F}$ = 29.2 Hz), 35.2, 28.1 (3 C), 23.2 (q, $^3J_{C\text{-}F}$ = 2.9 Hz), 14.4.

^{19}**F-NMR** (282 MHz, $CDCl_3$, 298 K) δ / ppm = -72.8.

HRMS (ESI-TOF): *m/z:* $[M+H]^+$ calcd for $C_{22}H_{26}F_3O_5^+$: 427.1727, found 427.1727.

$[\alpha]_D^{20}$(c = 1, $CHCl_3$, *e.r.* 99:1): +43.7.

HPLC (YMC-SB, *n*-hexane/IPA = 7/3, flow = 1.0 mL min^{-1}, T_{Column} = 20 °C, *l* = 269 nm) t_r: 3.9 min (minor), 4.2 min (major).

Ethyl (*R,Z*)-2-(4-phenyl-6-(trifluoromethyl)-3,4-dihydro-2*H*-pyran-2-ylidene)acetate (12a)

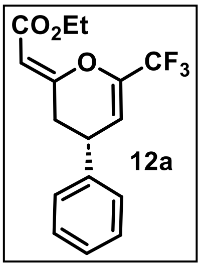

Product **12a** was synthesized according to general procedure **E** on a scale of 0.1 mmol. The compound was obtained as a colorless viscous residue in an isolated yield of 62% (19.3 mg with **ITU1**) and 12% (3.7 mg with **ISeU**) after preparative TLC (heptanes/EtOAc, 2/1, as eluent). The determined *e.r.* were >99:1 (**ITU1**) and >99:1 (**ISeU**).

^{1}**H-NMR** (300 MHz, $CDCl_3$, 298 K) δ / ppm = 7.39-7.26 (m, 3 H, Ar-**H**), 7.18-7.14 (m, 2 H, Ar-**H**), 5.88 (d, *J* = 4.0 Hz, 1 H, -C=C**H**), 5.05 (s, 1 H, -C=C**H**), 4.19 (q, *J* = 7.2 Hz, 2 H, -C**H₂**), 3.74-3.66 (m, 1 H, -C**H**), 2.83 (dd, J_1 = 14.8 Hz, J_2 = 7.7 Hz, 1 H, -C$\mathbf{H_2}$), 2.55 (dd, J_1 = 14.8 Hz, J_2 = 6.2 Hz, 1 H, -C$\mathbf{H_2}$), 1.29 (t, *J* = 7.2 Hz, 3 H, -C$\mathbf{H_3}$).

^{13}**C-NMR** (75 MHz, $CDCl_3$, 298 K) δ / ppm = 164.3, 157.8, 141.2 (q, *J* = 37.4 Hz, 1 C), 140.9, 129.2 (2 C), 127.8, 127.3 (2 C), 119.2 (q, *J* = 271.8 Hz, 1 C), 108.4 (q, *J* = 3.9 Hz, 1 C), 101.1, 60.3, 35.8, 35.1, 14.3.

^{19}**F-NMR** (282 MHz, $CDCl_3$, 298 K) δ / ppm = -72.0 (3 F, -C$\mathbf{F_3}$).

HRMS (ESI-TOF): *m/z:* $[M+H]^+$ calcd for $C_{16}H_{16}F_3O_3^+$: 313.1046, found 313.1065.

$[\alpha]_D^{20}$(c = 1, $CHCl_3$, *e.r.* 99:1): +121.5

HPLC (CHIRALCEL® OD-H, *n*-hexane/IPA = 4/1, flow = 0.5 mL min^{-1}, T_{Column} = 10 °C, *l* = 220 nm) t_r: 12.0 min (major), 14.0 min (minor).

Diethyl (*R,Z*)-2-(4-phenyl-6-(trifluoromethyl)-3,4-dihydro-2*H*-pyran-2-ylidene)succinate (13a)

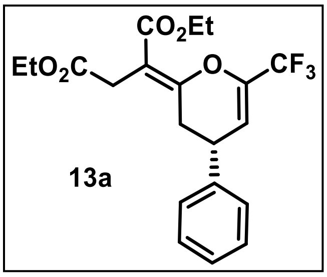

Product **13a** was synthesized according to general procedure **E** on a scale of 0.1 mmol. The compound was obtained as a colorless viscous residue in a yield of 40% (15.9 mg with **ITU1**) after preparative TLC (heptanes/EtOAc, 2/1, as eluent). The determined *e.r.* was >99:1 (**ITU1**).

^{1}**H-NMR** (300 MHz, $CDCl_3$, 298 K) δ / ppm = 7.38 – 7.27 (m, 3 H, Ar-H), 7.20 – 7.16 (m, 2 H, Ar-H), 5.81 (d, *J* = 3.6 Hz, 1 H, =CH), 4.25 (q, *J* = 7.1 Hz, 2 H, CH_2), 4.08 (q, *J* = 7.1 Hz, 2 H, CH_2), 3.77 – 3.67 (m, 1 H, Ar-CH), 3.28 (d, *J* = 17.1 Hz, 1 H,

CH_2-COO), 3.16 (d, J = 17.1 Hz, 1 H, CH_2-COO), 2.90 (dd, J_1 = 14.7 Hz, J_2 = 5.9 Hz, 1 H, =C-CH_2), 2.60 (dd, J_1 = 14.7 Hz, J_2 = 8.3 Hz, 1 H, =C-CH_2), 1.32 (t, J = 7.1 Hz, 3 H, CH_3), 1.22 (t, J = 7.1 Hz, 3 H, CH_3).

^{13}C-NMR (75 MHz, $CDCl_3$, 298 K) δ / ppm = 170.7, 166.6, 154.5, 141.4 (q, $^2J_{C\text{-}F}$ = 37.2 Hz), 141.2, 129.2 (2 C), 127.8, 127.3 (2 C), 119.3 (q, $^1J_{C\text{-}F}$ = 271.8 Hz), 107.8 (q, $^3J_{C\text{-}F}$ = 3.9 Hz), 107.2, 61.2, 61.1, 36.1, 33.8, 32.0, 14.3, 14.1.

^{19}F-NMR (282 MHz, $CDCl_3$, 298 K) δ / ppm = -72.0.

HRMS (ESI-TOF): *m/z:* $[M+H]^+$ calcd for $C_{20}H_{22}F_3O_5^+$: 399.1414, found: 399.1414.

$[\alpha]_D^{20}$(c = 1, $CHCl_3$, *e.r.* 99:1): +115.7

HPLC (YMC-SB, *n*-hexane/IPA = 4/1, flow = 0.5 mL min^{-1}, T_{Column} = 20 °C, l = 269 nm): t_r: 21.1 min (major), 23.2 min (minor).

Ethyl (*S,Z*)-2-(5-(4-methoxyphenyl)-1,3-dimethyl-2,4-dioxo-1,2,3,4,5,6-hexahydro-7*H*-pyrano[2,3-*d*]pyrimidin-7-ylidene)acetate (14a)

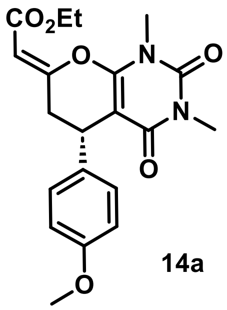

Product **14a** was synthesized according to general procedure **C** on a scale of 0.1 mmol. The compound was obtained as a slightly yellow viscous residue in an isolated yield of 94% (36.3 mg with **ITU1**) and 96% (37.1 mg with **ISeU**) after preparative TLC (heptanes/EtOAc, 1/1, as eluent). The determined *e.r.* were 80:20 (**ITU1**) and 86:14 (**ISeU**).

^{1}H-NMR (300 MHz, $CDCl_3$, 298 K) δ / ppm = 7.08-7.05 (m, 2 H, Ar-**H**), 6.82-6.79 (m, 2 H, Ar-**H**), 5.16 (d, J = 1.9 Hz, 1 H, -C**H**), 4.21-4.19 (m, 1 H, -C**H**), 4.18 (q, J = 7.1 Hz, 2 H, -C**H₂**), 3.74 (s, 3 H, -OC**H₃**), 3.61 (s, 3 H, -NC**H₃**), 3.30 (s, 3 H, -NC**H₃**), 2.92 (ddd, J_1 = 14.9 Hz, J_2 = 6.9 Hz, J_3 = 1.9 Hz, 1 H, -C**H₂**), 2.60 (dd, J_1 = 14.9 Hz, J_2 = 1.7 Hz, 1 H, -C**H₂**), 1.28 (t, J = 7.1 Hz, 3 H, -C**H₃**).

^{13}C-NMR (75 MHz, $CDCl_3$, 298 K) δ / ppm = 163.4 (1 C, -**C**OOEt), 161.8 (1 C, -N-**C**=O), 158.8 (1 C, -O-**C**=CH), 157.1 (1 C, -O-**C**-N), 153.0 (1 C, -N-**C**=O), 150.9 (1 C, $\mathbf{C_{Ar}}$), 133.8 (1 C, $\mathbf{C_{Ar}}$), 128.2 (2 C, $\mathbf{C_{Ar}}$), 114.2 (2 C, $\mathbf{C_{Ar}}$), 103.2 (1 C, -C=**C**H), 90.5 (1 C, -C-**C**=C-O), 60.3 (1 C, -O-**C**H_2-CH_3), 55.3 (1 C, -O-**C**H_3), 34.7 (1 C, -**C**H_2), 32.6 (1 C, -**C**H), 29.6 (1 C, -N**C**H_3), 28.2 (1 C, -N-**C**H_3), 14.3 (1 C, -O-CH_2-**C**H_3).

HRMS (ESI-TOF): *m/z:* $[M+H]^+$ calcd for $C_{20}H_{23}N_2O_6^+$: 387.1551, found 387.1553.

$[\alpha]_D^{20}$(c = 1, $CHCl_3$, *e.r.* 86:14): -35.1

HPLC (YMC-SB, *n*-hexane/IPA = 1/1, flow = 1.0 mL min^{-1}, T_{Column} = 10 °C, l = 210 nm) t_r: 18.9 min (major), 36.5 min (minor).

Ethyl (*S,Z*)-2-(1,3-dimethyl-2,4-dioxo-5-phenyl-1,2,3,4,5,6-hexahydro-7*H*-pyrano[2,3-*d*]pyrimidin-7-ylidene)acetate (14b)

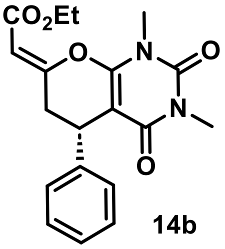

14b

Product **14b** was synthesized according to general procedure **C** on a scale of 0.1 mmol. The compound was obtained as a slightly yellowish viscous residue in an isolated yield of 92% (32.8 mg with **ITU1**) and 92% (32.8 mg with **ISeU**) after preparative TLC (heptanes/EtOAc, 1/1, as eluent). The determined *e.r.* were 72:28 (**ITU1**) and 78:22 (**ISeU**).

^{1}H-NMR (300 MHz, $CDCl_3$, 298 K) δ / ppm = 7.33-7.23 (m, 3 H, Ar-**H**), 7.18-7.15 (m, 2 H, Ar-**H**), 5.16 (d, J = 1.9 Hz, 1 H, -C=C**H**), 4.26 (dd, J_1 = 6.9 Hz, J_2 = 1.7 Hz, 1 H, -C**H**), 4.19 (q, J = 7.1 Hz, 2 H, -C**H₂**), 3.63 (s, 3 H, -NC**H₃**), 3.32 (s, 3 H, -NC**H₃**), 2.96 (ddd, J_1 = 15.0 Hz, J_2 = 6.9 Hz, J_3 = 1.9 Hz, 1 H, -C**H₂**), 2.61 (dd, J_1 = 15.0 Hz, J_2 = 1.7 Hz, 1 H, -C**H₂**), 1.27 (t, J = 7.1 Hz, 3 H, -C**H₃**).

^{13}C-NMR (75 MHz, $CDCl_3$, 298 K) δ / ppm = 163.4 (1 C, -**C**OOEt), 161.8 (1 C, -N-**C**=O), 157.0 (1 C, -O-**C**=CH), 153.2 (1 C, -**C**=C), 150.9 (1 C, -N-**C**=O), 141.7 (1 C, $\mathbf{C_{Ar}}$), 128.9 (2 C, $\mathbf{C_{Ar}}$), 127.4 (1 C, $\mathbf{C_{Ar}}$), 127.2 (2 C, $\mathbf{C_{Ar}}$), 103.3 (1 C, -C=**C**H), 90.2 (1 C, -**C**=CH), 60.4 (1 C, -O-**C**H_2-CH_3), 34.6 (1 C, -**C**H_2), 33.5 (1 C, -**C**H), 29.6 (1 C, -N-**C**H_3), 28.3 (1 C, -N-**C**H_3), 14.4 (1 C, -O-CH_2-**C**H_3).

HRMS (ESI-TOF): *m/z:* $[M+H]^+$ calcd for $C_{19}H_{21}N_2O_5^+$: 357.1445, found 357.1448.

$[\alpha]_D^{20}$(c = 1, $CHCl_3$, *e.r.* 78:22): -26.7

HPLC (YMC-SB, *n*-hexane/IPA = 1/1, flow = 1.0 mL min^{-1}, T_{Column} = 10 °C, l = 210 nm) t_r: 12.5 min (major), 24.5 min (minor).

Ethyl (*S,Z*)-2-(5-(4-(dimethylamino)phenyl)-1,3-dimethyl-2,4-dioxo-1,2,3,4,5,6-hexahydro-7*H*-pyrano[2,3-*d*]pyrimidin-7-ylidene)acetate (14c)

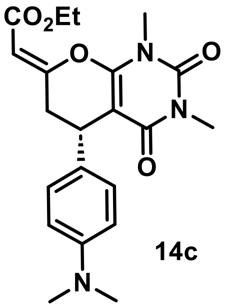

14c

Product **14c** was synthesized according to general procedure **C** on a scale of 0.1 mmol. The compound was obtained as a reddish viscous residue in an isolated yield of 92% (36.7 mg with **ITU1**) and 95% (37.9 mg with **ISeU**) after preparative TLC (heptanes/EtOAc, 1/1, as eluent). The determined *e.r.* were 80:20 (**ITU1**) and 86:14 (**ISeU**).

^{1}H-NMR (300 MHz, $CDCl_3$, 298 K) δ / ppm = 7.04-7.01 (m, 2 H, Ar-**H**), 6.65-6.62 (m, 2 H, Ar-**H**), 5.17 (d, J = 1.9 Hz, 1 H, -C=C**H**), 4.18 (q, J = 7.1 Hz, 2 H, -C**H₂**), 4.16-4.14 (m, 1 H, -C**H**), 3.61 (s, 3 H, -NC**H₃**), 3.30 (s, 3 H, -NC**H₃**), 2.94-2.86 (m, 1 H, -C**H₂**), 2.89 (s, 6 H, -N(C**H₃**)$_2$), 2.61 (dd, J_1 = 14.9 Hz, J_2 = 1.7 Hz, 1 H, -C**H₂**), 1.28 (t, J = 7.1 Hz, 3 H, -C**H₃**).

^{13}C-NMR (75 MHz, $CDCl_3$, 298 K) δ / ppm = 163.5, 161.8, 157.6, 152.8, 151.0, 149.9, 129.5, 127.8 (2 C), 112.8 (2 C), 103.0, 91.0, 60.3, 40.7 (2 C), 34.7, 32.5, 29.6, 28.2, 14.4.

HRMS (ESI-TOF): *m/z:* $[M+H]^+$ calcd for $C_{21}H_{26}N_3O_5^+$: 400.1867, found 400.1866.

$[\alpha]_D^{20}$(c = 1, $CHCl_3$, *e.r.* 86:14): -47.5

HPLC (YMC-SB, *n*-hexane/IPA = 1/1, flow = 1.0 mL min^{-1}, T_{Column} = 10 °C, *l* = 220 nm) t_r: 20.6 min (major), 33.6 min (minor).

Ethyl (*S,Z*)-2-(5-(4-fluorophenyl)-1,3-dimethyl-2,4-dioxo-1,2,3,4,5,6-hexahydro-7*H*-pyrano[2,3-*d*]pyrimidin-7-ylidene)acetate (14d)

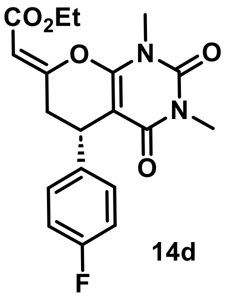

Product **14d** was synthesized according to general procedure **C** on a scale of 0.1 mmol. The compound was obtained as a colorless viscous residue in an isolated yield of 98% (36.7 mg with **ITU1**) and 97% (36.3 mg with **ISeU**) after preparative TLC (heptanes/EtOAc, 1/1, as eluent). The determined *e.r.* were 70:30 (**ITU1**) and 78:22 (**ISeU**).

^{1}H-NMR (300 MHz, $CDCl_3$, 298 K) δ / ppm = 7.15-7.10 (m, 2 H, Ar-**H**), 7.01-6.94 (m, 2 H, Ar-**H**), 5.16 (d, *J* = 1.9 Hz, 1 H, -C=C**H**), 4.25-4.22 (m, 1 H, -C**H**), 4.19 (q, *J* = 7.1 Hz, 2 H, -C**H$_2$**), 3.62 (s, 3 H, -NC**H$_3$**), 3.31 (s, 3 H, -NC**H$_3$**), 2.94 (ddd, J_1 = 14.9 Hz, J_2 = 6.9 Hz, J_3 = 1.9 Hz, 1 H, -C**H$_2$**), 2.58 (dd, J_1 = 14.9 Hz, J_2 = 1.7 Hz, 1 H, -C**H$_2$**), 1.29 (t, *J* = 7.1 Hz, 3 H, -C**H$_3$**).

^{13}C-NMR (75 MHz, $CDCl_3$, 298 K) δ / ppm = 163.3, 162.1 (d, *J* = 245.7 Hz, 1 C), 161.8, 156.6, 153.3, 150.9, 137.5 (d, *J* = 3.1 Hz, 1 C), 128.8 (d, *J* = 8.0 Hz, 2 C), 115.7 (d, *J* = 21.5 Hz, 2 C), 103.5, 90.1, 60.5, 34.7, 32.9, 29.7, 28.3, 14.4.

^{19}F-NMR (282 MHz, $CDCl_3$, 298 K) δ / ppm = -115.4 (1 F, Ar-**F**).

HRMS (ESI-TOF): *m/z:* $[M+H]^+$ calcd for $C_{19}H_{20}FN_2O_5^+$: 375.1351, found 375.1354.

$[\alpha]_D^{20}$(c = 1, $CHCl_3$, *e.r.* 78:22): -22.7

HPLC (YMC-SB, *n*-hexane/IPA = 1/1, flow = 1.0 mL min^{-1}, T_{Column} = 10 °C, *l* = 220 nm) t_r: 12.4 min (major), 29.6 min (minor).

Ethyl (*R,Z*)-2-(5-(2-fluorophenyl)-1,3-dimethyl-2,4-dioxo-1,2,3,4,5,6-hexahydro-7*H*-pyrano[2,3-*d*] pyrimidin-7-ylidene)acetate (14e)

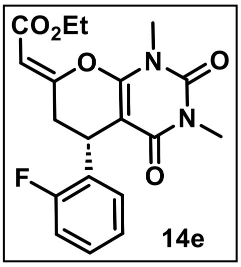

Product **14e** was synthesized according to general procedure **C** on a scale of 0.1 mmol. The compound was obtained as a colorless viscous residue in an isolated yield of 67% (25.1 mg with **ITU1**) and 88% (32.9 mg with **ISeU**) after preparative TLC (heptanes/EtOAc, 1/1, as eluent). The determined *e.r.* were 76:24 (**ITU1**) and 76:24 (**ISeU**).

^{1}H-NMR (300 MHz, $CDCl_3$, 298 K) δ / ppm = 7.24-7.17 (m, 1 H, Ar-**H**), 7.07-6.99 (m, 2 H, Ar-**H**), 6.91-6.86 (m, 1 H, Ar-**H**), 5.10 (d, J = 1.9 Hz, 1 H, -C=C**H**), 4.57 (dd, J_1 = 6.9 Hz, J_2 = 1.7 Hz, 1 H, -C**H**), 4.15 (q, J = 7.1 Hz, 2 H, -C**H$_2$**), 3.62 (s, 3 H, -NC**H$_3$**), 3.31 (s, 3 H, -NC**H$_3$**), 2.93 (ddd, J_1 = 14.9 Hz, J_2 = 6.9 Hz, J_3 = 1.9 Hz, 1 H, -C**H$_2$**), 2.65 (dd, J_1 = 14.9 Hz, J_2 = 1.7 Hz, 1 H, -C**H$_2$**), 1.26 (t, J = 7.1 Hz, 3 H, -C**H$_3$**).

^{13}C-NMR (75 MHz, $CDCl_3$, 298 K) δ / ppm = 163.3, 161.5, 160.5 (d, J = 246.2 Hz, 1 C), 156.6, 154.0, 150.9, 129.1 (d, J = 8.3 Hz, 1 C), 128.5 (d, J = 3.9 Hz, 1 C), 128.1 (d, J = 13.8 Hz, 1 C), 124.3 (d, J = 3.9 Hz, 1 C), 115.9 (d, J = 22.0 Hz, 1 C), 103.3, 88.5, 60.4, 33.6, 29.6, 28.3, 27.6, 27.5, 14.3.

^{19}F-NMR (282 MHz, $CDCl_3$, 298 K) δ / ppm = -118.4 (1 F, Ar-**F**).

HRMS (ESI-TOF): *m/z:* $[M+H]^+$ calcd for $C_{20}H_{23}N_2O_5^+$: 375.1351, found 375.1351.

$[\alpha]_D^{20}$(c = 1, $CHCl_3$, *e.r.* 76:24): -35.2

HPLC (YMC-SB, *n*-hexane/IPA = 1/1, flow = 1.0 mL min^{-1}, T_{Column} = 10 °C, *l* = 210 nm) t_r: 13.1 min (major), 17.9 min (minor).

Ethyl (*S,Z*)-2-(5-(4-bromophenyl)-1,3-dimethyl-2,4-dioxo-1,2,3,4,5,6-hexahydro-7*H*-pyrano[2,3-*d*]pyrimidin-7-ylidene)acetate (14f)

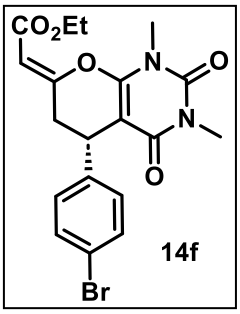

Product **14f** was synthesized according to general procedure **C** on a scale of 0.1 mmol. The compound was obtained as a slightly beige viscous residue in an isolated yield of 64% (27.8 mg with **ITU1**) and 82% (35.7 mg with **ISeU**) after preparative TLC (heptanes/EtOAc, 1/1, as eluent). The determined *e.r.* were 70:30 (**ITU1**) and 69:31 (**ISeU**).

^{1}H-NMR (300 MHz, $CDCl_3$, 298 K) δ / ppm = 7.41-7.38 (m, 2 H, Ar-**H**), 7.04-7.01 (m, 2 H, Ar-**H**), 5.15 (d, J = 1.9 Hz, 1 H, -C=C**H**), 4.21-4.18 (m, 1 H, -C**H**), 4.18 (q, J = 7.1 Hz, 2 H, -C**H$_2$**), 3.61 (s, 3 H, -NC**H$_3$**), 3.30 (s, 3 H, -NC**H$_3$**), 2.94 (ddd, J_1 = 14.9 Hz, J_2 = 6.9 Hz, J_3 = 1.9 Hz, 1 H, -C**H$_2$**), 2.57 (dd, J_1 = 14.9 Hz, J_2 = 1.8 Hz, 1 H, -C**H$_2$**), 1.28 (t, J = 7.1 Hz, 3 H, -C**H$_3$**).

^{13}C-NMR (75 MHz, $CDCl_3$, 298 K) δ / ppm = 163.2, 161.7, 156.4, 153.4, 150.8, 140.8, 131.9 (2 C), 128.9 (2 C), 121.3, 103.6, 89.7, 60.5, 34.4, 33.1, 29.6, 28.3, 14.3.

HRMS (ESI-TOF): *m/z:* $[M+H]^+$ calcd for $C_{19}H_{20}BrN_2O_5^+$: 437.0530 (^{81}Br isotope), found 437.0530 (^{81}Br isotope).

$[\alpha]_D^{20}$(c = 1, $CHCl_3$, *e.r.* 70:30): -17.5

HPLC (YMC-SB, *n*-hexane/IPA = 1/1, flow = 1.0 mL min^{-1}, T_{Column} = 10 °C, *l* = 220 nm) t_r: 13.8 min (major), 32.8 min (minor).

Ethyl (*R,Z*)-2-(1,3-dimethyl-2,4-dioxo-5-(perfluorophenyl)-1,2,3,4,5,6-hexahydro-7*H*-pyrano[2,3-*d*] pyrimidin-7-ylidene)acetate (14g)

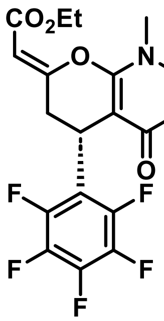

14g

Product **14g** was synthesized according to general procedure **C** on a scale of 0.1 mmol with slight adaptation. The reaction temperature was decreased to 80 °C whereas the reaction time was increased to 24 h. The compound was obtained as a colorless oily residue in an isolated yield of 14% (6.2 mg with **ITU1**) and 36% (16.1 mg with **ISeU**) after preparative TLC (heptanes/EtOAc, 1/1, as eluent). The determined *e.r.* were 72:28 (**ITU1**) and 73:27 (**ISeU**).

^{1}H-NMR (300 MHz, $CDCl_3$, 298 K) δ / ppm = 5.27 (d, *J* = 1.7 Hz, 1 H, -C=C**H**), 4.59 (dd, J_1 = 7.5 Hz, J_2 = 2.6 Hz, 1 H, -C**H**), 4.21 (q, *J* = 7.1 Hz, 2 H, -C**H₂**), 3.61 (s, 3 H, -NC**H₃**), 3.29 (s, 3 H, -NC**H₃**), 3.02 (ddd, J_1 = 15.4 Hz, J_2 = 7.5 Hz, J_3 = 1.7 Hz, 1 H, -C**H₂**), 2.65 (dd, J_1 = 15.4 Hz, J_2 = 2.6 Hz, 1 H, -C**H₂**), 1.30 (t, *J* = 7.1 Hz, 3 H, -C**H₃**).

^{13}C-NMR (75 MHz, $CDCl_3$, 298 K) δ / ppm = 163.1, 161.6, 155.5, 154.1, 150.7, 103.2, 86.7, 60.6, 32.4, 29.7, 28.2, 24.3, 14.4.

^{19}F-NMR (282 MHz, $CDCl_3$, 298 K) δ / ppm = -142.6 (2 F, Ar-**F**), -155.0 (1 F, Ar-**F**), -161.4 (2 F, Ar-**F**).

HRMS (ESI-TOF): *m/z:* $[M+H]^+$ calcd for $C_{19}H_{16}F_5N_2O_5^+$: 447.0974, found 447.0973.

$[\alpha]_D^{20}$(c = 1, $CHCl_3$, *e.r.* 73:28): +63.9

HPLC (YMC-SB, *n*-hexane/IPA = 1/1, flow = 1.0 mL min^{-1}, T_{Column} = 10 °C, *l* = 210 nm) t_r: 10.9 min (major), 17.2 min (minor).

Ethyl (*S,Z*)-2-(1,3-dimethyl-2,4-dioxo-5-(*p*-tolyl)-1,2,3,4,5,6-hexahydro-7*H*-pyrano[2,3-*d*]pyrimidin -7-ylidene)acetate (14h)

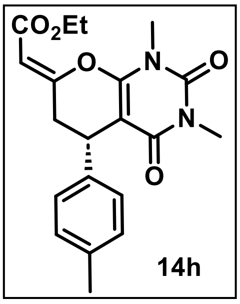

14h

Product **14h** was synthesized according to general procedure **C** on a scale of 0.1 mmol. The compound was obtained as a colorless viscous residue in an isolated yield of 84% (31.1 mg with **ITU1**) and 85% (31.5 mg with **ISeU**) after preparative TLC (heptanes/EtOAc, 1/1, as eluent). The determined *e.r.* were 76:24 (**ITU1**) and 81:19 (**ISeU**).

^{1}H-NMR (300 MHz, $CDCl_3$, 298 K) δ / ppm = 7.10-7.02 (m, 4 H, Ar-**H**), 5.15 (d, J = 1.9 Hz, 1 H, -C=C**H**), 4.21-4.19 (m, 1 H, -C**H**), 4.18 (q, J = 7.1 Hz, 2 H, -C**H$_2$**), 3.62 (s, 3 H, -NC**H$_3$**), 3.30 (s, 3 H, -NC**H$_3$**), 2.93 (ddd, J_1 = 14.9 Hz, J_2 = 6.9 Hz, J_3 = 1.9 Hz, 1 H, -C**H$_2$**), 2.60 (dd, J_1 = 14.9 Hz, J_2 = 1.7 Hz, 1 H, -C**H$_2$**), 2.28 (s, 3 H, -C**H$_3$**), 1.28 (t, J = 7.1 Hz, 3 H, -C**H$_3$**).

^{13}C-NMR (75 MHz, $CDCl_3$, 298 K) δ / ppm = 163.4, 161.8, 157.1, 153.1, 150.9, 138.8, 137.0, 129.5 (2 C), 127.0 (2 C), 103.2, 90.4, 60.3, 34.6, 33.1, 29.6, 28.2, 21.1, 14.3.

HRMS (ESI-TOF): *m/z:* $[M+H]^+$ calcd for $C_{20}H_{23}N_2O_5^+$: 371.1601, found 371.1603.

$[\alpha]_D^{20}$(c = 1, $CHCl_3$, *e.r.* 81:29): -29.4

HPLC (YMC-SB, *n*-hexane/IPA = 1/1, flow = 1.0 mL min^{-1}, T_{Column} = 10 °C, *l* = 210 nm) t_r: 12.5 min (major), 21.8 min (minor).

Ethyl (*S,Z*)-2-(1,3-dimethyl-2,4-dioxo-5-(*o*-tolyl)-1,2,3,4,5,6-hexahydro-7*H*-pyrano[2,3-*d*]pyrimidin-7-ylidene)acetate (14i)

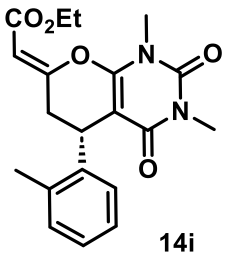

14i

Product **14i** was synthesized according to general procedure **C** on a scale of 0.1 mmol. The compound was obtained as a colorless viscous residue in an isolated yield of 96% (35.4 mg with **ITU1**) and 96% (35.5 mg with **ISeU**) after preparative TLC (heptanes/EtOAc, 1/1, as eluent). The determined *e.r.* were 72:28 (**ITU1**) and 75:25 (**ISeU**).

^{1}H-NMR (300 MHz, $CDCl_3$, 298 K) δ / ppm = 7.18-7.03 (m, 3 H, Ar-**H**), 6.78-6.74 (m, 1 H, Ar-**H**), 5.07 (d, J = 1.9 Hz, 1 H, -C=C**H**), 4.47 (dd, J_1 = 7.0 Hz, J_2 = 1.7 Hz 1 H, -C**H**), 4.17 (q, J = 7.1 Hz, 2 H, -C**H$_2$**), 3.64 (s, 3 H, -NC**H$_3$**), 3.29 (s, 3 H, -NC**H$_3$**), 2.92 (ddd, J_1 = 14.7 Hz, J_2 = 7.0 Hz, J_3 = 1.9 Hz, 1 H, -C**H$_2$**), 2.50 (s, 3 H, -C**H$_3$**), 2.45 (dd, J_1 = 14.7 Hz, J_2 = 1.7 Hz, 1 H, -C**H$_2$**), 1.27 (t, J = 7.1 Hz, 3 H, -C**H$_3$**).

^{13}C-NMR (75 MHz, $CDCl_3$, 298 K) δ / ppm = 163.4, 161.7, 156.7, 153.6, 150.9, 139.5, 135.2, 131.0, 127.3, 126.3, 126.2, 103.5, 90.4, 60.3, 33.6, 29.6, 29.6, 28.2, 19.6, 14.3.

HRMS (ESI-TOF): *m/z:* $[M+H]^+$ calcd for $C_{20}H_{23}N_2O_5^+$: 371.1601, found 371.1601.

$[\alpha]_D^{20}$(c = 1, $CHCl_3$, *e.r.* 75:25): -29.4

HPLC (YMC-SA, *n*-hexane/IPA = 4/1, flow = 0.5 mL min^{-1}, T_{Column} = 10 °C, *l* = 220 nm) t_r: 13.9 min (major), 15.5 min (minor).

Ethyl (*S,Z*)-2-(5-(4-(*tert*-butyl)phenyl)-1,3-dimethyl-2,4-dioxo-1,2,3,4,5,6-hexahydro-7*H*-pyrano [2,3-*d*]pyrimidin-7-ylidene)acetate (14j)

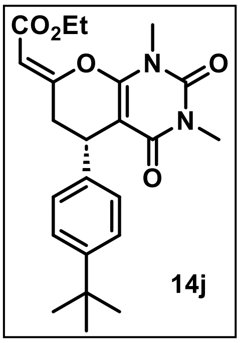

Product **14j** was synthesized according to general procedure **C** on a scale of 0.1 mmol. The compound was obtained as a beige viscous residue in an isolated yield of 90% (37.1 mg with **ITU1**) and 92% (37.9 mg with **ISeU**) after preparative TLC (heptanes/EtOAc, 1/1, as eluent). The determined *e.r.* were 75:25 (**ITU1**) and 81:19 (**ISeU**).

^{1}H-NMR (300 MHz, $CDCl_3$, 298 K) δ / ppm = 7.30-7.26 (m, 2 H, Ar-**H**), 7.10-7.07 (m, 2 H, Ar-**H**), 5.18 (d, J = 1.9 Hz, 1 H, -C=C**H**), 4.23-4.16 (m, 3 H, -C**H**, C**H$_2$**), 3.61 (s, 3 H, -NC**H$_3$**), 3.31 (s, 3 H, -NC**H$_3$**), 2.93 (ddd, J_1 = 15.0 Hz, J_2 = 6.9 Hz, J_3 = 1.9 Hz, 1 H, -C**H$_2$**), 2.65 (dd, J_1 = 15.0 Hz, J_2 = 1.7 Hz, 1 H, -C**H$_2$**), 1.29 (t, J = 7.1 Hz, 3 H, -C**H$_3$**), 1.27 (s, 9 H, -C(C**H**$_3$)$_3$).

^{13}C-NMR (75 MHz, $CDCl_3$, 298 K) δ / ppm = 163.5, 161.9, 157.3, 153.0, 150.9, 150.0, 138.7, 126.8 (2 C), 125.8 (2 C), 103.1, 90.6, 60.4, 34.5, 34.3, 32.8, 31.4 (3 C), 29.6, 28.3, 14.4

HRMS (ESI-TOF): *m/z:* $[M+H]^+$ calcd for $C_{23}H_{29}N_2O_5^+$: 413.2071, found 413.2070.

$[\alpha]_D^{20}$(c = 1, $CHCl_3$, *e.r.* 81:19): -28.4

HPLC (YMC-SB, *n*-hexane/IPA = 1/1, flow = 1.0 mL min^{-1}, T_{Column} = 10 °C, l = 220 nm) t_r: 11.4 min (major), 24.7 min (minor)

Ethyl (*R,Z*)-2-(1,3-dimethyl-2,4-dioxo-5-(thiophen-2-yl)-1,2,3,4,5,6-hexahydro-7*H*-pyrano[2,3-*d*] pyrimidin-7-ylidene)acetate (14k)

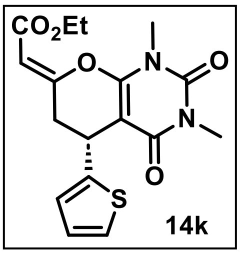

Product **14k** was synthesized according to general procedure **C** on a scale of 0.1 mmol. The compound was obtained as a beige viscous residue in an isolated yield of 91% (32.9 mg with **ITU1**) and 86% (31.1 mg with **ISeU**) after preparative TLC (heptanes/EtOAc, 1/1, as eluent). The determined *e.r.* were 75:25 (**ITU1**) and 83:17 (**ISeU**).

^{1}H-NMR (300 MHz, $CDCl_3$, 298 K) δ / ppm = 7.13 (dd, J_1 = 5.0 Hz, J_2 = 1.3 Hz, 1 H, Ar-**H**), 6.90-6.85 (m, 2 H, Ar-**H**), 5.29 (d, J = 1.9 Hz, -C=C**H**), 4.55-4.52 (m, 1 H, -C**H**), 4.18 (q, J = 7.1 Hz, 2 H, -C**H$_2$**), 3.58 (s, 3 H, -NC**H$_3$**), 3.33 (s, 3 H, -NC**H$_3$**), 2.93 (ddd, J_1 = 15.0 Hz, J_2 = 6.4 Hz, J_3 = 1.9 Hz, 1 H, -C**H$_2$**), 2.78 (dd, J_1 = 15.0 Hz, J_2 = 1.9 Hz, 1 H, -C**H$_2$**), 1.28 (t, J = 7.1 Hz, 3 H, -C**H$_3$**).

^{13}C-NMR (75 MHz, $CDCl_3$, 298 K) δ / ppm = 163.3, 161.6, 156.6, 152.9, 150.8, 145.0, 127.0, 124.7, 124.4, 103.9, 90.8, 60.4, 34.6, 29.6, 28.7, 28.3, 14.3.

HRMS (ESI-TOF): *m/z:* $[M+H]^+$ calcd for $C_{17}H_{19}N_2O_5S^+$: 363.1009, found 363.1010.

$[\alpha]_D^{20}$(c = 1, $CHCl_3$, *e.r.* 83:17): -68.9

HPLC (YMC-SB, *n*-hexane/IPA = 1/1, flow = 1.0 mL min^{-1}, T_{Column} = 10 °C, *l* = 220 nm) t_r: 19.4 min (major), 34.3 min (minor).

Ethyl (*S,Z*)-2-(1,3-dimethyl-5-(naphthalen-2-yl)-2,4-dioxo-1,2,3,4,5,6-hexahydro-7*H*-pyrano[2,3-*d*] pyrimidin-7-ylidene)acetate (14l)

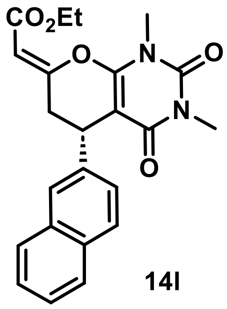

14l

Product **14l** was synthesized according to general procedure **C** on a scale of 0.1 mmol. The compound was obtained as a beige viscous residue in an isolated yield of 78% (31.7 mg with **ITU1**) and 80% (32.5 mg with **ISeU**) after preparative TLC (heptanes/EtOAc, 1/1, as eluent). The determined *e.r.* were 66:34 (**ITU1**) and 72:28 (**ISeU**).

^{1}H-NMR (300 MHz, $CDCl_3$, 298 K) δ / ppm = 7.80-7.76 (m, 3 H, Ar-**H**), 7.56-7.55 (m, 1 H, Ar-**H**), 7.47-7.40 (m, 2 H, Ar-**H**), 7.30 (dd, J_1 = 8.5 Hz, J_2 = 1.8 Hz, 1 H, Ar-**H**), 5.13 (d, *J* = 1.8 Hz, 1 H, -C=C**H**), 4.42-4.40 (m, 1 H, -C**H**), 4.17 (q, *J* = 7.1 Hz, 2 H, -C$\mathbf{H_2}$), 3.67 (s, 3 H, -NC$\mathbf{H_3}$), 3.32 (s, 3 H, -NC$\mathbf{H_3}$), 3.01 (ddd, J_1 = 14.9 Hz, J_2 = 6.9 Hz, J_3 = 1.8 Hz, 1 H, -C$\mathbf{H_2}$), 2.70 (dd, J_1 = 14.9 Hz, J_2 = 1.6 Hz, 1 H, -C$\mathbf{H_2}$), 1.26 (t, *J* = 7.1 Hz, 3 H, -C$\mathbf{H_3}$).

^{13}C-NMR (75 MHz, $CDCl_3$, 298 K) δ / ppm = 163.3, 161.8, 156.9, 153.4, 150.9, 139.1, 133.4, 132.7, 128.7, 128.0, 127.7, 126.3, 125.9, 125.7, 125.4, 103.3, 90.0, 60.3, 34.6, 33.6, 29.7, 28.3, 14.3.

HRMS (ESI-TOF): *m/z:* $[M+H]^+$ calcd for $C_{23}H_{23}N_2O_5^+$: 407.1601, found 407.1603.

$[\alpha]_D^{20}$(c = 1, $CHCl_3$, *e.r.* 72:28): -2.7

HPLC (YMC-SB, *n*-hexane/IPA = 1/1, flow = 1.0 mL min^{-1}, T_{Column} = 10 °C, *l* = 220 nm) t_r: 16.0 min (major), 31.8 min (minor).

***tert*-Butyl (*S,Z*)-2-(5-(4-methoxyphenyl)-1,3-dimethyl-2,4-dioxo-1,2,3,4,5,6-hexahydro-7*H*-pyrano [2,3-*d*]pyrimidin-7-ylidene)acetate (14m)**

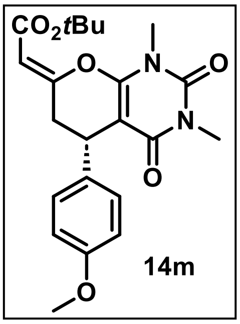

14m

Product **14m** was synthesized according to general procedure **C** on a scale of 0.1 mmol. The compound was obtained as a colorless viscous residue in an isolated yield of 81% (33.5 mg with **ITU1**) and 91% (37.7 mg with **ISeU**) after preparative TLC (heptanes/EtOAc, 1/1, as eluent). The determined *e.r.* were 78:22 (**ITU1**) and 86:14 (**ISeU**).

^{1}H-NMR (300 MHz, $CDCl_3$, 298 K) δ / ppm = 7.10-7.07 (m, 2 H, Ar-**H**), 6.83-6.80 (m, 2 H, Ar-**H**), 5.08 (d, *J* = 1.8 Hz, 1 H, -C=C**H**), 4.18 (dd, J_1 = 6.8 Hz, J_2 = 1.7 Hz, 1 H, -C**H**), 3.75 (s, 3 H, -OC$\mathbf{H_3}$), 3.61 (s, 3 H, -NC$\mathbf{H_3}$), 3.30 (s, 3 H, -NC$\mathbf{H_3}$), 2.89 (ddd, J_1 = 14.8 Hz, J_2 = 6.8 Hz, J_3 = 1.8 Hz, 1 H, -C$\mathbf{H_2}$), 2.54 (dd, J_1 = 14.8 Hz, J_2 = 1.7 Hz, 1 H, -C$\mathbf{H_2}$), 1.27 (s, 9 H, -C(C$\mathbf{H}_3$)$_3$).

^{13}C-NMR (75 MHz, $CDCl_3$, 298 K) δ / ppm = 162.6, 161.8, 158.8, 155.9, 153.2, 151.0, 134.0, 128.3 (2 C), 114.2 (2 C), 104.9, 90.5, 80.8, 55.3, 34.7, 32.8, 29.7, 28.3 (3 C), 28.2.

HRMS (ESI-TOF): *m/z:* $[M+H]^+$ calcd for $C_{22}H_{27}N_2O_6^+$: 415.1864, found 415.1867.

$[\alpha]_D^{20}$(c = 1, $CHCl_3$, *e.r.* 78:22): -20.0

HPLC (YMC-SB, *n*-hexane/IPA = 1/1, flow = 1.0 mL min^{-1}, T_{Column} = 10 °C, *l* = 210 nm) t_r: 13.9 min (major), 29.5 min (minor).

***tert*-Butyl (*S,Z*)-2-(5-(4-(*tert*-butyl)phenyl)-1,3-dimethyl-2,4-dioxo-1,2,3,4,5,6-hexahydro-7*H*-pyrano[2,3-*d*]pyrimidin-7-ylidene)acetate (14n)**

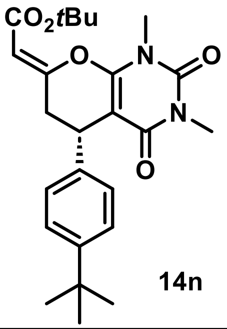

14n

Product **14n** was synthesized according to general procedure **C** on a scale of 0.1 mmol. The compound was obtained as a colorless viscous residue in an isolated yield of 76% (33.4 mg with **ITU1**) and 94% (41.4 mg with **ISeU**) after preparative TLC (heptanes/EtOAc, 1/1, as eluent). The determined *e.r.* were 75:25 (**ITU1**) and 80:20 (**ISeU**).

^{1}H-NMR (300 MHz, $CDCl_3$, 298 K) δ / ppm = 7.30-7.27 (m, 2 H, Ar-**H**), 7.11-7.09 (m, 2 H, Ar-**H**), 5.11 (d, *J* = 1.9 Hz, 1 H, -C**H**), 4.20-4.18 (m, 1 H, -C**H**), 3.61 (s, 3 H, -NC**H$_3$**), 3.30 (s, 3 H, -NC**H$_3$**), 2.90 (ddd, J_1 = 15.0 Hz, J_2 = 6.9 Hz, J_3 = 1.9 Hz, 1 H, -C**H$_2$**), 2.60 (dd, J_1 = 15.0 Hz, J_2 = 1.7 Hz, 1 H, -C**H$_2$**), 1.49 (s, 9 H, -C(C**H**$_3$)$_3$), 1.27 (s, 9 H, -C(C**H**$_3$)$_3$).

^{13}C-NMR (75 MHz, $CDCl_3$, 298 K) δ / ppm = 162.7, 161.9, 156.0, 153.1, 151.0, 150.0, 138.8, 126.9 (2 C), 125.7 (2 C), 104.8, 90.6, 80.8, 34.5, 34.3, 32.9, 31.4 (3 C), 29.7, 28.3 (3 C), 28.2.

HRMS (ESI-TOF): *m/z:* $[M+H]^+$ calcd for $C_{25}H_{33}N_2O_5^+$: 441.2384, found 441.2388.

$[\alpha\}_D^{20}$(c = 1, $CHCl_3$, *e.r.* 75:25): -15.9

HPLC (YMC-SB, *n*-hexane/IPA = 1/1, flow = 1.0 mL min^{-1}, T_{Column} = 10 °C, *l* = 210 nm) t_r: 8.4 min (major), 19.5 min (minor).

Ethyl (*S,Z*)-2-(1,3-diisopropyl-5-(4-methoxyphenyl)-2,4-dioxo-1,2,3,4,5,6-hexahydro-7*H*-pyrano [2,3-d]pyrimidin-7-ylidene)acetate (14o)

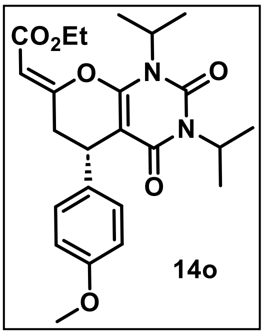

Product **14o** was synthesized according to general procedure **C** on a scale of 0.1 mmol. The compound was obtained as a colorless viscous residue in an isolated yield of 95% (42.0 mg with **ITU1**) and 98% (43.1 mg with **ISeU**) after preparative TLC (heptanes/EtOAc, 1/1, as eluent). The determined *e.r.* were 79:21 (**ITU1**) and 86:14 (**ISeU**).

^{1}H-NMR (300 MHz, $CDCl_3$, 298 K) δ / ppm = 7.07-7.02 (m, 2 H, Ar-**H**), 6.84-6.79 (m, 2 H, Ar-**H**), 5.30 (sep, J = 6.9 Hz, 1 H, -NC**H**), 5.14 (sep, J = 6.9 Hz, 1 H, -NC**H**), 5.08 (d, J = 1.9 Hz, 1 H, -C=C**H**), 4.20-4.14 (m, 1 H, -C**H**), 4.17 (q, J = 7.1 Hz, 2 H, -C**H₂**), 3.75 (s, 3 H, -OC**H₃**), 2.89 (ddd, J_1 = 14.8 Hz, J_2 = 6.7 Hz, J_3 = 1.9 Hz, 1 H, -C**H₂**), 2.55 (dd, J_1 = 14.8 Hz, J_2 = 1.7 Hz, 1 H, -C**H₂**), 1.64 (d, J = 6.9 Hz, 3 H, -C**H₃**), 1.59 (d, J = 6.9 Hz, 3 H, -C**H₃**), 1.41 (d, J = 6.9 Hz, 3 H, -C**H₃**), 1.40 (d, J = 6.9 Hz, 3 H, -C**H₃**), 1.26 (t, J = 7.1 Hz, 3 H, -C**H₃**).

^{13}C-NMR (75 MHz, $CDCl_3$, 298 K) δ / ppm = 163.2, 162.0, 158.7, 156.9, 153.5, 149.9, 133.9, 128.1 (2 C), 114.2 (2 C), 102.7, 91.2, 60.3, 55.3, 48.6, 46.0, 35.1, 32.7, 20.3, 19.9, 19.5, 19.4, 14.3.

HRMS (ESI-TOF): *m/z:* $[M+H]^+$ calcd for $C_{24}H_{31}N_2O_6^+$: 443.2177, found 443.2174.

$[\alpha]_D^{20}$(c = 1, $CHCl_3$, *e.r.* 86:14): -22.7

HPLC (YMC-SB, *n*-hexane/IPA = 1/1, flow = 1.0 mL min^{-1}, T_{Column} = 10 °C, l = 220 nm) t_r: 4.9 min (major), 6.6 min (minor).

Ethyl (*S,Z*)-2-(1,3-dibenzyl-5-(4-methoxyphenyl)-2,4-dioxo-1,2,3,4,5,6-hexahydro-7*H*-pyrano[2,3-*d*]pyrimidin-7-ylidene)acetate (14p)

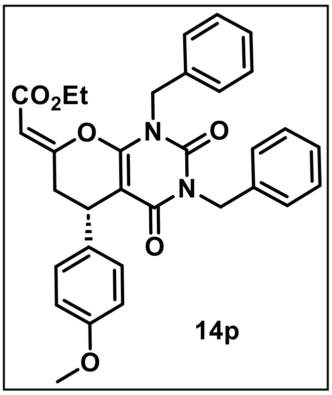

Product **14p** was synthesized according to general procedure **C** on a scale of 0.1 mmol. The compound was obtained as a colorless viscous residue in an isolated yield of 77% (41.5 mg with **ITU1**) and 82% (44.2 mg with **ISeU**) after preparative TLC (heptanes/EtOAc, 1/1, as eluent). The determined *e.r.* were 68:32 (**ITU1**) and 71:29 (**ISeU**).

^{1}H-NMR (300 MHz, $CDCl_3$, 298 K) δ / ppm = 7.52-7.49 (m, 2 H, Ar-**H**), 7.44-7.41 (m, 2 H, Ar-**H**), 7.35-7.22 (m, 6 H, Ar-**H**), 7.08-7.04 (m, 2 H, Ar-**H**), 6.85-6.81 (m, 2 H, Ar-**H**), 5.47 (d, J = 14.4 Hz, 1 H, N-C**H₂**), 5.29 (d, J = 14.4 Hz, 1 H, N-C**H₂**), 5.15 (d, J = 13.8 Hz, 1 H, N-C**H₂**), 5.14 (d, J = 1.9 Hz, 1 H, -C=C**H**), 5.02 (d, J = 13.8 Hz, 1 H, N-C**H₂**), 4.18 (q, J = 7.1 Hz, 2 H, -C**H₂**), 4.17-4.16 (m, 1 H, -C**H**), 3.77 (s, 3 H, -OC**H₃**), 2.84 (ddd, J_1 = 14.8 Hz, J_2 = 6.8 Hz, J_3 = 1.9 Hz, 1 H, -C**H₂**), 2.54 (dd, J_1 = 14.8 Hz, J_2 = 1.7 Hz, 1 H, -C**H₂**), 1.26 (t, J = 7.1 Hz, 3 H, -C**H₃**).

^{13}C-NMR (75 MHz, $CDCl_3$, 298 K) δ / ppm = 163.3, 161.5, 158.8, 156.9, 153.0, 150.9, 137.0, 136.5, 133.8, 129.1 (2 C), 128.9 (2 C), 128.8 (2 C), 128.5 (2 C), 128.2 (2 C), 128.1, 127.7, 114.3 (2 C), 103.4, 90.9, 60.5, 55.4, 46.0, 44.9, 34.9, 32.8, 14.4.

HRMS (ESI-TOF): *m/z:* $[M+H]^+$ calcd for $C_{32}H_{31}N_2O_6^+$: 539.2177, found 539.2179.

$[\alpha]_D^{20}$(c = 1, $CHCl_3$, *e.r.* 68:32): -6.5

HPLC (YMC-SB, *n*-hexane/IPA = 1/1, flow = 0.5 mL min^{-1}, T_{Column} = 10 °C, *l* = 210 nm) t_r: 23.3 min (major), 25.9 min (minor).

Ethyl (*S,Z*)-2-(5-(4-methoxyphenyl)-2,4-dioxo-1,3-diphenyl-1,2,3,4,5,6-hexahydro-7*H*-pyrano[2,3-d]pyrimidin-7-ylidene)acetate (14q)

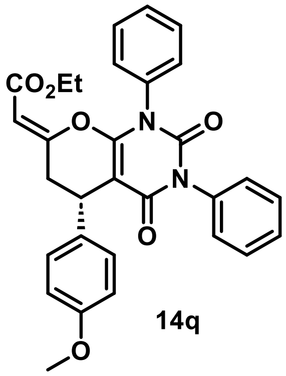

Product **14q** was synthesized according to general procedure **C** on a scale of 0.1 mmol. The compound was obtained as a colorless viscous residue in an isolated yield of 76% (33.6 mg with **ITU1**) and 97% (42.9 mg with **ISeU**) after preparative TLC (heptanes/EtOAc, 1/1, as eluent). The determined *e.r.* were 55:45 (**ITU1**) and 65:35 (**ISeU**).

^{1}H-NMR (300 MHz, $CDCl_3$, 298 K) δ / ppm = 7.54-7.34 (m, 8 H, Ar-**H**), 7.27-7.24 (m, 2 H, Ar-**H**), 7.20-7.16 (m, 2 H, Ar-**H**), 6.88-6.83 (m, 2 H, Ar-**H**), 5.06 (d, *J* = 1.7 Hz, 1 H, -C=C**H**), 4.28 (dd, J_1 = 6.7 Hz, J_2 = 1.6 Hz, 1 H, -C**H**), 3.90 (q, *J* = 7.1 Hz, 2 H, -C**H₂**), 3.78 (s, 3 H, -OC**H₃**), 2.97 (ddd, J_1 = 14.7 Hz, J_2 = 6.7 Hz, J_3 = 1.7 Hz, 1 H, -C**H₂**), 2.59 (dd, J_1 = 14.7 Hz, J_2 = 1.6 Hz, 1 H, -C**H₂**), 1.08 (t, *J* = 7.1 Hz, 3 H, -C**H₃**).

^{13}C-NMR (75 MHz, $CDCl_3$, 298 K) δ / ppm = 162.9, 161.7, 158.8, 155.5, 153.4, 150.4, 134.8, 133.8, 133.5, 129.3, 129.2 (6 C), 128.7, 128.5 (2 C), 128.4 (2 C), 114.2 (2 C), 104.2, 91.6, 60.1, 55.3, 34.7, 33.0, 14.3.

HRMS (ESI-TOF): *m/z:* $[M+H]^+$ calcd for $C_{30}H_{27}N_2O_6^+$: 511.1864, found 511.1871.

$[\alpha]_D^{20}$(c = 1, $CHCl_3$, *e.r.* 65:35): -5.7

HPLC (YMC-SB, *n*-hexane/IPA = 1/1, flow = 1.0 mL min^{-1}, T_{Column} = 10 °C, *l* = 220 nm) t_r: 8.9 min (major), 19.5 min (minor).

Ethyl (*Z*)-2-((5*S*,6*S*)-1,3,6-trimethyl-2,4-dioxo-5-phenyl-1,2,3,4,5,6-hexahydro-7*H*-pyrano[2,3-*d*] pyrimidin-7-ylidene)acetate (14r$_{major}$)

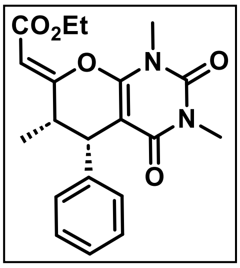

Product **14r$_{major}$** was synthesized according to general procedure **C** on a scale of 0.1 mmol. The compound was obtained as a colorless viscous residue in an isolated yield of 47% (17.6 mg with **ITU1**) and 74% (27.7 mg with **ISeU**) after preparative TLC (heptanes/EtOAc, 1/1, as eluent). The determined *e.r.* were 99:1 (**ITU1**) and 99:1 (**ISeU**).

^{1}H-NMR (300 MHz, $CDCl_3$, 298 K) *δ* / ppm = 7.29-7.18 (m, 3 H, Ar-**H**), 7.08-7.04 (m, 2 H, Ar-**H**), 5.16 (d, *J* = 1.8 Hz, 1 H, -C=C**H**), 4.21 (q, *J* = 7.1 Hz, 2 H, -C**H$_2$**), 4.01 (d, *J* = 6.2 Hz, 1 H, Ph-C**H**), 3.63 (s, 3 H, -NC**H$_3$**), 3.27 (s, 3 H, -NC**H$_3$**), 3.01-2.92 (m, 1 H, CH_3-C**H**), 1.30 (t, *J* = 7.1 Hz, 3 H, CH_2-C**H$_3$**), 0.96 (d, *J* = 6.7 Hz, 3 H, CH-C**H$_3$**).

^{13}C-NMR (75 MHz, $CDCl_3$, 298 K) *δ* / ppm = 163.7, 161.9, 161.6, 152.7, 151.0, 138.2, 128.6 (2 C), 128.5 (2 C), 127.5, 101.3, 91.8, 60.4, 40.0, 35.4, 29.6, 28.2, 14.4, 14.1.

HRMS (ESI-TOF): *m/z:* $[M+H]^+$ calcd for $C_{20}H_{23}N_2O_5^+$: 371.1601, found 371.1607.

$[\alpha]_D^{20}$(c = 1, $CHCl_3$, *e.r.* 99:1): +24.4

HPLC (YMC-SB, *n*-hexane/IPA = 1:1, flow = 1.0 mL min^{-1}, T_{Column} = 10 °C, *l* = 220 nm) t_r: 9.2 min (major), 16.3 min (minor).

Ethyl (Z)-2-((5S,6R)-1,3,6-trimethyl-2,4-dioxo-5-phenyl-1,2,3,4,5,6-hexahydro-7H-pyrano[2,3-d]pyrimidin-7-ylidene)acetate (14r$_{minor}$)

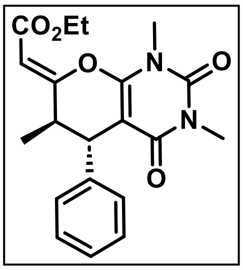

Product **14r$_{minor}$** was synthesized according to general procedure **C** on a scale of 0.1 mmol. The compound was obtained as a colorless viscous residue in an isolated yield of 21% (7.7 mg with **ITU1**) and 19% (7.1 mg with **ISeU**) after preparative TLC (heptanes/EtOAc, 1/1, as eluent). The determined *e.r.* were 68:32 (**ITU1**) and 71:29 (**ISeU**).

^{1}H-NMR (300 MHz, $CDCl_3$, 298 K) *δ* / ppm = 7.31-7.18 (m, 3 H, Ar-**H**), 7.13-7.10 (m, 2 H, Ar-**H**), 5.14 (s, 1 H, -C=C**H**), 4.18 (q, *J* = 7.1 Hz, 2 H, -C**H$_2$**), 3.90 (d, *J* = 1.3 Hz, 1 H, Ph-C**H**), 3.64 (s, 3 H, -NC**H$_3$**), 3.31 (s, 3 H, -NC**H$_3$**), 2.70 (qd, J_1 = 7.1 Hz, J_2 = 1.3 Hz, 1 H, CH_3-C**H**), 1.33 (d, *J* = 7.1 Hz, 3 H, CH-C**H$_3$**), 1.28 (t, *J* = 7.1 Hz, 3 H, CH_2-C**H$_3$**).

^{13}C-NMR (75 MHz, $CDCl_3$, 298 K) *δ* / ppm = 163.6, 162.3, 161.2, 152.3, 151.0, 142.3, 128.9 (2 C), 127.4, 127.1 (2 C), 102.6, 88.3, 60.4, 41.3, 39.9, 29.6, 28.3, 20.7, 14.4.

HRMS (ESI-TOF): *m/z:* $[M+H]^+$ calcd for $C_{20}H_{23}N_2O_5^+$: 371.1601, found 371.1605.

$[\alpha]_D^{20}$(c = 1, $CHCl_3$, *e.r.* 71:29): +2.2

HPLC (YMC-SB, *n*-hexane/IPA = 1:1, flow = 1.0 mL min^{-1}, T_{Column} = 10 °C, *l* = 220 nm) t_r: 6.4 min (major), 10.1 min (minor).

Ethyl (*Z*)-2-((5*S*,6*S*)-6-butyl-1,3-dimethyl-2,4-dioxo-5-phenyl-1,2,3,4,5,6-hexahydro-7*H*-pyrano [2,3-*d*] pyrimidin-7-ylidene)acetate (14s$_{major}$)

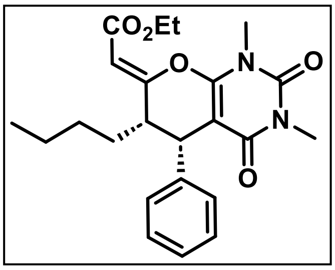

Product **14s$_{major}$** was synthesized according to general procedure **C** on a scale of 0.1 mmol, but with a reaction time of 24 h. The compound was obtained as a colorless viscous residue in an isolated yield of 51% (21.1 mg with **ITU1**) and 68% (28.3 mg with **ISeU**) after preparative TLC (heptanes/EtOAc, 1/1, as eluent). The determined *e.r.* were 99:1 (**ITU1**) and 99:1 (**ISeU**).

^{1}H-NMR (300 MHz, $CDCl_3$, 298 K) δ / ppm = 7.29-7.21 (m, 3 H, Ar-**H**), 7.12-7.09 (m, 2 H, Ar-**H**), 5.20 (d, J = 1.7 Hz, 1 H, -C=C**H**), 4.22 (q, J = 7.2 Hz, 2 H, -C**H$_2$**), 4.16 (d, J = 5.9 Hz, 1 H, Ph-C**H**), 3.62 (s, 3 H, -NC**H$_3$**), 3.28 (s, 3 H, -NC**H$_3$**), 2.76-2.71 (m, 1 H, CH_2-C**H**), 1.43-1.26 (m, 4 H, -C**H$_2$**), 1.32 (t, J = 7.2 Hz, 3 H, CH_2-C**H$_3$**), 1.23-1.09 (m, 2 H, -C**H$_2$**), 0.88 (t, J = 7.1 Hz, 3 H, CH_2-CH_2-C**H$_3$**).

^{13}C-NMR (75 MHz, $CDCl_3$, 298 K) δ / ppm = 163.8, 161.8, 161.5, 152.7, 151.0, 138.4, 128.7 (2 C), 128.5 (2 C), 127.5, 101.9, 92.1, 60.5, 40.8, 37.7, 29.7, 29.0, 28.2, 26.9, 22.6, 14.4, 14.0.

HRMS (ESI-TOF): *m/z:* $[M+H]^+$ calcd for $C_{23}H_{29}N_2O_5^+$: 413.2071, found 413.2074.

$[\alpha]_D^{20}$(c = 1, $CHCl_3$, *e.r.* 99:1): +42.0

HPLC (YMC-SB, *n*-hexane/IPA = 1:1, flow = 1.0 mL min^{-1}, T_{Column} = 10 °C, l = 220 nm) t_r: 7.2 min (minor), 8.4 min (major).

Ethyl (*Z*)-2-((5*S*,6*R*)-6-butyl-1,3-dimethyl-2,4-dioxo-5-phenyl-1,2,3,4,5,6-hexahydro-7*H*-pyrano[2,3-*d*]pyrimidin-7-ylidene)acetate (14s$_{minor}$)

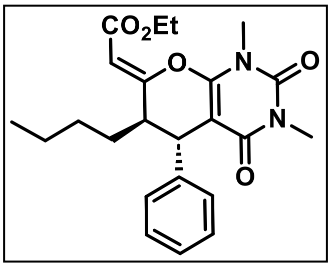

Product **14s$_{minor}$** was synthesized according to general procedure **C** on a scale of 0.1 mmol, but with a reaction time of 24 h. The compound was obtained as a colorless viscous residue in an isolated yield of 37% (15.3 mg with **ITU1**) and 30% (12.4 mg with **ISeU**) after preparative TLC (heptanes/EtOAc, 1/1, as eluent). The determined *e.r.* were 52:48 (**ITU1**) and 52:48 (**ISeU**).

^{1}H-NMR (300 MHz, $CDCl_3$, 298 K) δ / ppm = 7.31-7.25 (m, 2 H, Ar-**H**), 7.23-7.17 (m, 1 H, Ar-**H**), 7.12-7.08 (m, 2 H, Ar-**H**), 5.08 (s, 1 H, -C=C**H**), 4.18 (q, J = 7.1 Hz, 2 H, -C**H$_2$**), 4.01 (bs, 1 H, Ph-C**H**), 3.63 (s, 3 H, -NC**H$_3$**), 3.31 (s, 3 H, -NC**H$_3$**), 2.48-2.43 (m, 1 H, CH_2-C**H**), 1.65-1.45 (m, 3 H, -C**H$_2$**), 1.43-1.34 (m, 3 H, -C**H$_2$**), 1.29 (t, J = 7.1 Hz, 3 H, CH_2-CH_2-C**H$_3$**), 0.91 (t, J = 7.1 Hz, 3 H, CH_2-C**H$_3$**).

^{13}C-NMR (75 MHz, $CDCl_3$, 298 K) δ / ppm = 163.4, 162.3, 159.9, 152.5, 150.9, 142.4, 128.8 (2 C), 127.3, 127.1 (2 C), 103.6, 88.4, 60.4, 45.7, 39.9, 33.5, 29.6, 29.5, 28.3, 22.4, 14.3, 14.0.

HRMS (ESI-TOF): *m/z:* $[M+H]^+$ calcd for $C_{23}H_{29}N_2O_5^+$: 413.2071, found 413.2075.

$[\alpha]_D^{20}$(c = 1, $CHCl_3$, *e.r.* 52:48): +4.7

HPLC (YMC-SB, *n*-hexane/IPA = 1:1, flow = 1.0 mL min^{-1}, T_{Column} = 10 °C, *l* = 220 nm) t_r: 4.9 min (major), 6.4 min (minor).

Diethyl (S,*Z*)-2-(5-(4-methoxyphenyl)-1,3-dimethyl-2,4-dioxo-1,2,3,4,5,6-hexahydro-7*H*-pyrano[2,3-*d*]pyrimidin-7-ylidene)succinate (15a)

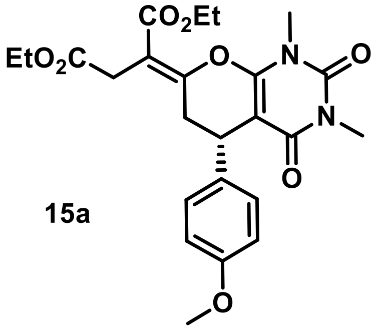

15a

Product **15a** was synthesized according to general procedure **C** on a scale of 0.1 mmol. The compound was obtained as a colorless viscous residue in a yield of 48% (22.6 mg with **ITU1**) and 80% (37.8 mg with **ISeU**) after preparative TLC (heptanes/EtOAc, 1/1, as eluent). The determined *e.r.* were 82:18 (**ITU1**) and 82:18 (**ISeU**).

^{1}H-NMR (300 MHz, $CDCl_3$, 298 K) δ / ppm = 7.10 – 7.04 (m, 2 H, Ar-H), 6.81 – 6.75 (m, 2 H, Ar-H), 4.24 (q, *J* = 7.1 Hz, 2 H, CH_2), 4.22 – 4.18 (m, 1 H, =C-CH), 4.03 (m, 2 H, CH_2), 3.74 (s, 3 H, O-CH_3), 3.56 (s, 3 H, N-CH_3), 3.29 (s, 3 H, N-CH_3), 3.21 (d, *J* = 17.3 Hz, 1 H, CH_2-COO), 2.97 (d, *J* = 17.3 Hz, 1 H, CH_2-COO), 2.92 (dd, J_1 = 15.0 Hz, J_2 = 2.0 Hz, 1 H, =C-CH_2), 2.74 (dd, J_1 = 15.0 Hz, J_2 = 6.5 Hz, 1 H, =C-CH_2), 1.29 (t, *J* = 7.1 Hz, 3 H, CH_3), 1.18 (t, *J* = 7.1 Hz, 3 H, CH_3).

^{13}C-NMR (75 MHz, $CDCl_3$, 298 K) δ / ppm = 170.1, 164.7, 161.8, 158.7, 153.8, 153.3, 150.9, 133.8, 128.0 (2 C), 114.2 (2 C), 110.0, 90.2, 61.3, 61.2, 55.3, 33.8, 33.0, 31.7, 29.6, 28.2, 14.3, 14.2.

HRMS (ESI-TOF): *m/z:* $[M+H]^+$ calcd for $C_{24}H_{29}N_2O_8^+$: 473.1918, found: 473.1918.

$[\alpha]_D^{20}$(c = 1, $CHCl_3$, *e.r.* 82:18): +27.2

HPLC (YMC-SA, *n*-hexane/IPA = 7/3, flow = 1.0 mL min^{-1}, T_{Column} = 20 °C, *l* = 269 nm) t_r: 9.8 min (minor), 12.5 min (major).

Diethyl (*S*,*Z*)-2-(1,3-dimethyl-2,4-dioxo-5-phenyl-1,2,3,4,5,6-hexahydro-7*H*-pyrano[2,3-*d*]pyrimidin-7-ylidene)succinate (15b)

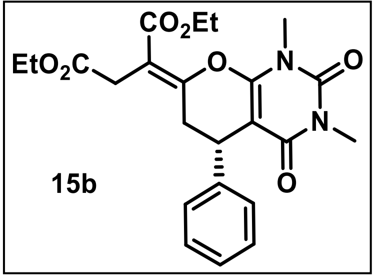

15b

Product **15b** was synthesized according to general procedure **C** on a scale of 0.1 mmol. The compound was obtained as a colorless viscous residue in a yield of 53% (23.5 mg with **ITU1**) and 64% (28.6 mg with **ISeU**) after preparative TLC (heptanes/EtOAc, 1/1, as eluent). The determined *e.r.* were 86:14 (**ITU1**) and 86:14 (**ISeU**).

^{1}H-NMR (300 MHz, $CDCl_3$, 298 K) δ / ppm = 7.29 – 7.13 (m, 5 H, Ar-H), 4.28 – 4.24 (m, 1 H, =C-CH), 4.24 (q, J = 7.1 Hz, 2 H, CH_2), 4.08 – 3.94 (m, 2 H, CH_2), 3.57 (s, 3 H, N-CH_3), 3.30 (s, 3 H, N-CH_3), 3.19 (d, J = 17.2 Hz, 1 H, CH_2-COO), 2.95 (d, J = 17.2 Hz, 1 H, CH_2-COO), 2.94 (dd, J_1 = 15.0 Hz, J_2 = 2.0 Hz, 1 H, =C-CH_2), 2.77 (dd, J_1 = 15.0 Hz, J_2 = 6.5 Hz, 1 H, =C-CH_2), 1.29 (t, J = 7.1 Hz, 3 H, CH_3), 1.17 (t, J = 7.1 Hz, 3 H, CH_3).

^{13}C-NMR (75 MHz, $CDCl_3$, 298 K) δ / ppm = 170.1, 164.7, 161.8, 153.7, 153.5, 151.0, 141.7, 128.8 (2 C), 127.3, 127.0 (2 C), 110.1, 89.9, 61.3, 61.2, 33.8, 33.7, 31.6, 29.6, 28.2, 14.3, 14.2.

HRMS (ESI-TOF): *m/z:* $[M+H]^+$ calcd for $C_{23}H_{27}N_2O_7^+$: 443.1813, found 443.1813.

$[\alpha]_D^{20}$(c = 1, $CHCl_3$, *e.r.* 86:14): +26.0

HPLC (YMC-SA, *n*-hexane/IPA = 7/3, flow = 1.0 mL min^{-1}, T_{Column} = 20 °C, *l* = 269 nm) t_r: 7.6 min (minor), 8.4 min (major).

Diethyl (*S*,*Z*)-2-(5-(4-(dimethylamino)phenyl)-1,3-dimethyl-2,4-dioxo-1,2,3,4,5,6-hexahydro-7*H*-pyrano[2,3-*d*]pyrimidin-7-ylidene)succinate (15c)

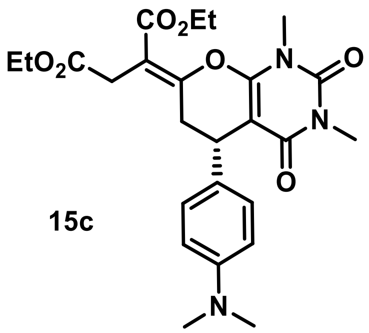

15c

Product **15c** was synthesized according to general procedure **C** on a scale of 0.1 mmol. The compound was obtained as a colorless viscous residue in a yield of 45% (22.0 mg with **ITU1**) and 62% (30.1 mg with **ISeU**) after preparative TLC (heptanes/EtOAc, 1/1, as eluent). The determined *e.r.* were 76:24 (**ITU1**) and 69:31 (**ISeU**).

^{1}H-NMR (300 MHz, $CDCl_3$, 298 K) δ / ppm = 7.05 – 7.00 (m, 2 H, Ar-H), 6.64 – 6.59 (m, 2 H, Ar-H), 4.25 (q, J = 7.1 Hz, 2 H, CH_2), 4.19 – 4.15 (m, 1 H, =C-CH), 4.09 – 4.01 (m, 2 H, CH_2), 3.56 (s, 3 H, N-CH_3), 3.29 (s, 3 H, N-CH_3), 3.27 (d, J = 17.1 Hz, 1 H, CH_2-COO), 3.01 (d, J = 17.1 Hz, 1 H, CH_2-COO), 2.94 (dd, J_1 = 15.1 Hz, J_2 = 2.0 Hz, 1 H, =C-CH_2), 2.88 (s, 6 H, $N(CH_3)_2$), 2.73 (dd, J_1 = 15.1 Hz, J_2 = 6.6 Hz, 1 H, =C-CH_2), 1.30 (t, J = 7.1 Hz, 3 H, CH_3), 1.19 (t, J = 7.1 Hz, 3 H, CH_3).

^{13}C-NMR (75 MHz, $CDCl_3$, 298 K) δ / ppm = 170.3, 164.9, 161.8, 154.5, 153.1, 151.0, 149.8, 129.6, 127.6 (2 C), 112.9 (2 C), 109.6, 90.7, 61.2 (2 C), 40.7 (2 C), 33.9, 32.8, 31.7, 29.6, 28.2, 14.4, 14.2.

HRMS (ESI-TOF): *m/z:* $[M+H]^+$ calcd for $C_{25}H_{32}N_3O_7^+$: 486.2235, found 486.2236.

$[\alpha]_D^{20}$(c = 1, $CHCl_3$, *e.r.* 86:14): +20.3

HPLC (YMC-SA, *n*-hexane/IPA = 7/3, flow = 1.0 mL min^{-1}, T_{Column} = 20 °C, *l* = 269 nm) t_r: 8.8 min (minor), 11.8 min (major).

Diethyl (*S*,*Z*)-2-(5-(4-fluorophenyl)-1,3-dimethyl-2,4-dioxo-1,2,3,4,5,6-hexahydro-7*H*-pyrano[2,3-*d*]pyrimidin-7-ylidene)succinate (15d)

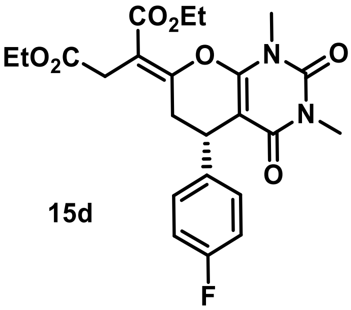
15d

Product **15d** was synthesized according to general procedure **C** on a scale of 0.1 mmol. The compound was obtained as a colorless viscous residue in a yield of 50% (23.2 mg with **ITU1**) and 52% (23.8 mg with **ISeU**) after preparative TLC (heptanes/EtOAc, 1/1, as eluent). The determined *e.r.* were 85:15 (**ITU1**) and 86:14 (**ISeU**).

^{1}H-NMR (300 MHz, $CDCl_3$, 298 K) δ / ppm = 7.16 – 7.10 (m, 2 H, Ar-H), 6.98 – 6.91 (m, 2 H, Ar-H), 4.26 (m, 1 H, =C-CH), 4.25 (q, J = 7.1 Hz, 2 H, CH_2), 4.09 – 3.98 (m, 2 H, CH_2), 3.57 (s, 3 H, N-CH_3), 3.30 (s, 3 H, N-CH_3), 3.18 (d, J = 17.2 Hz, 1 H, CH_2-COO), 2.97 (d, J = 17.2 Hz, 1 H, CH_2-COO), 2.90 (dd, J_1 = 15.1 Hz, J_2 = 2.1 Hz, 1 H, =C-CH_2), 2.76 (dd, J_1 = 15.1 Hz, J_2 = 6.4 Hz, 1 H, =C-CH_2), 1.30 (t, J = 7.1 Hz, 3 H, CH_3), 1.18 (t, J = 7.1 Hz, 3 H, CH_3).

^{13}C-NMR (75 MHz, $CDCl_3$, 298 K) δ / ppm = 170.0, 164.6, 162.0 (d, $^1J_{C\text{-}F}$ = 245.5 Hz), 161.8, 153.6, 153.3, 150.9, 137.5 (d, $^4J_{C\text{-}F}$ = 3.2 Hz), 128.6 (d, $^3J_{C\text{-}F}$ = 8.1 Hz, 2 C), 115.7 (d, $^2J_{C\text{-}F}$ = 21.5 Hz, 2 C), 110.5, 89.8, 61.3, 61.2, 33.7, 33.3, 31.6, 29.6, 28.2, 14.3, 14.2.

^{19}F-NMR (282 MHz, $CDCl_3$, 298 K) δ / ppm = -115.5.

HRMS (ESI-TOF): *m/z:* $[M+H]^+$ calcd for $C_{23}H_{26}FN_2O_7^+$: 461.1719, found 461.1718.

$[\alpha]_D^{20}$(c = 1, $CHCl_3$, *e.r.* 85:15): +20.7

HPLC (YMC-SA, *n*-hexane/IPA = 7/3, flow = 1.0 mL min^{-1}, T_{Column} = 20 °C, l = 269 nm) t_r: 7.0 min (minor), 9.5 min (major).

Diethyl (*R*,*Z*)-2-(5-(2-fluorophenyl)-1,3-dimethyl-2,4-dioxo-1,2,3,4,5,6-hexahydro-7*H*-pyrano[2,3-*d*]pyrimidin-7-ylidene)succinate (15e)

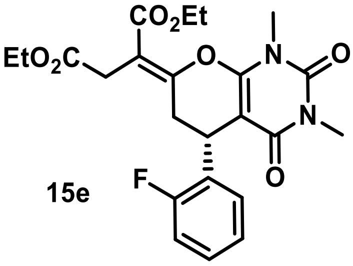
15e

Product **15e** was synthesized according to general procedure **C** on a scale of 0.1 mmol. The compound was obtained as a colorless viscous residue in a yield of 30% (13.7 mg with **ITU1**) and 92% (42.6 mg with **ISeU**) after preparative TLC (heptanes/EtOAc, 1/1, as eluent). The determined *e.r.* were 75:25 (**ITU1**) and 72:28 (**ISeU**).

^{1}H-NMR (300 MHz, $CDCl_3$, 298 K) δ / ppm = 7.23 – 7.14 (m, 1 H, Ar-H), 7.06 – 6.97 (m, 2 H, Ar-H), 6.92 – 6.85 (m, 1 H, Ar-H), 4.59 (dd, J_1 = 6.4 Hz, J_2 = 2.0 Hz, 1 H, =C-CH), 4.22 (q, J = 7.1 Hz, 2 H, CH_2), 4.05 – 3.94 (m, 2 H, CH_2), 3.57 (s, 3 H, N-CH_3), 3.30 (s, 3 H, N-CH_3), 3.12 (d, J = 17.2 Hz, 1 H, CH_2-COO), 3.00 (dd, J_1 = 15.1 Hz, J_2 = 2.0 Hz, 1 H, =C-CH_2), 2.93 (d, J = 17.2 Hz, 1 H, CH_2-COO), 2.75 (dd, J_1 = 15.1 Hz, J_2 = 6.4 Hz, 1 H, =C-CH_2), 1.27 (t, J = 7.1 Hz, 3 H, CH_3), 1.16 (t, J = 7.1 Hz, 3 H, CH_3).

^{13}C-NMR (75 MHz, $CDCl_3$, 298 K) δ / ppm = 170.0, 164.6, 161.6, 160.4 (d, $^1J_{C\text{-}F}$ = 246.0 Hz), 154.3, 153.3, 151.0, 129.0 (d, $^3J_{C\text{-}F}$ = 8.3 Hz), 128.4 (d, $^3J_{C\text{-}F}$ = 3.9 Hz), 128.1 (d, $^2J_{C\text{-}F}$ = 13.7 Hz), 124.3 (d, $^4J_{C\text{-}F}$ = 3.2 Hz), 115.7 (d, $^2J_{C\text{-}F}$ = 21.5 Hz), 110.4, 88.3, 61.2, 61.2, 33.6, 30.5, 29.6, 28.2, 27.8 (d, $^3J_{C\text{-}F}$ = 3.4 Hz), 14.3, 14.2.

^{19}F-NMR (282 MHz, $CDCl_3$, 298 K) δ / ppm = -118.7.

HRMS (ESI-TOF): *m/z:* $[M+H]^+$ calcd for $C_{23}H_{26}FN_2O_7^+$: 461.1719, found 461.1719.

$[\alpha]_D^{20}$(c = 1, $CHCl_3$, *e.r.* 85:15): -1.0

HPLC (YMC-SB, *n*-hexane/IPA = 7/3, flow = 1.0 mL min^{-1}, T_{Column} = 20 °C, *l* = 269 nm) t_r: 16.3 min (major), 19.9 min (minor).

Diethyl (*S*,*Z*)-2-(5-(4-bromophenyl)-1,3-dimethyl-2,4-dioxo-1,2,3,4,5,6-hexahydro-7*H*-pyrano[2,3-*d*]pyrimidin-7-ylidene)succinate (15f)

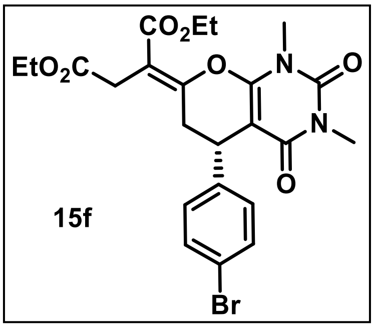

Product **15f** was synthesized according to general procedure **C** on a scale of 0.1 mmol. The compound was obtained as a colorless viscous residue in a yield of 82% (42.6 mg with **ITU1**) and 73% (38.2 mg with **ISeU**) after preparative TLC (heptanes/EtOAc, 1/1, as eluent). The determined *e.r.* were 81:19 (**ITU1**) and 79:21 (**ISeU**).

^{1}H-NMR (300 MHz, $CDCl_3$, 298 K) δ / ppm = 7.41 – 7.35 (m, 2 H, Ar-H), 7.07 – 7.01 (m, 2 H, Ar-H), 4.24 (q, J = 7.1 Hz, 2 H, CH_2), 4.23 – 4.18 (m, 1 H, =C-CH), 4.09 – 3.96 (m, 2 H, CH_2), 3.56 (s, 3 H, N-CH_3), 3.29 (s, 3 H, N-CH_3), 3.18 (d, J = 17.1 Hz, 1 H, CH_2-COO), 2.98 (d, J = 17.1 Hz, 1 H, CH_2-COO), 2.90 (dd, J_1 = 15.1 Hz, J_2 = 2.2 Hz, 1 H, =C-CH_2), 2.77 (dd, J_1 = 15.1 Hz, J_2 = 6.4 Hz, 1 H, =C-CH_2), 1.30 (t, J = 7.1 Hz, 3 H, CH_3), 1.18 (t, J = 7.1 Hz, 3 H, CH_3).

^{13}C-NMR (75 MHz, $CDCl_3$, 298 K) δ / ppm = 169.9, 164.6, 161.7, 153.7, 153.0, 150.9, 140.8, 131.9 (2 C), 128.8 (2 C), 121.3, 110.6, 89.4, 61.3 (2 C), 33.8, 33.5, 31.4, 29.6, 28.2, 14.3, 14.2.

HRMS (ESI-TOF): *m/z:* $[M+H]^+$ calcd for $C_{23}H_{26}{}^{81}BrN_2O_7^+$: 523.0897 (^{81}Br isotope), found 523.0895 (^{81}Br isotope).

$[\alpha]_D^{20}$(c = 1, $CHCl_3$, *e.r.* 81:19): +23.4

HPLC (YMC-SA, *n*-hexane/IPA = 7/3, flow = 1.0 mL min^{-1}, T_{Column} = 20 °C, *l* = 269 nm) t_r: 8.1 min (minor), 12.4 min (major).

Diethyl (*R*,*Z*)-2-(1,3-dimethyl-2,4-dioxo-5-(perfluorophenyl)-1,2,3,4,5,6-hexahydro-7*H*-pyrano[2,3-*d*]pyrimidin-7-ylidene)succinate (15g)

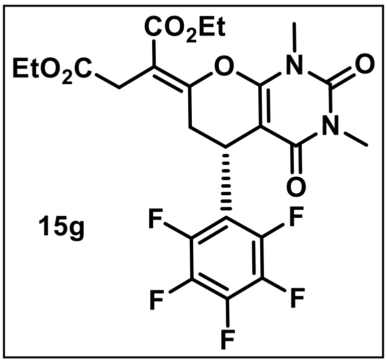

Product **15g** was synthesized according to general procedure **C** on a scale of 0.1 mmol. The compound was obtained as a colorless viscous residue in a yield of 29% (15.6 mg with **ITU1**) and 46% (24.5 mg with **ISeU**) after preparative TLC (heptanes/EtOAc, 1/1, as eluent). The determined *e.r.* were 86:14 (**ITU1**) and 88:12 (**ISeU**).

^{1}**H-NMR** (300 MHz, $CDCl_3$, 298 K) δ / ppm = 4.59 (t, J = 5.3 Hz, 1 H, =C-CH), 4.27 (q, J = 7.1 Hz, 2 H, CH_2), 4.06 (q, J = 7.1 Hz, 2 H, CH_2), 3.57 (s, 3 H, N-CH_3), 3.29 (s, 3 H, N-CH_3), 3.29 (d, J = 17.1 Hz, 1 H, CH_2-COO), 3.21 (d, J = 17.1 Hz, 1 H, CH_2-COO), 2.92 (d, J = 5.3 Hz, 2 H, =C-CH_2), 1.31 (t, J = 7.1 Hz, 3 H, CH_3), 1.21 (t, J = 7.1 Hz, 3 H, CH_3).

^{13}**C-NMR** (175 MHz, $CDCl_3$, 298 K) δ / ppm = 169.9, 164.5, 161.6, 154.3, 152.6, 150.7, 146.1 – 144.6 (m, 2 C), 141.4 – 139.7 (m), 138.6 – 136.9 (m, 2 C), 115.0 – 114.8 (m), 109.8, 86.7, 61.5, 61.4, 33.9, 29.7, 29.3, 28.2, 24.5, 14.3, 14.2.

^{19}**F-NMR** (282 MHz, $CDCl_3$, 298 K) δ / ppm =-142.6 – -142.8 (m), -155.3 – -155.6 (m), -161.9 – -161.6 (m).

HRMS (ESI-TOF): *m/z:* $[M+H]^+$ calcd for $C_{23}H_{22}F_5N_2O_7^+$: 533.1342, found 533.1345.

$[\alpha]_D^{20}$(c = 1, $CHCl_3$, *e.r.* 86:14): +66.7

HPLC (YMC-SB, *n*-hexane/IPA = 4/1, flow = 1.0 mL min^{-1}, T_{Column} = 20 °C, l = 269 nm) t_r: 24.5 min (major), 26.7 min (minor).

Diethyl (*S*,*Z*)-2-(1,3-dimethyl-2,4-dioxo-5-(*p*-tolyl)-1,2,3,4,5,6-hexahydro-7*H*-pyrano[2,3-*d*]pyrimidin-7-ylidene)succinate (15h)

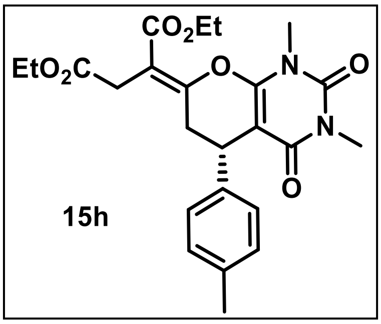

Product **15h** was synthesized according to general procedure **C** on a scale of 0.1 mmol. The compound was obtained as a colorless viscous residue in a yield of 66% (30.0 mg with **ITU1**) and 57% (26.5 mg with **ISeU**) after preparative TLC (heptanes/EtOAc, 1/1, as eluent). The determined *e.r.* were 84:16 (**ITU1**) and 84:16 (**ISeU**).

^{1}**H-NMR** (500 MHz, $CDCl_3$, 298 K) δ / ppm = 7.08 – 7.03 (m, 4 H, Ar-H), 4.25 (q, J = 7.1 Hz, 2 H, CH_2), 4.22 (dd, J_1 = 6.8 Hz, J_2 = 2.0 Hz, 1 H, =C-CH), 4.03 (m, 2 H, CH_2), 3.57 (s, 3 H, N-CH_3), 3.30 (s, 3 H, N-CH_3), 3.21 (d, J = 17.2 Hz, 1 H, CH_2-COO), 2.97 (d, J = 17.2 Hz, 1 H, CH_2-COO), 2.93 (dd, J_1 = 15.0 Hz, J_2 = 2.0 Hz, 1 H, =C-CH_2), 2.76 (dd, J_1 = 15.0 Hz, J_2 = 6.8 Hz, 1 H, =C-CH_2), 2.27 (s, 3 H, Ar-CH_3), 1.31 (t, J = 7.1 Hz, 3 H, CH_3), 1.19 (t, J = 7.1 Hz, 3 H, CH_3).

^{13}C-NMR (125 MHz, $CDCl_3$, 298 K) *δ* / ppm = 170.2, 164.8, 161.8, 153.9, 153.4, 151.0, 138.8, 136.9, 129.5 (2 C), 126.9 (2 C), 110.0, 90.2, 61.3, 61.2, 33.8, 33.5, 31.7, 29.6, 28.2, 21.1, 14.4, 14.2.

HRMS (ESI-TOF): *m/z:* $[M+H]^+$ calcd for $C_{24}H_{29}N_2O_7^+$: 457.1969, found 457.1969.

$[\alpha]_D^{20}$(c = 1, $CHCl_3$, *e.r.* 84:16): +28.9

HPLC (YMC-SA, *n*-hexane/IPA = 7/3, flow = 1.0 mL min^{-1}, T_{Column} = 20 °C, *l* = 269 nm) t_r: 7.0 min (minor), 8.3 min (major).

Diethyl (*S*,*Z*)-2-(1,3-dimethyl-2,4-dioxo-5-(o-tolyl)-1,2,3,4,5,6-hexahydro-7*H*-pyrano[2,3-*d*]pyrimidin-7-ylidene)succinate (15i)

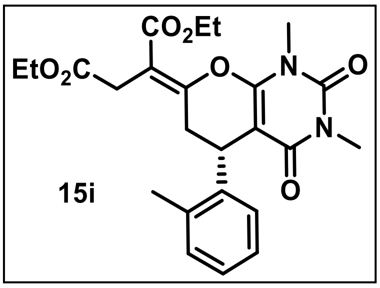

15i

Product **15i** was synthesized according to general procedure **C** on a scale of 0.1 mmol. The compound was obtained as a colorless viscous residue in a yield of 75% (34.1 mg with **ITU1**) and 62% (28.2 mg with **ISeU**) after preparative TLC (heptanes/EtOAc, 1/1, as eluent). The determined *e.r.* were 67:33 (**ITU1**) and 67:33 (**ISeU**).

^{1}H-NMR (300 MHz, $CDCl_3$, 298 K) *δ* / ppm = 7.17 – 7.10 (m, 3 H, Ar-H), 6.76 (dd, J_1 = 7.4 Hz, J_2 = 1.5 Hz, 1 H, Ar-H), 4.50 – 4.47 (m, 1 H, =C-CH), 4.25 (q, *J* = 7.1 Hz, 2 H, CH_2), 4.05 – 3.93 (m, 2 H, CH_2), 3.59 (s, 3 H, N-CH_3), 3.29 (s, 3 H, N-CH_3), 3.10 (d, *J* = 17.1 Hz, 1 H, CH_2-COO), 2.83 (d, *J* = 17.1 Hz, 1 H, CH_2-COO), 2.77 – 2.73 (m, 2 H, =C-CH_2), 2.51 (s, 3 H, Ar-CH_3), 1.30 (t, *J* = 7.1 Hz, 3 H, CH_3), 1.16 (t, *J* = 7.1 Hz, 3 H, CH_3).

^{13}C-NMR (75 MHz, $CDCl_3$, 298 K) *δ* / ppm = 170.1, 164.7, 161.7, 153.9, 153.4, 151.0, 139.5, 135.0, 130.9, 127.2, 126.3, 126.0, 110.5, 90.2, 61.3, 61.2, 33.7, 30.5, 30.0, 29.6, 28.2, 19.6, 14.3, 14.2.

HRMS (ESI-TOF): *m/z:* $[M+H]^+$ calcd for $C_{24}H_{29}N_2O_7^+$: 457.1969, found 457.1970.

$[\alpha]_D^{20}$(c = 1, $CHCl_3$, *e.r.* 67:33): +18.8

HPLC (YMC-SB, *n*-hexane/IPA = 4/1, flow = 1.0 mL min^{-1}, T_{Column} = 20 °C, *l* = 254 nm) t_r: 26.0 min (minor), 28.9 min (major).

Diethyl (*S*,*Z*)-2-(5-(4-(*tert*-butyl)phenyl)-1,3-dimethyl-2,4-dioxo-1,2,3,4,5,6-hexahydro-7*H*-pyrano[2,3-*d*]pyrimidin-7-ylidene)succinate (15j)

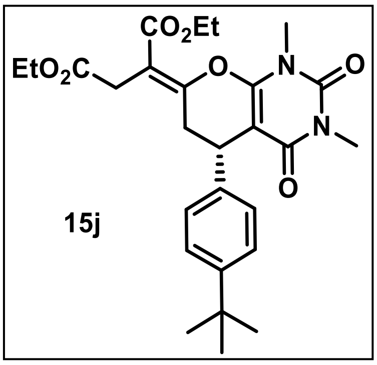

Product **15j** was synthesized according to general procedure **C** on a scale of 0.1 mmol. The compound was obtained as a colorless viscous residue in a yield of 41% (20.6 mg with **ITU1**) and 66% (32.9 mg with **ISeU**) after preparative TLC (heptanes/EtOAc, 1/1, as eluent). The determined *e.r.* were 85:15 (**ITU1**) and 85:15 (**ISeU**).

^{1}H-NMR (300 MHz, $CDCl_3$, 298 K) δ / ppm = 7.29 – 7.25 (m, 2 H, Ar-H), 7.12 – 7.07 (m, 2 H, Ar-H), 4.26 (q, *J* = 7.1 Hz, 2 H, CH_2), 4.25 – 4.21 (m, 1 H, =C-CH), 4.09 – 4.00 (m, 2 H, CH_2), 3.57 (s, 3 H, N-CH_3), 3.30 (s, 3 H, N-CH_3), 3.25 (d, *J* = 17.3 Hz, 1 H, CH_2-COO), 3.02 (d, *J* = 17.3 Hz, 1 H, CH_2-COO), 2.97 (dd, J_1 = 15.1 Hz, J_2 = 2.0 Hz, 1 H, =C-CH_2), 2.77 (dd, J_1 = 15.1 Hz, J_2 = 6.6 Hz, 1 H, =C-CH_2), 1.31 (t, *J* = 7.1 Hz, 3 H, CH_3), 1.25 (s, 9 H, $C(CH_3)_3$), 1.18 (t, *J* = 7.1 Hz, 3 H, CH_3).

^{13}C-NMR (75 MHz, $CDCl_3$, 298 K) δ / ppm = 170.2, 164.8, 161.9, 154.1, 153.2, 151.0, 150.0, 138.7, 126.6 (2 C), 125.7 (2 C), 109.7, 90.3, 61.3, 61.2, 34.5, 33.8, 33.1, 31.4 (3 C), 31.3, 29.6, 28.2, 14.3, 14.2.

HRMS (ESI-TOF): *m/z:* $[M+H]^+$ calcd for $C_{27}H_{35}N_2O_7^+$: 499.2439, found 499.2439.

$[\alpha]_D^{20}$(c = 1, $CHCl_3$, *e.r.* 85:15): +29.8

HPLC (YMC-SA, *n*-hexane/IPA = 7/3, flow = 1.0 mL min^{-1}, T_{Column} = 20 °C, *l* = 269 nm) t_r: 5.5 min (minor), 7.0 min (major).

Diethyl (*R*,*Z*)-2-(1,3-dimethyl-2,4-dioxo-5-(thiophen-2-yl)-1,2,3,4,5,6-hexahydro-7*H*-pyrano[2,3-*d*]pyrimidin-7-ylidene)succinate (15k)

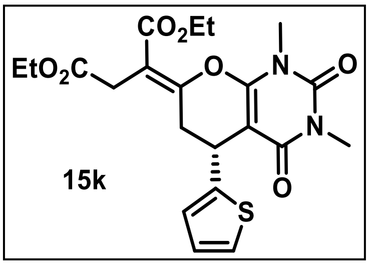

Product **15k** was synthesized according to general procedure **C** on a scale of 0.1 mmol. The compound was obtained as a colorless viscous residue in a yield of 37% (16.8 mg with **ITU1**) and 45% (20.4 mg with **ISeU**) after preparative TLC (heptanes/EtOAc, 1/1, as eluent). The determined *e.r.* were 78:22 (**ITU1**) and 67:33 (**ISeU**).

^{1}H-NMR (300 MHz, $CDCl_3$, 298 K) δ / ppm = 7.11 (dd, J_1 = 5.0 Hz, J_2 = 1.4 Hz, 1 H, Ar-H), 6.90 – 6.83 (m, 2 H, Ar-H), 4.59 – 4.54 (m, 1 H, =C-CH), 4.25 (q, *J* = 7.1 Hz, 2 H, CH_2), 4.15 – 4.11 (m, 2 H, CH_2), 3.54 (s, 3 H, N-CH_3), 3.39 (d, *J* = 17.3 Hz, 1 H, CH_2-COO), 3.33 (s, 3 H, N-CH_3), 3.14 (d, *J* = 17.3 Hz, 1 H, CH_2-COO), 3.11 (dd, J_1 = 15.1 Hz, J_2 = 2.0 Hz, 1 H, =C-CH_2), 2.76 (dd, J_1 = 15.1 Hz, J_2 = 6.2 Hz, 1 H, =C-CH_2), 1.30 (t, *J* = 7.1 Hz, 3 H, CH_3), 1.22 (t, *J* = 7.1 Hz, 3 H, CH_3).

^{13}C-NMR (75 MHz, $CDCl_3$, 298 K) δ / ppm = 170.2, 164.7, 161.7, 153.3, 153.2, 150.9, 145.0, 127.1, 124.8, 124.3, 110.7, 90.5, 61.3 (2 C), 34.0, 31.6, 29.6, 29.1, 28.3, 14.3, 14.2.

HRMS (ESI-TOF): *m/z:* $[M+H]^+$ calcd for for $C_{21}H_{25}N_2O_7S^+$: 449.1377, found 449.1378.

$[\alpha]_D^{20}$(c = 1, $CHCl_3$, *e.r.* 78:22): +2.3

HPLC (YMC-SB, *n*-hexane/IPA = 1/1, flow = 1.0 mL min^{-1}, T_{Column} = 20 °C, *l* = 269 nm) t_r: 21.9 min (major), 49.9 min (minor).

Diethyl (*S*,*Z*)-2-(1,3-dimethyl-5-(naphthalen-2-yl)-2,4-dioxo-1,2,3,4,5,6-hexahydro-7*H*-pyrano[2,3-*d*]pyrimidin-7-ylidene)succinate (15l)

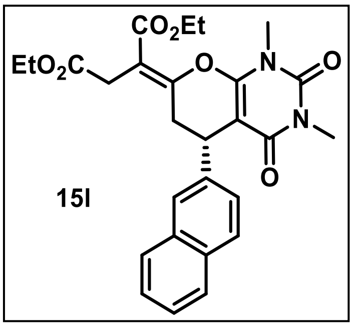

Product **15l** was synthesized according to general procedure **C** on a scale of 0.1 mmol. The compound was obtained as a colorless viscous residue in a yield of 70% (34.7 mg with **ITU1**) and 63% (31.3 mg with **ISeU**) after preparative TLC (heptanes/EtOAc, 1/1, as eluent). The determined *e.r.* were 77:23 (**ITU1**) and 75:25 (**ISeU**).

^{1}H-NMR (300 MHz, $CDCl_3$, 298 K) δ / ppm = 7.80 – 7.73 (m, 3 H, Ar-H), 7.57 – 7.54 (m, 1 H, Ar-H), 7.47 – 7.38 (m, 2 H, Ar-H), 7.32 (dd, J_1 = 8.5 Hz, J_2 = 1.8 Hz, 1 H, Ar-H), 4.43 (dd, J_1 = 6.5 Hz, J_2 = 2.0 Hz, 1 H, =C-CH), 4.25 (q, *J* = 7.1 Hz, 2 H, CH_2), 3.95 – 3.75 (m, 2 H, CH_2), 3.62 (s, 3 H, N-CH_3), 3.31 (s, 3 H, N-CH_3), 3.13 (d, *J* = 17.3 Hz, 1 H, CH_2-COO), 3.05 (dd, J_1 = 15.0 Hz, J_2 = 2.0 Hz, 1 H, =C-CH_2), 2.94 (d, *J* = 17.3 Hz, 1 H, CH_2-COO), 2.85 (dd, J_1 = 15.0 Hz, J_2 = 6.5 Hz, 1 H, =C-CH_2), 1.30 (t, *J* = 7.1 Hz, 3 H, CH_3), 1.05 (t, *J* = 7.1 Hz, 3 H, CH_3).

^{13}C-NMR (75 MHz, $CDCl_3$, 298 K) δ / ppm = 170.0, 164.7, 161.8, 153.7, 153.5, 151.0, 139.1, 133.5, 132.7, 128.7, 128.0, 127.6, 126.2, 125.9, 125.7, 125.2, 110.3, 89.8, 61.2, 61.1, 34.0, 33.8, 31.6, 29.6, 28.2, 14.3, 14.1.

HRMS (ESI-TOF): *m/z:* $[M+H]^+$ calcd for for $C_{27}H_{29}N_2O_7^+$: 493.1969, found 493.1971.

$[\alpha]_D^{20}$(c = 1, $CHCl_3$, *e.r.* 77:23): +48.7

HPLC (YMC-SA, *n*-hexane/IPA = 7/3, flow = 1.0 mL min^{-1}, T_{Column} = 20 °C, *l* = 269 nm) t_r: 9.2 min (minor), 12.1 min (major).

4-(*tert*-butyl) 1-ethyl (*S*,*Z*)-2-(5-(4-methoxyphenyl)-1,3-dimethyl-2,4-dioxo-1,2,3,4,5,6-hexahydro-7*H*-pyrano[2,3-*d*]pyrimidin-7-ylidene)succinate (15m)

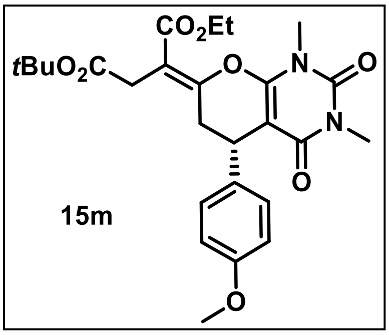

Product **15m** was synthesized according to general procedure **C** on a scale of 0.1 mmol. The compound was obtained as a colorless viscous residue in a yield of 58% (29.0 mg with **ITU1**) and 94% (47.3 mg with **ISeU**) after preparative TLC (heptanes/EtOAc, 1/1, as eluent). The determined *e.r.* were 86:14 (**ITU1**) and 82:18 (**ISeU**).

^{1}H-NMR (300 MHz, $CDCl_3$, 298 K) δ / ppm = 7.11 – 7.04 (m, 2 H, Ar-H), 6.81 – 6.75 (m, 2 H, Ar-H), 4.24 (q, J = 7.1 Hz, 2 H, CH_2), 4.23 – 4.19 (m, 1 H, =C-CH), 3.73 (s, 3 H, O-CH_3), 3.56 (s, 3 H, N-CH_3), 3.29 (s, 3 H, N-CH_3), 3.13 (d, J = 17.2 Hz, 1 H, CH_2-COO), 2.91 (dd, J_1 = 15.1 Hz, J_2 = 2.1 Hz, 1 H, =C-CH_2), 2.90 (d, J = 17.2 Hz, 1 H, CH_2-COO), 2.74 (dd, J_1 = 15.1 Hz, J_2 = 6.5 Hz, 1 H, =C-CH_2), 1.37 (s, 9 H, $C(CH_3)_3$), 1.30 (t, J = 7.1 Hz, 3 H, CH_3).

^{13}C-NMR (75 MHz, $CDCl_3$, 298 K) δ / ppm = 169.4, 164.8, 161.8, 158.7, 153.4, 153.3, 151.0, 134.0, 128.0 (2 C), 114.2 (2 C), 110.5, 90.3, 81.6, 61.1, 55.3, 34.9, 33.1, 31.6, 29.5, 28.2, 28.0 (3 C), 14.3.

HRMS (ESI-TOF): *m/z:* $[M+H]^+$ calcd for for $C_{26}H_{33}N_2O_8^+$: 501.2231, found 501.2235.

$[\alpha]_D^{20}$(c = 1, $CHCl_3$, *e.r.* 86:14): +35.3

HPLC (YMC-SA, *n*-hexane/IPA = 7/3, flow = 1.0 mL min^{-1}, T_{Column} = 20 °C, *l* = 269 nm) t_r: 7.1 min (minor), 9.3 min (major).

4-(*tert*-butyl) 1-ethyl (*S*,*Z*)-2-(1,3-dimethyl-2,4-dioxo-5-phenyl-1,2,3,4,5,6-hexahydro-7*H*-pyrano[2,3-*d*]pyrimidin-7-ylidene)succinate (15n)

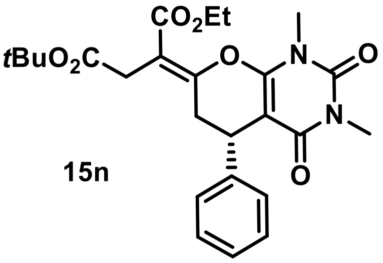

15n

Product **15n** was synthesized according to general procedure **C** on a scale of 0.1 mmol. The compound was obtained as a colorless viscous residue in a yield of 57% (27.0 mg with **ITU1**) and 73% (35.0 mg with **ISeU**) after preparative TLC (heptanes/EtOAc, 1/1, as eluent). The determined *e.r.* were 83:17 (**ITU1**) and 83:17 (**ISeU**).

^{1}H-NMR (300 MHz, $CDCl_3$, 298 K) δ / ppm = 7.30 – 7.16 (m, 5 H, Ar-H), 4.26 (q, J = 7.1 Hz, 2 H, CH_2), 4.28 – 4.25 (m, 1 H, =C-CH), 3.58 (s, 3 H, N-CH_3), 3.31 (s, 3 H, N-CH_3), 3.13 (d, J = 17.2 Hz, 1 H, CH_2-COO), 2.95 (dd, J_1 = 15.1 Hz, J_2 = 2.1 Hz, 1 H, =C-CH_2), 2.88 (d, J = 17.2 Hz, 1 H, CH_2-COO), 2.78 (dd, J_1 = 15.1 Hz, J_2 = 6.5 Hz, 1 H, =C-CH_2), 1.38 (s, 9 H, $C(CH_3)_3$), 1.32 (t, J_1 = 7.1 Hz, 3 H, CH_3).

^{13}C-NMR (75 MHz, $CDCl_3$, 298 K) δ / ppm = 169.3, 164.8, 161.8, 153.5, 153.2, 151.0, 141.9, 128.8 (2 C), 127.3, 127.0 (2 C), 110.6, 89.9, 81.6, 61.2, 34.9, 33.8, 31.4, 29.6, 28.2, 28.1 (3 C), 14.3.

HRMS (ESI-TOF): *m/z:* $[M+H]^+$ calcd for for $C_{25}H_{31}N_2O_7^+$: 471.2126, found 471.2124.

$[\alpha]_D^{20}$(c = 1, $CHCl_3$, *e.r.* 83:17): +30.1

HPLC (YMC-SA, *n*-hexane/IPA = 7/3, flow = 1.0 mL min^{-1}, T_{Column} = 20 °C, *l* = 269 nm) t_r: 6.0 min (minor), 7.0 min (major).

4-(*tert*-butyl) 1-ethyl (*S*,*Z*)-2-(1,3-dimethyl-2,4-dioxo-5-(*p*-tolyl)-1,2,3,4,5,6-hexahydro-7*H*-pyrano[2,3-*d*]pyrimidin-7-ylidene)succinate (15o)

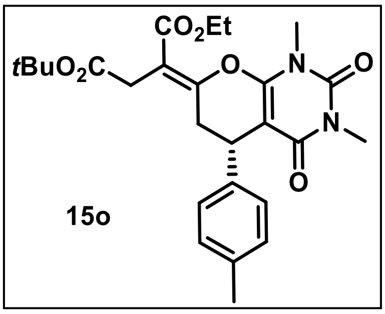

Product **15o** was synthesized according to general procedure **C** on a scale of 0.1 mmol. The compound was obtained as a colorless viscous residue in a yield of 80% (38.9 mg with **ITU1**) and 72% (34.5 mg with **ISeU**) after preparative TLC (heptanes/EtOAc, 1/1, as eluent). The determined *e.r.* were 86:14 (**ITU1**) and 84:16 (**ISeU**).

^{1}H-NMR (300 MHz, $CDCl_3$, 298 K) δ / ppm = 7.09 – 7.01 (m, 4 H, Ar-H), 4.24 (q, J = 7.1 Hz, 2 H, CH_2), 4.22 – 4.20 (m, 1 H, =C-CH), 3.56 (s, 3 H, N-CH_3), 3.29 (s, 3 H, N-CH_3), 3.13 (d, J = 17.2 Hz, 1 H, CH_2-COO), 2.92 (dd, J_1 = 15.1 Hz, J_2 = 2.1 Hz, 1 H, =C-CH_2), 2.89 (d, J = 17.2 Hz, 1 H, CH_2-COO), 2.75 (dd, J_1 = 15.1 Hz, J_2 = 6.5 Hz, 1 H, =C-CH_2), 2.26 (s, 3 H, Ar-CH_3), 1.37 (s, 9 H, $C(CH_3)_3$), 1.30 (t, J = 7.1 Hz, 3 H, CH_3).

^{13}C-NMR (75 MHz, $CDCl_3$, 298 K) δ / ppm = 169.4, 164.9, 161.8, 153.4, 153.3, 151.0, 138.9, 136.8, 129.5 (2 C), 126.8 (2 C), 110.5, 90.2, 81.6, 61.1, 34.9, 33.5, 31.5, 29.6, 28.2, 28.0 (3 C), 21.1, 14.4.

HRMS (ESI-TOF): *m/z:* $[M+H]^+$ calcd for $C_{26}H_{33}N_2O_7^+$: 485.2282, found 485.2283.

$[\alpha]_D^{20}$(c = 1, $CHCl_3$, *e.r.* 86:14): +33.1

HPLC (YMC-SA, *n*-hexane/IPA = 7/3, flow = 1.0 mL min^{-1}, T_{Column} = 20 °C, *l* = 269 nm) t_r: 5.5 min (minor), 6.9 min (major).

4-(*tert*-butyl) 1-ethyl (*S*,*Z*)-2-(5-(4-bromophenyl)-1,3-dimethyl-2,4-dioxo-1,2,3,4,5,6-hexahydro-7*H*-pyrano[2,3-*d*]pyrimidin-7-ylidene)succinate (15p)

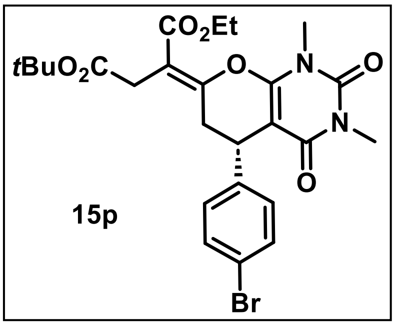

Product **15p** was synthesized according to general procedure **C** on a scale of 0.1 mmol. The compound was obtained as a colorless viscous residue in a yield of 66% (36.2 mg with **ITU1**) and 72% (39.6 mg with **ISeU**) after preparative TLC (heptanes/EtOAc, 1/1, as eluent). The determined *e.r.* were 83:17 (**ITU1**) and 82:18 (**ISeU**).

^{1}H-NMR (300 MHz, $CDCl_3$, 298 K) δ / ppm = 7.41 – 7.35 (m, 2 H, Ar-H), 7.08 – 7.02 (m, 2 H, Ar-H), 4.24 (q, J = 7.1 Hz, 2 H, CH_2), 4.22 – 4.19 (m, 1 H, =C-CH), 3.56 (s, 3 H, CH_3), 3.29 (s, 3 H, CH_3), 3.10 (d, J = 17.2 Hz, 1 H, CH_2-COO), 2.90 (d, J = 17.2 Hz, 1 H, CH_2-COO), 2.89 (dd, J_1 = 15.1 Hz, J_2 = 2.1 Hz, 1 H, =C-CH_2), 2.76 (dd, J_1 = 15.1 Hz, J_2 = 6.5 Hz, 1 H, =C-CH_2), 1.37 (s, 9 H, $C(CH_3)_3$), 1.30 (t, J = 7.1 Hz, 3 H, CH_3).

^{13}C-NMR (75 MHz, $CDCl_3$, 298 K) δ / ppm = 169.1, 164.7, 161.8, 153.7, 152.5, 150.9, 141.0, 131.9 (2 C), 128.8 (2 C), 121.3, 111.1, 89.4, 81.7, 61.2, 34.8, 33.5, 31.2, 29.6, 28.2, 28.0 (3 C), 14.3.

HRMS (ESI-TOF): *m/z:* $[M+H]^+$ calcd for $C_{25}H_{30}BrN_2O_7^+$: 551.1210 (^{81}Br isotope), found 551.1210 (^{81}Br isotope).

$[\boldsymbol{\alpha}]_{\boldsymbol{D}}^{\mathbf{20}}$(c = 1, $CHCl_3$, *e.r.* 83:17): +31.0

HPLC (YMC-SA, *n*-hexane/IPA = 7/3, flow = 1.0 mL min^{-1}, T_{Column} = 20 °C, *l* = 269 nm) t_r: 6.0 min (minor), 8.8 min (major).

7. Product diversification

3-(ethoxycarbonyl)-4,8-dioxo-8-phenyl-6-(trifluoromethyl)octanoic acid (16)

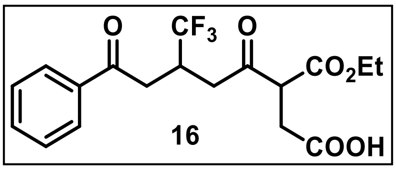

First, the respective *tert*-butyl ester (24.3 mg, 0.057 mmol) was dissolved in 3.0 mL DCM. Afterwards, TFA (0.67 mL, 8.7 mmol, 150 eq.) was added dropwise and stirring at rt. was continued for 4 h. After completion, the reaction mixture was slowly poured over ice and extracted with DCM (3x5 mL). The combined organic layer was dried with Na_2SO_4, filtered, and concentrated. Purification was done using preparative TLC (heptanes/EtOAc/AcOH, 100/100/1). The purified acid was obtained as a roughly 1:1 mixture of diastereomers in the form of an off-white oily residue (18.3 mg, 0.047 mmol, 83%).

^{1}H-NMR (500 MHz, $CDCl_3$, 298 K, mixture of the two diastereomers) δ / ppm = 7.95 – 7.92 (m, 2 H, Ar-H), 7.61 – 7.57 (m, 1 H, Ar-H), 7.49 – 7.45 (m, 2 H, Ar-H), 4.22 – 4.17 (m, 2 H, COO-CH_2), 4.00 – 3.93 (m, 1 H, CO-CH-CO), 3.72 – 3.60 (m, 1 H, CH-CF_3), 3.36 – 2.82 (m, 6 H, Ph-CO-C**H**$_2$-CH(CF_3)-C**H**$_2$-CO-CH(CO_2Et)-C**H**$_2$-COOH), 1.26 (t, *J* = 7.1 Hz, CH_3).

^{13}C-NMR (125 MHz, $CDCl_3$, 298 K, mixture of diastereomers) δ / ppm = 200.2, 196.2, 196.0, 176.7, 176.6, 167.8, 167.5, 136.3, 133.8, 133.7, 128.9 (2 C), 128.3 (2 C), 128.2 (2 C), 127.7 (q, $^{1}J_{C\text{-}F}$ = 279 Hz), 127.6 (q, $^{1}J_{C\text{-}F}$ = 279 Hz), 62.3, 53.7, 53.6, 41.5, 41.3, 36.8, 34.4, 34.3 (q, $^{2}J_{C\text{-}F}$ = 27.6 Hz), (q, $^{2}J_{C\text{-}F}$ = 27.6 Hz), 32.1, 14.1, 14.0.

^{19}F-NMR (282 MHz, $CDCl_3$, 298 K, mixture of the two diastereomers) δ / ppm = -71.6, -71.7.

HRMS (ESI-TOF): *m/z:* $[M+H]^+$ calcd for $C_{18}H_{20}F_3O_6^+$: 389.1206, found: 389.1208.

Ethyl 2-((2*R*,4*R*)-6-phenyl-4-(trifluoromethyl)-3,4-dihydro-2H-pyran-2-yl)acetate (17)

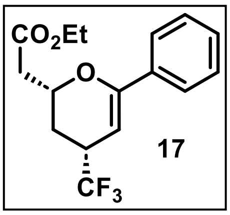

According to literature [8], ethyl (*R,Z*)-2-(6-phenyl-4-(trifluoromethyl)-3,4-dihydro-2*H*-pyran-2-ylidene)acetate **10a** (20.0 mg, 0.064 mmol, *e.r.* 99:1) and Wilkinson's catalyst (6.3 mg, 10 mol%) were dissolved in EtOAc (0.05 M) in a sealed vial. The seal was pierced with a needle and placed in an autoclave. The autoclave was flushed with H_2 two times and finally pressurized to 50 bar (H_2). The mixture was stirred overnight at 60 °C under H_2. The autoclave was cooled to rt. and flushed with air. The mixture was evaporated to dryness and the residue was purified by preparative TLC (heptanes/EtOAc, 2/1) to yield the pure *cis*-product **17** as colorless oily residue (18.1 mg, 0.058, 90%, >95:5 *d.r.*). The determined *e.r.* was 94:6.

^{1}H-NMR (300 MHz, $CDCl_3$, 298 K) δ / ppm = 7.57-7.52 (m, 2 H, Ar-**H**), 7.35-7.30 (m, 3 H, Ar-**H**), 5.26 (bs, 1 H, -C=C**H**), 4.51-4.43 (m, 1 H, -C**H**-O-), 4.23 (q, *J* = 7.1 Hz, 2 H, -C**H**$_2$), 3.38-3.23 (m, 1 H, CF_3-C**H**), 2.87 (dd, J_1 = 15.7 Hz, J_2 = 7.4 Hz, 1 H, -C**H**$_2$-CO_2Et), 2.67 (dd, J_1 = 15.7 Hz, J_2 = 5.8 Hz, 1 H, -C**H**$_2$-CO_2Et), 2.30 (ddt,, J_1 = 13.1 Hz, J_2 = 6.4 Hz, J_3 = 1.6 Hz, 1 H, -C**H**$_2$), 1.81 (dt, J_1 = 13.1 Hz, J_2 = 11.5 Hz, 1 H, -C**H**$_2$), 1.31 (t, *J* = 7.1 Hz, 3 H, -C**H**$_3$).

^{13}**C-NMR** (75 MHz, $CDCl_3$, 298 K) δ / ppm = 170.4, 155.1, 134.6, 129.1, 128.4 (2 C), 126.8 (q, J = 278.7 Hz, 1 C), 125.1 (2 C), 90.5 (q, J = 3.5 Hz, 1 C), 71.8, 61.0, 40.6, 38.0 (q, J = 28.6 Hz, 1 C), 27.3 (q, J = 2.7 Hz, 1 C), 14.4.

^{19}**F-NMR** (282 MHz, $CDCl_3$, 298 K) δ / ppm = -73.4 (3 F, -C**F₃**).

HRMS (ESI-TOF): *m/z:* $[M+H]^+$ calcd for $C_{16}H_{18}F_3O_3^+$: 315.1203, found 315.1204.

$[\alpha]_D^{20}$(c = 1, $CHCl_3$, *e.r.* 94:6): -46.5

HPLC (CHIRALPAK® AD-H, *n*-hexane/IPA = 4/1, flow = 0.5 mL min^{-1}, T_{Column} = 10 °C, *l* = 220 nm) t_r: 14.3 min (minor), 15.7 min (major).

Ethyl 2-((2*R*,4*R*)-4-phenyl-6-(trifluoromethyl)-3,4-dihydro-2H-pyran-2-yl)acetate (18)

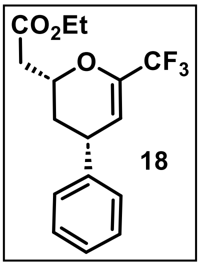

According to literature [8], ethyl (*S,Z*)-2-(4-phenyl-6-(trifluoromethyl)-3,4-dihydro-2*H*-pyran-2-ylidene)acetate **12a** (19.5 mg, 0.062 mmol, *e.r.* 99:1) and Wilkinson's catalyst (5.8 mg, 10 mol%) were dissolved in EtOAc (0.05 M) in a sealed vial. The seal was pierced with a needle and placed in an autoclave. The autoclave was flushed with H_2 two times and finally pressurized to 50 bar (H_2). The mixture was stirred overnight at 60 °C under H_2. The autoclave was cooled to rt. and flushed with air. The mixture was evaporated to dryness and the residue was purified by preparative TLC (heptanes/EtOAc, 2/1) to yield the product **18** as colorless viscous residue (16.4 mg, 0.052 mmol, 84%, >95:5 *d.r.*). The determined *e.r.* was 99:1.

^{1}**H-NMR** (300 MHz, $CDCl_3$, 298 K) δ / ppm = 7.36-7.25 (m, 3 H, Ar-**H**), 7.24-7.18 (m, 2 H, Ar-**H**), 5.53 (bs, 1 H, -C=C**H**), 4.55 (m, 1 H, -C**H**-O-), 4.18 (q, J = 7.1 Hz, 2 H, -C**H₂**), 3.72-3.64 (m, 1 H, -C**H**), 2.81 (dd, J_1 = 15.8 Hz, J_2 = 6.6 Hz, 1 H, -C**H₂**-CO_2Et), 2.58 (dd, J_1 = 15.8 Hz, J_2 = 6.7 Hz, 1 H, -C**H₂**-CO_2Et), 2.30 (ddt,, J_1 = 13.7 Hz, J_2 = 6.4 Hz, J_3 = 1.7 Hz, 1 H, -C**H₂**), 1.70 (dt, J_1 = 13.7 Hz, J_2 = 11.3 Hz, 1 H, -C**H₂**), 1.27 (t, J = 7.1 Hz, 3 H, -C**H₃**).

^{13}**C-NMR** (75 MHz, $CDCl_3$, 298 K) δ / ppm = 170.2, 143.6 (q, J = 35.3 Hz, 1 C), 142.9, 129.0 (2 C), 127.2 (3 C), 119.8 (q, J = 272.3 Hz, 1 C), 106.6 (q, J = 3.9 Hz, 1 C), 74.1, 61.0, 40.2, 37.9, 37.0, 14.3.

^{19}**F-NMR** (282 MHz, $CDCl_3$, 298 K) δ / ppm = -72.6 (3 F, -C**F₃**).

HRMS (ESI-TOF): *m/z:* $[M+H]^+$ calcd for $C_{16}H_{18}F_3O_3^+$: 315.1203, found 315.1205.

$[\alpha]_D^{20}$(c = 1, $CHCl_3$, *e.r.* 99:1): +67.7

HPLC (YMC-SA, *n*-hexane/IPA = 4/1, flow = 1.0 mL min^{-1}, T_{Column} = 10 °C, *l* = 220 nm) t_r: 10.4 min (major), 14.8 min (minor).

(*Z*)-2-(5-(4-(*tert*-butyl)phenyl)-1,3-dimethyl-2,4-dioxo-1,2,3,4,5,6-hexahydro-7*H*-pyrano[2,3-*d*]pyrimidin-7-ylidene)acetic acid (19)

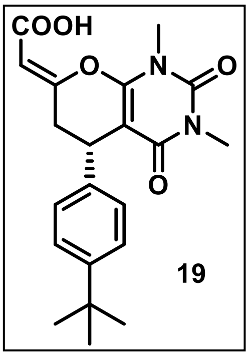

According to literature [9], the respective *tert*-butyl ester **14n** (25.8 mg, 0.059 mmol, *e.r.* 75:25) was dissolved in 3.0 mL DCM. Afterwards, TFA (0.1 mL, 1.3 mmol, 22 eq.) was added and stirring at rt. was continued for 4 h. After completion, the reaction mixture was slowly poured over ice and extracted with DCM (3x5 mL). The combined organic layer was dried with Na_2SO_4, filtered and concentrated to give the pure acid **19** in quantitative yield as transparent oily residue (22.5 mg, 0.059 mmol, >99%, *e.r.* 75:25).

^{1}H-NMR (300 MHz, $CDCl_3$, 298 K) δ / ppm = 8.25 (bs, 1 H, -COO**H**), 7.30 (d, J = 8.3 Hz, 2 H, Ar-**H**), 7.08 (d, J = 8.3 Hz, 2 H, Ar-**H**), 5.21 (d, J = 1.2 Hz, 1 H, -C**H**), 4.25 (d, J = 6.9 Hz, 1 H, -C**H**), 3.58 (s, 3 H, -NC**H₃**), 3.32 (s, 3 H, -NC**H₃**), 2.97 (ddd, J_1 = 15.0 Hz, J_2 = 6.9 Hz, J_3 = 1.6 Hz, 1 H, -C**H₂**), 2.70 (dd, J_1 = 15.0 Hz, J_2 = 1.2 Hz, 1 H, -C**H₂**), 1.27 (s, 9 H, -C(C**H**$_3$)$_3$).

^{13}C-NMR (75 MHz, $CDCl_3$, 298 K) δ / ppm = 168.5, 162.0, 159.6, 153.0, 150.9, 150.3, 138.3, 126.7 (2 C), 125.9 (2 C), 102.4, 90.8, 34.6, 32.7, 31.4 (3 C), 29.8, 29.6, 28.4.

HRMS (ESI-TOF): *m/z:* [M+H]$^+$ calcd for $C_{21}H_{25}N_2O_5^+$: 385.1758, found 385.1759.

$[\alpha]_D^{20}$(c = 1, $CHCl_3$): -15.8

HPLC (YMC-SB, *n*-hexane/IPA = 1/1, flow = 1.0 mL min^{-1}, T_{Column} = 10 °C, l = 220 nm) t_r: 8.5 min (major), 17.5 min (minor).

(*Z*)-3-(1,3-dimethyl-2,4-dioxo-5-phenyl-1,2,3,4,5,6-hexahydro-7*H*-pyrano[2,3-*d*]pyrimidin-7-ylidene)-4-ethoxy-4-oxobutanoic acid (20)

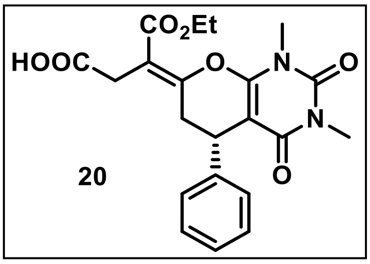

First, the respective *tert*-butyl ester (27.0 mg, 0.057 mmol) was dissolved in 3.0 mL DCM. Afterwards, TFA (0.72 mL, 9.3 mmol, 150 eq.) was added dropwise and stirring at rt. was continued for 4 h. After completion, the reaction mixture was slowly poured over ice and extracted with DCM (3x5 mL). The combined organic layer was dried with Na_2SO_4, filtered, and concentrated. Purification was done using preparative TLC (heptanes/EtOAc/AcOH, 100/100/1). The purified acid was obtained as an off-white oily residue (16.1 mg, 0.039 mmol, 68%). The determined *e.r.* was 84:16.

^{1}H-NMR (300 MHz, $CDCl_3$, 298 K) δ / ppm = 7.27 – 7.12 (m, 5 H, Ar-H), 5.90 (bs, 1 H, COOH), 4.28 – 4.24 (m, 1 H, =C-CH), 4.24 (q, J = 7.1 Hz, 2 H, CH_2), 3.55 (s, 3 H, N-CH_3), 3.30 (s, 3 H, N-CH_3), 3.15 (d, J = 17.2 Hz, 1 H, CH_2-COO), 2.94 (dd, J_1 = 15.1 Hz, J_2 = 1.7 Hz, 1 H, =C-CH_2), 2.90 (d, J = 17.2 Hz, 1 H, CH_2-COO), 2.75 (dd, J_1 = 15.1 Hz, J_2 = 6.5 Hz, 1 H, =C-CH_2), 1.29 (t, J = 7.1 Hz, 3 H, CH_3).

^{13}C-NMR (125 MHz, $CDCl_3$, 298 K) δ / ppm = 175.2, 164.7, 161.9, 154.3, 153.5, 151.0, 141.5, 128.9 (2 C), 127.4, 127.0 (2 C), 109.5, 90.0, 61.5, 33.8, 33.5, 31.8, 29.6, 28.3, 14.3.

HRMS (ESI-TOF): *m/z:* $[M+H]^+$ calcd for $C_{21}H_{23}N_2O_7^+$: 415.1500, found: 415.1504.

$[\alpha]_D^{20}$(c = 1, $CHCl_3$, *e.r.* 84:16): +37.1

HPLC (YMC-SB, *n*-hexane/IPA = 1/1, flow = 1.0 mL min^{-1}, T_{Column} = 20 °C, *l* = 290 nm) t_r: 9.0 min (major), 16.8 min (minor).

(*S,Z*)-7-(2-hydroxyethylidene)-1,3-dimethyl-5-phenyl-1,5,6,7-tetrahydro-2*H*-pyrano[2,3-*d*]pyrimidine-2,4(3H)-dione (21)

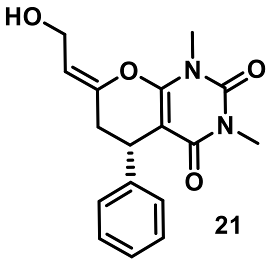

21

In analogy to literature [10], a flame-dried reaction flask was charged with ethyl (*S,Z*)-2-(1,3-dimethyl-2,4-dioxo-5-phenyl-1,2,3,4,5,6-hexahydro-7*H*-pyrano[2,3-*d*]pyrimidin-7-ylidene)acetate **14b** (35.3 mg, 0.099 mmol, *e.r.* 78:22), $LiAlH_4$ (17.3 mg, 0.793 mmol, 8 eq.) and anhydrous Et_2O (5 mL). The reaction mixture was stirred at rt. overnight. The reaction was carefully quenched by the addition of 20 mL 4 M HCl and the phases were separated. Then the aqueous phase was extracted with DCM (3x), the collected organic phases were dried with Na_2SO_4, filtered and evaporated to dryness. The residue was purified by preparative TLC (heptanes/EtOAc, 1/1) to give the desired alcohol **21** in moderate yield as a colorless oily residue(53%, 16.5 mg, 0.052 mmol, *e.r.* 78:22).

^{1}H-NMR (300 MHz, $CDCl_3$, 298 K) δ / ppm = 7.31-7.20 (m, 3 H, Ar-**H**), 7.19-7.12 (m, 2 H, Ar-**H**), 4.89 (td, J_1 = 6.9 Hz, J_2 = 1.9 Hz, 1 H, -C**H₂**), 4.41-4.28 (m, 2 H, HO-C**H₂**), 4.21 (dd, J_1 = 6.3 Hz, J_2 = 1.9 Hz, 1 H, -C**H**), 3.50 (s, 3 H, -NC**H₃**), 3.30 (s, 3 H, -NC**H₃**), 2.87-2.79 (m, 1 H, -C**H₂**), 2.53 (dd, J_1 = 14.4 Hz, J_2 = 1.9 Hz, 1 H, -C**H₂**).

^{13}C-NMR (75 MHz, $CDCl_3$, 298 K) δ / ppm = 162.0, 153.9, 151.1, 146.2, 142.6, 128.6 (2 C), 127.3 (2 C), 127.1, 112.0, 90.1, 56.2, 34.3, 33.8, 29.0, 28.3.

HRMS (ESI-TOF): *m/z:* $[M+H]^+$ calcd for $C_{17}H_{19}N_2O_4^+$: 315.1339, found 315.1340.

$[\alpha]_D^{20}$(c = 1, $CHCl_3$, *e.r.* 78:22): -28.7

HPLC (YMC-SB, *n*-hexane/IPA = 1/1, flow = 1.0 mL min^{-1}, T_{Column} = 10 °C, *l* = 220 nm) t_r: 17.1 min (major), 19.1 min (minor).

(*Z*)-7-(1,4-dihydroxybutan-2-ylidene)-1,3-dimethyl-5-phenyl-1,5,6,7-tetrahydro-2*H*-pyrano[2,3-*d*]pyrimidine-2,4(3*H*)-dione (22)

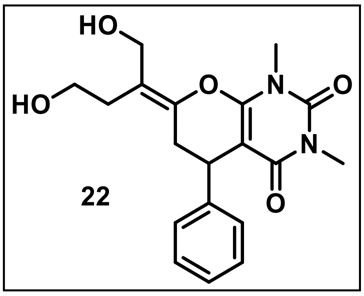

A flame-dried reaction flask was charged with diethyl (*S*,*Z*)-2-(1,3-dimethyl-2,4-dioxo-5-phenyl-1,2,3,4,5,6-hexahydro-7*H*-pyrano[2,3-*d*]pyrimidin-7-ylidene)succinate (22.2 mg, 0.050 mmol), $LiAlH_4$ (16.7 mg, 0.766 mmol, 15 eq.) and anhydrous Et_2O (25 mL). The reaction mixture was stirred at rt. overnight. The reaction was carefully quenched by the addition of 20 mL 4 M HCl and the phases were separated. Then the aqueous phase was extracted with DCM (3x), the collected organic phases were dried with Na_2SO_4, filtered, and evaporated to dryness. The residue was purified by preparative TLC (pure EtOAc) to give the desired alcohol as viscous colorless residue in moderate yield (35%, 6.2 mg, 0.017 mmol).

^{1}H-NMR (500 MHz, $CDCl_3$, 298 K) δ / ppm = 7.30 – 7.26 (m, 2 H, Ar-H), 7.23 – 7.19 (m, 1 H, Ar-H), 7.15 – 7.12 (m, 2 H, Ar-H), 4.43 (d, J = 12.1 Hz, 1 H, =C-C**H**$_2$-OH), 4.28 – 4.25 (m, 1 H, CH-Ar), 4.27 (d, J = 12.1 Hz, 1 H, =C-C**H**$_2$-OH), 3.52 (s, 3 H, N-CH_3), 3.40 – 3.35 (m, 1 H, CH_2-C**H**$_2$-OH), 3.31 (s, 3 H, N-CH_3), 2.84 – 2.79 (m, 1 H, CH_2-C**H**$_2$-OH), 2.79 (dd, J_1 = 14.3 Hz, J_2 = 2.1 Hz, 1 H, Ar-CH-C**H**$_2$), 2.65 (dd, J_1 = 14.3 Hz, J_2 = 5.8 Hz, 1 H, Ar-CH-C**H**$_2$), 2.18 – 2.14 (m, 2 H, =C-C**H**$_2$-CH_2-OH).

^{13}C-NMR (125 MHz, $CDCl_3$, 298 K) δ / ppm = 160.2, 154.5, 151.1, 143.2, 142.8, 128.6 (2 C), 127.4 (2 C), 127.3, 121.3, 89.4, 61.9, 59.6, 34.8, 31.5, 30.6, 29.1, 28.2.

HRMS (ESI-TOF): *m/z:* $[M+H]^+$ calcd for $C_{19}H_{23}N_2O_5^+$: 359.1601, found: 359.1598.

Ethyl 2-((5*S*,7*R*)-1,3-dimethyl-2,4-dioxo-5-phenyl-1,3,4,5,6,7-hexahydro-2*H*-pyrano[2,3-*d*]pyrimidin-7-yl)acetate (23)

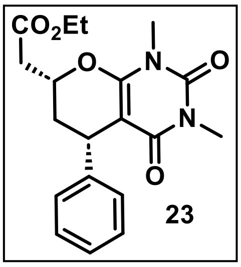

According to literature [8], ethyl (*S,Z*)-2-(1,3-dimethyl-2,4-dioxo-5-phenyl-1,2,3,4,5,6-hexahydro-7*H*-pyrano[2,3-*d*]pyrimidin-7-ylidene) acetate **14b** (35.9 mg, 0.101 mmol, *e.r.* 78:22) and Wilkinson's catalyst (9.3 mg, 10 mol%) were dissolved in EtOAc (0.05 M) in a sealed vial. The seal was pierced with a needle and placed in an autoclave. The autoclave was flushed with H_2 two times and finally filled to 50 bar (H_2). The mixture was stirred overnight at 60 °C under H_2. The autoclave was cooled to rt. and flushed with air. The mixture was evaporated to dryness and the residue was purified by preparative TLC (heptanes/EtOAc, 1/1) to yield the *cis*-product **23** as off-white oily residue (36.0 mg, 0.100 mmol, 99%, >95:5 *d.r.*). The determined *e.r.* was 78:22.

^{1}H-NMR (300 MHz, $CDCl_3$, 298 K) δ / ppm = 7.19-7.14 (m, 2 H, Ar-**H**), 7.09-7.04 (m, 3 H, Ar-**H**), 4.62-4.53 (m, 1 H, -OC**H**), 4.07 (q, J = 7.1 Hz, 2 H, -C**H$_2$**), 3.88 (dd, J_1 = 10.8 Hz, J_2 = 7.1 Hz, 1 H, -C**H**), 3.24 (s, 3 H, -NC**H$_3$**), 3.11 (s, 3 H, -NC**H$_3$**), 2.66 (dd, J_1 = 16.1 Hz, J_2 = 8.3 Hz, 1 H, -C**H$_2$**-CO_2Et), 2.48 (dd, J_1 = 16.1 Hz, J_2 = 4.9 Hz, 1 H, -C**H$_2$**-CO_2Et), 2.28 (ddd, J_1 = 14.3 Hz, J_2 = 7.1 Hz, J_3 = 2.0 Hz, 1 H, -C**H$_2$**), 1.71 (dt, J_1 = 14.3 Hz, J_2 = 10.8 Hz, 1 H, -C**H$_2$**), 1.16 (t, J = 7.1 Hz, 3 H, -C**H$_3$**).

^{13}C-NMR (75 MHz, $CDCl_3$, 298 K) δ / ppm = 169.5, 162.0, 157.1, 151.2, 144.0, 128.7 (2 C), 126.6 (3 C), 90.5, 76.2, 61.2, 39.8, 38.5, 37.1, 28.7, 28.0, 14.3.

HRMS (ESI-TOF): *m/z:* $[M+H]^+$ calcd for $C_{19}H_{23}N_2O_5^+$: 359.1601, found 359.1600.

$[\alpha]_D^{20}$(c = 1, $CHCl_3$, *e.r.* 78:22): -28.7

HPLC (YMC-SB, *n*-hexane/IPA = 1/1, flow = 1.0 mL min^{-1}, T_{Column} = 10 °C, *l* = 220 nm) t_r: 21.0 min (major), 41.8 min (minor).

8. Crystallographic structure of 10a

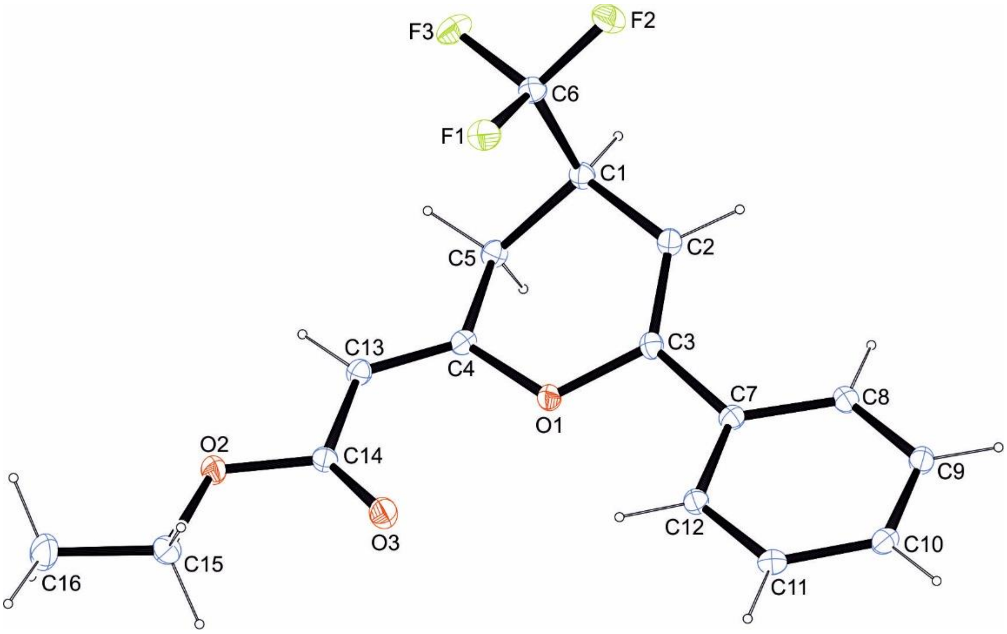

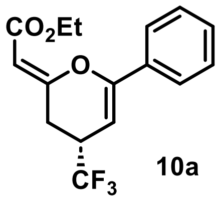

10a

Experimental details:

The X-ray intensity data of **10a** were measured on a 'XtaLAB Synergy R, HyPix-Arc 150' system equipped with a mirror monochromator and a Cu Kα 'Rotating-anode X-ray tube' (λ = 1.54184 Å). The frames were integrated with the CrysAlis^{Pro} software package [11]. Data were corrected for absorption effects using the Multi-Scan method (SCALE3 ABSPACK) integrated in CrysAlis^{Pro}. The structure was solved with SHELXT and refined using the SHELXLE Software Package [12]. All C-bound hydrogen atoms have been calculated in ideal geometry riding on their parent atoms. The figures have been drawn at the 25% ellipsoid probability level [13].

Crystallographic data have been deposited with the Cambridge Crystallographic Data Centre, CCDC, 12 Union Road, Cambridge CB21EZ, UK. Copies of the data can be obtained free of charge on quoting the depository numbers CCDC- 2324831. (https://www.ccdc.cam.ac.uk/structures/).

Table 1: Crystallographic data of **10a.**

net formula	$C_{16}H_{15}F_3O_3$
M_r/g mol^{-1}	312.28
crystal size/mm	0.060 × 0.040 × 0.030
T/K	100(2)
radiation	CuKα
diffractometer	'XtaLAB Synergy R, HyPix-Arc 150'
crystal system	orthorhombic
space group	'P 21 21 2'
a/Å	29.7873(2)
b/Å	9.53680(10)
c/Å	5.04440(10)
α/°	90
β/°	90
γ/°	90
V/Å^3	1432.99(3)
Z	4
calc. density/g cm^{-3}	1.447
μ/mm^{-1}	1.076
absorption correction	multi-scan
transmission factor range	0.81509–1.00000
refls. measured	50454
R_{int}	0.0294
mean σ(I)/I	0.0106
θ range	5.508–74.480
observed refls.	2846
x, y (weighting scheme)	0.0327, 0.2854
hydrogen refinement	constr
Flack parameter	0.02(3)
refls in refinement	2936
parameters	201
restraints	0
$R(F_{obs})$	0.0230
$R_w(F^2)$	0.0607
S	1.037
shift/error$_{max}$	0.001
max electron density/e Å^{-3}	0.167
min electron density/e Å^{-3}	−0.159

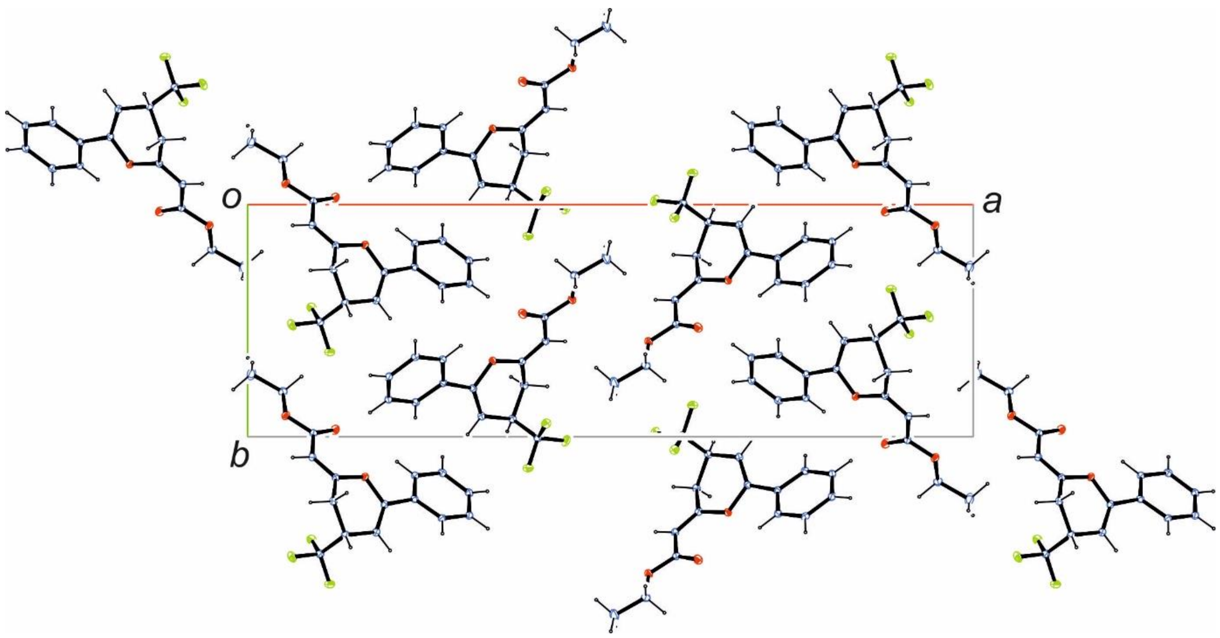
o
a
b

10. References

[1] C. M. Young, A. Elmi, D. J. Pascoe, R. K. Morris, C. McLaughlin, A. M. Woods, A. B. Frost, A. Houpliere, K. B. Ling, T. K. Smith, A. M. Z. Slawin, P. H. Willoughby, S. Cockroft, A. D. Smith, *Angew. Chem., Int. Ed*. **2020**, *59*, 3705−3710.

[2] L. S. Vogl, P. Mayer, R. Robiette, M. Waser, *Angew. Chem. Int. Ed.* **2024**, *63*, e202315345.

[3] M. G. Sankar, M. Garcia-Castro, C. Golz, C. Strohmann, K. Kumar, *Angew. Chem. Int. Ed.*, **2016**, *55*, 9709-9713

[4] H. Liu, Y. Liu, C. Yuan, G.-P. Wang, S.-F. Zhu, Y. Wu, B. Wang, Z. Sun, Y. Xiao, Q.-L. Zhou, H. Guo, *Org. Lett.* **2016**, *18*, 1302-1305.

[5] J. L. Serrano, P. F. Soeiro, M. A. Reis, R. E. F. Boto, S. Silvestre, P. Almeida, *Mol. Divers*. **2020**, *24*, 155-166.

[6] D. G. Stark, L. C. Morrill, P.-P. Yeh, A. M. Z. Slawin, T. J. C. O'Riordan, A. D. Smith, *Angew. Chem. Int. Ed.* **2013**, *52*, 11642-11646.

[7] A. V. Popov, V. A. Kobelevskaya, N. I. Borodin, S. V. Zinchenko, *J. Fluor. Chem.* **2023**, *267*, 110108.

[8] K. Kasten, D. B. Cordes, A. M. Z. Slawin, A. D. Smith, *Eur. J. Org. Chem.* **2016**, *21*, 3619-3624

[9] S. Petersen, J. M. Alonso, A. Specht, P. Duodu, M. Goeldner, A. del Campo, *Angew. Chem. Int. Ed.* **2018**, *47*, 3192-3195

[10] M. N. Iskander, P. R. Andrews, *J. Chem. Edu.* **1985**, *62*, 913

[11] Rigaku Oxford Diffraction **2023**. CrysAlis^{Pro}

[12] C. B. Huebschle, G. M. Sheldrick, B. Dittrich, *J. Appl. Cryst*. **2011**, *44*, 1281-1284

[13] L. J. Farrugia, *J. Appl. Cryst*. **2012**, *45*, 849-854

11. Appendix: NMR spectra and HPLC chromatograms

11.1 NMR spectra of the novel starting materials - barbiturates

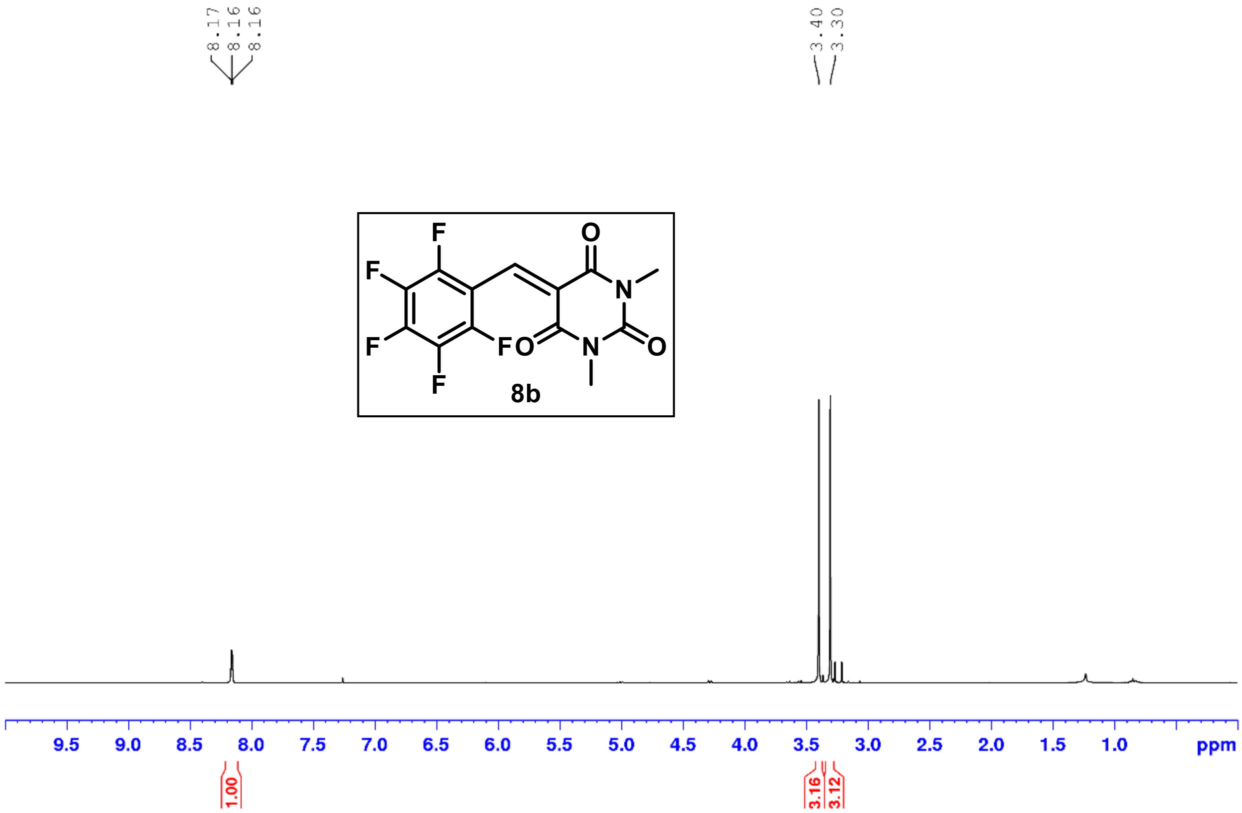

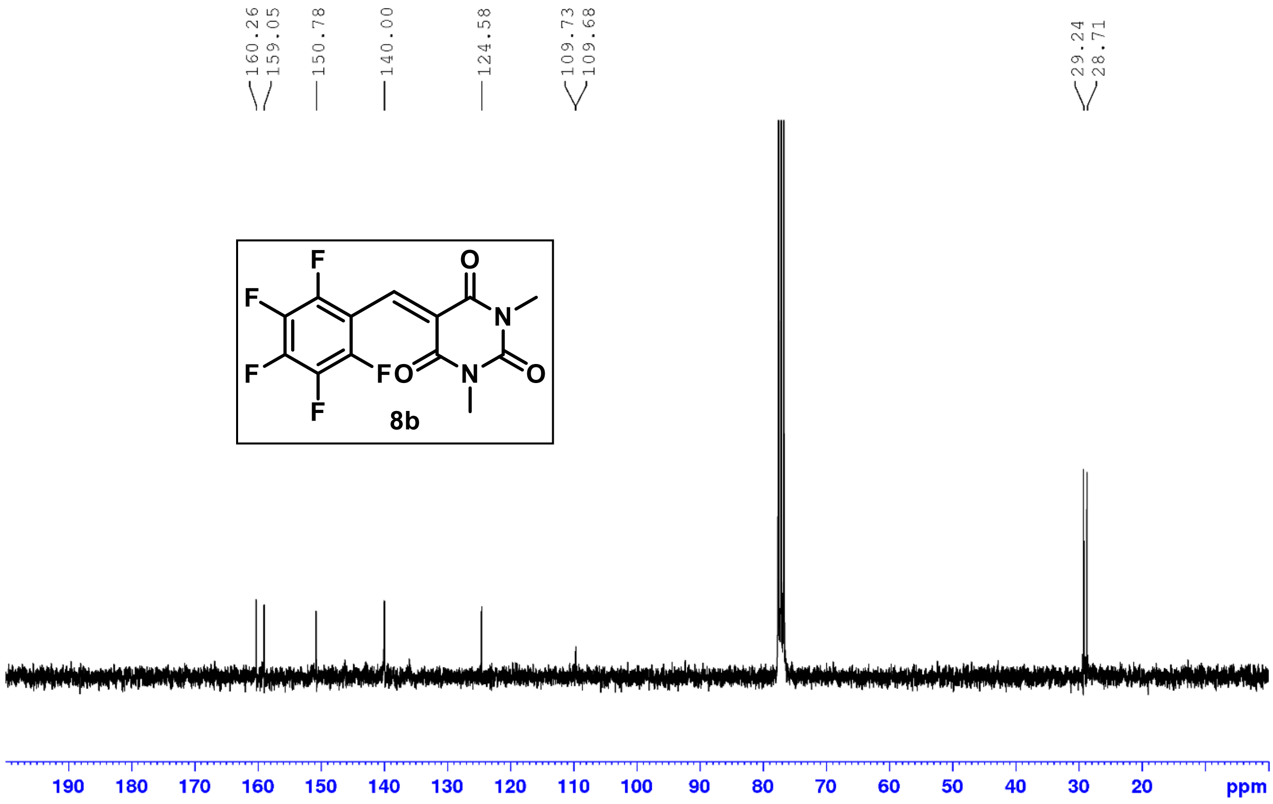

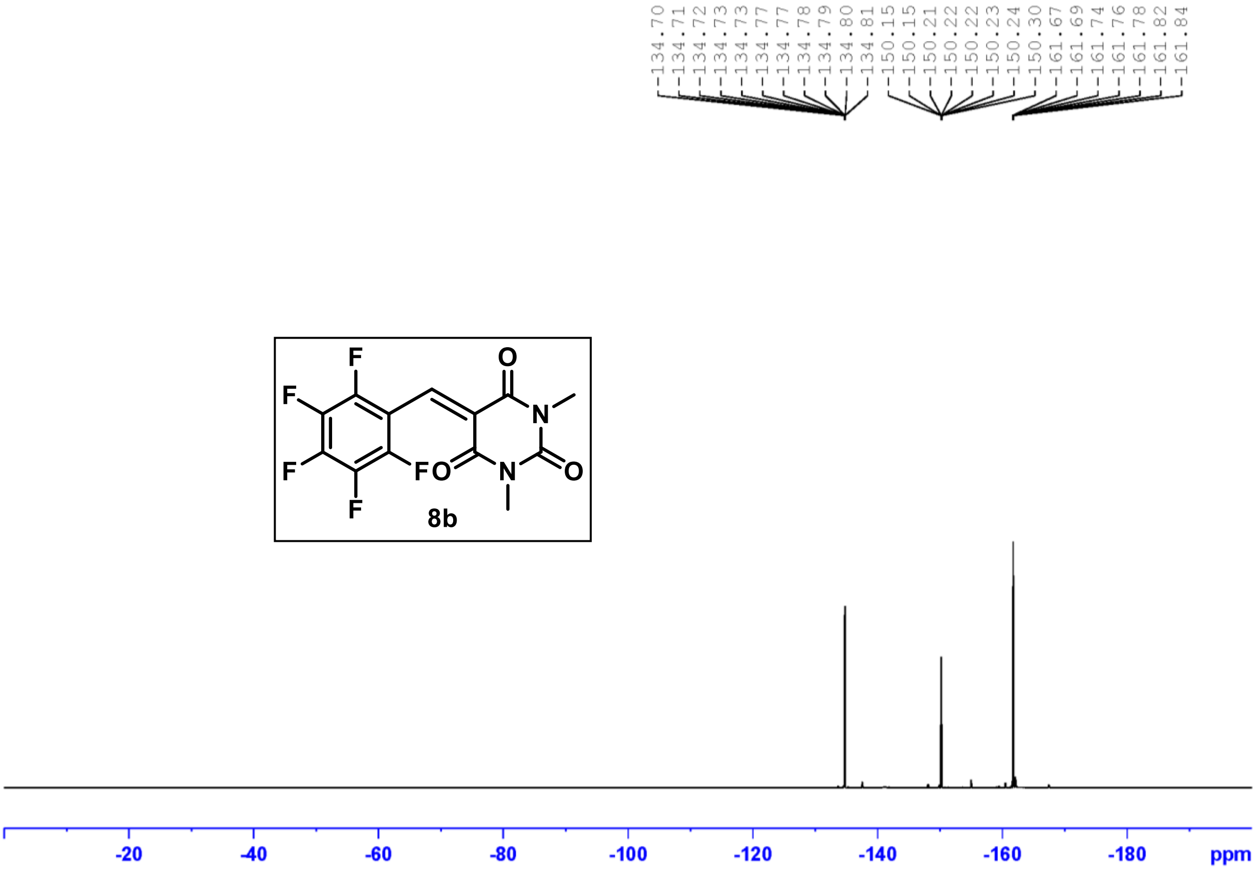
134.70
134.71
134.72
134.73
134.73
134.77
134.77
134.78
134.79
134.80
134.81
150.15
150.15
150.21
150.22
150.22
150.23
150.24
150.30
161.67
161.69
161.74
161.76
161.78
161.82
161.84
F
F
F
F
F
O
N
O
N
O
8b
-20
-40
-60
-80
-100
-120
-140
-160
-180
ppm

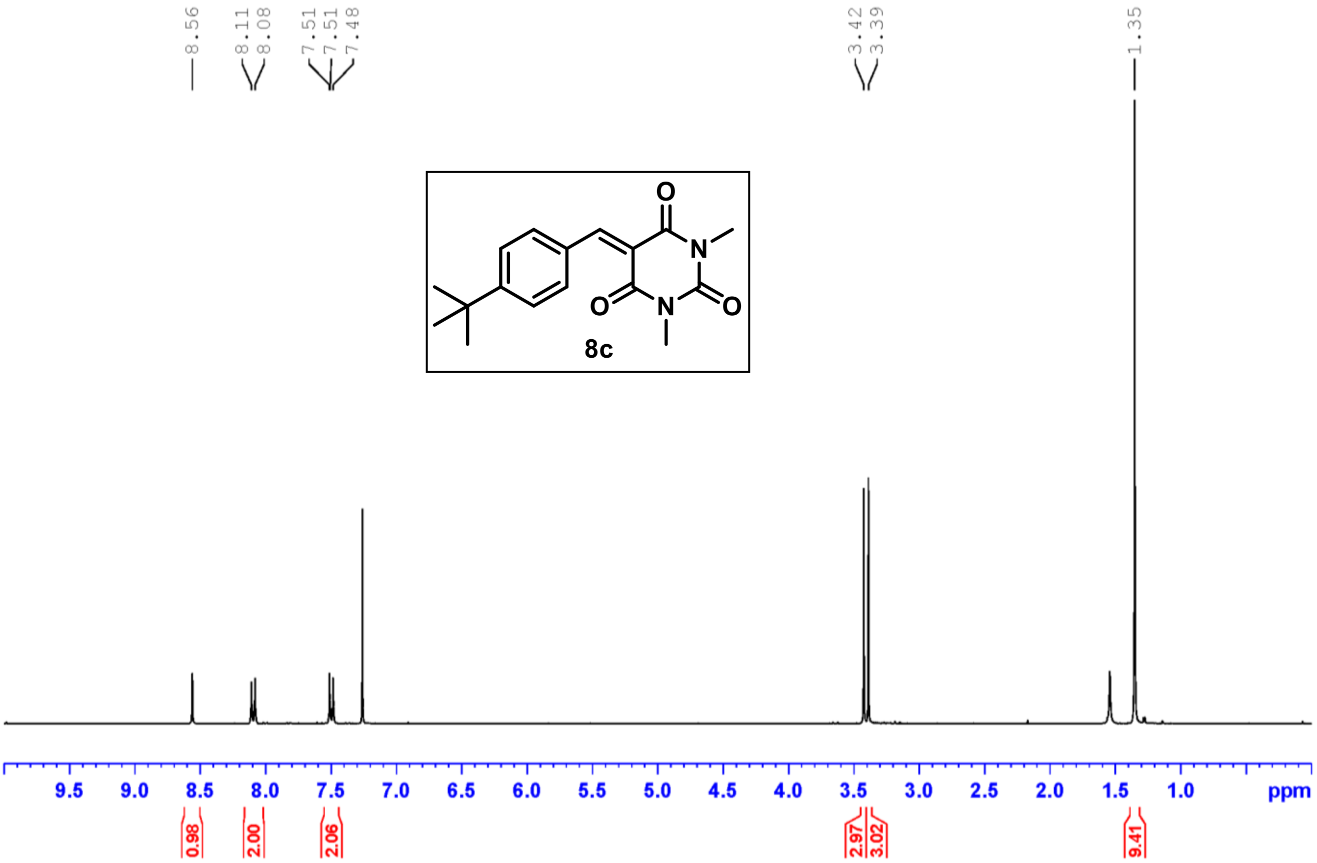
8.56
8.11
8.08
7.51
7.51
7.48
3.42
3.39
1.35
O
N
O
N
O
8c
9.5
9.0
8.5
8.0
7.5
7.0
6.5
6.0
5.5
5.0
4.5
4.0
3.5
3.0
2.5
2.0
1.5
1.0
ppm
0.98
2.00
2.06
2.97
3.02
9.41

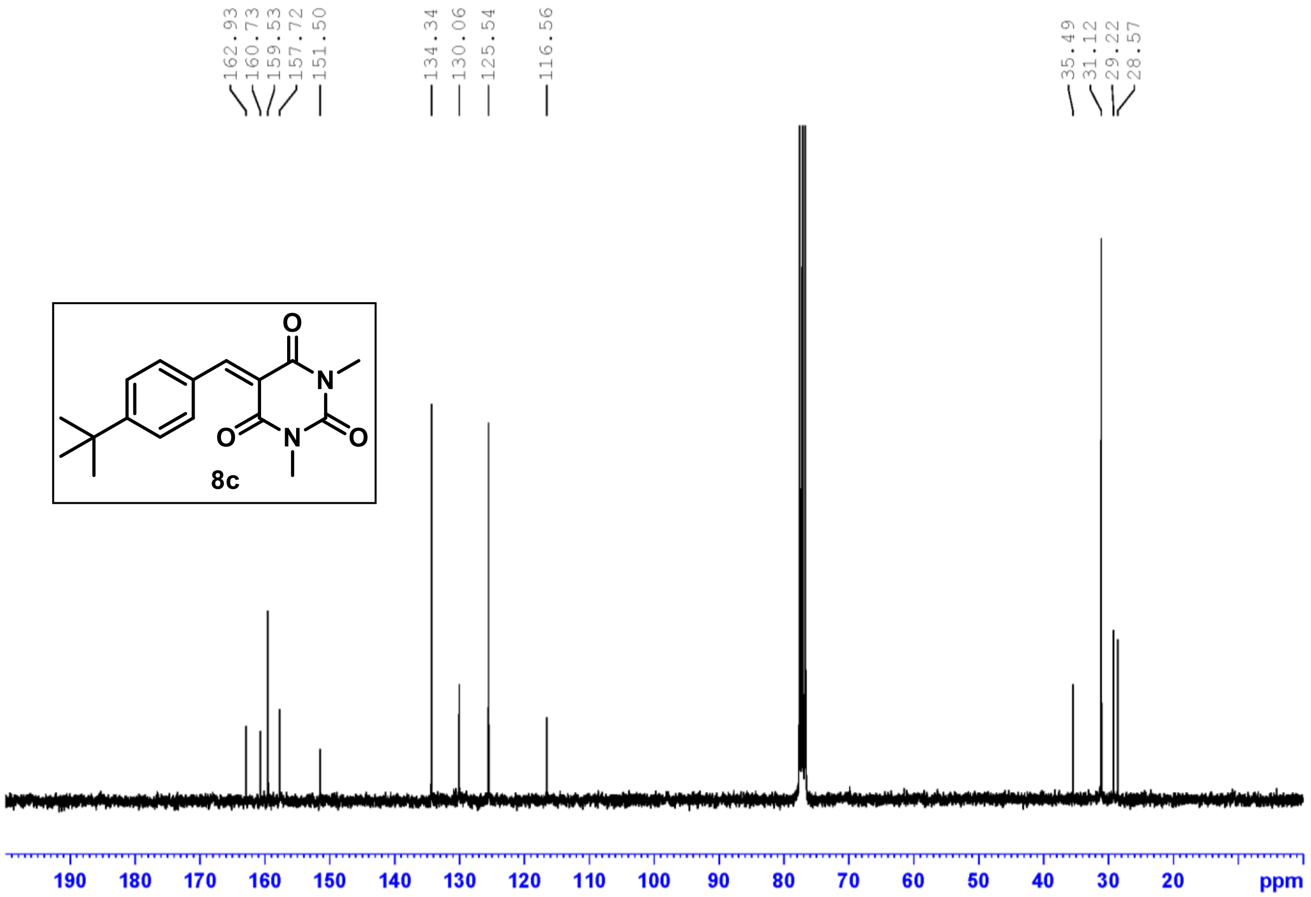
162.93
160.73
159.53
157.72
151.50
134.34
130.06
125.54
116.56
35.49
31.12
29.22
28.57
8c
190 180 170 160 150 140 130 120 110 100 90 80 70 60 50 40 30 20 ppm

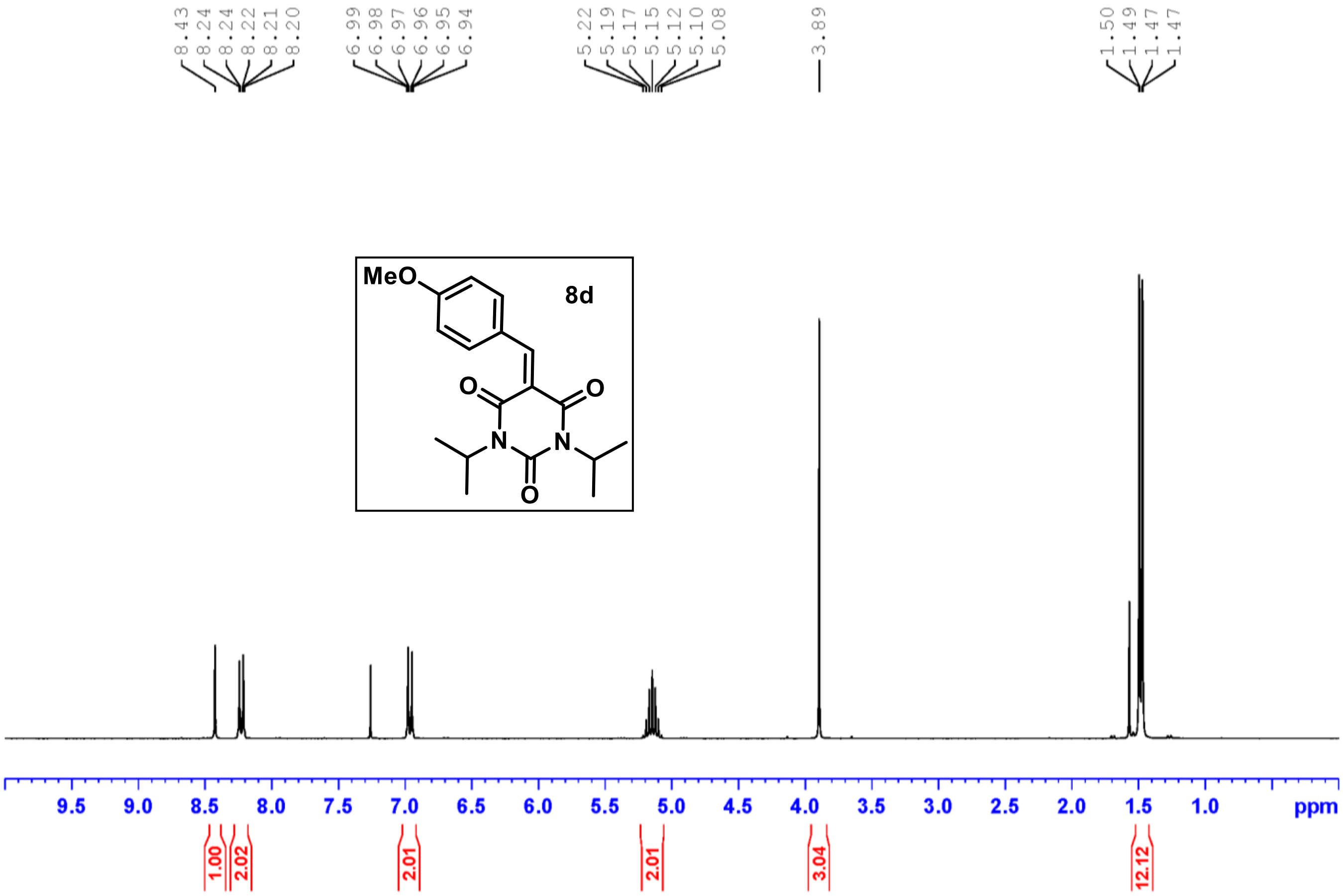
8.43
8.24
8.24
8.22
8.21
8.20
6.99
6.98
6.97
6.96
6.95
6.94
5.22
5.19
5.17
5.15
5.12
5.10
5.08
3.89
1.50
1.49
1.47
1.47
MeO
8d
9.5 9.0 8.5 8.0 7.5 7.0 6.5 6.0 5.5 5.0 4.5 4.0 3.5 3.0 2.5 2.0 1.5 1.0 ppm
1.00
2.02
2.01
2.01
3.04
12.12

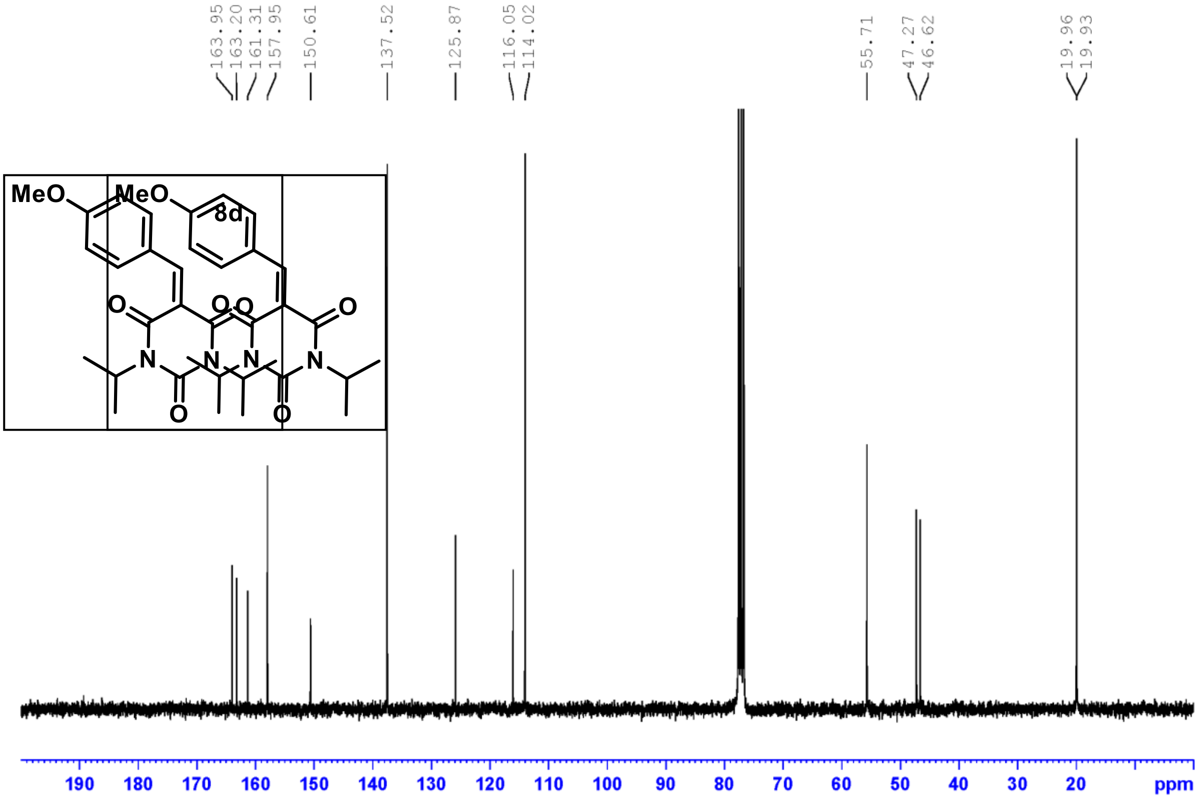
163.95
163.20
161.31
157.95
150.61
137.52
125.87
116.05
114.02
55.71
47.27
46.62
19.96
19.93
MeO
8d
O
N
190 180 170 160 150 140 130 120 110 100 90 80 70 60 50 40 30 20 ppm

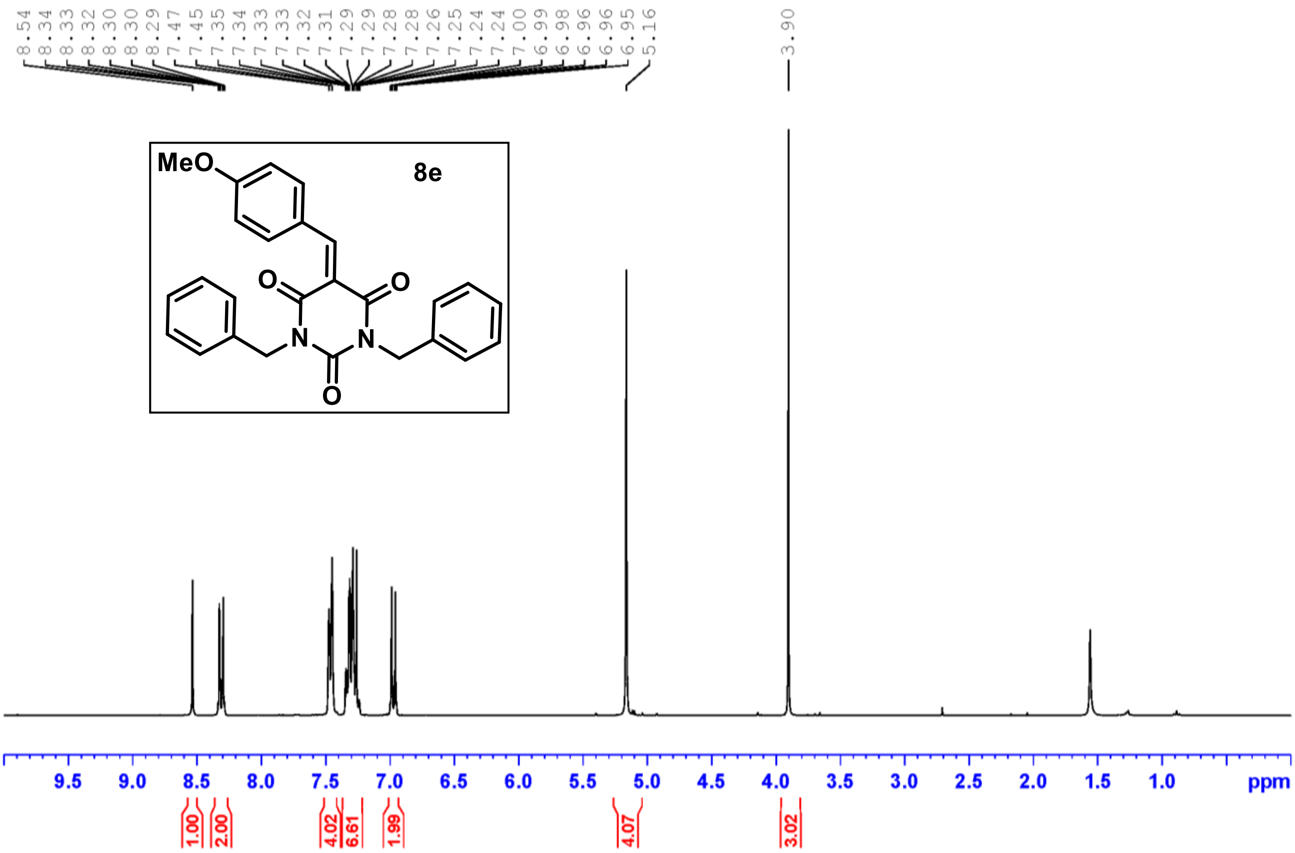
8.54
8.34
8.33
8.32
8.30
8.30
8.29
7.47
7.45
7.35
7.34
7.33
7.33
7.32
7.31
7.29
7.29
7.28
7.28
7.26
7.25
7.24
7.24
7.00
6.99
6.98
6.96
6.96
6.95
5.16
3.90
MeO
8e
O
N
9.5 9.0 8.5 8.0 7.5 7.0 6.5 6.0 5.5 5.0 4.5 4.0 3.5 3.0 2.5 2.0 1.5 1.0 ppm
1.00
2.00
4.02
6.61
1.99
4.07
3.02

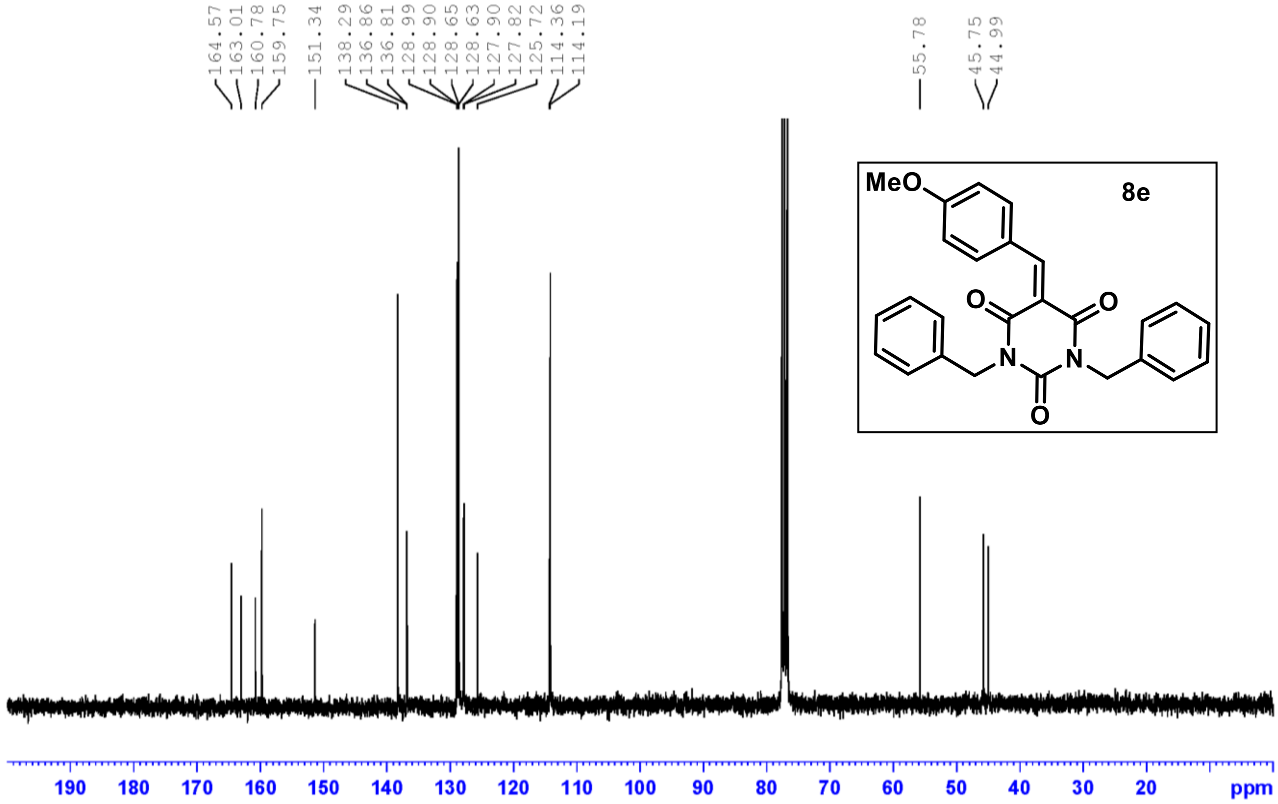
164.57
163.01
160.78
159.75
151.34
138.29
136.86
136.81
128.99
128.90
128.65
128.63
127.90
127.82
125.72
114.36
114.19
55.78
45.75
44.99
MeO
8e
O
N
O
190 180 170 160 150 140 130 120 110 100 90 80 70 60 50 40 30 20 ppm

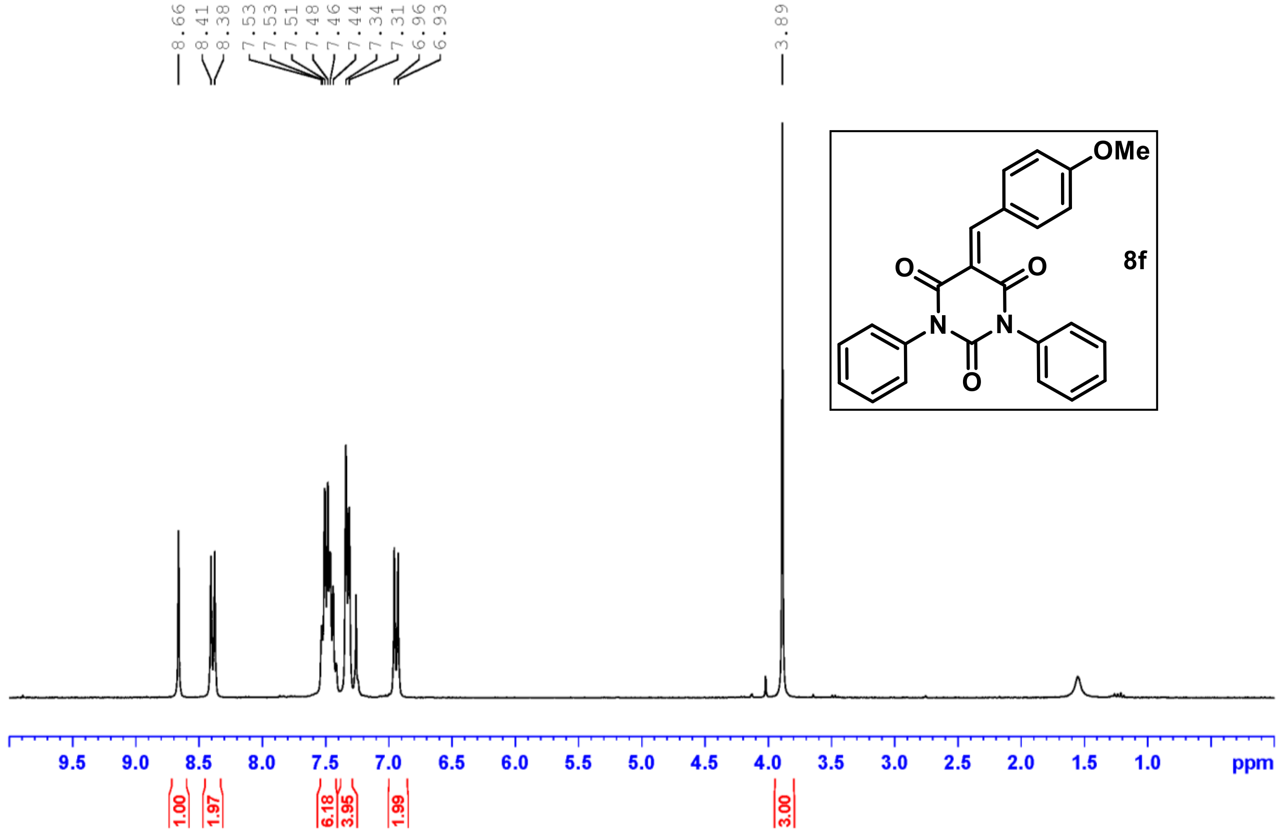
8.66
8.41
8.38
7.53
7.53
7.51
7.48
7.46
7.44
7.34
7.31
6.96
6.93
3.89
OMe
8f
O
N
O
9.5 9.0 8.5 8.0 7.5 7.0 6.5 6.0 5.5 5.0 4.5 4.0 3.5 3.0 2.5 2.0 1.5 1.0 ppm
1.00
1.97
6.18
3.95
1.99
3.00

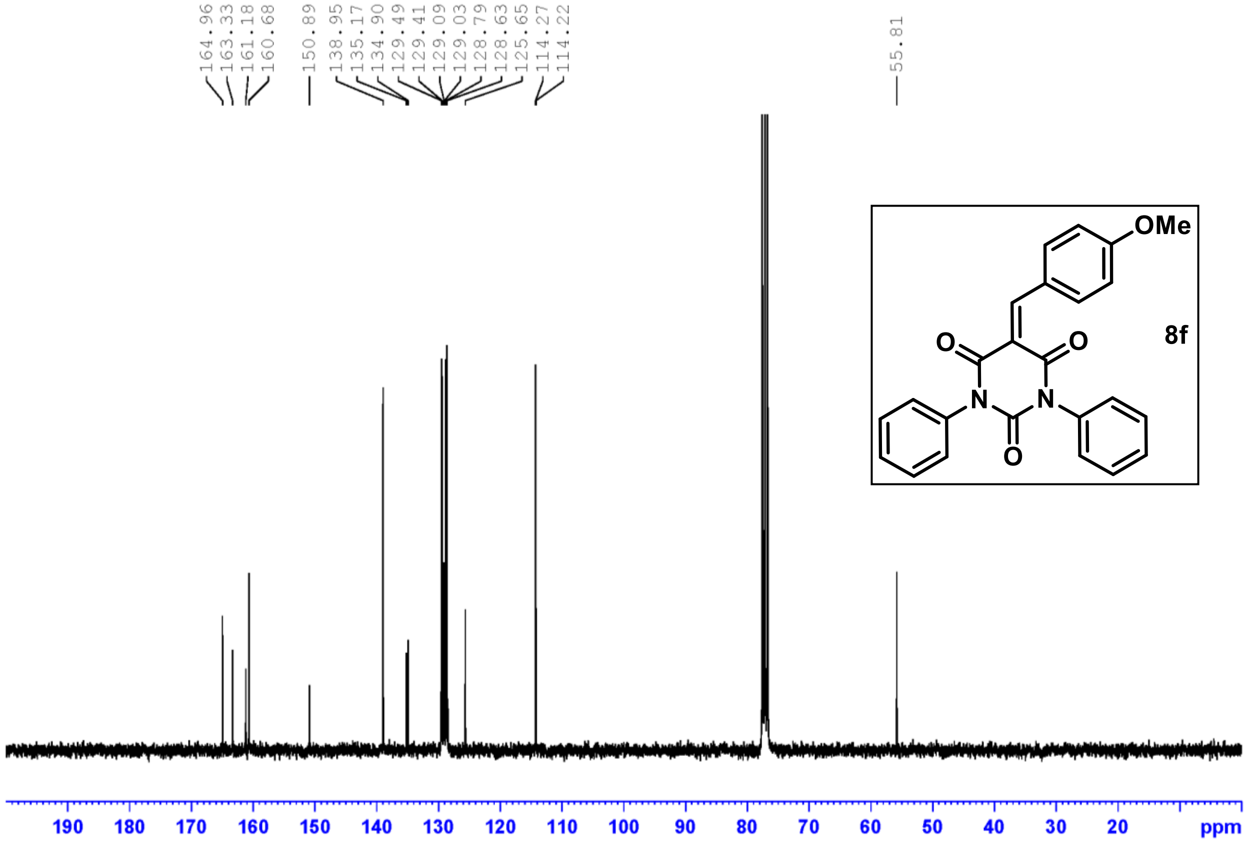
164.96
163.33
161.18
160.68
150.89
138.95
135.17
134.90
129.49
129.41
129.09
129.03
128.79
128.63
125.65
114.27
114.22
55.81
OMe
O
O
N
N
O
8f
190
180
170
160
150
140
130
120
110
100
90
80
70
60
50
40
30
20
ppm

11.2 NMR spectra of the 4+2 cyclization products

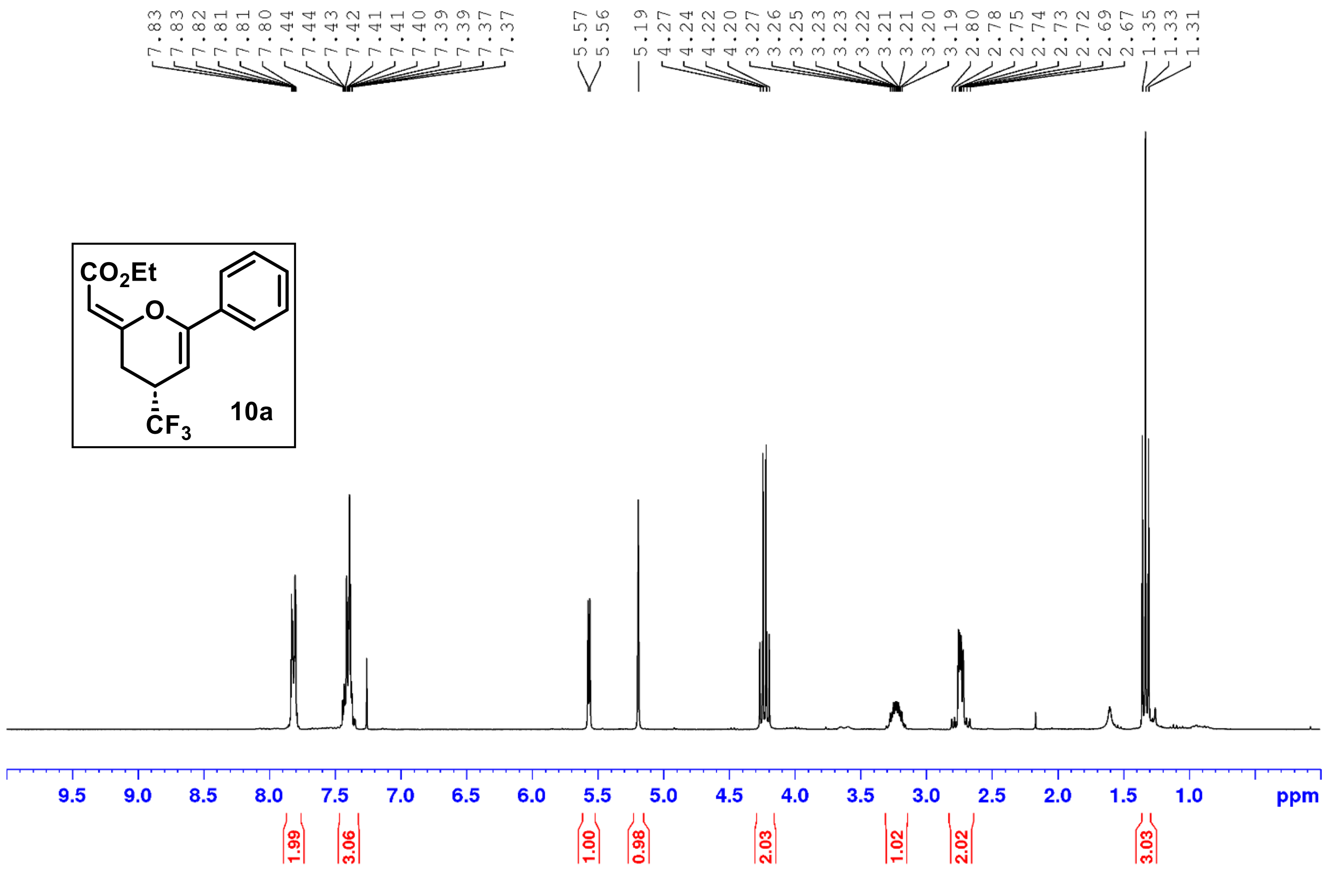

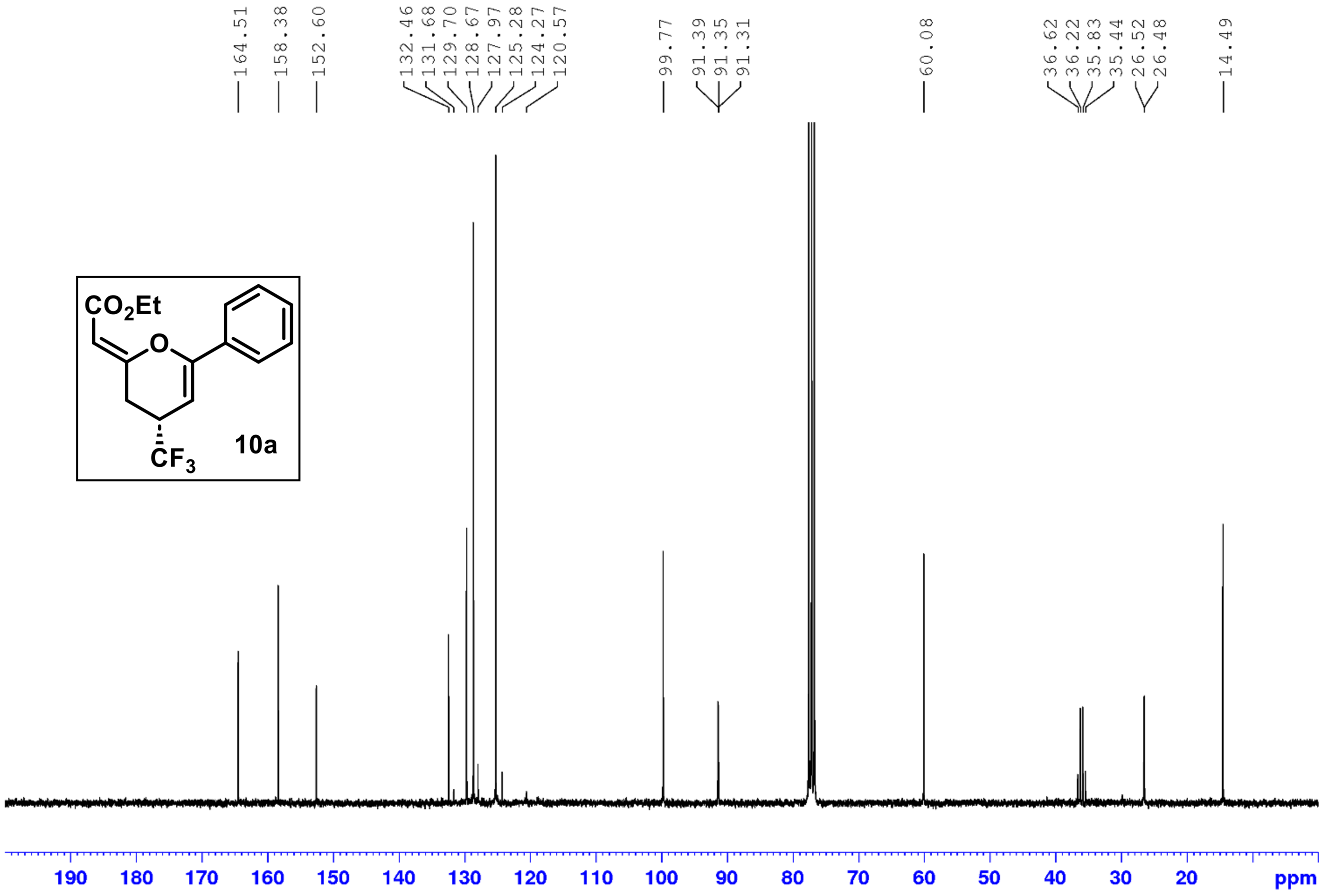

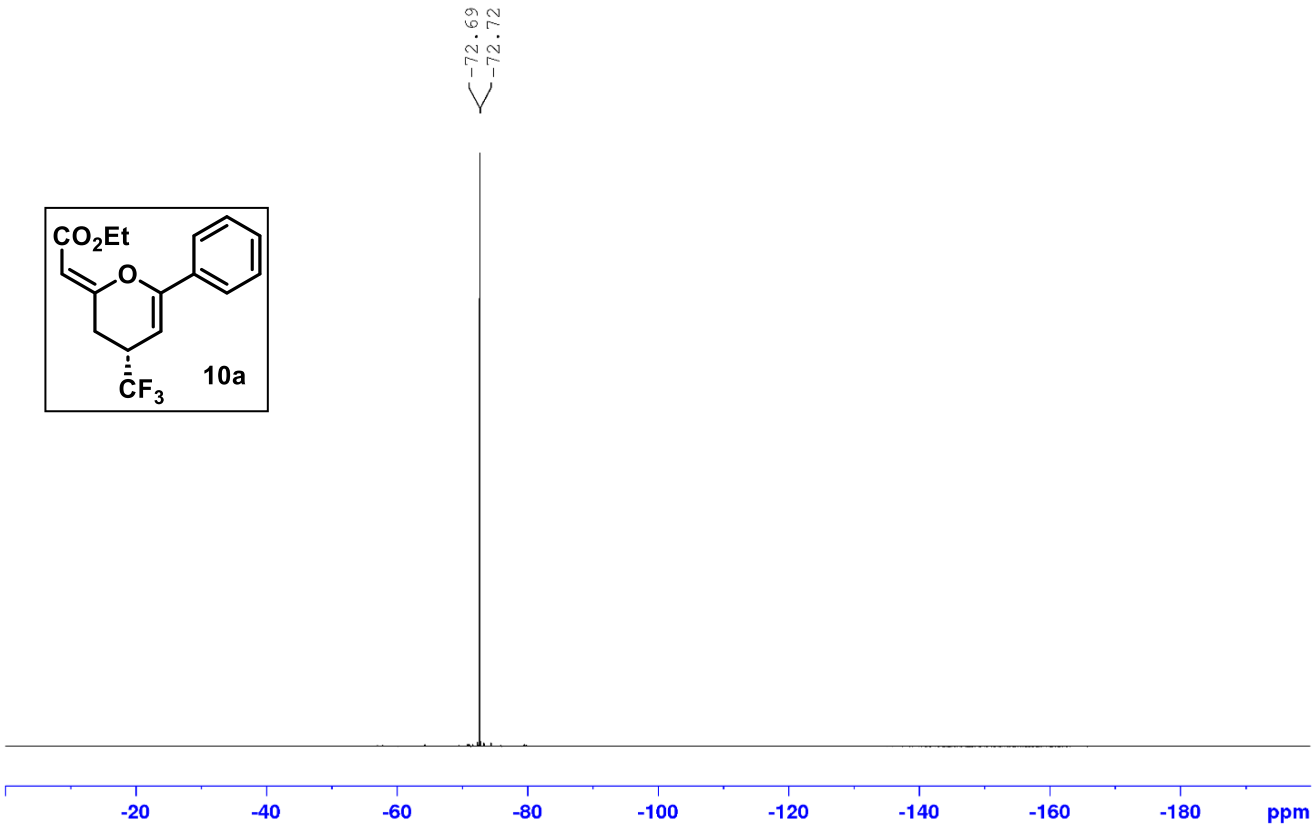

-72.69
-72.72
CO2Et
O
CF3
10a
-20
-40
-60
-80
-100
-120
-140
-160
-180
ppm

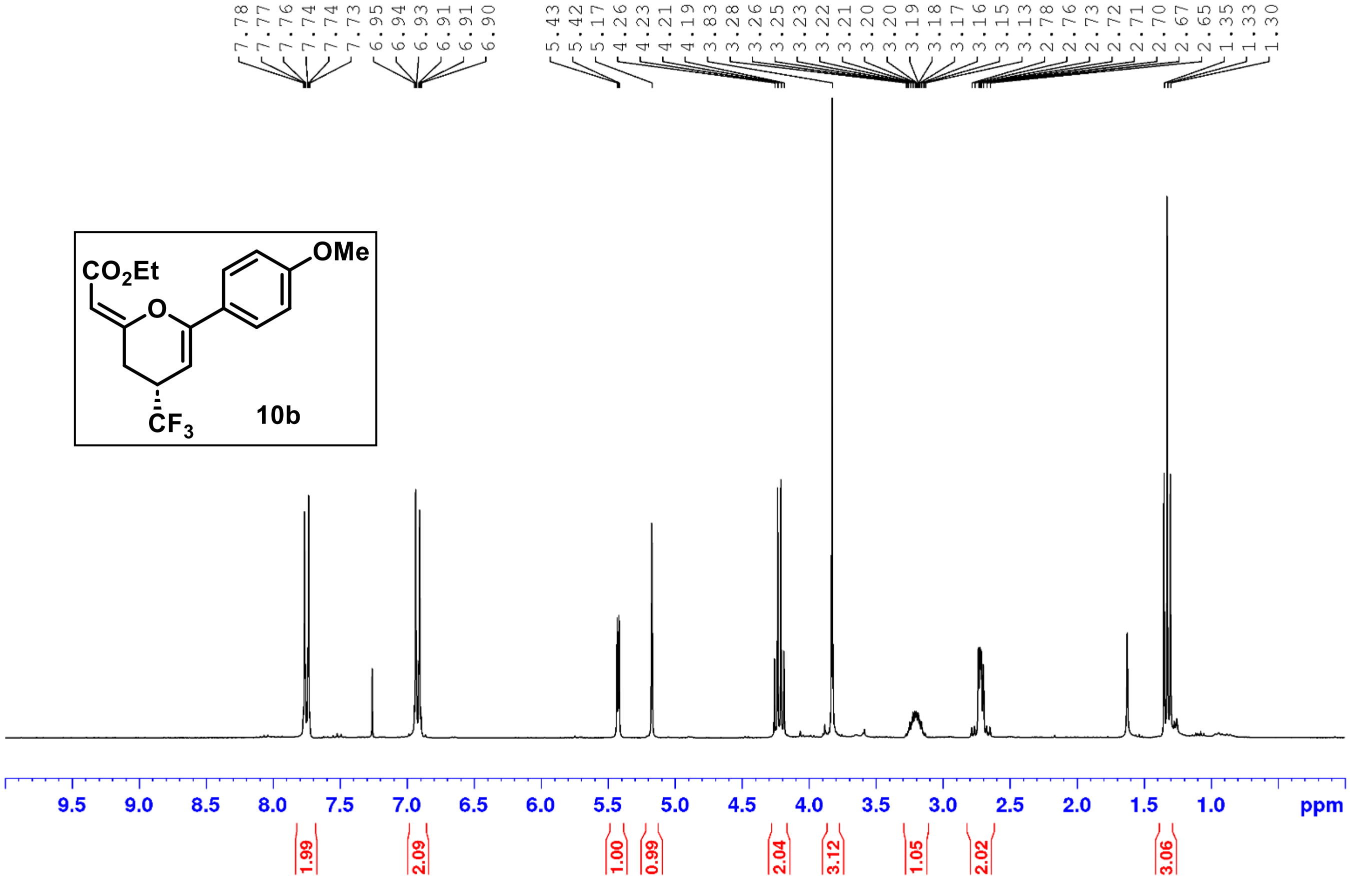

7.78 7.77 7.76 7.74 7.74 7.73 6.95 6.94 6.93 6.91 6.91 6.90
5.43 5.42 5.17 4.26 4.23 4.21 4.19 3.83 3.28 3.26 3.25 3.23 3.22 3.21 3.20 3.19 3.18 3.17 3.16 3.15 3.13 2.78 2.76 2.73 2.72 2.71 2.70 2.67 2.65 1.35 1.33 1.30
OMe
CO2Et
O
CF3
10b
9.5
9.0
8.5
8.0
7.5
7.0
6.5
6.0
5.5
5.0
4.5
4.0
3.5
3.0
2.5
2.0
1.5
1.0
ppm
1.99
2.09
1.00
0.99
2.04
3.12
1.05
2.02
3.06

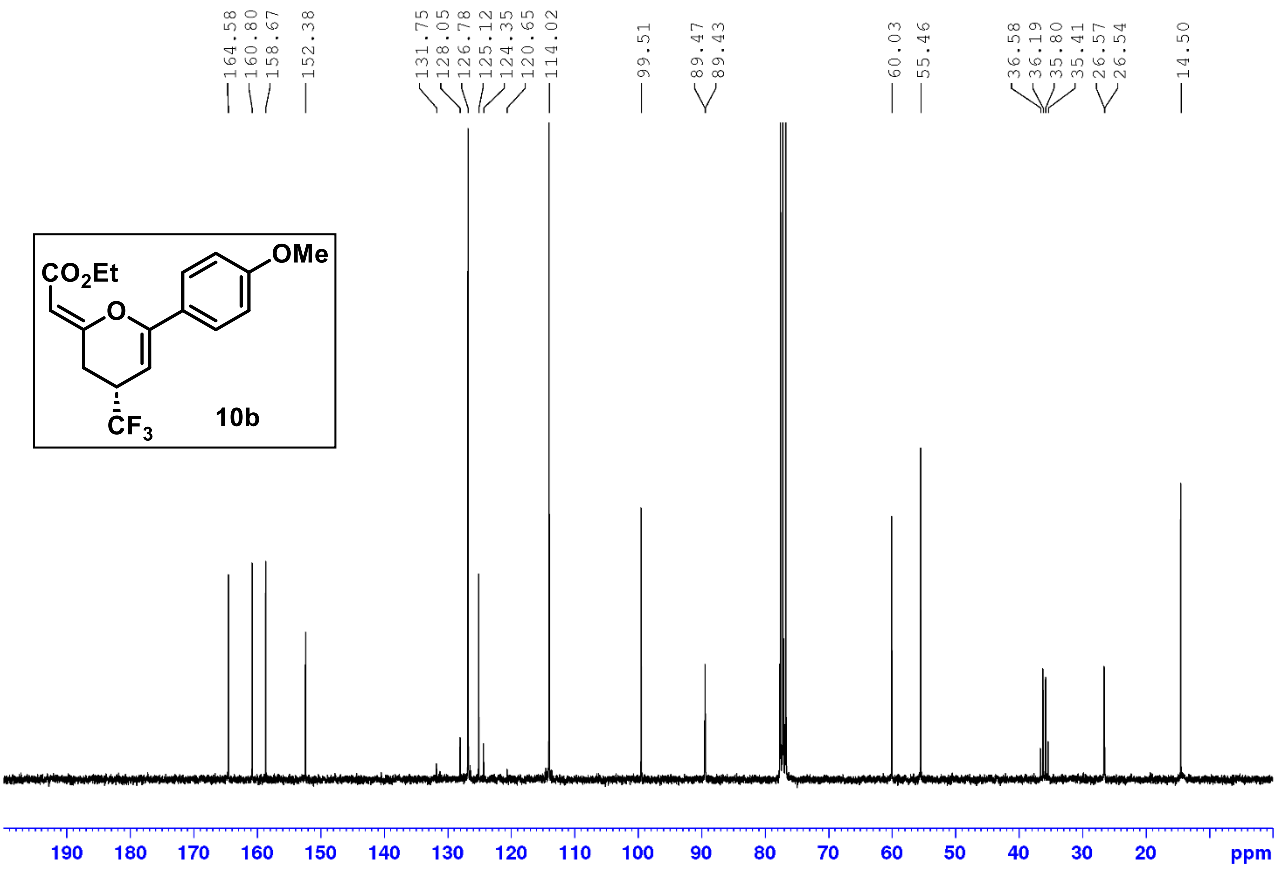
164.58
160.80
158.67
152.38
131.75
128.05
126.78
125.12
124.35
120.65
114.02
99.51
89.47
89.43
60.03
55.46
36.58
36.19
35.80
35.41
26.57
26.54
14.50
CO_2Et
OMe
O
CF_3
10b
190 180 170 160 150 140 130 120 110 100 90 80 70 60 50 40 30 20 ppm

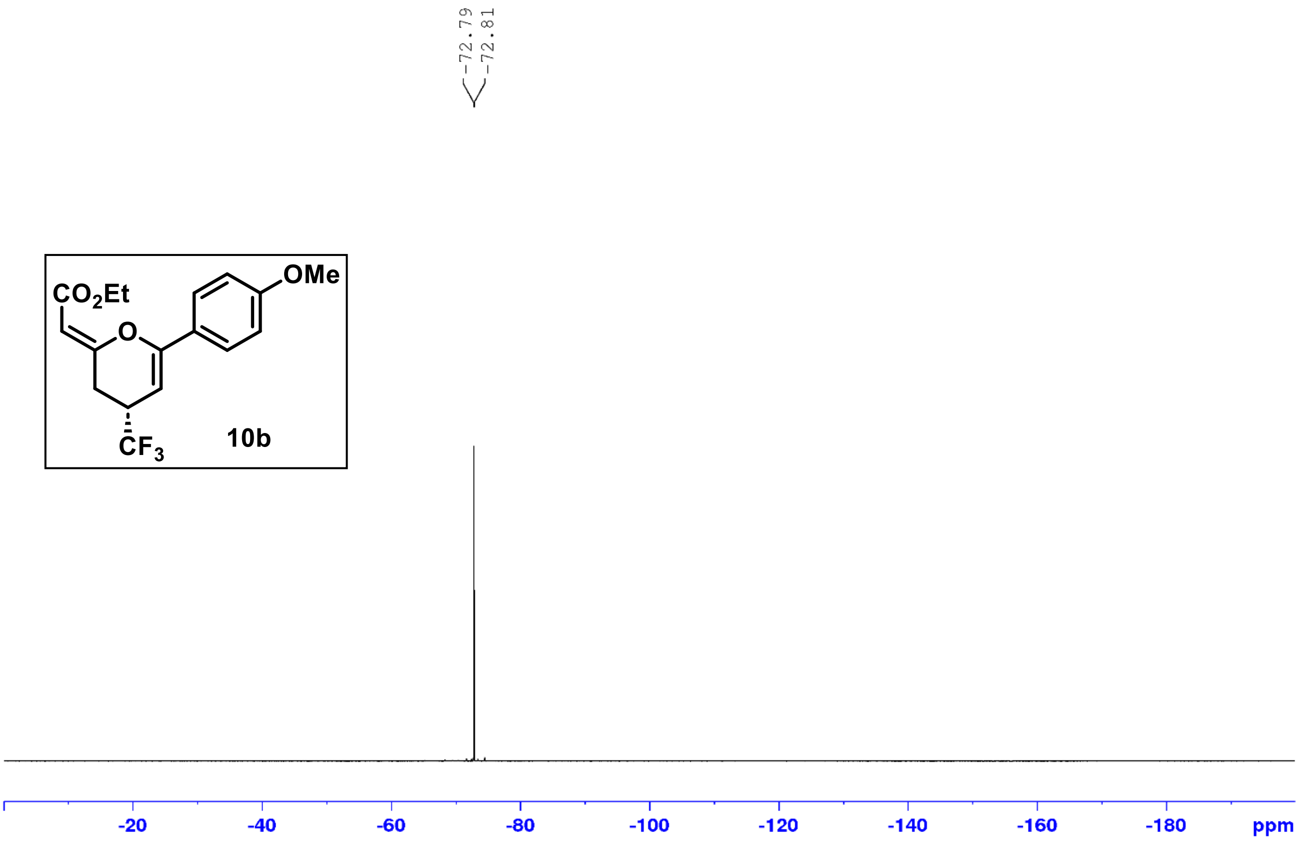
-72.79
-72.81
CO_2Et
OMe
O
CF_3
10b
-20 -40 -60 -80 -100 -120 -140 -160 -180 ppm

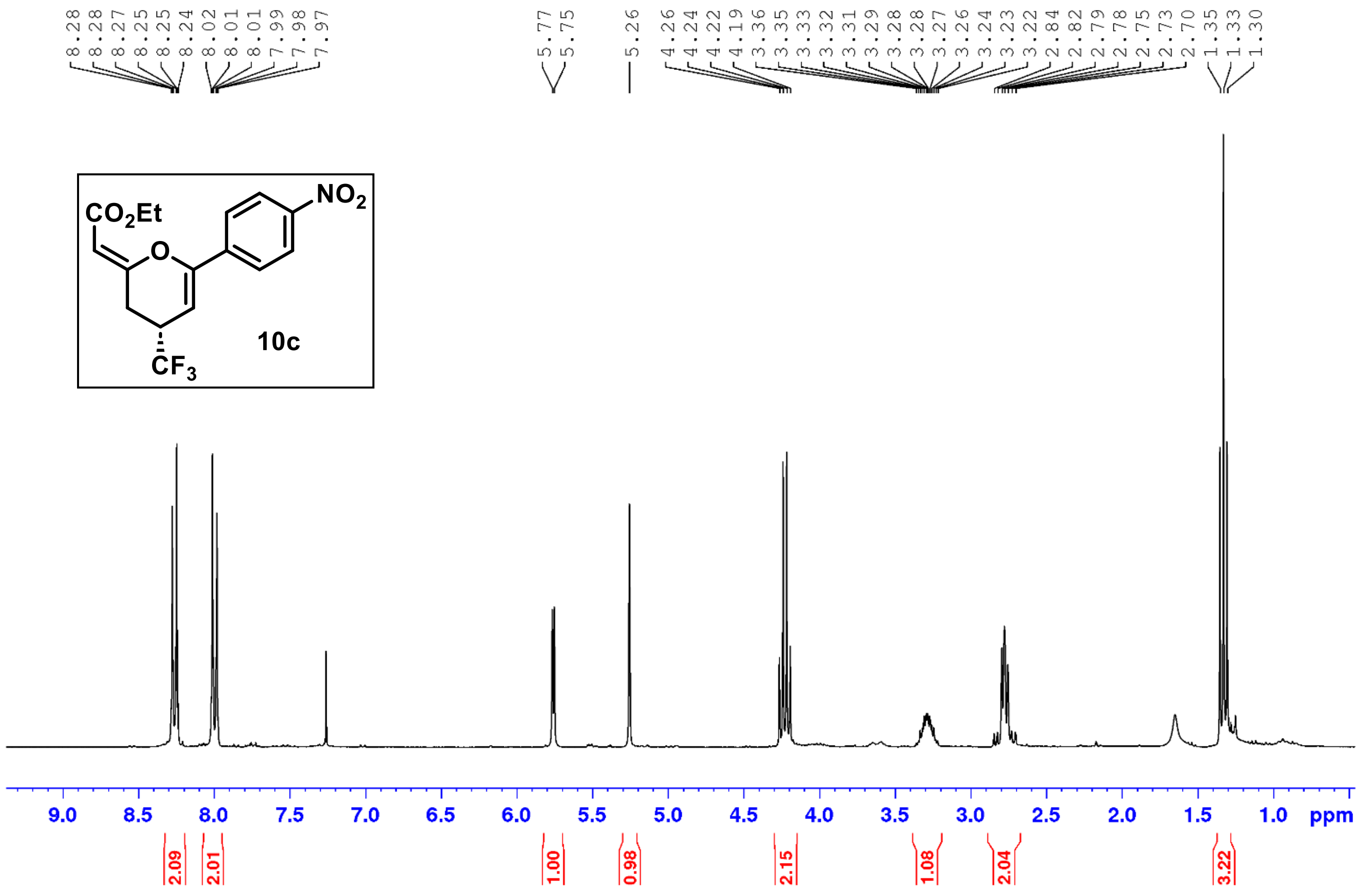
8.28 8.28 8.27 8.25 8.25 8.24 8.02 8.01 8.01 7.99 7.98 7.97
5.77 5.75
5.26
4.26 4.24 4.22 4.19 3.36 3.35 3.33 3.32 3.31 3.29 3.28 3.28 3.27 3.26 3.24 3.23 3.22 2.84 2.82 2.79 2.78 2.75 2.73 2.70 1.35 1.33 1.30
CO2Et
NO2
O
CF3
10c
9.0 8.5 8.0 7.5 7.0 6.5 6.0 5.5 5.0 4.5 4.0 3.5 3.0 2.5 2.0 1.5 1.0 ppm
2.09 2.01 1.00 0.98 2.15 1.08 2.04 3.22

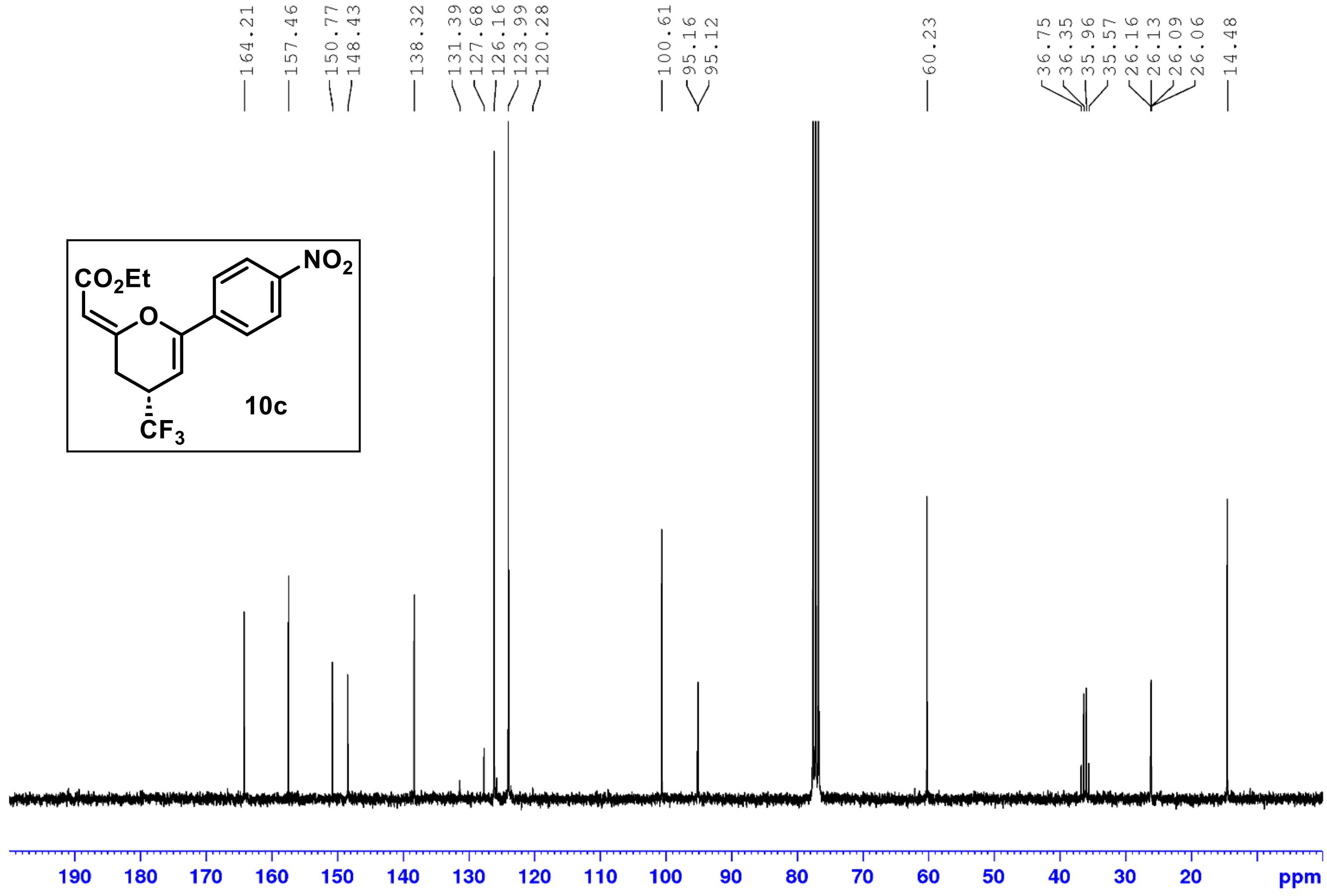
164.21
157.46
150.77 148.43
138.32
131.39 127.68 126.16 123.99 120.28
100.61
95.16 95.12
60.23
36.75 36.35 35.96 35.57 26.16 26.13 26.09 26.06
14.48
CO2Et
NO2
O
CF3
10c
190 180 170 160 150 140 130 120 110 100 90 80 70 60 50 40 30 20 ppm

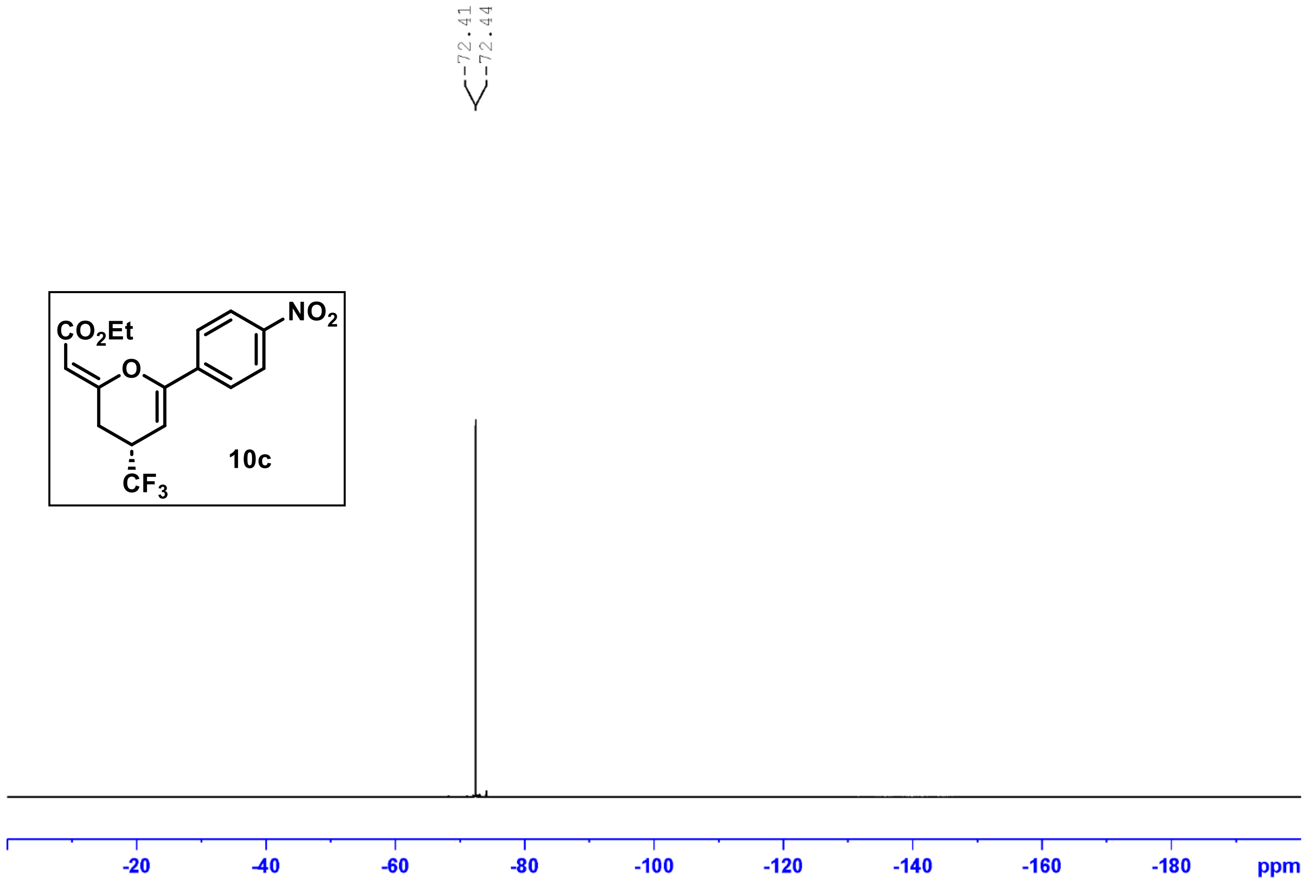

-72.41
-72.44
CO2Et
NO2
O
10c
CF3
-20
-40
-60
-80
-100
-120
-140
-160
-180
ppm

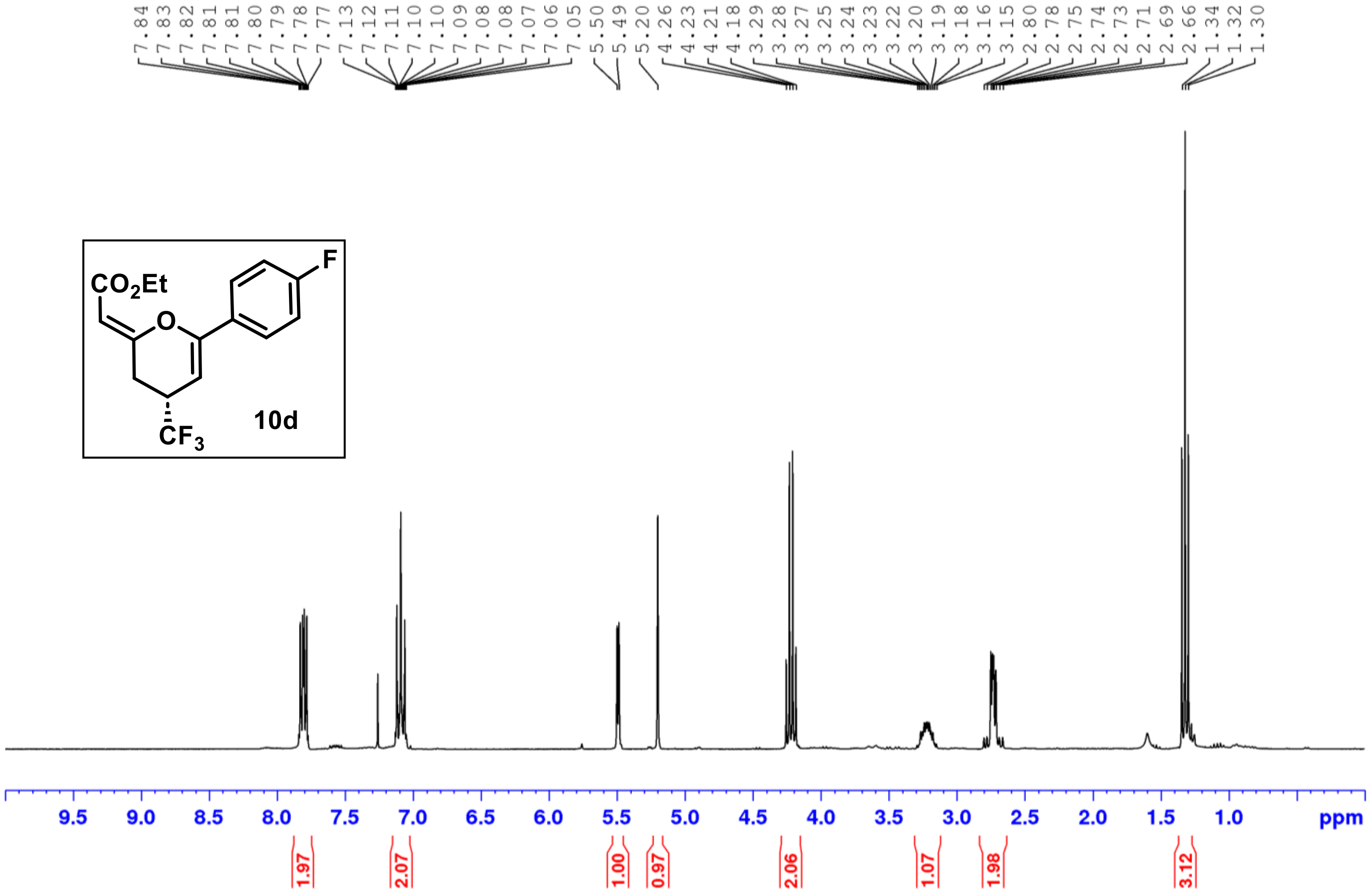

7.84 7.83 7.82 7.81 7.81 7.80 7.79 7.78 7.77 7.13 7.12 7.11 7.10 7.10 7.09 7.08 7.08 7.07 7.06 7.05 5.50 5.49 5.20 4.26 4.23 4.21 4.18 3.29 3.28 3.27 3.25 3.24 3.23 3.22 3.20 3.19 3.18 3.16 3.15 2.80 2.78 2.75 2.74 2.73 2.71 2.69 2.66 1.34 1.32 1.30
CO2Et
F
O
10d
CF3
9.5 9.0 8.5 8.0 7.5 7.0 6.5 6.0 5.5 5.0 4.5 4.0 3.5 3.0 2.5 2.0 1.5 1.0
ppm
1.97 2.07 1.00 0.97 2.06 1.07 1.98 3.12

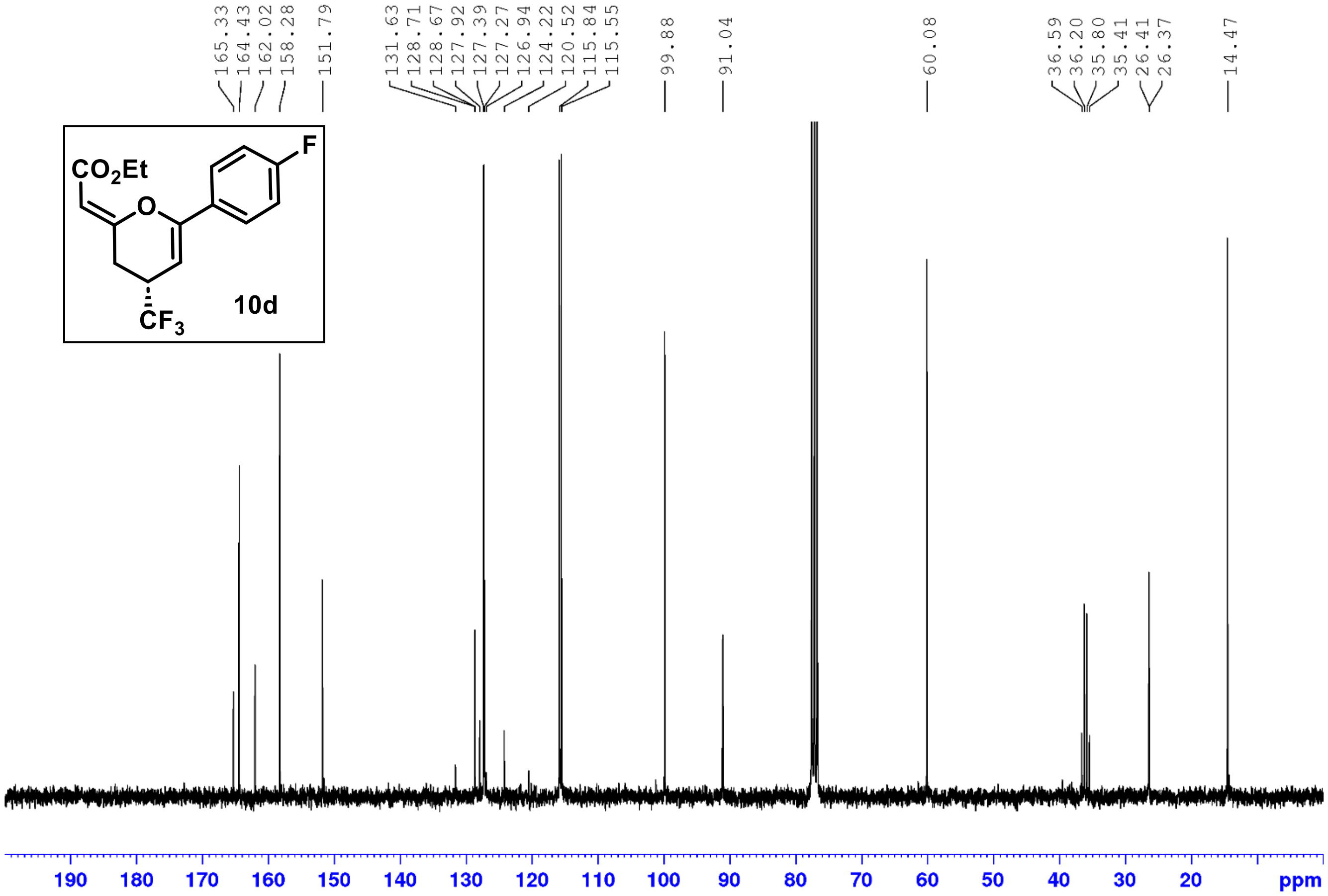
165.33
164.43
162.02
158.28
151.79
131.63
128.71
128.67
127.92
127.39
127.27
126.94
124.22
120.52
115.84
115.55
99.88
91.04
60.08
36.59
36.20
35.80
35.41
26.41
26.37
14.47
CO2Et
O
F
CF3
10d
190 180 170 160 150 140 130 120 110 100 90 80 70 60 50 40 30 20 ppm

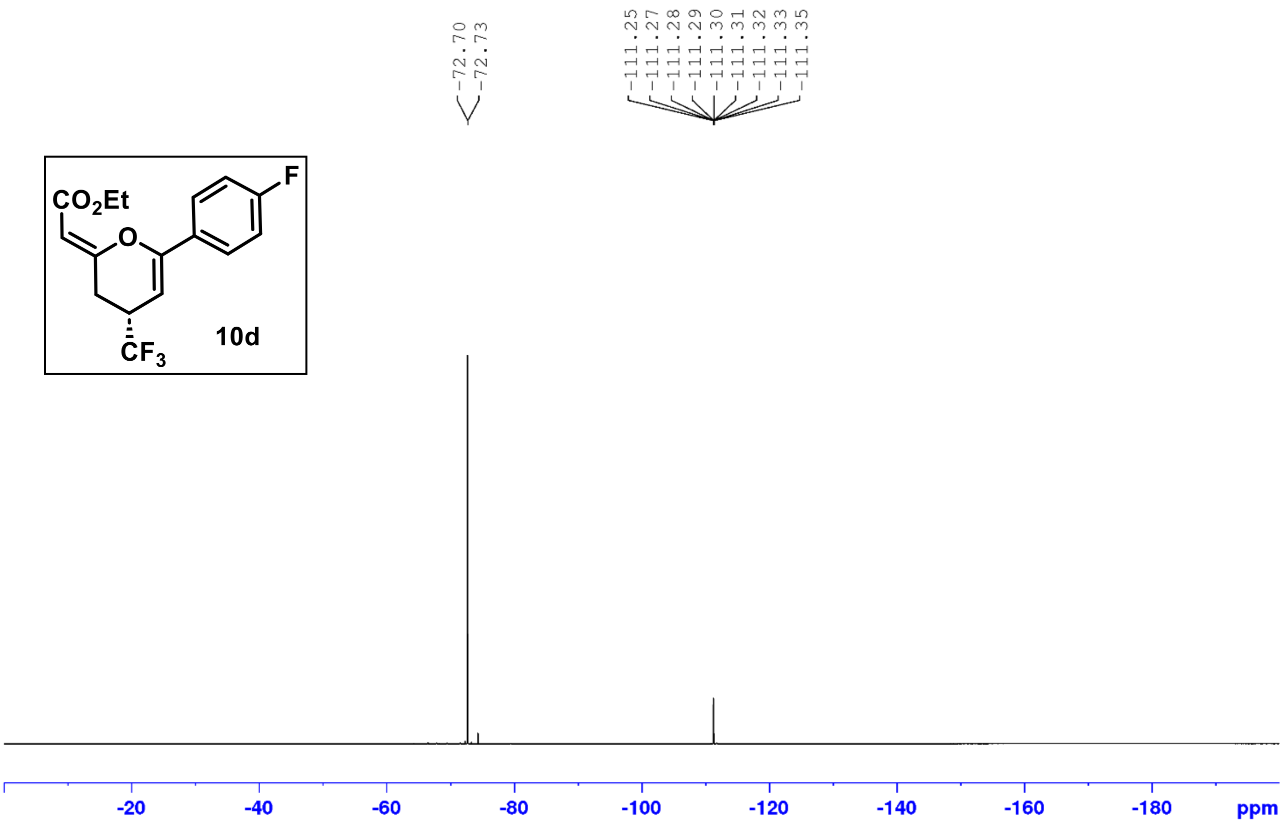
-72.70
-72.73
-111.25
-111.27
-111.28
-111.29
-111.30
-111.31
-111.32
-111.33
-111.35
CO2Et
O
F
CF3
10d
-20 -40 -60 -80 -100 -120 -140 -160 -180 ppm

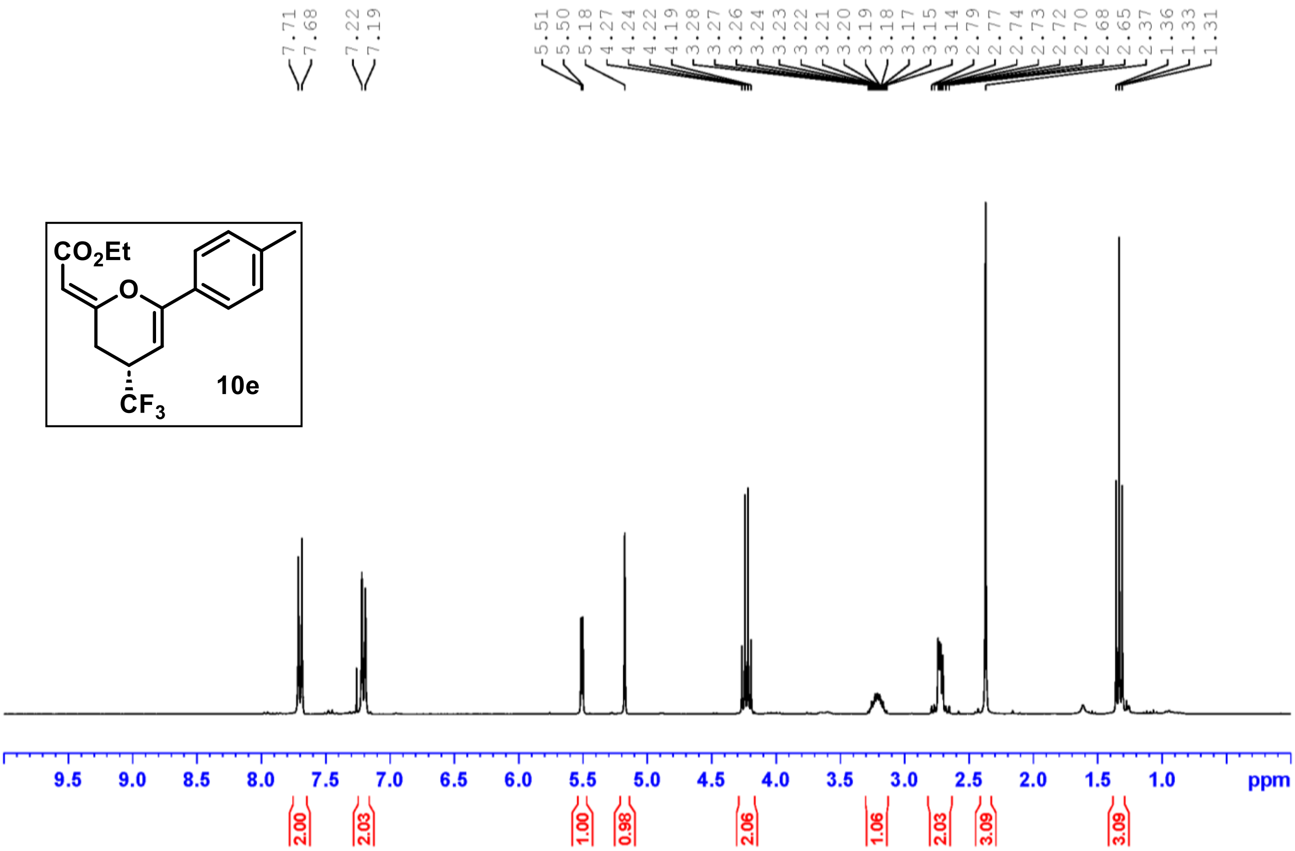
7.71
7.68
7.22
7.19
5.51
5.50
5.18
4.27
4.24
4.22
4.19
3.28
3.27
3.26
3.24
3.23
3.22
3.21
3.20
3.19
3.18
3.17
3.15
3.14
2.79
2.77
2.74
2.73
2.72
2.70
2.68
2.65
2.37
1.36
1.33
1.31
CO_2Et
O
CF_3
10e
9.5 9.0 8.5 8.0 7.5 7.0 6.5 6.0 5.5 5.0 4.5 4.0 3.5 3.0 2.5 2.0 1.5 1.0 ppm
2.00
2.03
1.00
0.98
2.06
1.06
2.03
3.09
3.09

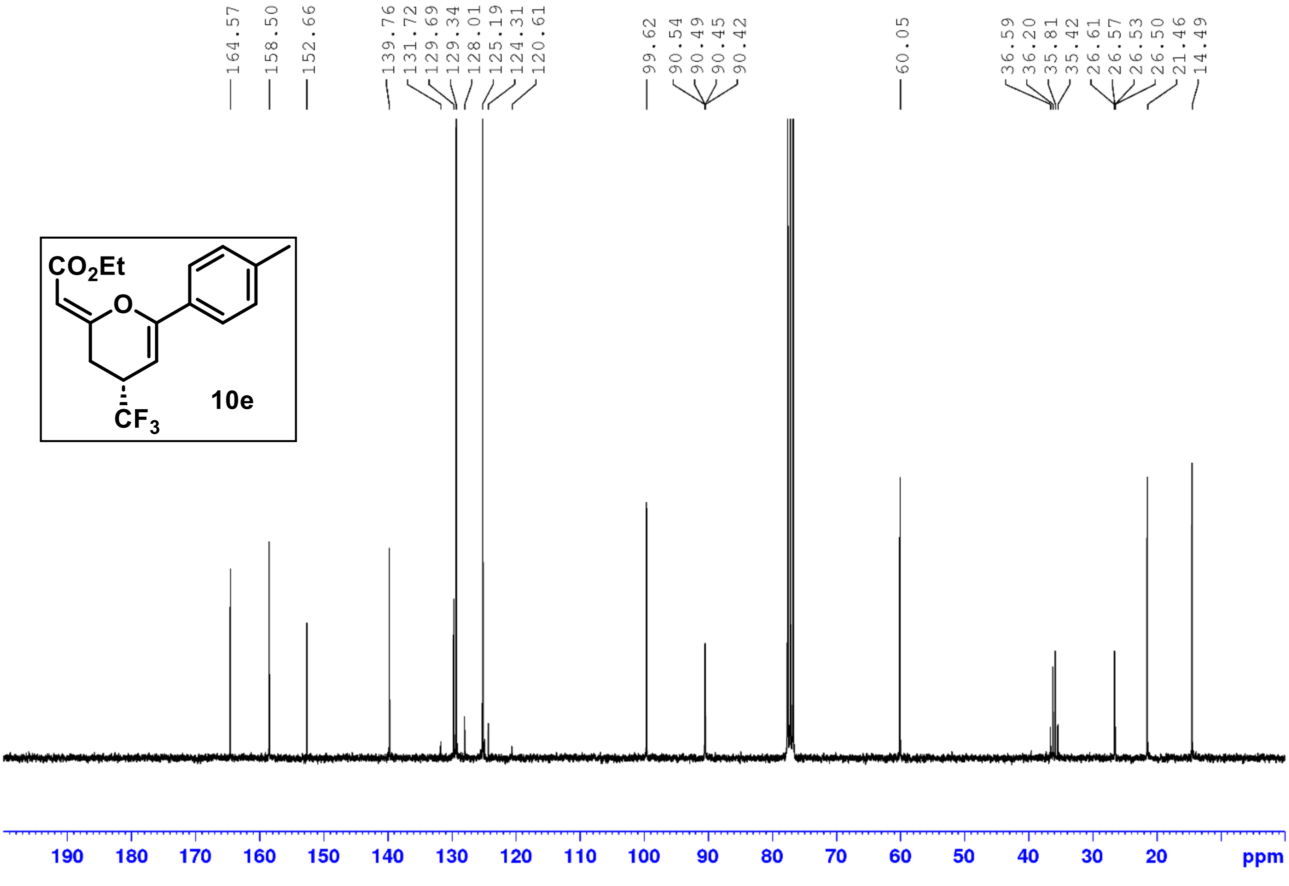
164.57
158.50
152.66
139.76
131.72
129.69
129.34
128.01
125.19
124.31
120.61
99.62
90.54
90.49
90.45
90.42
60.05
36.59
36.20
35.81
35.42
26.61
26.57
26.53
26.50
21.46
14.49
CO_2Et
O
CF_3
10e
190 180 170 160 150 140 130 120 110 100 90 80 70 60 50 40 30 20 ppm

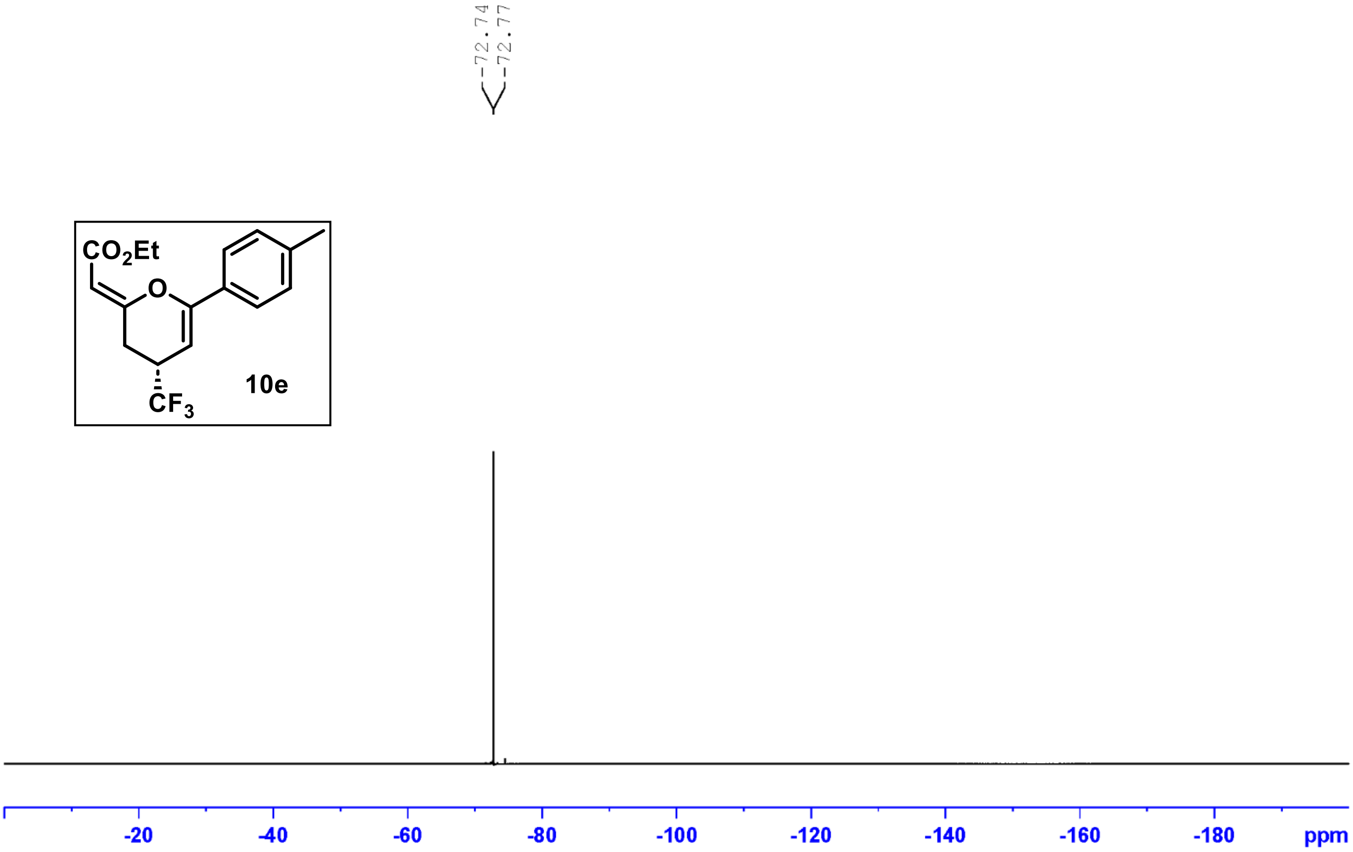
-72.74
-72.77
CO2Et
O
CF3
10e
-20
-40
-60
-80
-100
-120
-140
-160
-180
ppm

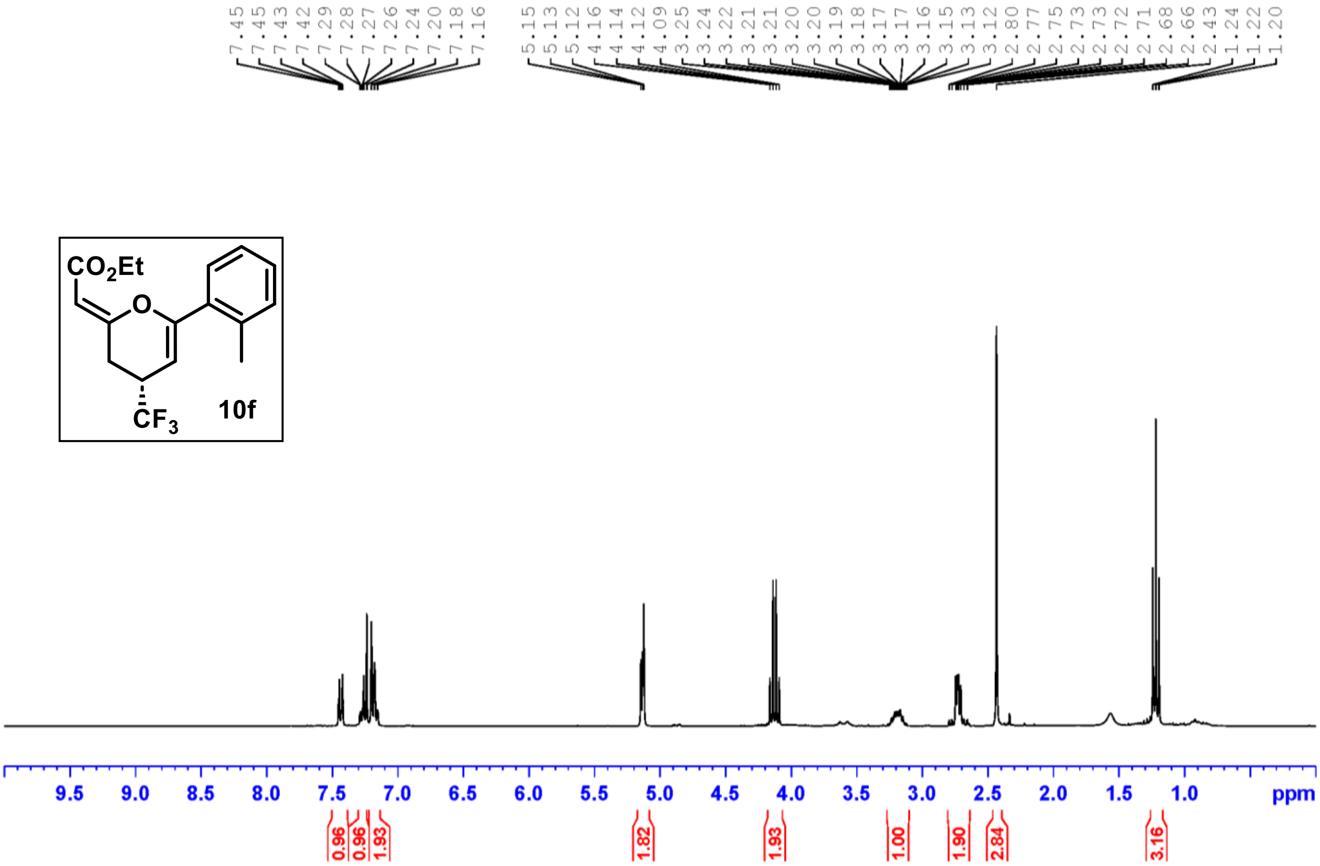
7.45 7.45 7.43 7.42 7.29 7.28 7.27 7.26 7.24 7.20 7.18 7.16
5.15 5.13 5.12 4.16 4.14 4.12 4.09 3.25 3.24 3.22 3.21 3.21 3.20 3.20 3.19 3.18 3.17 3.17 3.16 3.15 3.13 3.12 2.80 2.77 2.75 2.73 2.73 2.72 2.72 2.71 2.68 2.66 2.43 1.24 1.22 1.20
CO2Et
O
CF3
10f
9.5 9.0 8.5 8.0 7.5 7.0 6.5 6.0 5.5 5.0 4.5 4.0 3.5 3.0 2.5 2.0 1.5 1.0 ppm
0.96 0.96 1.93 1.82 1.93 1.00 1.90 2.84 3.16

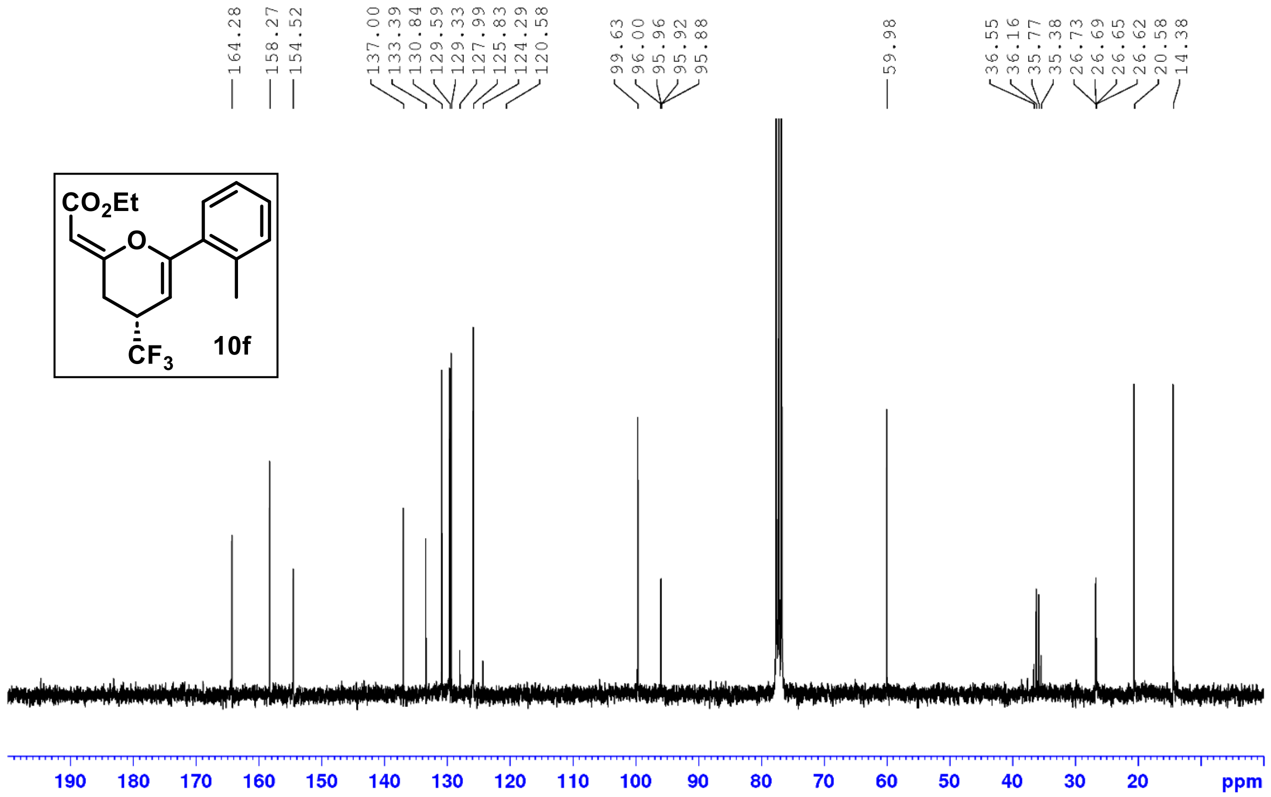

164.28
158.27
154.52
137.00
133.39
130.84
129.59
129.33
127.99
125.83
124.29
120.58
99.63
96.00
95.96
95.92
95.88
59.98
36.55
36.16
35.77
35.38
26.73
26.69
26.65
26.62
20.58
14.38
CO2Et
O
CF3
10f
190 180 170 160 150 140 130 120 110 100 90 80 70 60 50 40 30 20 ppm

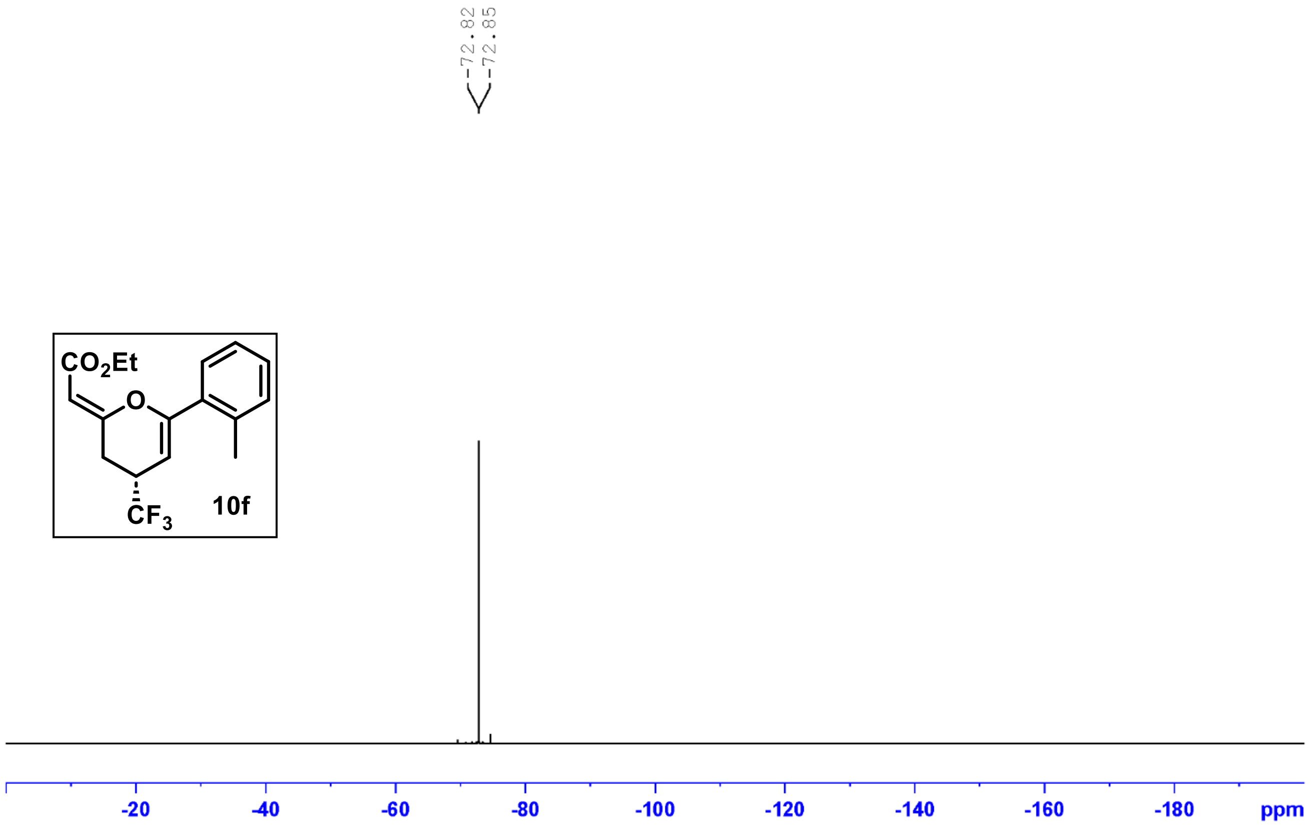

-72.82
-72.85
CO2Et
O
CF3
10f
-20 -40 -60 -80 -100 -120 -140 -160 -180 ppm

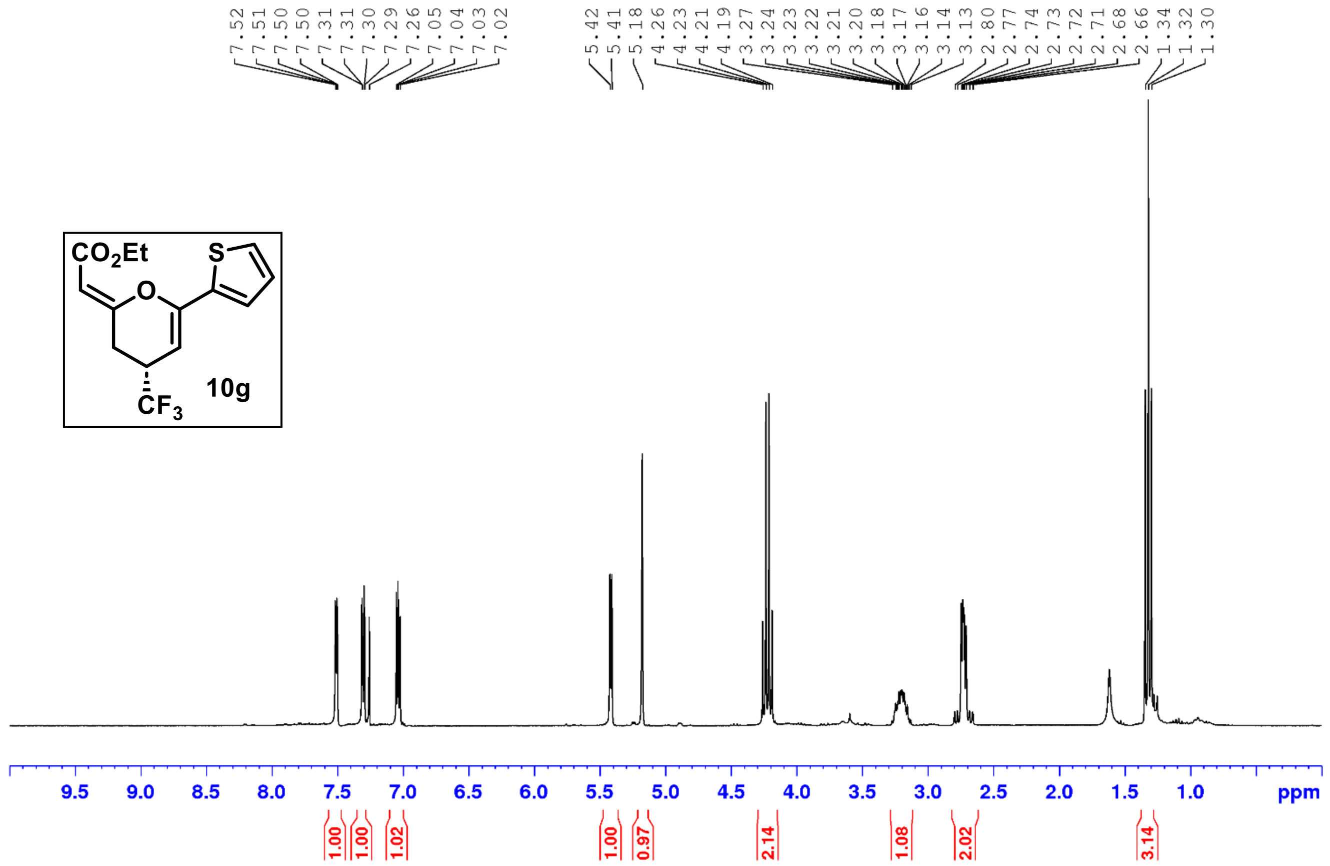
7.52 7.51 7.50 7.50 7.31 7.31 7.30 7.29 7.26 7.05 7.04 7.03 7.02
5.42 5.41 5.18 4.26 4.23 4.21 4.19 3.27 3.24 3.23 3.22 3.21 3.20 3.18 3.17 3.16 3.14 3.13 2.80 2.77 2.74 2.73 2.72 2.71 2.68 2.66 1.34 1.32 1.30
CO2Et
S
O
CF3
10g
9.5 9.0 8.5 8.0 7.5 7.0 6.5 6.0 5.5 5.0 4.5 4.0 3.5 3.0 2.5 2.0 1.5 1.0 ppm
1.00 1.00 1.02 1.00 0.97 2.14 1.08 2.02 3.14

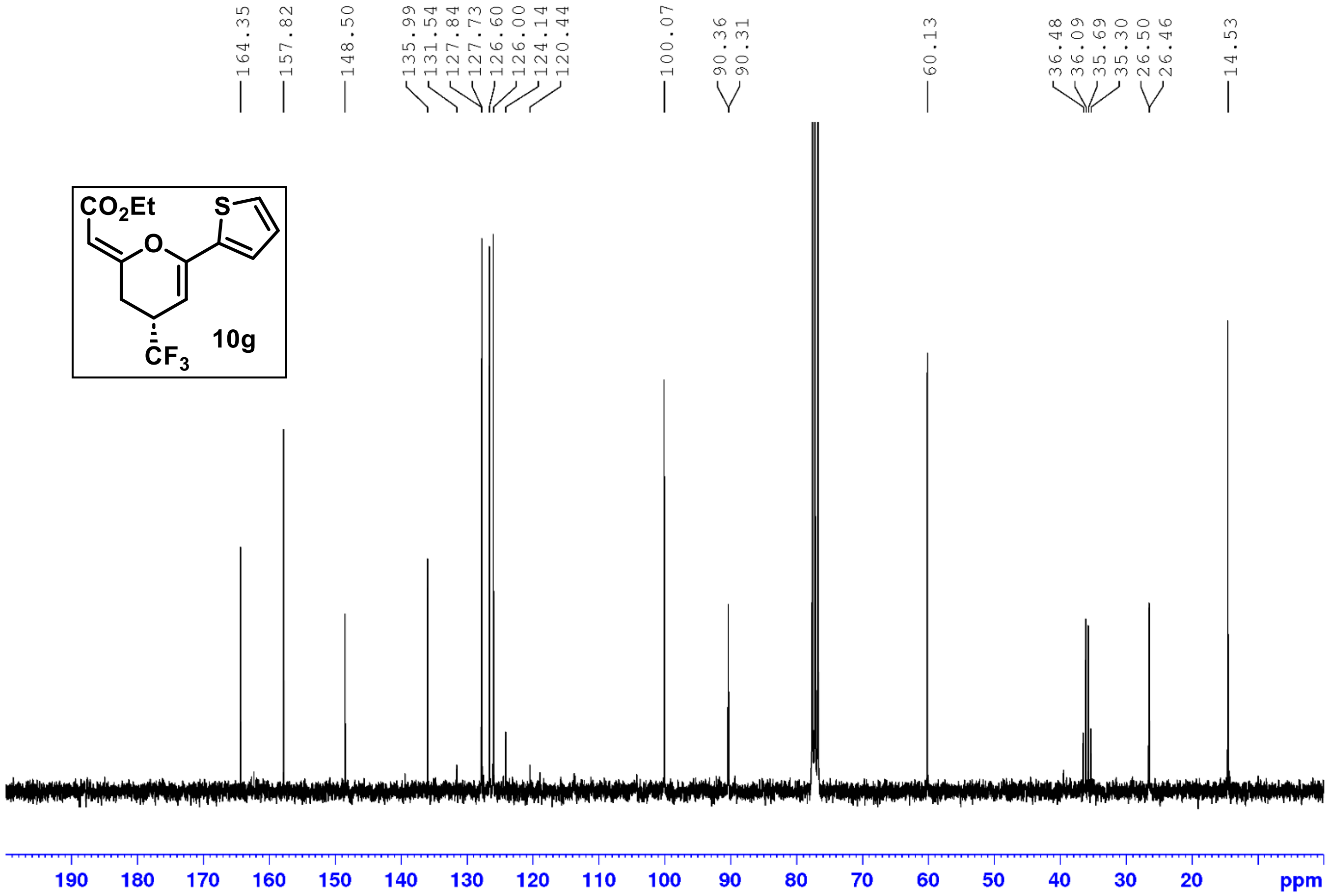
164.35 157.82 148.50 135.99 131.54 127.84 127.73 126.60 126.00 124.14 120.44 100.07 90.36 90.31 60.13 36.48 36.09 35.69 35.30 26.50 26.46 14.53
CO2Et
S
O
CF3
10g
190 180 170 160 150 140 130 120 110 100 90 80 70 60 50 40 30 20 ppm

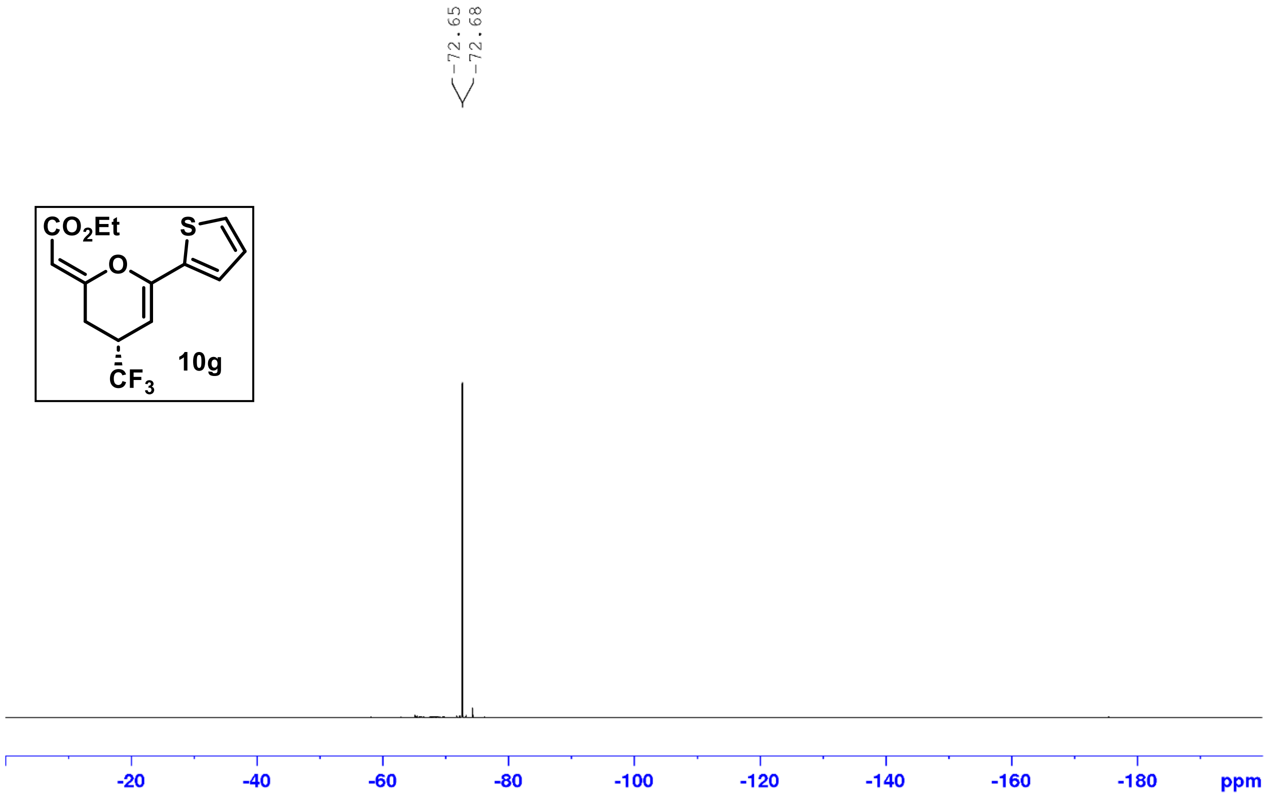
-72.65
-72.68
CO2Et
S
O
CF3
10g
-20
-40
-60
-80
-100
-120
-140
-160
-180
ppm

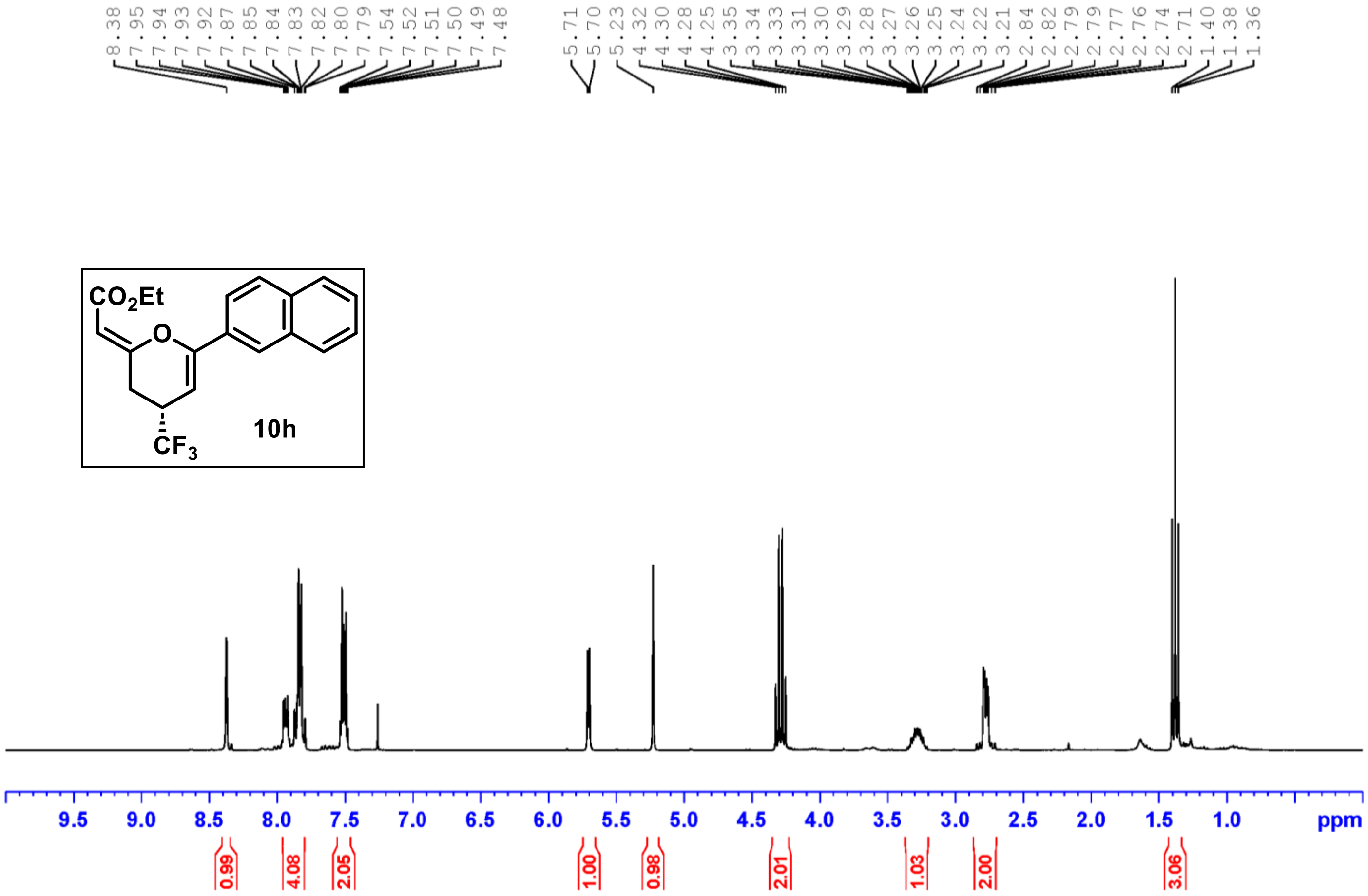
8.38 7.95 7.94 7.93 7.92 7.87 7.85 7.84 7.83 7.82 7.80 7.79 7.54 7.52 7.51 7.50 7.49 7.48
5.71 5.70 5.23 4.32 4.30 4.28 4.25 3.35 3.34 3.33 3.31 3.30 3.29 3.28 3.27 3.26 3.25 3.24 3.22 3.21 2.84 2.82 2.79 2.79 2.77 2.76 2.74 2.71 1.40 1.38 1.36
CO2Et
O
CF3
10h
9.5
9.0
8.5
8.0
7.5
7.0
6.5
6.0
5.5
5.0
4.5
4.0
3.5
3.0
2.5
2.0
1.5
1.0
ppm
0.99
4.08
2.05
1.00
0.98
2.01
1.03
2.00
3.06

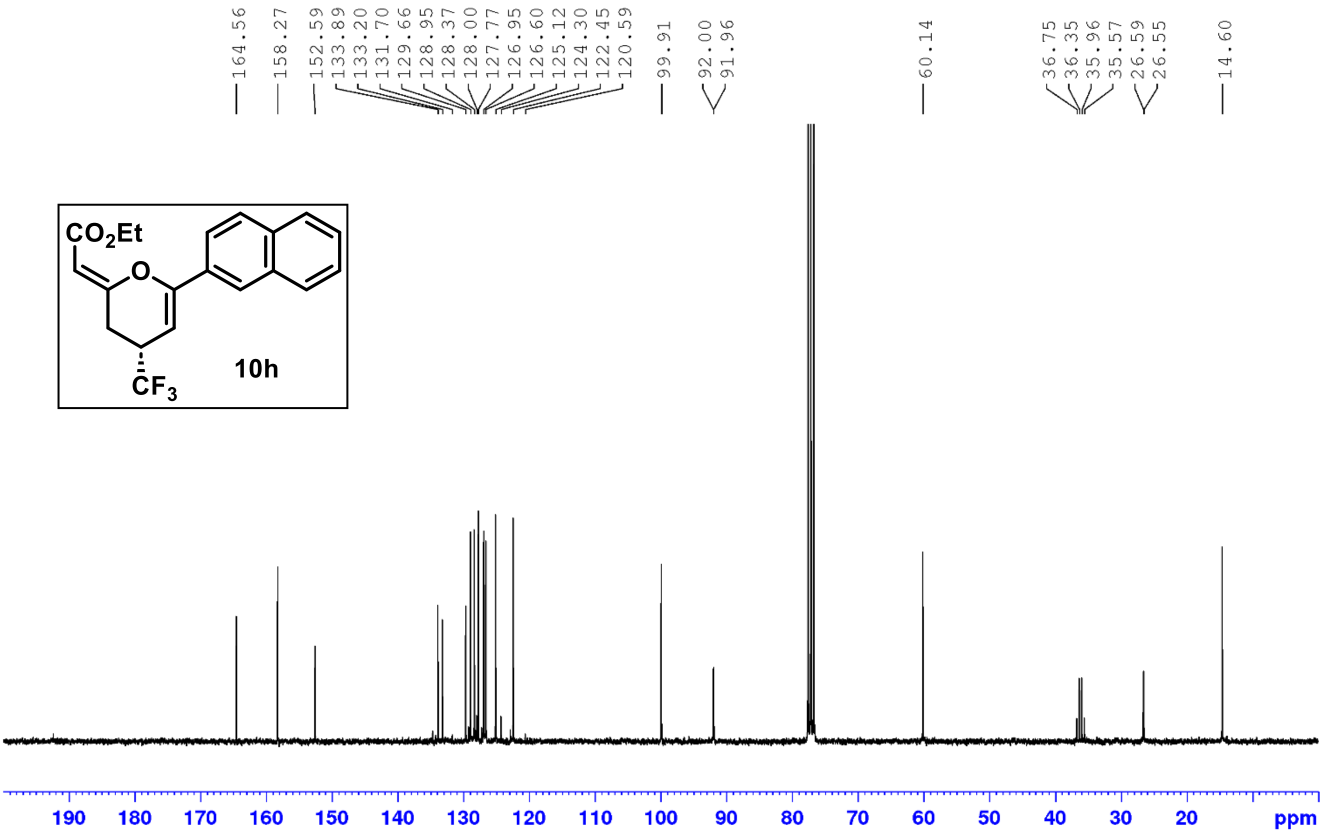
164.56
158.27
152.59
133.89
133.20
131.70
129.66
128.95
128.37
128.00
127.77
126.95
126.60
125.12
124.30
122.45
120.59
99.91
92.00
91.96
60.14
36.75
36.35
35.96
35.57
26.59
26.55
14.60
CO2Et
O
CF3
10h
190 180 170 160 150 140 130 120 110 100 90 80 70 60 50 40 30 20 ppm

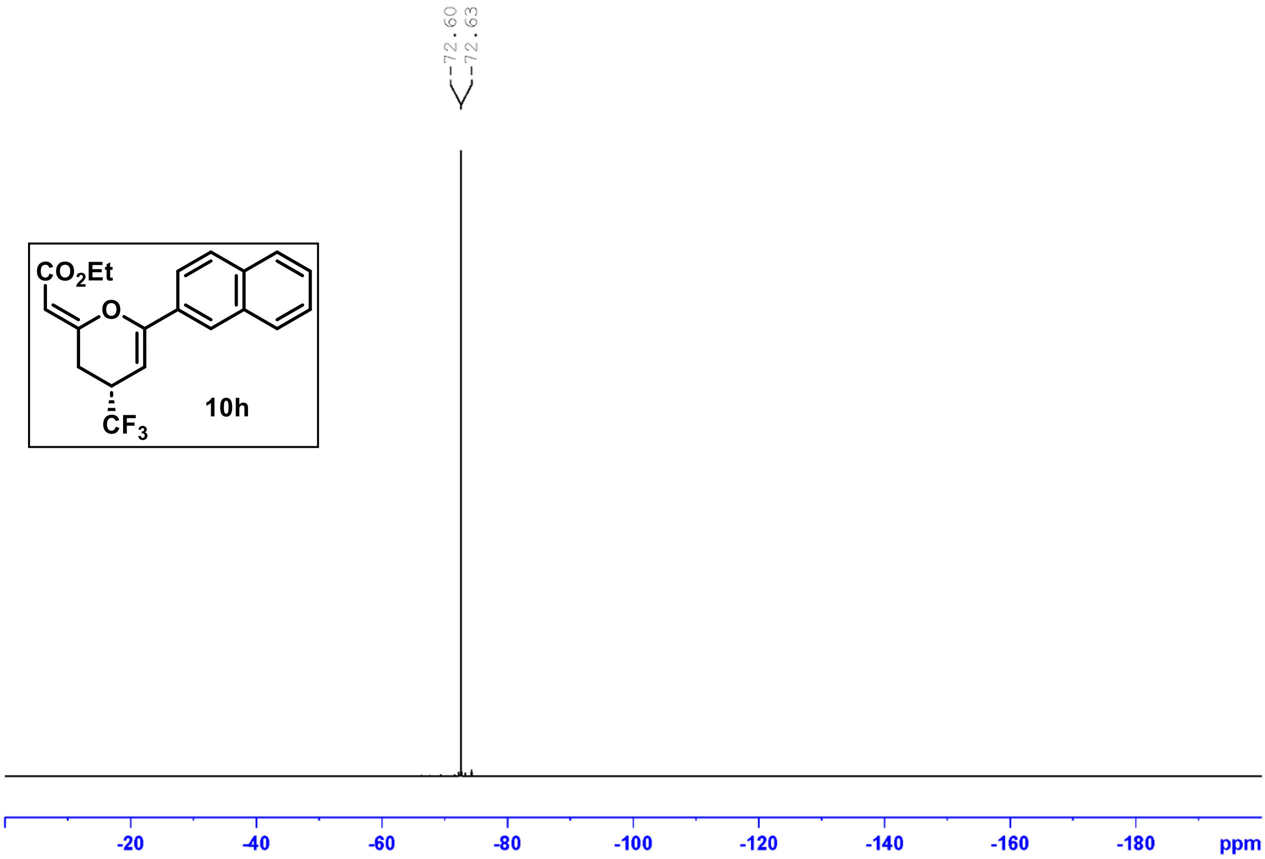
-72.60
-72.63
CO2Et
O
CF3
10h
-20 -40 -60 -80 -100 -120 -140 -160 -180 ppm

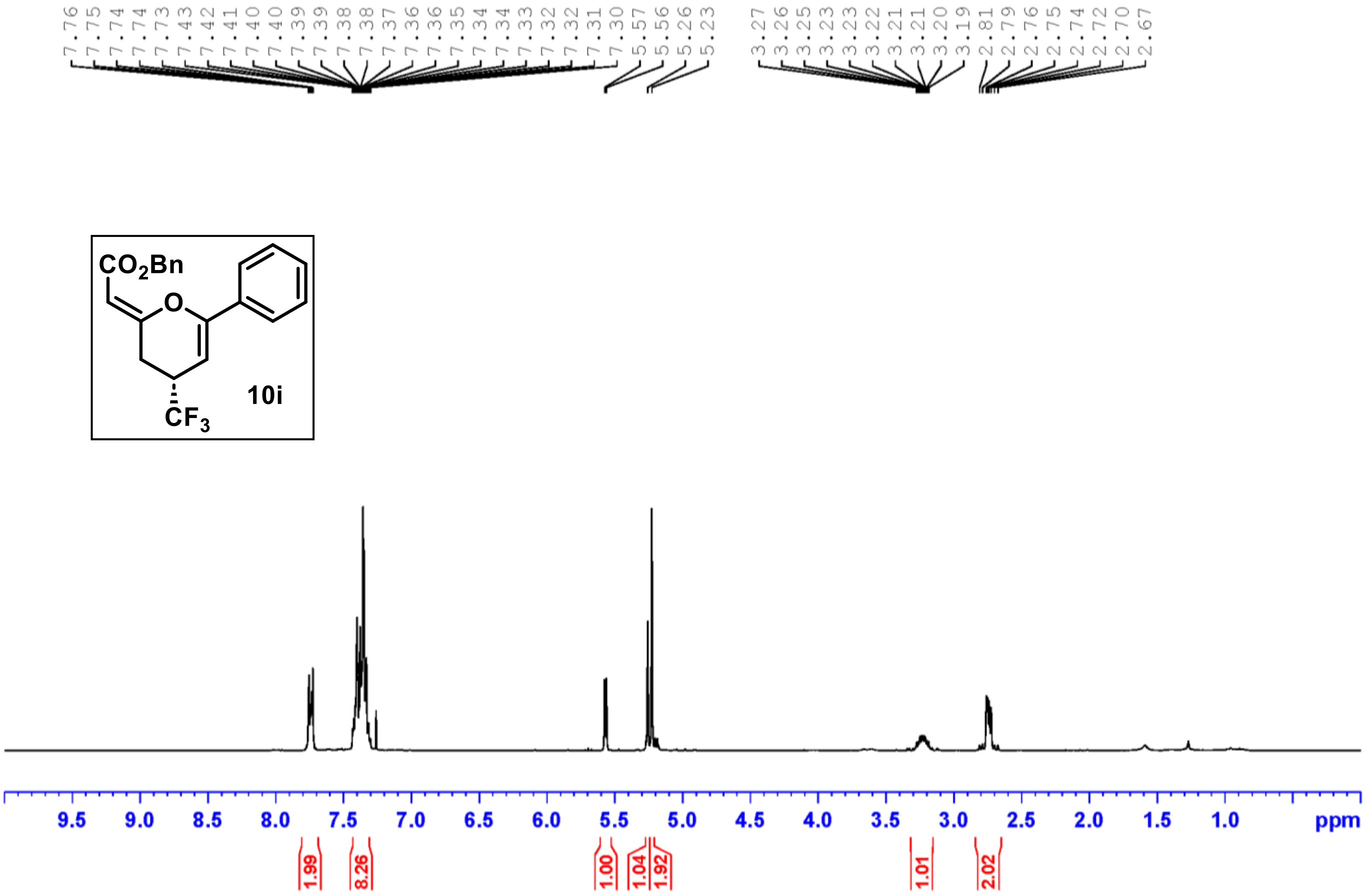
7.76 7.75 7.74 7.74 7.73 7.43 7.42 7.41 7.40 7.40 7.39 7.39 7.38 7.38 7.37 7.36 7.36 7.35 7.34 7.34 7.33 7.32 7.32 7.31 7.30 5.57 5.56 5.26 5.23
3.27 3.26 3.25 3.23 3.23 3.22 3.21 3.21 3.20 3.19 2.81 2.79 2.76 2.75 2.74 2.72 2.70 2.67
CO_2Bn
O
CF_3
10i
9.5 9.0 8.5 8.0 7.5 7.0 6.5 6.0 5.5 5.0 4.5 4.0 3.5 3.0 2.5 2.0 1.5 1.0 ppm
1.99 8.26 1.00 1.04 1.92 1.01 2.02

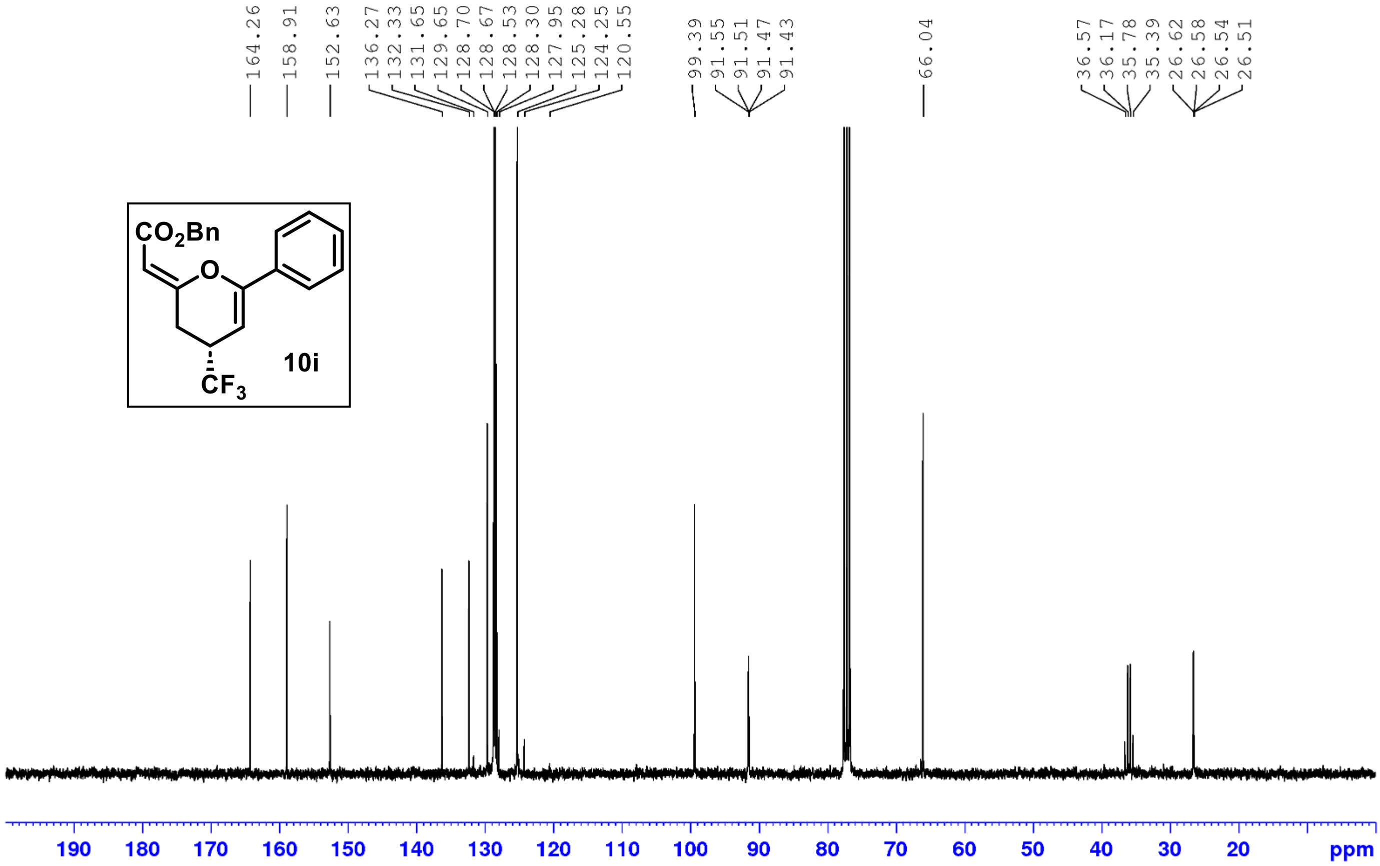
164.26 158.91 152.63 136.27 132.33 131.65 129.65 128.70 128.67 128.53 128.30 127.95 125.28 124.25 120.55 99.39 91.55 91.51 91.47 91.43 66.04 36.57 36.17 35.78 35.39 26.62 26.58 26.54 26.51
CO_2Bn
O
CF_3
10i
190 180 170 160 150 140 130 120 110 100 90 80 70 60 50 40 30 20 ppm

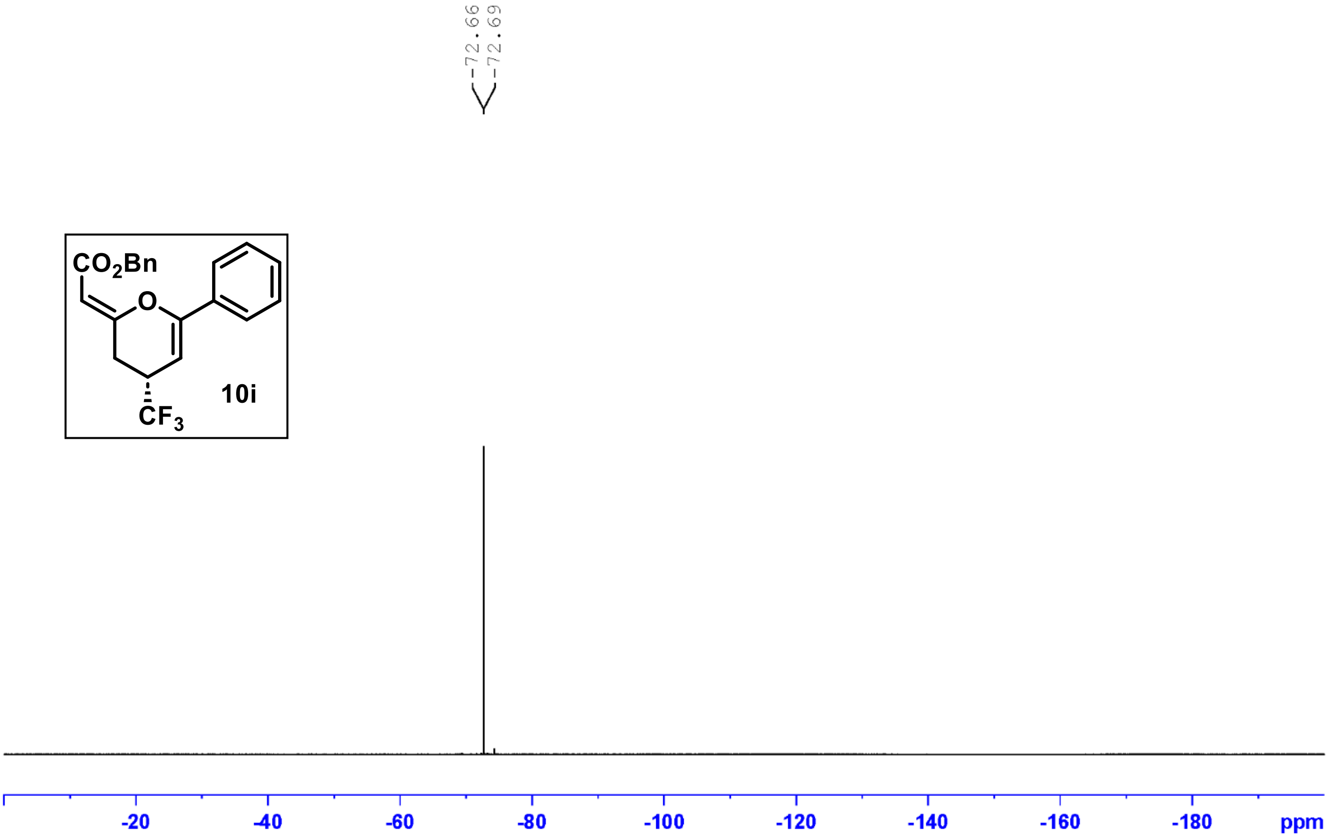
-72.66
-72.69
CO2Bn
O
10i
CF3
-20
-40
-60
-80
-100
-120
-140
-160
-180
ppm

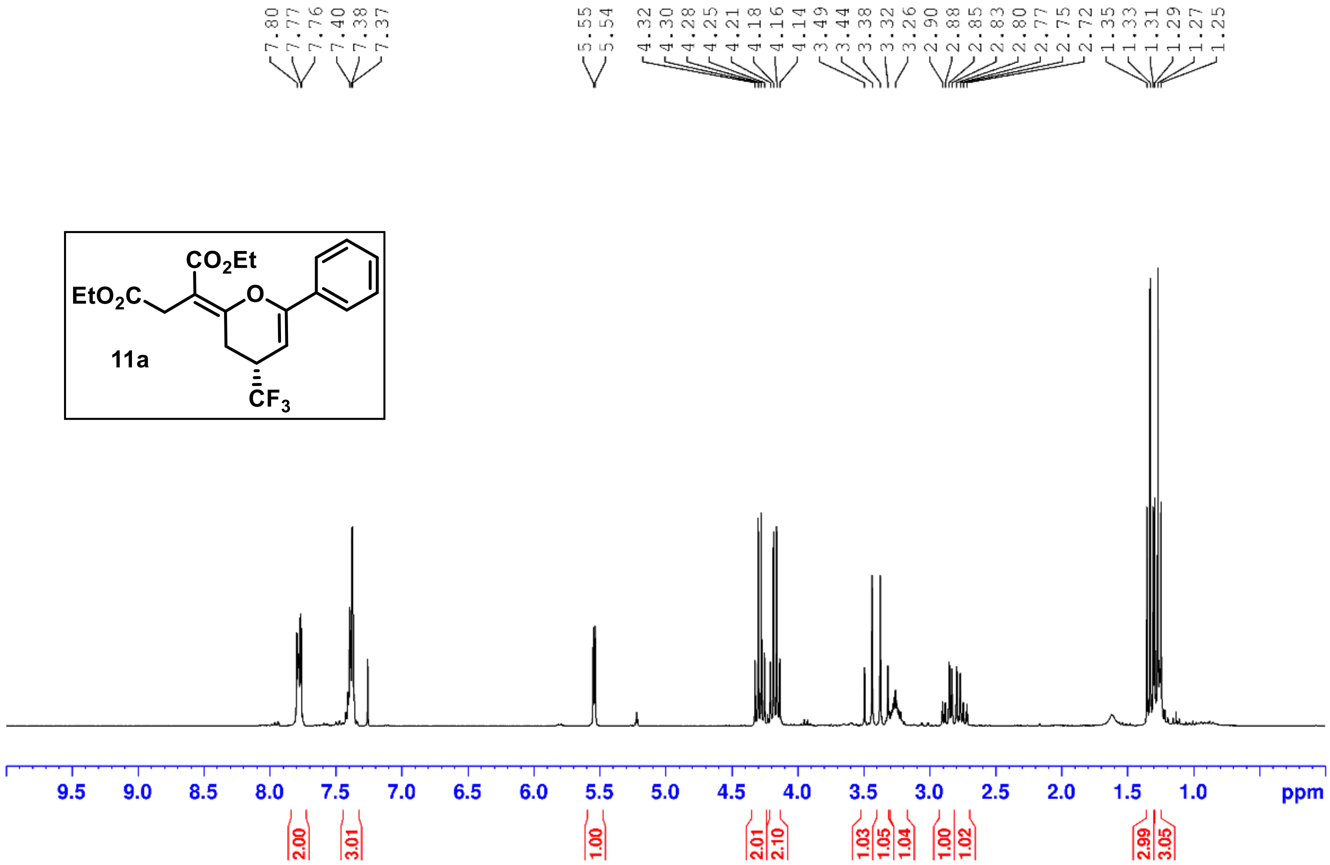
7.80 7.77 7.76 7.40 7.38 7.37
5.55 5.54
4.32 4.30 4.28 4.25 4.21 4.18 4.16 4.14
3.49 3.44 3.38 3.32 3.26
2.90 2.88 2.85 2.83 2.80 2.77 2.75 2.72
1.35 1.33 1.31 1.29 1.27 1.25
CO2Et
EtO2C
O
11a
CF3
9.5 9.0 8.5 8.0 7.5 7.0 6.5 6.0 5.5 5.0 4.5 4.0 3.5 3.0 2.5 2.0 1.5 1.0 ppm
2.00 3.01 1.00 2.01 2.10 1.03 1.05 1.04 1.00 1.02 2.99 3.05

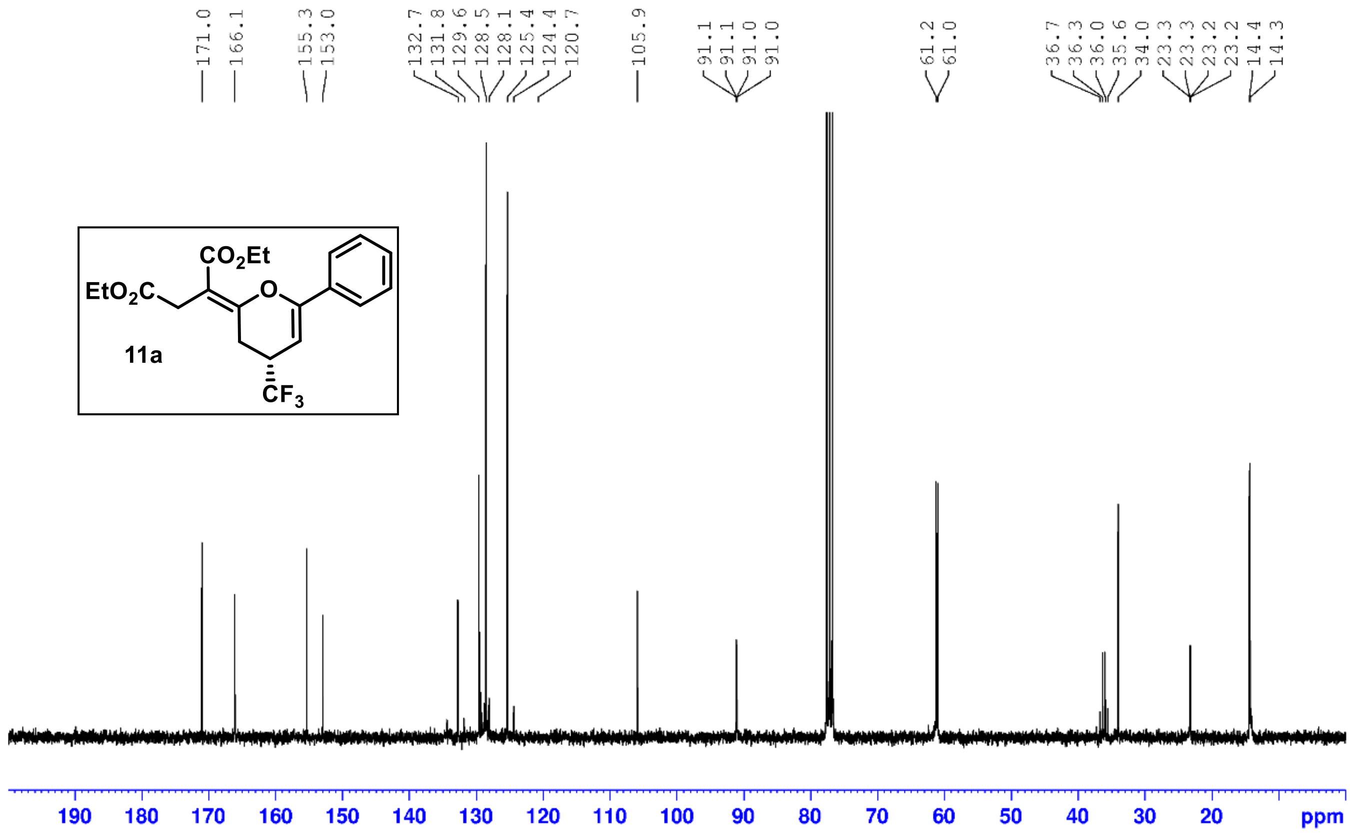
171.0
166.1
155.3
153.0
132.7
131.8
129.6
128.5
128.1
125.4
124.4
120.7
105.9
91.1
91.1
91.0
91.0
61.2
61.0
36.7
36.3
36.0
35.6
34.0
23.3
23.3
23.2
23.2
14.4
14.3
CO2Et
EtO2C
O
11a
CF3
190 180 170 160 150 140 130 120 110 100 90 80 70 60 50 40 30 20 ppm

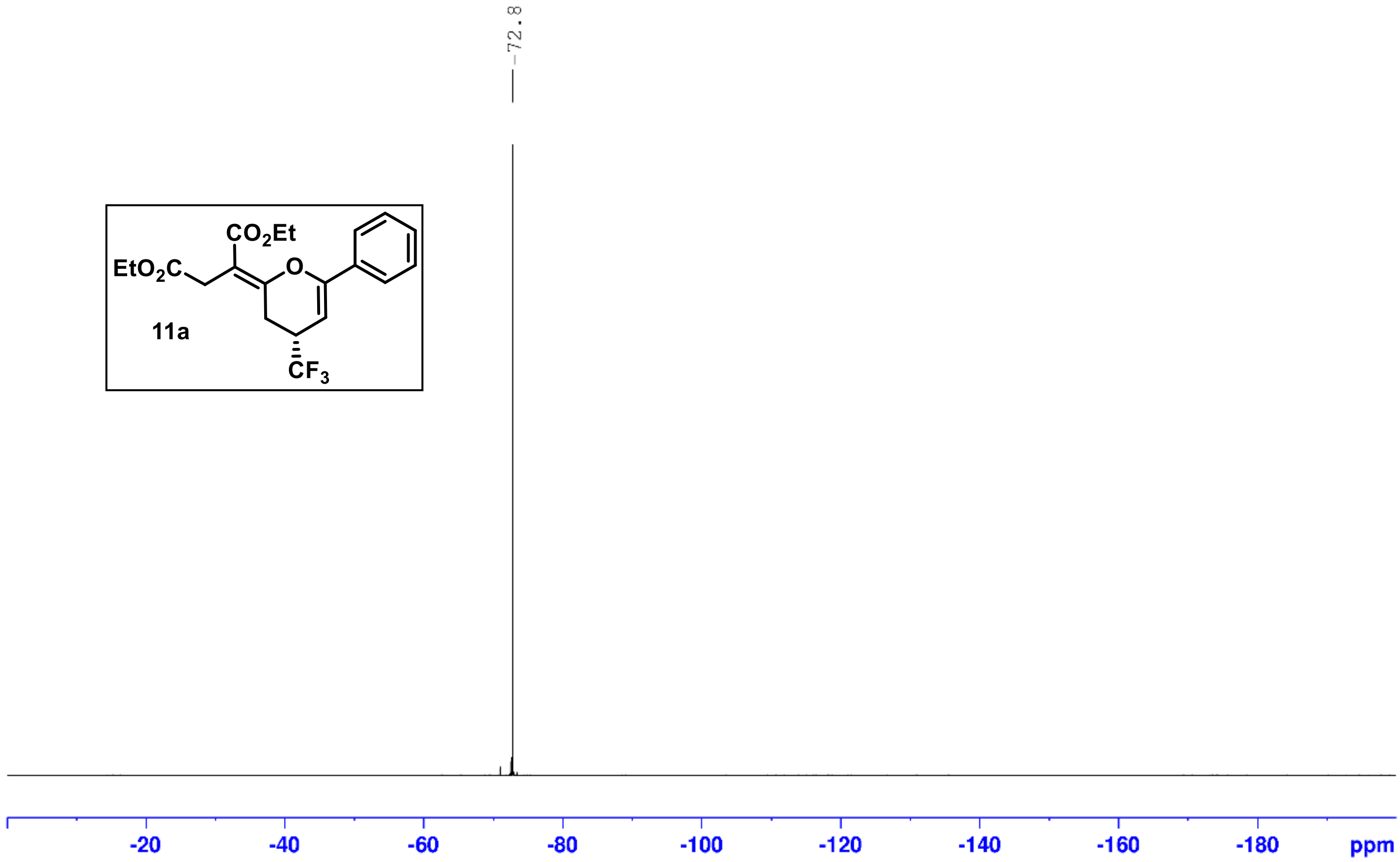
-72.8
CO2Et
EtO2C
O
11a
CF3
-20 -40 -60 -80 -100 -120 -140 -160 -180 ppm

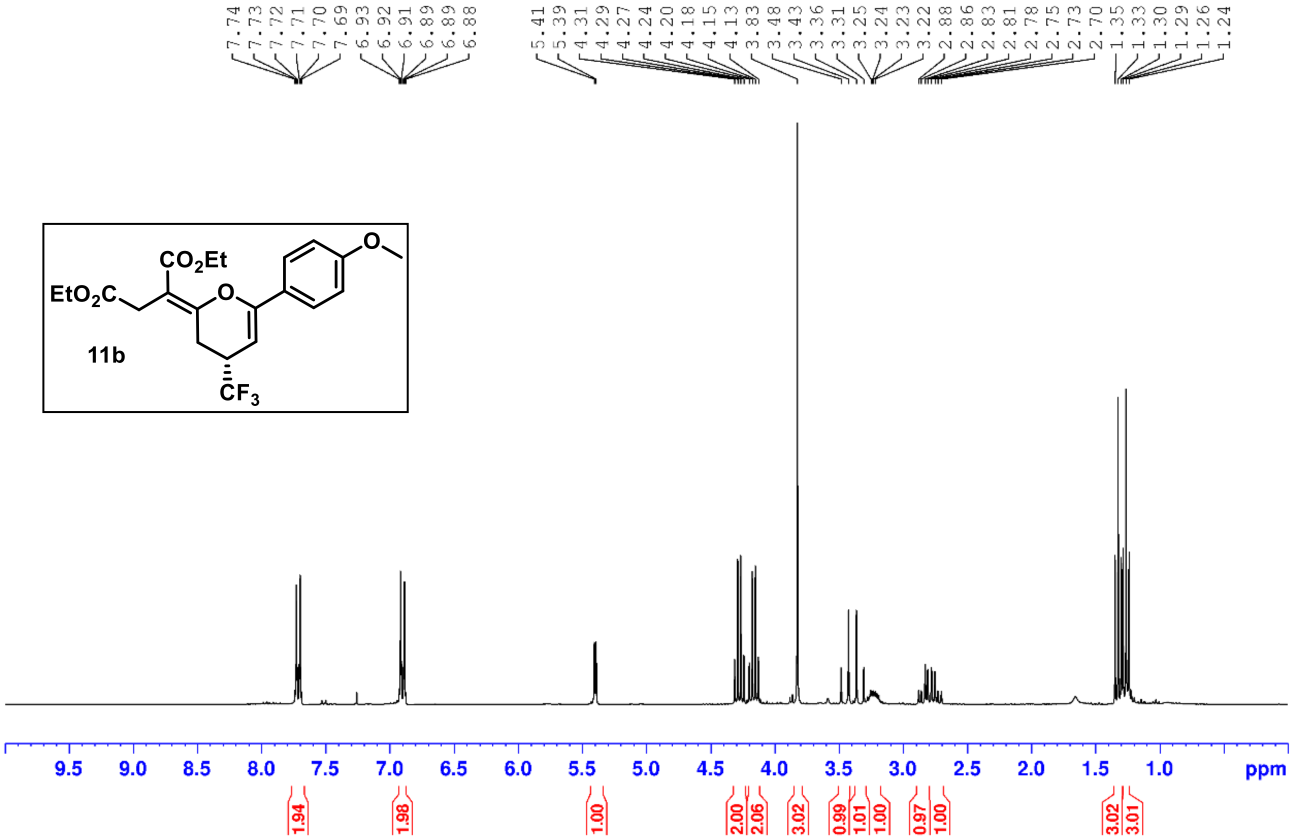
7.74 7.73 7.72 7.71 7.70 7.69 6.93 6.92 6.91 6.89 6.88
5.41 5.39 4.31 4.29 4.27 4.24 4.20 4.18 4.15 4.13 3.83 3.48 3.43 3.36 3.31 3.25 3.24 3.23 3.22 2.88 2.86 2.83 2.81 2.78 2.75 2.73 2.70 1.35 1.33 1.30 1.29 1.26 1.24
CO2Et
EtO2C
O
11b
CF3
9.5 9.0 8.5 8.0 7.5 7.0 6.5 6.0 5.5 5.0 4.5 4.0 3.5 3.0 2.5 2.0 1.5 1.0 ppm
1.94 1.98 1.00 2.00 2.06 3.02 0.99 1.01 1.00 0.97 1.00 3.02 3.01

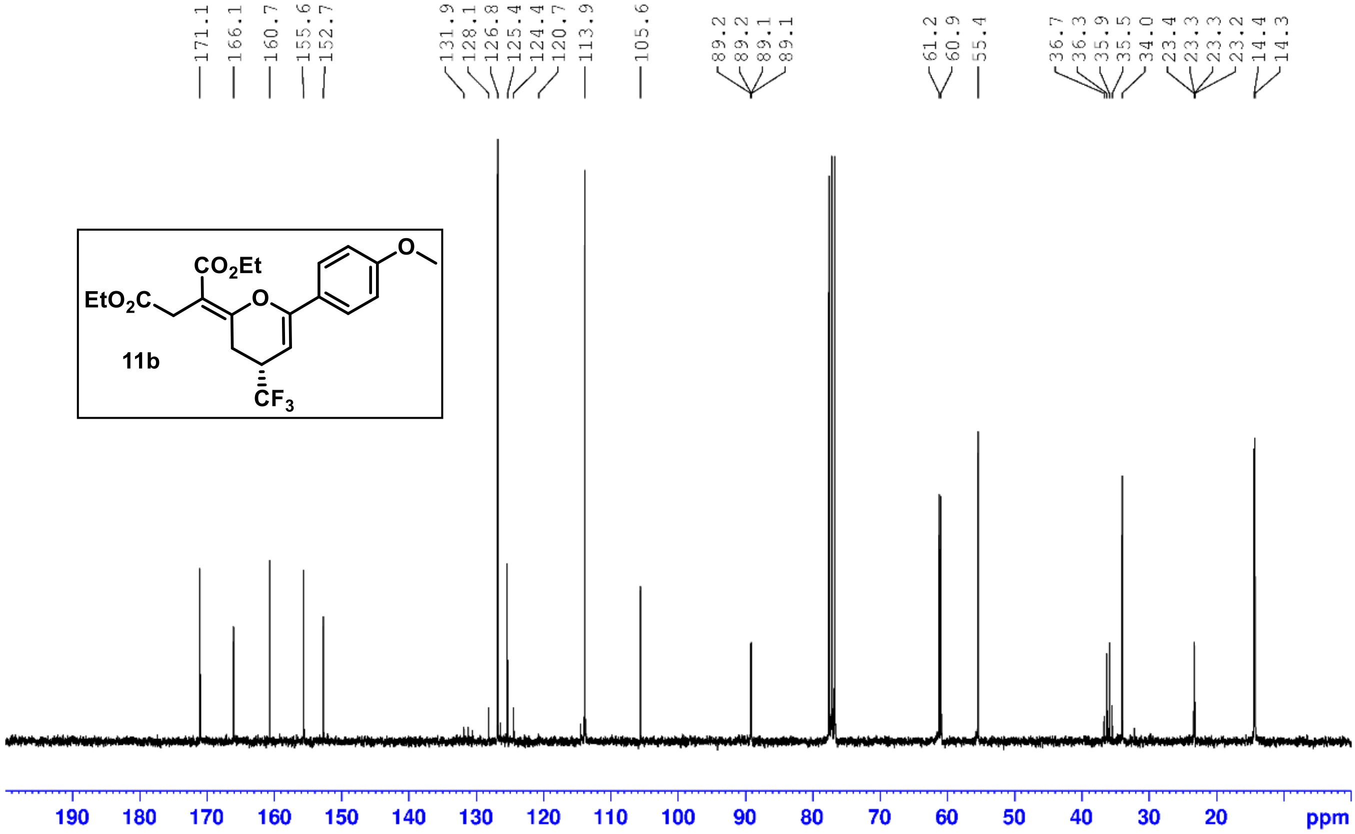
171.1 166.1 160.7 155.6 152.7
131.9 128.1 126.8 125.4 124.4 120.7 113.9 105.6
89.2 89.2 89.1 89.1
61.2 60.9 55.4
36.7 36.3 35.9 35.5 34.0 23.4 23.3 23.3 23.2 14.4 14.3
CO2Et
EtO2C
O
11b
CF3
190 180 170 160 150 140 130 120 110 100 90 80 70 60 50 40 30 20 ppm

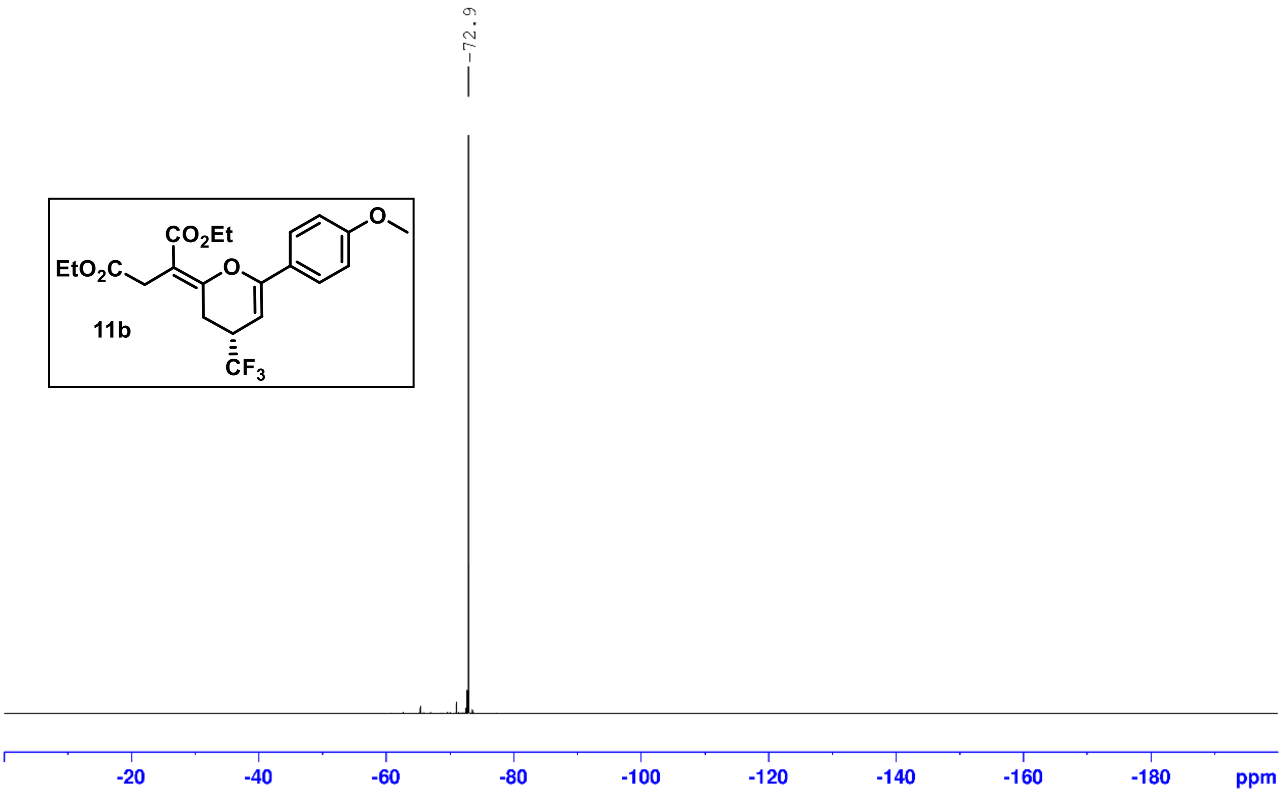
-72.9
CO_2Et
EtO_2C
O
11b
CF_3
-20
-40
-60
-80
-100
-120
-140
-160
-180
ppm

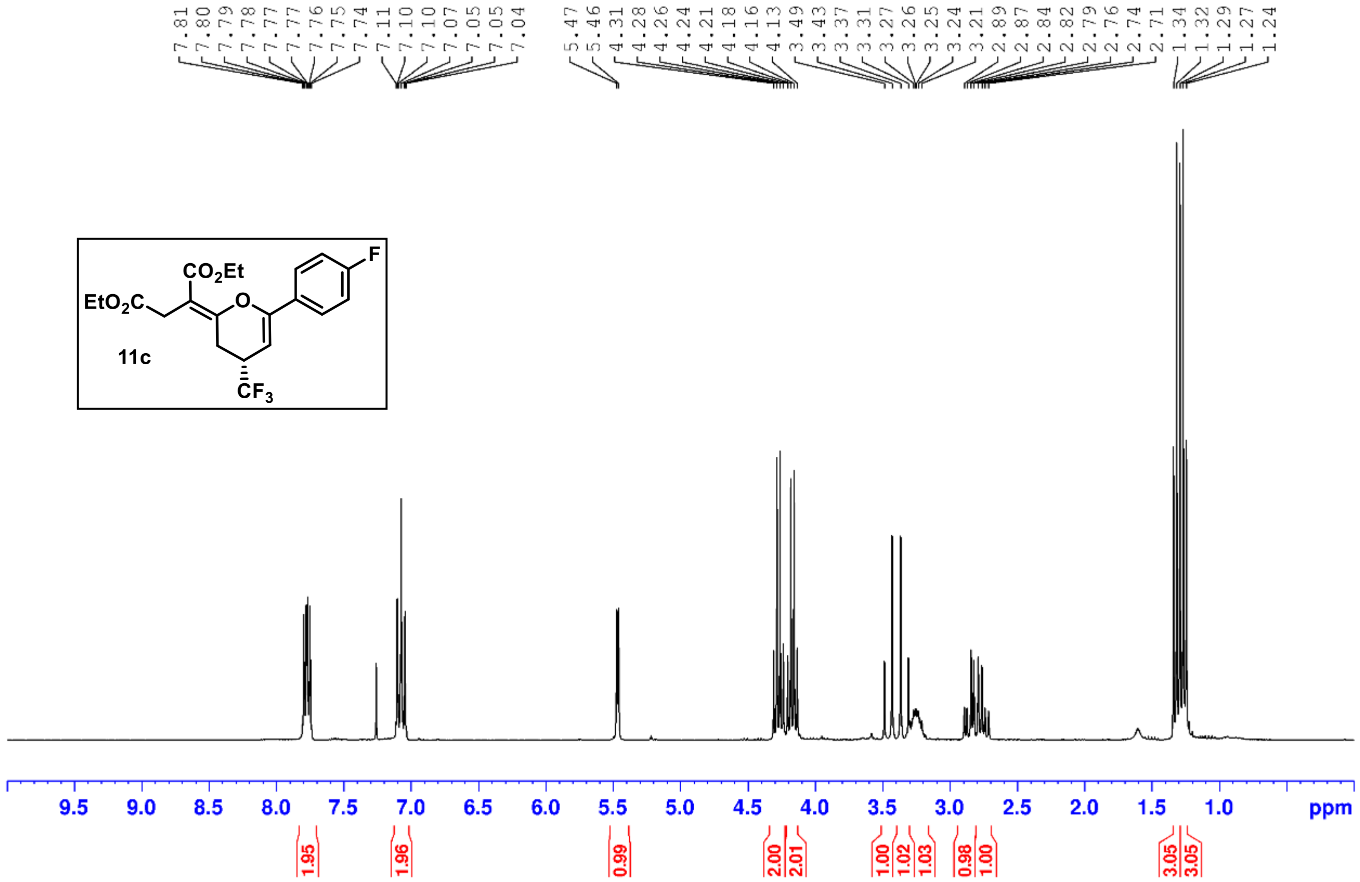
7.81 7.80 7.79 7.78 7.77 7.77 7.76 7.75 7.74 7.11 7.10 7.07 7.07 7.05 7.05 7.04
5.47 5.46 4.31 4.28 4.26 4.24 4.21 4.18 4.16 4.13 3.49 3.43 3.37 3.31 3.27 3.26 3.25 3.24 3.21 2.89 2.87 2.84 2.82 2.79 2.76 2.74 2.71 1.34 1.32 1.29 1.27 1.24
CO_2Et
EtO_2C
O
F
11c
CF_3
9.5
9.0
8.5
8.0
7.5
7.0
6.5
6.0
5.5
5.0
4.5
4.0
3.5
3.0
2.5
2.0
1.5
1.0
ppm
1.95
1.96
0.99
2.00
2.01
1.00
1.02
1.03
0.98
1.00
3.05
3.05

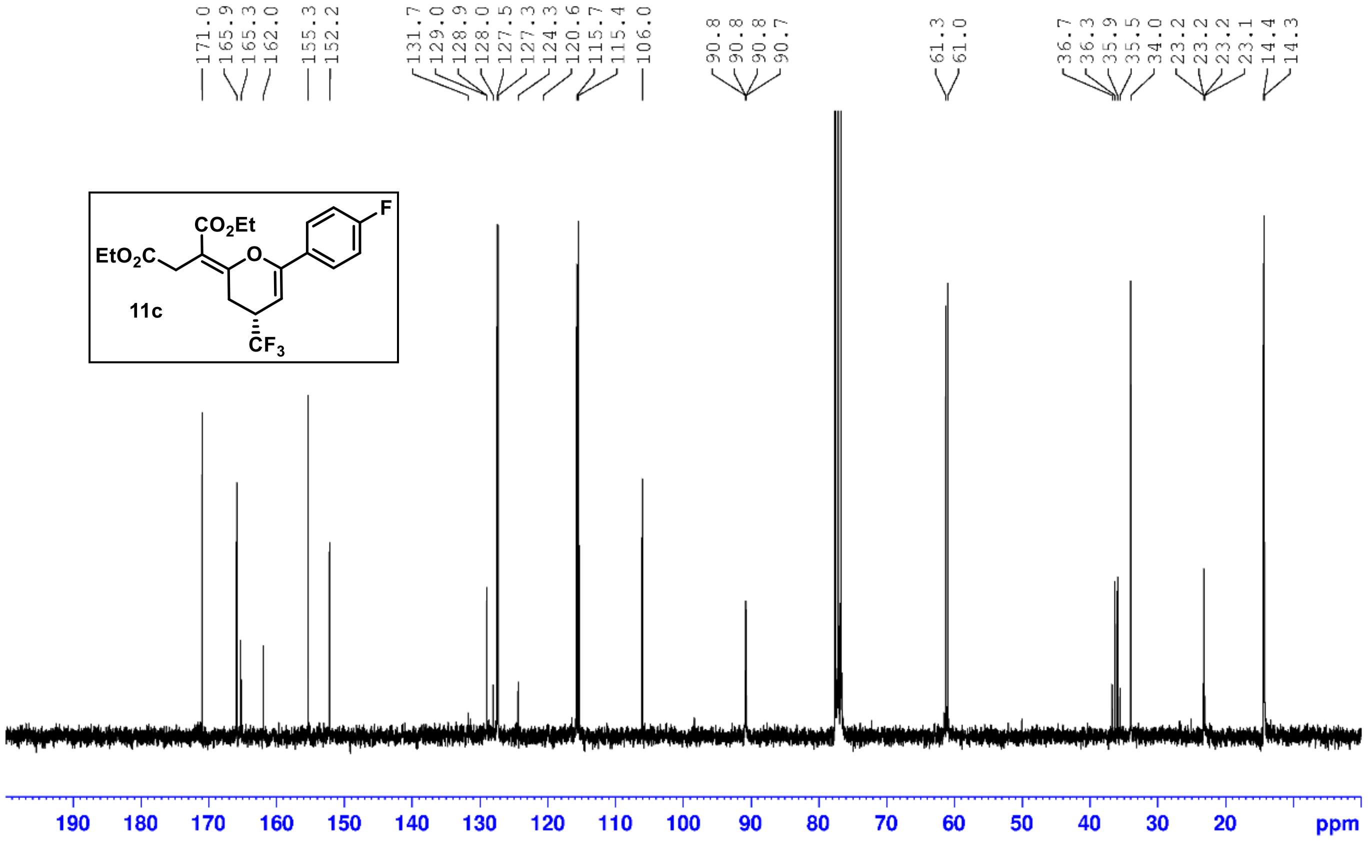
171.0
165.9
165.3
162.0
155.3
152.2
131.7
129.0
128.9
128.0
127.5
127.3
124.3
120.6
115.7
115.4
106.0
90.8
90.8
90.8
90.7
61.3
61.0
36.7
36.3
35.9
35.5
34.0
23.2
23.2
23.2
23.1
14.4
14.3
CO2Et
EtO2C
O
F
11c
CF3
190 180 170 160 150 140 130 120 110 100 90 80 70 60 50 40 30 20 ppm

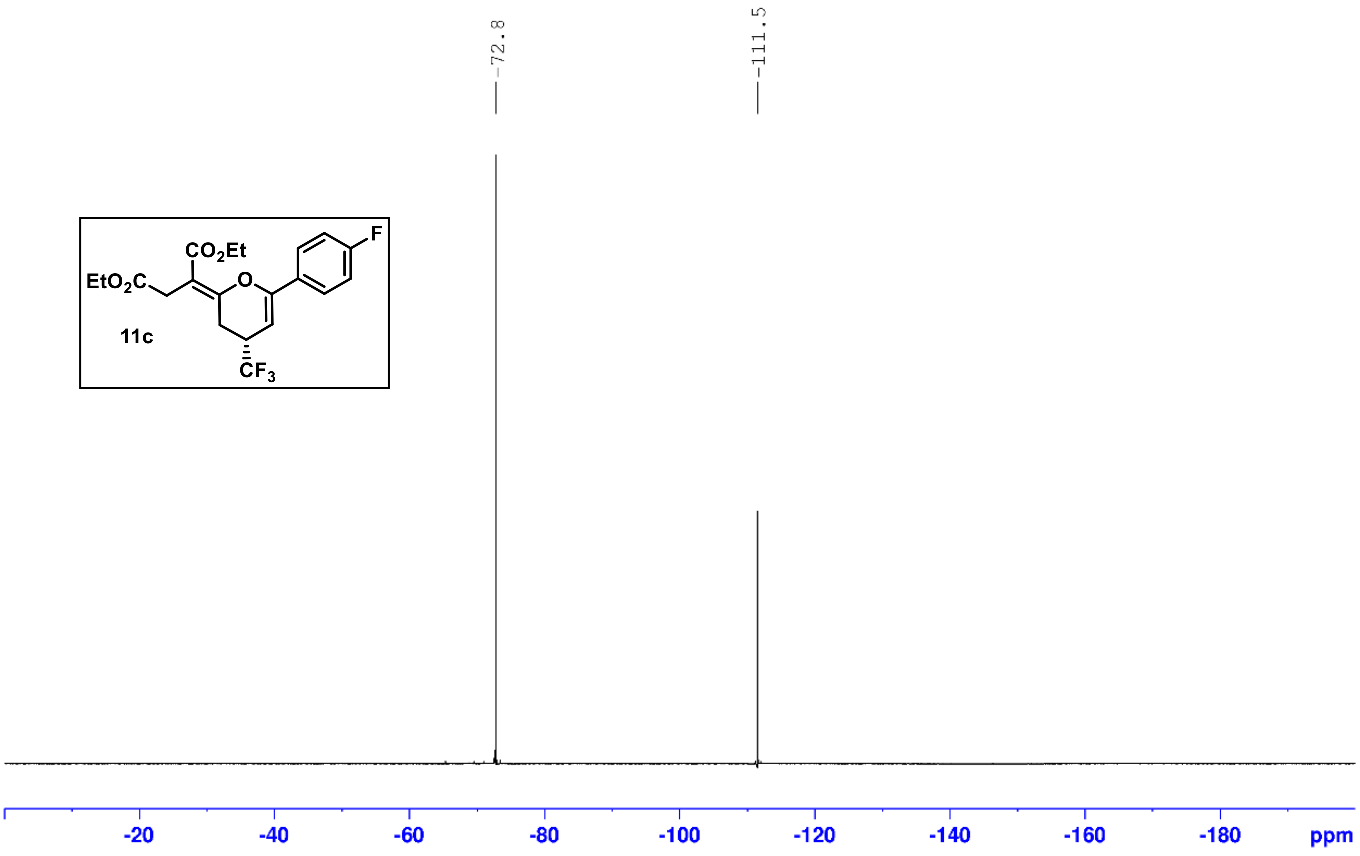
-72.8
-111.5
CO2Et
EtO2C
O
F
11c
CF3
-20 -40 -60 -80 -100 -120 -140 -160 -180 ppm

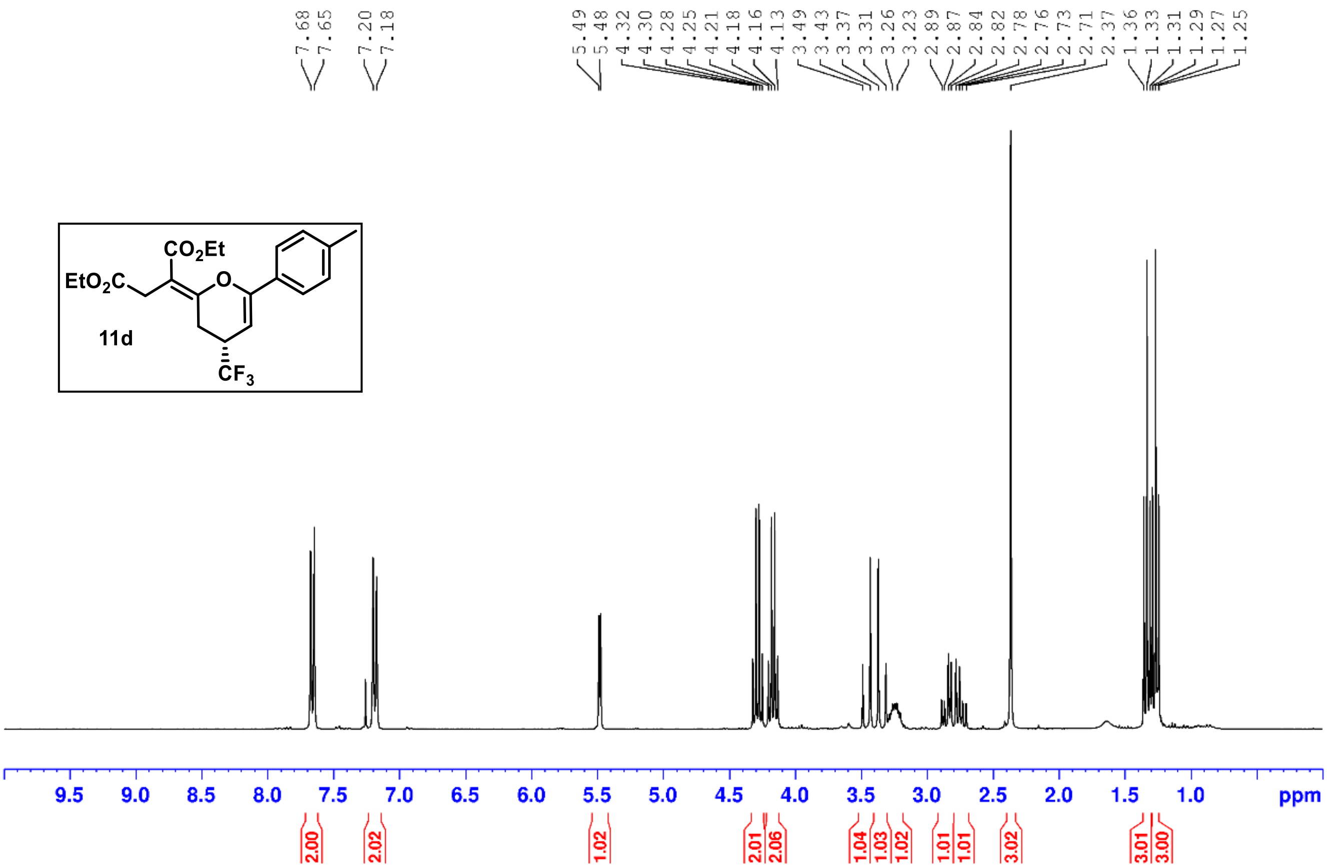
7.68 7.65 7.20 7.18 5.49 5.48 4.32 4.30 4.28 4.25 4.21 4.18 4.16 4.13 3.49 3.43 3.37 3.31 3.26 3.23 2.89 2.87 2.84 2.82 2.78 2.76 2.73 2.71 2.37 1.36 1.33 1.31 1.29 1.27 1.25
CO2Et
EtO2C
O
11d
CF3
9.5 9.0 8.5 8.0 7.5 7.0 6.5 6.0 5.5 5.0 4.5 4.0 3.5 3.0 2.5 2.0 1.5 1.0 ppm
2.00 2.02 1.02 2.01 2.06 1.04 1.03 1.02 1.01 1.01 3.02 3.01 3.00

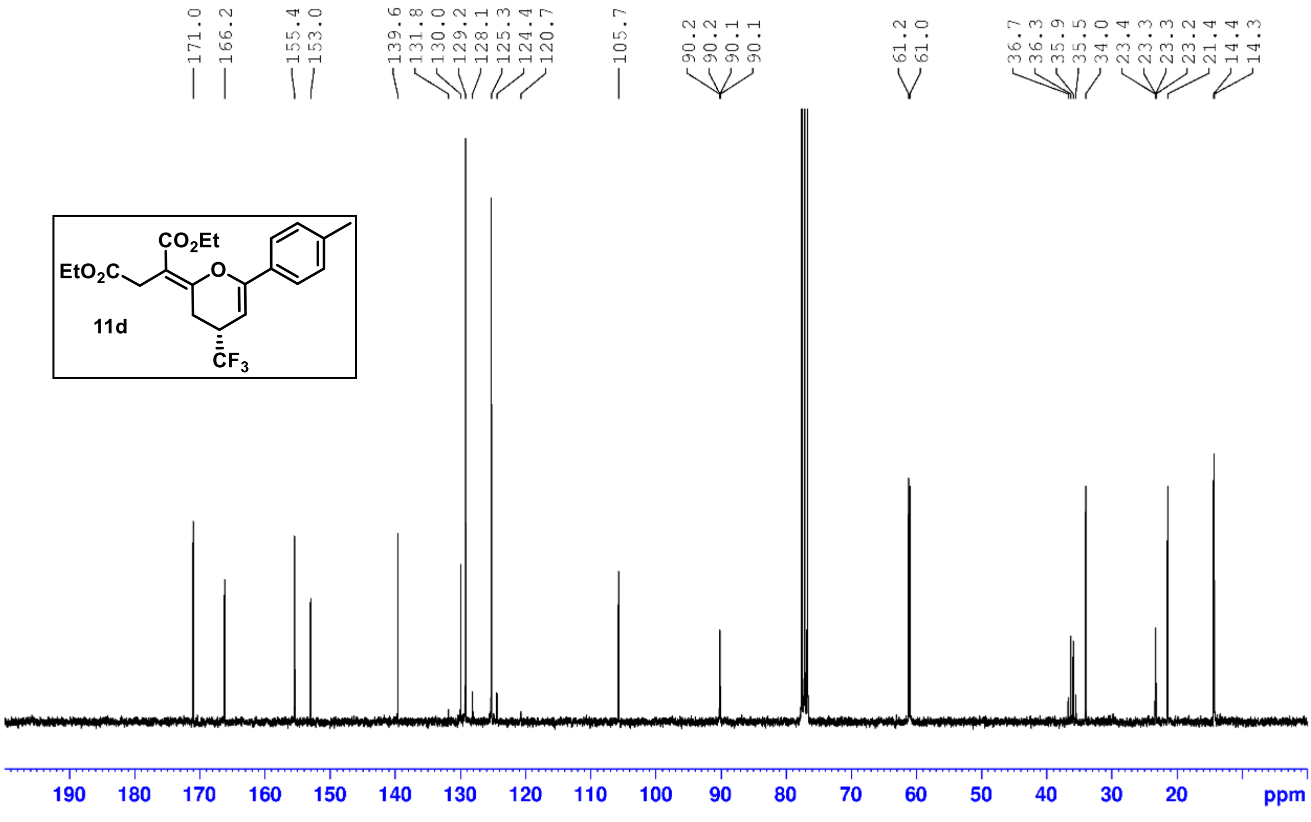
171.0 166.2 155.4 153.0 139.6 131.8 130.0 129.2 128.1 125.3 124.4 120.7 105.7 90.2 90.2 90.1 90.1 61.2 61.0 36.7 36.3 35.9 35.5 34.0 23.4 23.3 23.3 23.2 21.4 14.4 14.3
CO2Et
EtO2C
O
11d
CF3
190 180 170 160 150 140 130 120 110 100 90 80 70 60 50 40 30 20 ppm

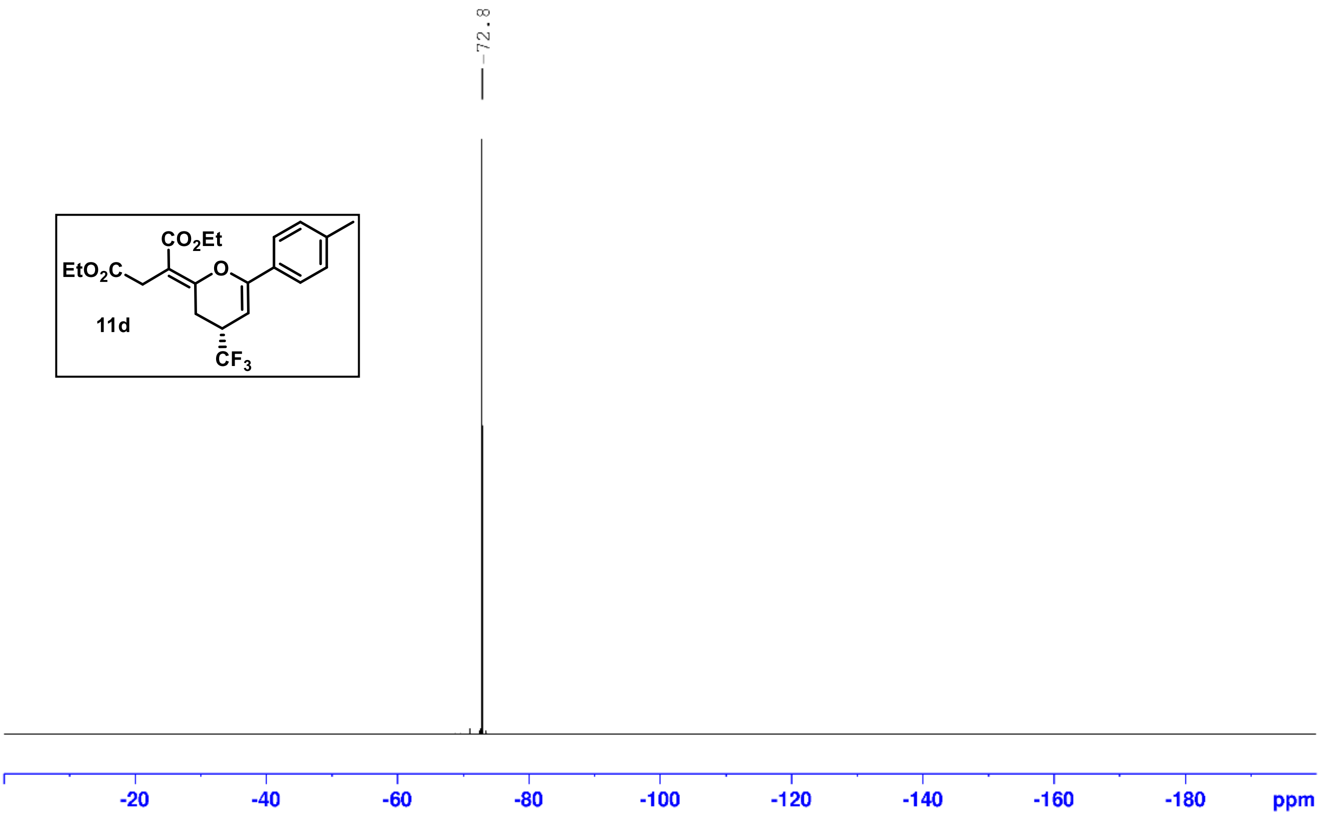
-72.8
EtO2C
CO2Et
O
11d
CF3
-20
-40
-60
-80
-100
-120
-140
-160
-180
ppm

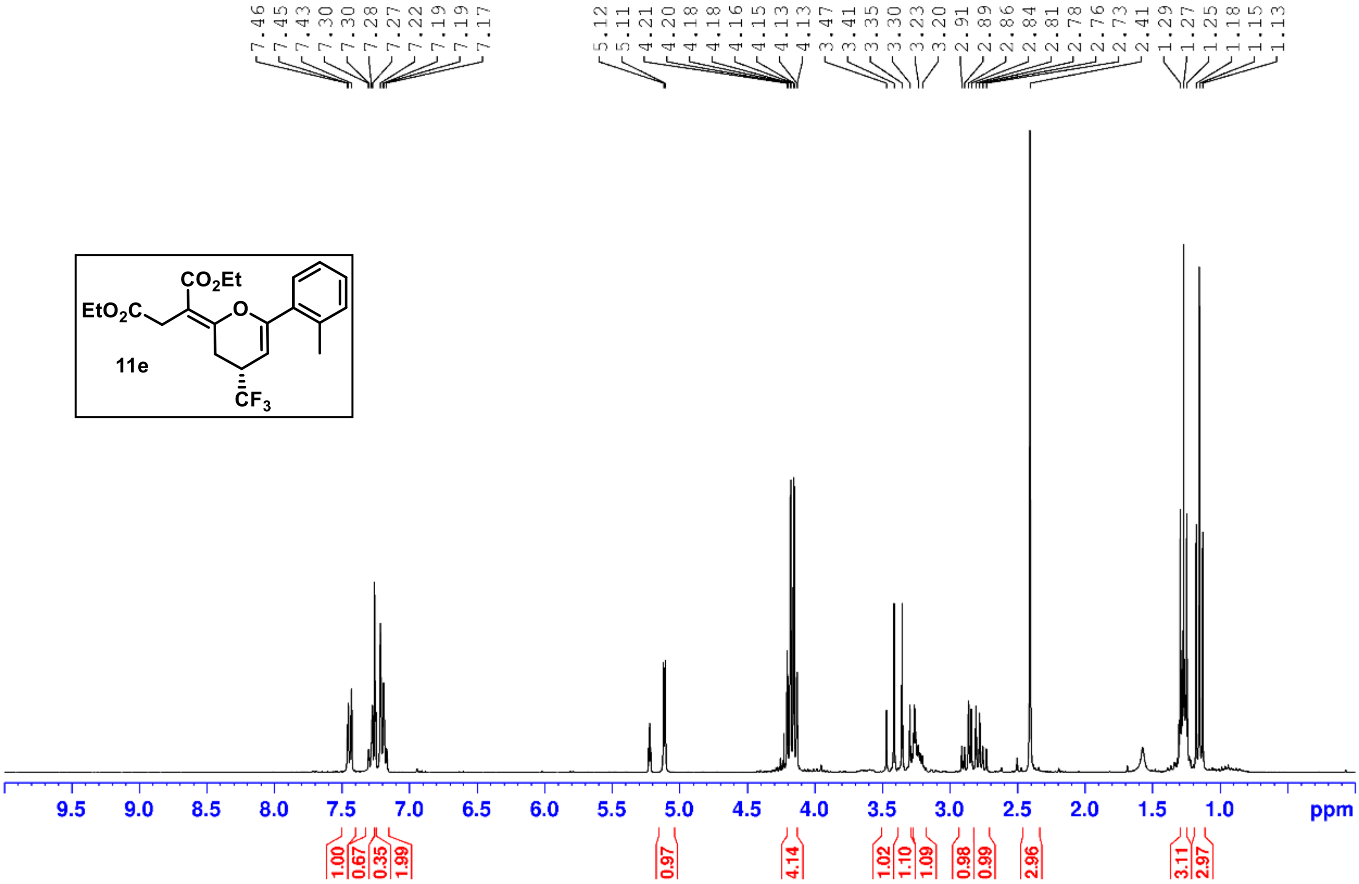
EtO2C
CO2Et
O
11e
CF3
9.5
9.0
8.5
8.0
7.5
7.0
6.5
6.0
5.5
5.0
4.5
4.0
3.5
3.0
2.5
2.0
1.5
1.0
ppm

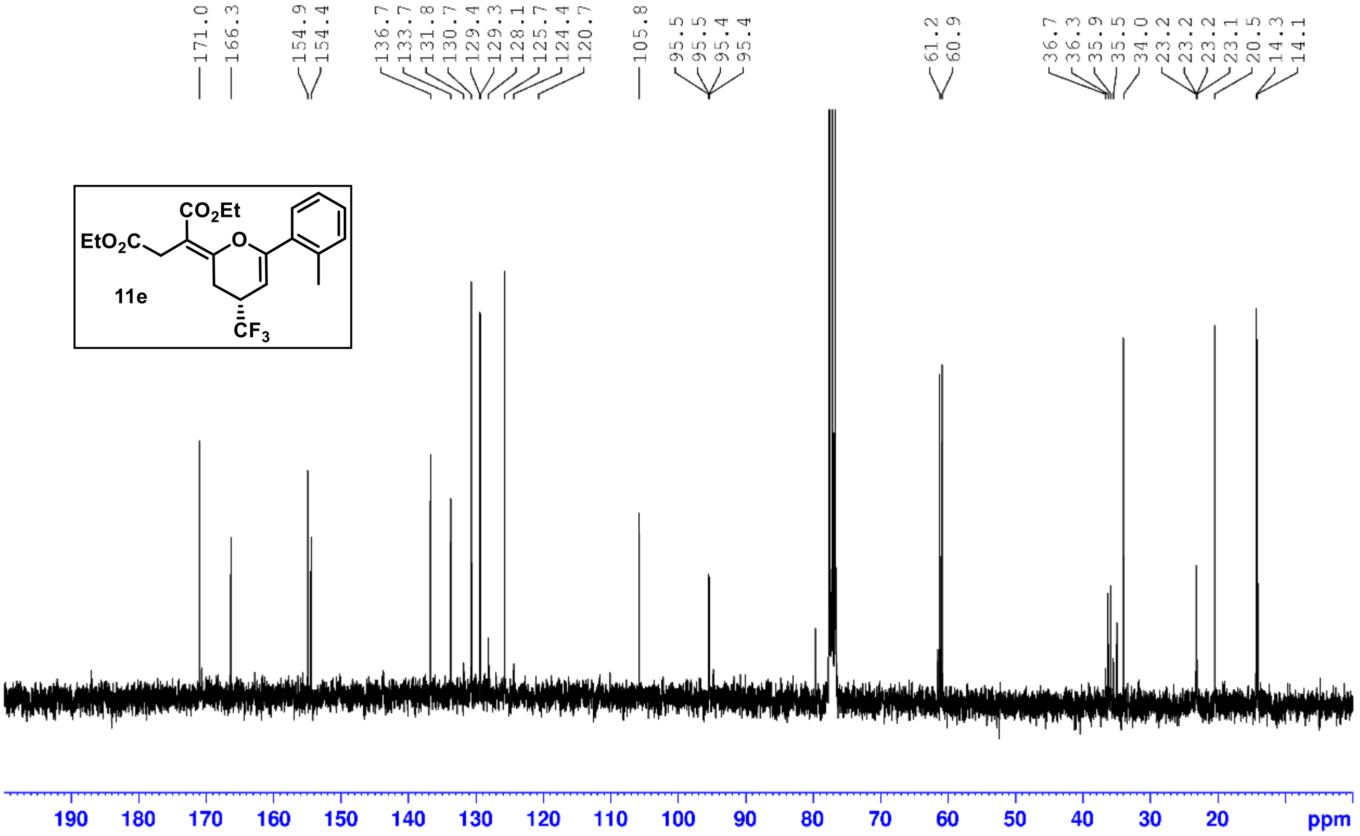
171.0
166.3
154.9
154.4
136.7
133.7
131.8
130.7
129.4
129.3
128.1
125.7
124.4
120.7
105.8
95.5
95.5
95.4
95.4
61.2
60.9
36.7
36.3
35.9
35.5
34.0
23.2
23.2
23.2
23.1
20.5
14.3
14.1
CO2Et
EtO2C
O
11e
CF3
190 180 170 160 150 140 130 120 110 100 90 80 70 60 50 40 30 20 ppm

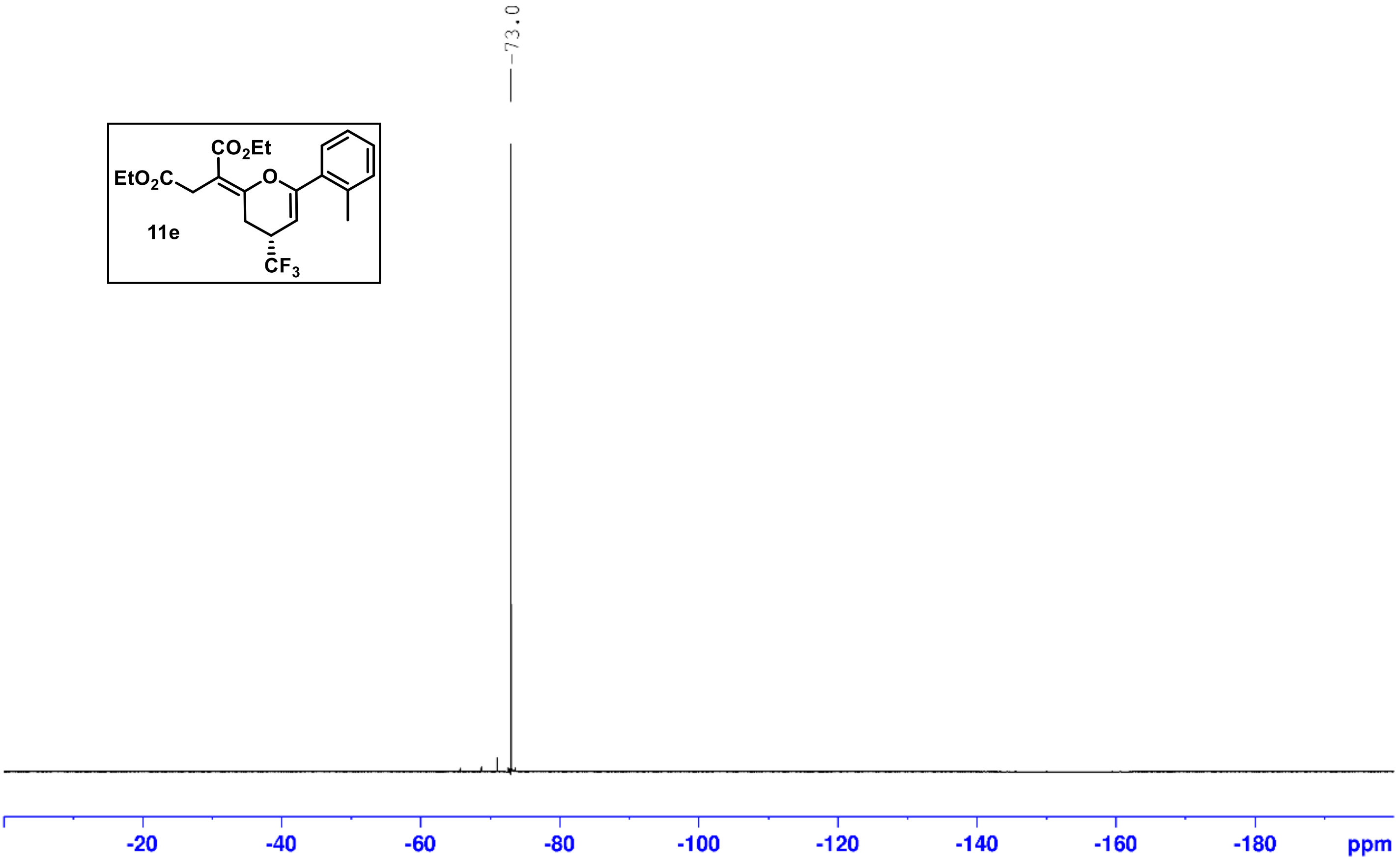
-73.0
CO2Et
EtO2C
O
11e
CF3
-20 -40 -60 -80 -100 -120 -140 -160 -180 ppm

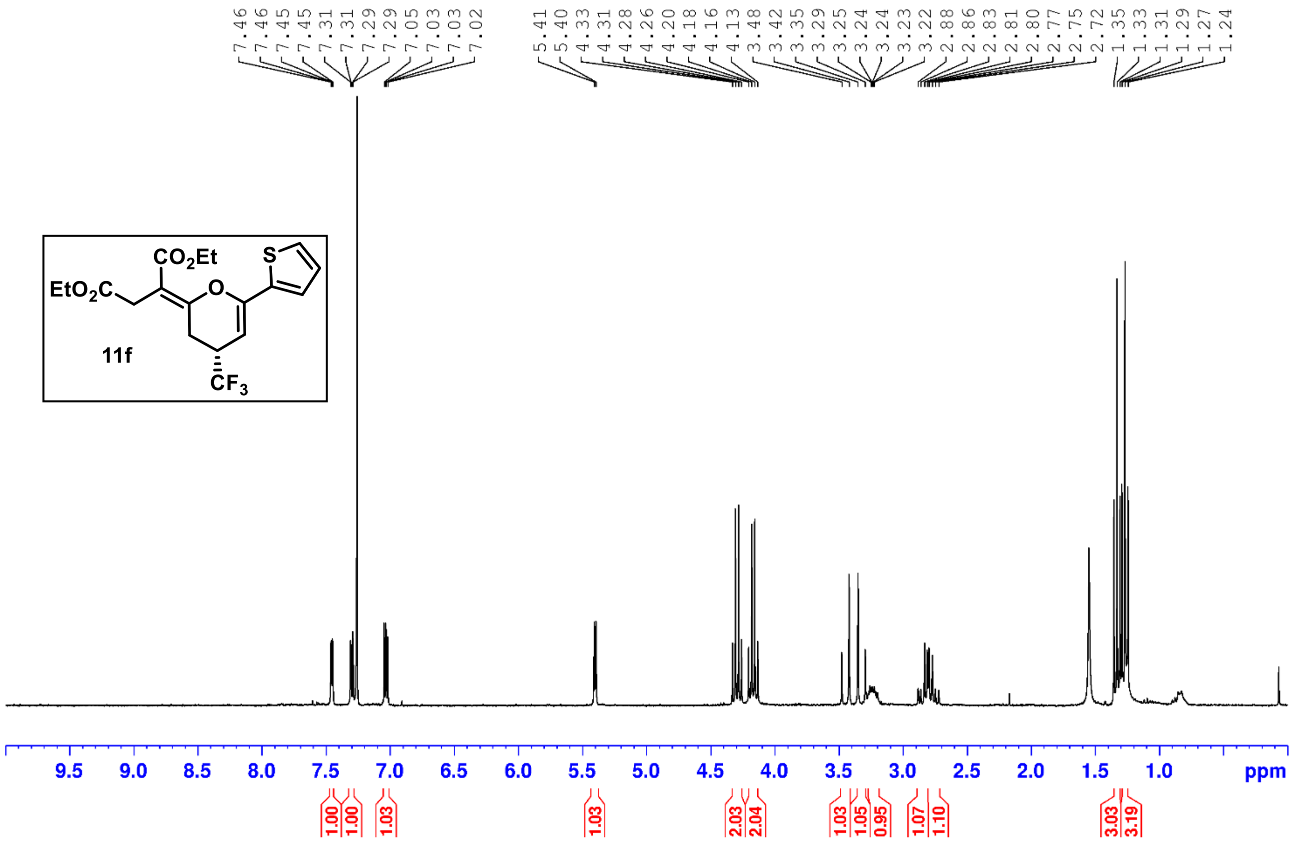

7.46 7.46 7.45 7.45 7.31 7.31 7.29 7.29 7.05 7.03 7.03 7.02
5.41 5.40 4.33 4.31 4.28 4.26 4.20 4.18 4.16 4.13 3.48 3.42 3.35 3.29 3.25 3.24 3.24 3.23 3.22 2.88 2.86 2.83 2.81 2.80 2.77 2.75 2.72 1.35 1.33 1.31 1.29 1.27 1.24
CO2Et
EtO2C
O
S
CF3
11f
9.5 9.0 8.5 8.0 7.5 7.0 6.5 6.0 5.5 5.0 4.5 4.0 3.5 3.0 2.5 2.0 1.5 1.0 ppm
1.00 1.00 1.03 1.03 2.03 2.04 1.03 1.05 0.95 1.07 1.10 3.03 3.19

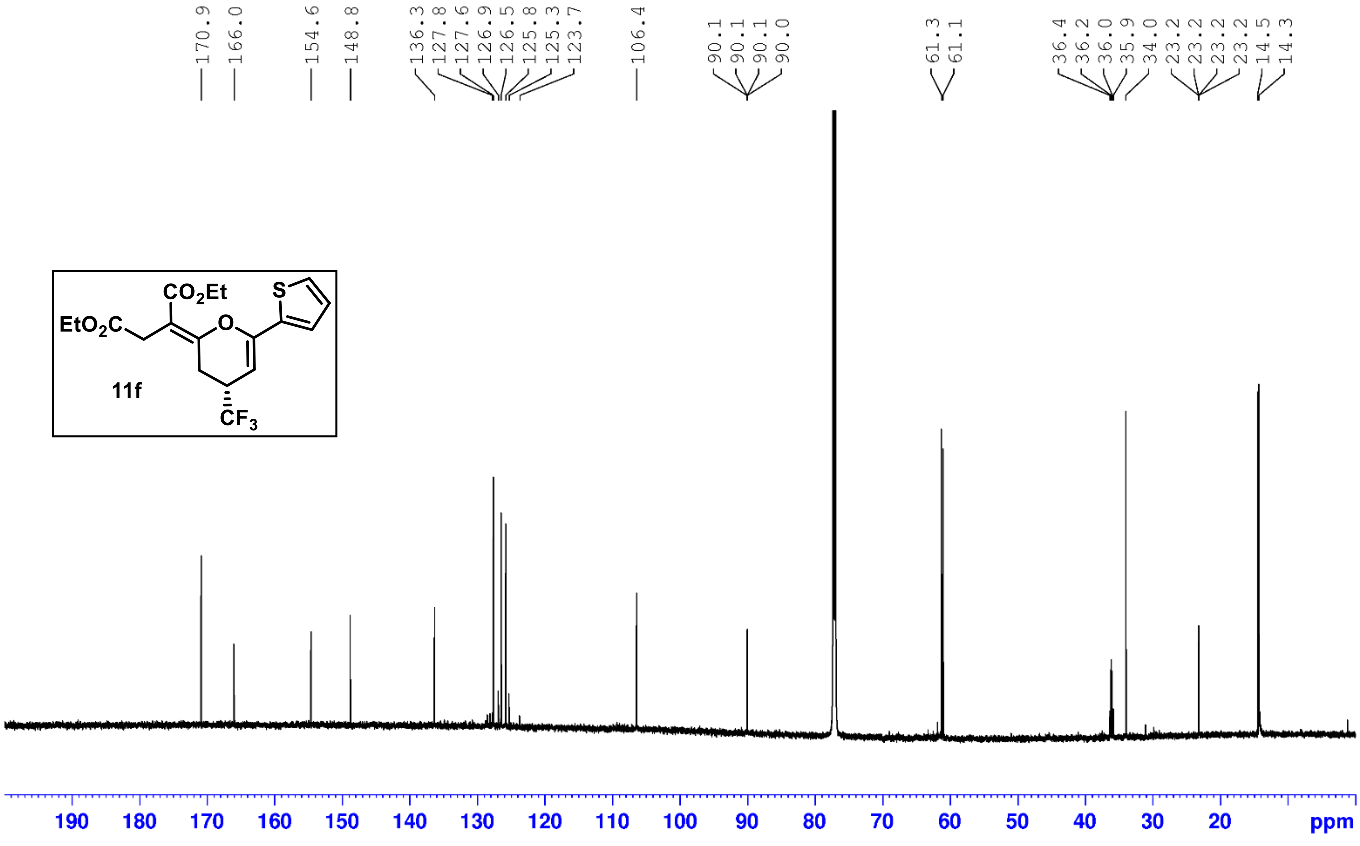

170.9 166.0 154.6 148.8 136.3 127.8 127.6 126.9 126.5 125.8 125.3 123.7 106.4 90.1 90.1 90.1 90.0 61.3 61.1 36.4 36.2 36.0 35.9 34.0 23.2 23.2 23.2 23.2 14.5 14.3
CO2Et
EtO2C
O
S
CF3
11f
190 180 170 160 150 140 130 120 110 100 90 80 70 60 50 40 30 20 ppm

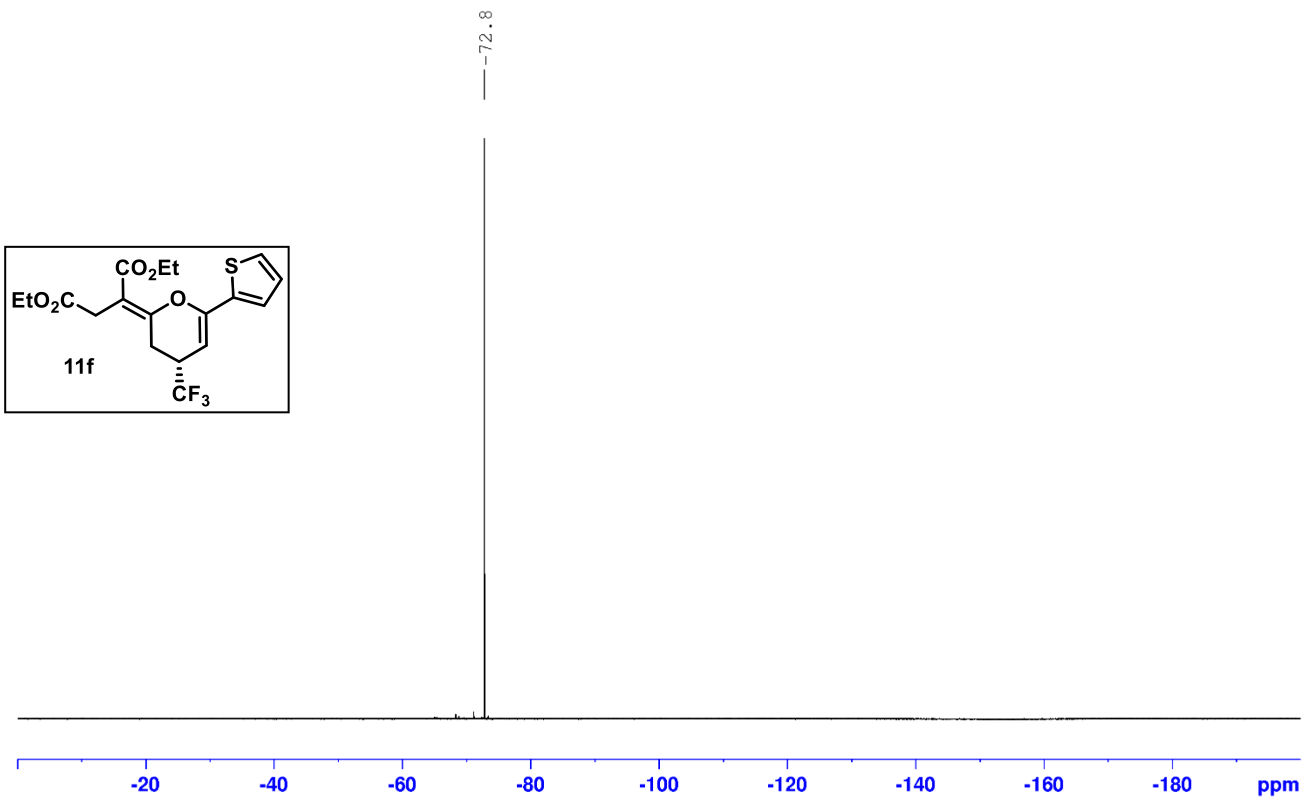
-72.8
CO2Et
EtO2C
O
S
11f
CF3
-20
-40
-60
-80
-100
-120
-140
-160
-180
ppm

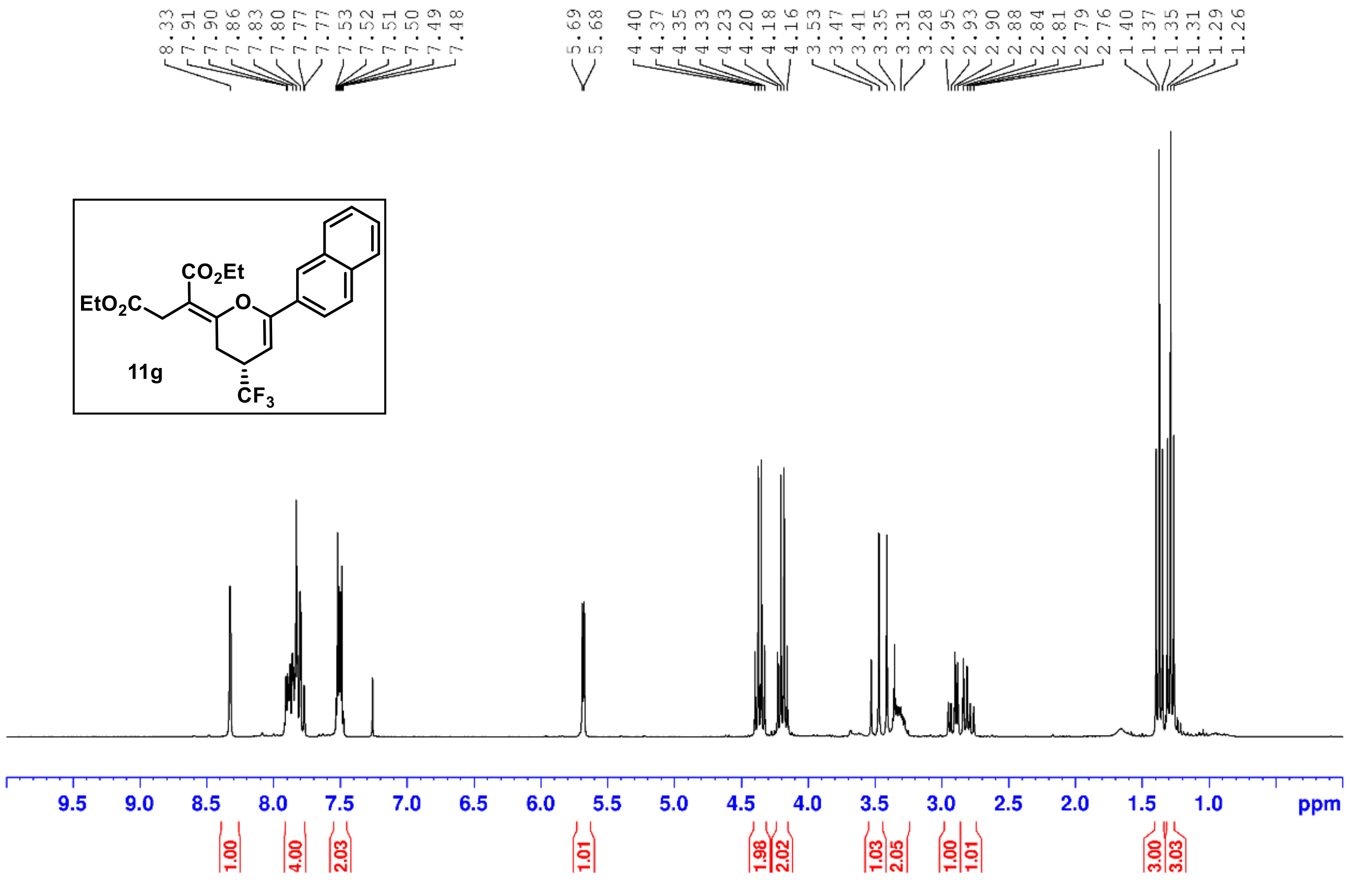
8.33
7.91
7.90
7.86
7.83
7.80
7.77
7.77
7.53
7.52
7.51
7.50
7.49
7.48
5.69
5.68
4.40
4.37
4.35
4.33
4.23
4.20
4.18
4.16
3.53
3.47
3.41
3.35
3.31
3.28
2.95
2.93
2.90
2.88
2.84
2.81
2.79
2.76
1.40
1.37
1.35
1.31
1.29
1.26
CO2Et
EtO2C
O
11g
CF3
9.5
9.0
8.5
8.0
7.5
7.0
6.5
6.0
5.5
5.0
4.5
4.0
3.5
3.0
2.5
2.0
1.5
1.0
ppm
1.00
4.00
2.03
1.01
1.98
2.02
1.03
2.05
1.00
1.01
3.00
3.03

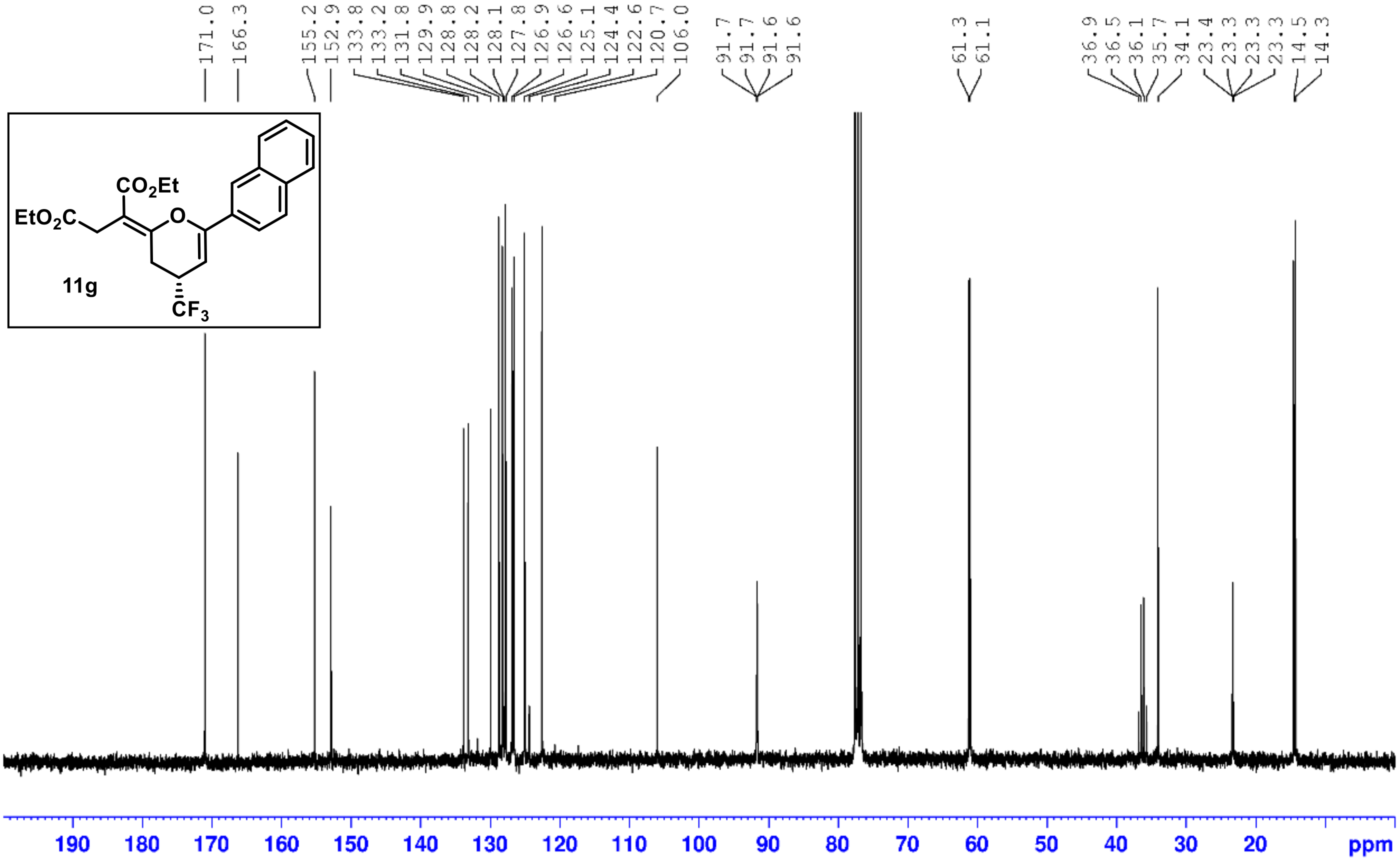
171.0
166.3
155.2
152.9
133.8
133.2
131.8
129.9
128.8
128.2
128.1
127.8
126.9
126.6
125.1
124.4
122.6
120.7
106.0
91.7
91.7
91.6
91.6
61.3
61.1
36.9
36.5
36.1
35.7
34.1
23.4
23.3
23.3
23.3
14.5
14.3
CO2Et
EtO2C
O
11g
CF3
190
180
170
160
150
140
130
120
110
100
90
80
70
60
50
40
30
20
ppm

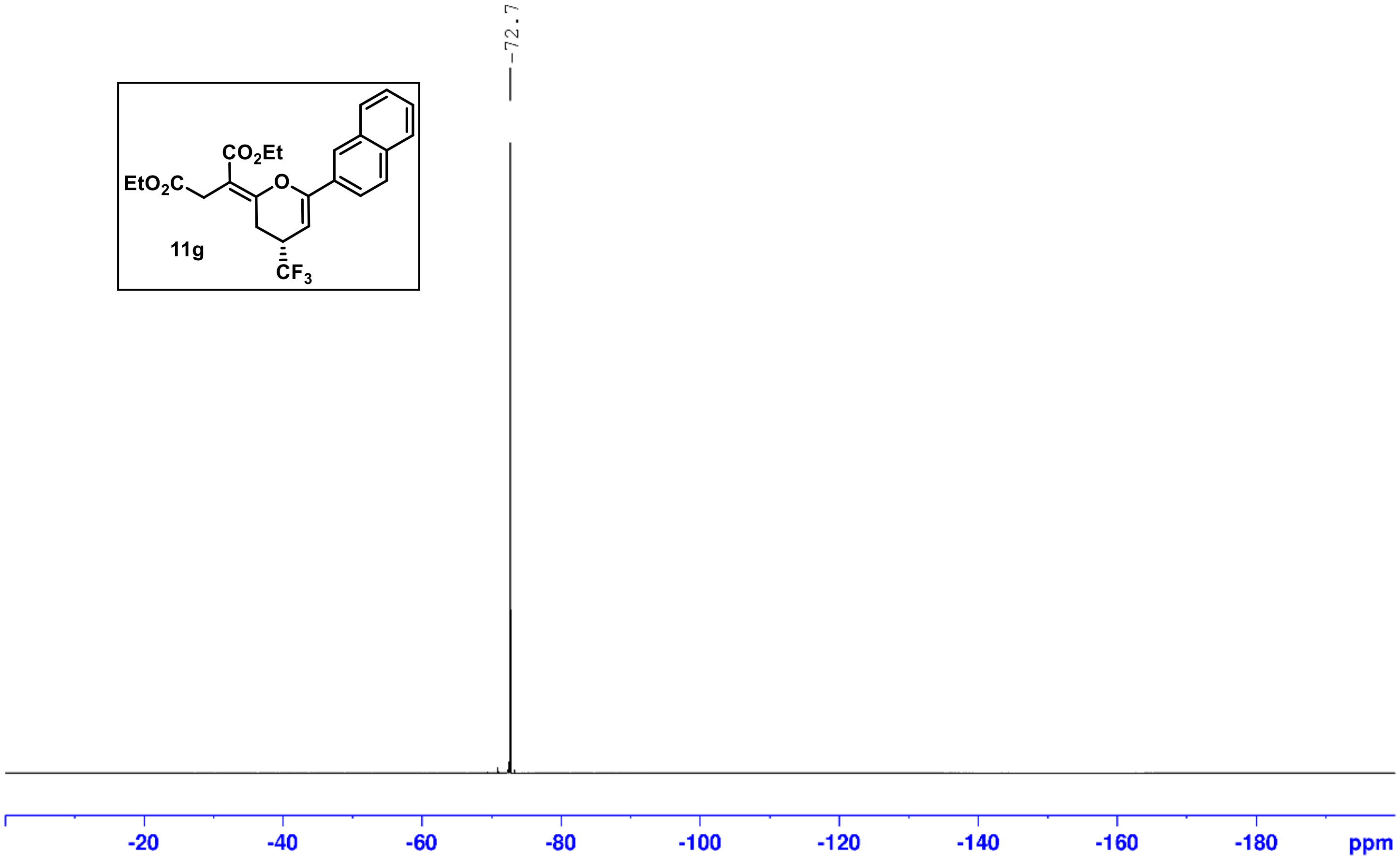
-72.7
CO2Et
EtO2C
O
11g
CF3
-20
-40
-60
-80
-100
-120
-140
-160
-180
ppm

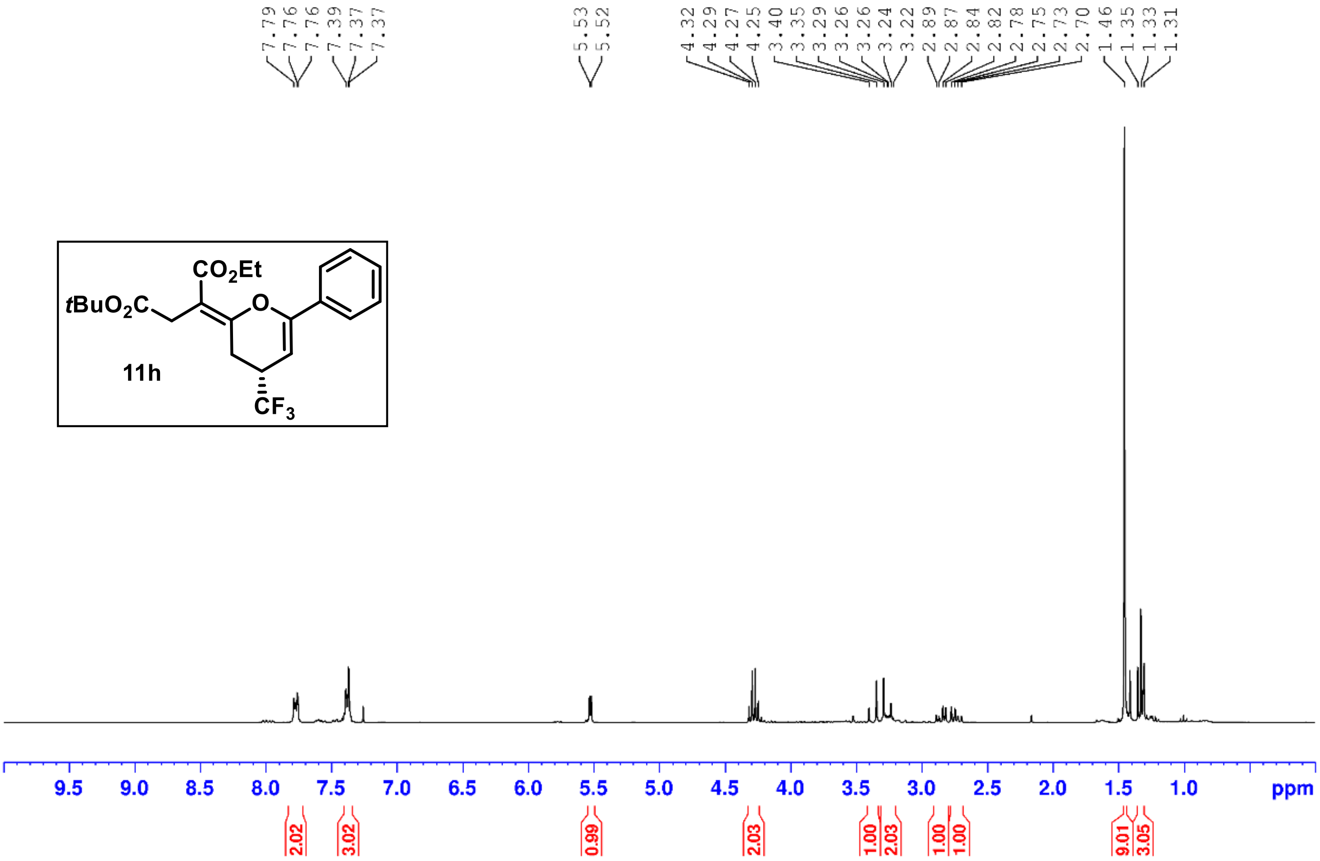
7.79
7.76
7.76
7.39
7.37
7.37
5.53
5.52
4.32
4.29
4.27
4.25
3.40
3.35
3.29
3.26
3.26
3.24
3.22
2.89
2.87
2.84
2.82
2.78
2.75
2.73
2.70
1.46
1.35
1.33
1.31
CO2Et
tBuO2C
O
11h
CF3
9.5
9.0
8.5
8.0
7.5
7.0
6.5
6.0
5.5
5.0
4.5
4.0
3.5
3.0
2.5
2.0
1.5
1.0
ppm
2.02
3.02
0.99
2.03
1.00
2.03
1.00
1.00
9.01
3.05

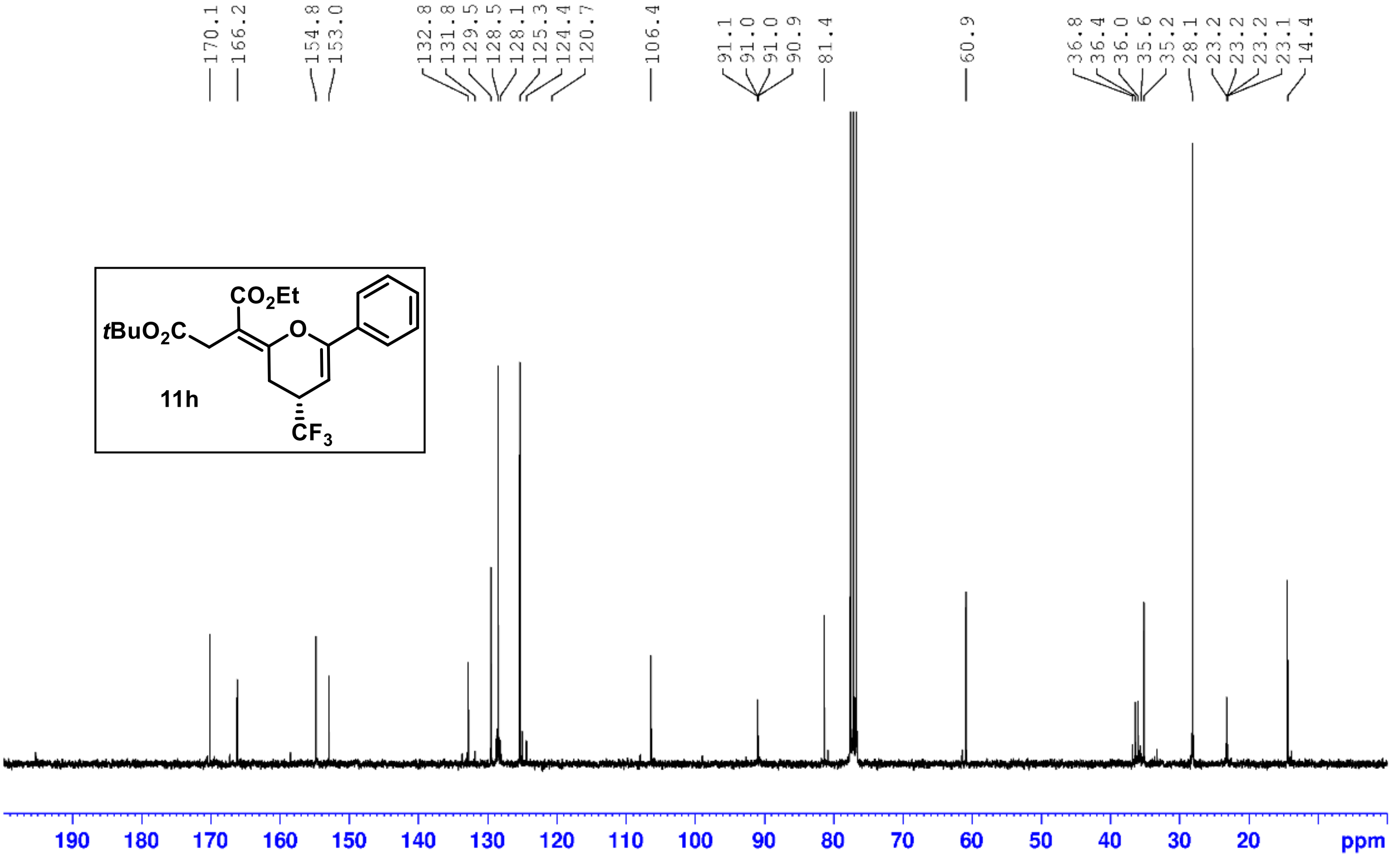
170.1
166.2
154.8
153.0
132.8
131.8
129.5
128.5
128.1
125.3
124.4
120.7
106.4
91.1
91.0
91.0
90.9
81.4
60.9
36.8
36.4
36.0
35.6
35.2
28.1
23.2
23.2
23.2
23.1
14.4
CO2Et
tBuO2C
O
11h
CF3
190
180
170
160
150
140
130
120
110
100
90
80
70
60
50
40
30
20
ppm

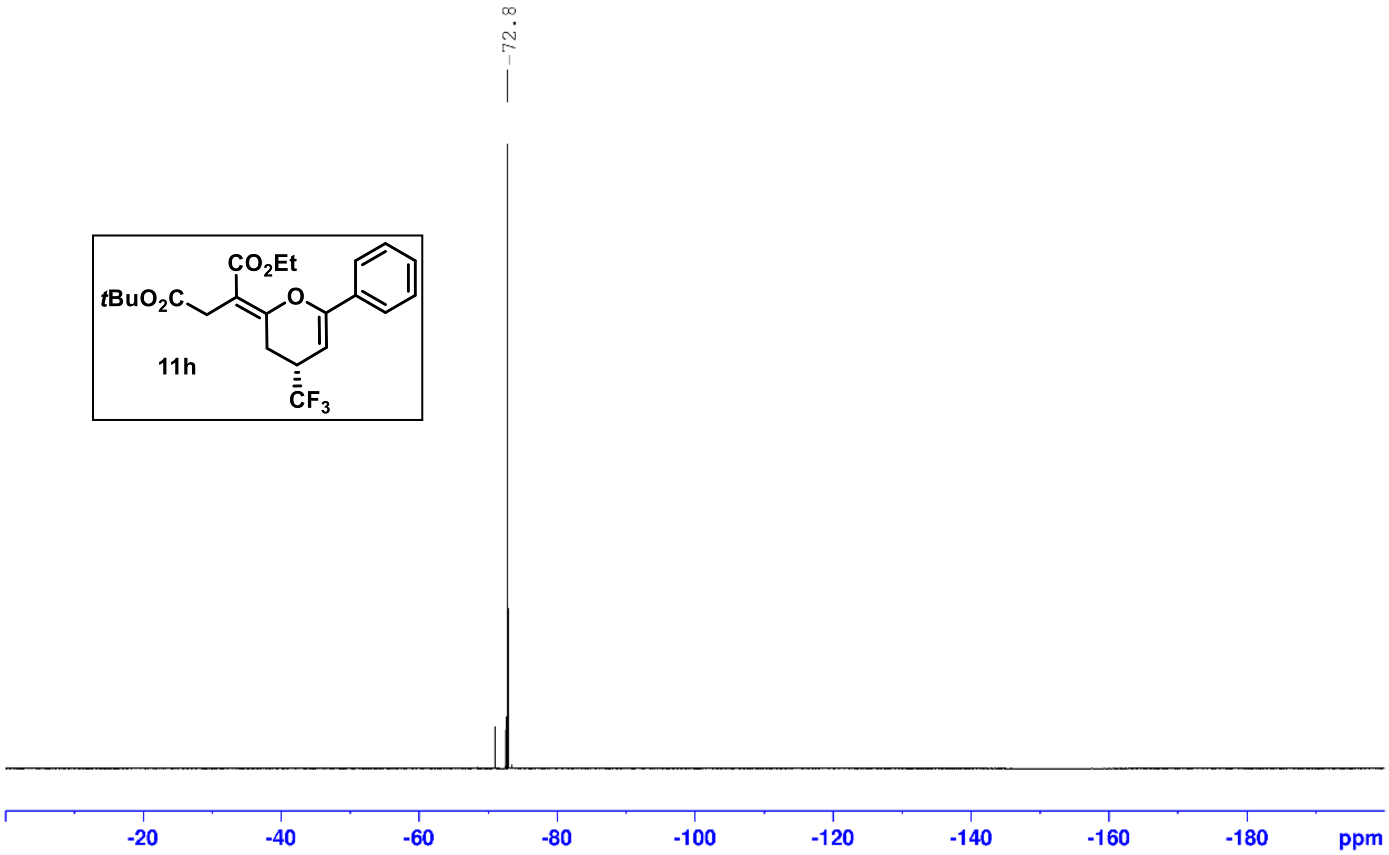

-72.8
CO2Et
tBuO2C
O
11h
CF3
-20 -40 -60 -80 -100 -120 -140 -160 -180 ppm

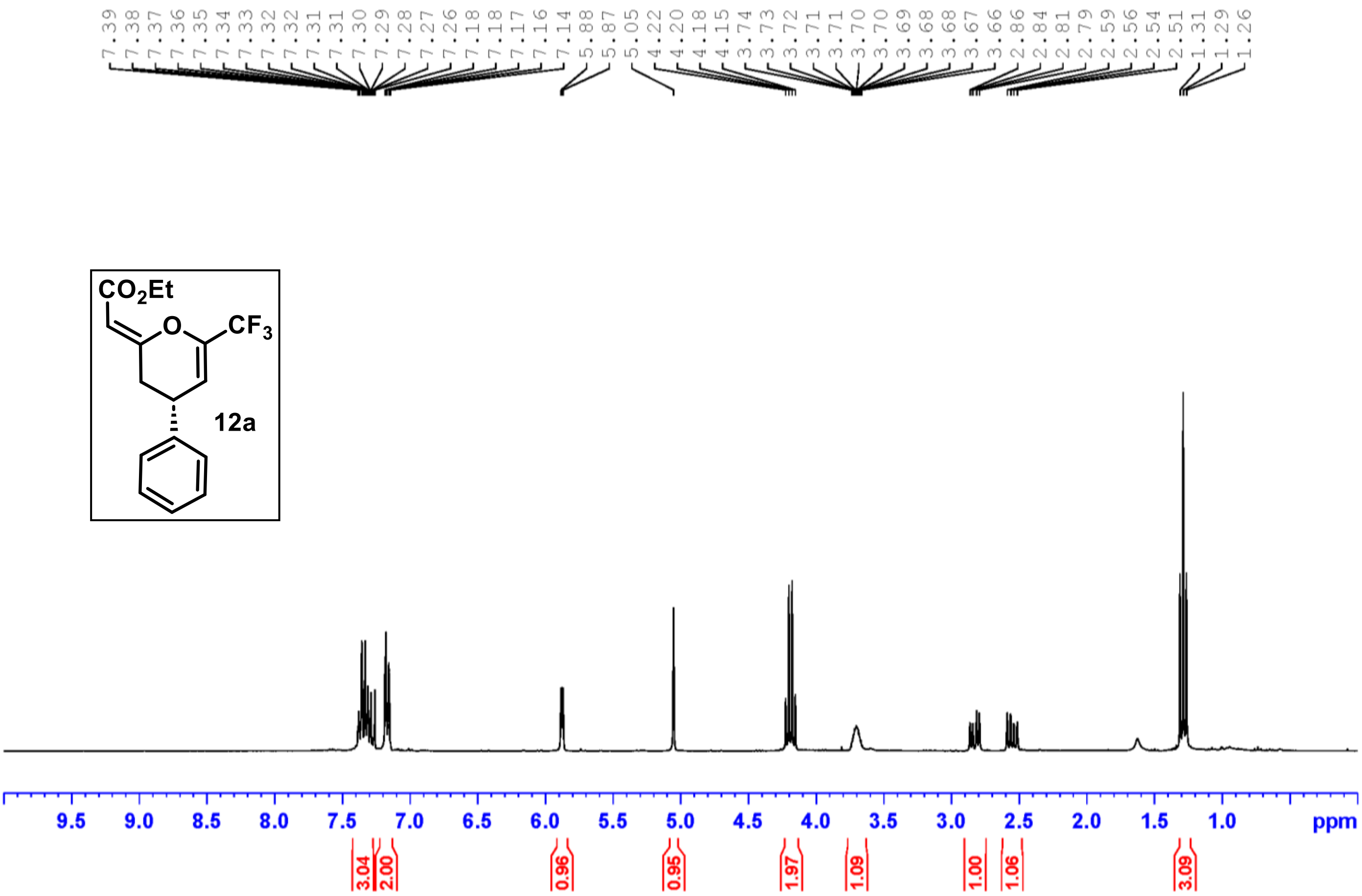

7.39 7.38 7.37 7.36 7.35 7.34 7.33 7.32 7.32 7.31 7.31 7.30 7.29 7.28 7.27 7.26 7.18 7.18 7.17 7.16 7.14 5.88 5.87 5.05 4.22 4.20 4.18 4.15 3.74 3.73 3.72 3.71 3.71 3.70 3.70 3.69 3.68 3.68 3.67 3.66 2.86 2.84 2.81 2.79 2.59 2.56 2.54 2.51 1.31 1.29 1.26
CO2Et
O
CF3
12a
9.5 9.0 8.5 8.0 7.5 7.0 6.5 6.0 5.5 5.0 4.5 4.0 3.5 3.0 2.5 2.0 1.5 1.0 ppm
3.04 2.00 0.96 0.95 1.97 1.09 1.00 1.06 3.09

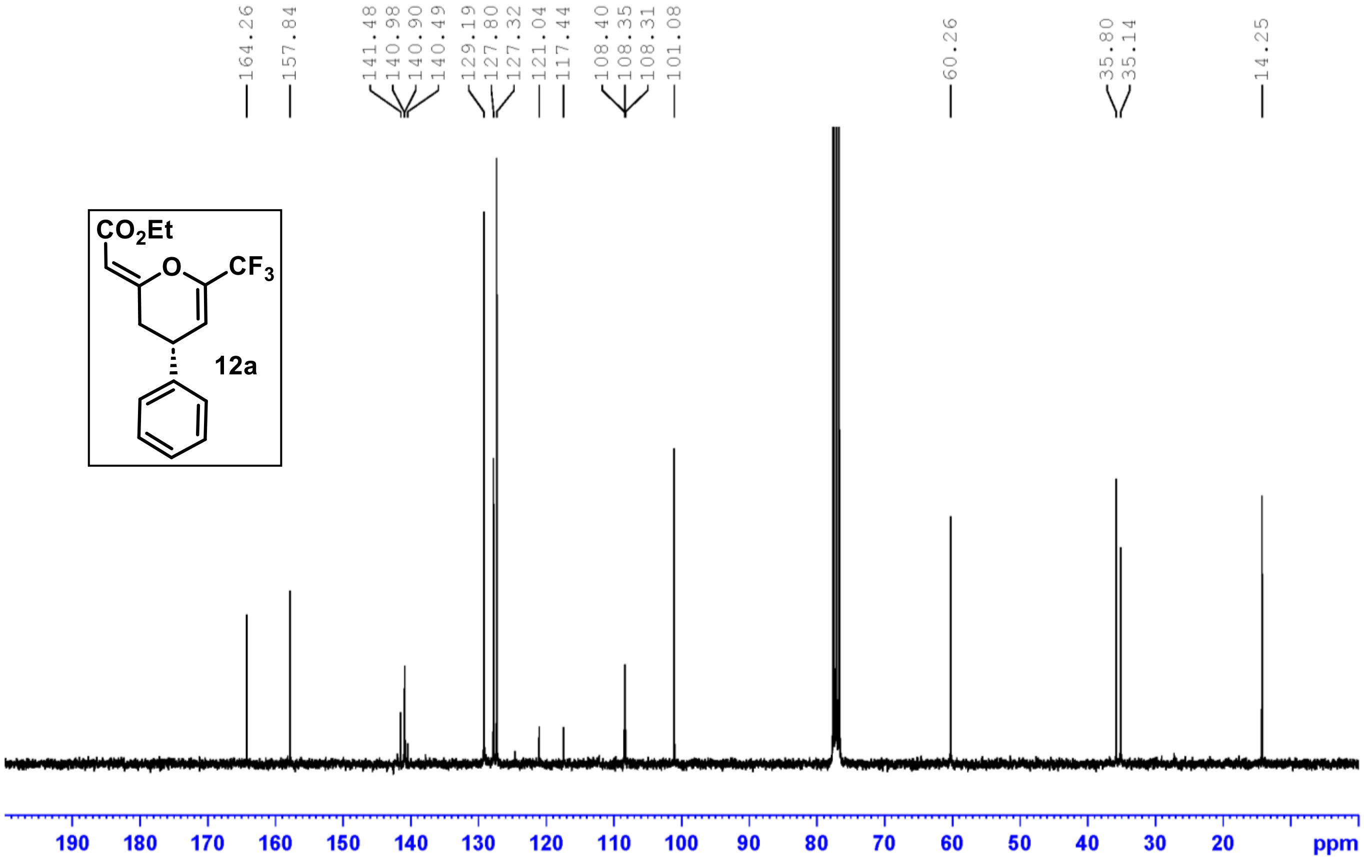
164.26
157.84
141.48
140.98
140.90
140.49
129.19
127.80
127.32
121.04
117.44
108.40
108.35
108.31
101.08
60.26
35.80
35.14
14.25
CO2Et
O
CF3
12a
190 180 170 160 150 140 130 120 110 100 90 80 70 60 50 40 30 20 ppm

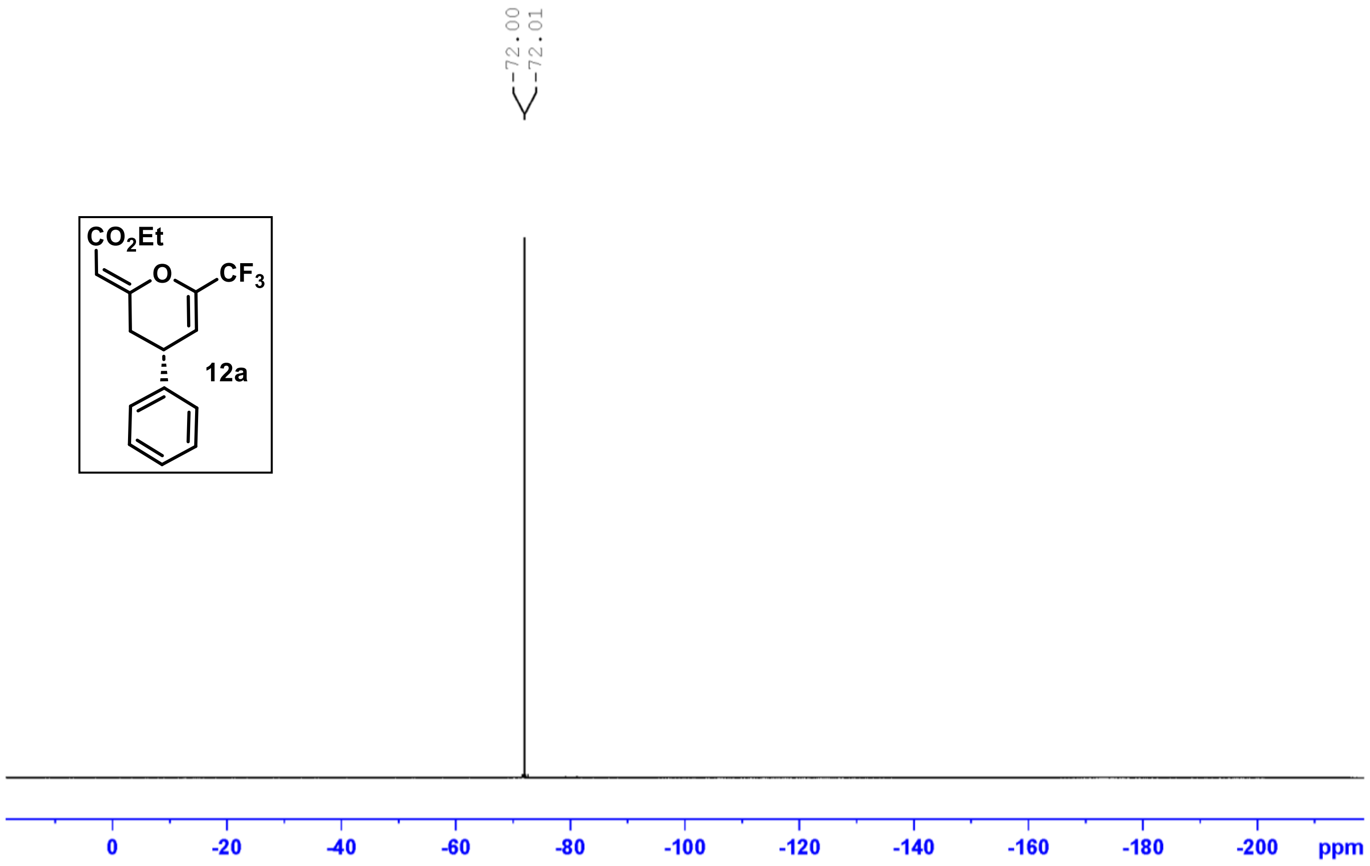
-72.00
-72.01
CO2Et
O
CF3
12a
0 -20 -40 -60 -80 -100 -120 -140 -160 -180 -200 ppm

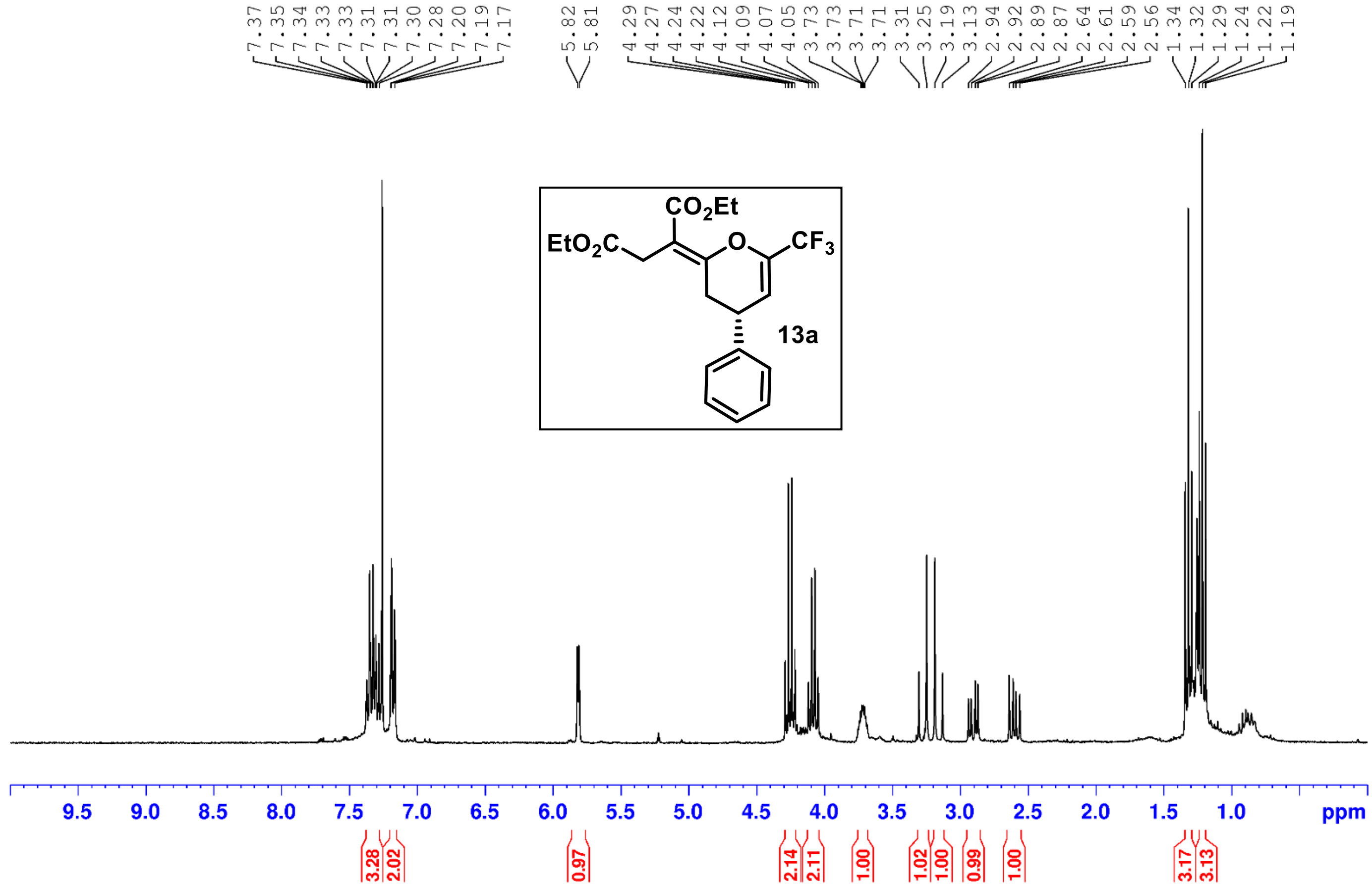
7.37 7.35 7.34 7.33 7.33 7.31 7.31 7.30 7.28 7.20 7.19 7.17
5.82 5.81
4.29 4.27 4.24 4.22 4.12 4.09 4.07 4.05 3.73 3.73 3.71 3.71 3.31 3.25 3.19 3.13 2.94 2.92 2.89 2.87 2.64 2.61 2.59 2.56 1.34 1.32 1.29 1.24 1.22 1.19
CO2Et
EtO2C
O
CF3
13a
9.5 9.0 8.5 8.0 7.5 7.0 6.5 6.0 5.5 5.0 4.5 4.0 3.5 3.0 2.5 2.0 1.5 1.0 ppm
3.28 2.02 0.97 2.14 2.11 1.00 1.02 1.00 0.99 1.00 3.17 3.13

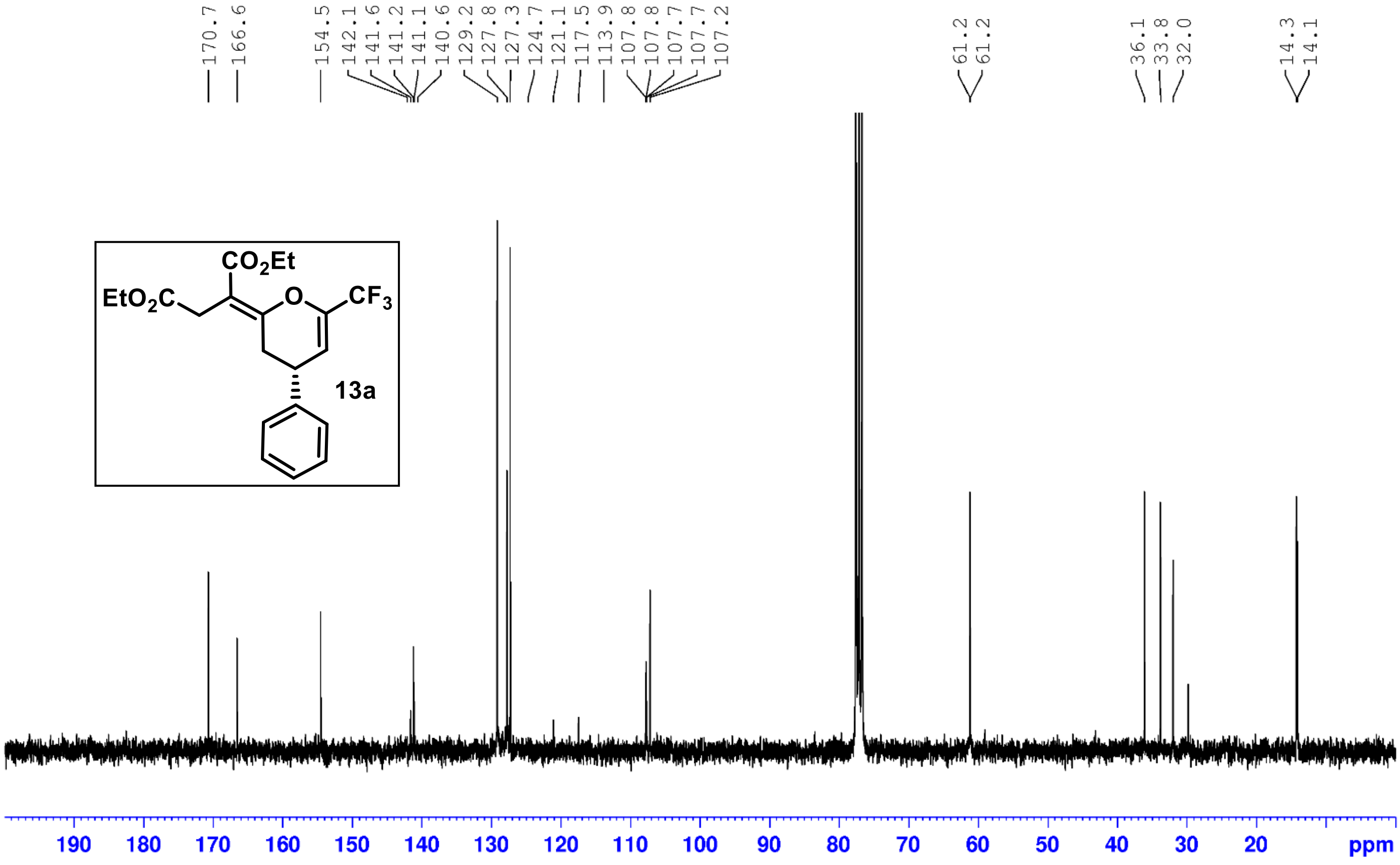
170.7 166.6 154.5 142.1 141.6 141.2 141.1 140.6 129.2 127.8 127.3 124.7 121.1 117.5 113.9 107.8 107.8 107.7 107.7 107.2
61.2 61.2
36.1 33.8 32.0
14.3 14.1
CO2Et
EtO2C
O
CF3
13a
190 180 170 160 150 140 130 120 110 100 90 80 70 60 50 40 30 20 ppm

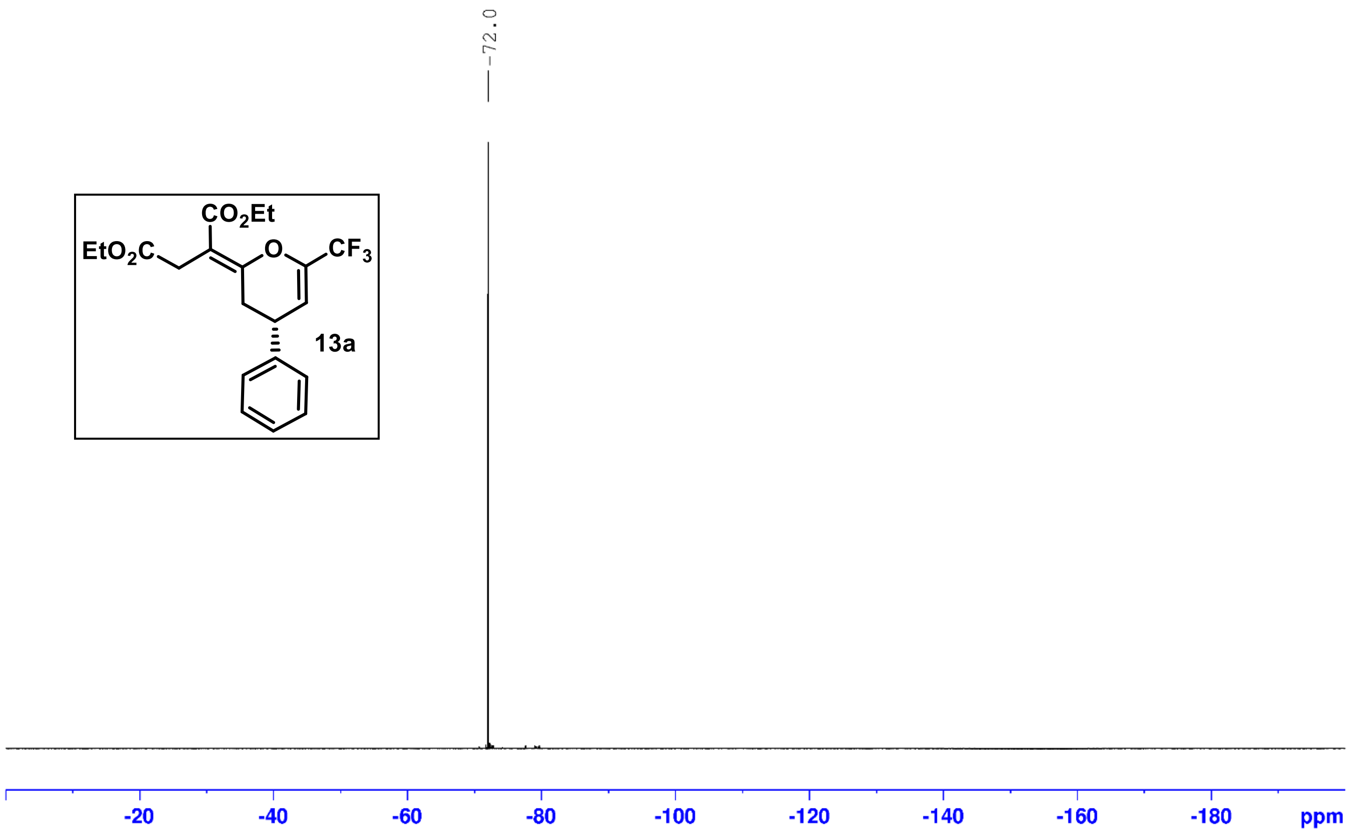
-72.0
CO2Et
EtO2C
O
CF3
13a
-20
-40
-60
-80
-100
-120
-140
-160
-180
ppm

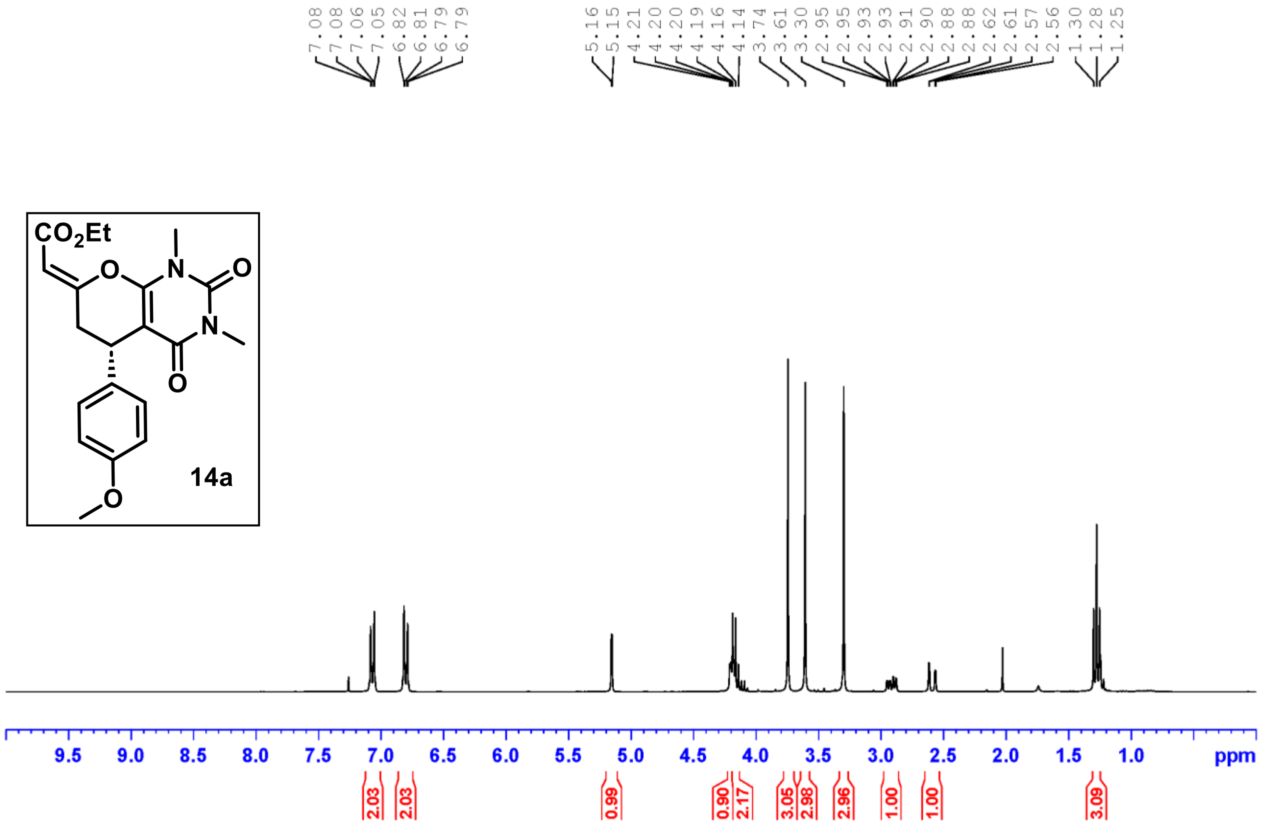
7.08 7.08 7.06 7.05 6.82 6.81 6.79 6.79
5.16 5.15 4.21 4.20 4.20 4.19 4.16 4.14 3.74 3.61 3.30 2.95 2.95 2.93 2.93 2.91 2.90 2.88 2.88 2.62 2.61 2.57 2.56 1.30 1.28 1.25
CO2Et
O
N
O
N
O
O
14a
9.5
9.0
8.5
8.0
7.5
7.0
6.5
6.0
5.5
5.0
4.5
4.0
3.5
3.0
2.5
2.0
1.5
1.0
ppm
2.03
2.03
0.99
0.90
2.17
3.05
2.98
2.96
1.00
1.00
3.09

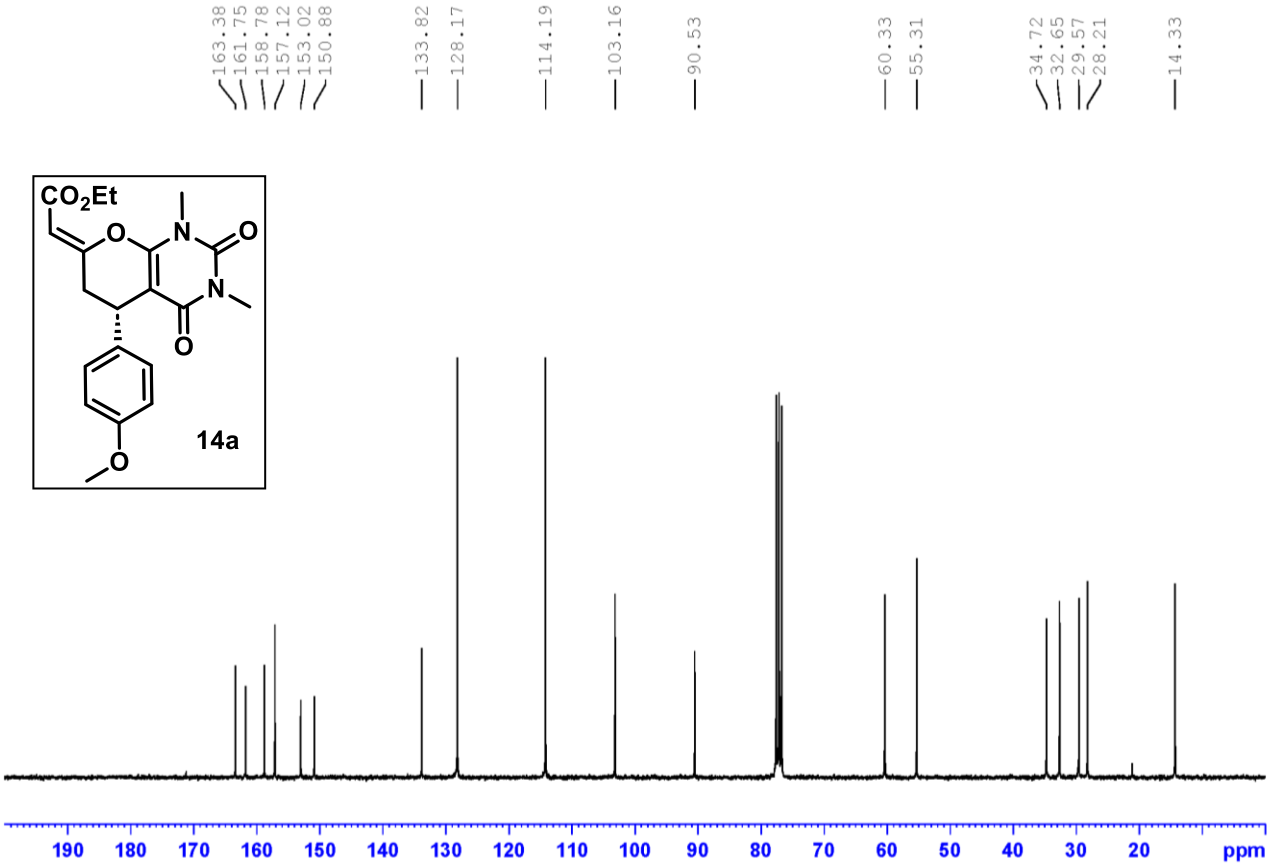
163.38
161.75
158.78
157.12
153.02
150.88
133.82
128.17
114.19
103.16
90.53
60.33
55.31
34.72
32.65
29.57
28.21
14.33
CO_2Et
14a
ppm

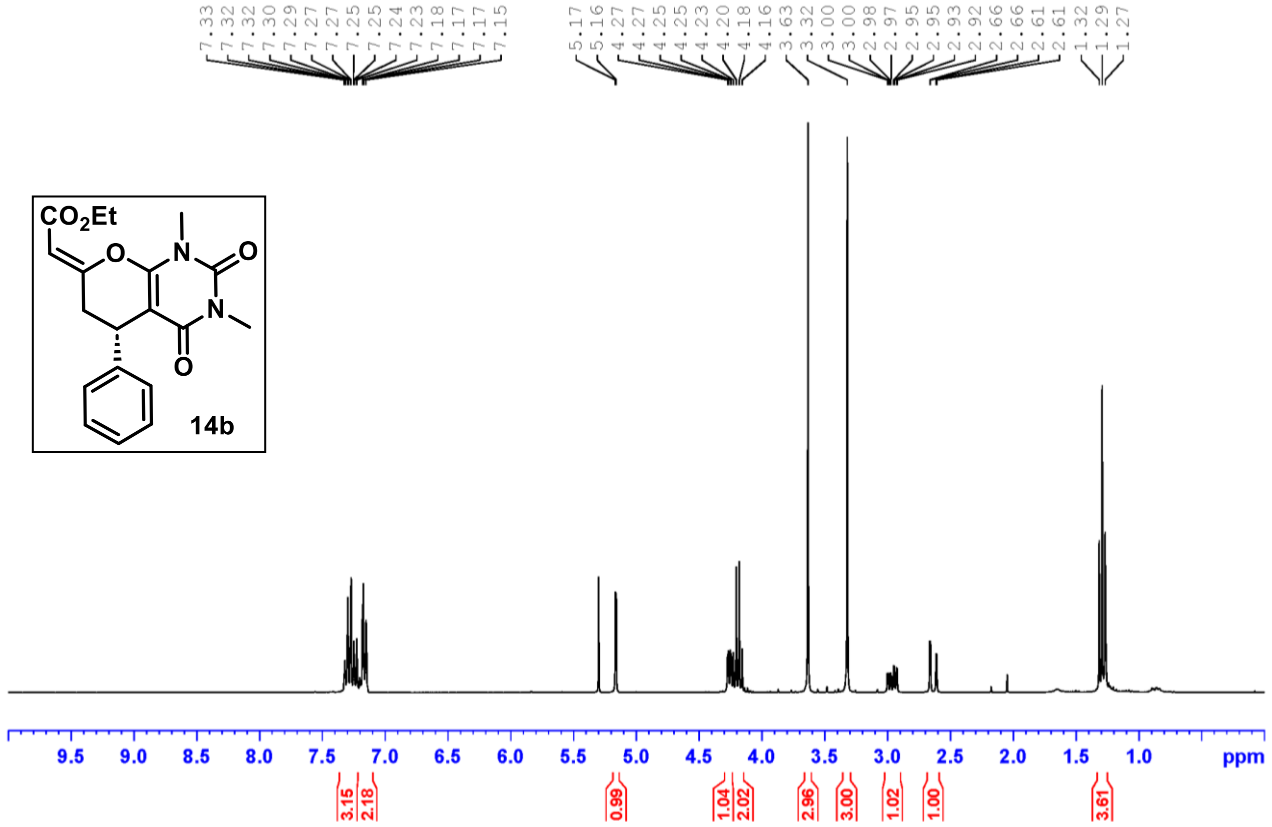
7.33
7.32
7.32
7.30
7.29
7.27
7.27
7.25
7.25
7.24
7.23
7.18
7.17
7.17
7.15
5.17
5.16
4.27
4.27
4.25
4.25
4.23
4.20
4.18
4.16
3.63
3.32
3.00
2.98
2.97
2.95
2.95
2.93
2.92
2.66
2.66
2.61
2.61
1.32
1.29
1.27
CO_2Et
14b
ppm
3.15
2.18
0.99
1.04
2.02
2.96
3.00
1.02
1.00
3.61

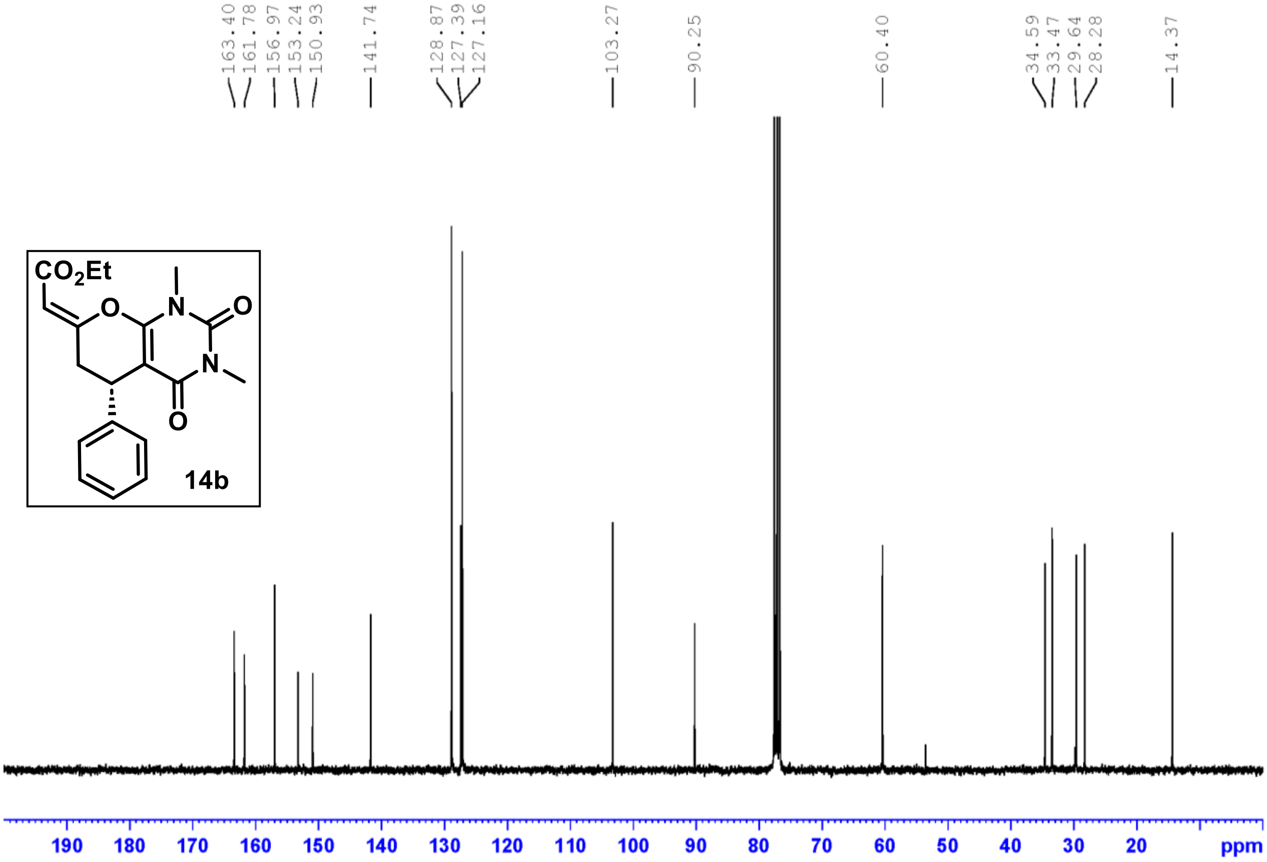
163.40
161.78
156.97
153.24
150.93
141.74
128.87
127.39
127.16
103.27
90.25
60.40
34.59
33.47
29.64
28.28
14.37
CO_2Et
O
N
O
N
O
14b
190 180 170 160 150 140 130 120 110 100 90 80 70 60 50 40 30 20 ppm

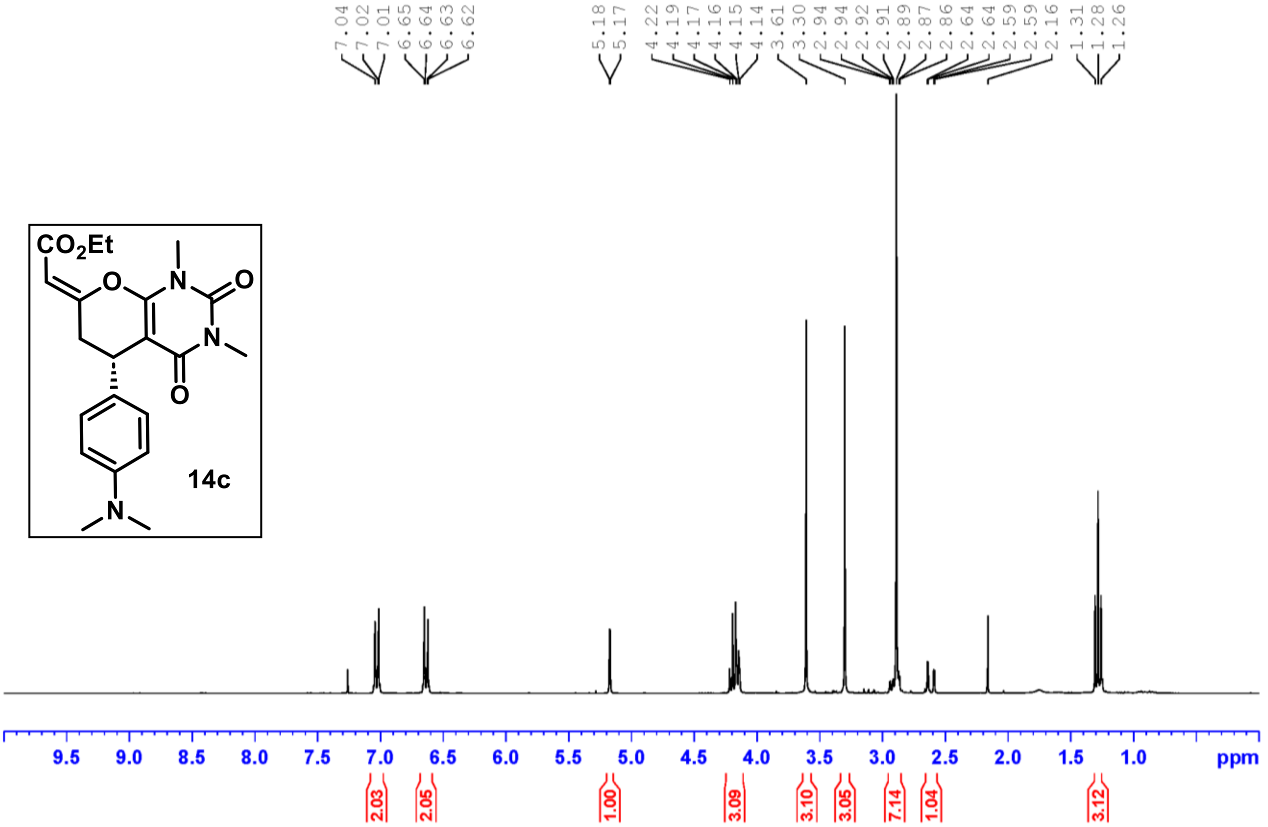
7.04
7.02
7.01
6.65
6.64
6.63
6.62
5.18
5.17
4.22
4.19
4.17
4.16
4.15
4.14
3.61
3.30
2.94
2.94
2.92
2.91
2.89
2.87
2.86
2.64
2.64
2.59
2.59
2.16
1.31
1.28
1.26
CO_2Et
O
N
O
N
O
N
14c
9.5 9.0 8.5 8.0 7.5 7.0 6.5 6.0 5.5 5.0 4.5 4.0 3.5 3.0 2.5 2.0 1.5 1.0 ppm
2.03
2.05
1.00
3.09
3.10
3.05
7.14
1.04
3.12

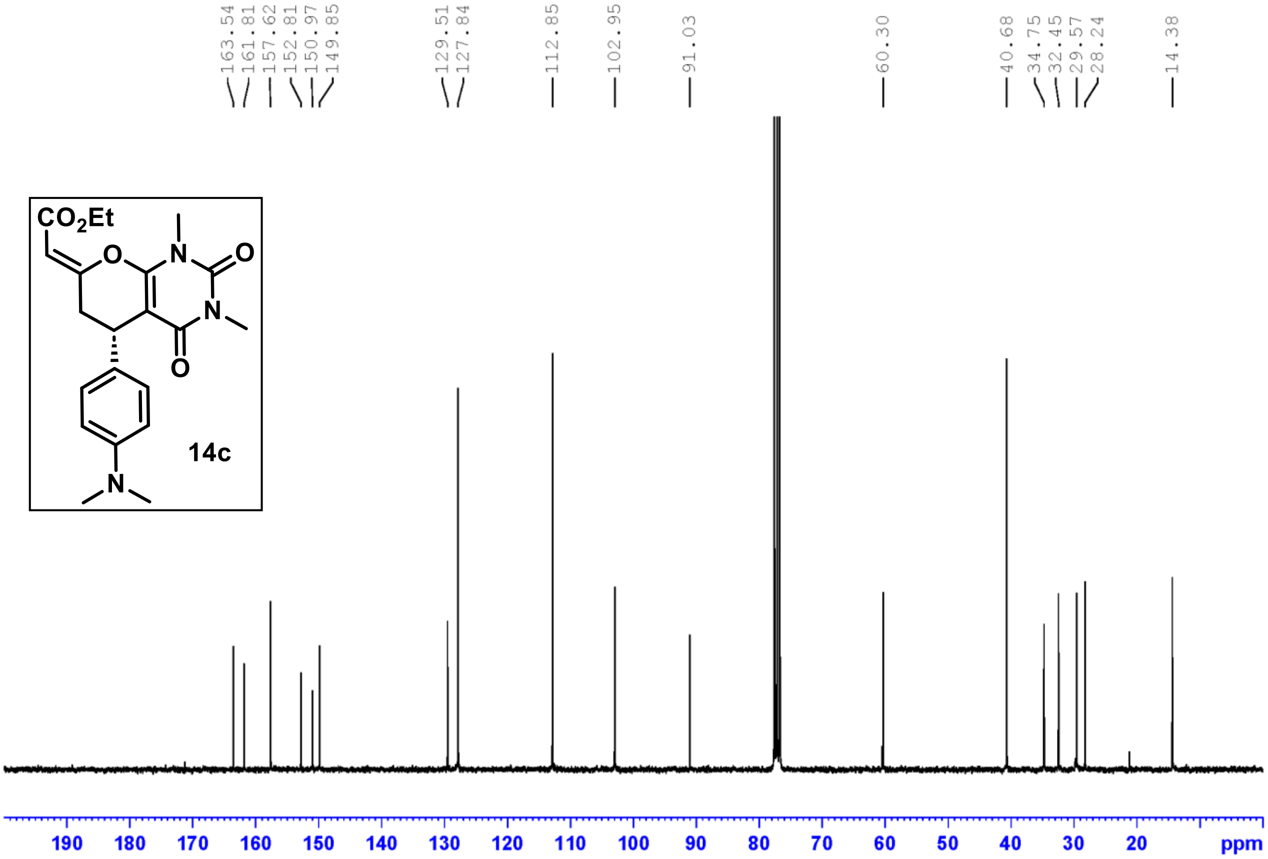
163.54
161.81
157.62
152.81
150.97
149.85
129.51
127.84
112.85
102.95
91.03
60.30
40.68
34.75
32.45
29.57
28.24
14.38
14c
190 180 170 160 150 140 130 120 110 100 90 80 70 60 50 40 30 20 ppm

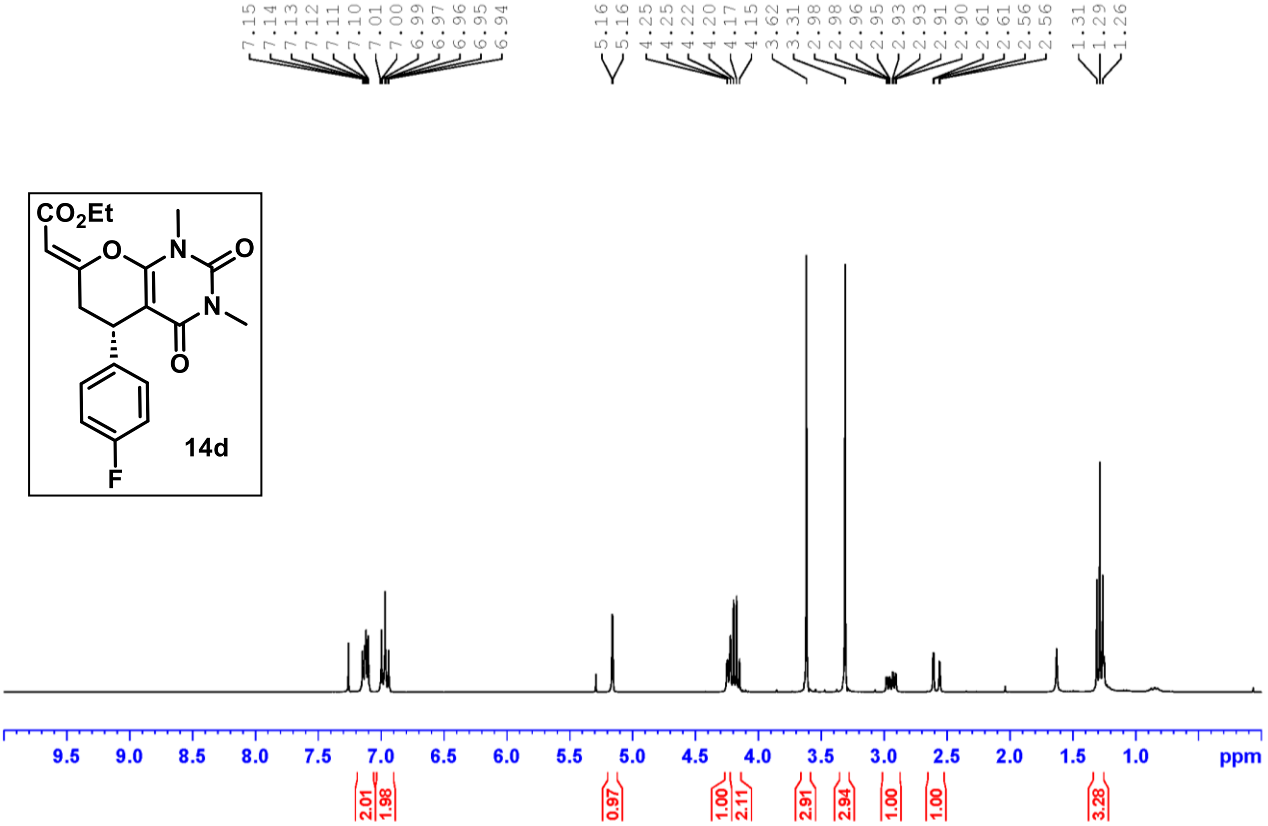
7.15
7.14
7.13
7.12
7.11
7.10
7.01
7.00
6.99
6.97
6.96
6.95
6.94
5.16
5.16
4.25
4.25
4.22
4.20
4.17
4.15
3.62
3.31
2.98
2.98
2.96
2.95
2.93
2.93
2.91
2.90
2.61
2.56
2.56
1.31
1.29
1.26
14d
9.5 9.0 8.5 8.0 7.5 7.0 6.5 6.0 5.5 5.0 4.5 4.0 3.5 3.0 2.5 2.0 1.5 1.0 ppm
2.01
1.98
0.97
1.00
2.11
2.91
2.94
1.00
1.00
3.28

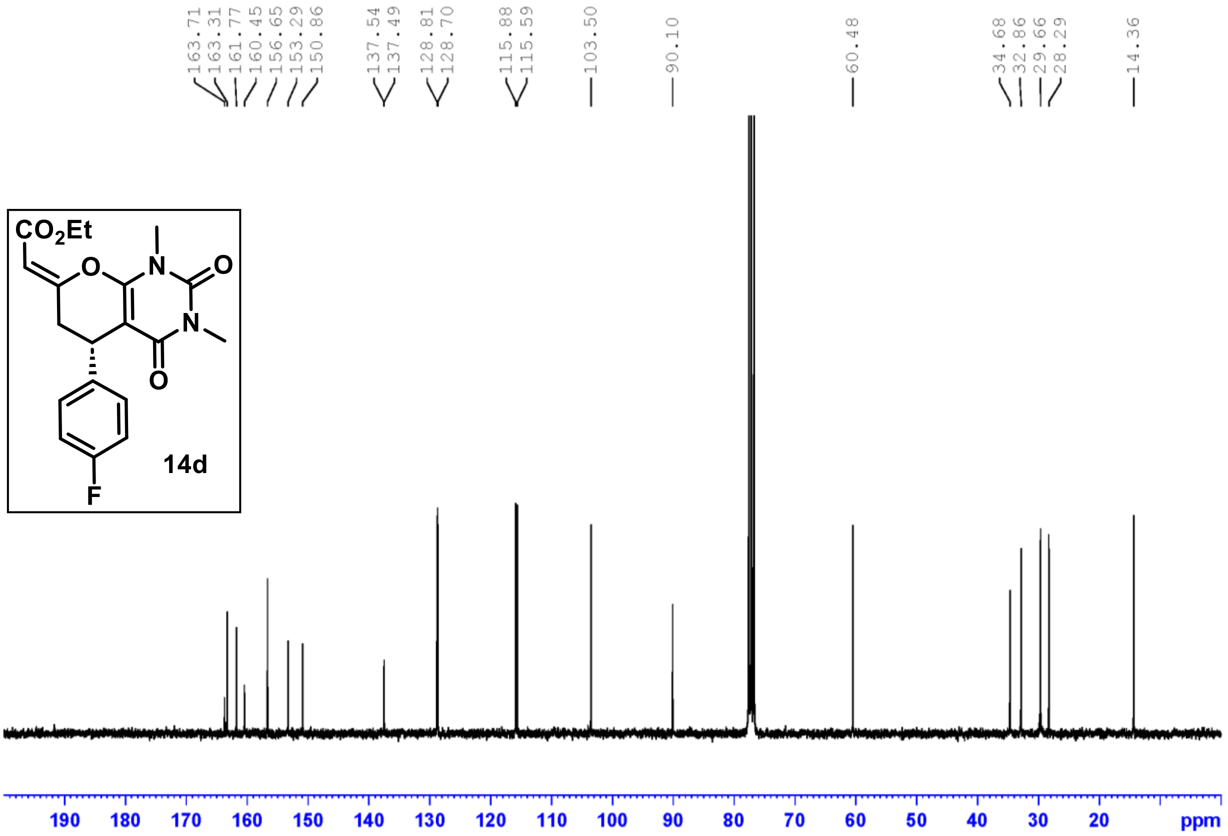

163.71
163.31
161.77
160.45
156.65
153.29
150.86
137.54
137.49
128.81
128.70
115.88
115.59
103.50
90.10
60.48
34.68
32.86
29.66
28.29
14.36
CO_2Et
O
N
O
N
O
F
14d
190 180 170 160 150 140 130 120 110 100 90 80 70 60 50 40 30 20 ppm

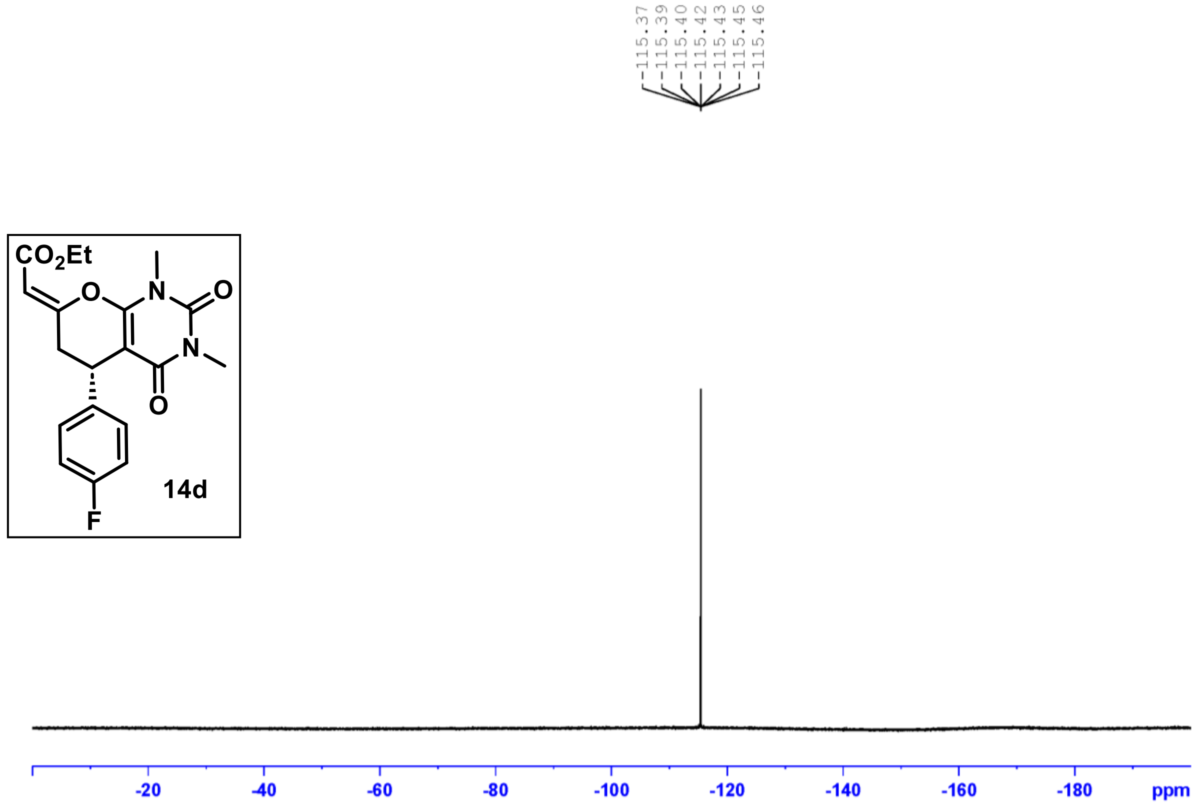

-115.37
-115.39
-115.40
-115.42
-115.43
-115.45
-115.46
CO_2Et
O
N
O
N
O
F
14d
-20 -40 -60 -80 -100 -120 -140 -160 -180 ppm

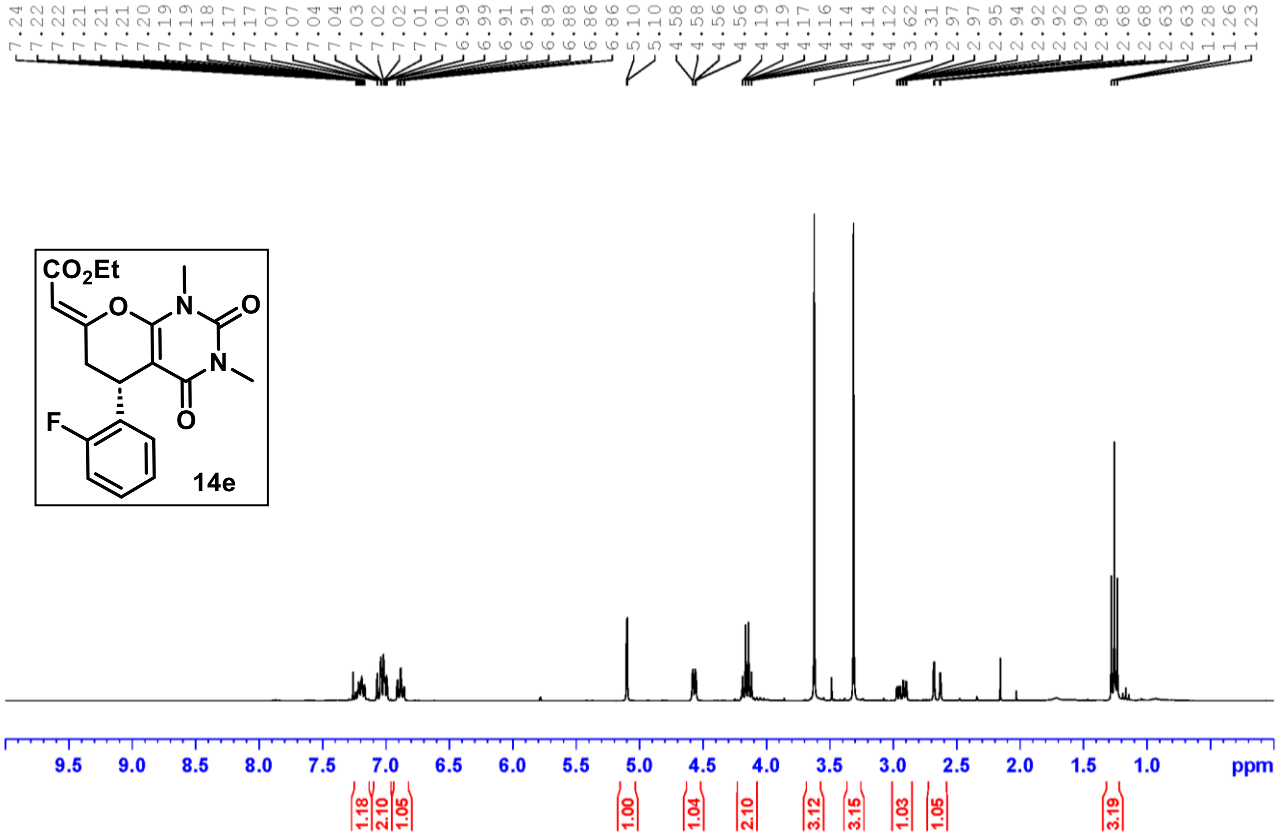
7.24 7.22 7.22 7.21 7.21 7.21 7.20 7.19 7.19 7.18 7.17 7.17 7.07 7.07 7.04 7.04 7.03 7.02 7.02 7.01 7.01 6.99 6.99 6.91 6.91 6.89 6.88 6.86 6.86 5.10 5.10 4.58 4.58 4.56 4.56 4.19 4.19 4.17 4.16 4.14 4.14 4.12 3.62 3.31 2.97 2.97 2.95 2.94 2.92 2.92 2.90 2.89 2.68 2.68 2.63 2.63 1.28 1.26 1.23
CO2Et
O
N
O
N
O
F
14e
9.5 9.0 8.5 8.0 7.5 7.0 6.5 6.0 5.5 5.0 4.5 4.0 3.5 3.0 2.5 2.0 1.5 1.0 ppm
1.18 2.10 1.05 1.00 1.04 2.10 3.12 3.15 1.03 1.05 3.19

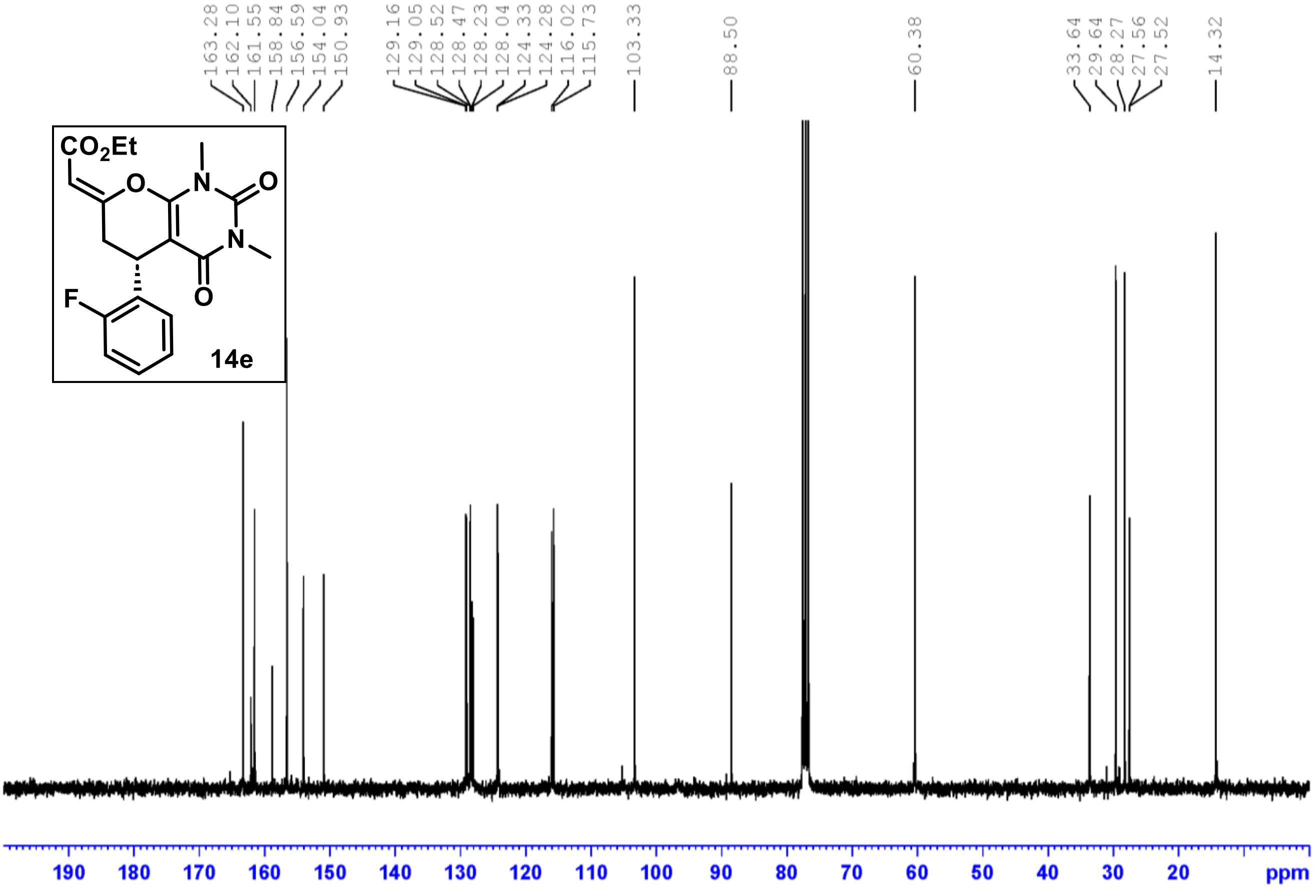
163.28 162.10 161.55 158.84 156.59 154.04 150.93 129.16 129.05 128.52 128.47 128.23 128.04 124.33 124.28 116.02 115.73 103.33 88.50 60.38 33.64 29.64 28.27 27.56 27.52 14.32
CO2Et
O
N
O
N
O
F
14e
190 180 170 160 150 140 130 120 110 100 90 80 70 60 50 40 30 20 ppm

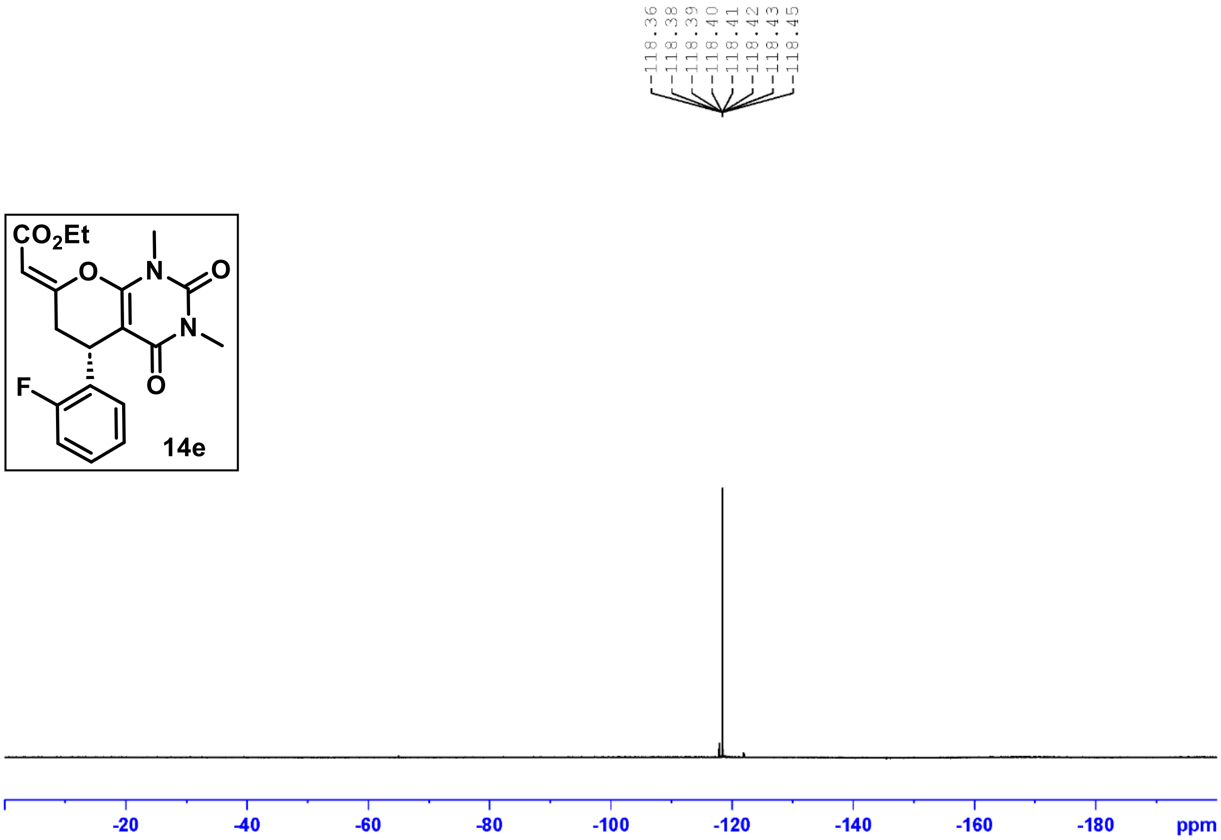
-118.36
-118.38
-118.39
-118.40
-118.41
-118.42
-118.43
-118.45
CO2Et
O
N
N
O
O
F
14e
-20
-40
-60
-80
-100
-120
-140
-160
-180
ppm

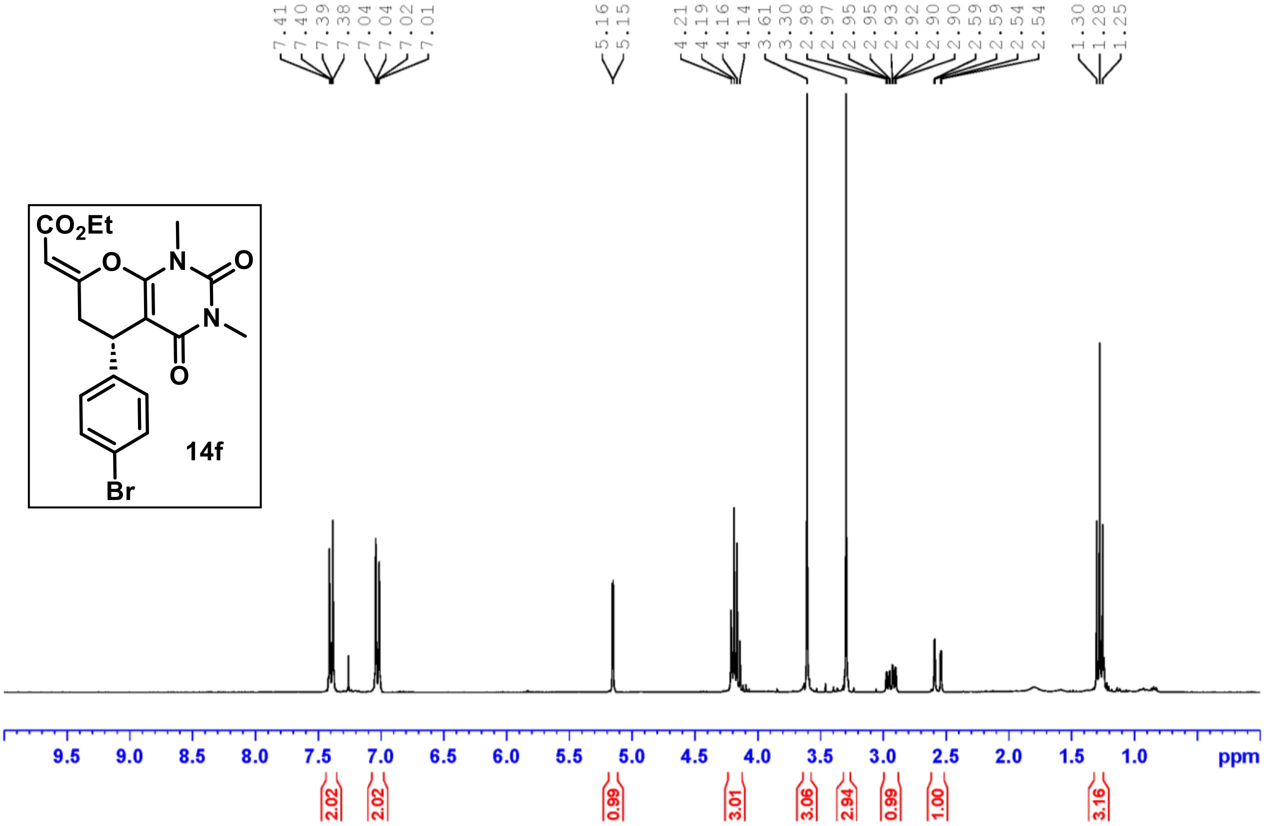
7.41
7.40
7.39
7.38
7.04
7.04
7.02
7.01
5.16
5.15
4.21
4.19
4.16
4.14
3.61
3.30
2.98
2.97
2.95
2.93
2.92
2.90
2.59
2.59
2.54
2.54
1.30
1.28
1.25
CO2Et
O
N
N
O
O
Br
14f
9.5
9.0
8.5
8.0
7.5
7.0
6.5
6.0
5.5
5.0
4.5
4.0
3.5
3.0
2.5
2.0
1.5
1.0
ppm
2.02
2.02
0.99
3.01
3.06
2.94
0.99
1.00
3.16

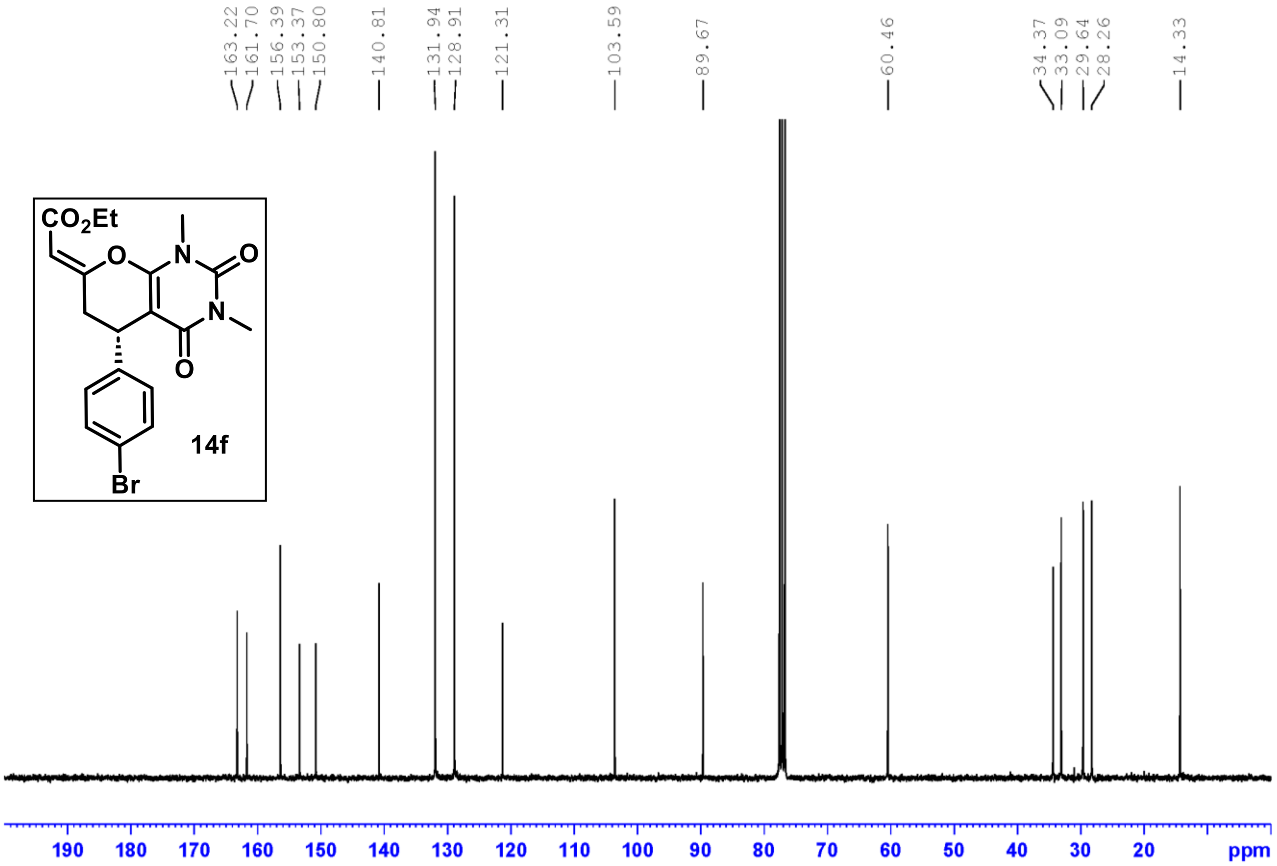
163.22
161.70
156.39
153.37
150.80
140.81
131.94
128.91
121.31
103.59
89.67
60.46
34.37
33.09
29.64
28.26
14.33
CO2Et
N
O
N
O
O
Br
14f
190 180 170 160 150 140 130 120 110 100 90 80 70 60 50 40 30 20 ppm

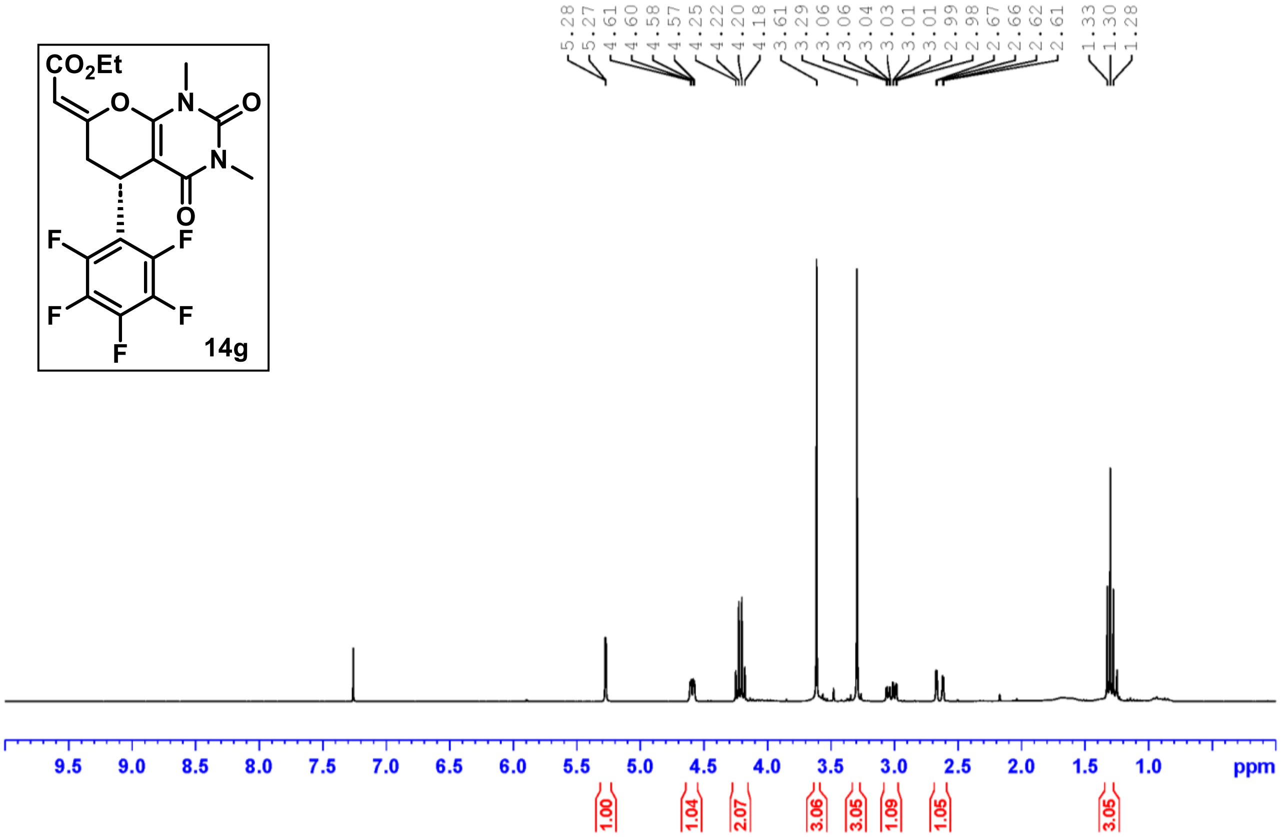
5.28
5.27
4.61
4.60
4.58
4.57
4.25
4.22
4.20
4.18
3.61
3.29
3.06
3.06
3.04
3.03
3.01
3.01
2.99
2.98
2.67
2.66
2.62
2.61
1.33
1.30
1.28
CO2Et
N
O
N
O
O
F
F
F
F
F
14g
9.5 9.0 8.5 8.0 7.5 7.0 6.5 6.0 5.5 5.0 4.5 4.0 3.5 3.0 2.5 2.0 1.5 1.0 ppm
1.00
1.04
2.07
3.06
3.05
1.09
1.05
3.05

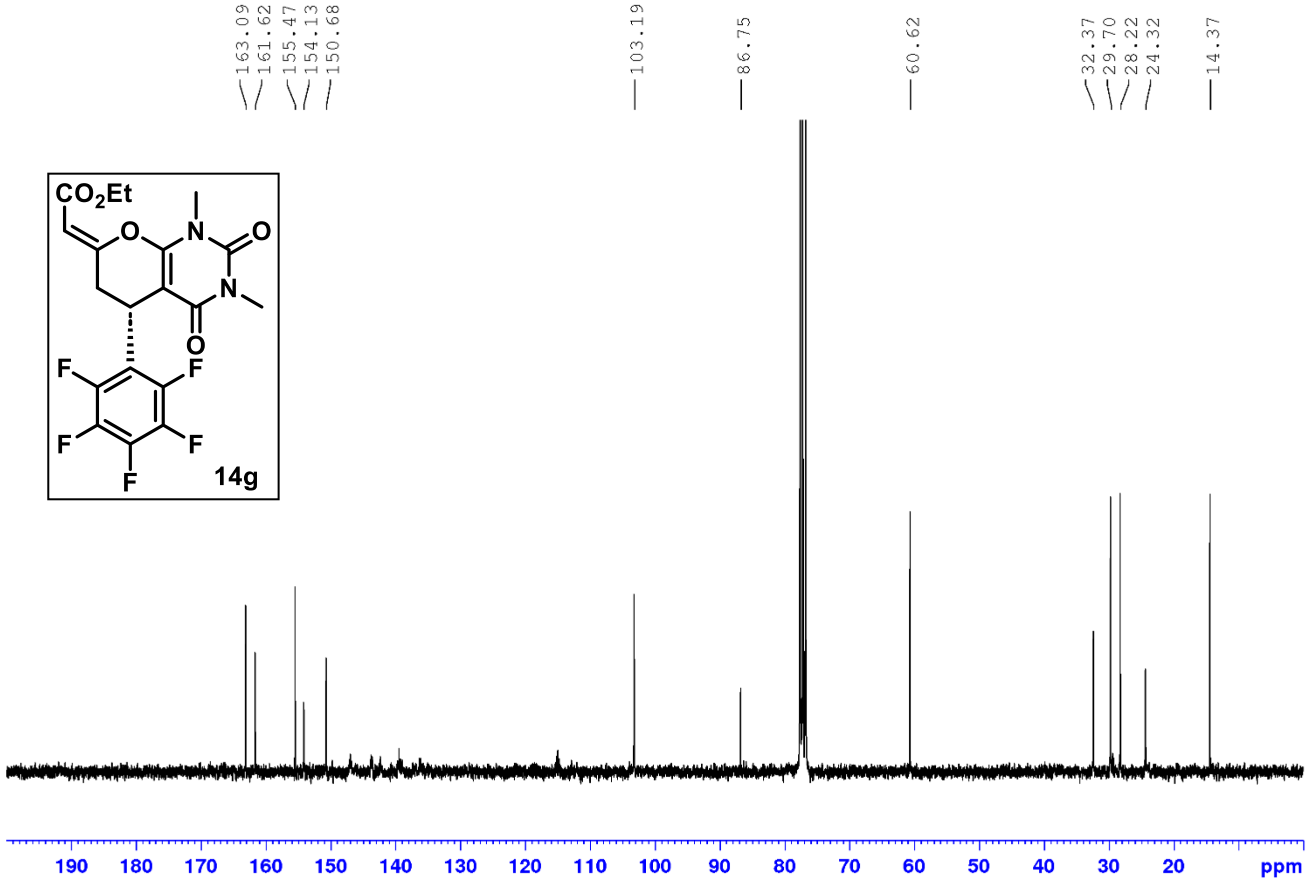

163.09
161.62
155.47
154.13
150.68
103.19
86.75
60.62
32.37
29.70
28.22
24.32
14.37
CO_2Et
O
N
O
N
O
F
F
F
F
F
14g
190 180 170 160 150 140 130 120 110 100 90 80 70 60 50 40 30 20 ppm

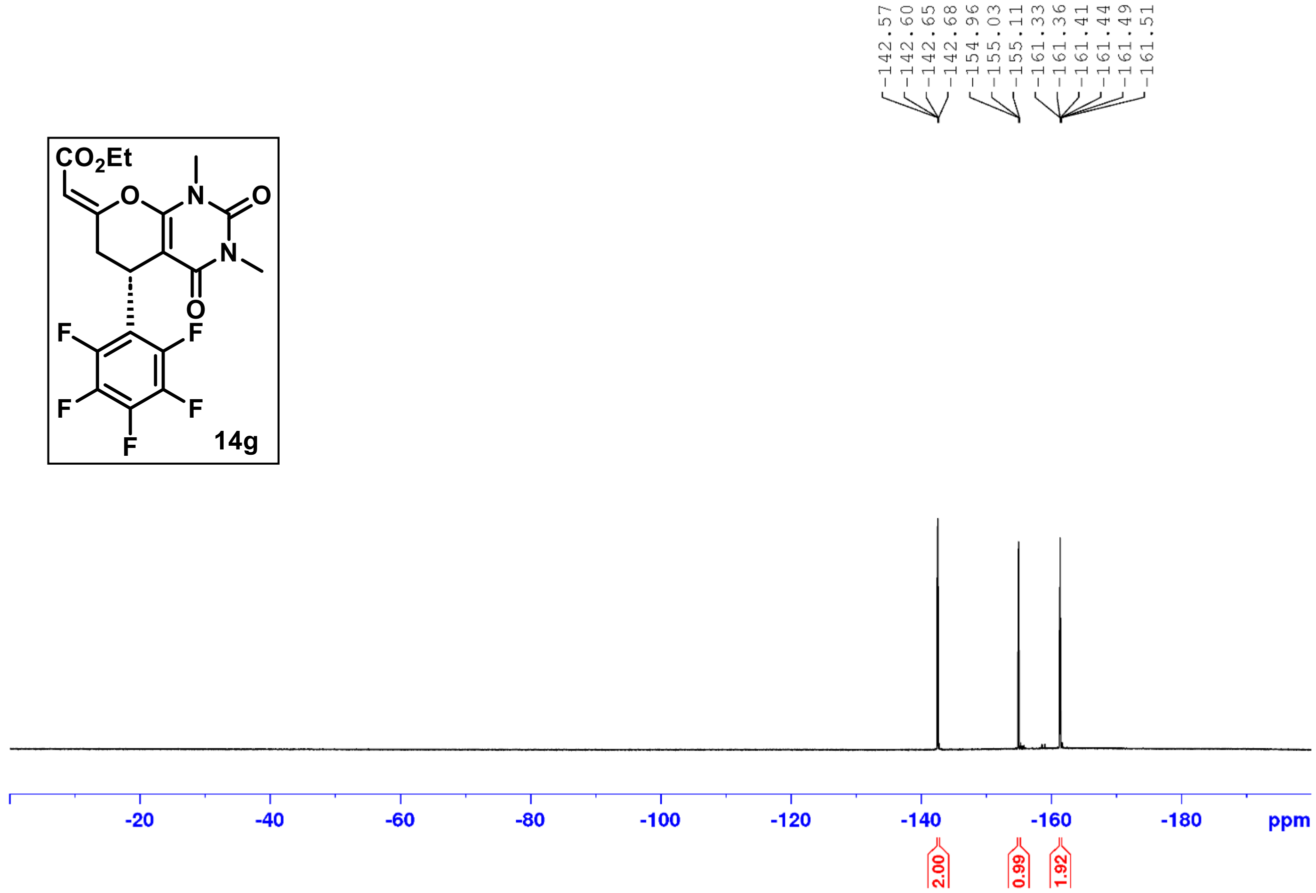

-142.57
-142.60
-142.65
-142.68
-154.96
-155.03
-155.11
-161.33
-161.36
-161.41
-161.44
-161.49
-161.51
CO_2Et
O
N
O
N
O
F
F
F
F
F
14g
-20 -40 -60 -80 -100 -120 -140 -160 -180 ppm
2.00
0.99
1.92

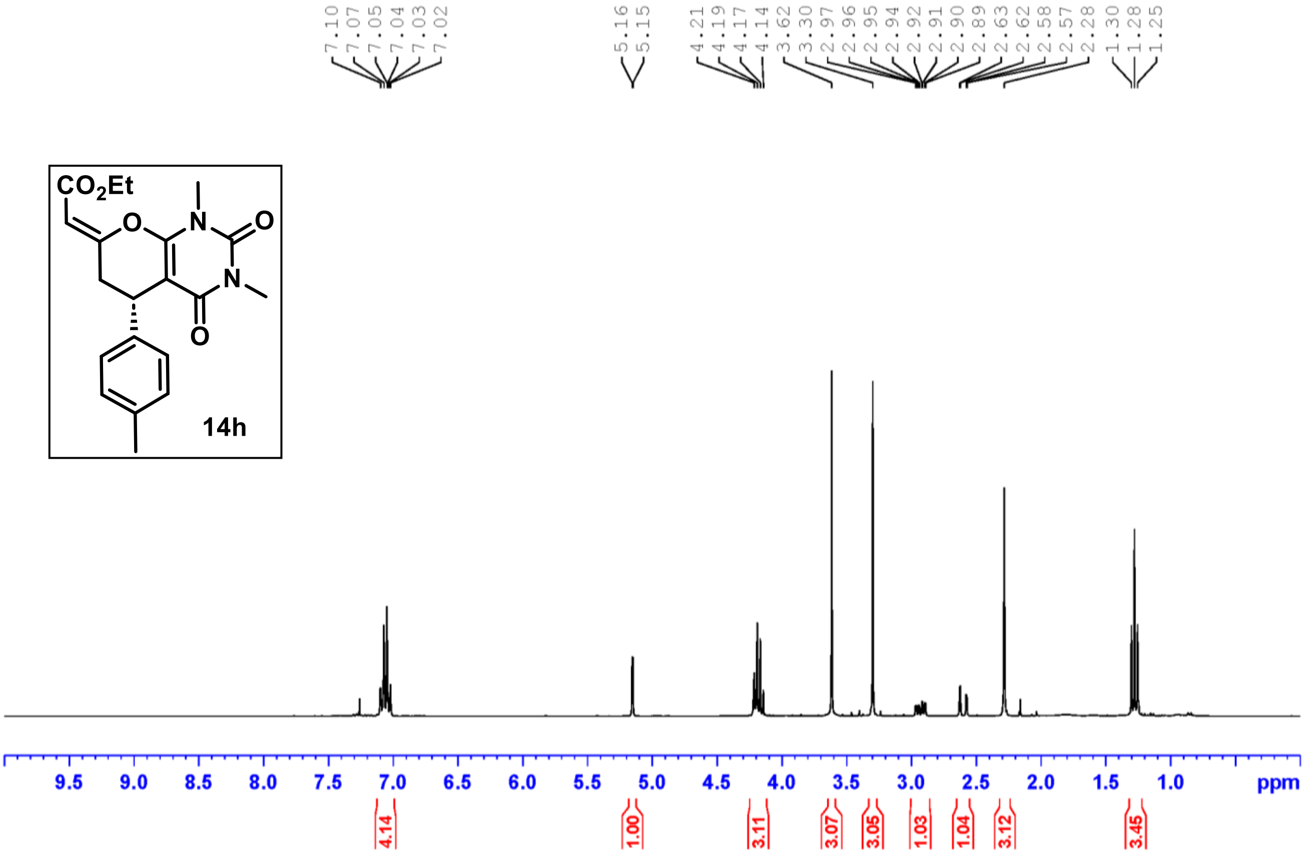
7.10 7.07 7.05 7.04 7.03 7.02
5.16 5.15
4.21 4.19 4.17 4.14 3.62 3.30 2.97 2.96 2.95 2.94 2.92 2.91 2.90 2.89 2.63 2.62 2.58 2.57 2.28 1.30 1.28 1.25
CO_2Et
O
N
O
N
O
14h
9.5 9.0 8.5 8.0 7.5 7.0 6.5 6.0 5.5 5.0 4.5 4.0 3.5 3.0 2.5 2.0 1.5 1.0 ppm
4.14 1.00 3.11 3.07 3.05 1.03 1.04 3.12 3.45

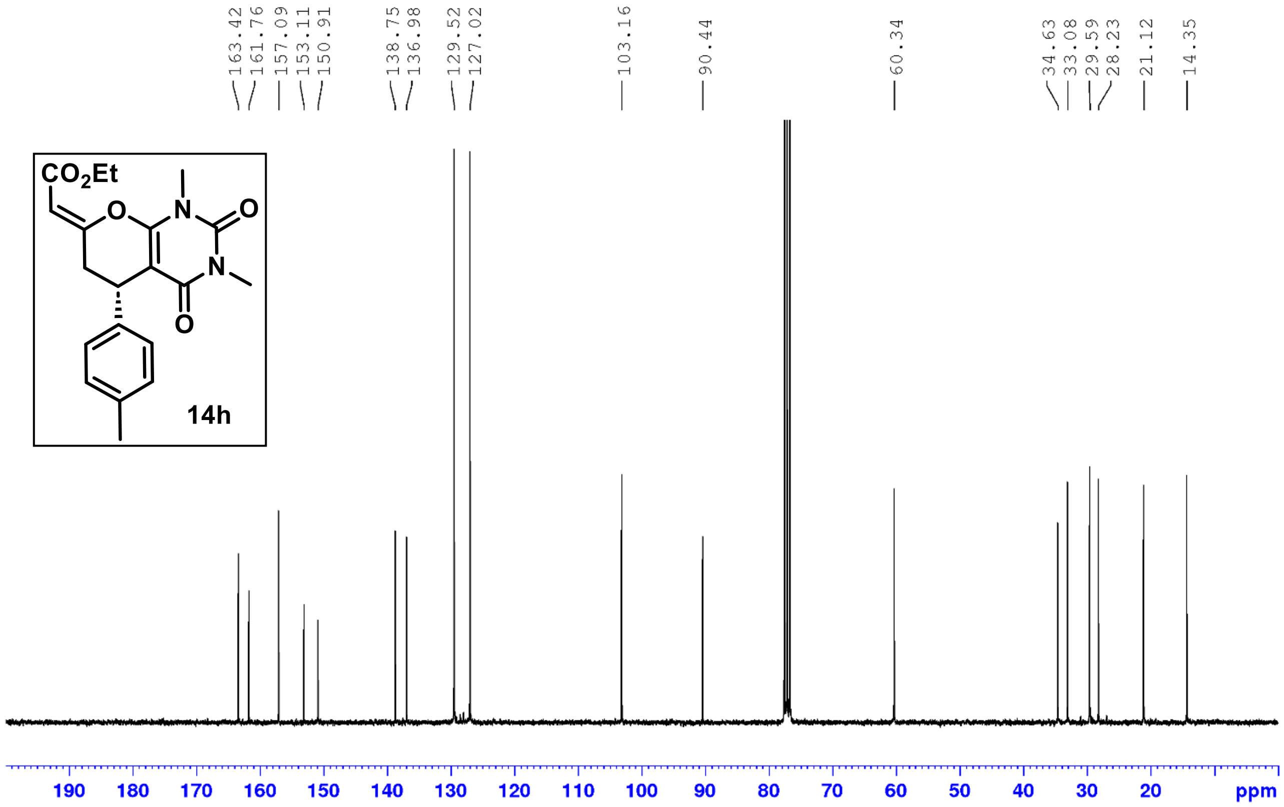
163.42 161.76 157.09 153.11 150.91 138.75 136.98 129.52 127.02
103.16
90.44
60.34
34.63 33.08 29.59 28.23 21.12 14.35
CO_2Et
O
N
O
N
O
14h
190 180 170 160 150 140 130 120 110 100 90 80 70 60 50 40 30 20 ppm

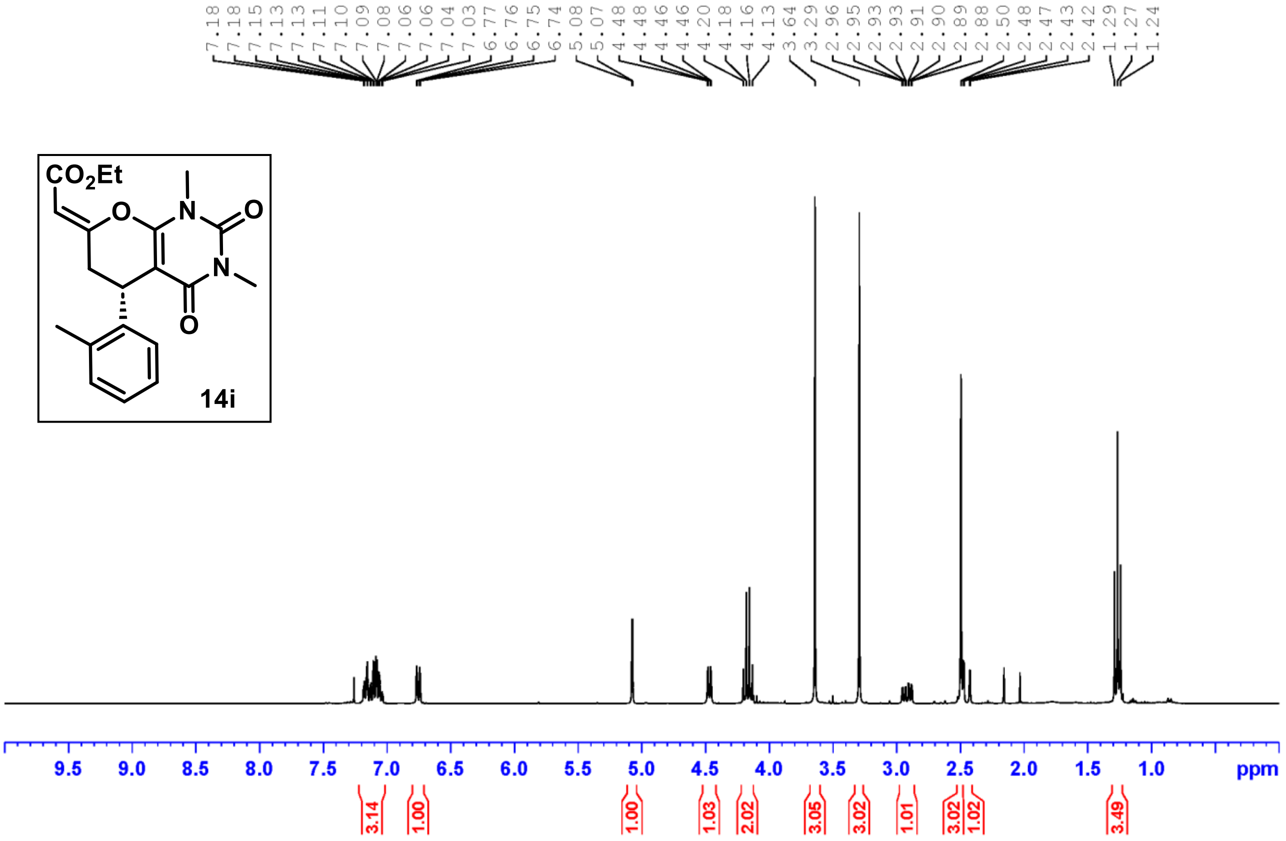
7.18 7.18 7.15 7.13 7.13 7.11 7.10 7.09 7.08 7.06 7.06 7.04 7.03 6.77 6.76 6.75 6.74 5.08 5.07 4.48 4.48 4.46 4.46 4.20 4.18 4.16 4.13 3.64 3.29 2.96 2.95 2.93 2.93 2.91 2.90 2.89 2.88 2.50 2.48 2.47 2.43 2.42 1.29 1.27 1.24
CO_2Et
O
N
O
N
O
14i
9.5 9.0 8.5 8.0 7.5 7.0 6.5 6.0 5.5 5.0 4.5 4.0 3.5 3.0 2.5 2.0 1.5 1.0 ppm
3.14 1.00 1.00 1.03 2.02 3.05 3.02 1.01 3.02 1.02 3.49

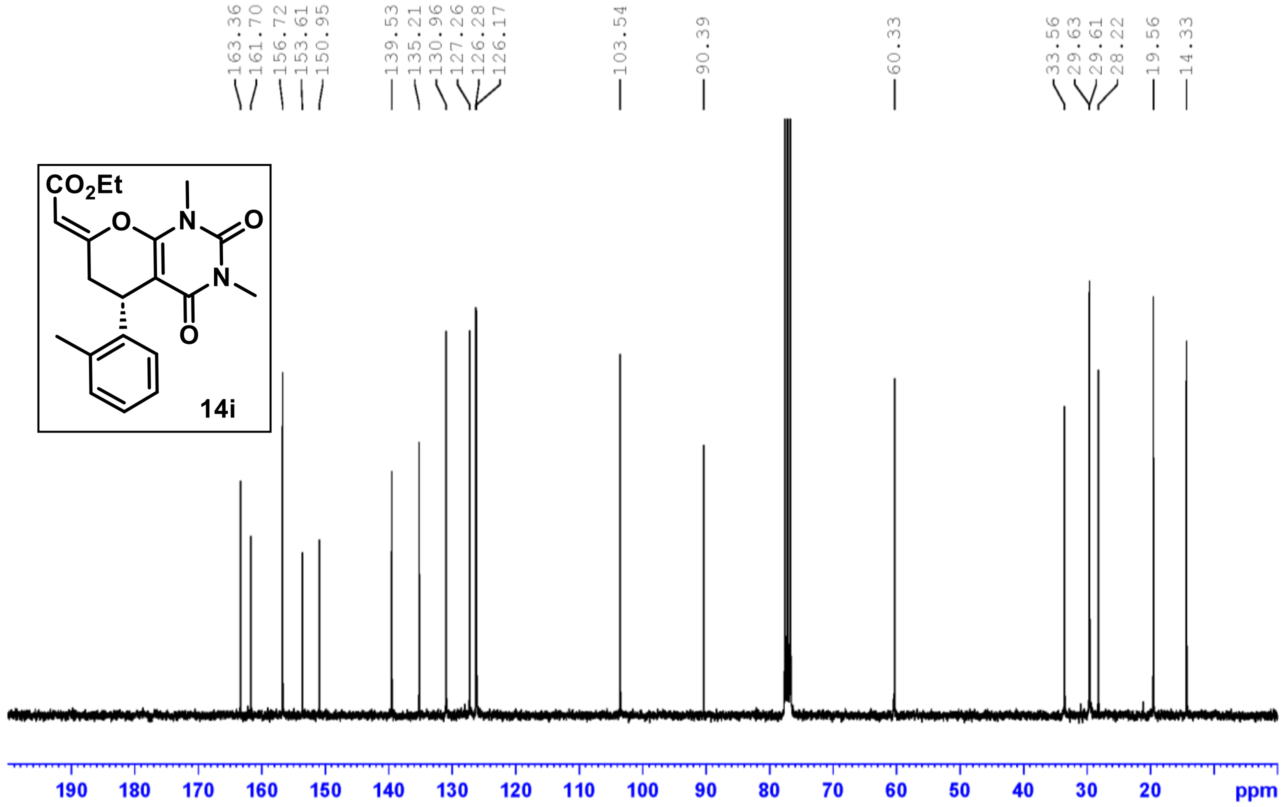
163.36 161.70 156.72 153.61 150.95 139.53 135.21 130.96 127.26 126.28 126.17 103.54 90.39 60.33 33.56 29.63 29.61 28.22 19.56 14.33
CO_2Et
O
N
O
N
O
14i
190 180 170 160 150 140 130 120 110 100 90 80 70 60 50 40 30 20 ppm

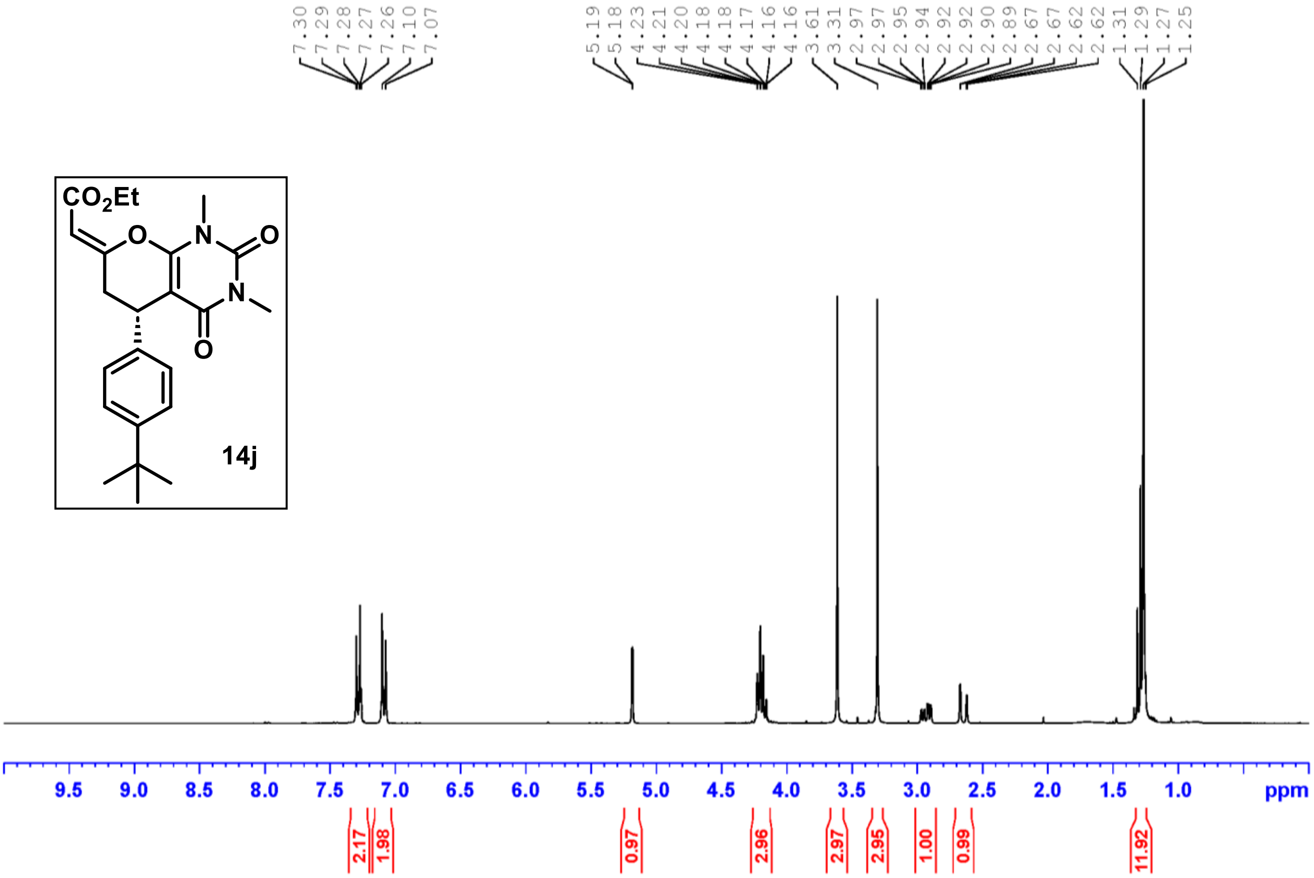
7.30
7.29
7.28
7.27
7.26
7.10
7.07
5.19
5.18
4.23
4.21
4.20
4.18
4.18
4.17
4.16
4.16
3.61
3.31
2.97
2.97
2.95
2.94
2.92
2.92
2.90
2.89
2.67
2.67
2.62
2.62
1.31
1.29
1.27
1.25
14j
9.5 9.0 8.5 8.0 7.5 7.0 6.5 6.0 5.5 5.0 4.5 4.0 3.5 3.0 2.5 2.0 1.5 1.0 ppm
2.17
1.98
0.97
2.96
2.97
2.95
1.00
0.99
11.92

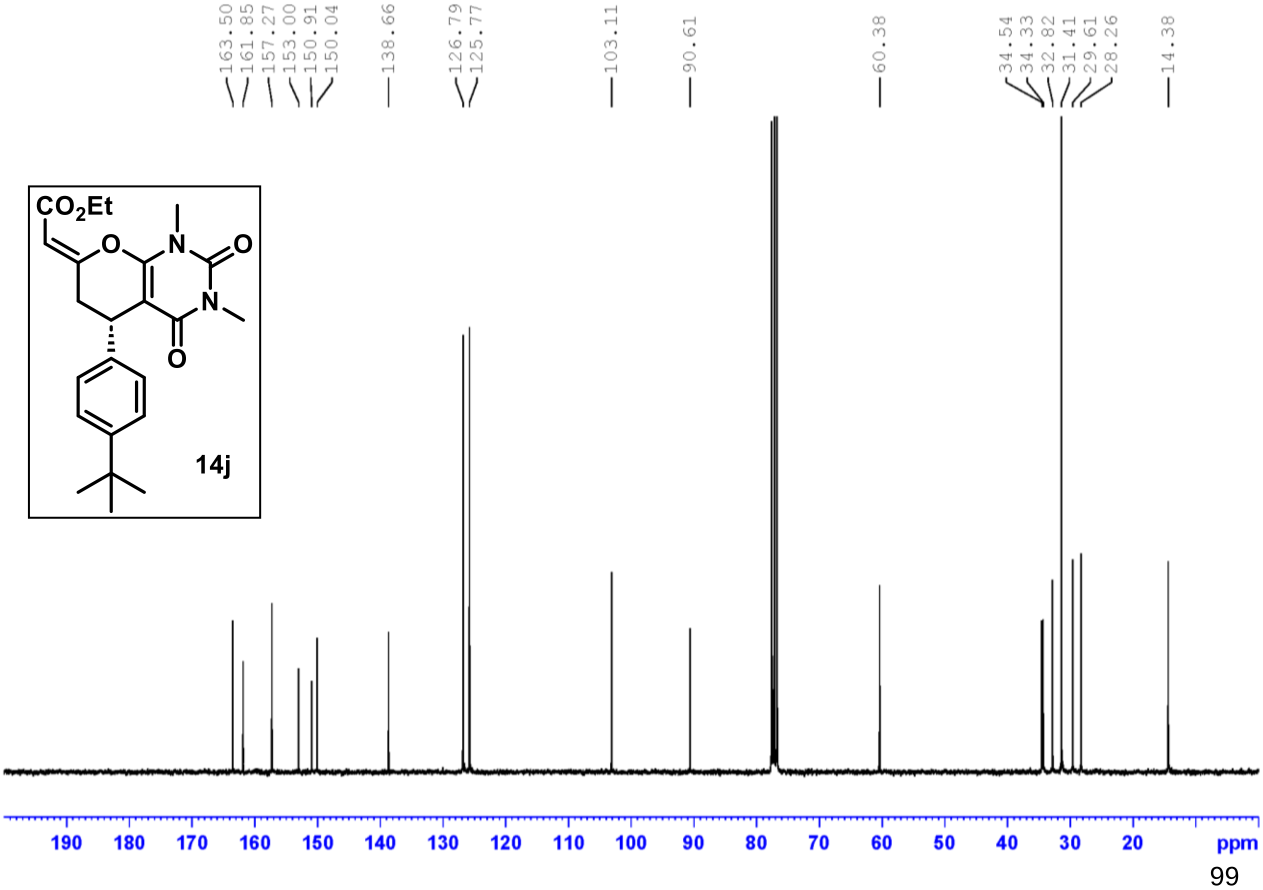
163.50
161.85
157.27
153.00
150.91
150.04
138.66
126.79
125.77
103.11
90.61
60.38
34.54
34.33
32.82
31.41
29.61
28.26
14.38
14j
190 180 170 160 150 140 130 120 110 100 90 80 70 60 50 40 30 20 ppm

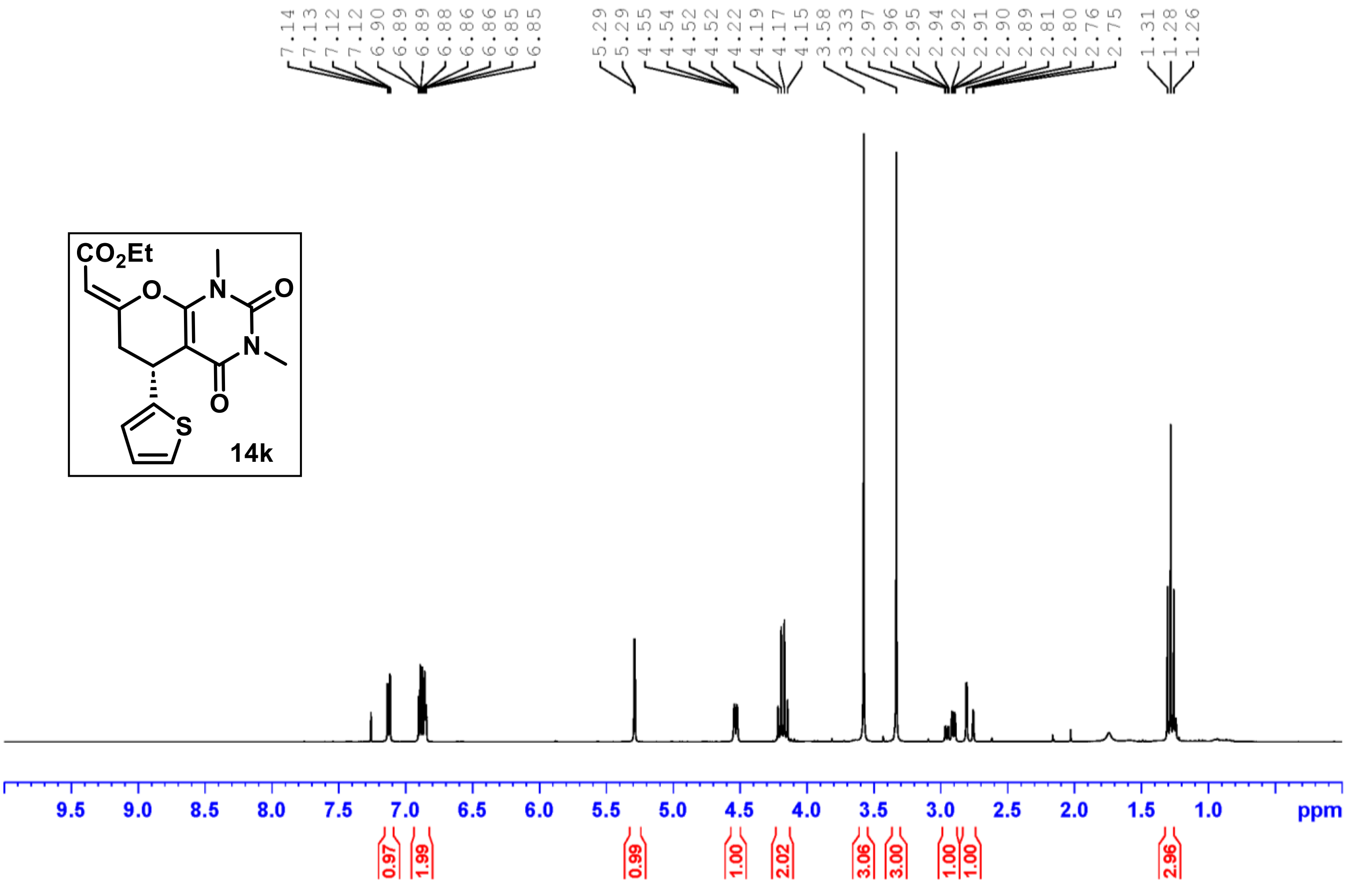
CO2Et
O
N
N
O
O
S
14k
7.14 7.13 7.12 7.12 6.90 6.89 6.89 6.88 6.86 6.86 6.85 6.85 5.29 5.29 4.55 4.54 4.52 4.52 4.22 4.19 4.17 4.15 3.58 3.33 2.97 2.96 2.95 2.94 2.92 2.91 2.90 2.89 2.81 2.80 2.76 2.75 1.31 1.28 1.26
9.5 9.0 8.5 8.0 7.5 7.0 6.5 6.0 5.5 5.0 4.5 4.0 3.5 3.0 2.5 2.0 1.5 1.0 ppm
0.97 1.99 0.99 1.00 2.02 3.06 3.00 1.00 1.00 2.96

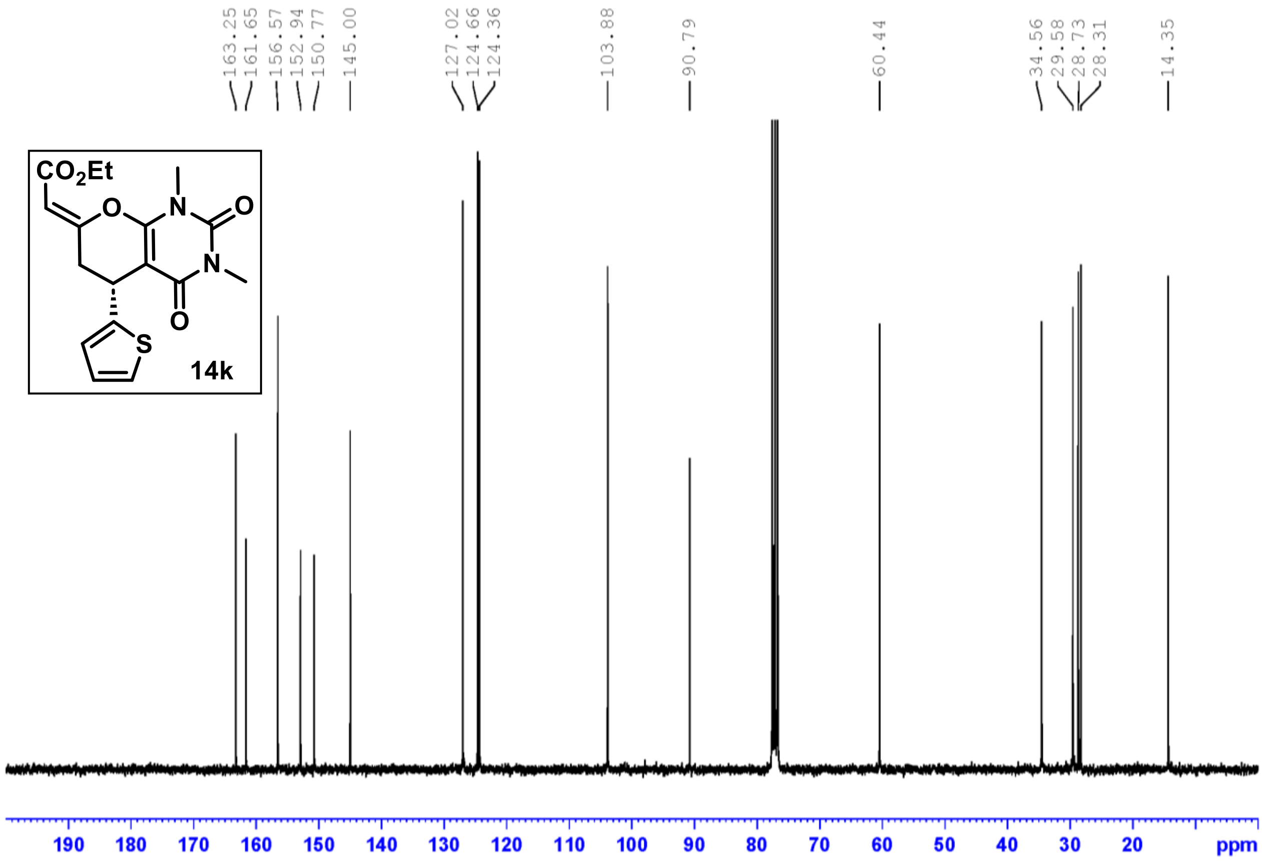
CO2Et
O
N
N
O
O
S
14k
163.25 161.65 156.57 152.94 150.77 145.00 127.02 124.66 124.36 103.88 90.79 60.44 34.56 29.58 28.73 28.31 14.35
190 180 170 160 150 140 130 120 110 100 90 80 70 60 50 40 30 20 ppm

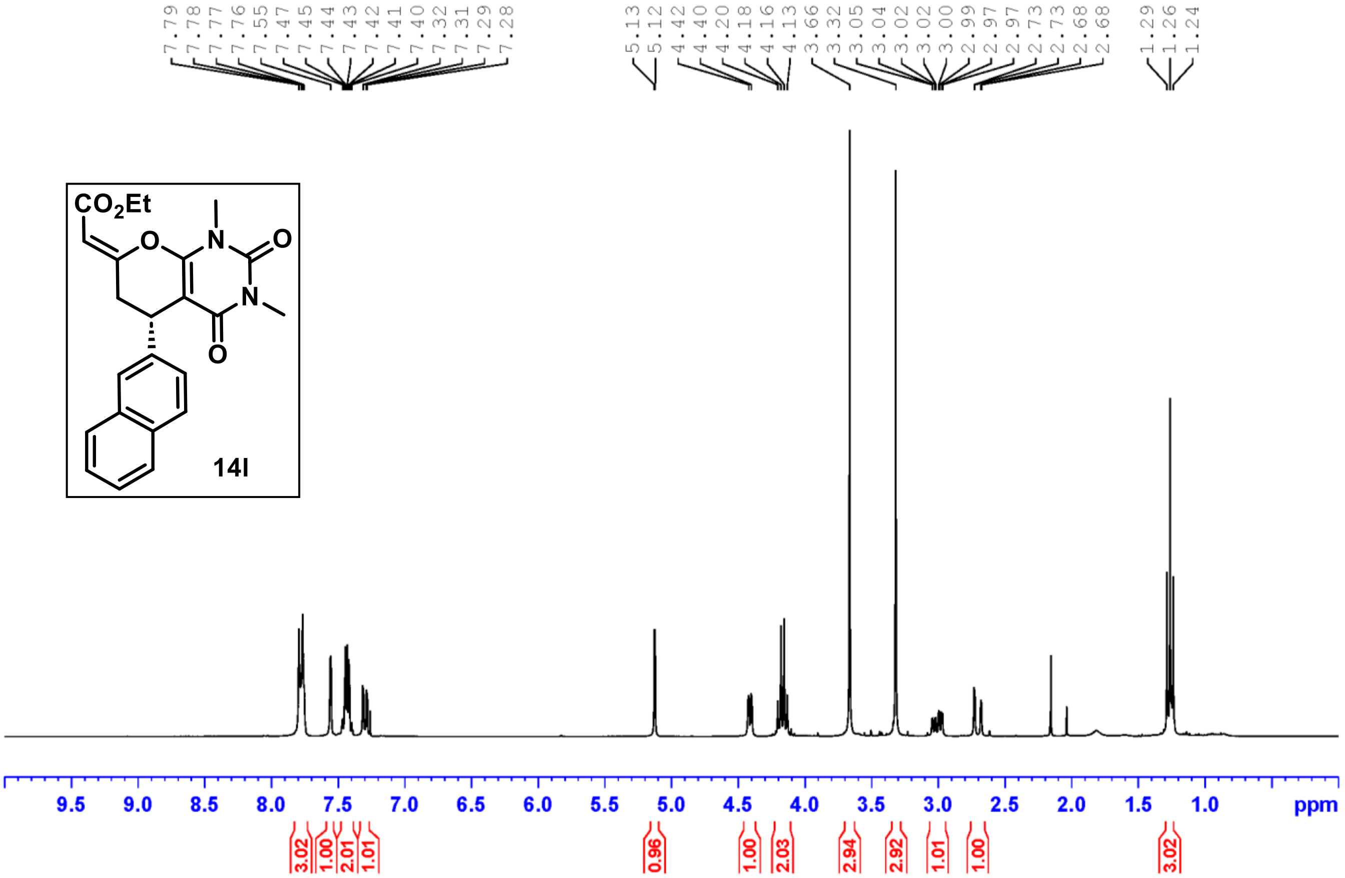
7.79 7.78 7.77 7.76 7.55 7.47 7.45 7.44 7.43 7.42 7.41 7.40 7.32 7.31 7.29 7.28
5.13 5.12 4.42 4.40 4.20 4.18 4.16 4.13 3.66 3.32 3.05 3.04 3.02 3.02 3.00 2.99 2.97 2.97 2.73 2.73 2.68 2.68
1.29 1.26 1.24
CO2Et
N
O
N
O
O
14l
9.5 9.0 8.5 8.0 7.5 7.0 6.5 6.0 5.5 5.0 4.5 4.0 3.5 3.0 2.5 2.0 1.5 1.0 ppm
3.02 1.00 2.01 1.01 0.96 1.00 2.03 2.94 2.92 1.01 1.00 3.02

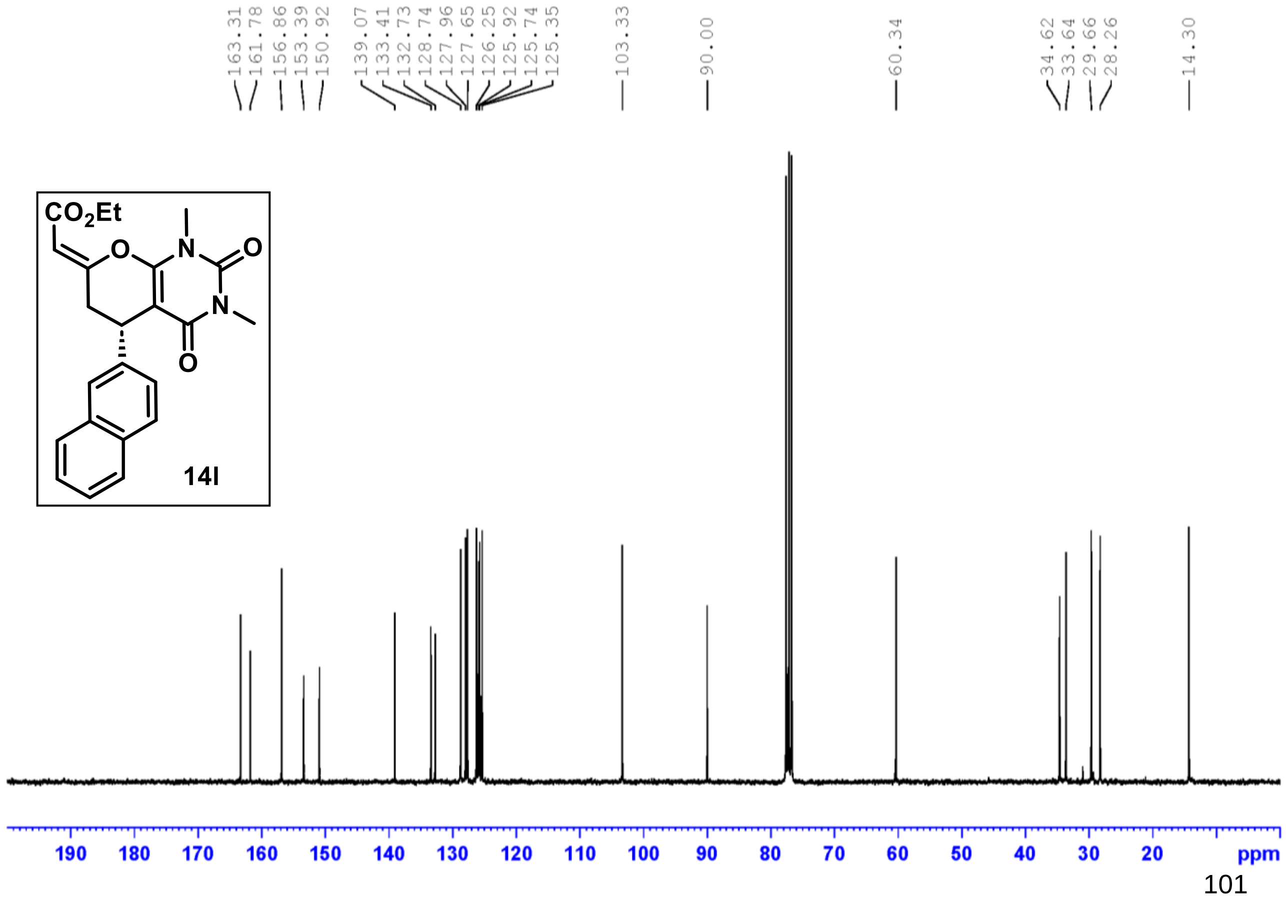
163.31 161.78 156.86 153.39 150.92 139.07 133.41 132.73 128.74 127.96 127.65 126.25 125.92 125.74 125.35 103.33 90.00 60.34 34.62 33.64 29.66 28.26 14.30
CO2Et
N
O
N
O
O
14l
190 180 170 160 150 140 130 120 110 100 90 80 70 60 50 40 30 20 ppm

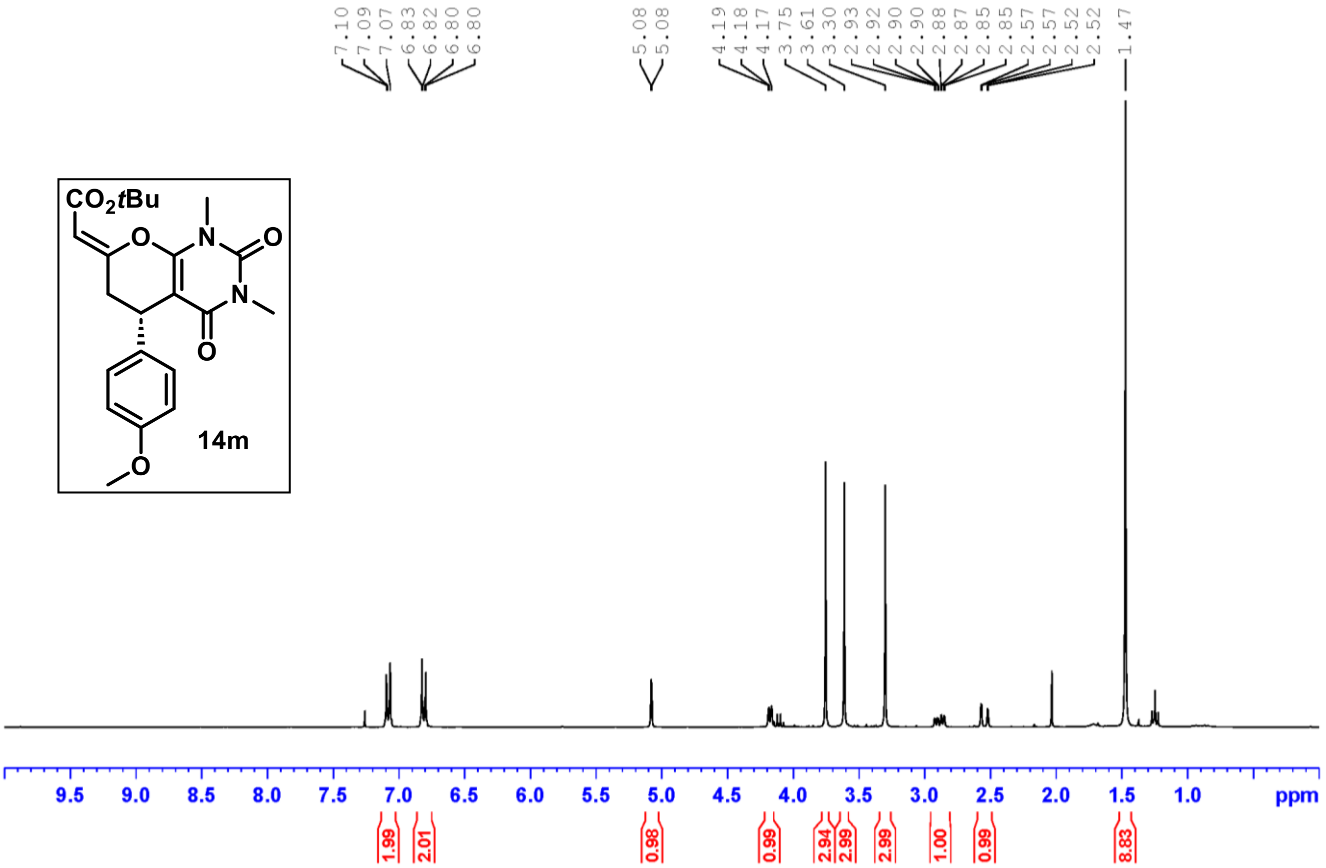
7.10 7.09 7.07 6.83 6.82 6.80 6.80
5.08 5.08
4.19 4.18 4.17 3.75 3.61 3.30 2.93 2.92 2.90 2.90 2.88 2.87 2.85 2.85 2.57 2.57 2.52 2.52
1.47
CO2tBu
14m
9.5 9.0 8.5 8.0 7.5 7.0 6.5 6.0 5.5 5.0 4.5 4.0 3.5 3.0 2.5 2.0 1.5 1.0 ppm
1.99 2.01 0.98 0.99 2.94 2.99 2.99 1.00 0.99 8.83

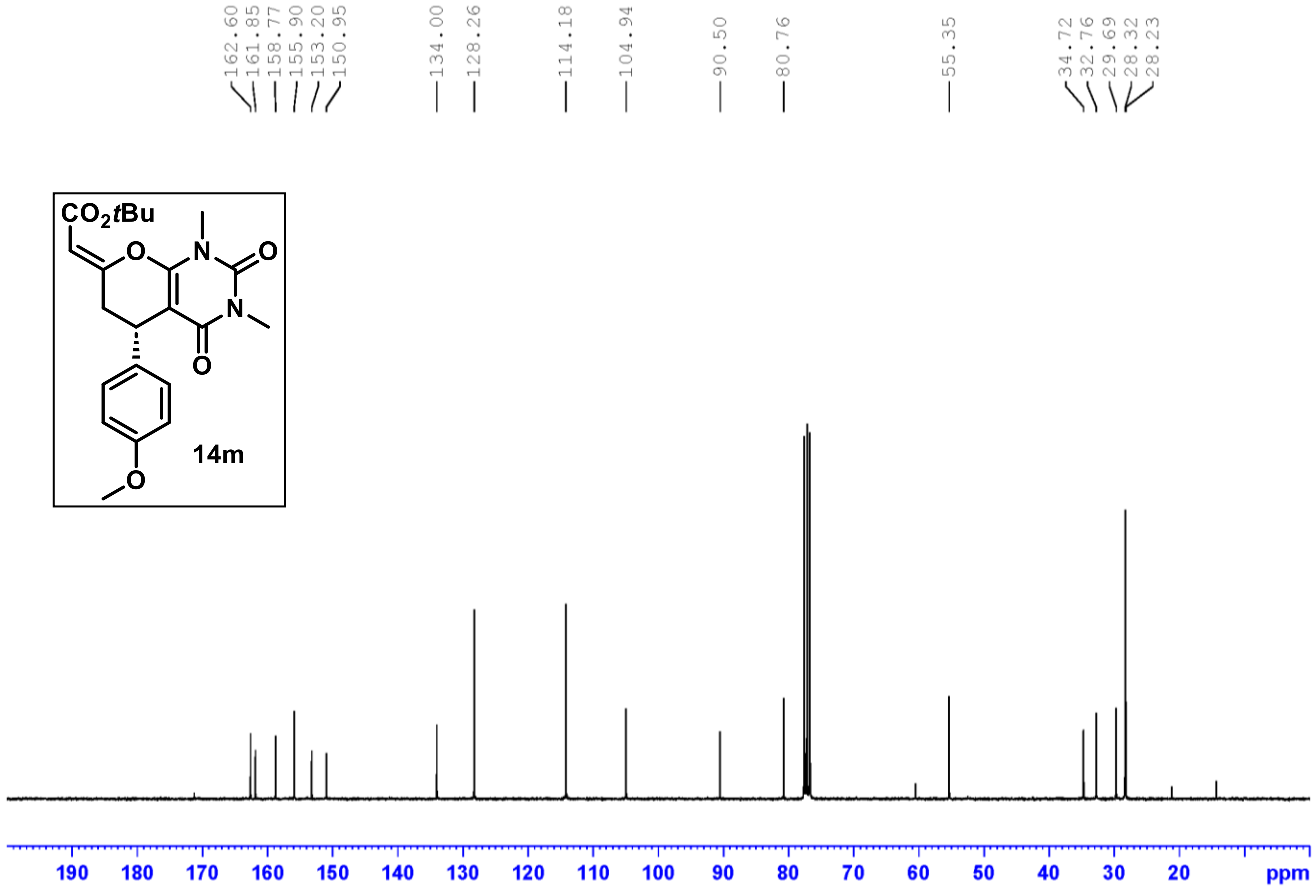
162.60 161.85 158.77 155.90 153.20 150.95
134.00 128.26
114.18
104.94
90.50
80.76
55.35
34.72 32.76 29.69 28.32 28.23
CO2tBu
14m
190 180 170 160 150 140 130 120 110 100 90 80 70 60 50 40 30 20 ppm

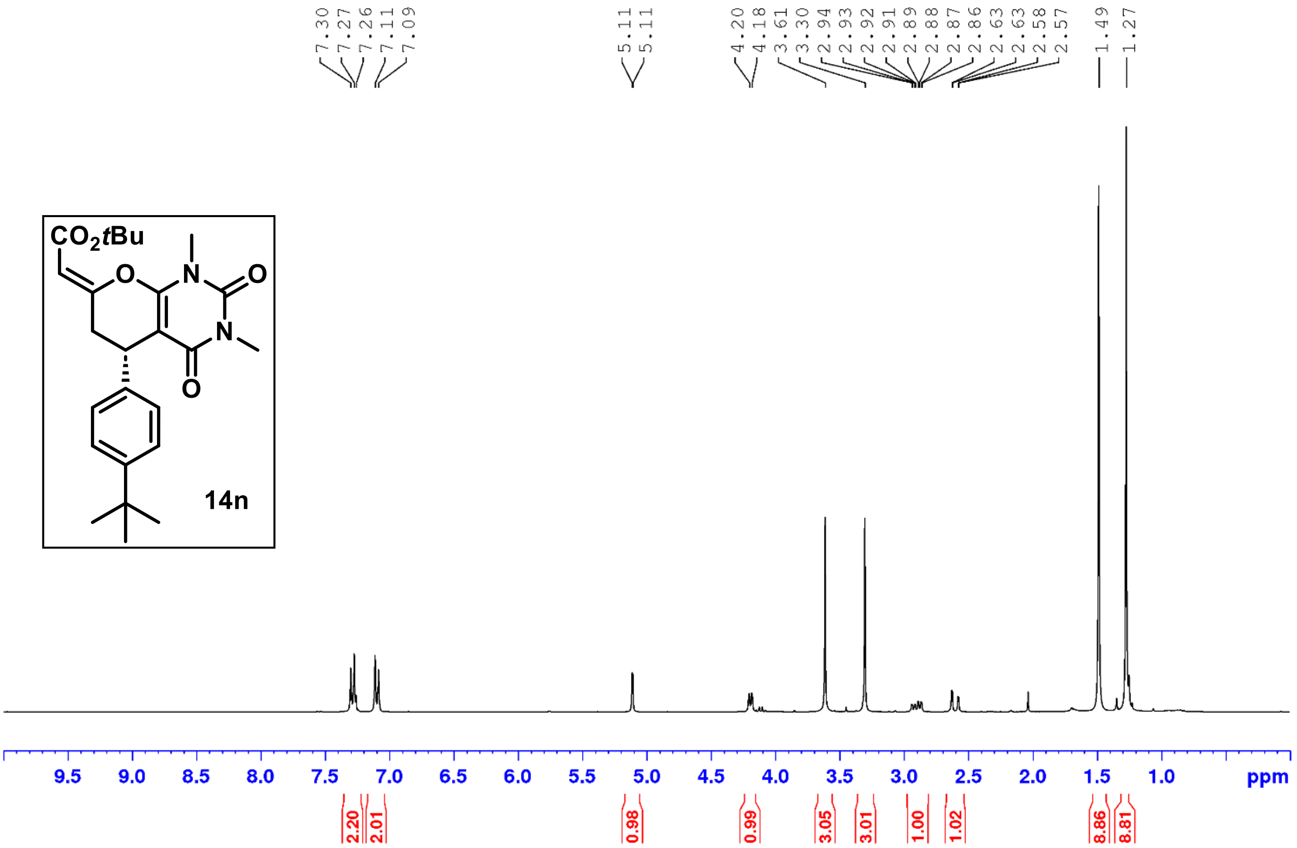
7.30 7.27 7.26 7.11 7.09
5.11 5.11
4.20 4.18 3.61 3.30 2.94 2.93 2.92 2.91 2.89 2.88 2.87 2.86 2.63 2.63 2.58 2.57
1.49 1.27
CO2tBu
O
N
O
N
O
14n
9.5 9.0 8.5 8.0 7.5 7.0 6.5 6.0 5.5 5.0 4.5 4.0 3.5 3.0 2.5 2.0 1.5 1.0 ppm
2.20 2.01 0.98 0.99 3.05 3.01 1.00 1.02 8.86 8.81

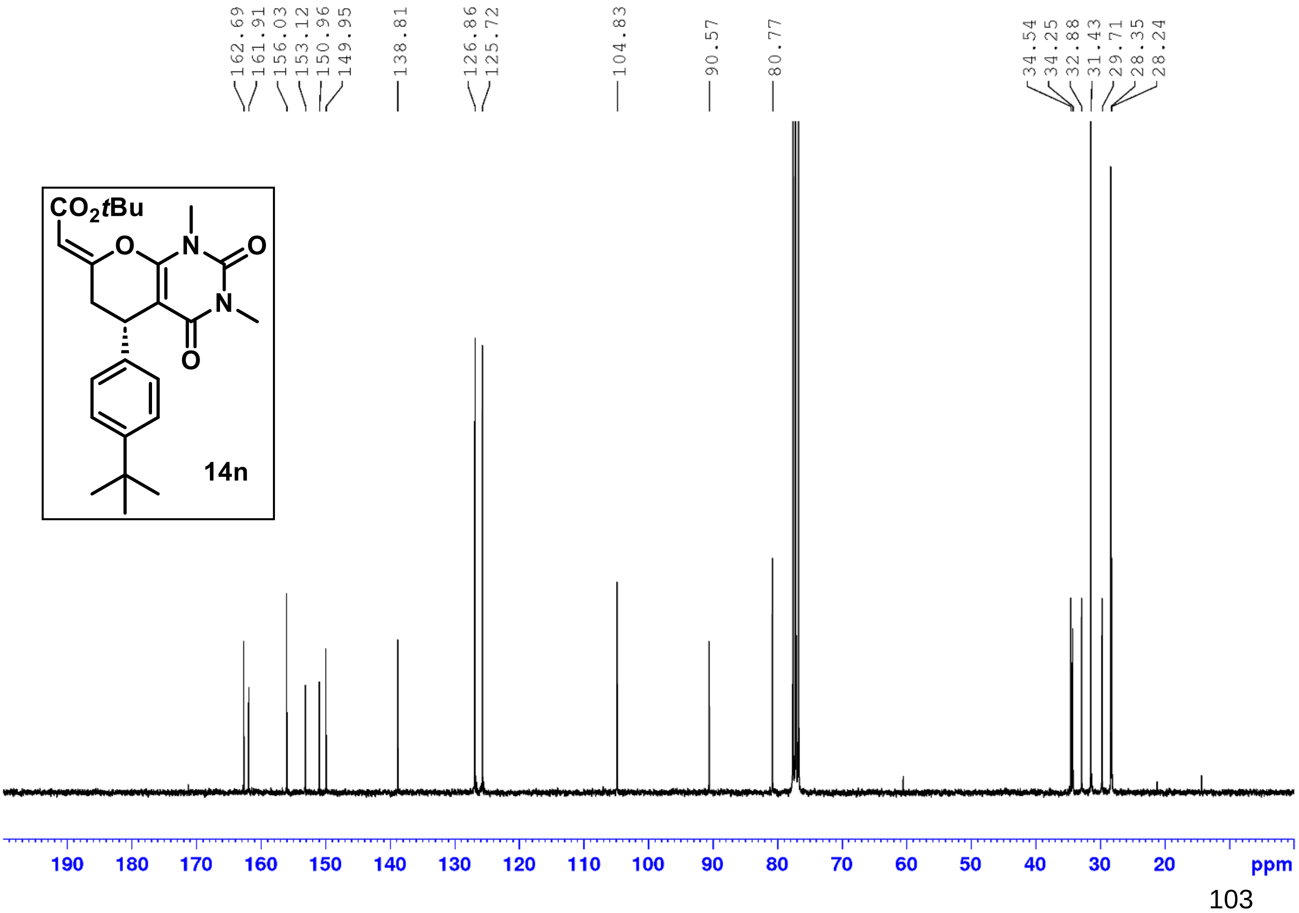
162.69 161.91 156.03 153.12 150.96 149.95
138.81
126.86 125.72
104.83
90.57
80.77
34.54 34.25 32.88 31.43 29.71 28.35 28.24
CO2tBu
O
N
O
N
O
14n
190 180 170 160 150 140 130 120 110 100 90 80 70 60 50 40 30 20 ppm

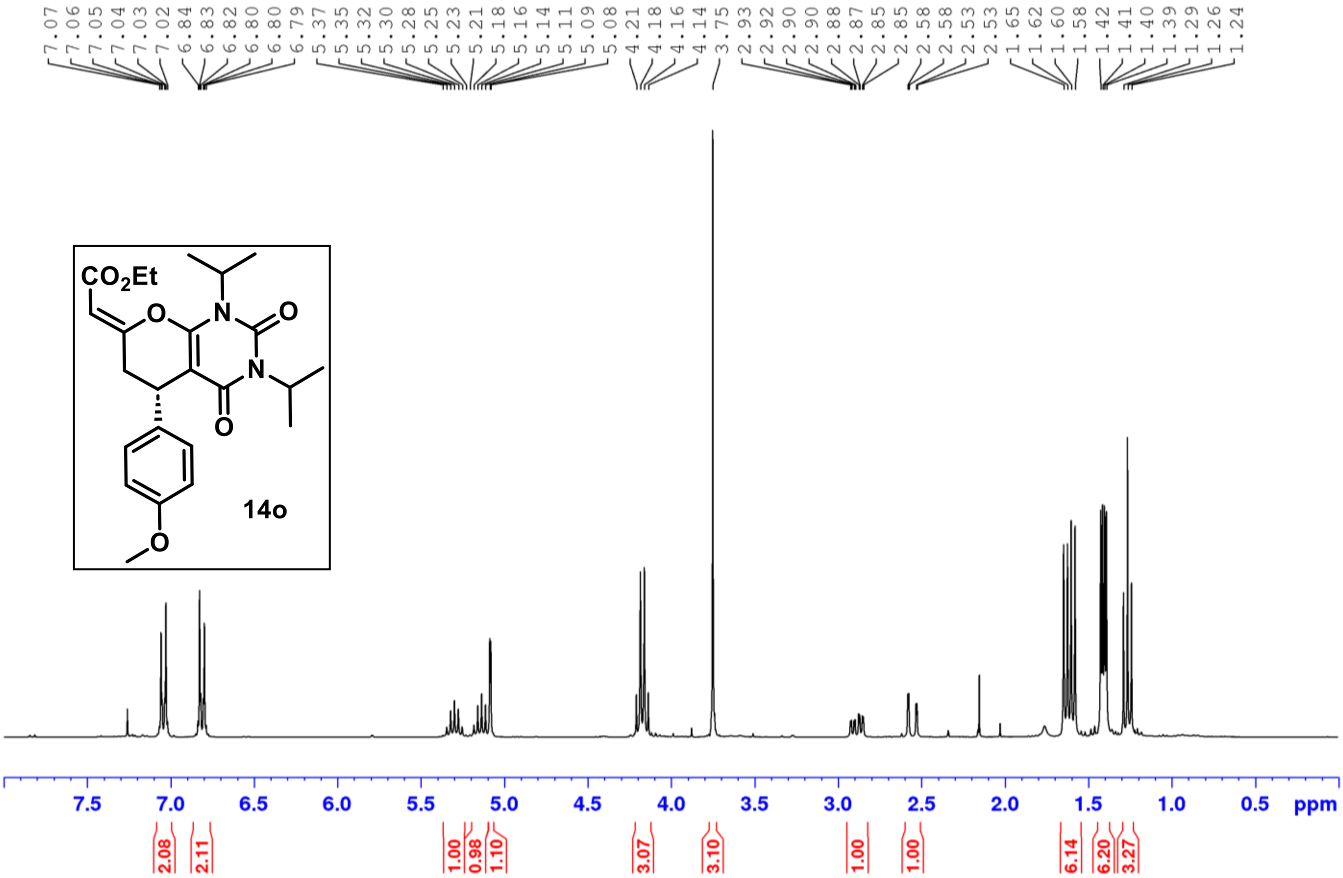
7.07 7.06 7.05 7.04 7.03 7.02 6.84 6.83 6.82 6.80 6.80 6.79 5.37 5.35 5.32 5.30 5.28 5.25 5.23 5.21 5.18 5.16 5.14 5.11 5.09 5.08 4.21 4.18 4.16 4.14 3.75 2.93 2.92 2.90 2.88 2.87 2.85 2.85 2.58 2.58 2.53 2.53 1.65 1.62 1.60 1.58 1.42 1.41 1.40 1.39 1.29 1.26 1.24
CO_2Et
O
N
O
N
O
14o
O
7.5 7.0 6.5 6.0 5.5 5.0 4.5 4.0 3.5 3.0 2.5 2.0 1.5 1.0 0.5 ppm
2.08 2.11 1.00 0.98 1.10 3.07 3.10 1.00 1.00 6.14 6.20 3.27

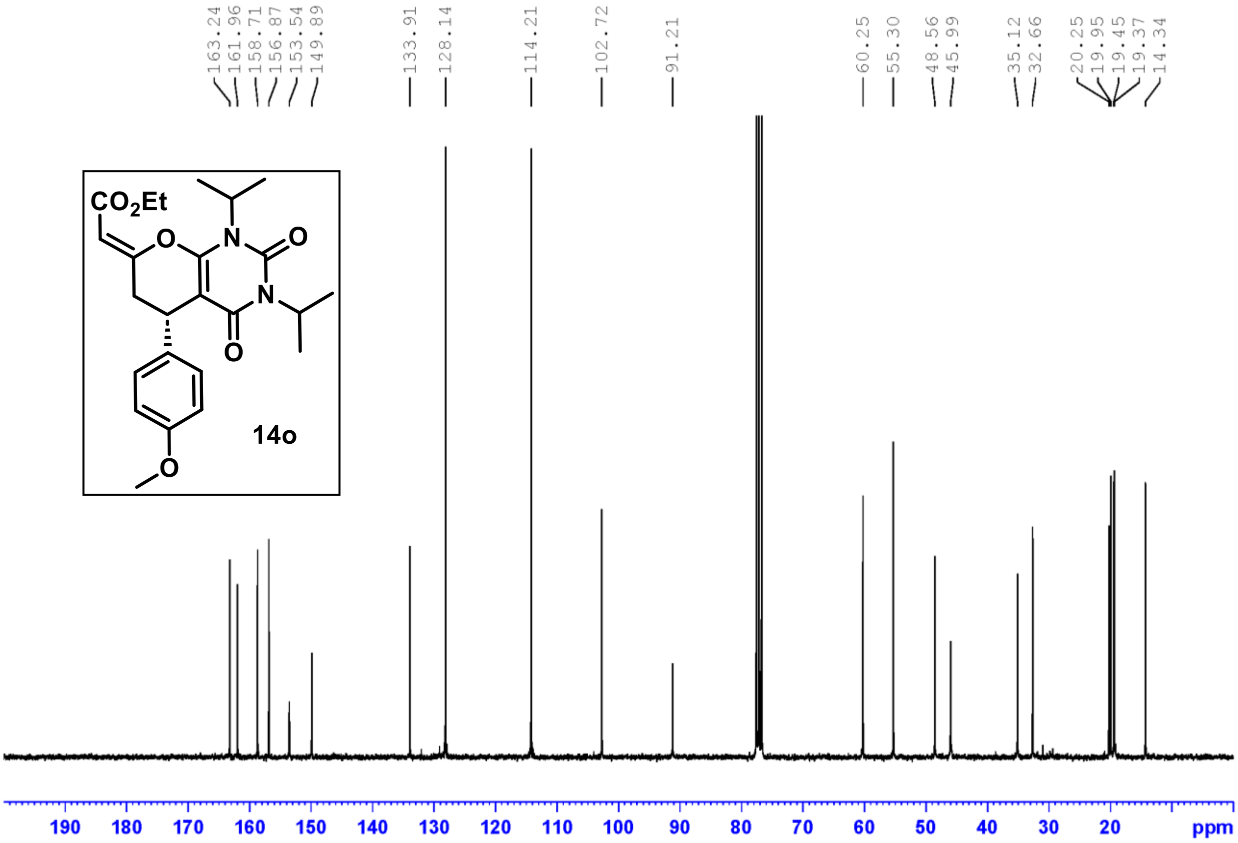
163.24 161.96 158.71 156.87 153.54 149.89 133.91 128.14 114.21 102.72 91.21 60.25 55.30 48.56 45.99 35.12 32.66 20.25 19.95 19.45 19.37 14.34
CO_2Et
O
N
O
N
O
14o
O
190 180 170 160 150 140 130 120 110 100 90 80 70 60 50 40 30 20 ppm

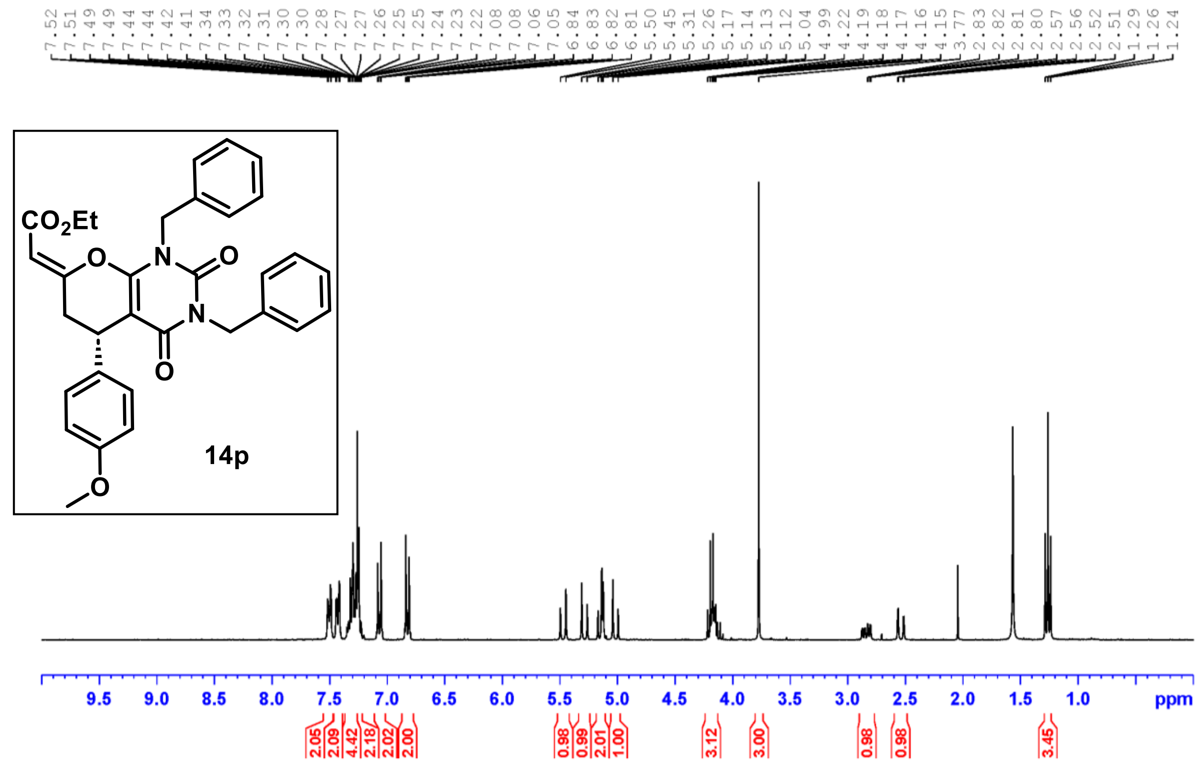
7.52 7.51 7.49 7.49 7.44 7.44 7.42 7.41 7.34 7.33 7.32 7.31 7.30 7.30 7.28 7.27 7.27 7.26 7.25 7.25 7.24 7.23 7.22 7.08 7.08 7.06 7.05 6.84 6.83 6.82 6.81 5.50 5.45 5.31 5.26 5.17 5.14 5.13 5.12 5.04 4.99 4.22 4.19 4.18 4.17 4.16 4.15 3.77 2.83 2.82 2.81 2.80 2.57 2.56 2.52 2.51 1.29 1.26 1.24
CO2Et
O
N
N
O
O
O
14p
9.5 9.0 8.5 8.0 7.5 7.0 6.5 6.0 5.5 5.0 4.5 4.0 3.5 3.0 2.5 2.0 1.5 1.0 ppm
2.05 2.09 4.42 2.18 2.02 2.00 0.98 0.99 2.01 1.00 3.12 3.00 0.98 0.98 3.45

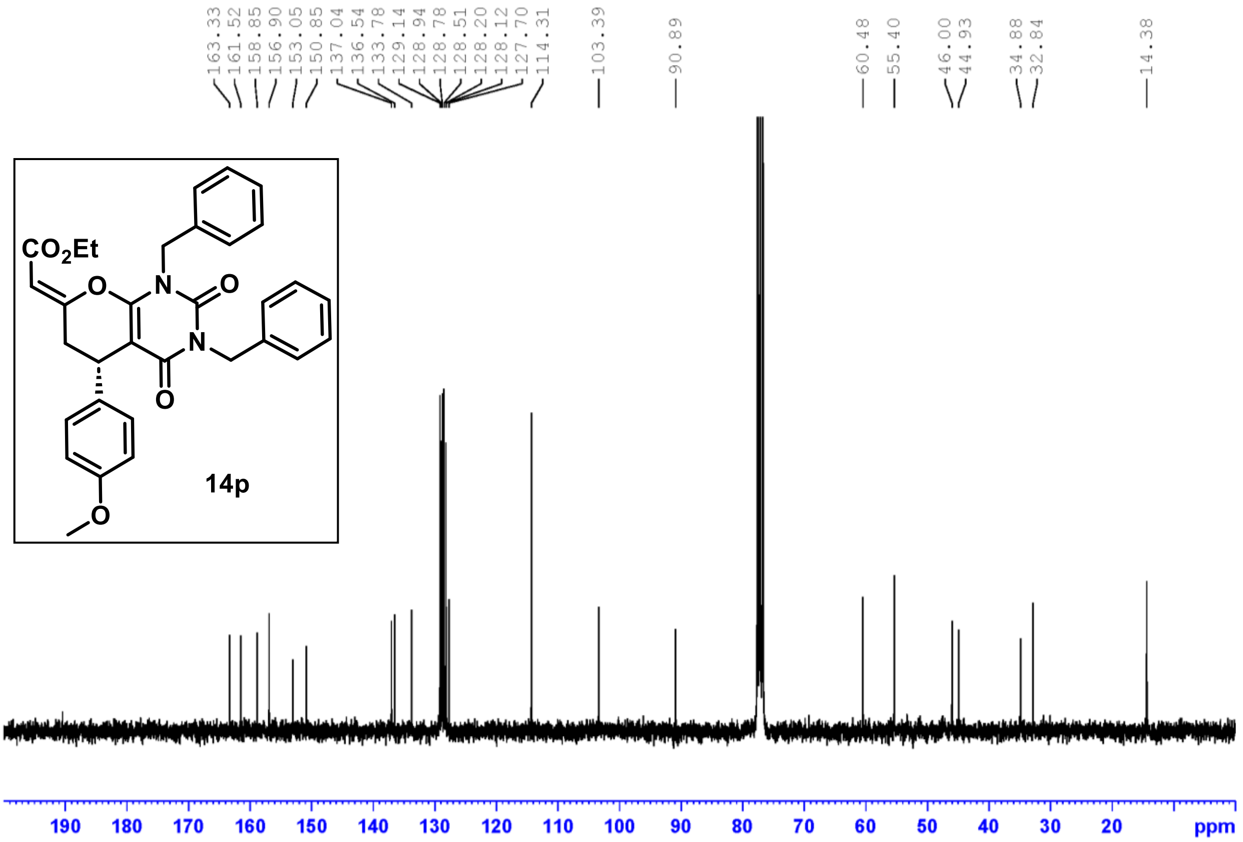
163.33 161.52 158.85 156.90 153.05 150.85 137.04 136.54 133.78 129.14 128.94 128.78 128.51 128.20 128.12 127.70 114.31 103.39 90.89 60.48 55.40 46.00 44.93 34.88 32.84 14.38
CO2Et
O
N
N
O
O
O
14p
190 180 170 160 150 140 130 120 110 100 90 80 70 60 50 40 30 20 ppm

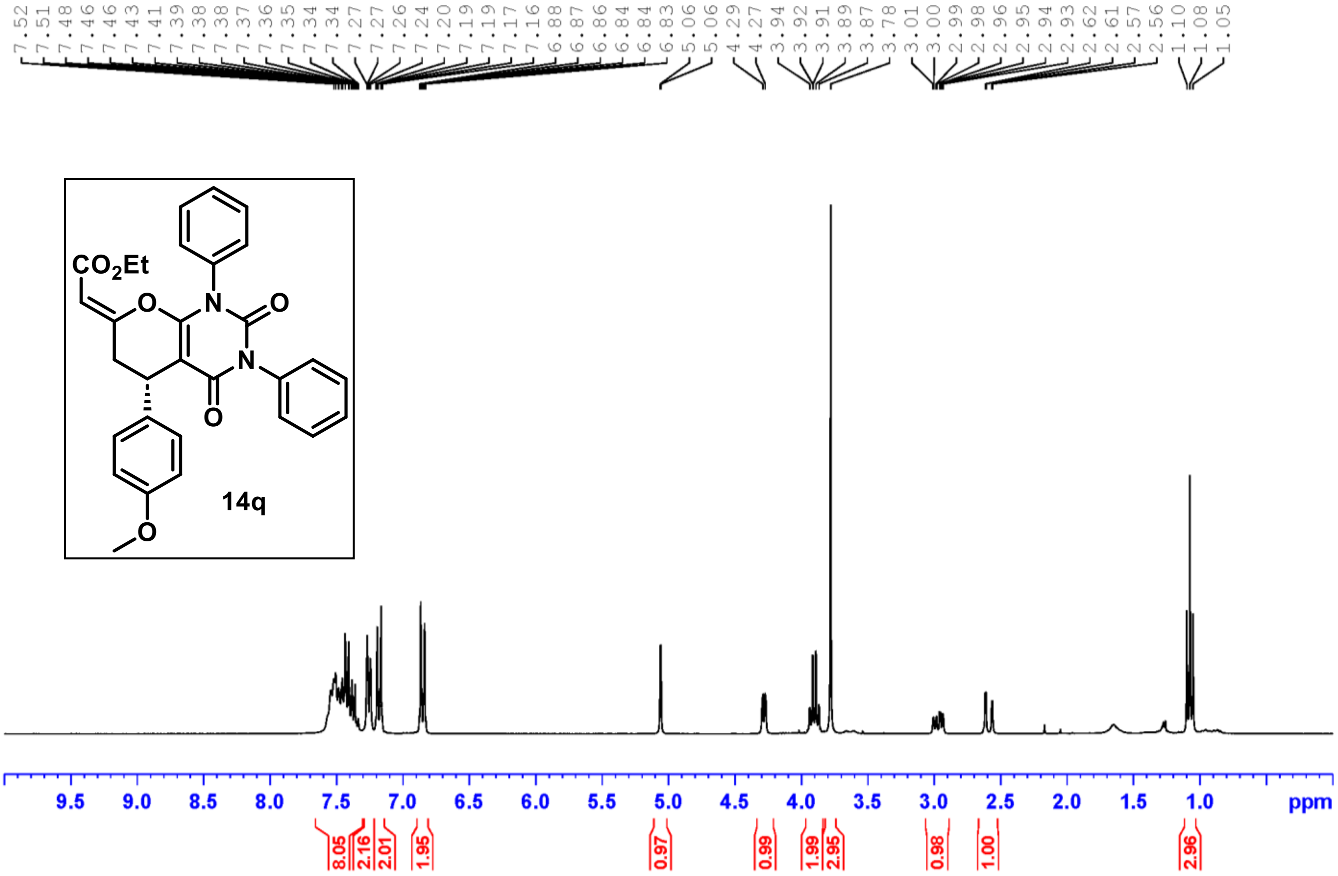
7.52 7.51 7.48 7.46 7.46 7.43 7.41 7.39 7.38 7.38 7.37 7.36 7.35 7.34 7.34 7.27 7.27 7.26 7.24 7.20 7.19 7.19 7.17 7.16 6.88 6.87 6.86 6.84 6.83 5.06 5.06 4.29 4.27 3.94 3.92 3.91 3.89 3.87 3.78 3.01 3.00 2.99 2.98 2.96 2.95 2.94 2.93 2.62 2.61 2.57 2.56 1.10 1.08 1.05
CO2Et
N
O
N
O
O
O
14q
9.5 9.0 8.5 8.0 7.5 7.0 6.5 6.0 5.5 5.0 4.5 4.0 3.5 3.0 2.5 2.0 1.5 1.0 ppm
8.05 2.16 2.01 1.95 0.97 0.99 1.99 2.95 0.98 1.00 2.96

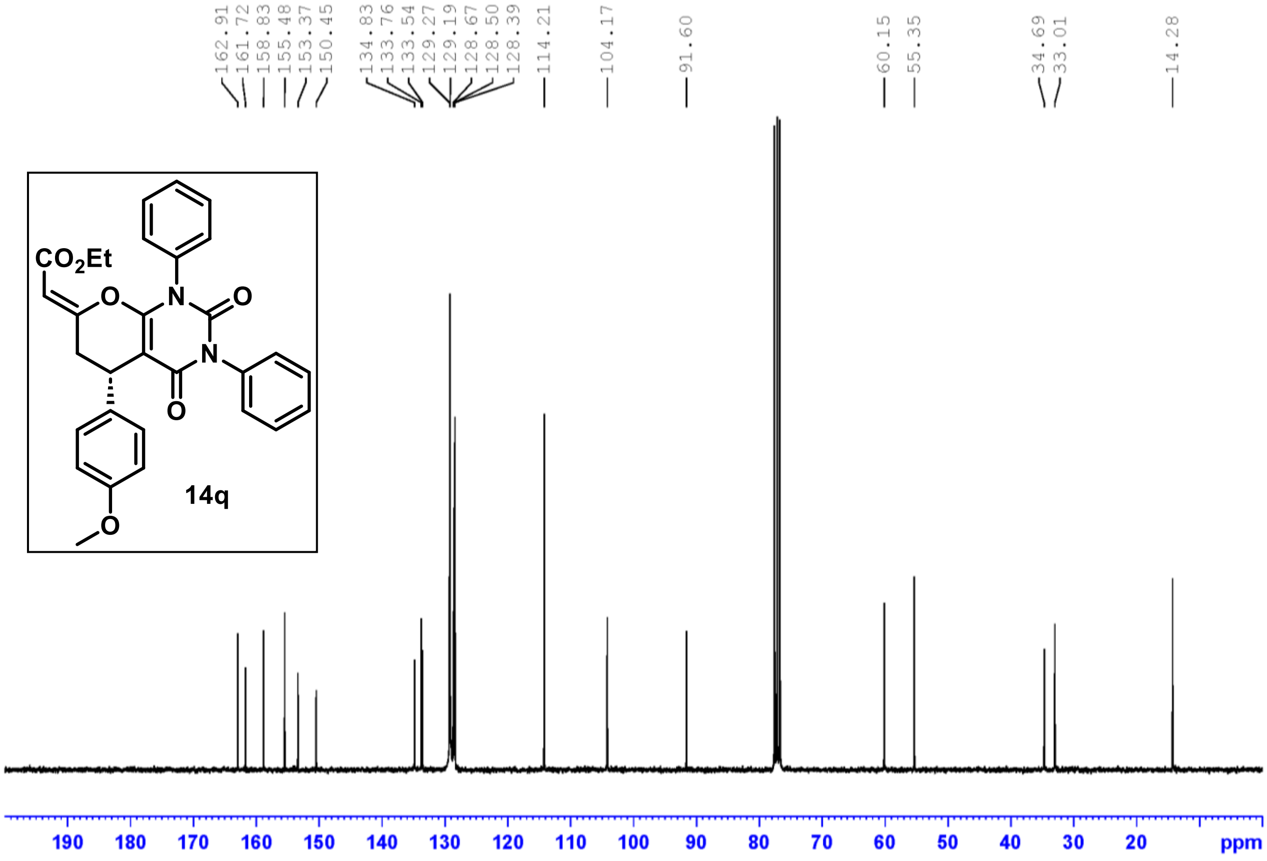
162.91 161.72 158.83 155.48 153.37 150.45 134.83 133.76 133.54 129.27 129.19 128.67 128.50 128.39 114.21 104.17 91.60 60.15 55.35 34.69 33.01 14.28
CO2Et
N
O
N
O
O
O
14q
190 180 170 160 150 140 130 120 110 100 90 80 70 60 50 40 30 20 ppm

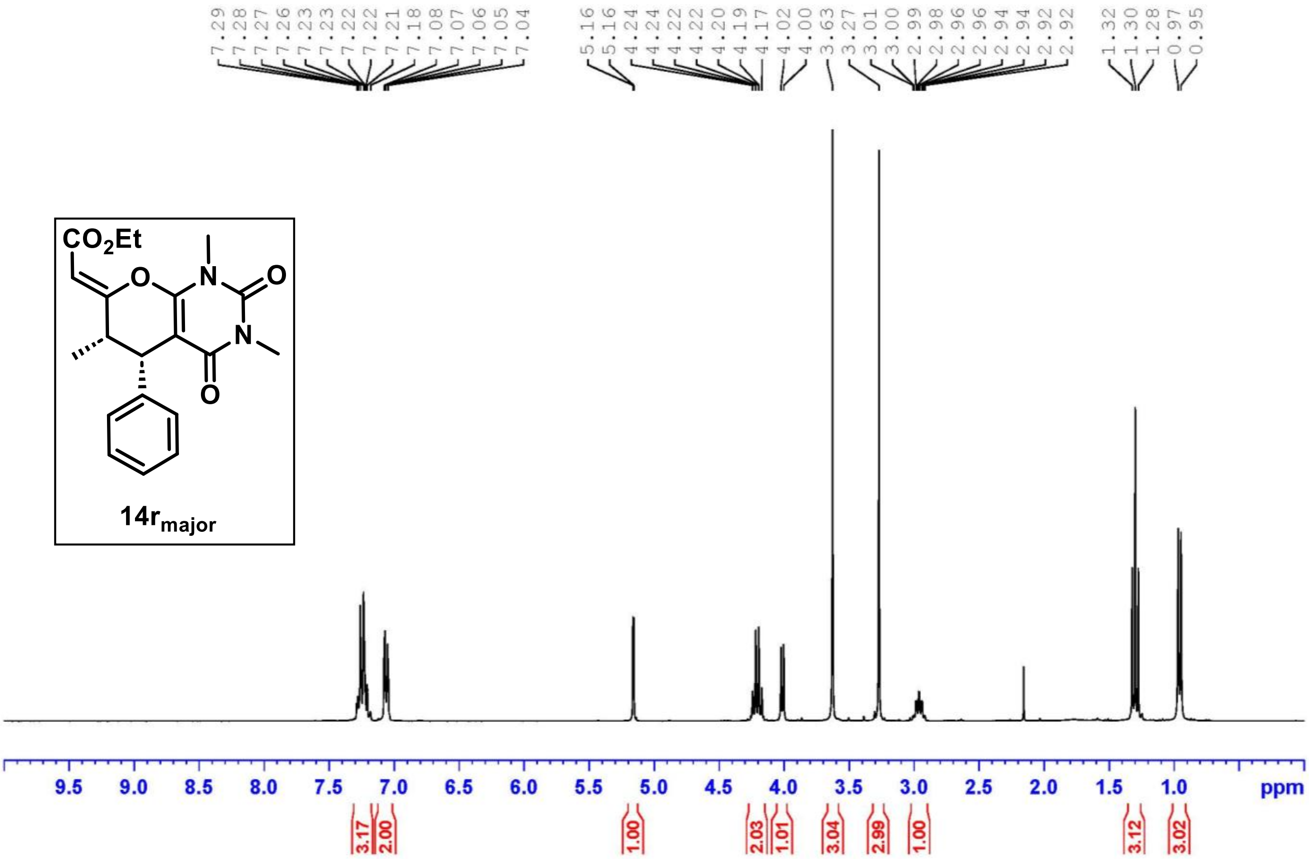
7.29 7.28 7.27 7.26 7.23 7.23 7.22 7.22 7.21 7.18 7.08 7.07 7.06 7.05 7.04
5.16 5.16 4.24 4.24 4.22 4.22 4.20 4.19 4.17 4.02 4.00 3.63 3.27 3.01 3.00 2.99 2.98 2.96 2.96 2.94 2.94 2.92 2.92
1.32 1.30 1.28 0.97 0.95
CO2Et
O
N
N
O
O
14rmajor
9.5 9.0 8.5 8.0 7.5 7.0 6.5 6.0 5.5 5.0 4.5 4.0 3.5 3.0 2.5 2.0 1.5 1.0 ppm
3.17 2.00 1.00 2.03 1.01 3.04 2.99 1.00 3.12 3.02

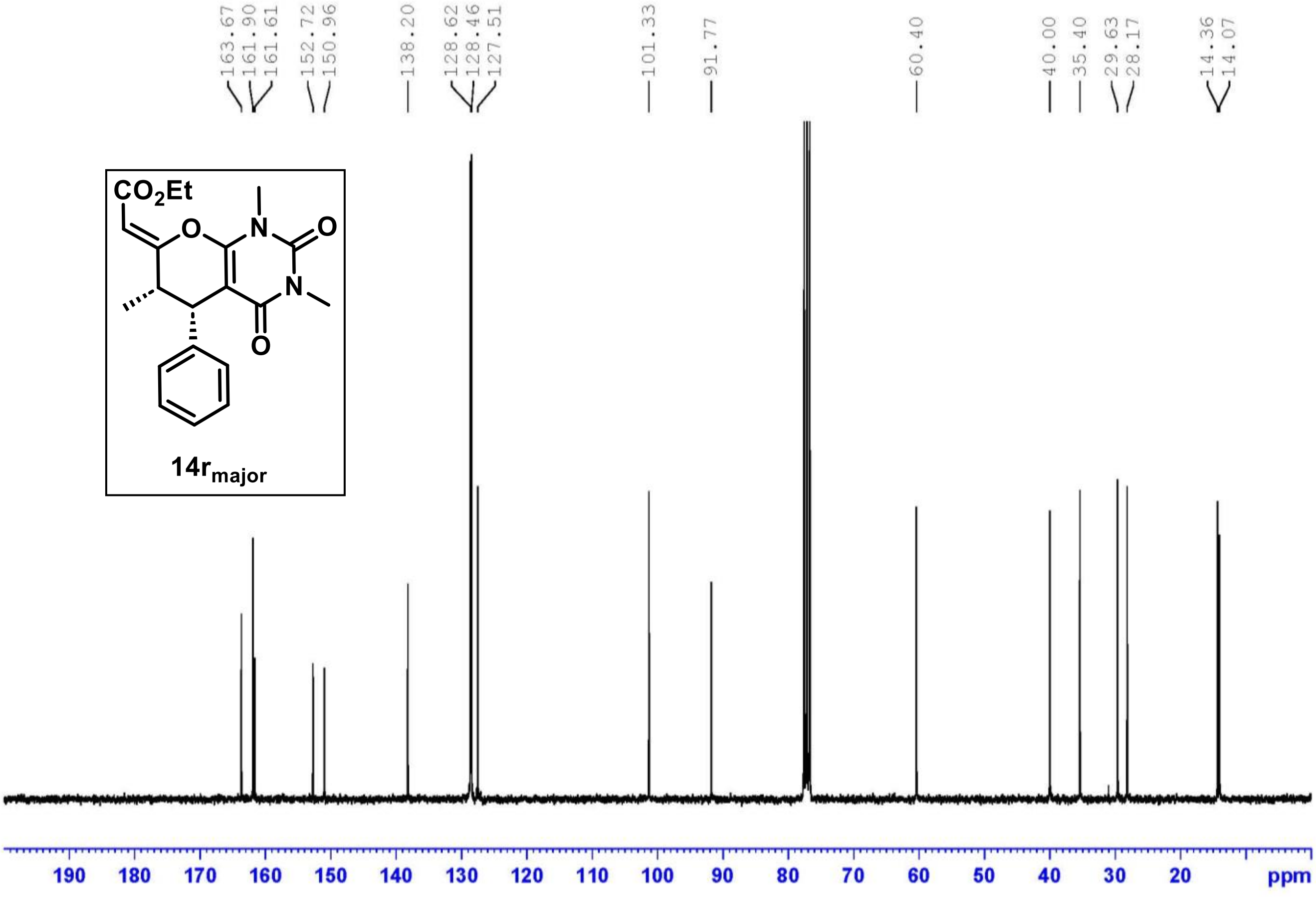
163.67 161.90 161.61 152.72 150.96 138.20 128.62 128.46 127.51 101.33 91.77 60.40 40.00 35.40 29.63 28.17 14.36 14.07
CO2Et
O
N
N
O
O
14rmajor
190 180 170 160 150 140 130 120 110 100 90 80 70 60 50 40 30 20 ppm

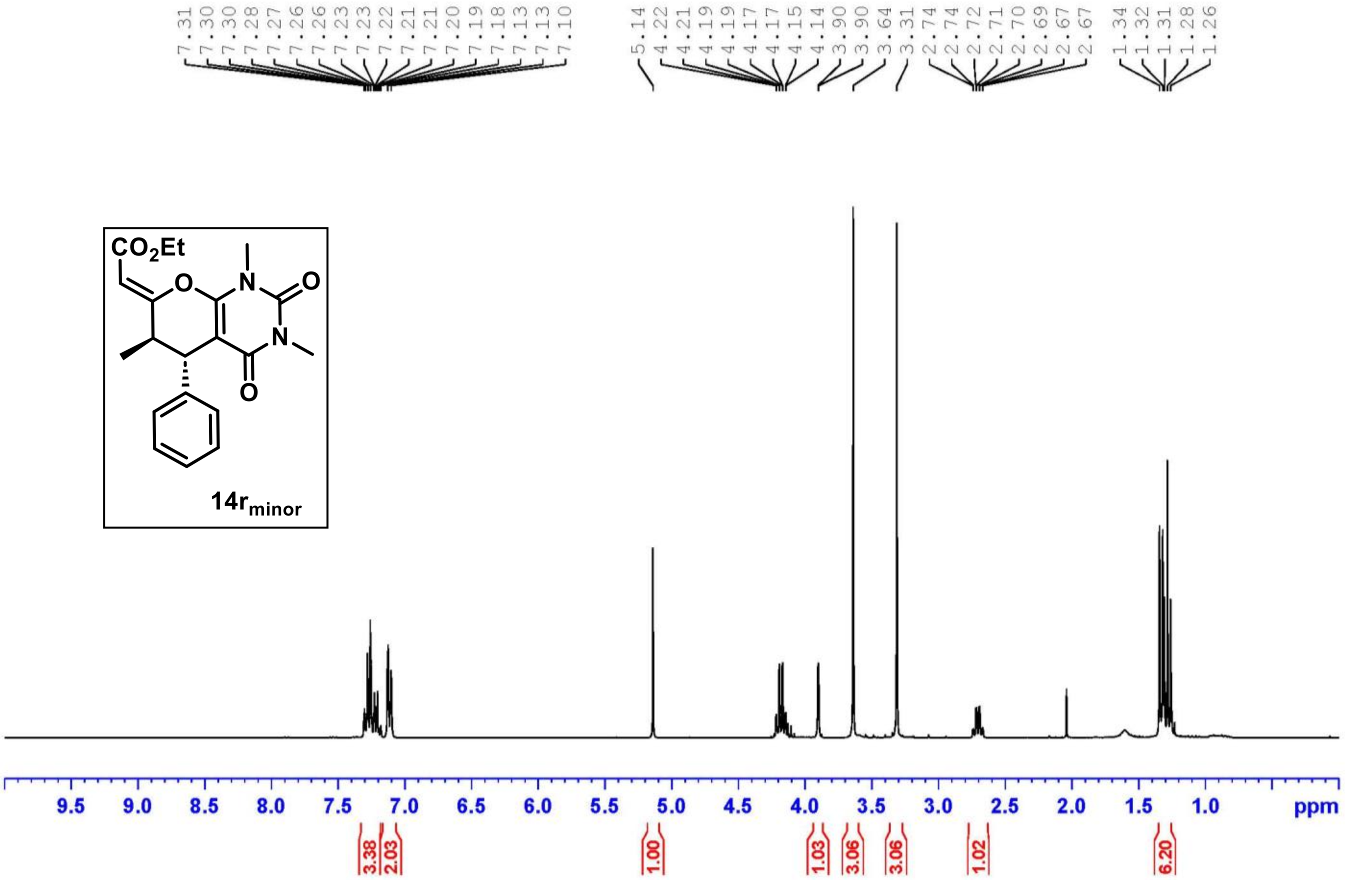
7.31 7.30 7.30 7.28 7.27 7.26 7.26 7.23 7.23 7.22 7.21 7.21 7.20 7.19 7.18 7.13 7.13 7.10
5.14 4.22 4.21 4.19 4.19 4.17 4.17 4.15 4.14 3.90 3.64 3.31 2.74 2.74 2.72 2.71 2.70 2.69 2.67 2.67 1.34 1.32 1.31 1.28 1.26
CO2Et
O
N
O
N
O
14rminor
9.5 9.0 8.5 8.0 7.5 7.0 6.5 6.0 5.5 5.0 4.5 4.0 3.5 3.0 2.5 2.0 1.5 1.0 ppm
3.38 2.03 1.00 1.03 3.06 3.06 1.02 6.20

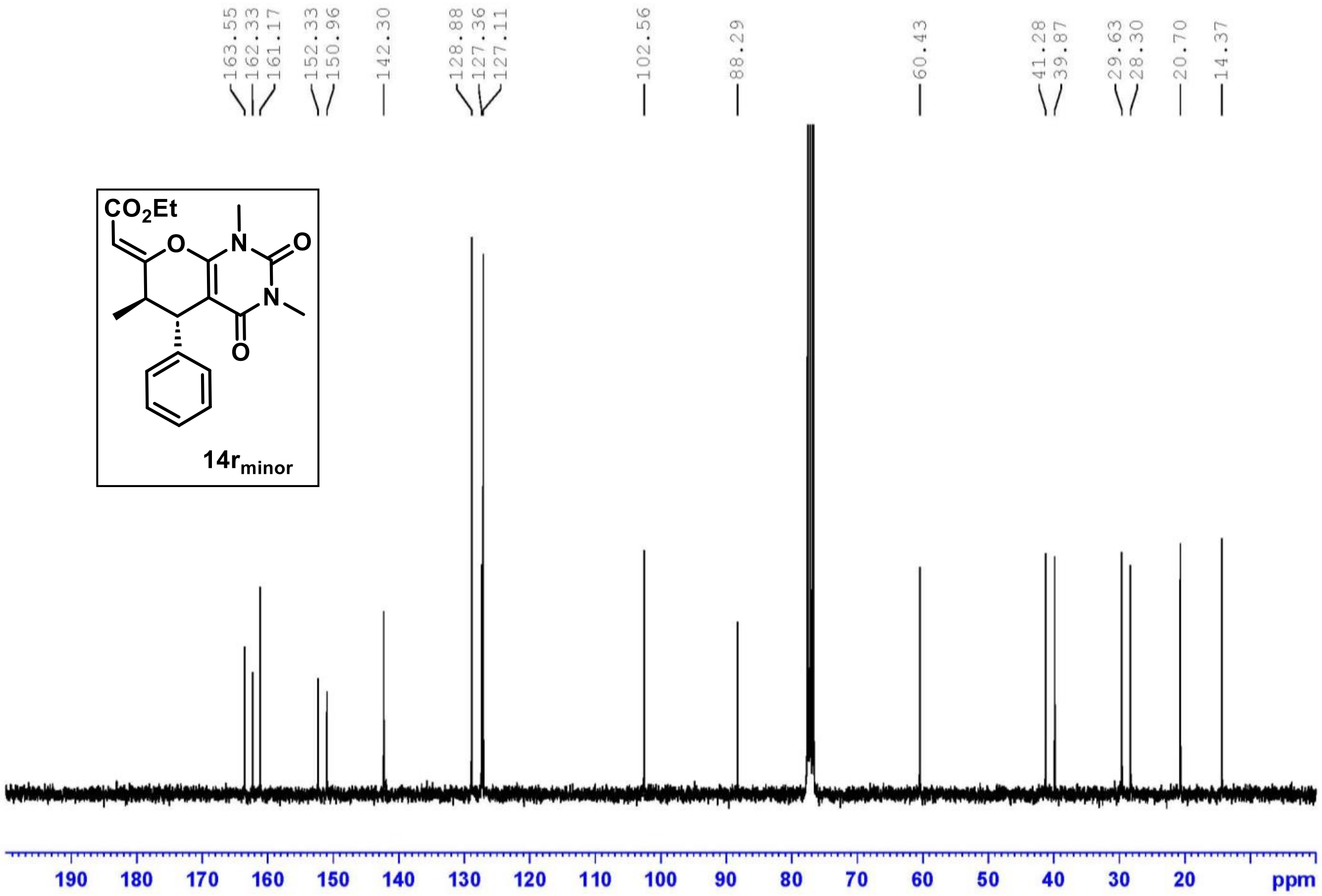
163.55 162.33 161.17 152.33 150.96 142.30 128.88 127.36 127.11 102.56 88.29 60.43 41.28 39.87 29.63 28.30 20.70 14.37
CO2Et
O
N
O
N
O
14rminor
190 180 170 160 150 140 130 120 110 100 90 80 70 60 50 40 30 20 ppm

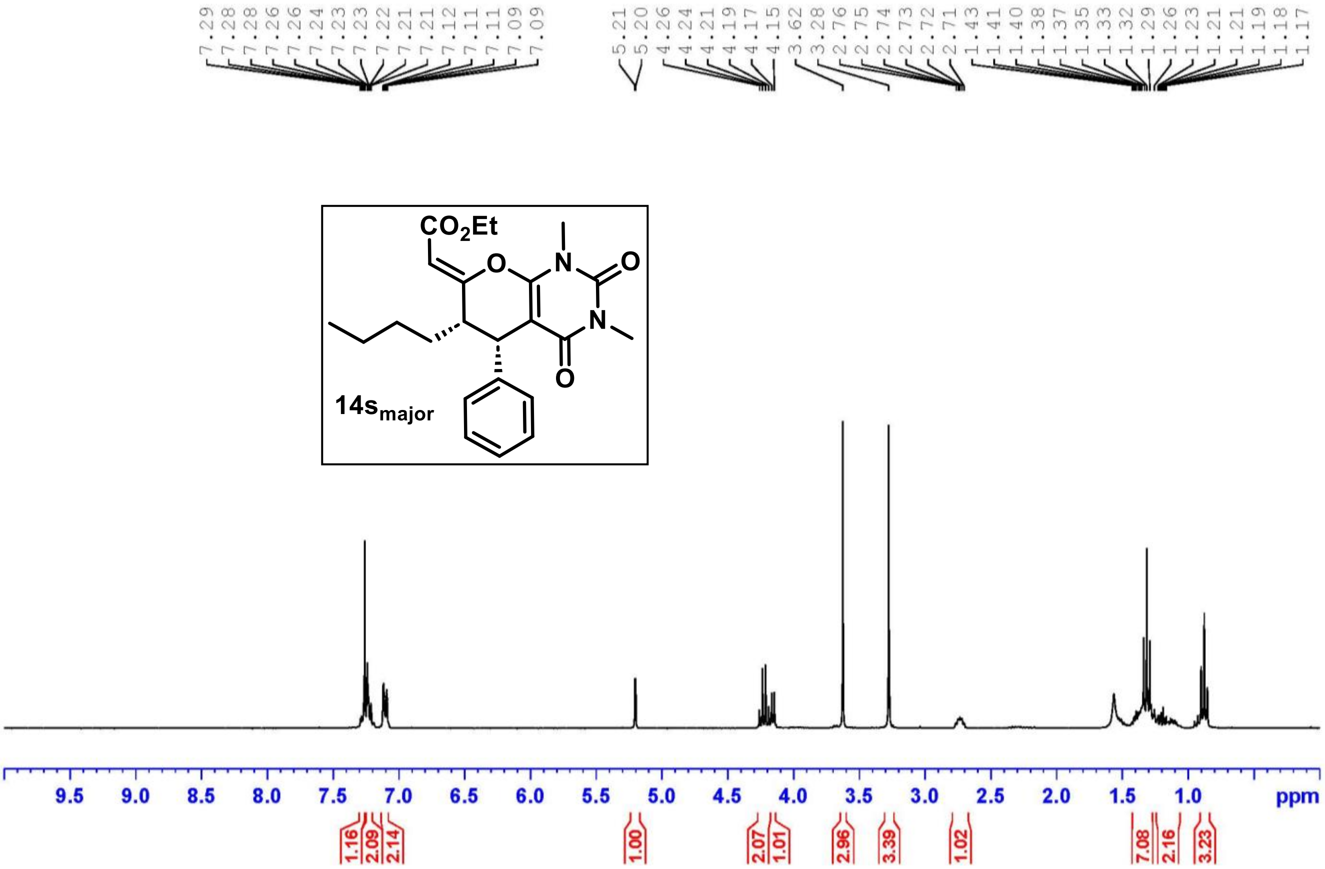
7.29 7.28 7.28 7.26 7.26 7.24 7.23 7.23 7.22 7.21 7.21 7.12 7.11 7.11 7.09 7.09
5.21 5.20 4.26 4.24 4.21 4.19 4.17 4.15 3.62 3.28 2.76 2.75 2.74 2.73 2.72 2.71 1.43 1.41 1.40 1.38 1.37 1.35 1.33 1.32 1.29 1.26 1.23 1.21 1.21 1.19 1.18 1.17
CO2Et
N
O
O
N
O
O
14s major
9.5 9.0 8.5 8.0 7.5 7.0 6.5 6.0 5.5 5.0 4.5 4.0 3.5 3.0 2.5 2.0 1.5 1.0 ppm
1.16 2.09 2.14 1.00 2.07 1.01 2.96 3.39 1.02 7.08 2.16 3.23

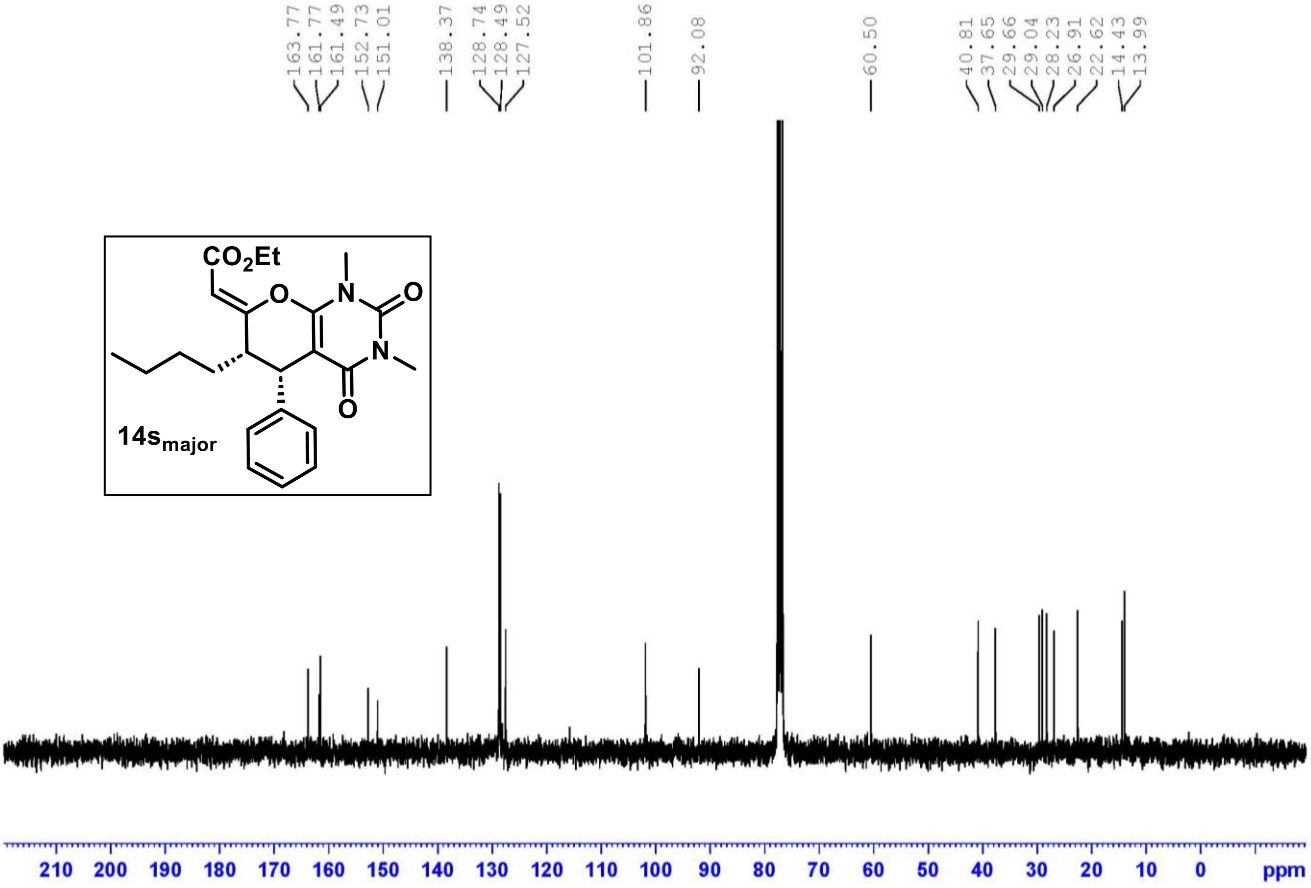
163.77 161.77 161.49 152.73 151.01 138.37 128.74 128.49 127.52 101.86 92.08 60.50 40.81 37.65 29.66 29.04 28.23 26.91 22.62 14.43 13.99
CO2Et
N
O
O
N
O
O
14s major
210 200 190 180 170 160 150 140 130 120 110 100 90 80 70 60 50 40 30 20 10 0 ppm

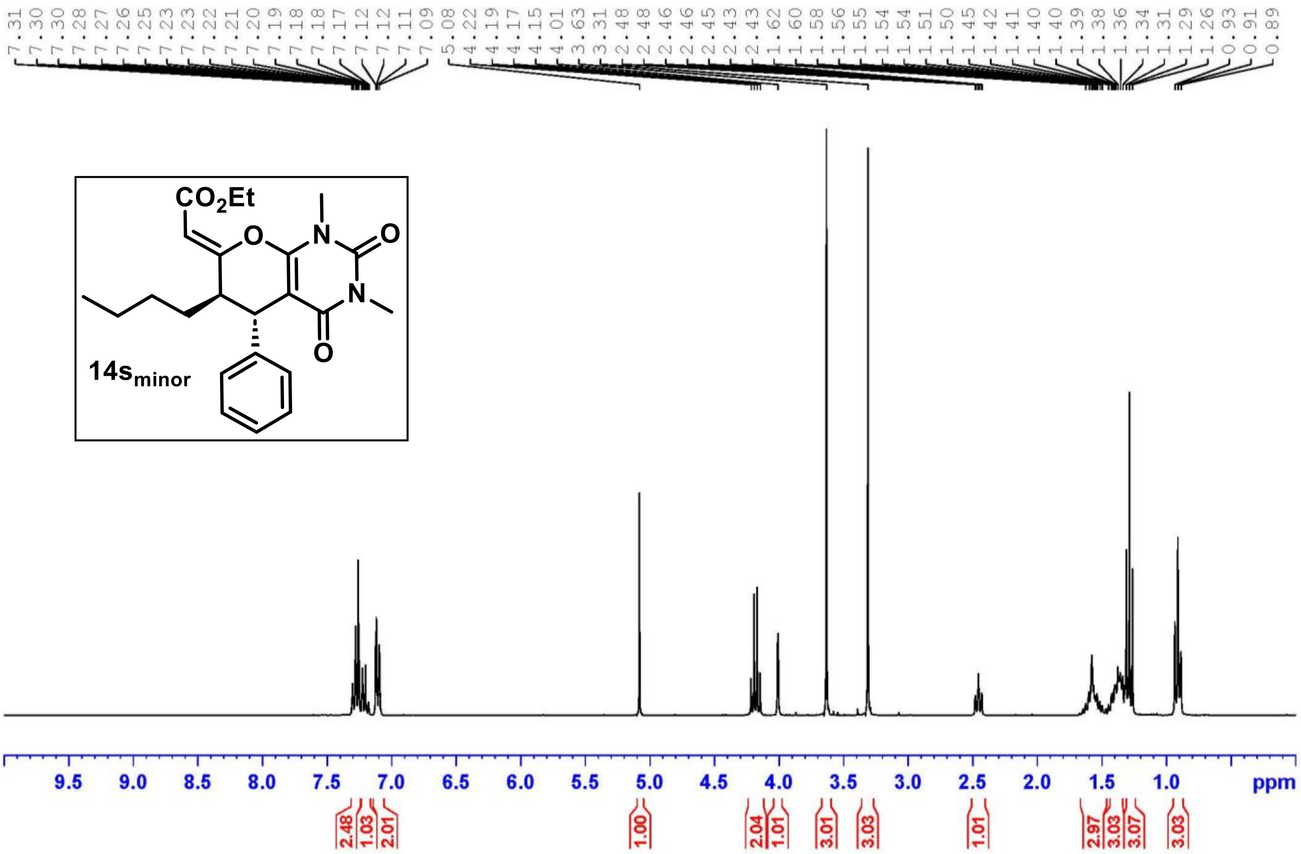
7.31 7.30 7.30 7.28 7.27 7.26 7.25 7.23 7.23 7.22 7.21 7.20 7.19 7.18 7.18 7.17 7.12 7.12 7.11 7.09 5.08 4.22 4.19 4.17 4.15 4.01 3.63 3.31 2.48 2.48 2.46 2.45 2.43 2.43 1.62 1.60 1.58 1.56 1.55 1.54 1.54 1.51 1.50 1.45 1.42 1.41 1.40 1.40 1.39 1.38 1.36 1.34 1.31 1.29 1.26 0.93 0.91 0.89
CO2Et
N
O
N
O
O
14s minor
9.5 9.0 8.5 8.0 7.5 7.0 6.5 6.0 5.5 5.0 4.5 4.0 3.5 3.0 2.5 2.0 1.5 1.0 ppm
2.48 1.03 2.01 1.00 2.04 1.01 3.01 3.03 1.01 2.97 3.03 3.07 3.03

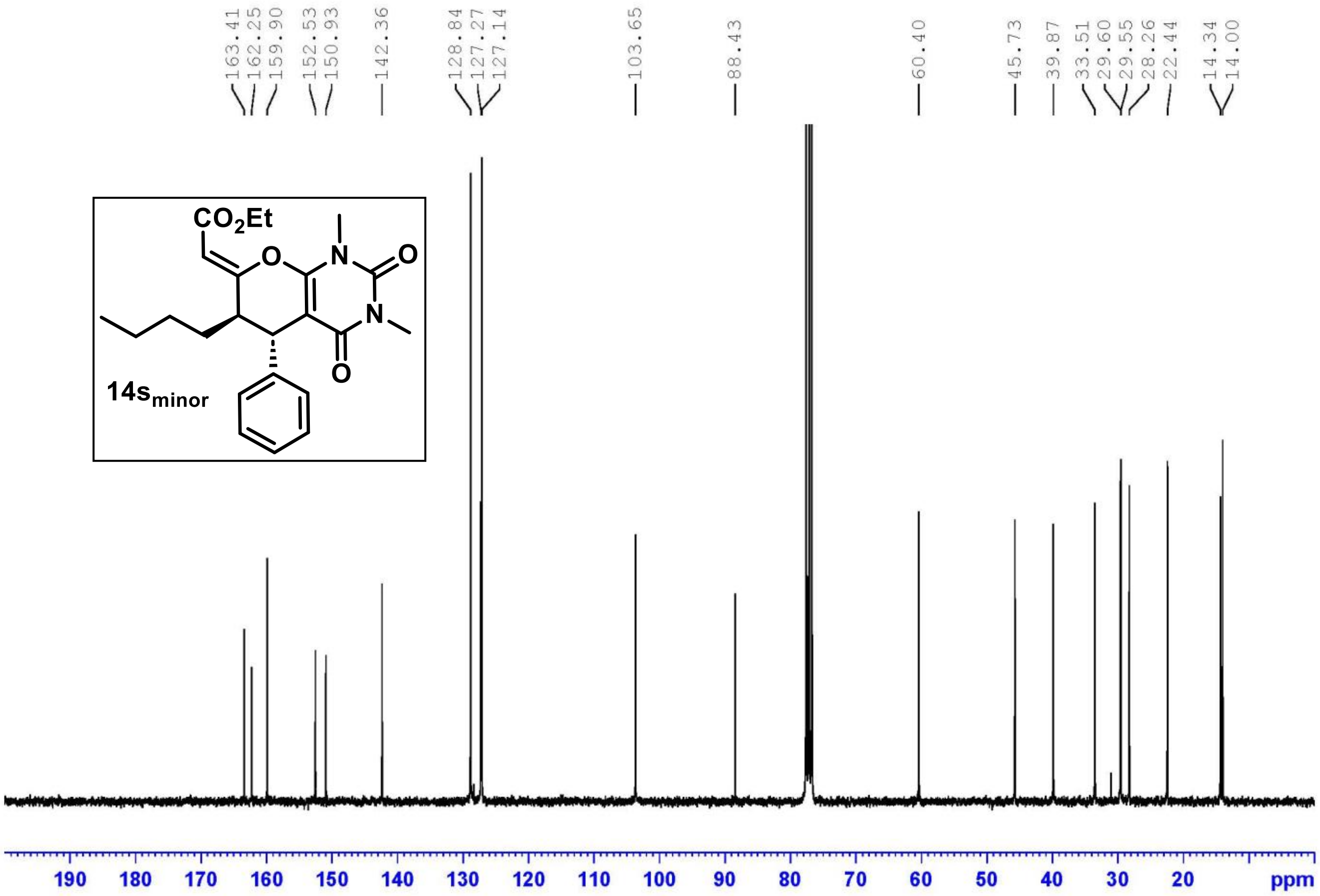
163.41 162.25 159.90 152.53 150.93 142.36 128.84 127.27 127.14 103.65 88.43 60.40 45.73 39.87 33.51 29.60 29.55 28.26 22.44 14.34 14.00
CO2Et
N
O
N
O
O
14s minor
190 180 170 160 150 140 130 120 110 100 90 80 70 60 50 40 30 20 ppm

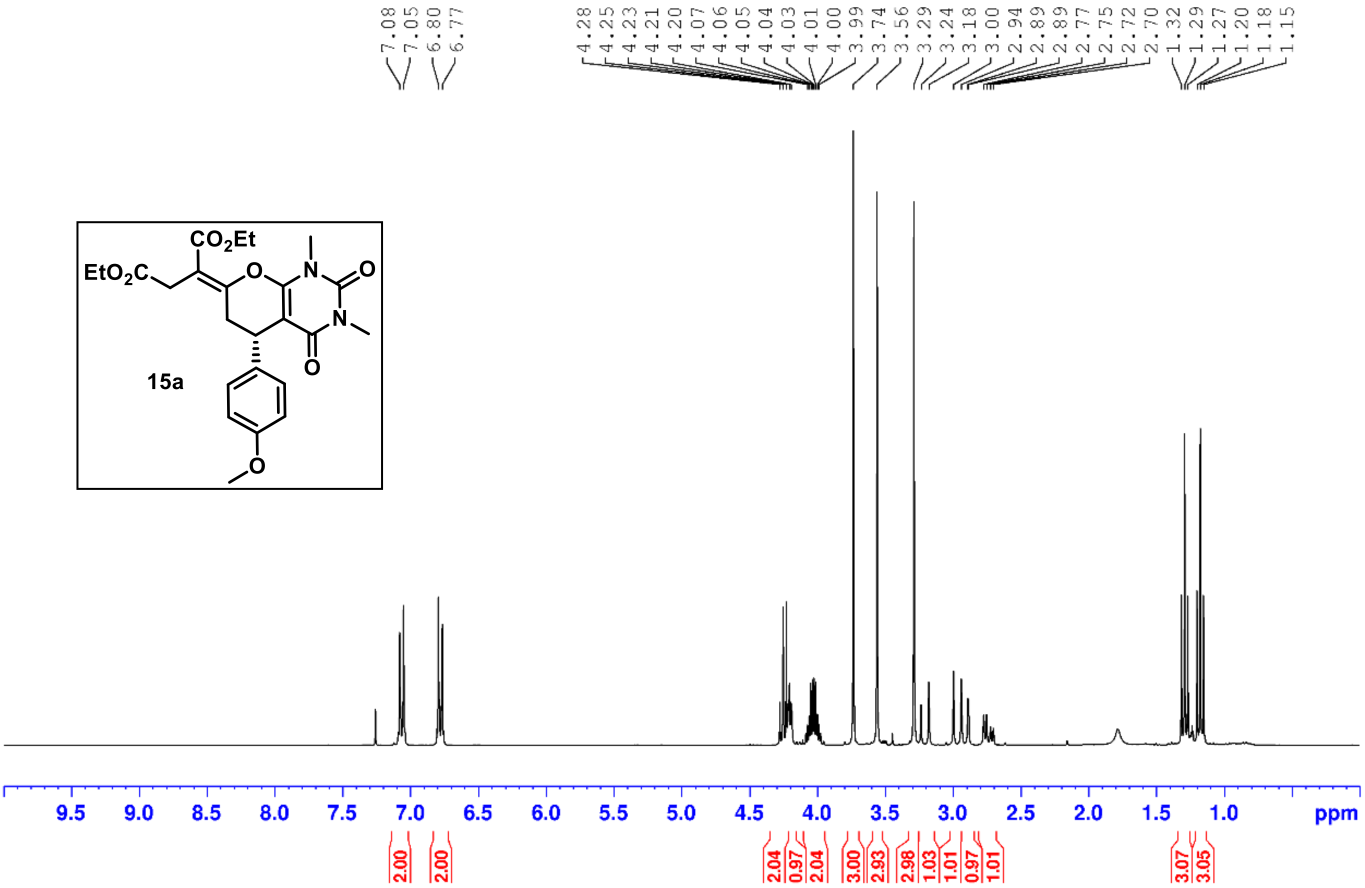
7.08 7.05 6.80 6.77
4.28 4.25 4.23 4.21 4.20 4.07 4.06 4.05 4.04 4.03 4.01 4.00 3.99 3.74 3.56 3.29 3.24 3.18 3.00 2.94 2.89 2.89 2.77 2.75 2.72 2.70 1.32 1.29 1.27 1.20 1.18 1.15
CO2Et
EtO2C
O
N
O
N
O
O
15a
9.5 9.0 8.5 8.0 7.5 7.0 6.5 6.0 5.5 5.0 4.5 4.0 3.5 3.0 2.5 2.0 1.5 1.0 ppm
2.00 2.00 2.04 0.97 2.04 3.00 2.93 2.98 1.03 1.01 0.97 1.01 3.07 3.05

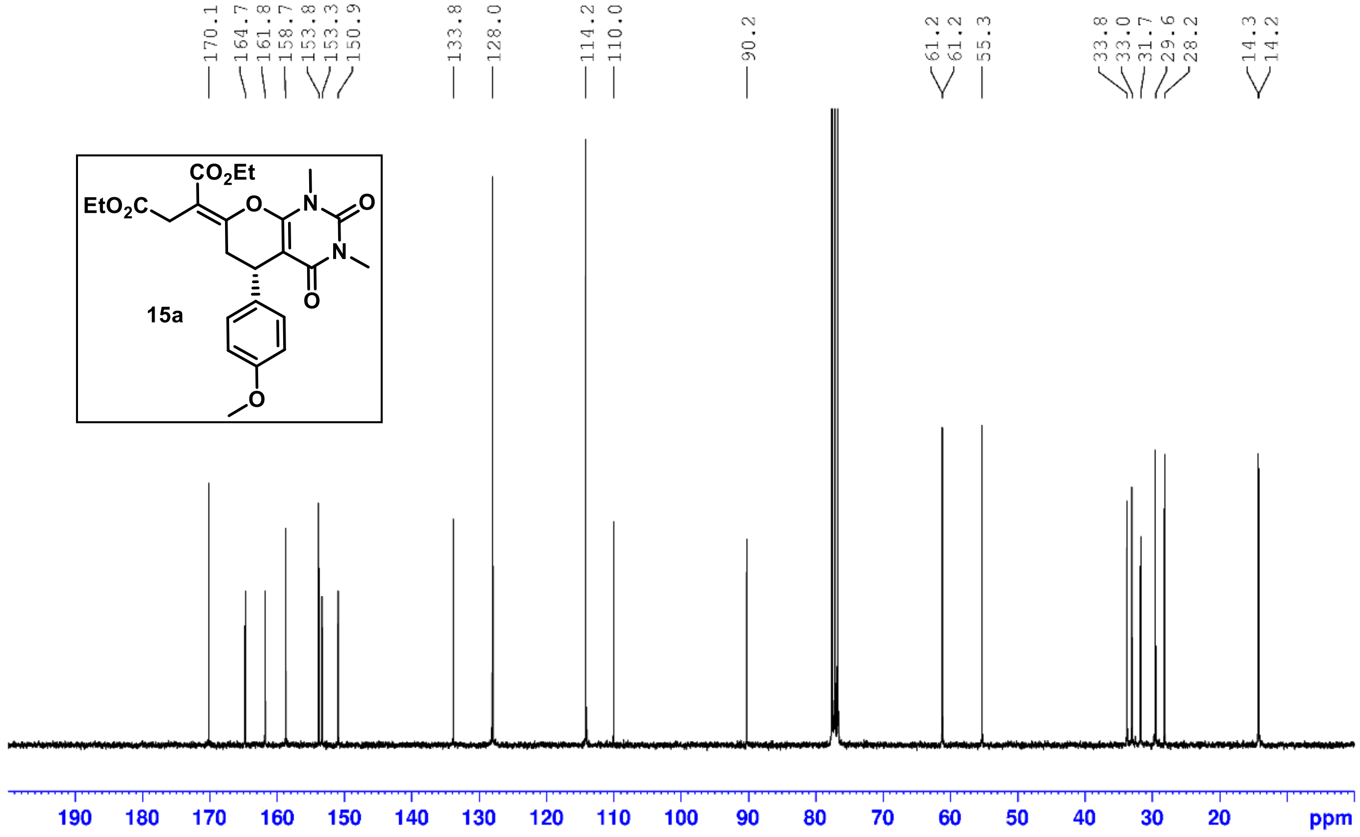
170.1 164.7 161.8 158.7 153.8 153.3 150.9 133.8 128.0 114.2 110.0 90.2 61.2 61.2 55.3 33.8 33.0 31.7 29.6 28.2 14.3 14.2
CO2Et
EtO2C
O
N
O
N
O
O
15a
190 180 170 160 150 140 130 120 110 100 90 80 70 60 50 40 30 20 ppm

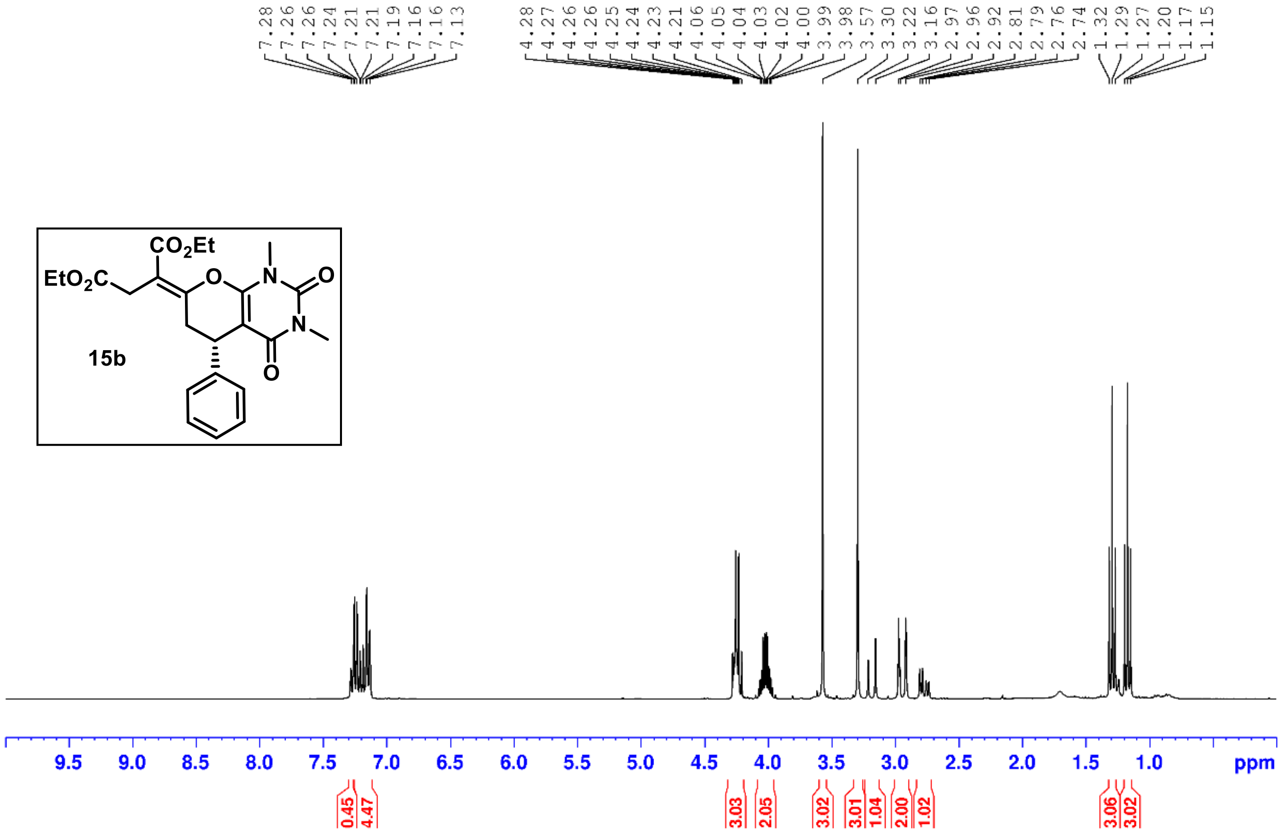
7.28 7.26 7.26 7.24 7.21 7.21 7.19 7.16 7.16 7.13
4.28 4.27 4.26 4.26 4.25 4.24 4.24 4.23 4.21 4.06 4.05 4.04 4.03 4.02 4.00 3.99 3.98 3.57 3.30 3.22 3.16 2.97 2.96 2.92 2.81 2.79 2.76 2.74 1.32 1.29 1.27 1.20 1.17 1.15
CO2Et
EtO2C
O
N
O
N
O
15b
9.5 9.0 8.5 8.0 7.5 7.0 6.5 6.0 5.5 5.0 4.5 4.0 3.5 3.0 2.5 2.0 1.5 1.0 ppm
0.45 4.47 3.03 2.05 3.02 3.01 1.04 2.00 1.02 3.06 3.02

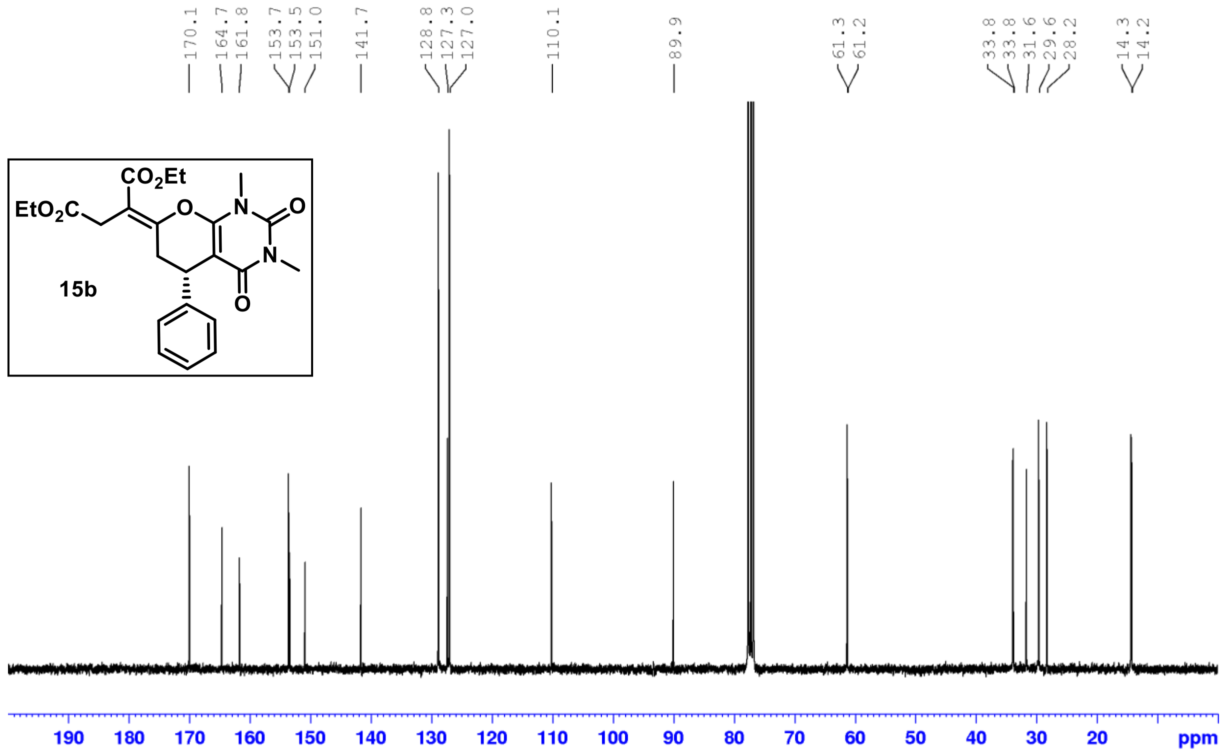
170.1 164.7 161.8 153.7 153.5 151.0 141.7 128.8 127.3 127.0 110.1 89.9 61.3 61.2 33.8 33.8 31.6 29.6 28.2 14.3 14.2
CO2Et
EtO2C
O
N
O
N
O
15b
190 180 170 160 150 140 130 120 110 100 90 80 70 60 50 40 30 20 ppm

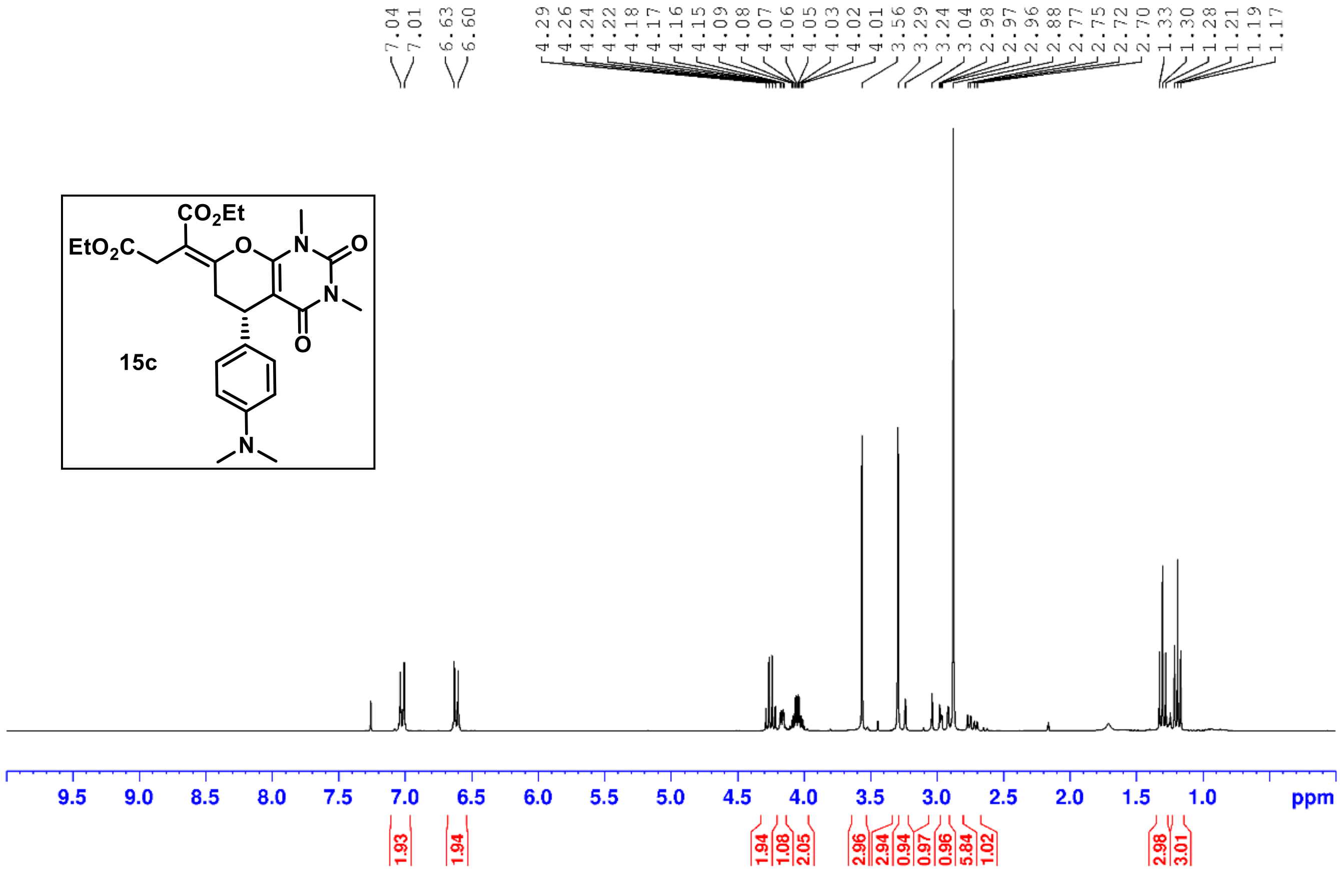
7.04
7.01
6.63
6.60
4.29
4.26
4.24
4.22
4.18
4.17
4.16
4.15
4.09
4.08
4.07
4.06
4.05
4.03
4.02
4.01
3.56
3.29
3.24
3.04
2.98
2.97
2.96
2.88
2.77
2.75
2.72
2.70
1.33
1.30
1.28
1.21
1.19
1.17
CO_2Et
EtO_2C
O
N
O
N
O
N
15c
9.5
9.0
8.5
8.0
7.5
7.0
6.5
6.0
5.5
5.0
4.5
4.0
3.5
3.0
2.5
2.0
1.5
1.0
ppm
1.93
1.94
1.94
1.08
2.05
2.96
2.94
0.94
0.97
0.96
5.84
1.02
2.98
3.01

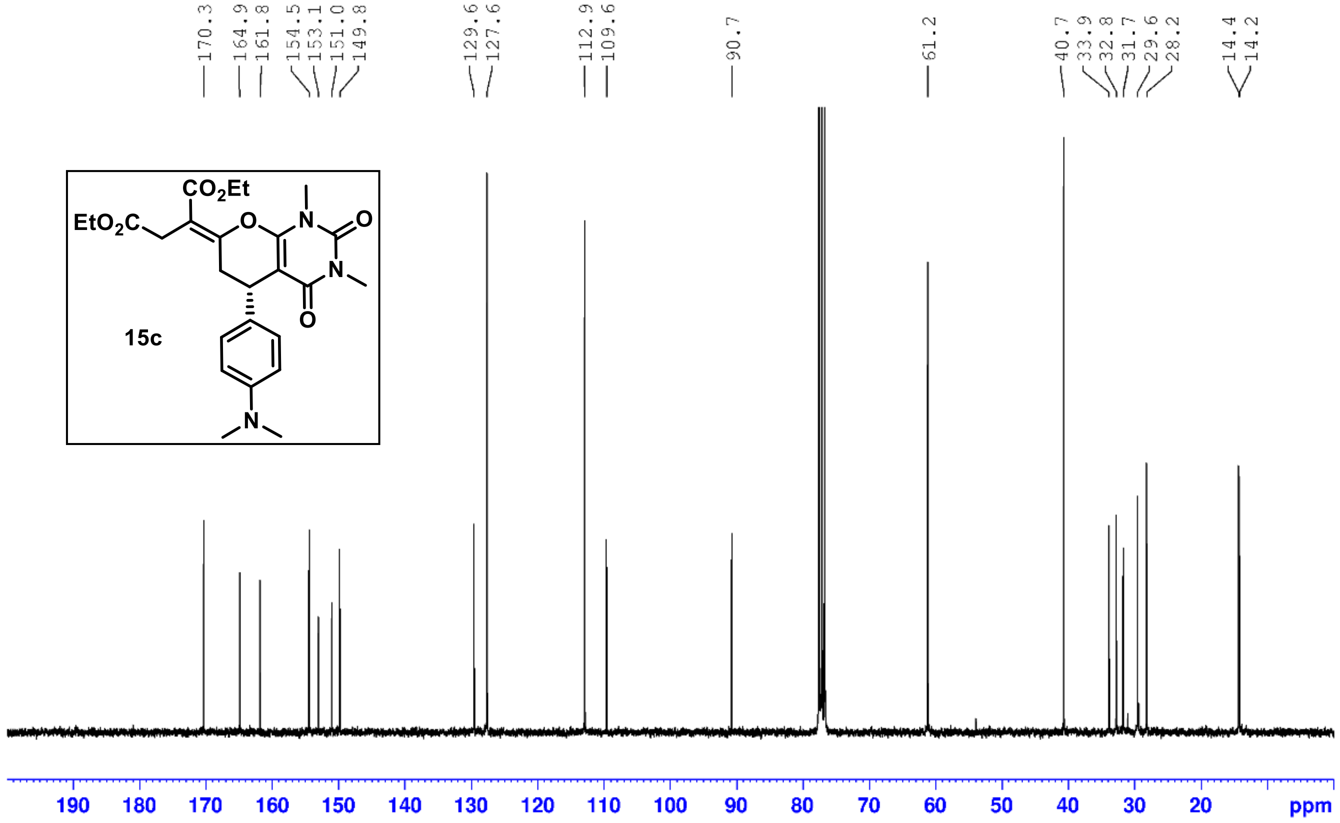
170.3
164.9
161.8
154.5
153.1
151.0
149.8
129.6
127.6
112.9
109.6
90.7
61.2
40.7
33.9
32.8
31.7
29.6
28.2
14.4
14.2
CO_2Et
EtO_2C
O
N
O
N
O
N
15c
190
180
170
160
150
140
130
120
110
100
90
80
70
60
50
40
30
20
ppm

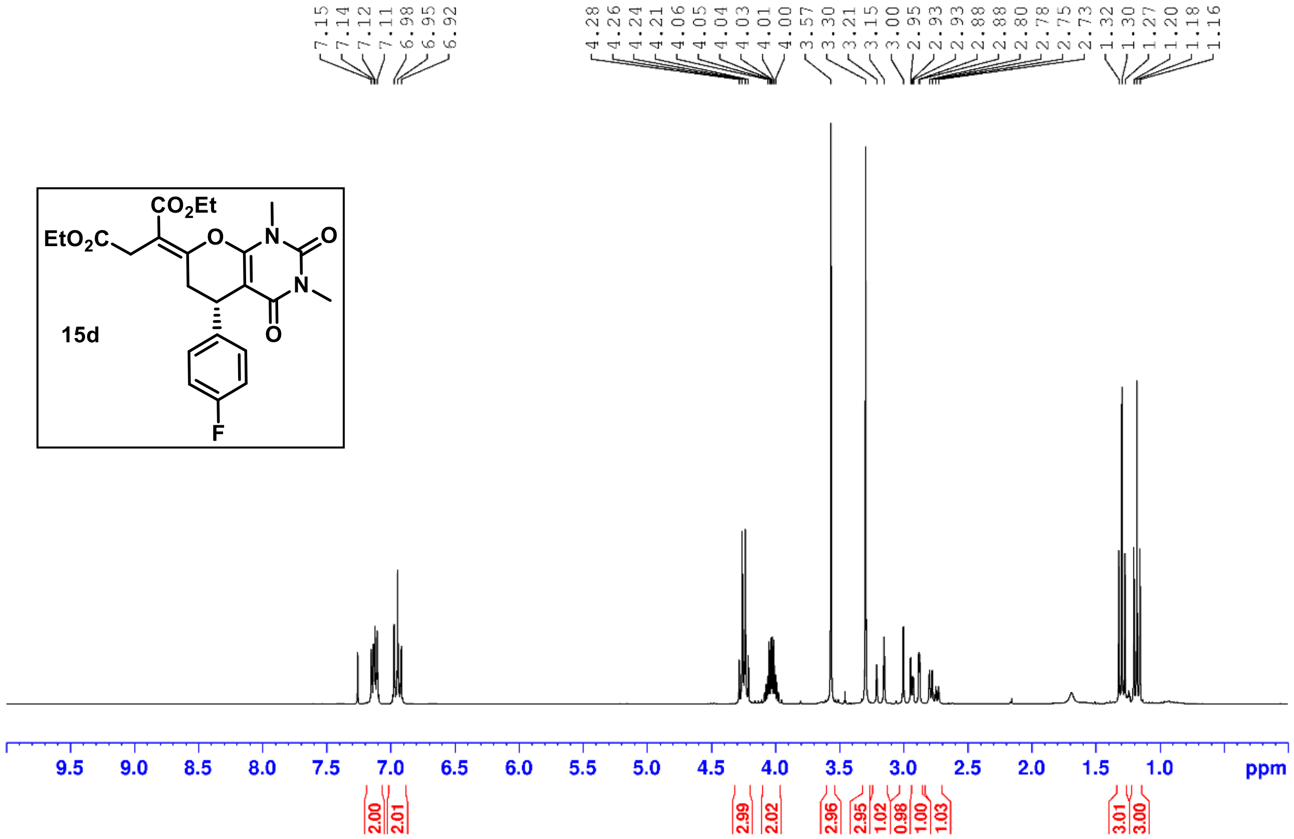
7.15 7.14 7.12 7.11 6.98 6.95 6.92
4.28 4.26 4.24 4.21 4.06 4.05 4.04 4.03 4.01 4.00 3.57 3.30 3.21 3.15 3.00 2.95 2.93 2.93 2.88 2.88 2.80 2.78 2.75 2.73 1.32 1.30 1.27 1.20 1.18 1.16
CO2Et
EtO2C
O
N
O
N
O
F
15d
9.5 9.0 8.5 8.0 7.5 7.0 6.5 6.0 5.5 5.0 4.5 4.0 3.5 3.0 2.5 2.0 1.5 1.0 ppm
2.00 2.01 2.99 2.02 2.96 2.95 1.02 0.98 1.00 1.03 3.01 3.00

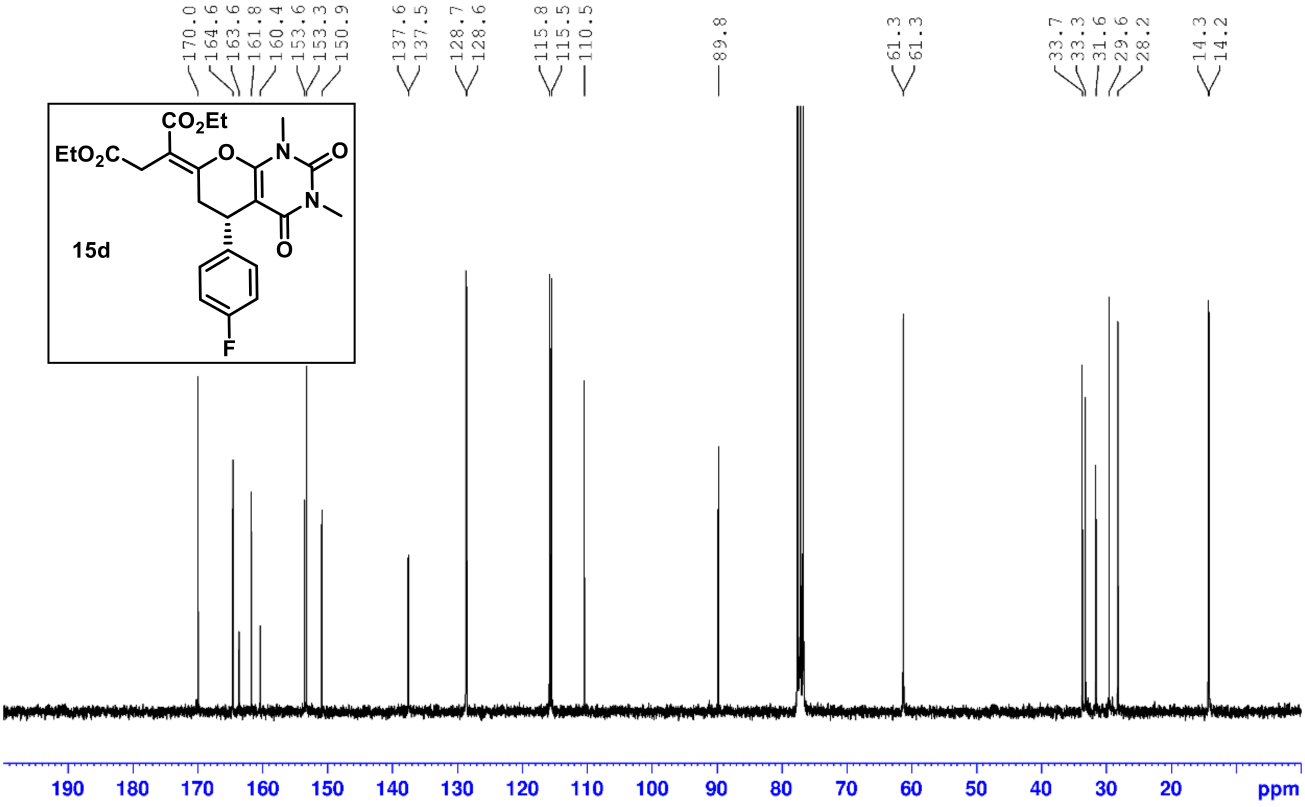
170.0 164.6 163.6 161.8 160.4 153.6 153.3 150.9 137.6 137.5 128.7 128.6 115.8 115.5 110.5 89.8 61.3 61.3 33.7 33.3 31.6 29.6 28.2 14.3 14.2
CO2Et
EtO2C
O
N
O
N
O
F
15d
190 180 170 160 150 140 130 120 110 100 90 80 70 60 50 40 30 20 ppm

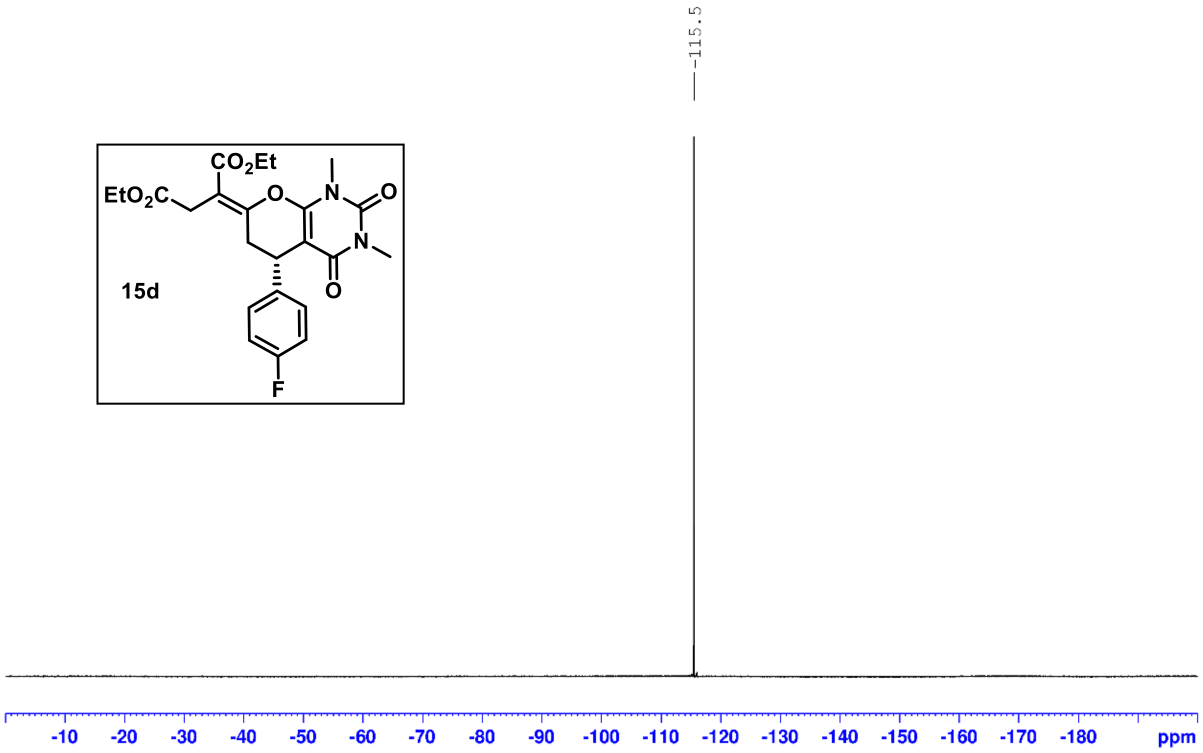
-115.5
CO2Et
EtO2C
O
N
O
N
O
15d
F
-10 -20 -30 -40 -50 -60 -70 -80 -90 -100 -110 -120 -130 -140 -150 -160 -170 -180 ppm

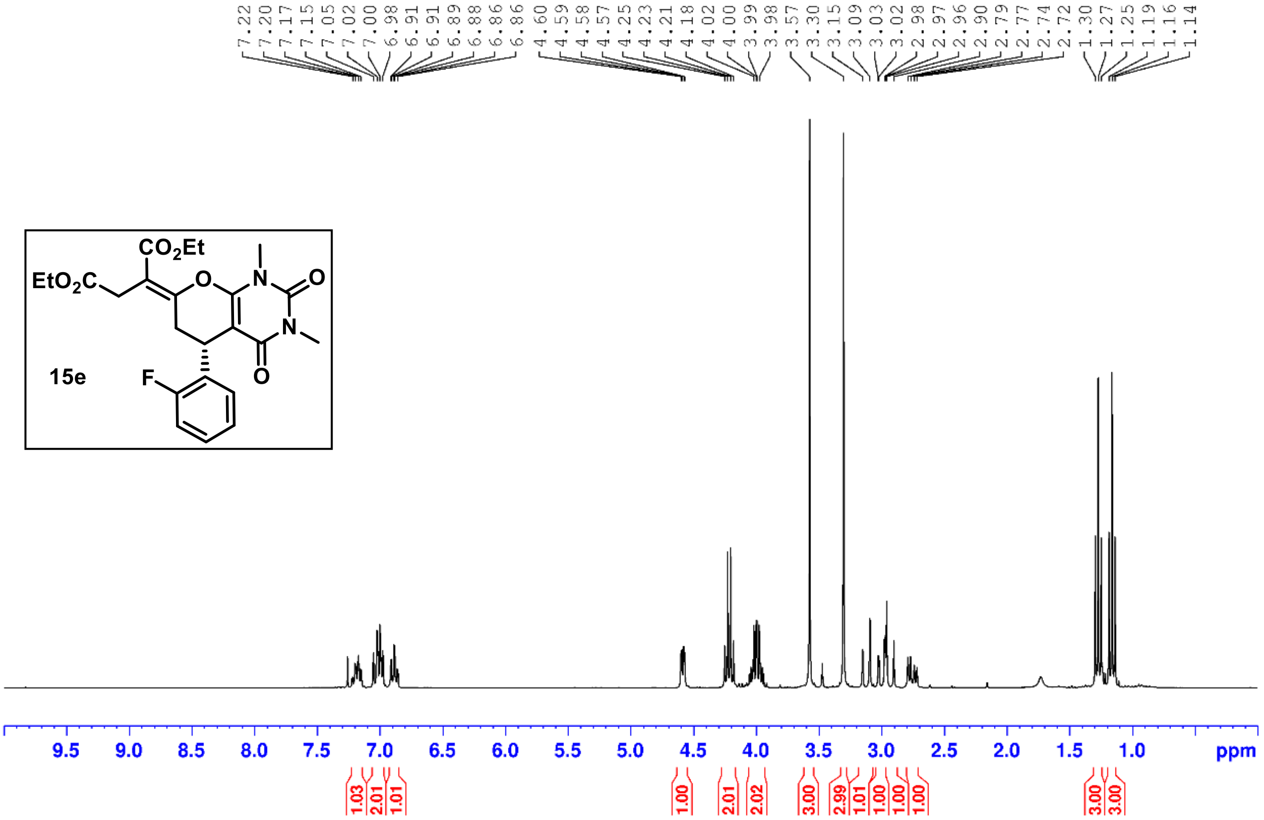
7.22 7.20 7.17 7.15 7.05 7.02 7.00 6.98 6.91 6.91 6.89 6.88 6.86 4.60 4.59 4.58 4.57 4.25 4.23 4.21 4.18 4.02 4.00 3.99 3.98 3.57 3.30 3.15 3.09 3.03 3.02 2.98 2.97 2.96 2.90 2.79 2.77 2.74 2.72 1.30 1.27 1.25 1.19 1.16 1.14
CO2Et
EtO2C
O
N
O
N
O
15e
F
9.5 9.0 8.5 8.0 7.5 7.0 6.5 6.0 5.5 5.0 4.5 4.0 3.5 3.0 2.5 2.0 1.5 1.0 ppm
1.03 2.01 1.01 1.00 2.01 2.02 3.00 2.99 1.01 1.00 1.00 1.00 3.00 3.00

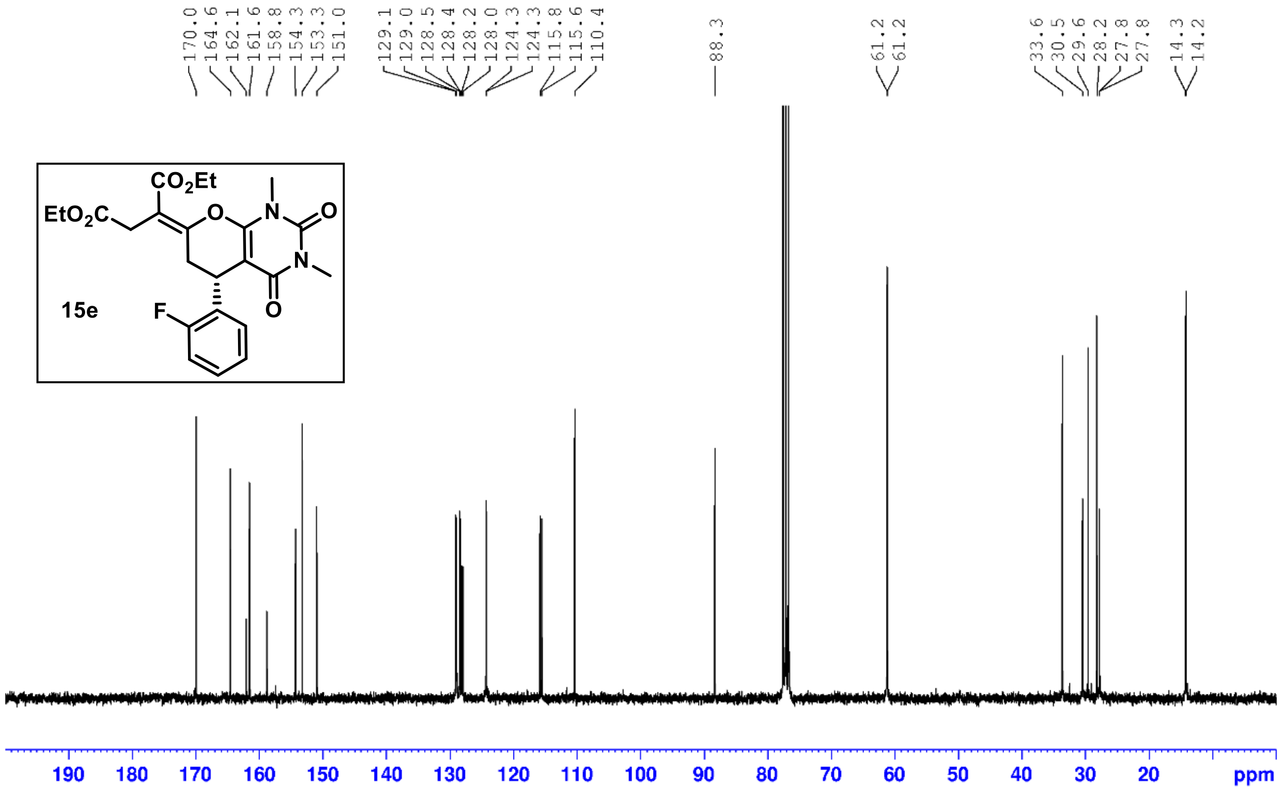
170.0
164.6
162.1
161.6
158.8
154.3
153.3
151.0
129.1
129.0
128.5
128.4
128.2
128.0
124.3
124.3
115.8
115.6
110.4
88.3
61.2
61.2
33.6
30.5
29.6
28.2
27.8
27.8
14.3
14.2
190
180
170
160
150
140
130
120
110
100
90
80
70
60
50
40
30
20
ppm
15e

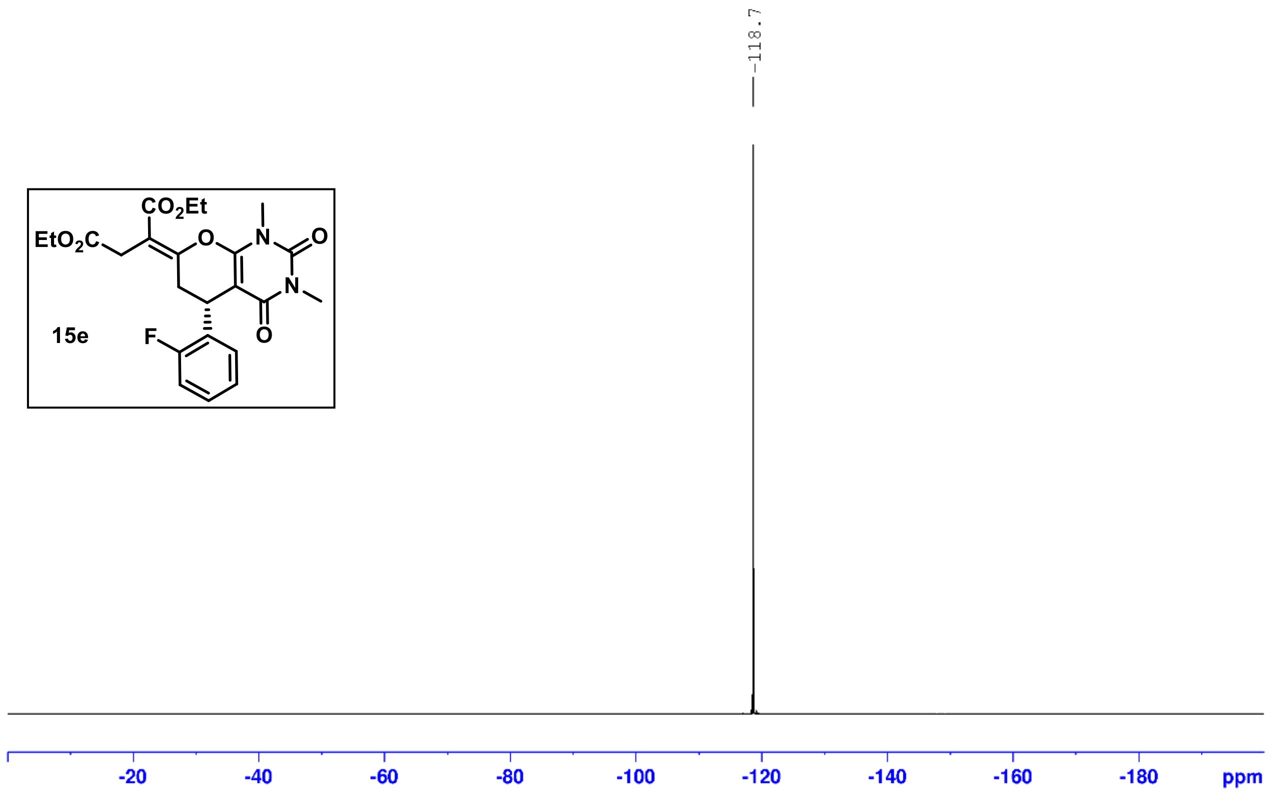
-118.7
-20
-40
-60
-80
-100
-120
-140
-160
-180
ppm
15e

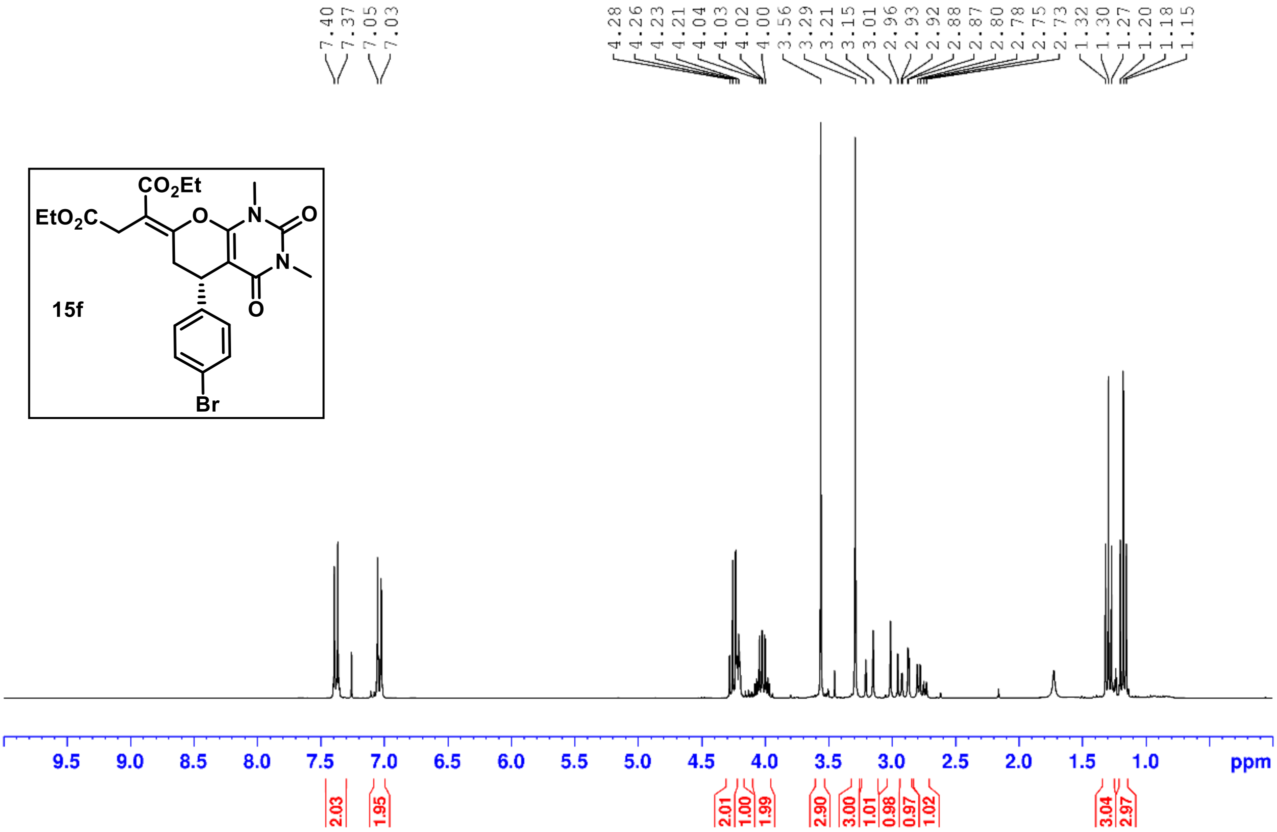
7.40
7.37
7.05
7.03
4.28
4.26
4.23
4.21
4.04
4.03
4.02
4.00
3.56
3.29
3.21
3.15
3.01
2.96
2.93
2.92
2.88
2.87
2.80
2.78
2.75
2.73
1.32
1.30
1.27
1.20
1.18
1.15
CO2Et
EtO2C
O
N
O
N
O
15f
Br
9.5 9.0 8.5 8.0 7.5 7.0 6.5 6.0 5.5 5.0 4.5 4.0 3.5 3.0 2.5 2.0 1.5 1.0 ppm
2.03
1.95
2.01
1.00
1.99
2.90
3.00
1.01
0.98
0.97
1.02
3.04
2.97

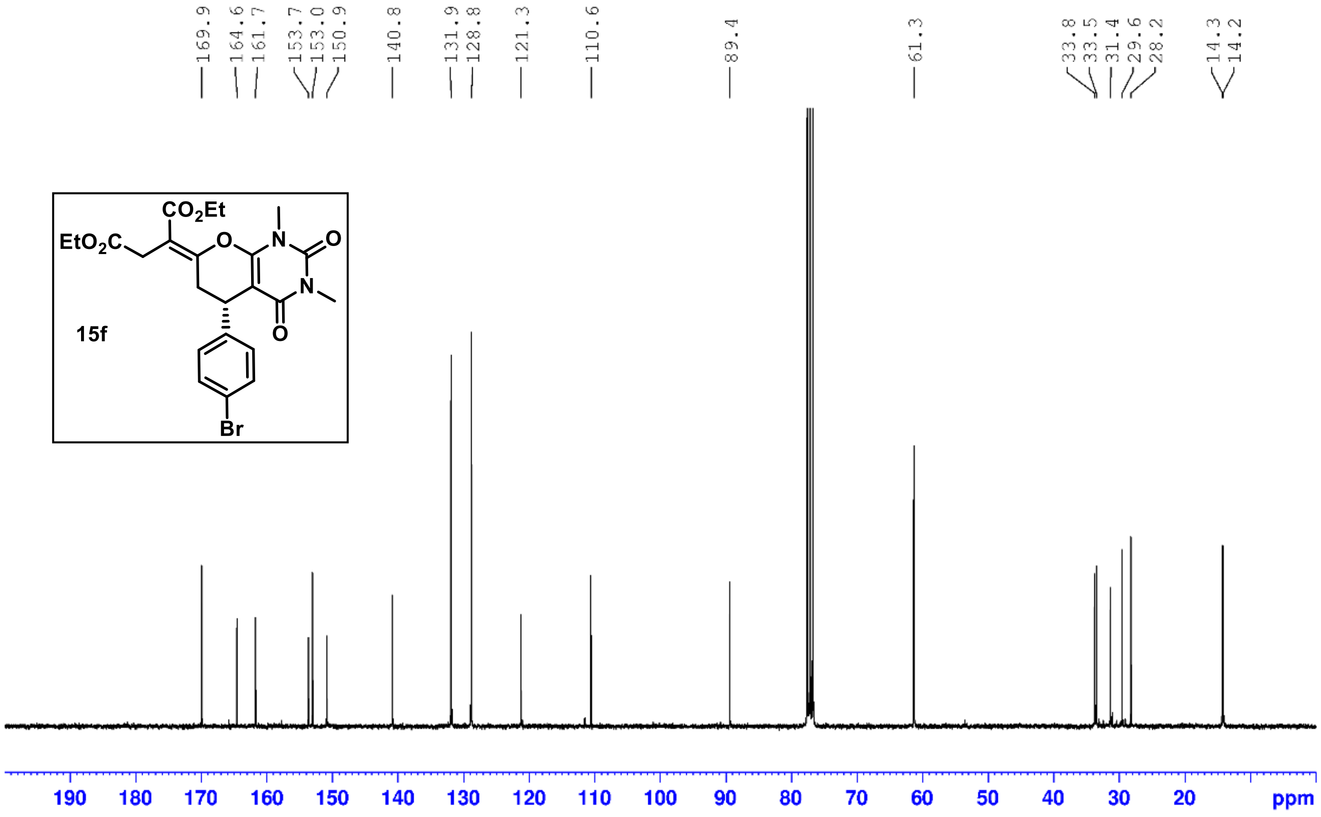
169.9
164.6
161.7
153.7
153.0
150.9
140.8
131.9
128.8
121.3
110.6
89.4
61.3
33.8
33.5
31.4
29.6
28.2
14.3
14.2
CO2Et
EtO2C
O
N
O
N
O
15f
Br
190 180 170 160 150 140 130 120 110 100 90 80 70 60 50 40 30 20 ppm

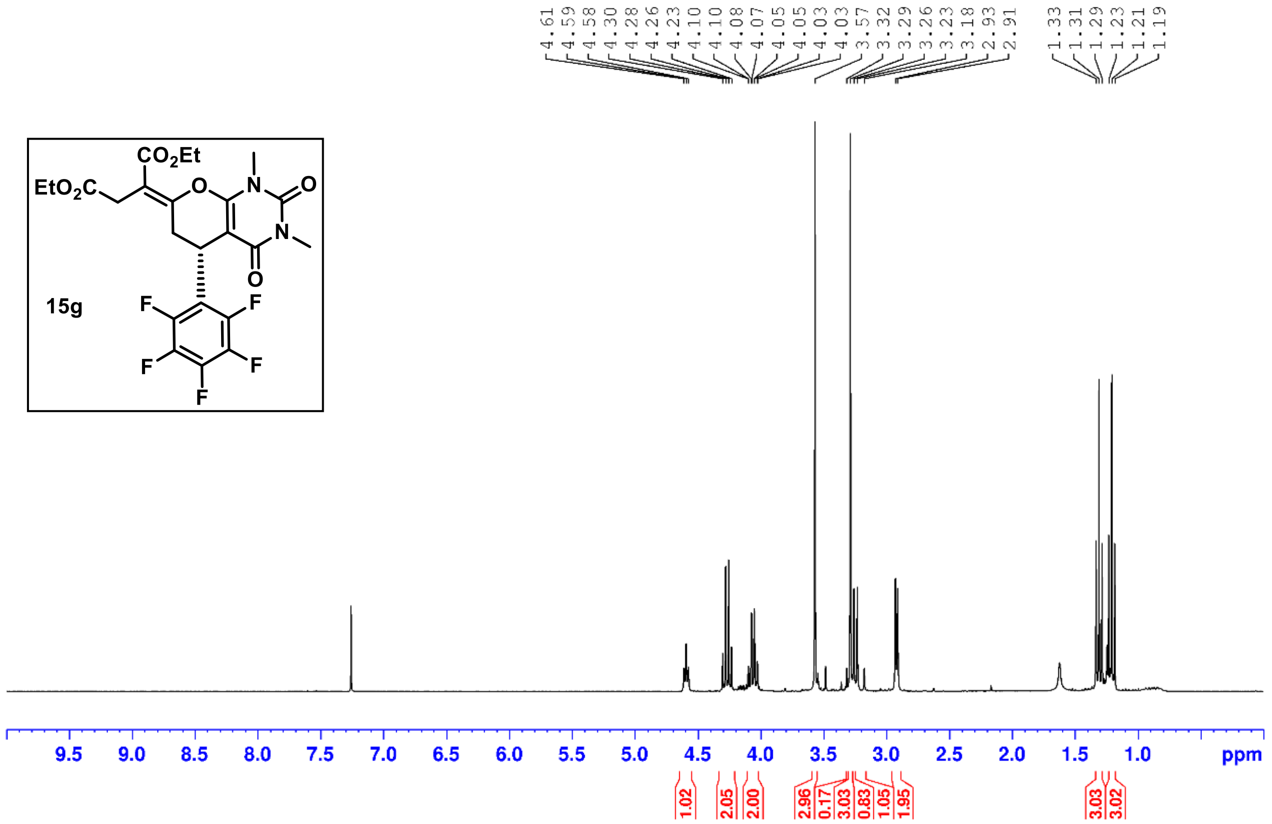
4.61 4.59 4.58 4.30 4.28 4.26 4.23 4.10 4.10 4.08 4.07 4.05 4.05 4.03 4.03 3.57 3.32 3.29 3.26 3.23 3.18 2.93 2.91
1.33 1.31 1.29 1.23 1.21 1.19
15g
9.5 9.0 8.5 8.0 7.5 7.0 6.5 6.0 5.5 5.0 4.5 4.0 3.5 3.0 2.5 2.0 1.5 1.0 ppm
1.02 2.05 2.00 2.96 0.17 3.03 0.83 1.05 1.95 3.03 3.02

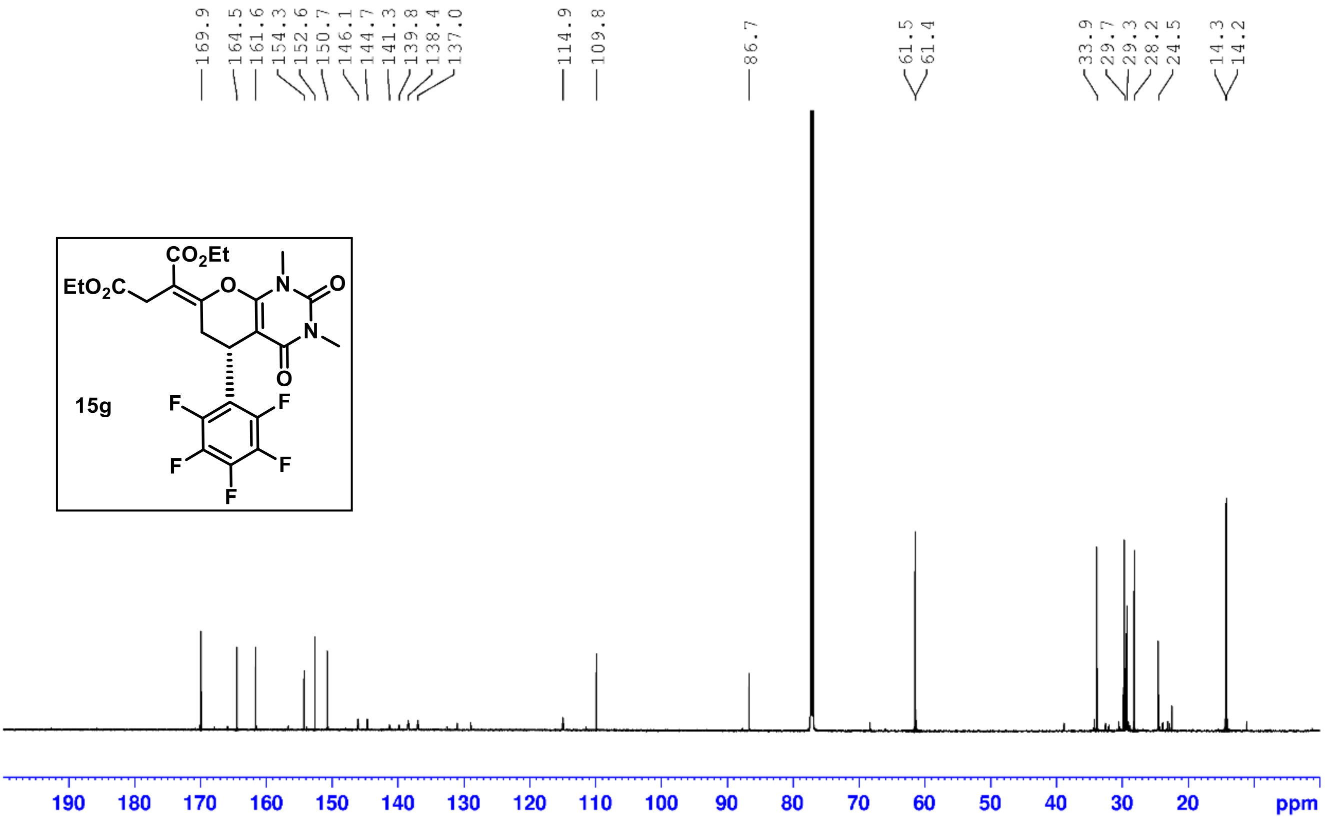
169.9 164.5 161.6 154.3 152.6 150.7 146.1 144.7 141.3 139.8 138.4 137.0
114.9 109.8
86.7
61.5 61.4
33.9 29.7 29.3 28.2 24.5
14.3 14.2
15g
190 180 170 160 150 140 130 120 110 100 90 80 70 60 50 40 30 20 ppm

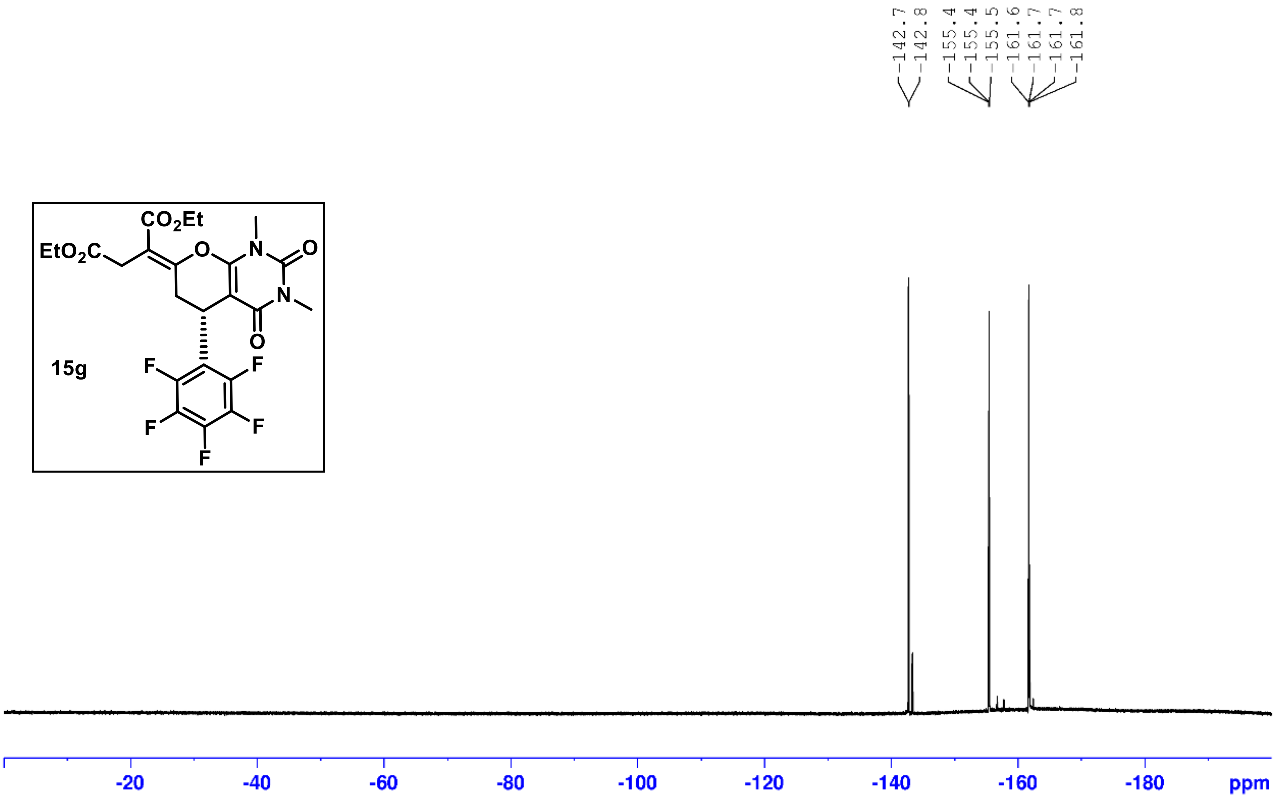

15g
CO2Et
EtO2C
O
N
N
O
O
F
F
F
F
F
ppm

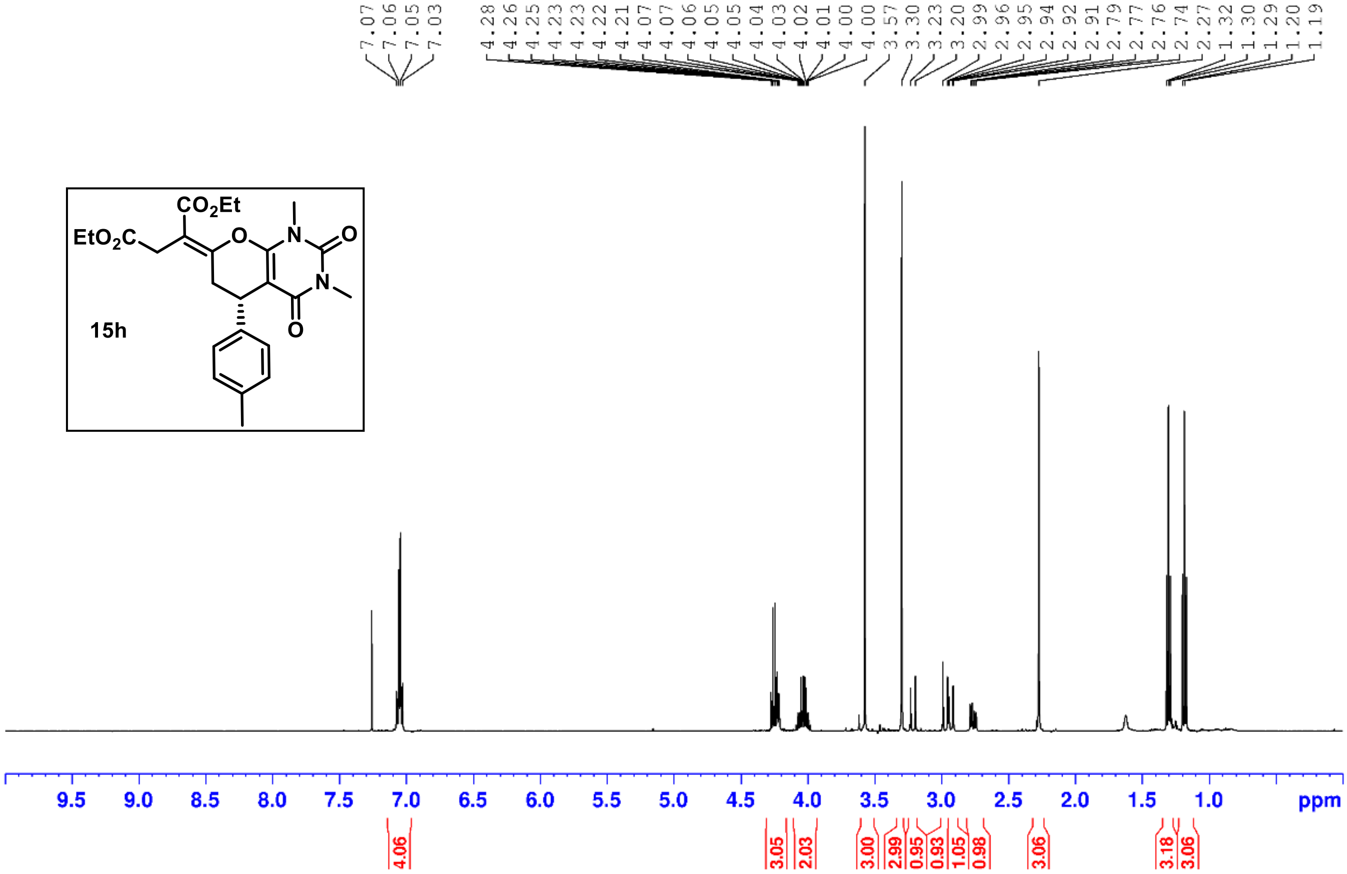

15h
CO2Et
EtO2C
O
N
N
O
O
ppm

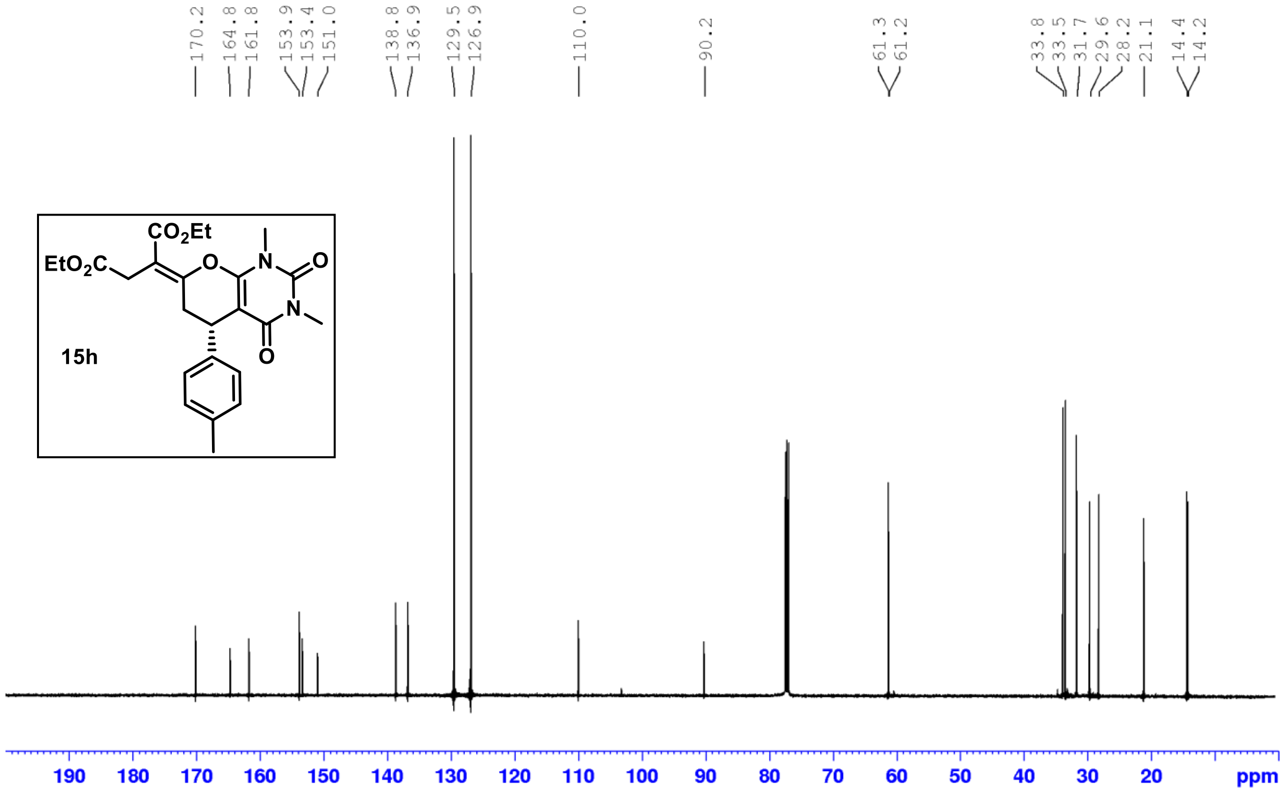
170.2
164.8
161.8
153.9
153.4
151.0
138.8
136.9
129.5
126.9
110.0
90.2
61.3
61.2
33.8
33.5
31.7
29.6
28.2
21.1
14.4
14.2
CO2Et
EtO2C
O
N
N
O
O
15h
190 180 170 160 150 140 130 120 110 100 90 80 70 60 50 40 30 20 ppm

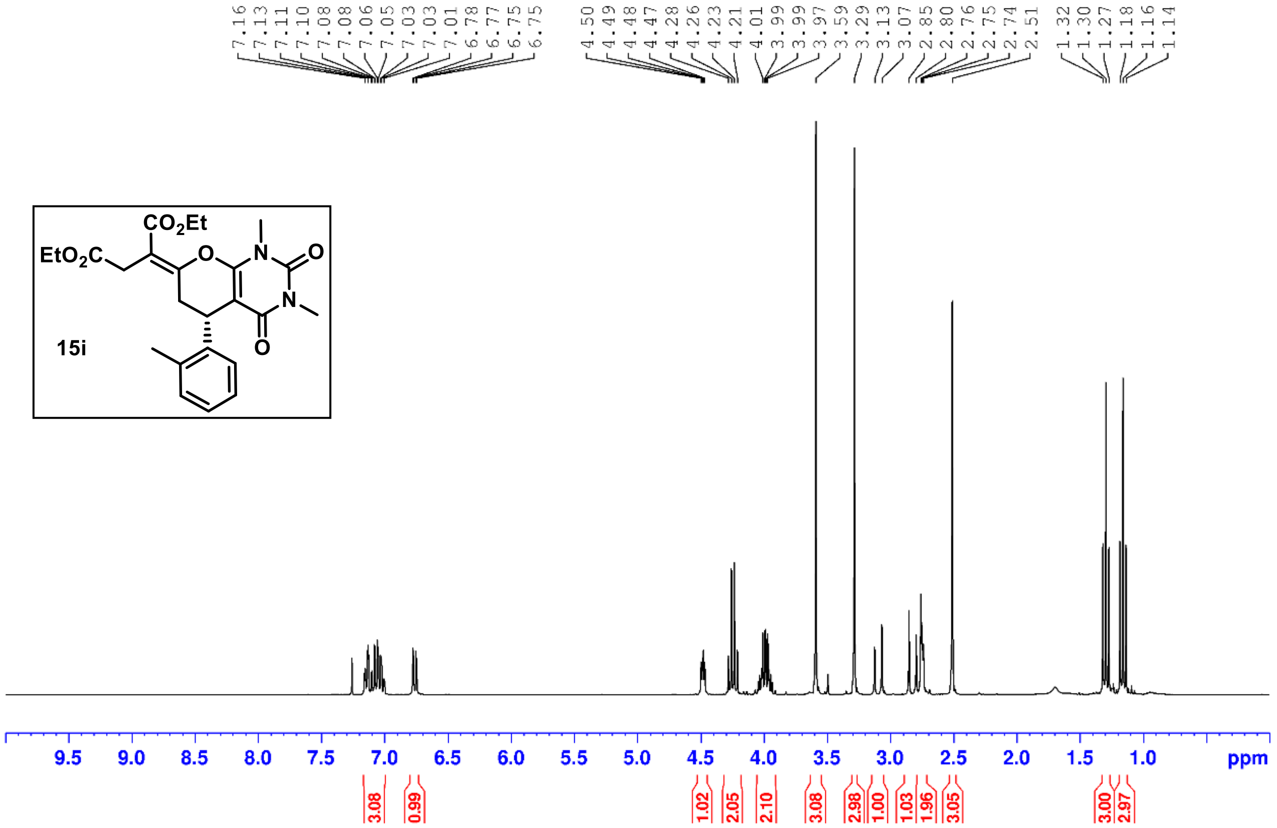
7.16
7.13
7.11
7.10
7.08
7.08
7.06
7.05
7.03
7.03
7.01
6.78
6.77
6.75
6.75
4.50
4.49
4.48
4.47
4.28
4.26
4.23
4.21
4.01
3.99
3.99
3.97
3.59
3.29
3.13
3.07
2.85
2.80
2.76
2.75
2.74
2.51
1.32
1.30
1.27
1.18
1.16
1.14
CO2Et
EtO2C
O
N
N
O
O
15i
9.5 9.0 8.5 8.0 7.5 7.0 6.5 6.0 5.5 5.0 4.5 4.0 3.5 3.0 2.5 2.0 1.5 1.0 ppm
3.08
0.99
1.02
2.05
2.10
3.08
2.98
1.00
1.03
1.96
3.05
3.00
2.97

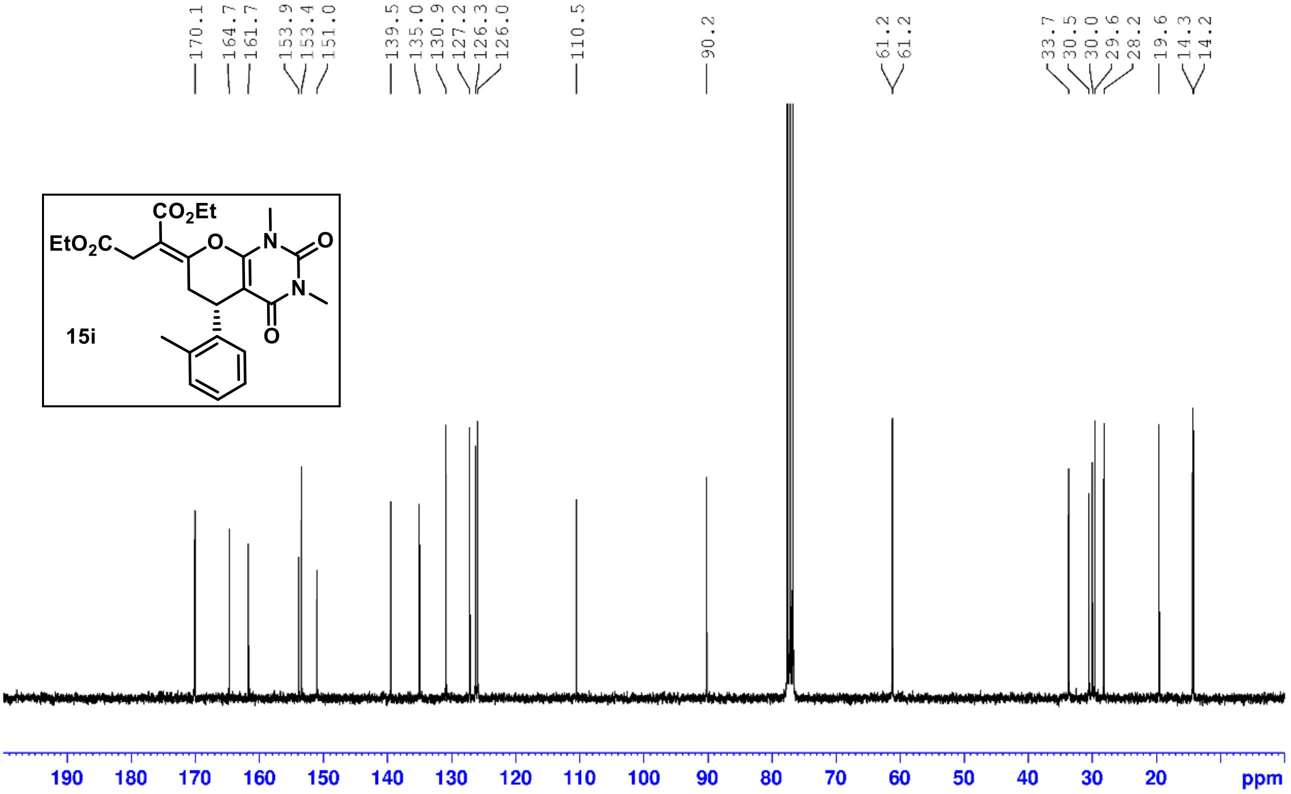
170.1
164.7
161.7
153.9
153.4
151.0
139.5
135.0
130.9
127.2
126.3
126.0
110.5
90.2
61.2
61.2
33.7
30.5
30.0
29.6
28.2
19.6
14.3
14.2
CO_2Et
EtO_2C
O
N
O
N
O
15i
190
180
170
160
150
140
130
120
110
100
90
80
70
60
50
40
30
20
ppm

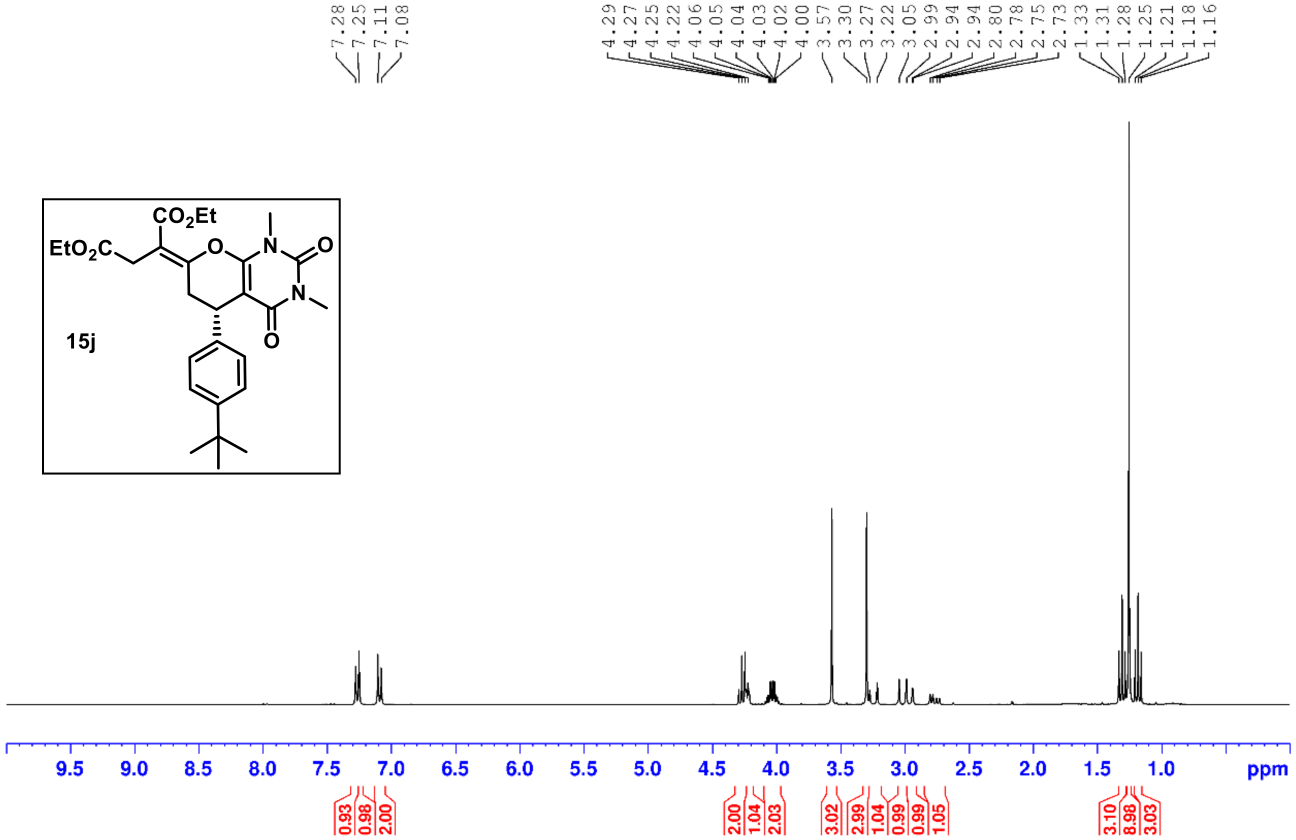
7.28
7.25
7.11
7.08
4.29
4.27
4.25
4.22
4.06
4.05
4.04
4.03
4.02
4.00
3.57
3.30
3.27
3.22
3.05
2.99
2.94
2.94
2.80
2.78
2.75
2.73
1.33
1.31
1.28
1.25
1.21
1.18
1.16
CO_2Et
EtO_2C
O
N
O
N
O
15j
9.5
9.0
8.5
8.0
7.5
7.0
6.5
6.0
5.5
5.0
4.5
4.0
3.5
3.0
2.5
2.0
1.5
1.0
ppm
0.93
0.98
2.00
2.00
1.04
2.03
3.02
2.99
1.04
0.99
0.99
1.05
3.10
8.98
3.03

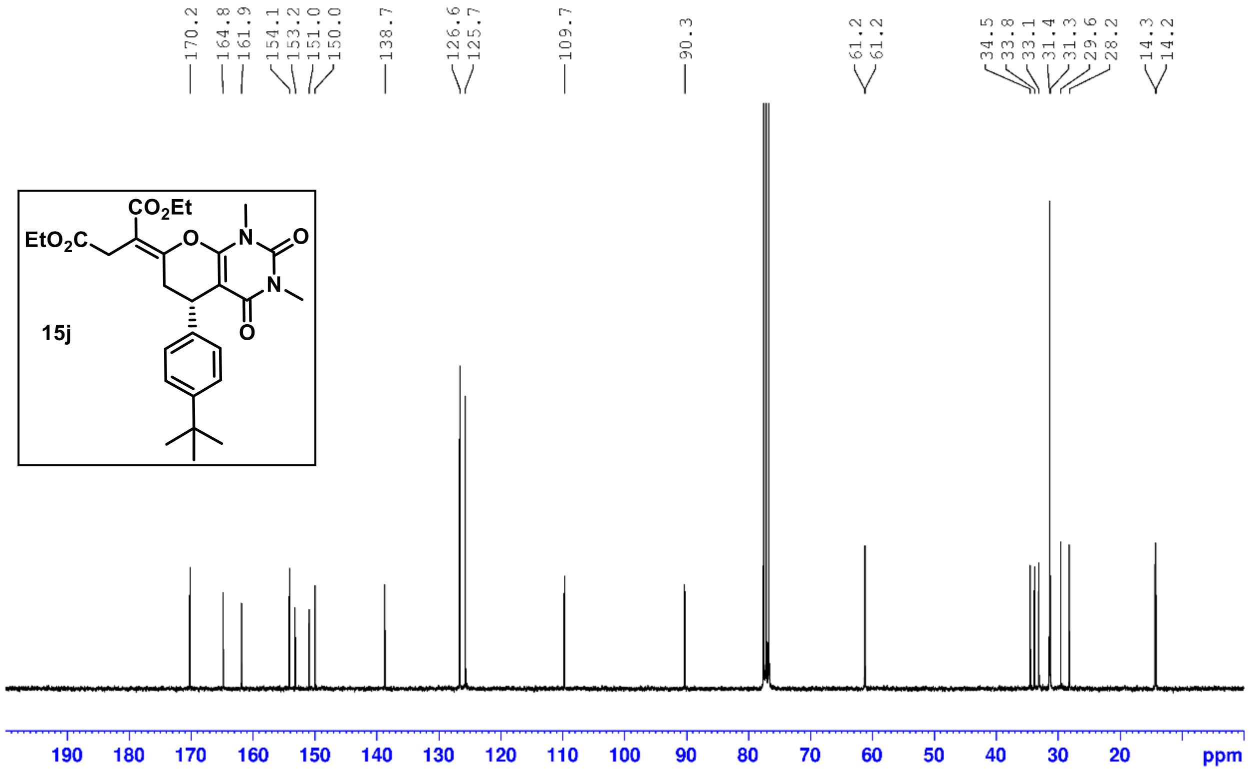
170.2
164.8
161.9
154.1
153.2
151.0
150.0
138.7
126.6
125.7
109.7
90.3
61.2
61.2
34.5
33.8
33.1
31.4
31.3
29.6
28.2
14.3
14.2
CO2Et
EtO2C
O
N
O
N
O
15j
190 180 170 160 150 140 130 120 110 100 90 80 70 60 50 40 30 20 ppm

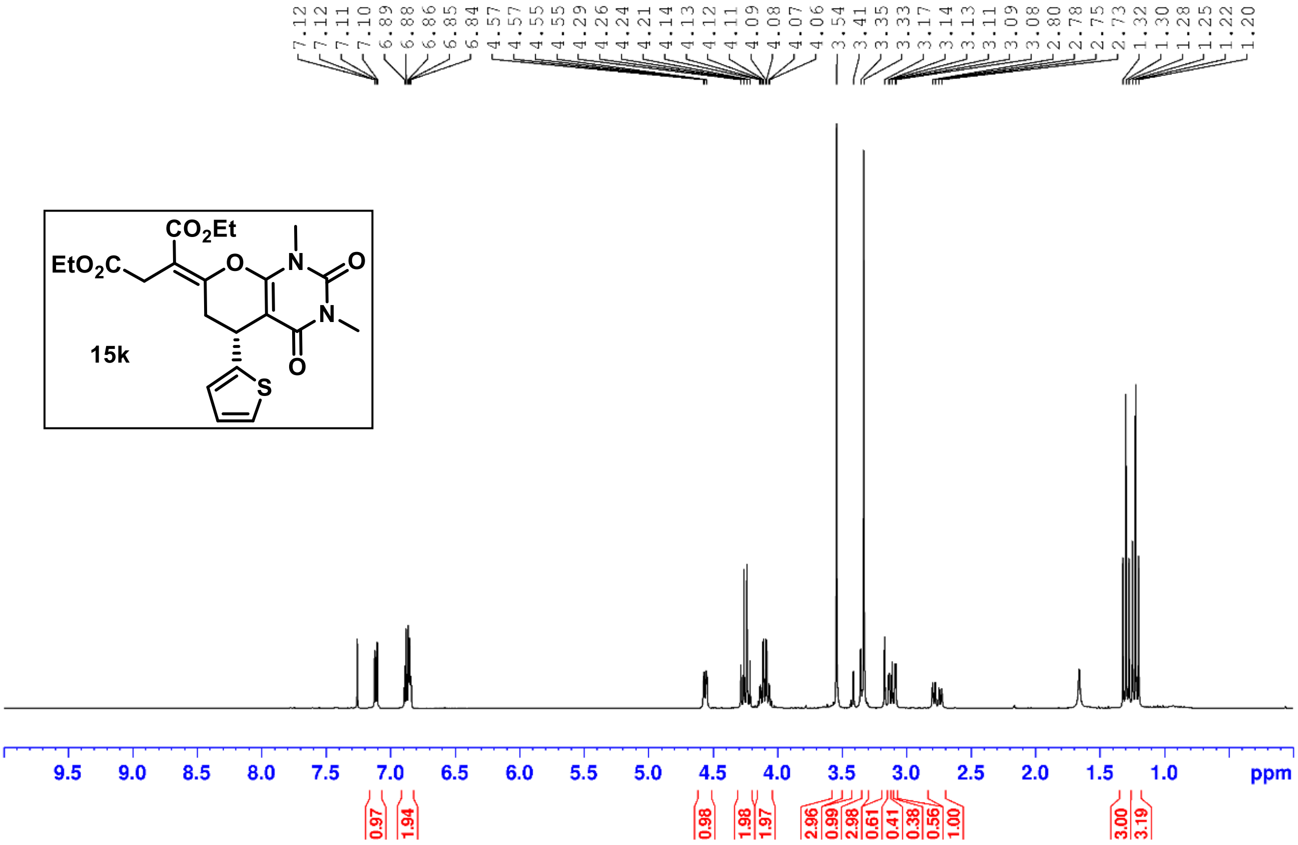
7.12
7.12
7.11
7.10
6.89
6.88
6.86
6.85
6.84
4.57
4.57
4.55
4.55
4.29
4.26
4.24
4.21
4.14
4.13
4.12
4.11
4.09
4.08
4.07
4.06
3.54
3.41
3.35
3.33
3.17
3.14
3.13
3.11
3.09
3.08
2.80
2.78
2.75
2.73
1.32
1.30
1.28
1.25
1.22
1.20
CO2Et
EtO2C
O
N
O
N
O
S
15k
9.5 9.0 8.5 8.0 7.5 7.0 6.5 6.0 5.5 5.0 4.5 4.0 3.5 3.0 2.5 2.0 1.5 1.0 ppm
0.97
1.94
0.98
1.98
1.97
2.96
0.99
2.98
0.61
0.41
0.38
0.56
1.00
3.00
3.19

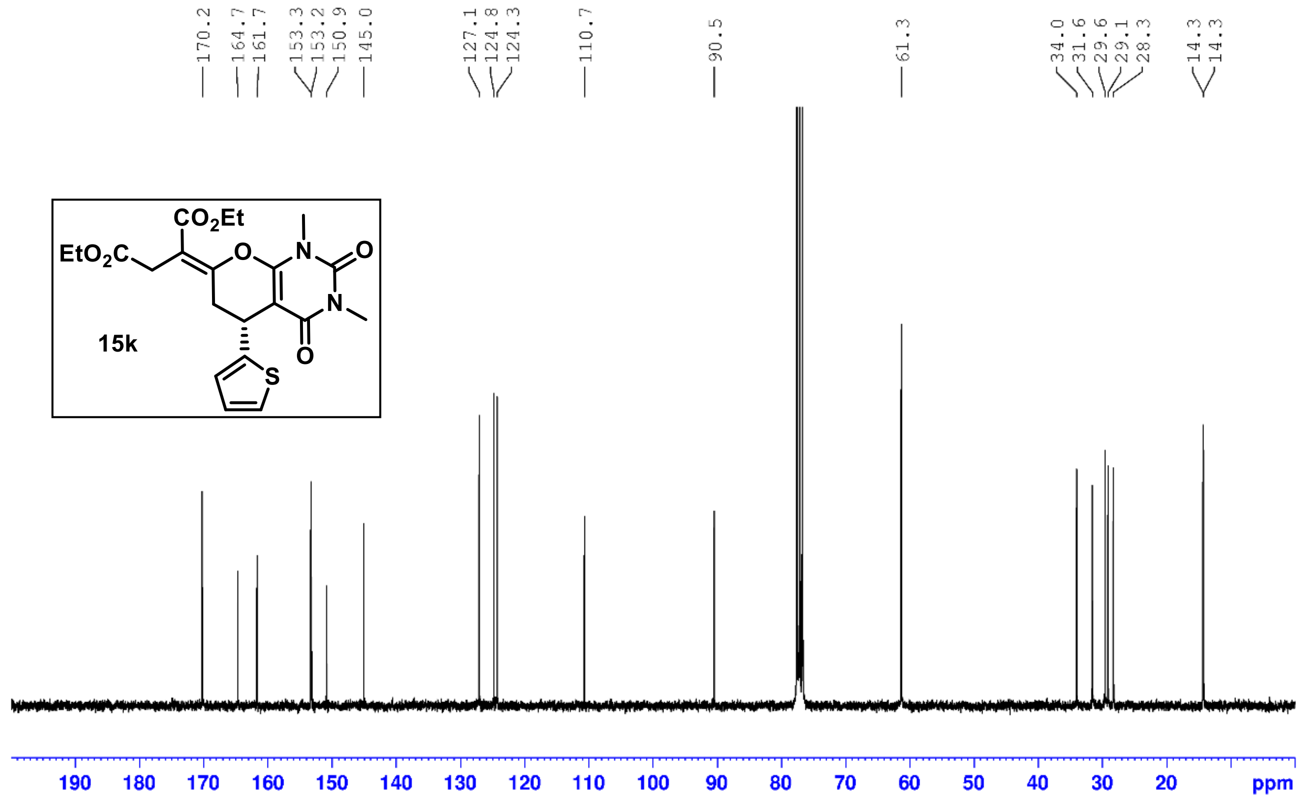

170.2
164.7
161.7
153.3
153.2
150.9
145.0
127.1
124.8
124.3
110.7
90.5
61.3
34.0
31.6
29.6
29.1
28.3
14.3
14.3
CO2Et
EtO2C
O
N
O
N
O
S
15k
190 180 170 160 150 140 130 120 110 100 90 80 70 60 50 40 30 20 ppm

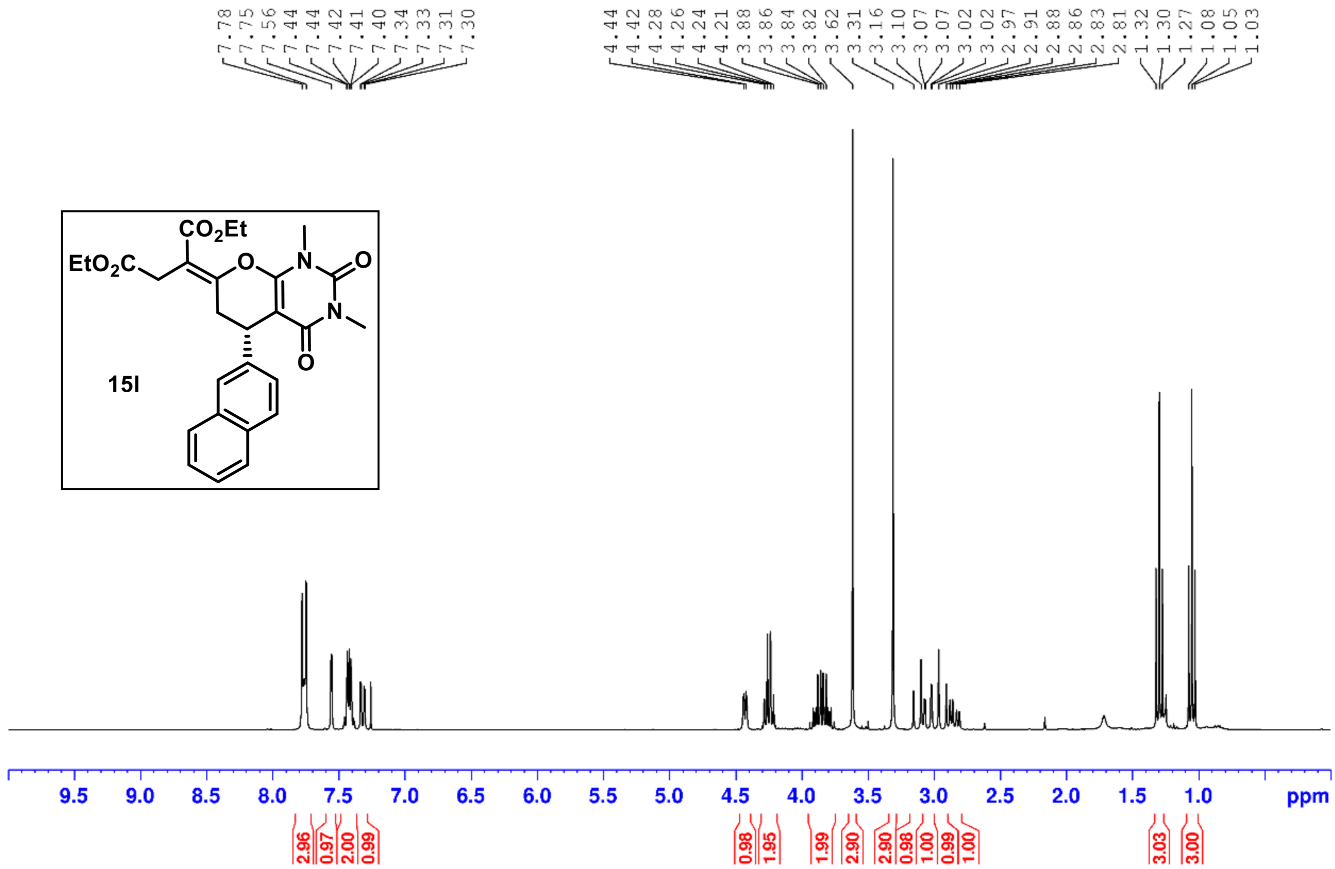

7.78
7.75
7.56
7.44
7.44
7.42
7.41
7.40
7.34
7.33
7.31
7.30
4.44
4.42
4.28
4.26
4.24
4.21
3.88
3.86
3.84
3.82
3.62
3.31
3.16
3.10
3.07
3.07
3.02
2.97
2.91
2.88
2.86
2.83
2.81
1.32
1.30
1.27
1.08
1.05
1.03
CO2Et
EtO2C
O
N
O
N
O
15l
9.5 9.0 8.5 8.0 7.5 7.0 6.5 6.0 5.5 5.0 4.5 4.0 3.5 3.0 2.5 2.0 1.5 1.0 ppm
2.96
0.97
2.00
0.99
0.98
1.95
1.99
2.90
2.90
0.98
1.00
0.99
1.00
3.03
3.00

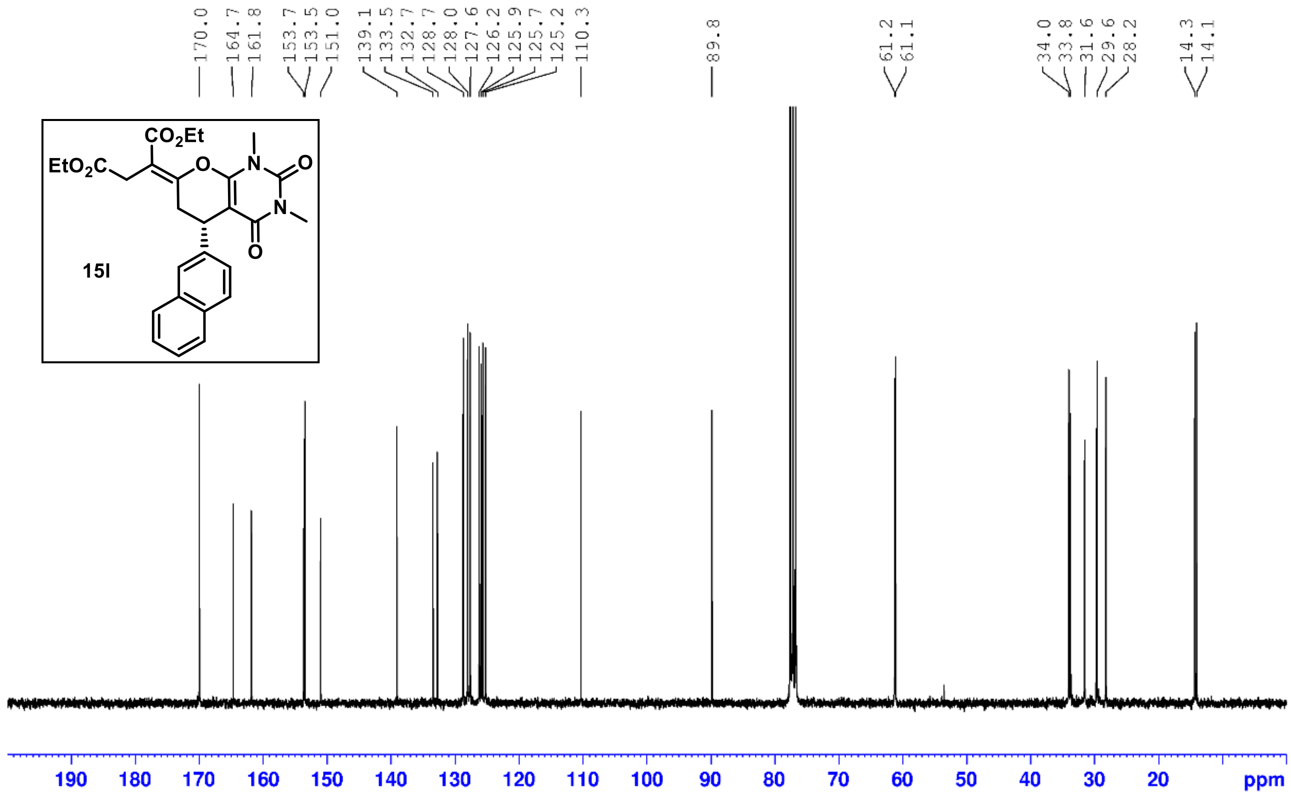
170.0
164.7
161.8
153.7
153.5
151.0
139.1
133.5
132.7
128.7
128.0
127.6
126.2
125.9
125.7
125.2
110.3
89.8
61.2
61.1
34.0
33.8
31.6
29.6
28.2
14.3
14.1
15l
190 180 170 160 150 140 130 120 110 100 90 80 70 60 50 40 30 20 ppm

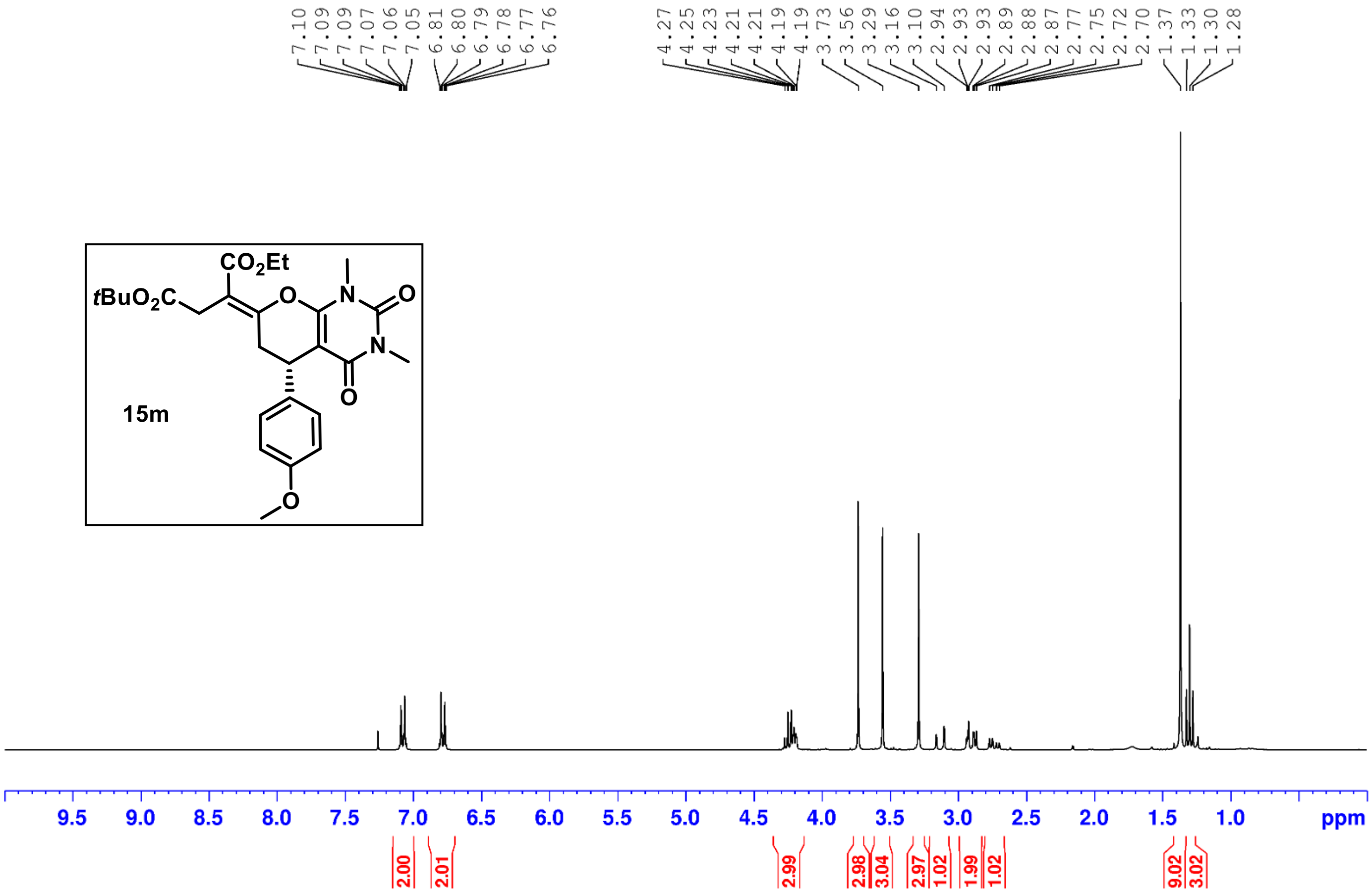
7.10
7.09
7.09
7.07
7.06
7.05
6.81
6.80
6.79
6.78
6.77
6.76
4.27
4.25
4.23
4.21
4.21
4.19
4.19
3.73
3.56
3.29
3.16
3.10
2.94
2.93
2.93
2.89
2.88
2.87
2.77
2.75
2.72
2.70
1.37
1.33
1.30
1.28
15m
9.5 9.0 8.5 8.0 7.5 7.0 6.5 6.0 5.5 5.0 4.5 4.0 3.5 3.0 2.5 2.0 1.5 1.0 ppm
2.00
2.01
2.99
2.98
3.04
2.97
1.02
1.99
1.02
9.02
3.02

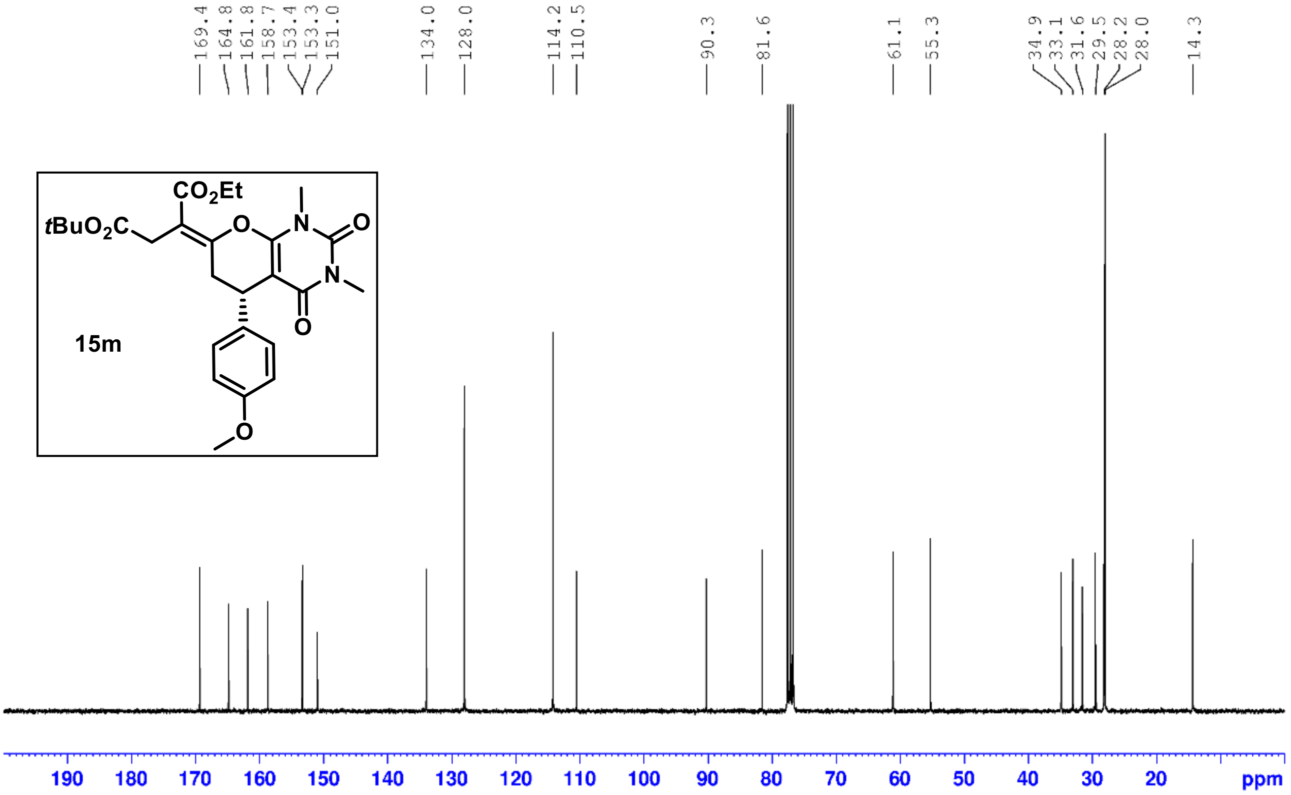
169.4
164.8
161.8
158.7
153.4
153.3
151.0
134.0
128.0
114.2
110.5
90.3
81.6
61.1
55.3
34.9
33.1
31.6
29.5
28.2
28.0
14.3
CO2Et
tBuO2C
O
N
O
N
O
O
15m
190 180 170 160 150 140 130 120 110 100 90 80 70 60 50 40 30 20 ppm

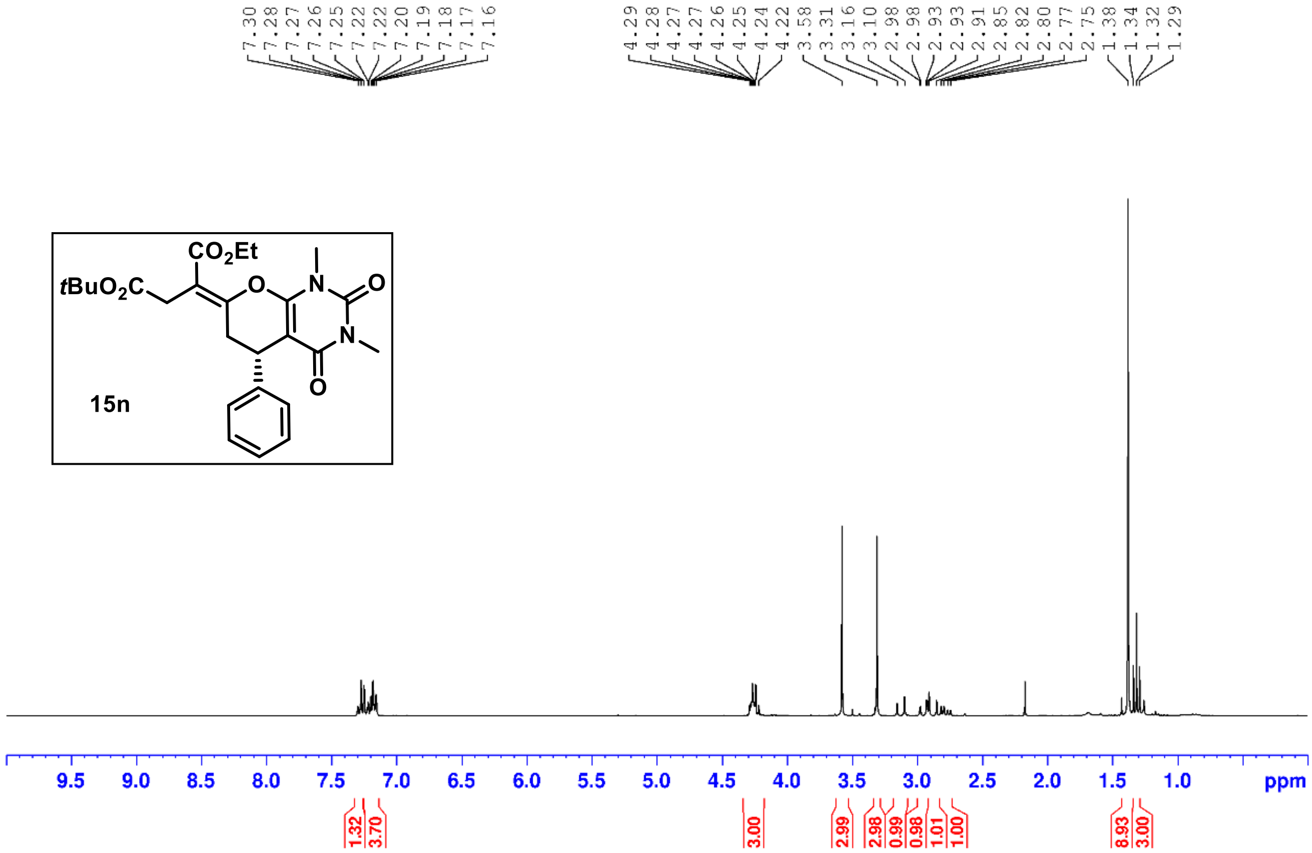
7.30
7.28
7.27
7.26
7.25
7.22
7.22
7.20
7.19
7.18
7.17
7.16
4.29
4.28
4.27
4.27
4.26
4.25
4.24
4.22
3.58
3.31
3.16
3.10
2.98
2.98
2.93
2.93
2.91
2.85
2.82
2.80
2.77
2.75
1.38
1.34
1.32
1.29
CO2Et
tBuO2C
O
N
O
N
O
15n
9.5 9.0 8.5 8.0 7.5 7.0 6.5 6.0 5.5 5.0 4.5 4.0 3.5 3.0 2.5 2.0 1.5 1.0 ppm
1.32
3.70
3.00
2.99
2.98
0.99
0.98
1.01
1.00
8.93
3.00

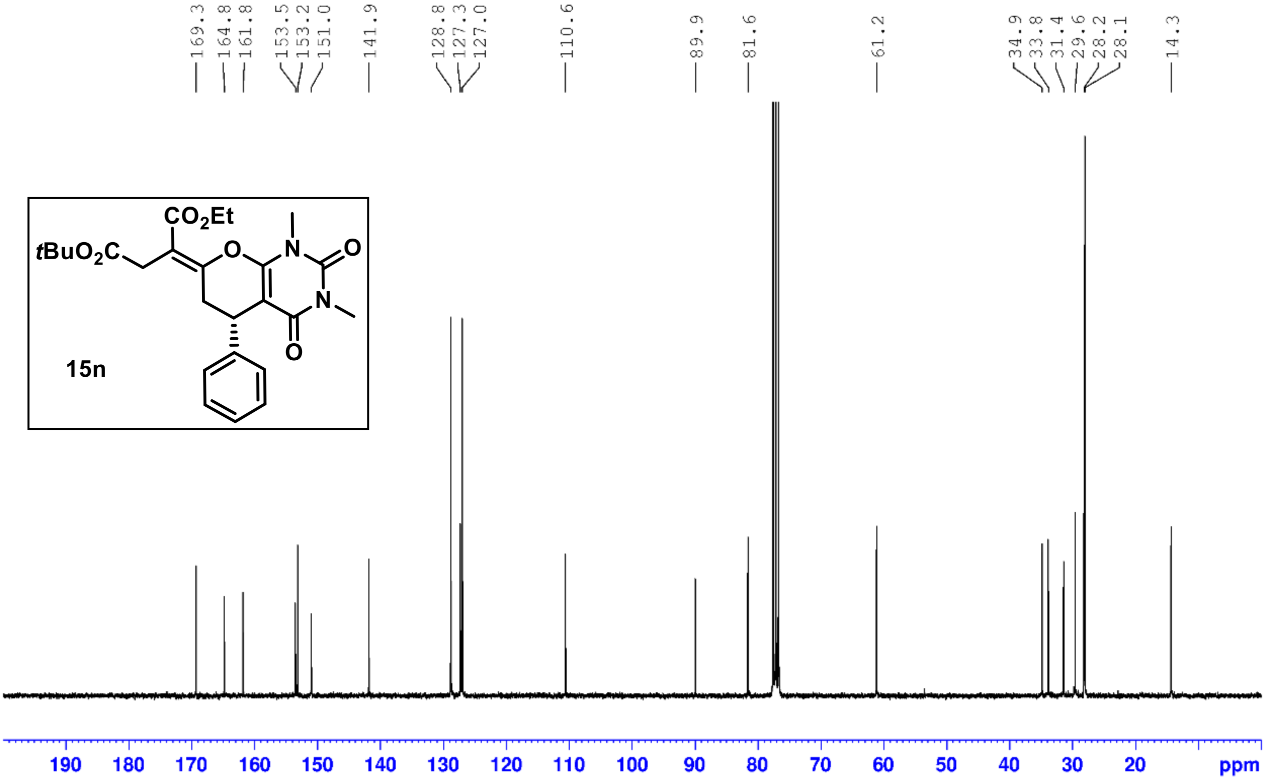
169.3
164.8
161.8
153.5
153.2
151.0
141.9
128.8
127.3
127.0
110.6
89.9
81.6
61.2
34.9
33.8
31.4
29.6
28.2
28.1
14.3
CO2Et
tBuO2C
O
N
O
N
O
15n
190 180 170 160 150 140 130 120 110 100 90 80 70 60 50 40 30 20 ppm

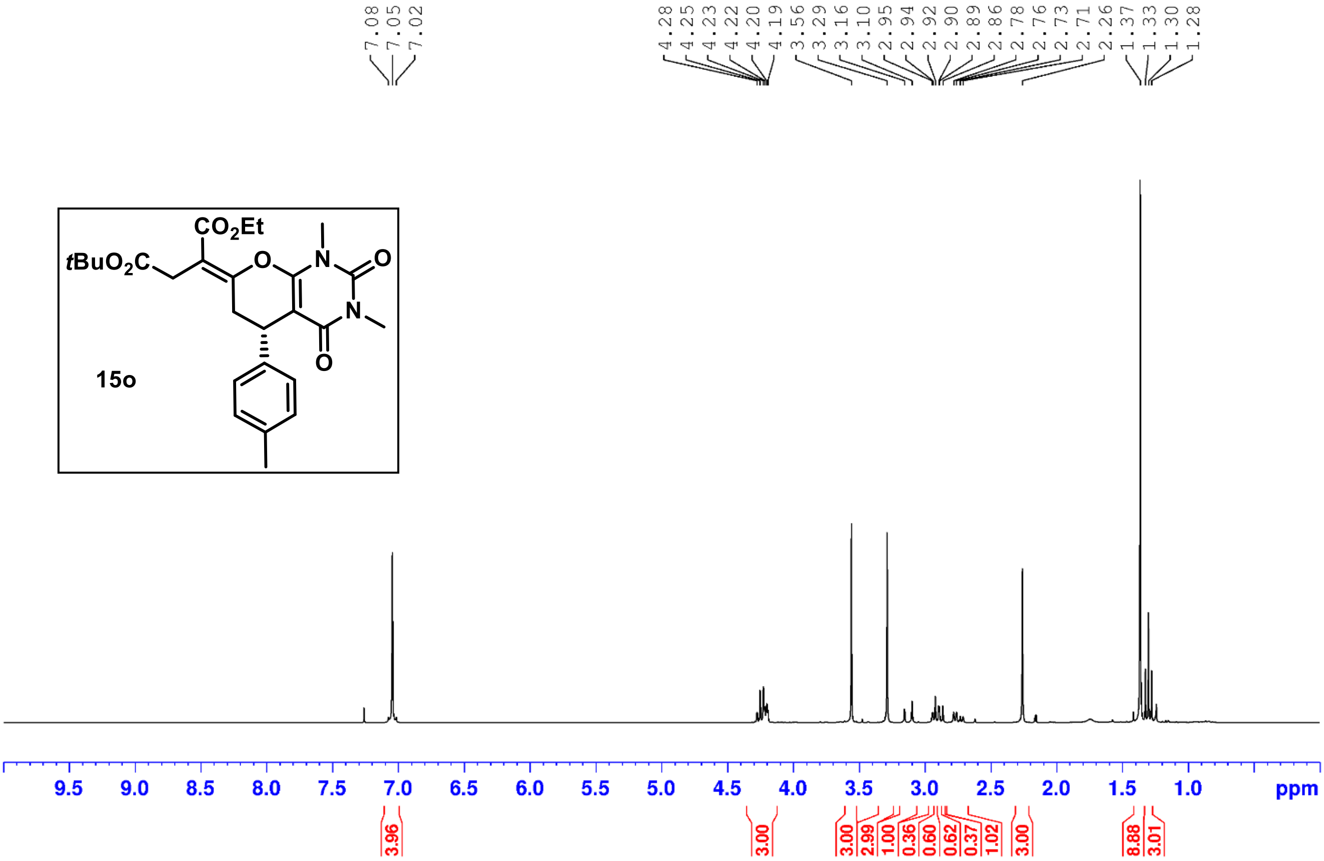
7.08
7.05
7.02
4.28
4.25
4.23
4.22
4.20
4.19
3.56
3.29
3.16
3.10
2.95
2.94
2.92
2.90
2.89
2.86
2.78
2.76
2.73
2.71
2.26
1.37
1.33
1.30
1.28
CO2Et
tBuO2C
O
N
O
N
O
15o
9.5 9.0 8.5 8.0 7.5 7.0 6.5 6.0 5.5 5.0 4.5 4.0 3.5 3.0 2.5 2.0 1.5 1.0 ppm
3.96
3.00
3.00
2.99
1.00
0.36
0.60
0.62
0.37
1.02
3.00
8.88
3.01

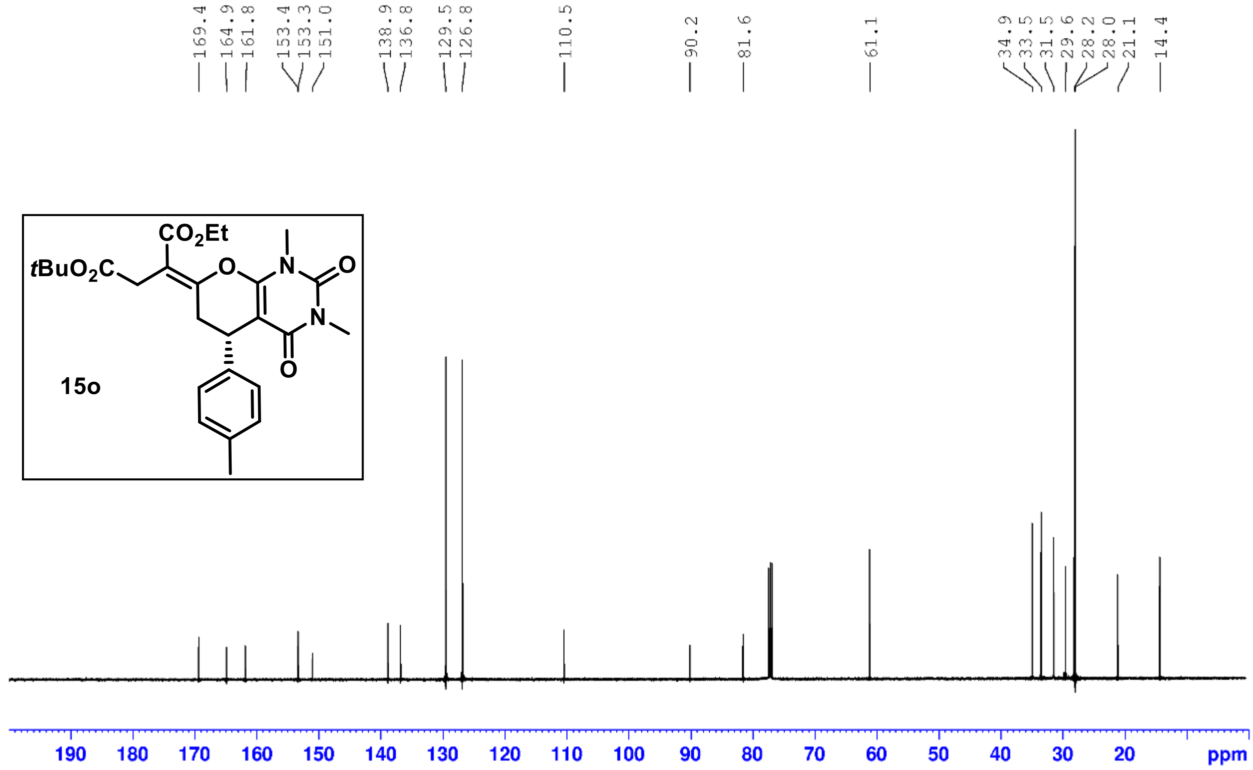
169.4
164.9
161.8
153.4
153.3
151.0
138.9
136.8
129.5
126.8
110.5
90.2
81.6
61.1
34.9
33.5
31.5
29.6
28.2
28.0
21.1
14.4
CO2Et
tBuO2C
15o
ppm

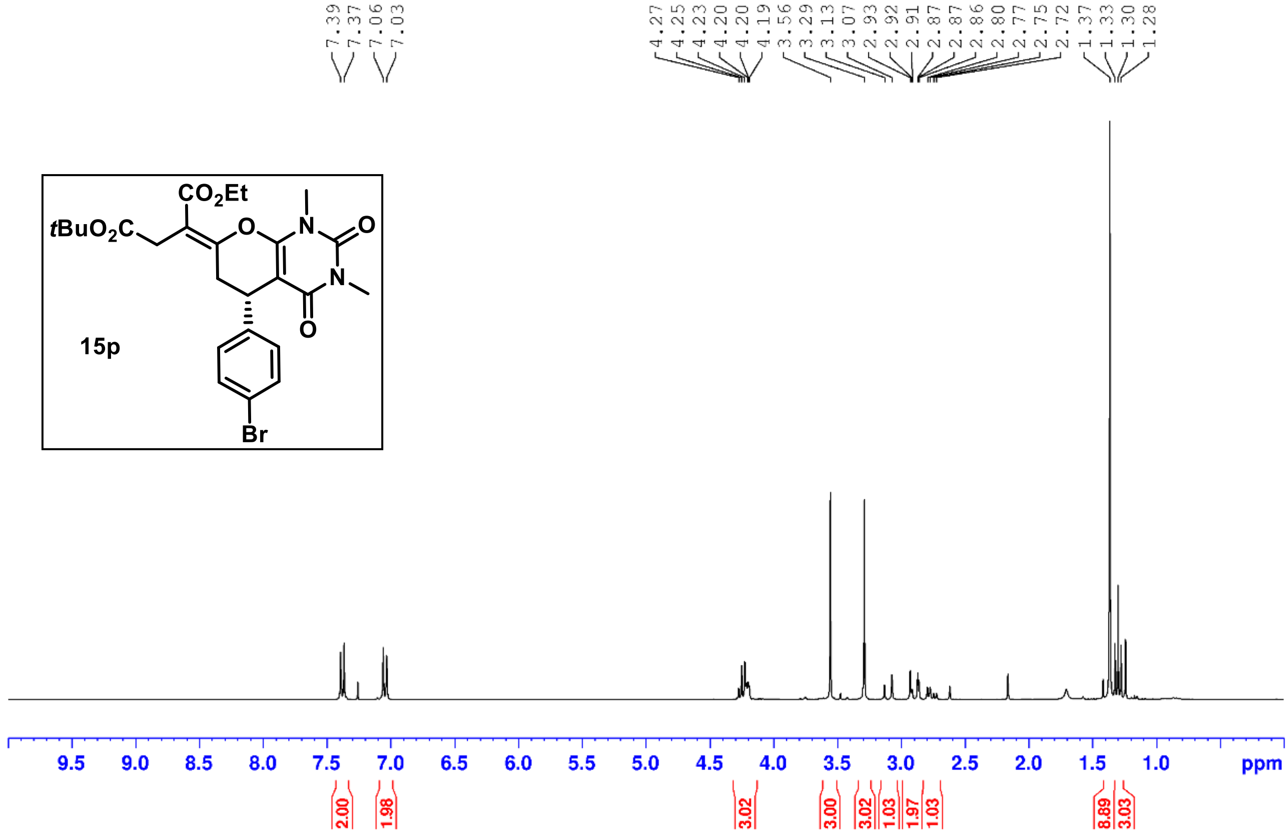
7.39
7.37
7.06
7.03
4.27
4.25
4.23
4.20
4.20
4.19
3.56
3.29
3.13
3.07
2.93
2.92
2.91
2.87
2.87
2.86
2.80
2.77
2.75
2.72
1.37
1.33
1.30
1.28
CO2Et
tBuO2C
15p
Br
ppm
2.00
1.98
3.02
3.00
3.02
1.03
1.97
1.03
8.89
3.03

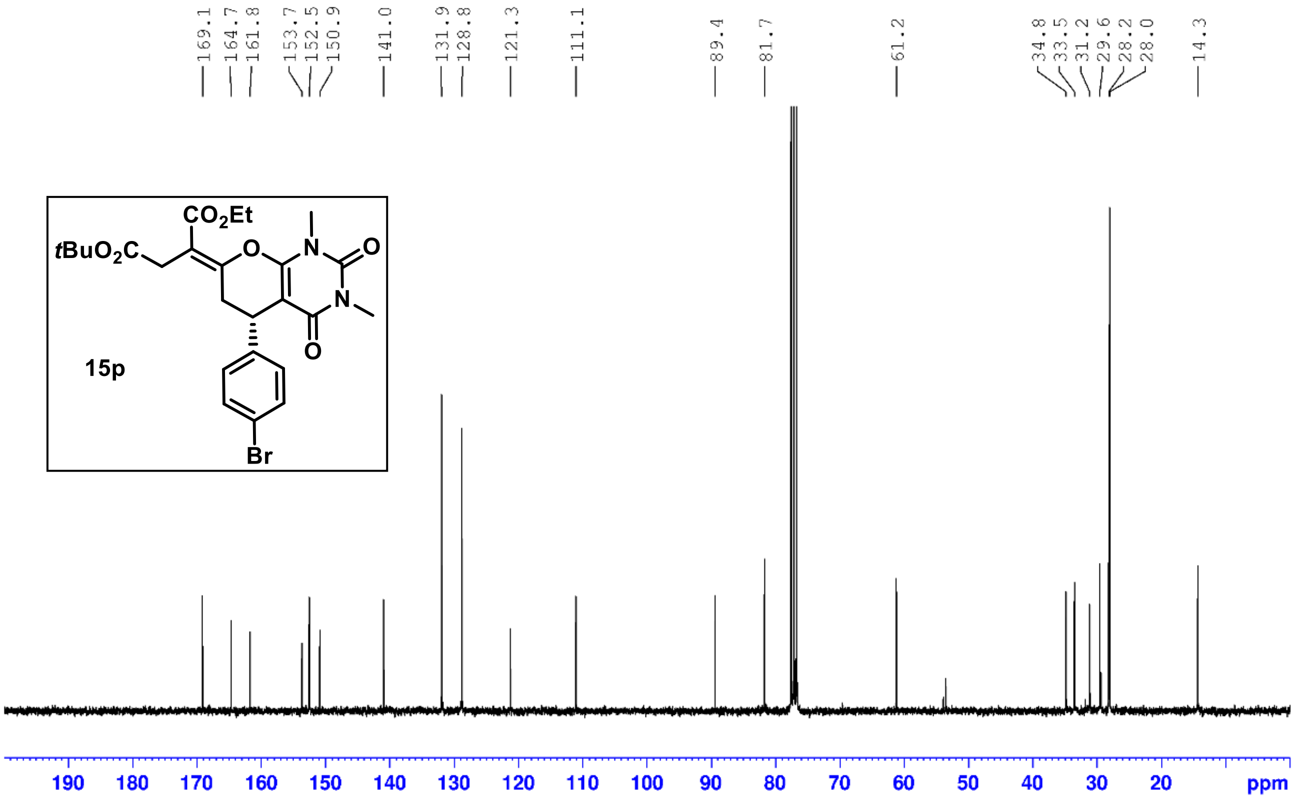
169.1
164.7
161.8
153.7
152.5
150.9
141.0
131.9
128.8
121.3
111.1
89.4
81.7
61.2
34.8
33.5
31.2
29.6
28.2
28.0
14.3
15p
190 180 170 160 150 140 130 120 110 100 90 80 70 60 50 40 30 20
ppm

11.3 NMR spectra of the product diversification

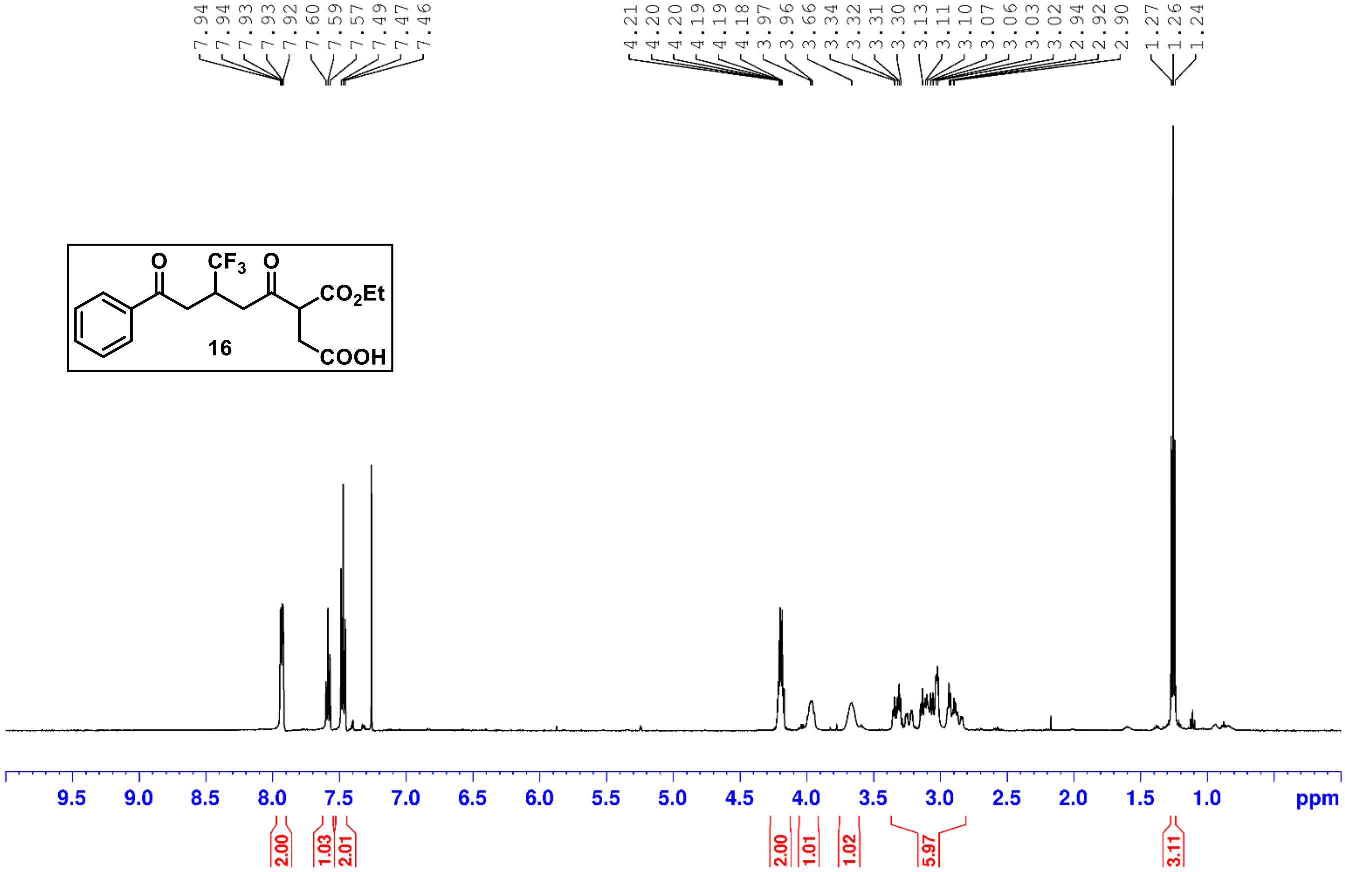

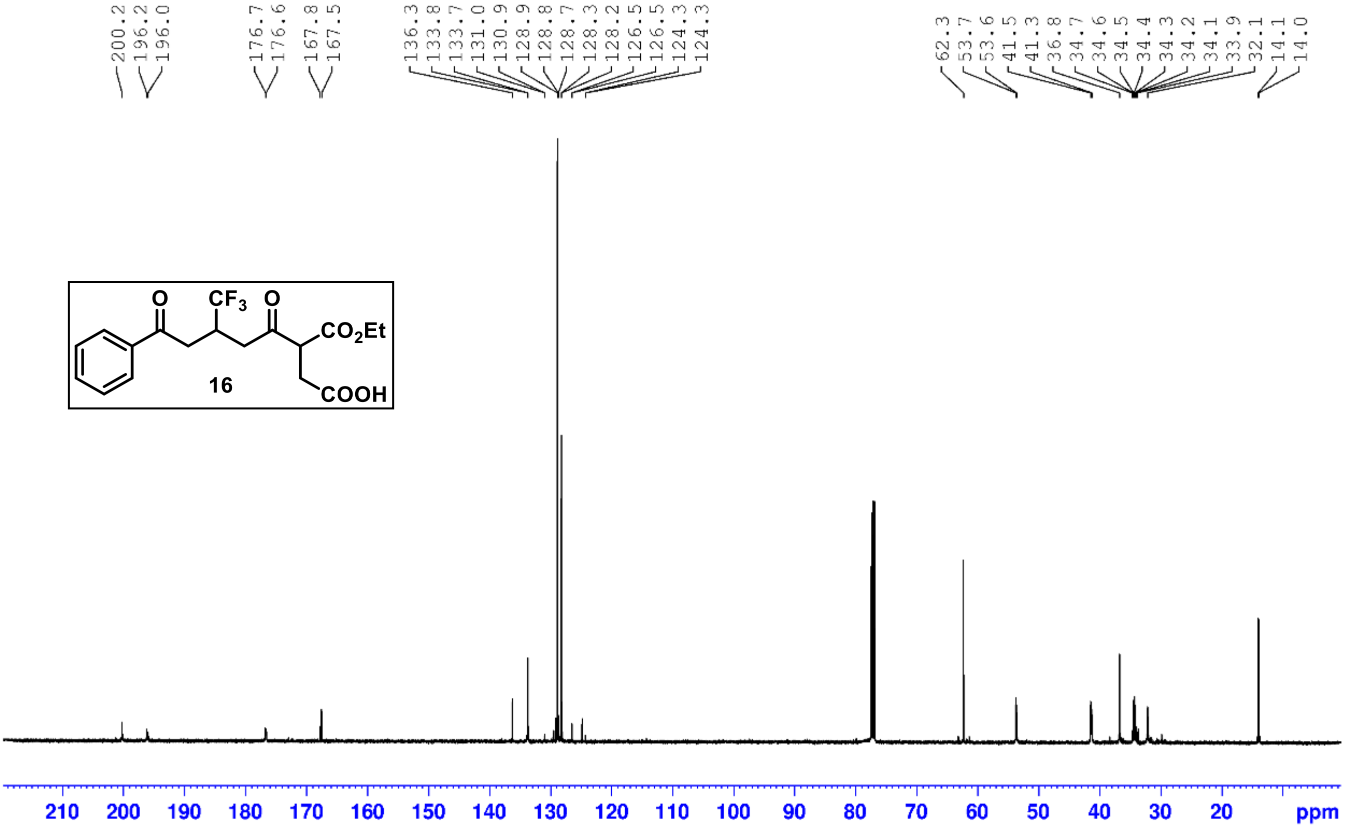

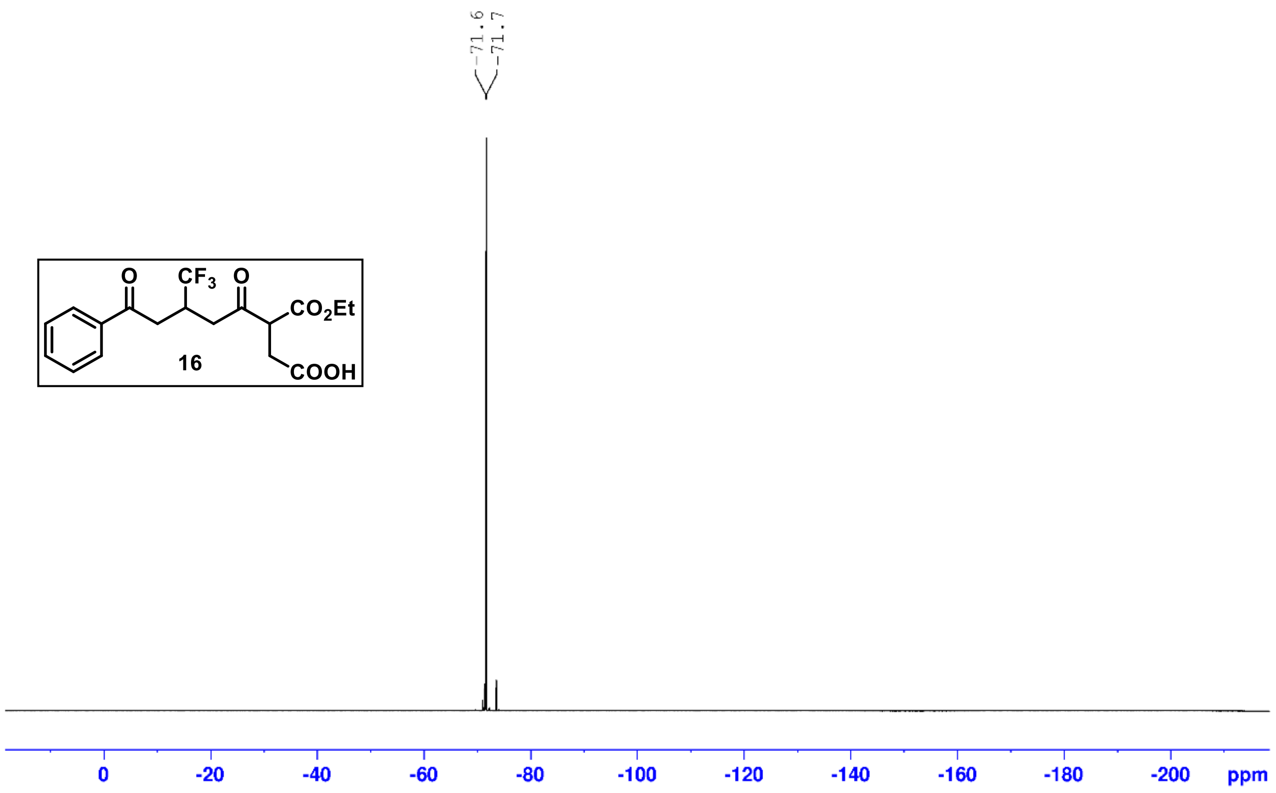
-71.6
-71.7
CF3
CO2Et
COOH
16
ppm

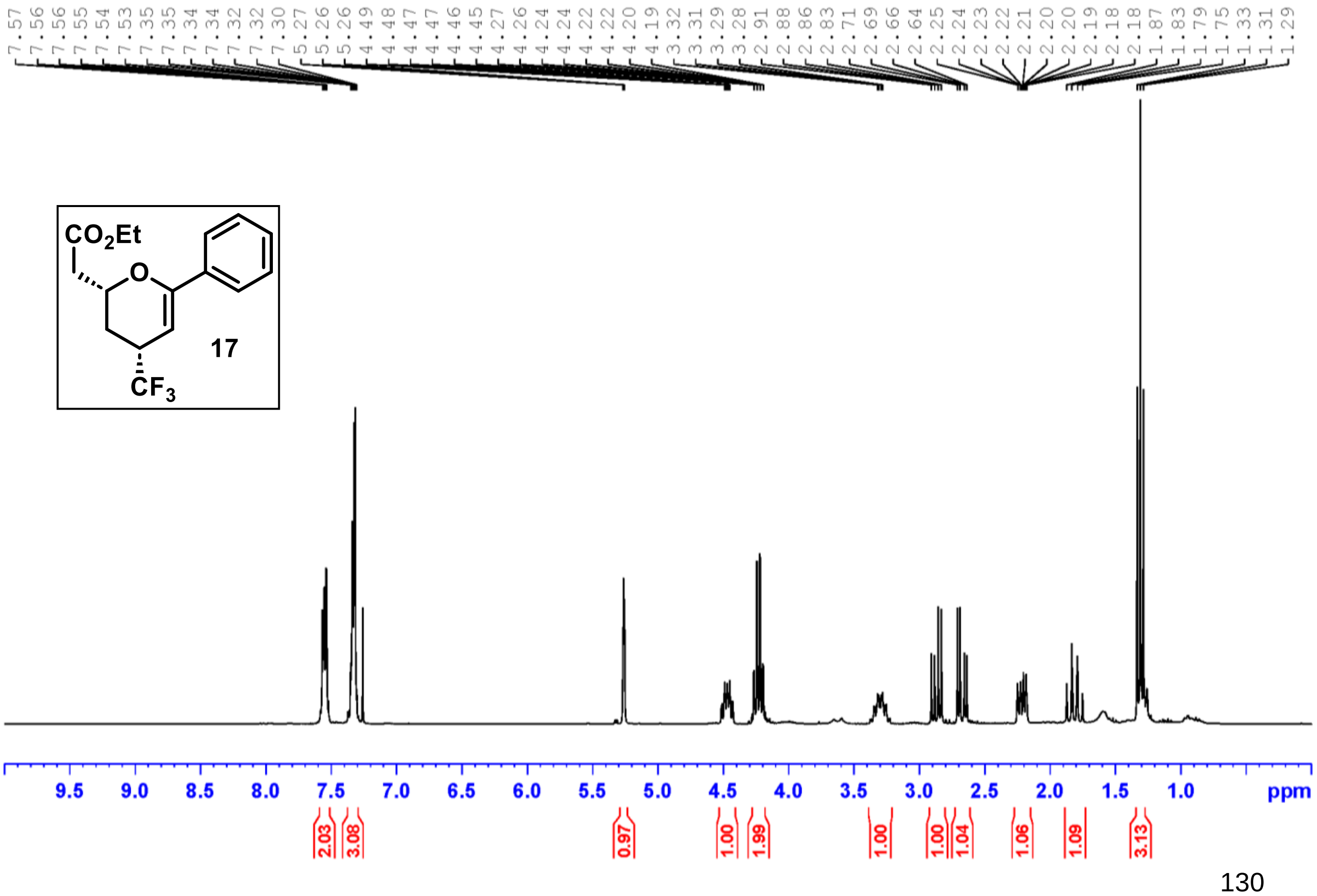
CO2Et
O
17
CF3
ppm

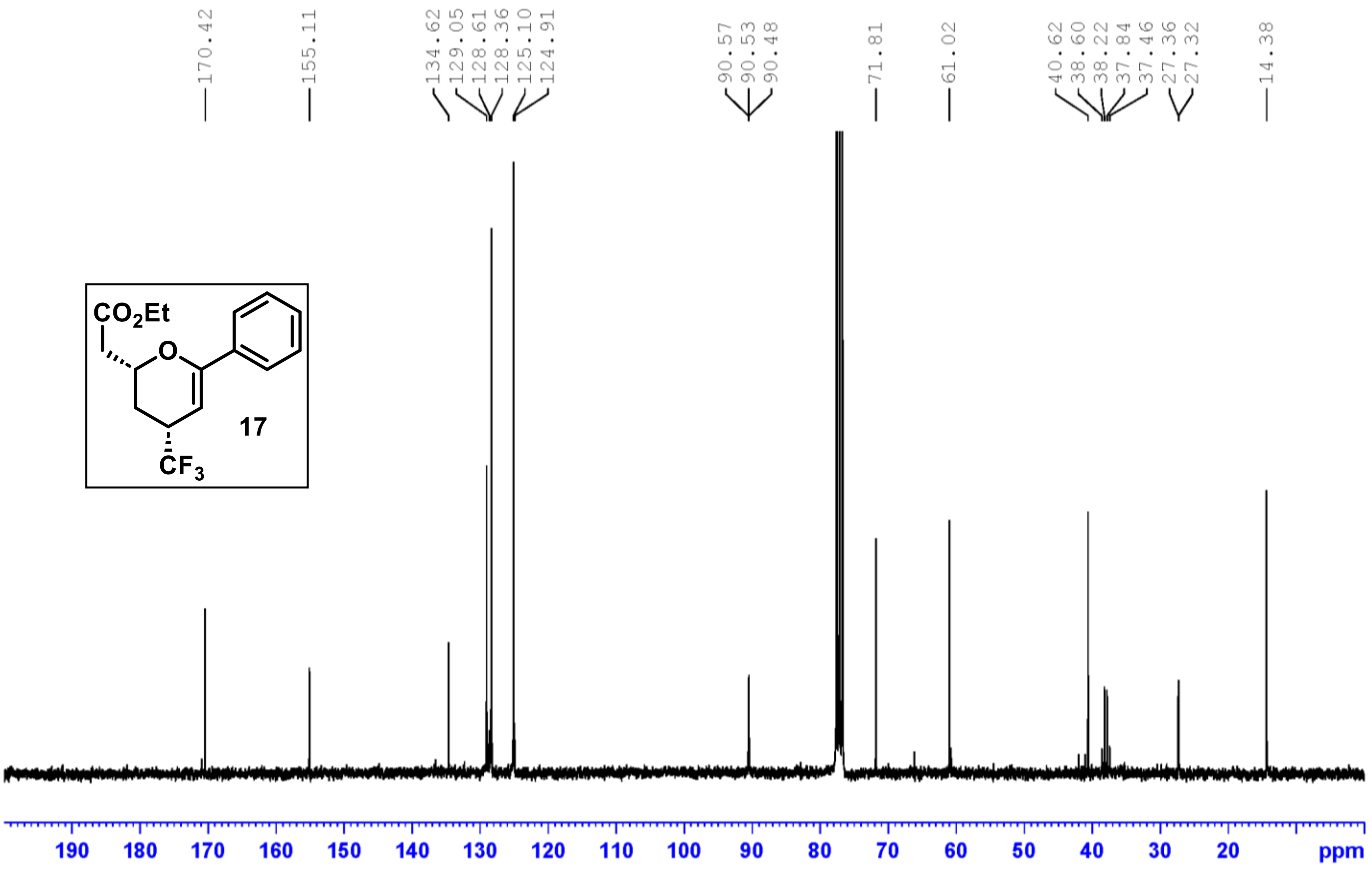
170.42
155.11
134.62
129.05
128.61
128.36
125.10
124.91
90.57
90.53
90.48
71.81
61.02
40.62
38.60
38.22
37.84
37.46
27.36
27.32
14.38
CO2Et
O
17
CF3
190
180
170
160
150
140
130
120
110
100
90
80
70
60
50
40
30
20
ppm

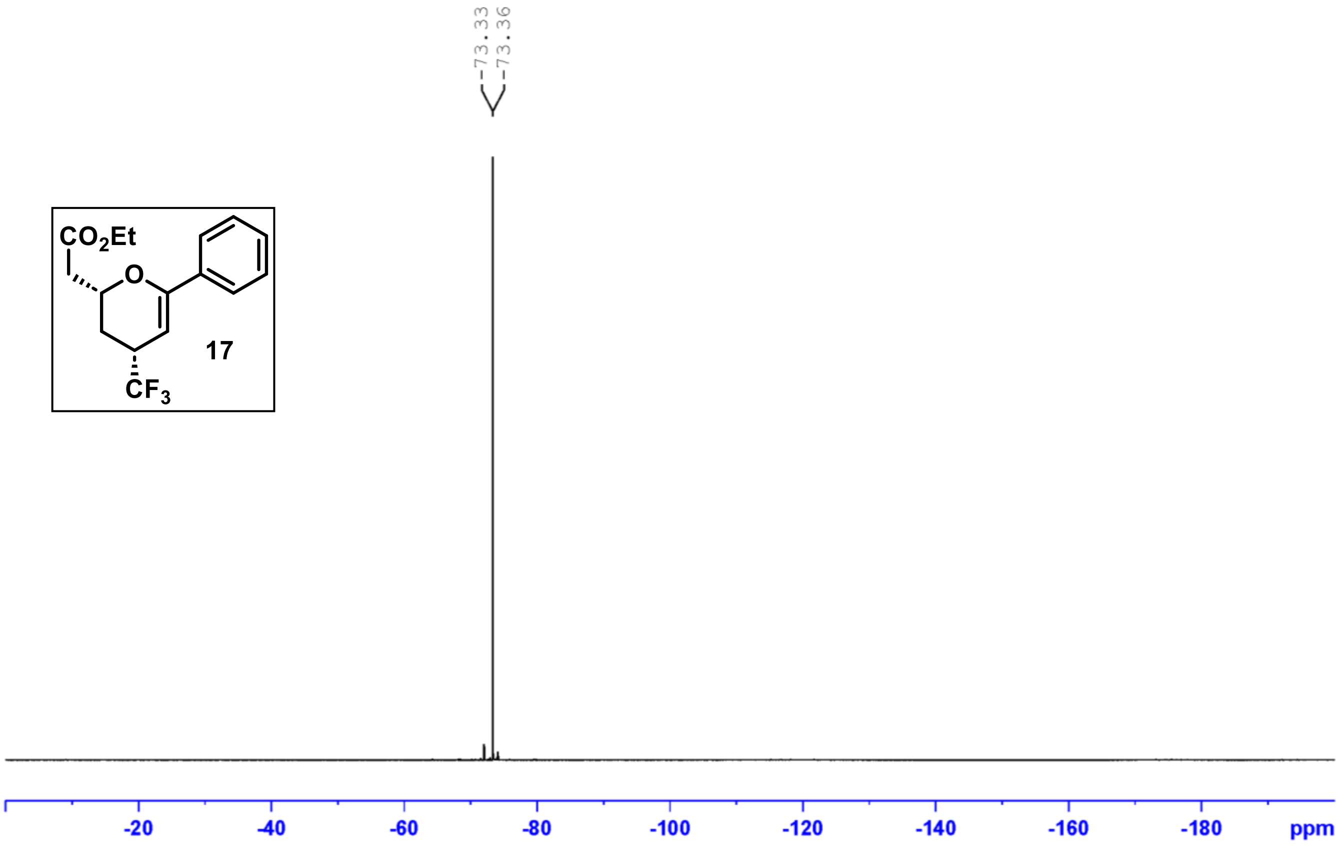
-73.33
-73.36
CO2Et
O
17
CF3
-20
-40
-60
-80
-100
-120
-140
-160
-180
ppm

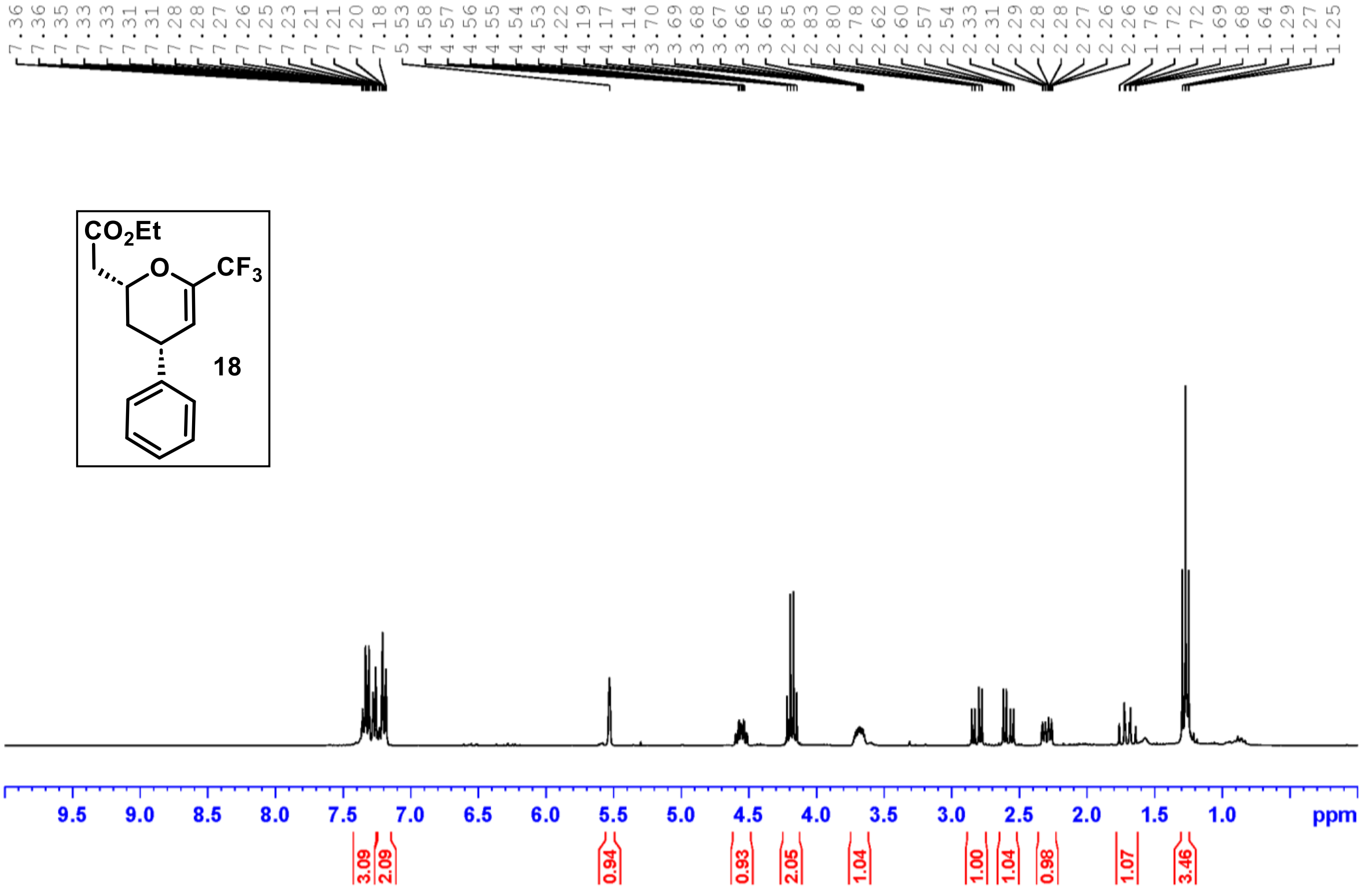
7.36 7.36 7.35 7.33 7.33 7.31 7.28 7.28 7.27 7.26 7.25 7.23 7.21 7.21 7.20 7.18 5.53 4.58 4.57 4.56 4.55 4.54 4.53 4.22 4.19 4.17 4.14 3.70 3.69 3.68 3.67 3.66 3.65 2.85 2.83 2.80 2.78 2.62 2.60 2.57 2.54 2.33 2.31 2.29 2.28 2.28 2.27 2.26 2.26 1.76 1.72 1.72 1.69 1.68 1.64 1.29 1.27 1.25
CO2Et
O
CF3
18
9.5 9.0 8.5 8.0 7.5 7.0 6.5 6.0 5.5 5.0 4.5 4.0 3.5 3.0 2.5 2.0 1.5 1.0 ppm
3.09 2.09 0.94 0.93 2.05 1.04 1.00 1.04 0.98 1.07 3.46

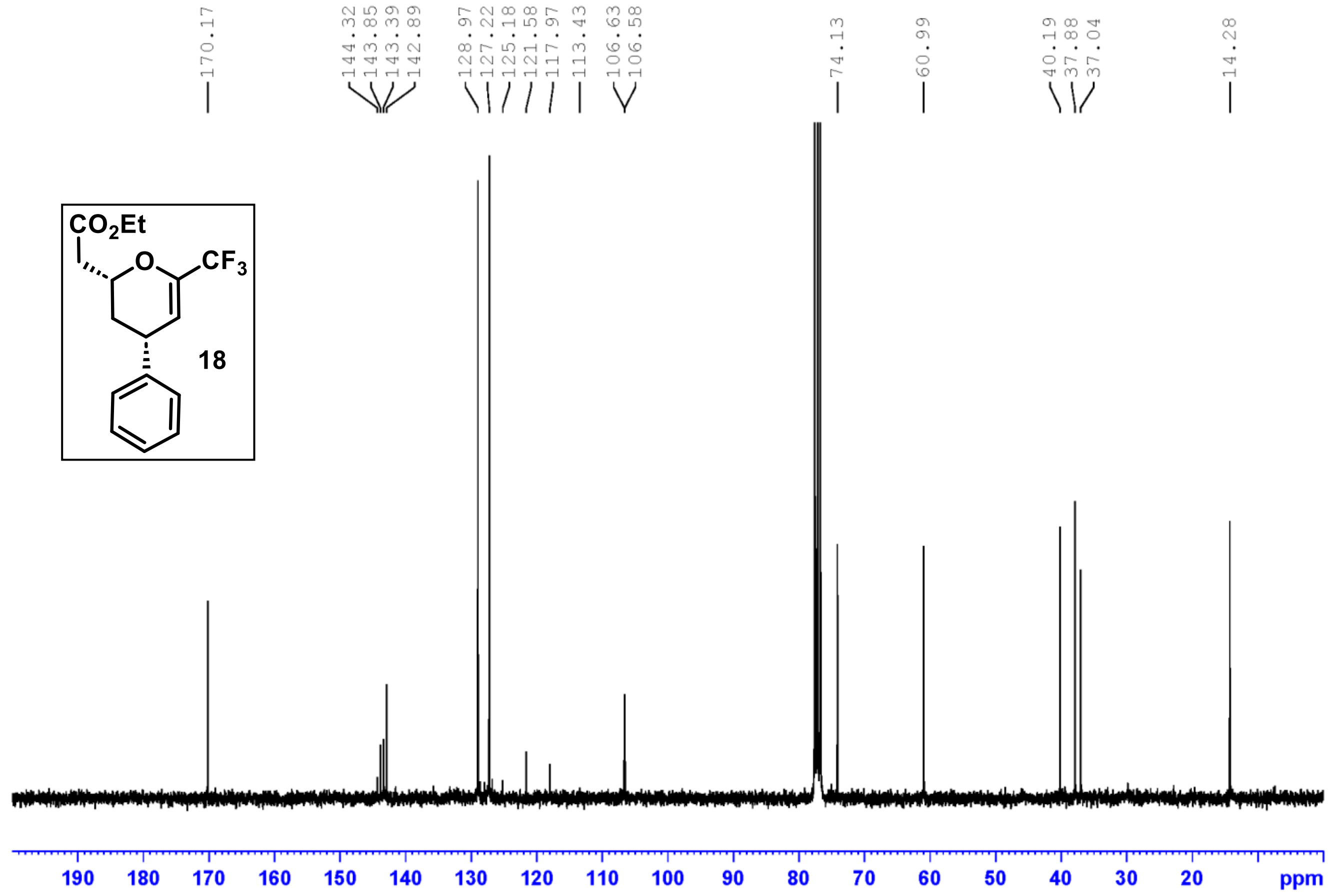
170.17
144.32 143.85 143.39 142.89
128.97 127.22 125.18 121.58 117.97 113.43 106.63 106.58
74.13
60.99
40.19 37.88 37.04
14.28
CO2Et
O
CF3
18
190 180 170 160 150 140 130 120 110 100 90 80 70 60 50 40 30 20 ppm

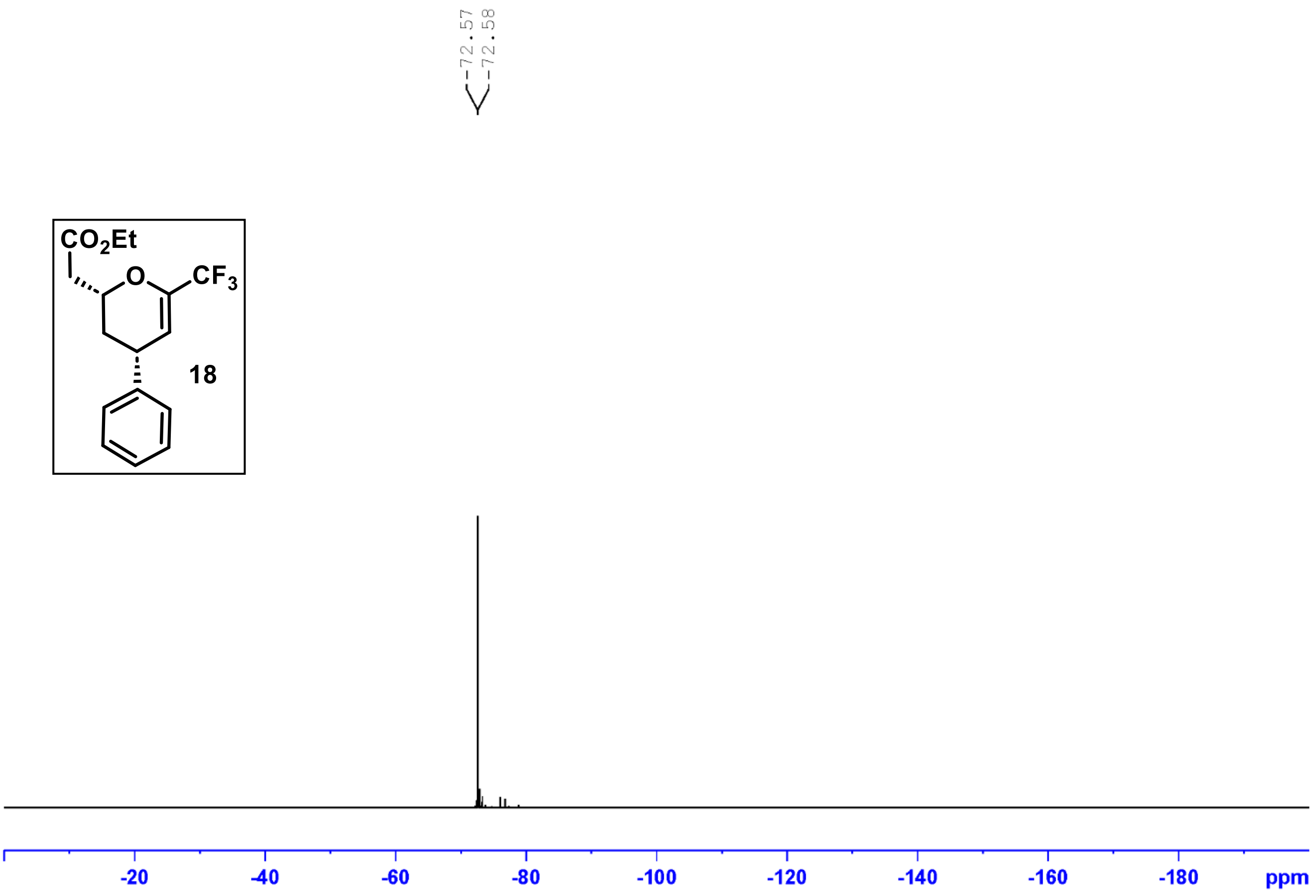

-72.57
-72.58
CO2Et
O
CF3
18
-20
-40
-60
-80
-100
-120
-140
-160
-180
ppm

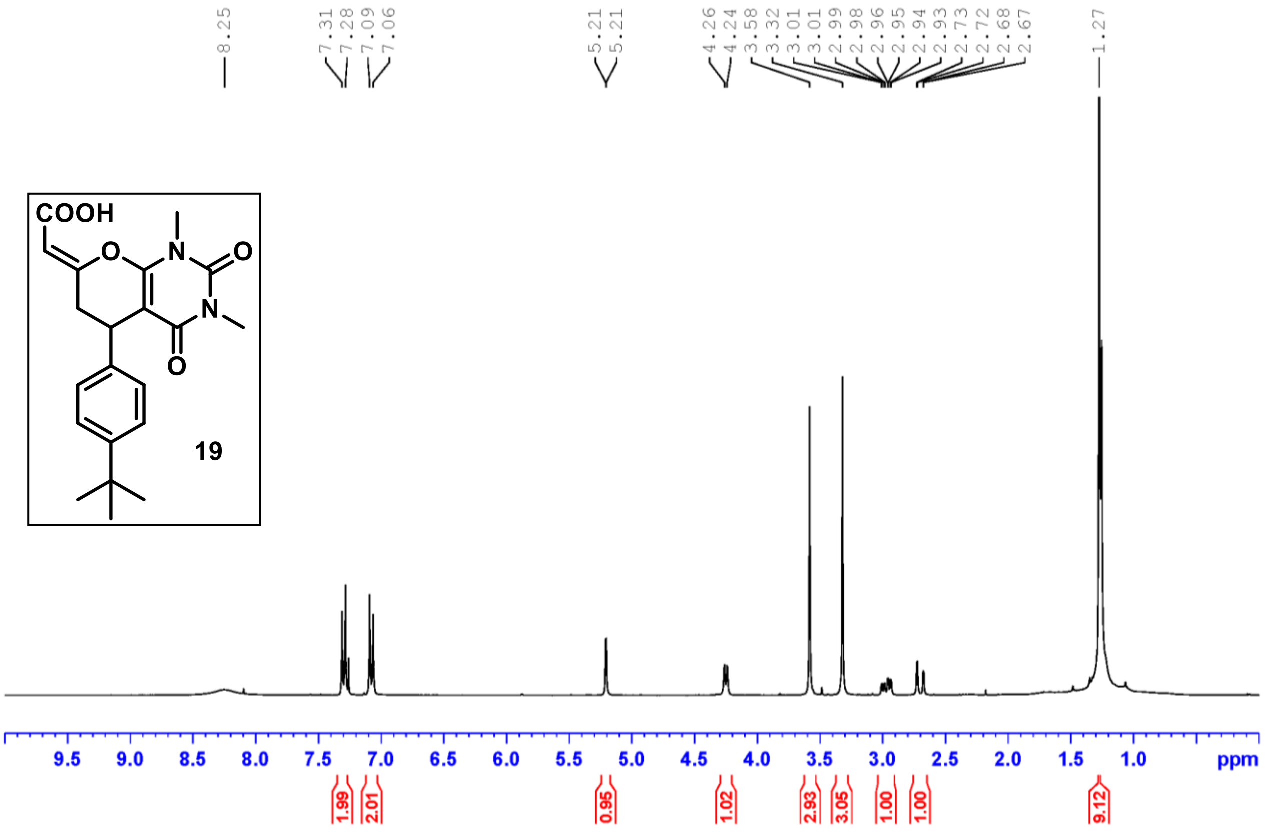

8.25
7.31
7.28
7.09
7.06
5.21
5.21
4.26
4.24
3.58
3.32
3.01
2.99
2.98
2.96
2.95
2.94
2.93
2.73
2.72
2.68
2.67
1.27
COOH
O
N
O
N
O
19
9.5
9.0
8.5
8.0
7.5
7.0
6.5
6.0
5.5
5.0
4.5
4.0
3.5
3.0
2.5
2.0
1.5
1.0
ppm
1.99
2.01
0.95
1.02
2.93
3.05
1.00
1.00
9.12

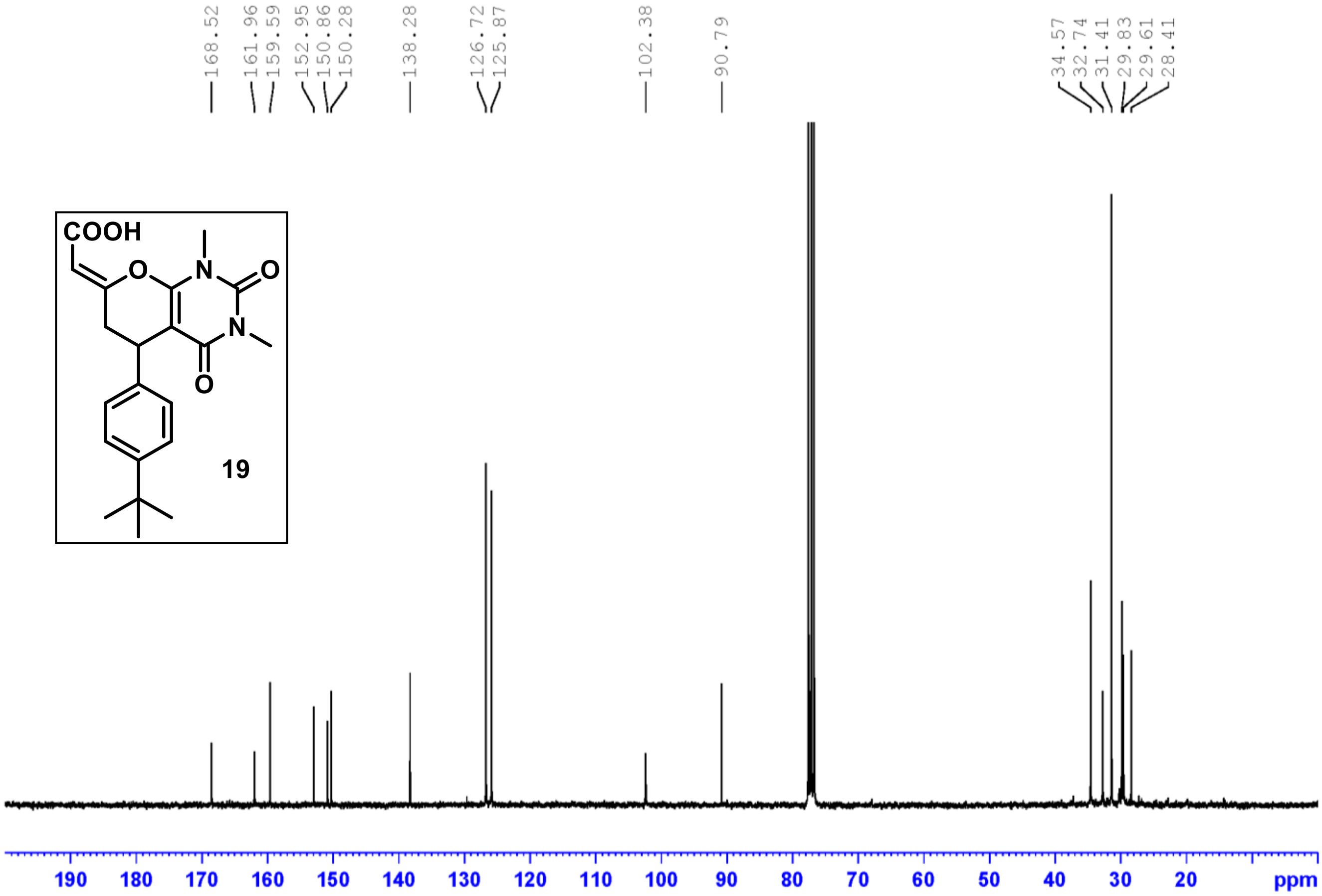
168.52
161.96
159.59
152.95
150.86
150.28
138.28
126.72
125.87
102.38
90.79
34.57
32.74
31.41
29.83
29.61
28.41
COOH
O
N
O
N
O
19
190 180 170 160 150 140 130 120 110 100 90 80 70 60 50 40 30 20 ppm

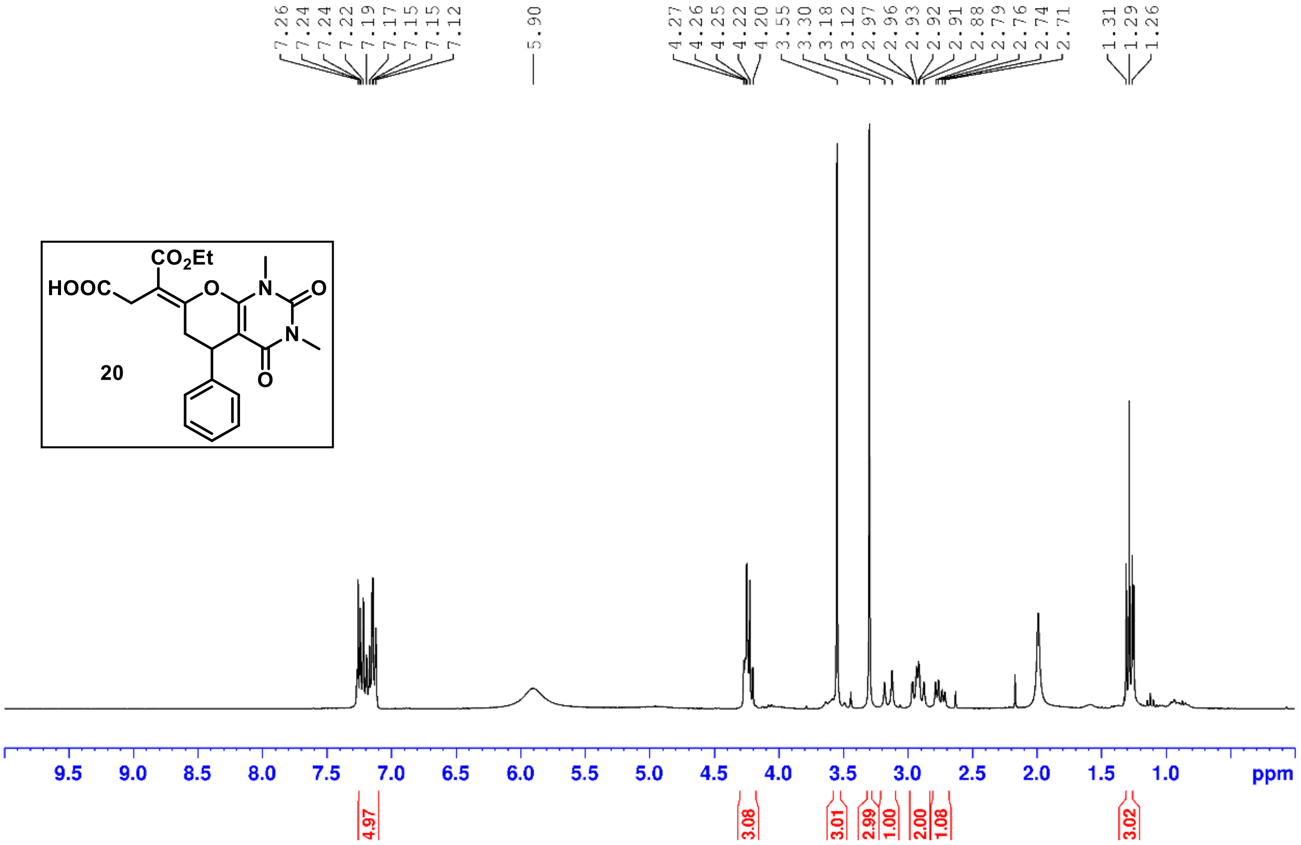
7.26
7.24
7.24
7.22
7.19
7.17
7.15
7.15
7.12
5.90
4.27
4.26
4.25
4.22
4.20
3.55
3.30
3.18
3.12
2.97
2.96
2.93
2.92
2.91
2.88
2.79
2.76
2.74
2.71
1.31
1.29
1.26
CO_2Et
HOOC
O
N
O
N
O
20
9.5 9.0 8.5 8.0 7.5 7.0 6.5 6.0 5.5 5.0 4.5 4.0 3.5 3.0 2.5 2.0 1.5 1.0 ppm
4.97
3.08
3.01
2.99
1.00
2.00
1.08
3.02

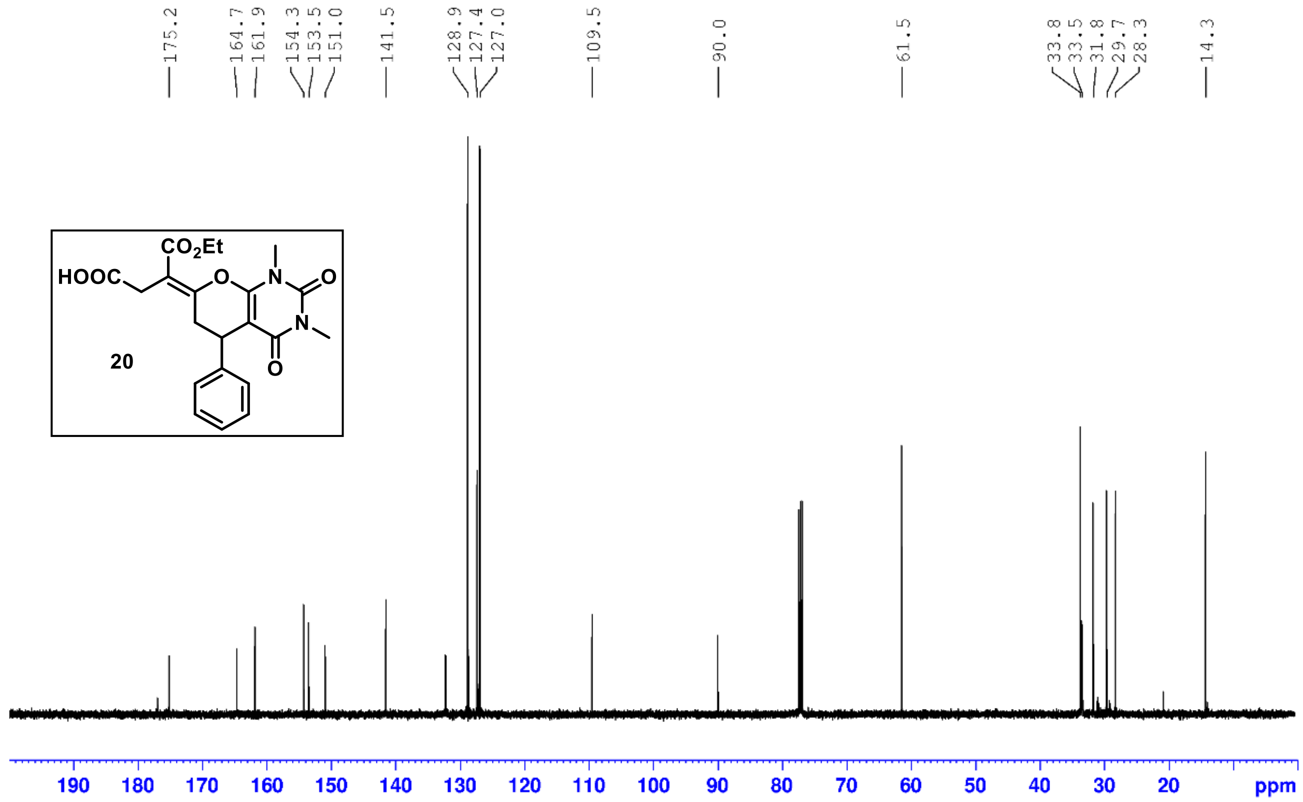
175.2
164.7
161.9
154.3
153.5
151.0
141.5
128.9
127.4
127.0
109.5
90.0
61.5
33.8
33.5
31.8
29.7
28.3
14.3
CO_2Et
HOOC
O
N
O
N
O
20
190 180 170 160 150 140 130 120 110 100 90 80 70 60 50 40 30 20 ppm

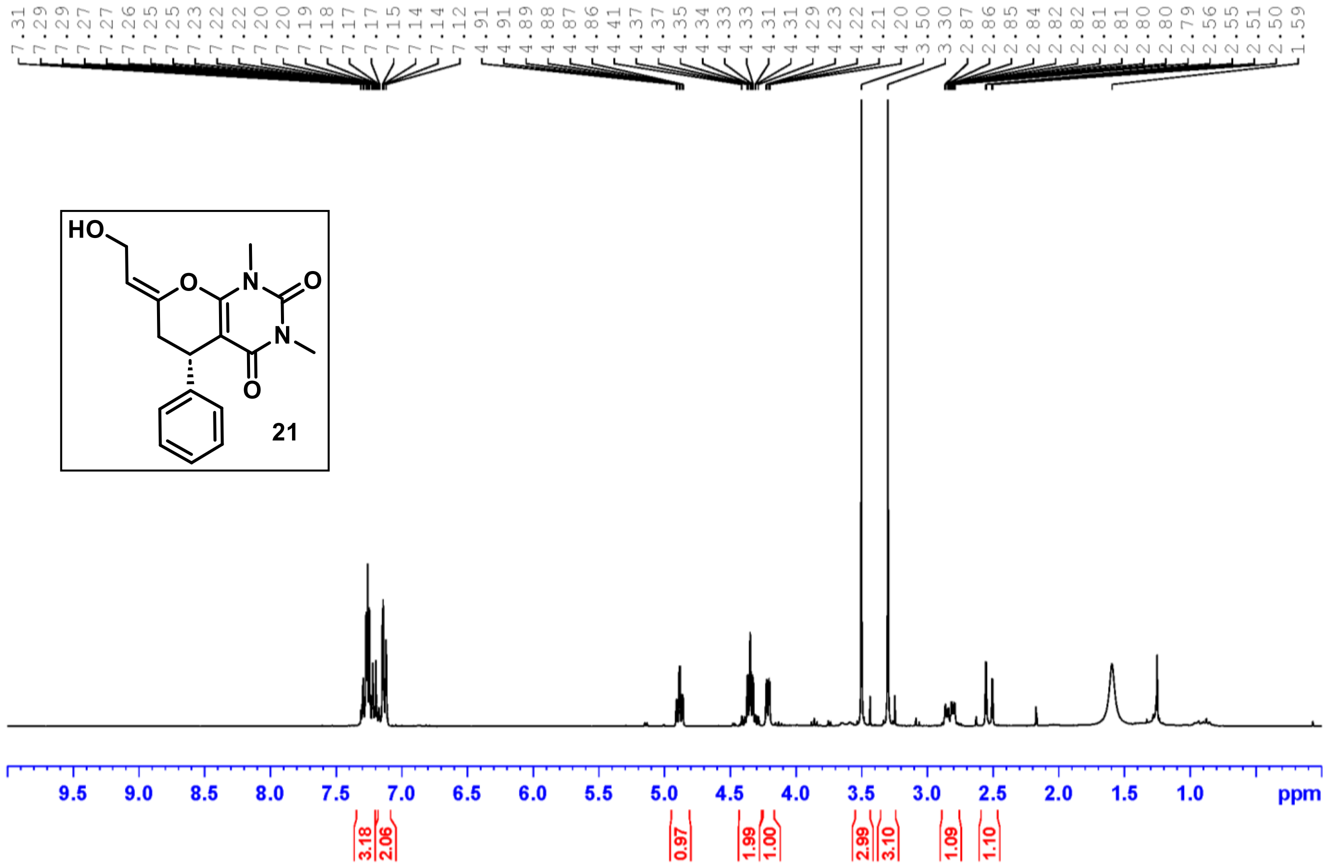
7.31 7.29 7.29 7.27 7.27 7.26 7.25 7.25 7.23 7.22 7.22 7.20 7.20 7.19 7.18 7.17 7.17 7.15 7.14 7.14 7.12
4.91 4.91 4.89 4.88 4.87 4.86 4.41 4.37 4.37 4.35 4.34 4.33 4.33 4.33 4.31 4.31 4.29 4.23 4.22 4.21 4.20
3.50 3.30
2.87 2.86 2.85 2.84 2.82 2.82 2.81 2.81 2.80 2.80 2.79 2.56 2.55 2.51 2.50
1.59
HO
O
N
O
N
O
21
9.5 9.0 8.5 8.0 7.5 7.0 6.5 6.0 5.5 5.0 4.5 4.0 3.5 3.0 2.5 2.0 1.5 1.0 ppm
3.18
2.06
0.97
1.99
1.00
2.99
3.10
1.09
1.10

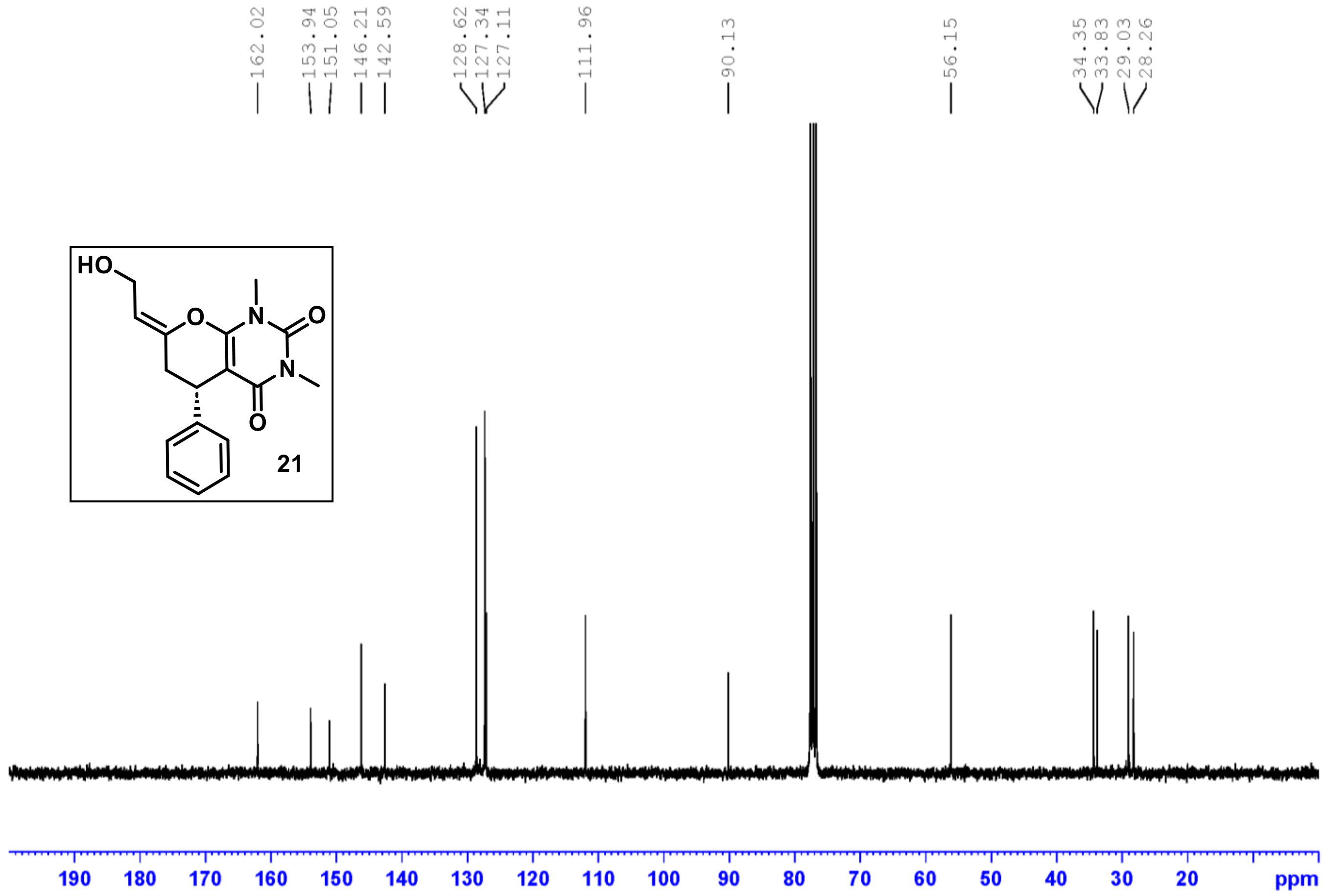
162.02
153.94
151.05
146.21
142.59
128.62
127.34
127.11
111.96
90.13
56.15
34.35
33.83
29.03
28.26
HO
O
N
O
N
O
21
190 180 170 160 150 140 130 120 110 100 90 80 70 60 50 40 30 20 ppm

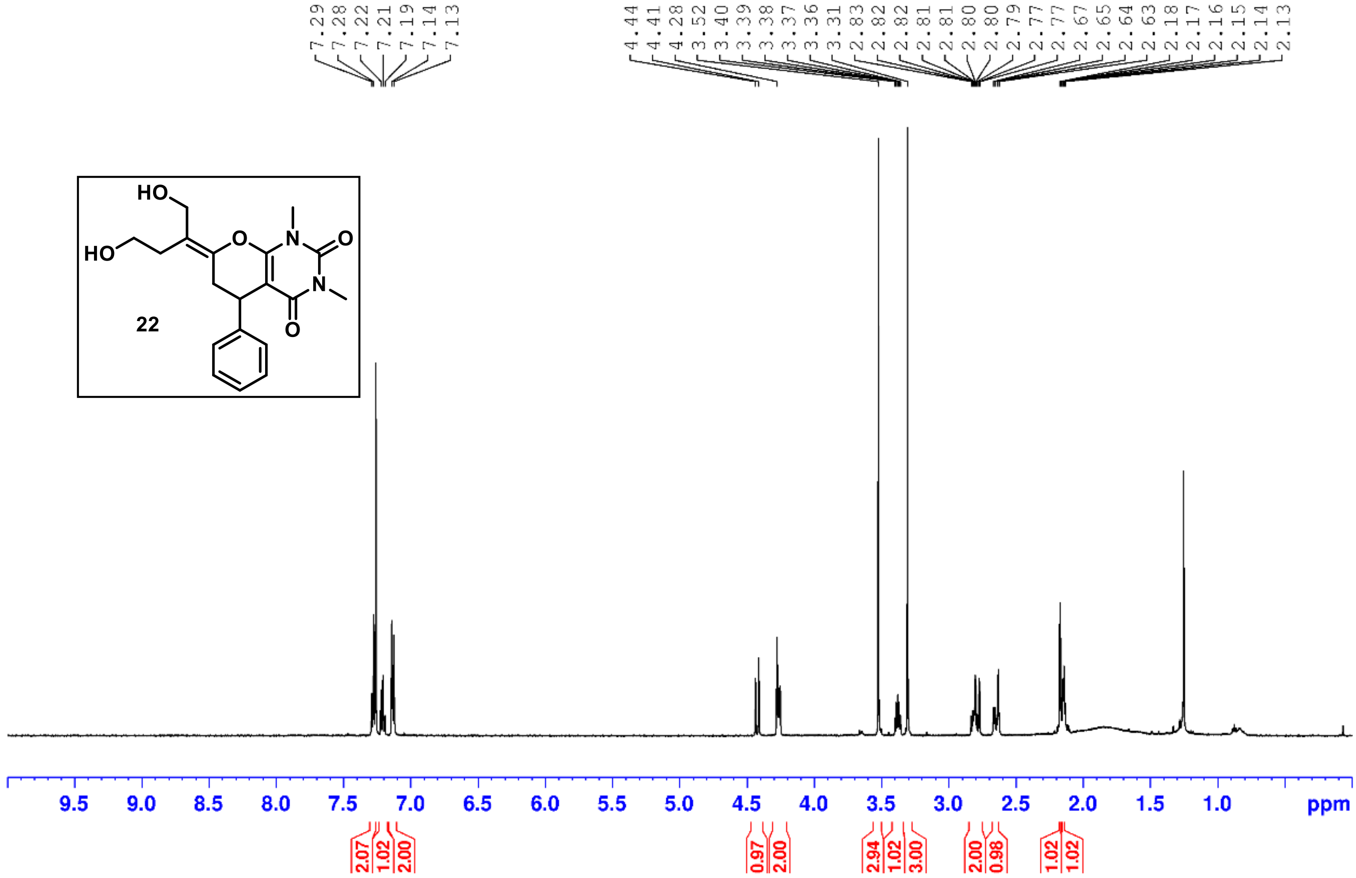
7.29
7.28
7.22
7.21
7.19
7.14
7.13
4.44
4.41
4.28
3.52
3.40
3.39
3.38
3.37
3.36
3.31
2.83
2.82
2.82
2.81
2.80
2.80
2.79
2.77
2.77
2.67
2.65
2.64
2.63
2.18
2.17
2.16
2.15
2.14
2.13
HO
HO
O
N
O
N
O
22
9.5 9.0 8.5 8.0 7.5 7.0 6.5 6.0 5.5 5.0 4.5 4.0 3.5 3.0 2.5 2.0 1.5 1.0 ppm
2.07
1.02
2.00
0.97
2.00
2.94
1.02
3.00
2.00
0.98
1.02
1.02

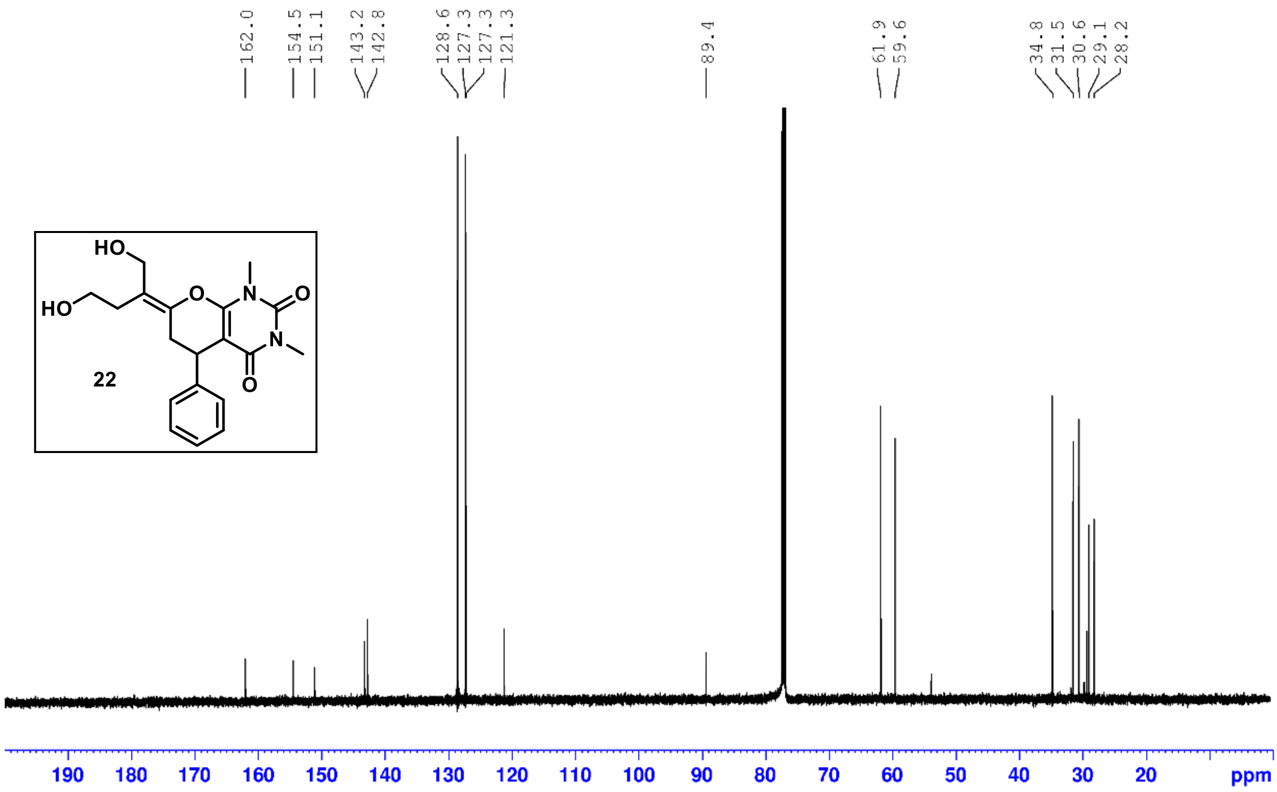
162.0
154.5
151.1
143.2
142.8
128.6
127.3
127.3
121.3
89.4
61.9
59.6
34.8
31.5
30.6
29.1
28.2
HO
HO
O
N
O
N
O
22
190 180 170 160 150 140 130 120 110 100 90 80 70 60 50 40 30 20 ppm

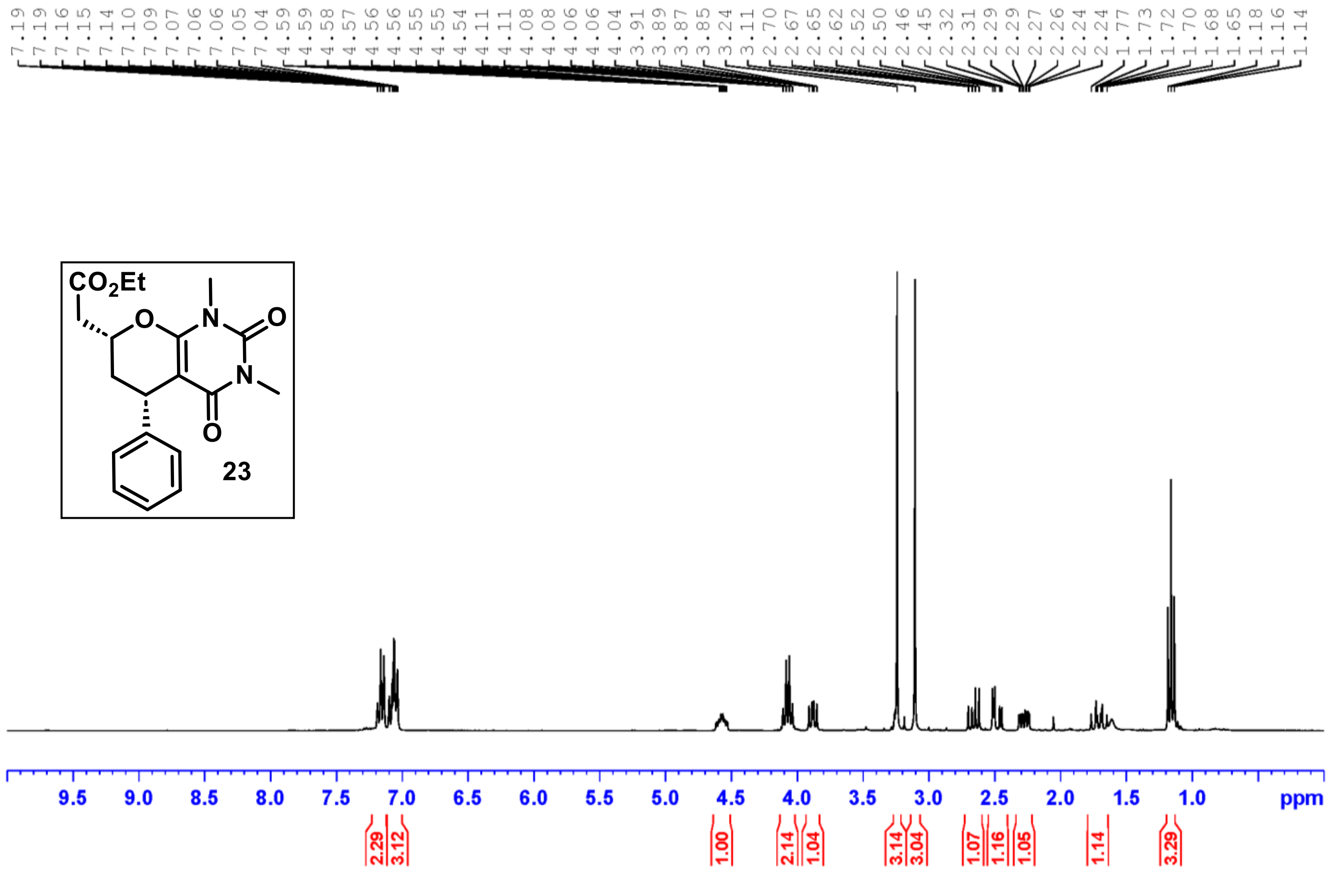
7.19 7.19 7.16 7.15 7.14 7.10 7.09 7.07 7.06 7.06 7.05 7.04 4.59 4.59 4.58 4.57 4.56 4.56 4.55 4.55 4.54 4.11 4.11 4.08 4.08 4.06 4.06 4.04 3.91 3.89 3.87 3.85 3.24 3.11 2.70 2.67 2.65 2.62 2.52 2.50 2.46 2.45 2.32 2.31 2.29 2.29 2.27 2.26 2.24 2.24 1.77 1.73 1.72 1.70 1.68 1.65 1.18 1.16 1.14
CO_2Et
O
N
O
N
O
23
9.5 9.0 8.5 8.0 7.5 7.0 6.5 6.0 5.5 5.0 4.5 4.0 3.5 3.0 2.5 2.0 1.5 1.0 ppm
2.29 3.12 1.00 2.14 1.04 3.14 3.04 1.07 1.16 1.05 1.14 3.29

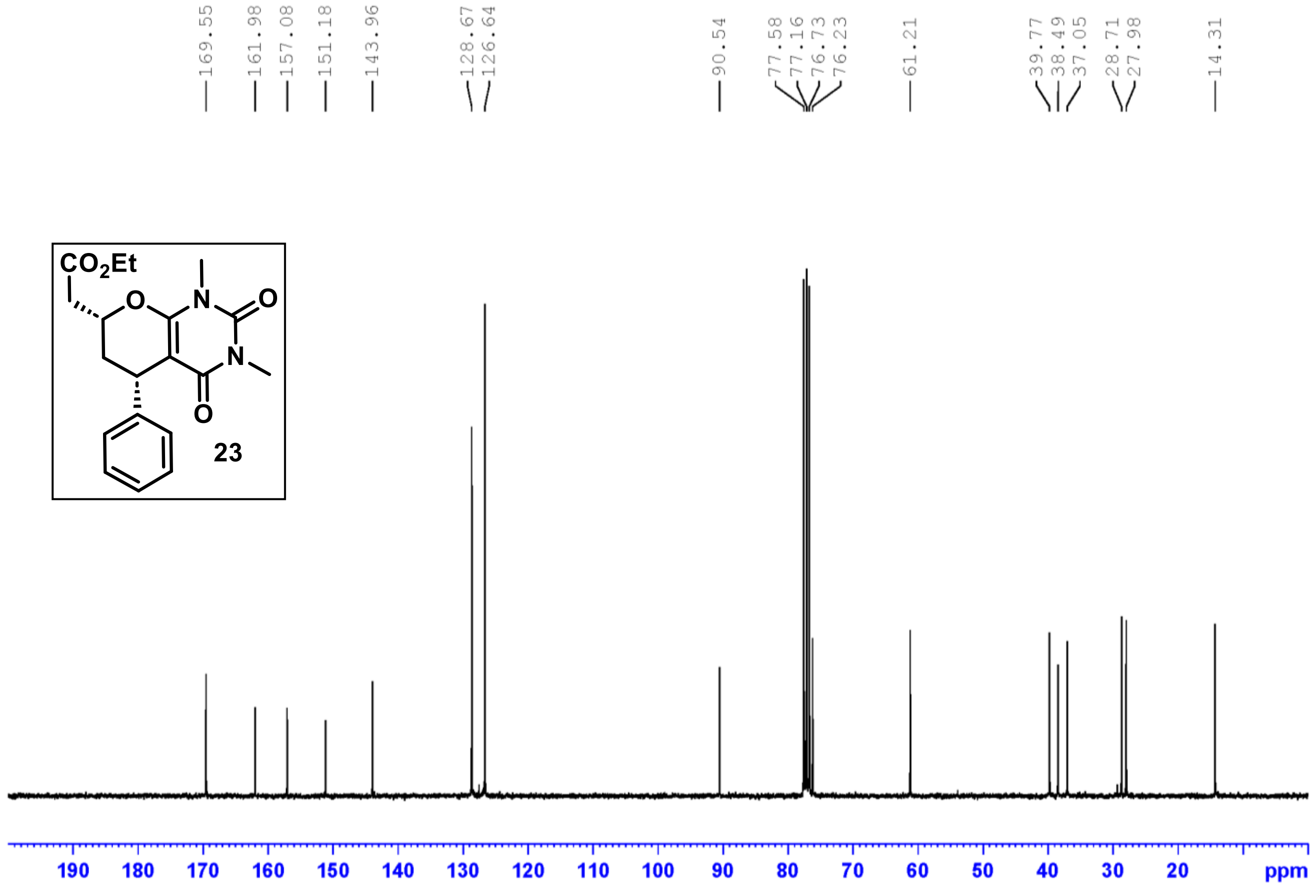
169.55
161.98
157.08
151.18
143.96
128.67
126.64
90.54
77.58
77.16
76.73
76.23
61.21
39.77
38.49
37.05
28.71
27.98
14.31
CO2Et
23
190 180 170 160 150 140 130 120 110 100 90 80 70 60 50 40 30 20 ppm

11.4 HPLC chromatograms of the cyclization products

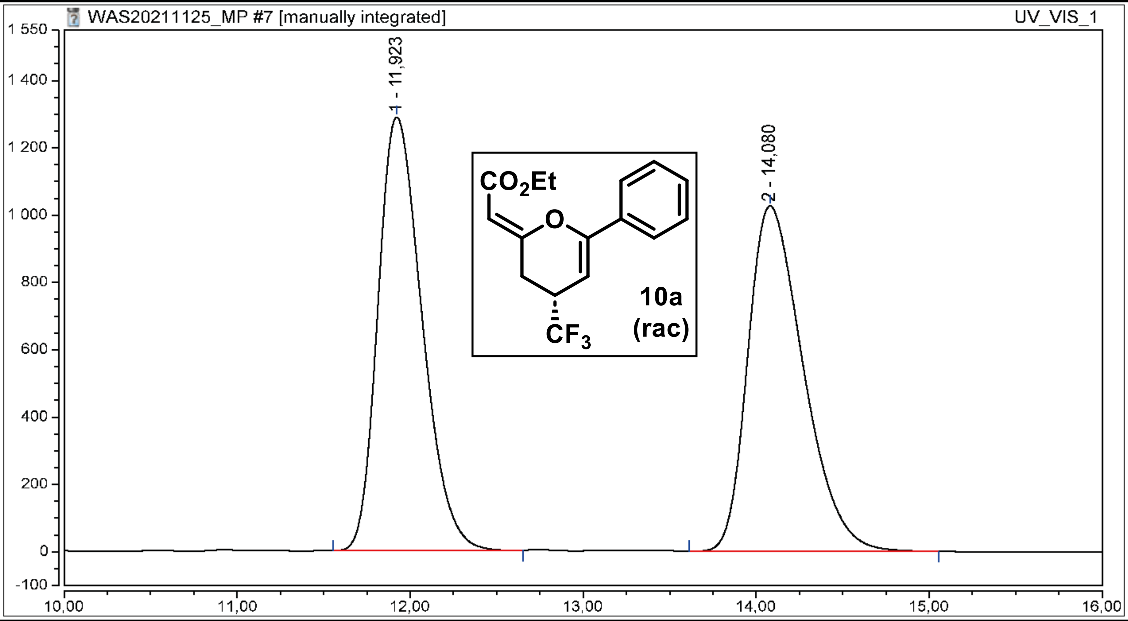

Integration Results

No.	Peak Name	Retention Time<br>min	Area<br>mAU*min	Height<br>mAU	Relative Area<br>%	Relative Height<br>%	Amount<br>n.a.
1		11,923	379,485	1288,376	49,89	55,66	n.a.
2		14,080	381,098	1026,226	50,11	44,34	n.a.
**Total:**			**760,584**	**2314,601**	**100,00**	**100,00**	

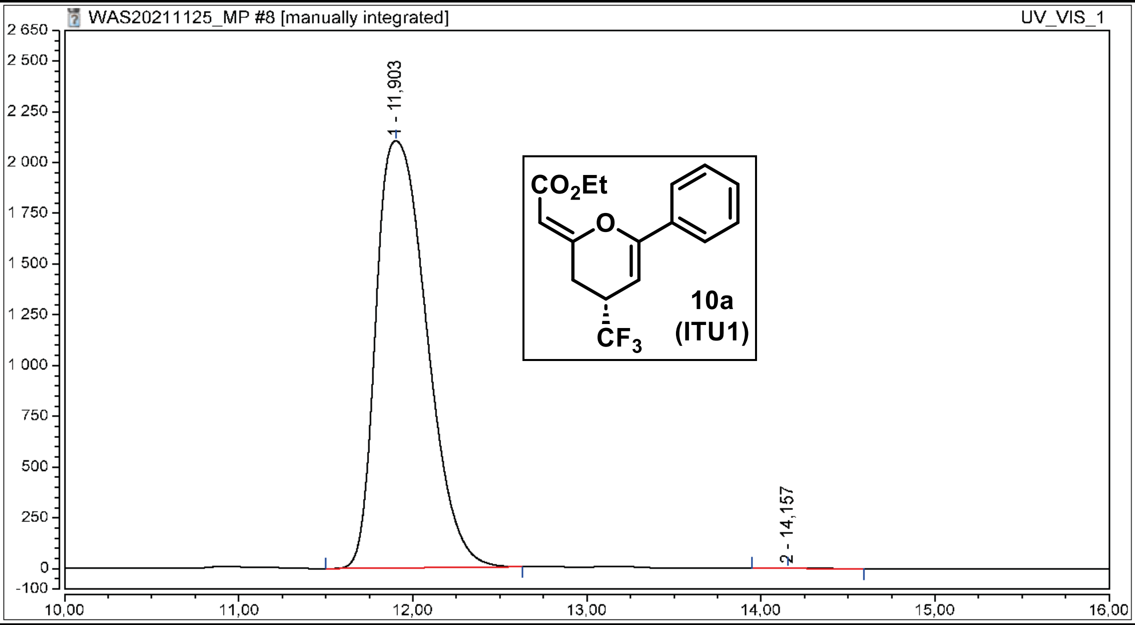

Integration Results

No.	Peak Name	Retention Time<br>min	Area<br>mAU*min	Height<br>mAU	Relative Area<br>%	Relative Height<br>%	Amount<br>n.a.
1		11,903	708,348	2102,868	99,89	99,89	n.a.
2		14,157	0,792	2,380	0,11	0,11	n.a.
**Total:**			**709,140**	**2105,248**	**100,00**	**100,00**	

Chromatogram

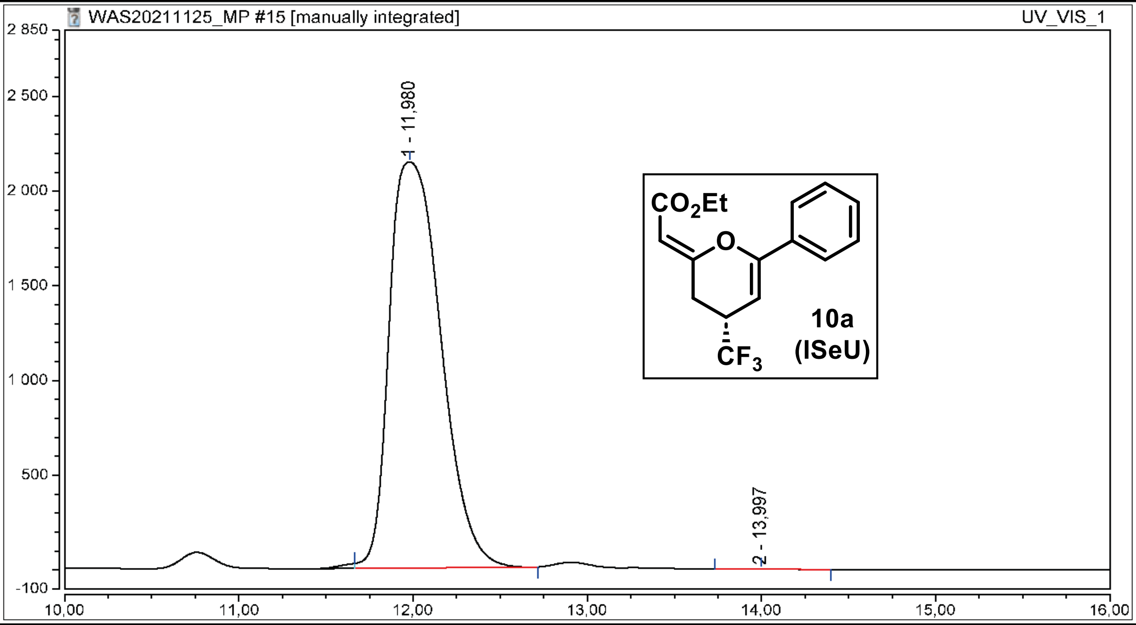

Integration Results

No.	Peak Name	Retention Time min	Area mAU*min	Height mAU	Relative Area %	Relative Height %	Amount n.a.
1		11,980	737,938	2144,788	99,94	99,93	n.a.
2		13,997	0,462	1,605	0,06	0,07	n.a.
**Total:**			**738,400**	**2146,393**	**100,00**	**100,00**	

Chromatogram

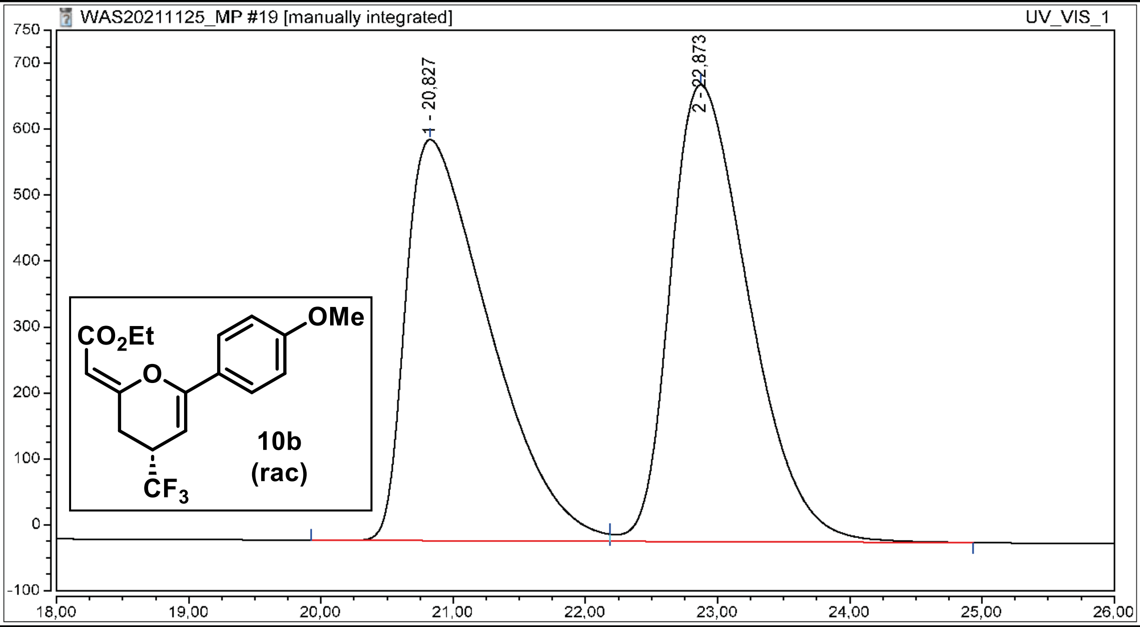

Integration Results

No.	Peak Name	Retention Time<br>min	Area<br>mAU*min	Height<br>mAU	Relative Area<br>%	Relative Height<br>%	Amount<br>n.a.
1		20,827	446,556	608,130	48,34	46,75	n.a.
2		22,873	477,136	692,679	51,66	53,25	n.a.
**Total:**			**923,693**	**1300,809**	**100,00**	**100,00**	

Chromatogram

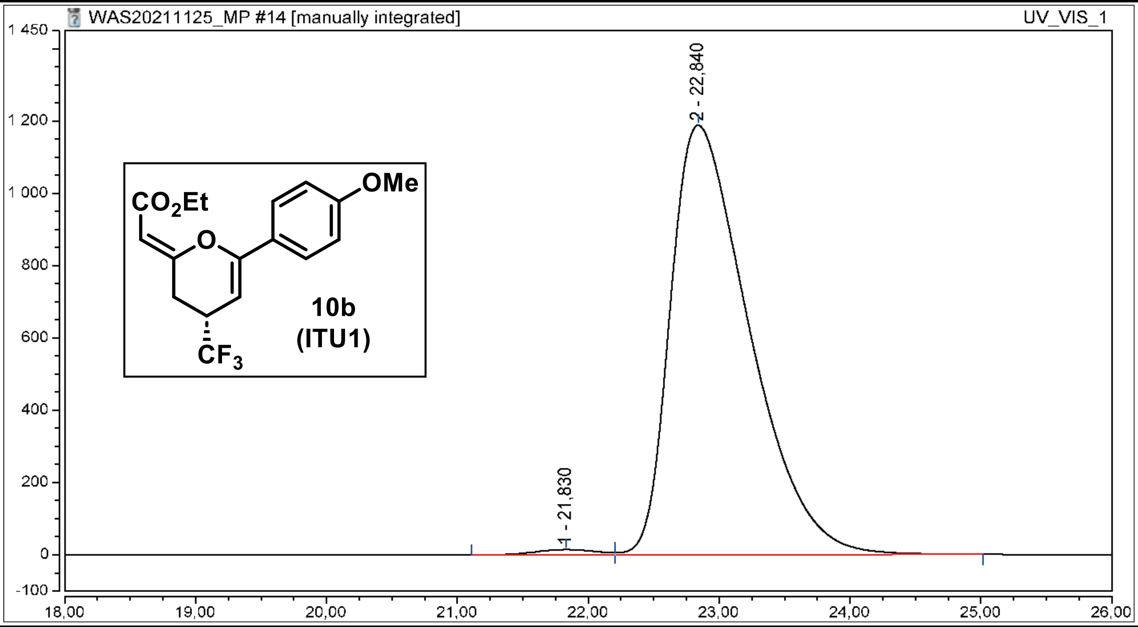

Integration Results

No.	Peak Name	Retention Time<br>min	Area<br>mAU*min	Height<br>mAU	Relative Area<br>%	Relative Height<br>%	Amount<br>n.a.
1		21,830	7,808	14,643	0,93	1,22	n.a.
2		22,840	829,259	1188,070	99,07	98,78	n.a.
**Total:**			**837,067**	**1202,713**	**100,00**	**100,00**	

Chromatogram

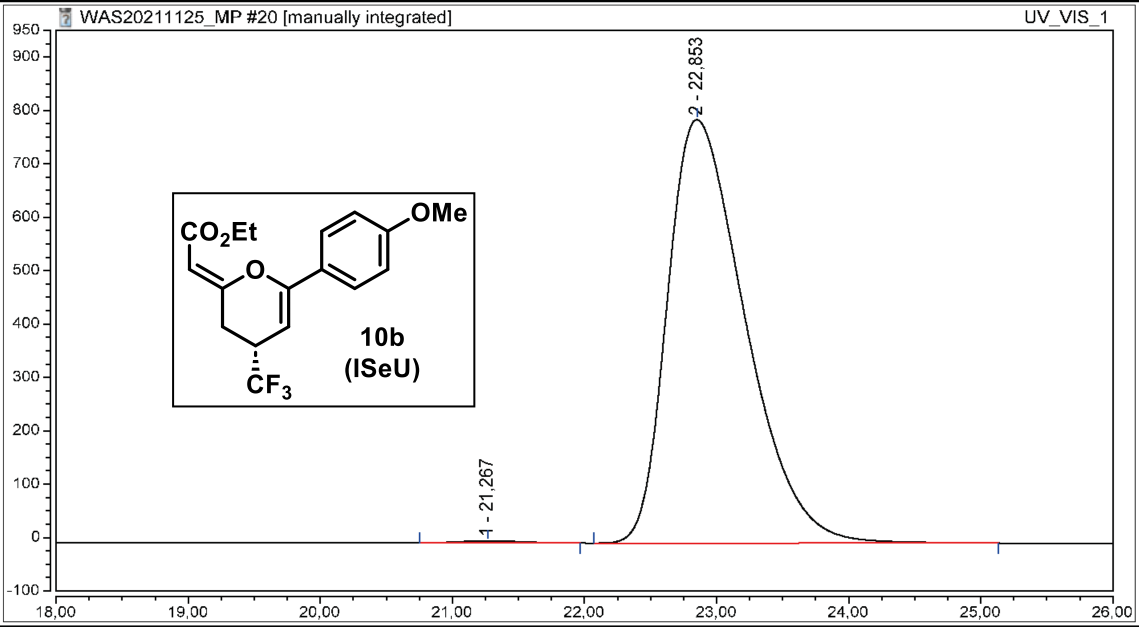

Integration Results

No.	Peak Name	Retention Time min	Area mAU*min	Height mAU	Relative Area %	Relative Height %	Amount n.a.
1		21,267	2,203	3,937	0,41	0,49	n.a.
2		22,853	540,144	793,494	99,59	99,51	n.a.
**Total:**			**542,348**	**797,431**	**100,00**	**100,00**	

Chromatogram

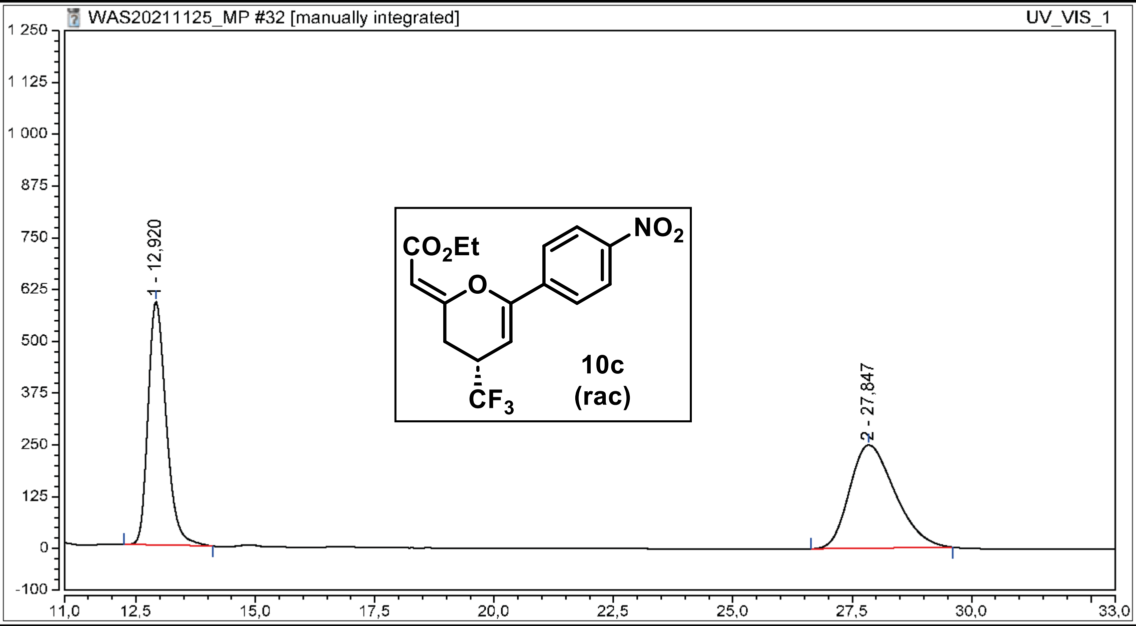

Integration Results

No.	Peak Name	Retention Time min	Area mAU*min	Height mAU	Relative Area %	Relative Height %	Amount n.a.
1		12,920	257,016	587,958	48,42	70,24	n.a.
2		27,847	273,747	249,152	51,58	29,76	n.a.
**Total:**			**530,763**	**837,110**	**100,00**	**100,00**	

Chromatogram

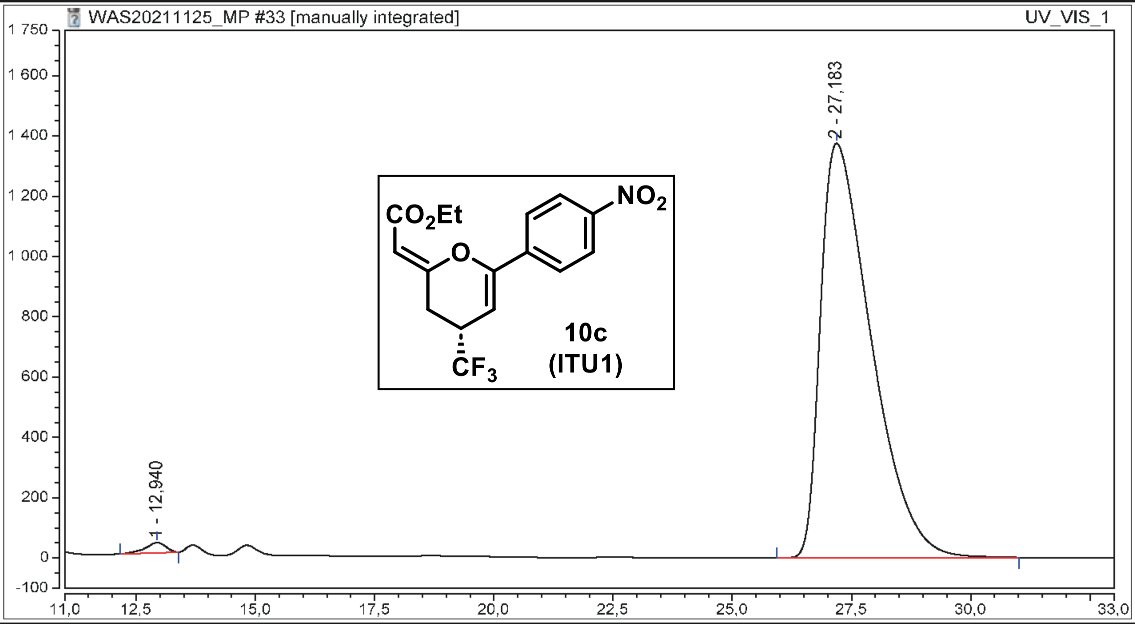

Integration Results

No.	Peak Name	Retention Time min	Area mAU*min	Height mAU	Relative Area %	Relative Height %	Amount n.a.
1		12,940	16,824	33,701	0,98	2,39	n.a.
2		27,183	1696,504	1375,641	99,02	97,61	n.a.
**Total:**			**1713,329**	**1409,342**	**100,00**	**100,00**	

Chromatogram

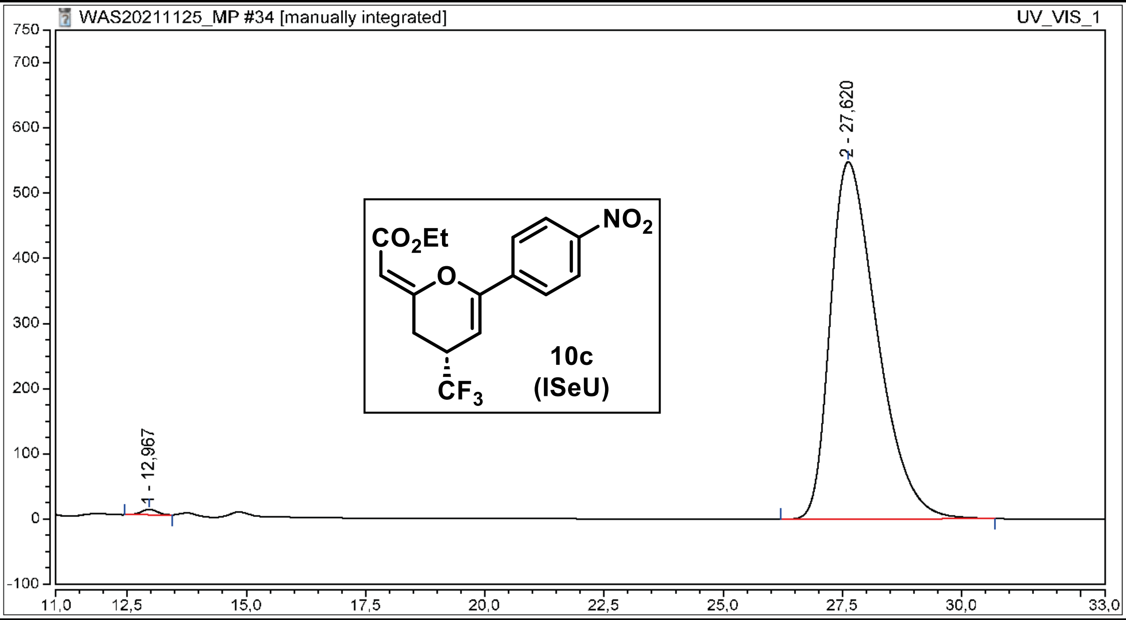

Integration Results

No.	Peak Name	Retention Time min	Area mAU*min	Height mAU	Relative Area %	Relative Height %	Amount n.a.
1		12,967	3,203	8,167	0,51	1,47	n.a.
2		27,620	624,721	547,869	99,49	98,53	n.a.
**Total:**			**627,924**	**556,036**	**100,00**	**100,00**	

Chromatogram

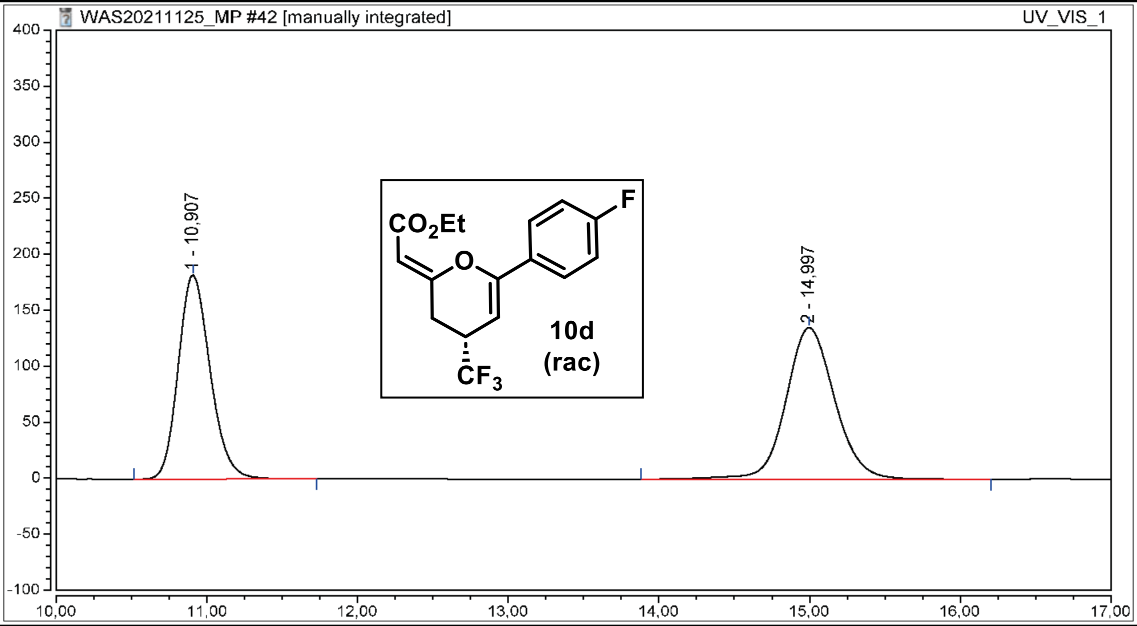

Integration Results

No.	Peak Name	Retention Time min	Area mAU*min	Height mAU	Relative Area %	Relative Height %	Amount n.a.
1		10,907	44,727	182,135	46,85	57,37	n.a.
2		14,997	50,735	135,359	53,15	42,63	n.a.
**Total:**			**95,462**	**317,494**	**100,00**	**100,00**	

Chromatogram

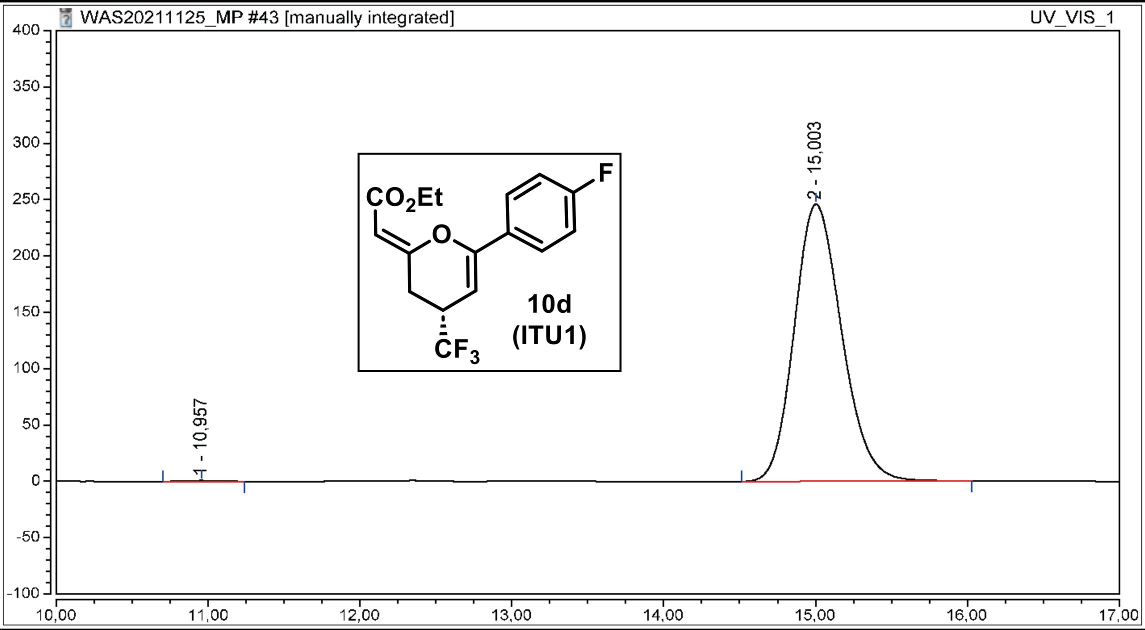

Integration Results

No.	Peak Name	Retention Time min	Area mAU*min	Height mAU	Relative Area %	Relative Height %	Amount n.a.
1		10,957	0,189	0,775	0,21	0,31	n.a.
2		15,003	89,300	246,216	99,79	99,69	n.a.
**Total:**			**89,489**	**246,991**	**100,00**	**100,00**	

Chromatogram

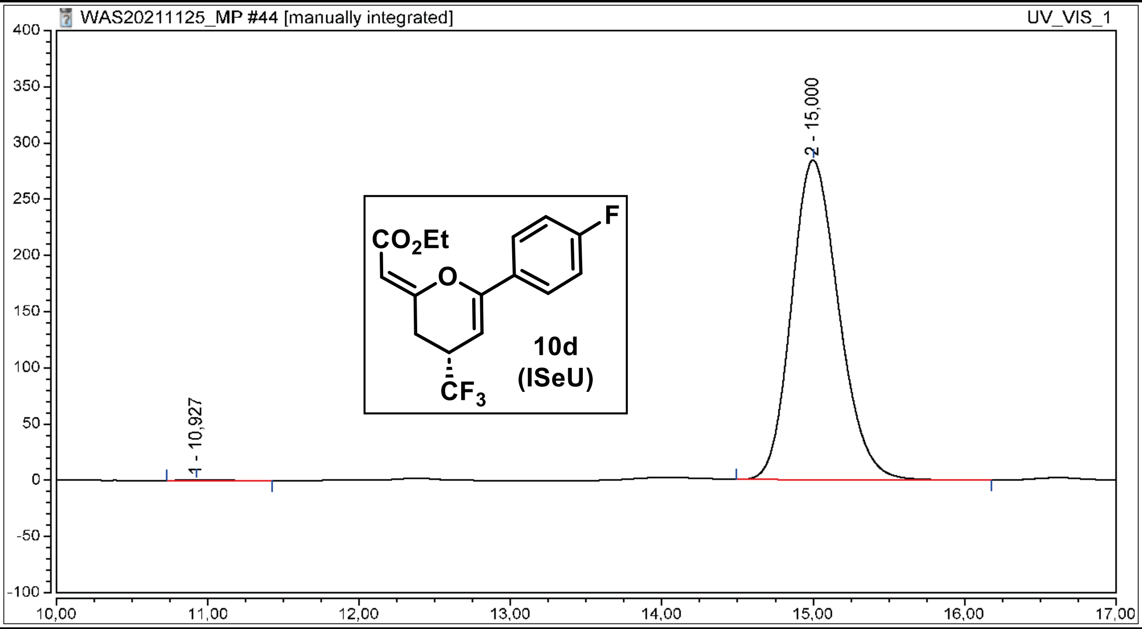

Integration Results

No.	Peak Name	Retention Time min	Area mAU*min	Height mAU	Relative Area %	Relative Height %	Amount n.a.
1		10,927	0,145	0,572	0,14	0,20	n.a.
2		15,000	102,615	284,279	99,86	99,80	n.a.
**Total:**			**102,760**	**284,852**	**100,00**	**100,00**	

Chromatogram

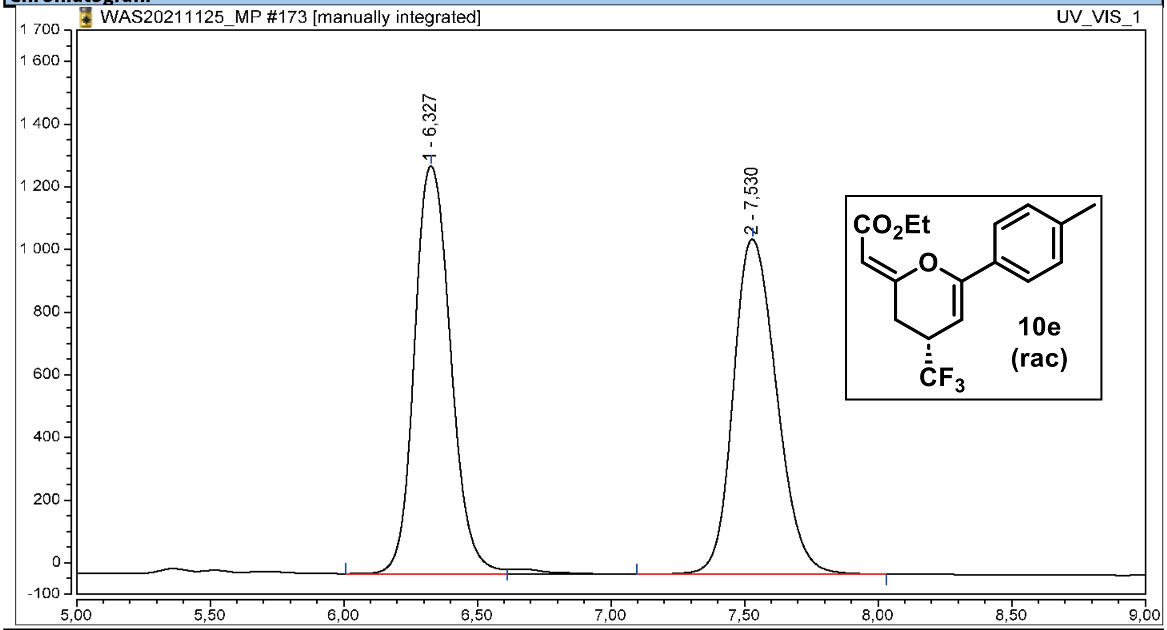

Integration Results

No.	Peak Name	Retention Time min	Area mAU*min	Height mAU	Relative Area %	Relative Height %	Amount n.a.
1		6,327	204,279	1301,987	50,41	54,89	n.a.
2		7,530	200,956	1069,891	49,59	45,11	n.a.
**Total:**			**405,235**	**2371,878**	**100,00**	**100,00**	

Chromatogram

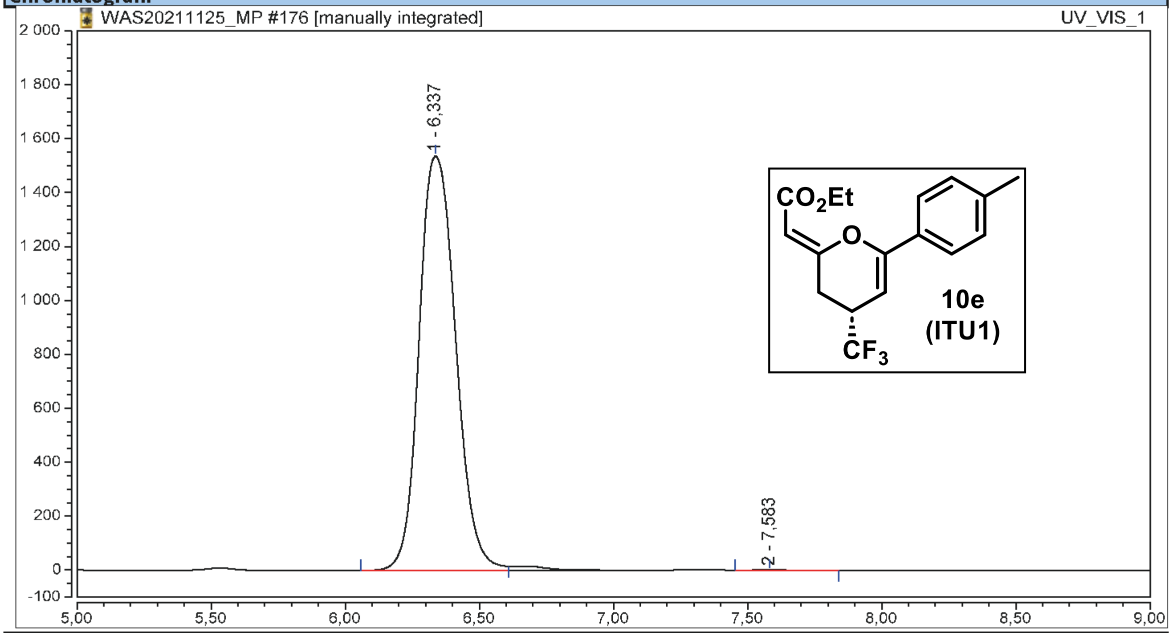

Integration Results

No.	Peak Name	Retention Time min	Area mAU*min	Height mAU	Relative Area %	Relative Height %	Amount n.a.
1		6,337	243,115	1537,216	99,92	99,92	n.a.
2		7,583	0,199	1,230	0,08	0,08	n.a.
**Total:**			**243,314**	**1538,446**	**100,00**	**100,00**	

Chromatogram

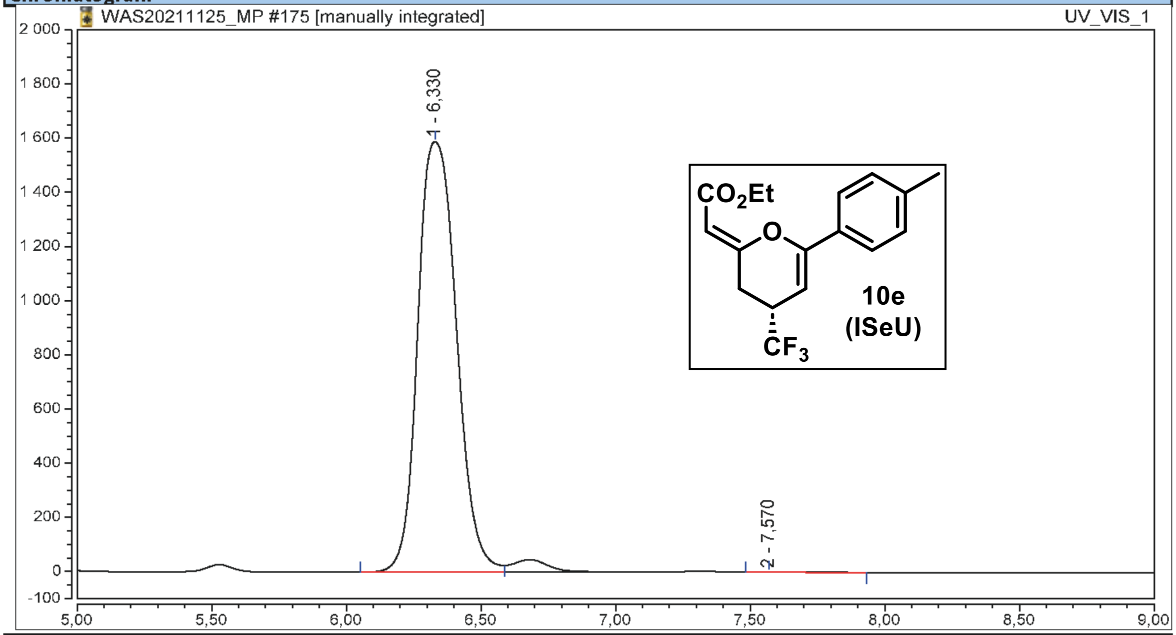

Integration Results

No.	Peak Name	Retention Time min	Area mAU*min	Height mAU	Relative Area %	Relative Height %	Amount n.a.
1		6,330	264,311	1589,185	99,96	99,97	n.a.
2		7,570	0,105	0,519	0,04	0,03	n.a.
**Total:**			**264,416**	**1589,704**	**100,00**	**100,00**	

Chromatogram

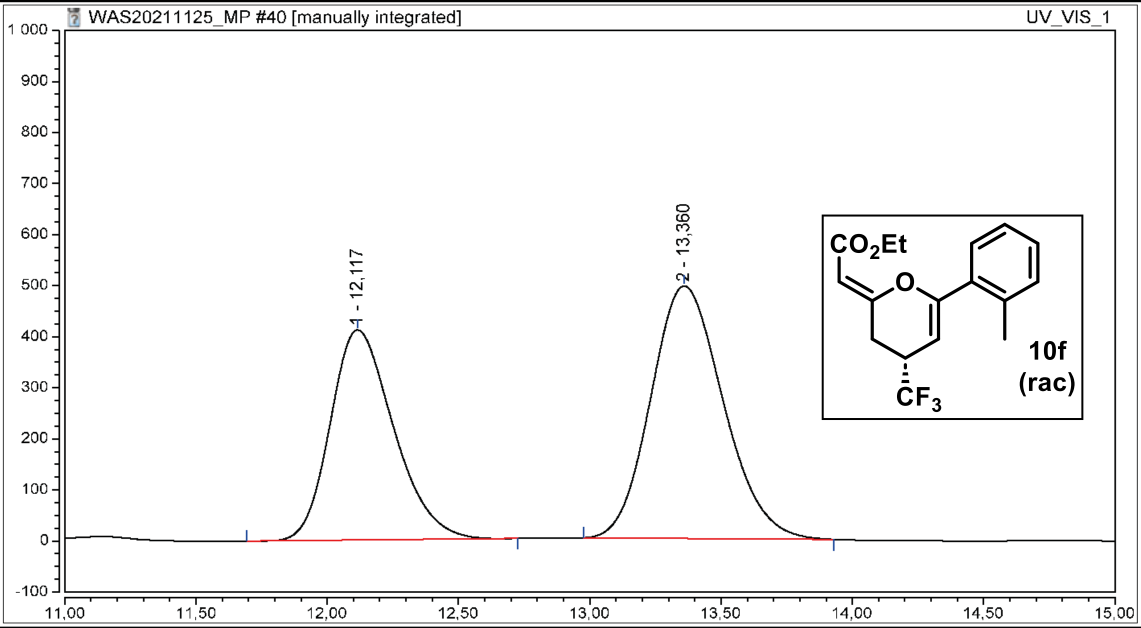

Integration Results

No.	Peak Name	Retention Time min	Area mAU*min	Height mAU	Relative Area %	Relative Height %	Amount n.a.
1		12,117	114,837	411,361	42,08	45,40	n.a.
2		13,360	158,076	494,700	57,92	54,60	n.a.
**Total:**			**272,913**	**906,062**	**100,00**	**100,00**	

Chromatogram

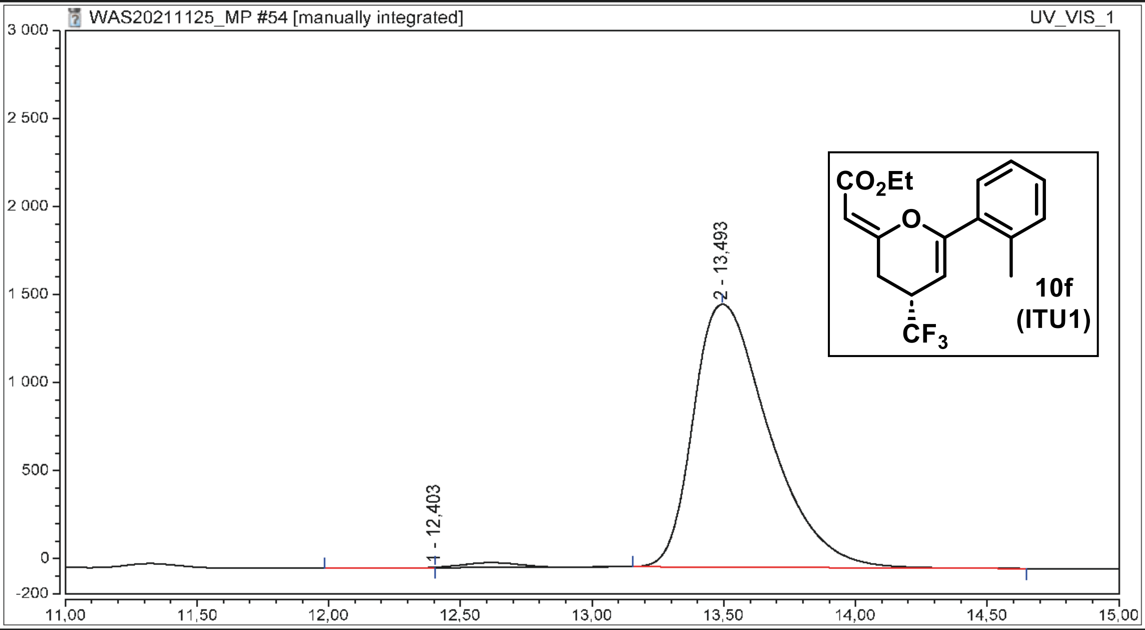

Integration Results

No.	Peak Name	Retention Time min	Area mAU*min	Height mAU	Relative Area %	Relative Height %	Amount n.a.
1		12,403	0,101	2,681	0,02	0,18	n.a.
2		13,493	497,576	1492,675	99,98	99,82	n.a.
**Total:**			**497,676**	**1495,356**	**100,00**	**100,00**	

Chromatogram

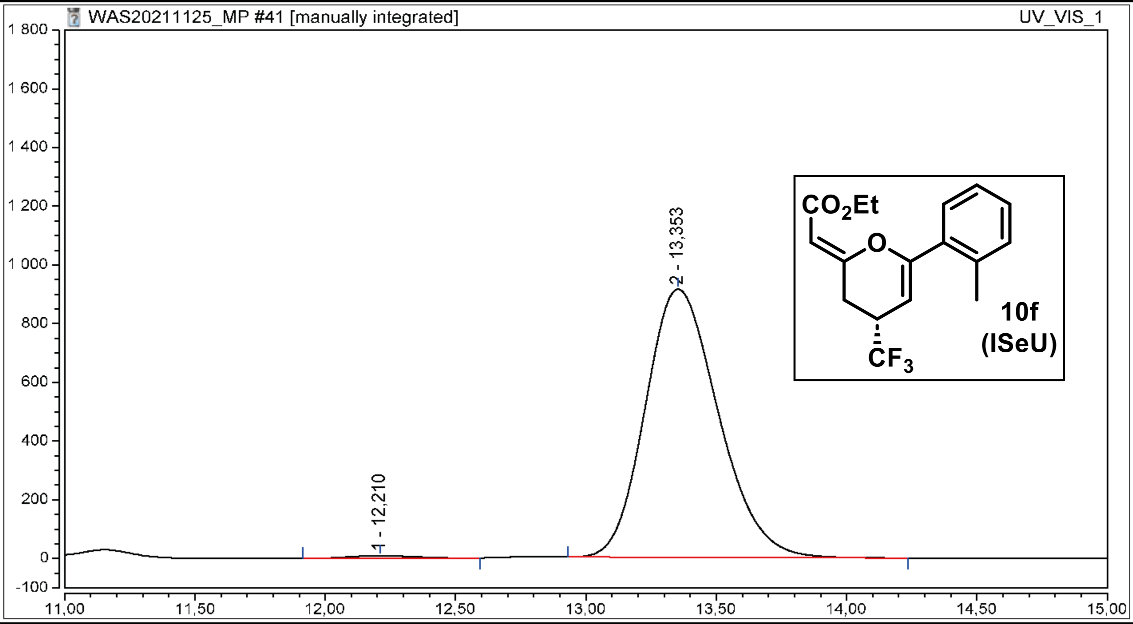

Integration Results

No.	Peak Name	Retention Time min	Area mAU*min	Height mAU	Relative Area %	Relative Height %	Amount n.a.
1		12,210	2,328	8,627	0,78	0,94	n.a.
2		13,353	296,645	913,808	99,22	99,06	n.a.
**Total:**			**298,973**	**922,435**	**100,00**	**100,00**	

Chromatogram

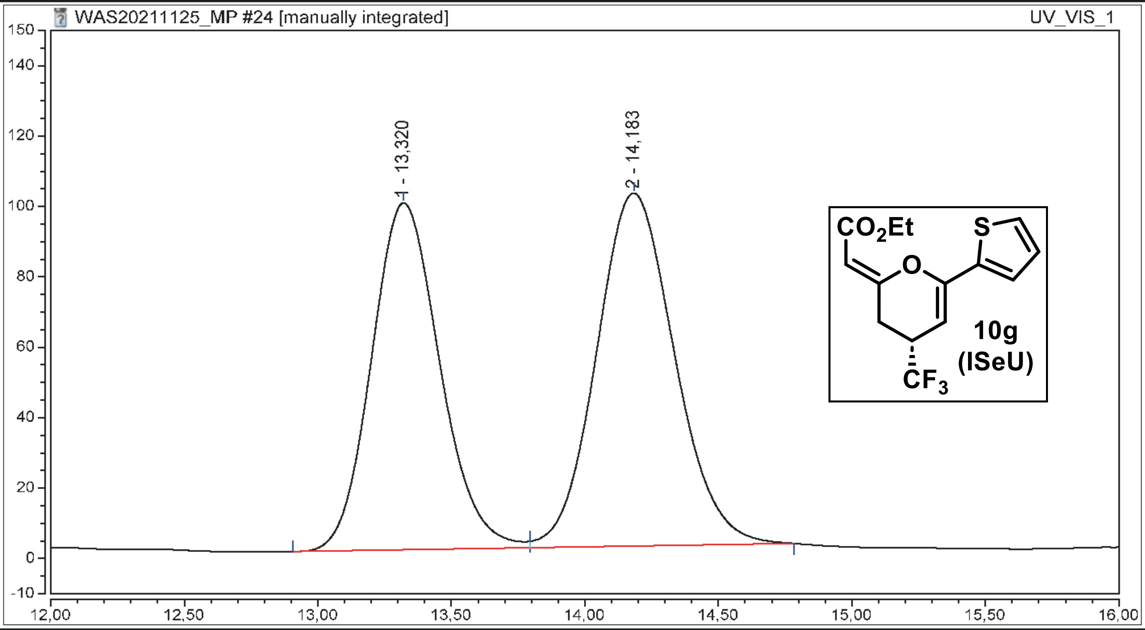

Integration Results

No.	Peak Name	Retention Time min	Area mAU*min	Height mAU	Relative Area %	Relative Height %	Amount n.a.
1		13,320	29,151	98,514	46,24	49,56	n.a.
2		14,183	33,885	100,261	53,76	50,44	n.a.
**Total:**			**63,036**	**198,775**	**100,00**	**100,00**	

Chromatogram

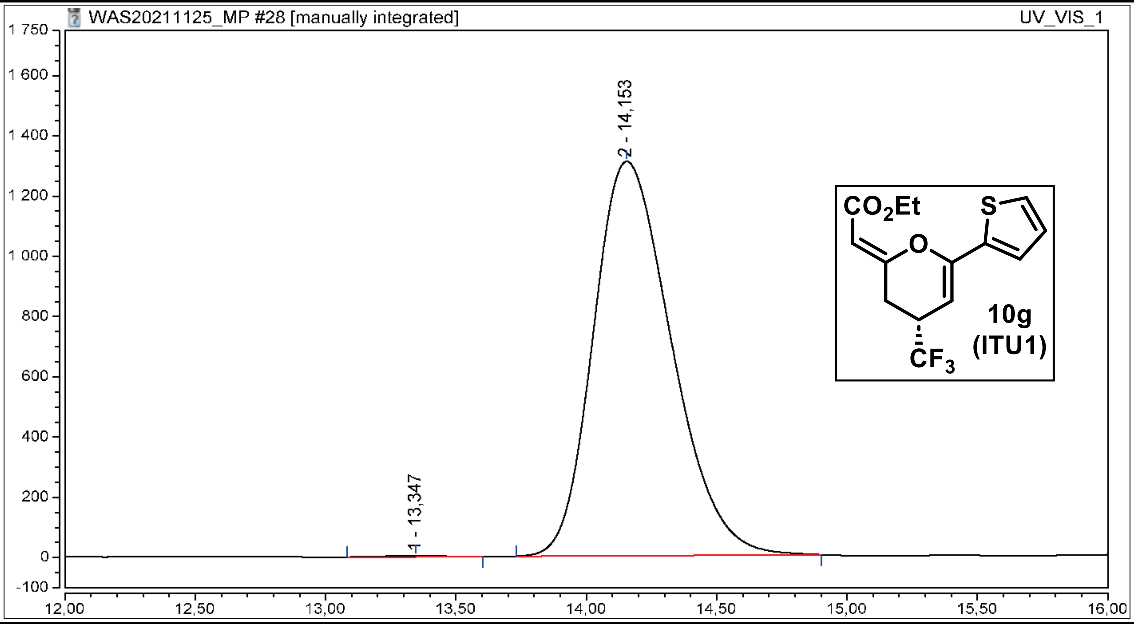

Integration Results

No.	Peak Name	Retention Time min	Area mAU*min	Height mAU	Relative Area %	Relative Height %	Amount n.a.
1		13,347	1,067	4,321	0,23	0,33	n.a.
2		14,153	454,576	1308,881	99,77	99,67	n.a.
**Total:**			**455,643**	**1313,202**	**100,00**	**100,00**	

Chromatogram

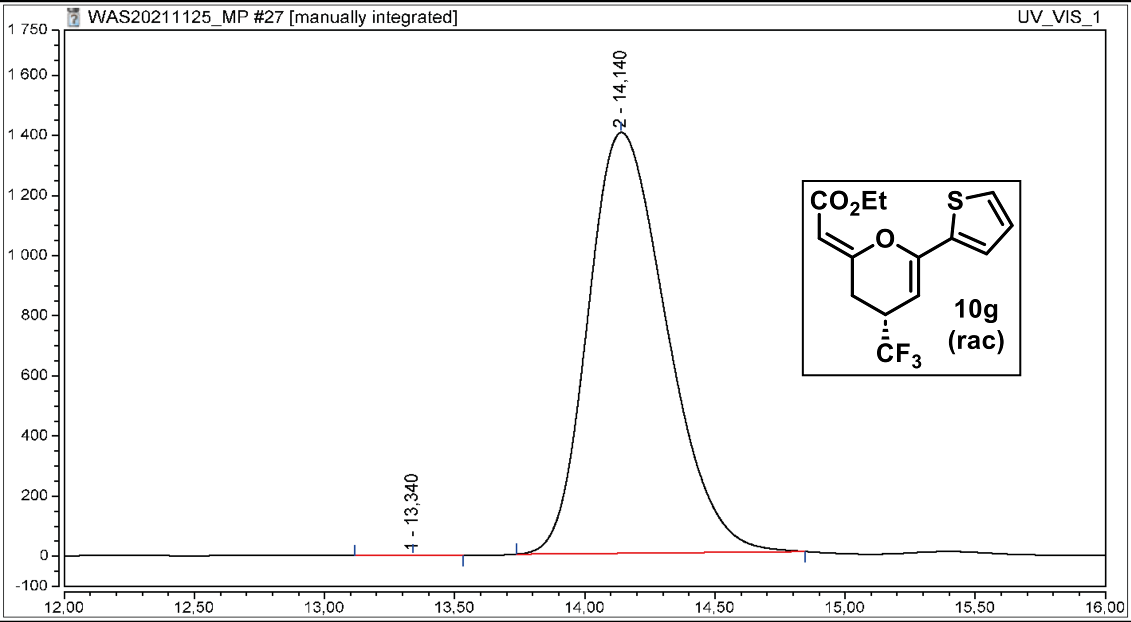

Integration Results

No.	Peak Name	Retention Time min	Area mAU*min	Height mAU	Relative Area %	Relative Height %	Amount n.a.
1		13,340	0,145	0,766	0,03	0,05	n.a.
2		14,140	489,202	1400,004	99,97	99,95	n.a.
**Total:**			**489,346**	**1400,770**	**100,00**	**100,00**	

Chromatogram

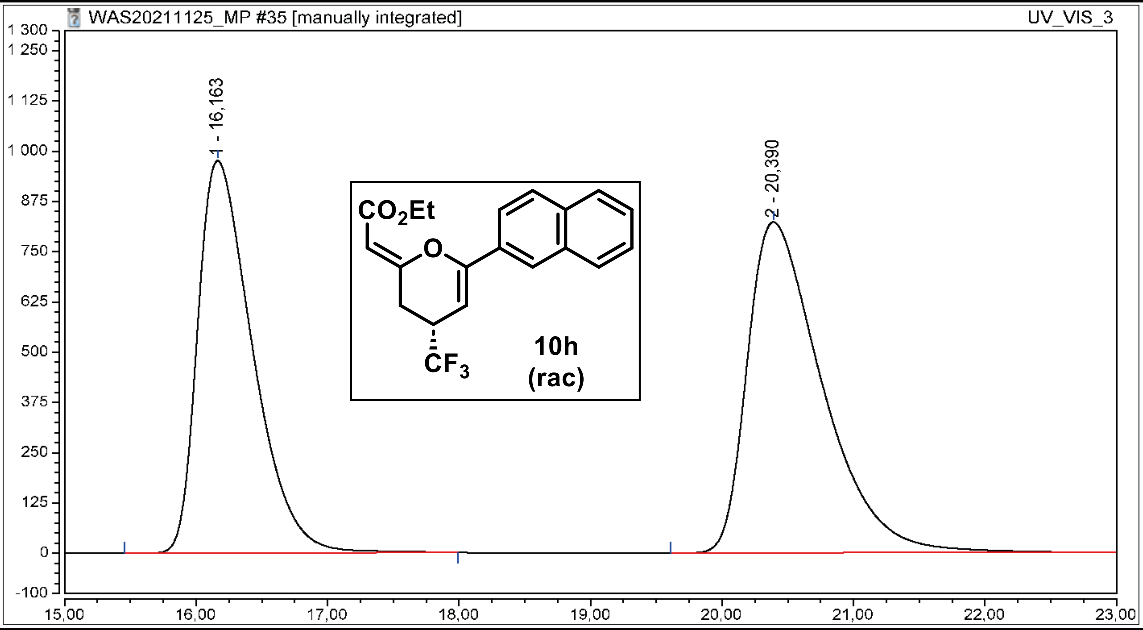

Integration Results

No.	Peak Name	Retention Time min	Area mAU*min	Height mAU	Relative Area %	Relative Height %	Amount n.a.
1		16,163	471,324	976,372	46,45	54,25	n.a.
2		20,390	543,335	823,252	53,55	45,75	n.a.
**Total:**			**1014,659**	**1799,623**	**100,00**	**100,00**	

Chromatogram

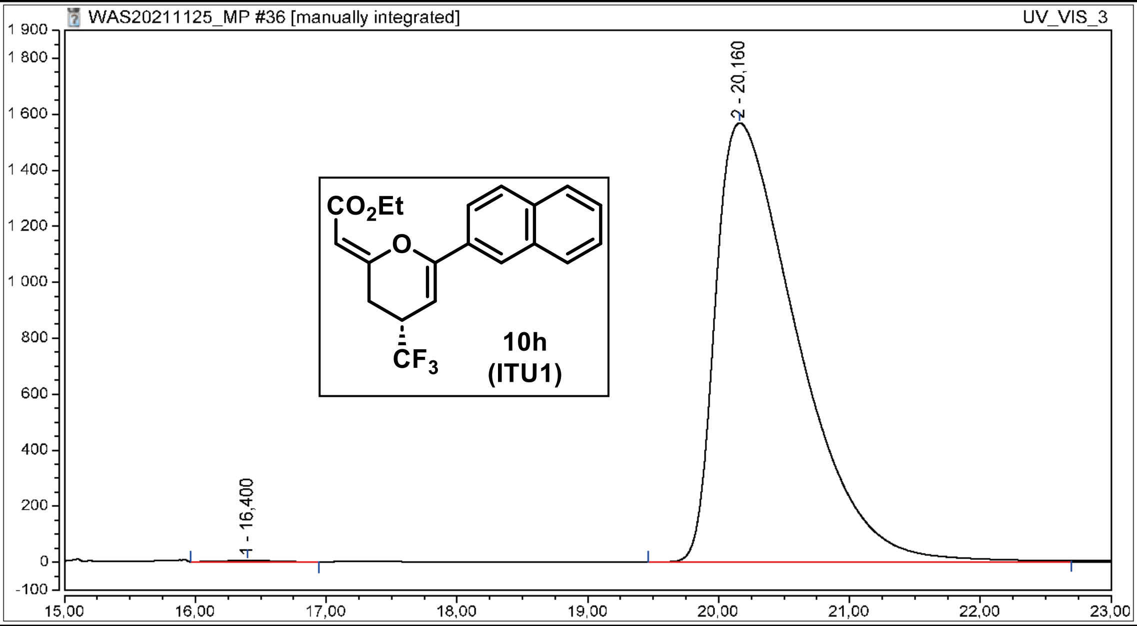

Integration Results

No.	Peak Name	Retention Time min	Area mAU*min	Height mAU	Relative Area %	Relative Height %	Amount n.a.
1		16,400	2,107	5,247	0,19	0,33	n.a.
2		20,160	1134,117	1568,191	99,81	99,67	n.a.
**Total:**			**1136,224**	**1573,438**	**100,00**	**100,00**	

Chromatogram

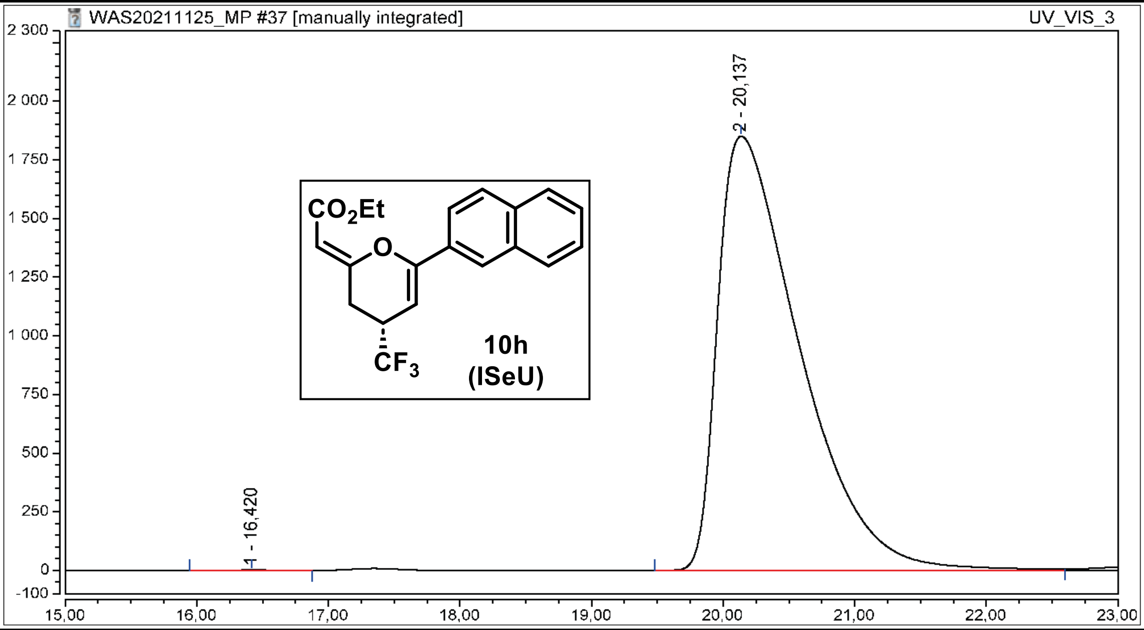

Integration Results

No.	Peak Name	Retention Time min	Area mAU*min	Height mAU	Relative Area %	Relative Height %	Amount n.a.
1		16,420	0,736	1,762	0,06	0,10	n.a.
2		20,137	1333,979	1850,743	99,94	99,90	n.a.
**Total:**			**1334,715**	**1852,505**	**100,00**	**100,00**	

Chromatogram

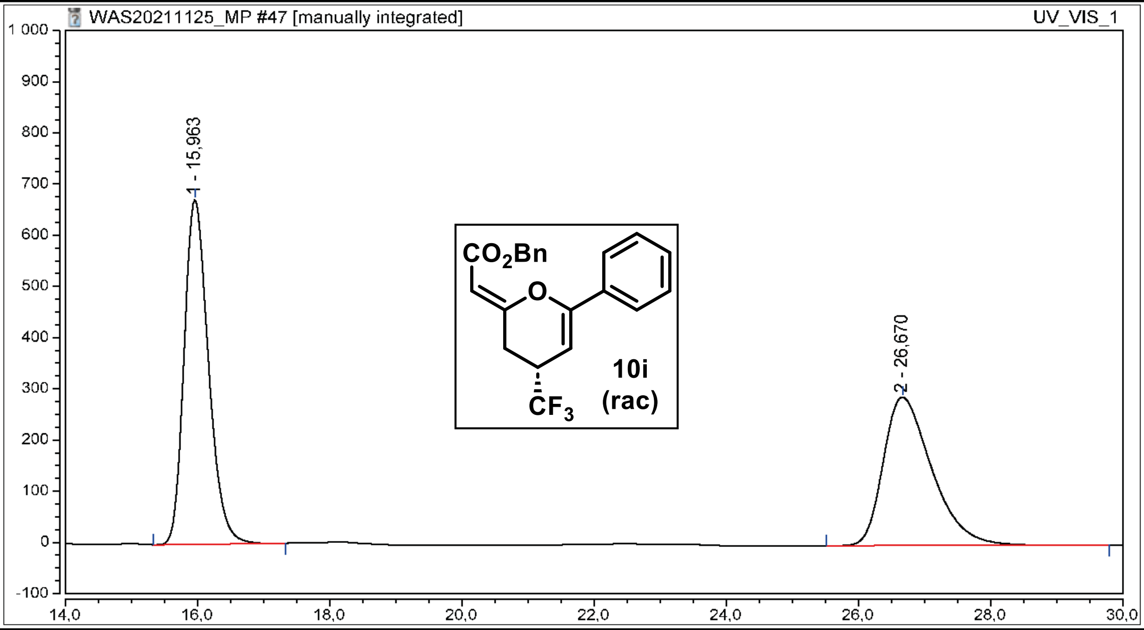

Integration Results

No.	Peak Name	Retention Time<br>min	Area<br>mAU*min	Height<br>mAU	Relative Area<br>%	Relative Height<br>%	Amount<br>n.a.
1		15,963	286,777	673,040	53,87	69,89	n.a.
2		26,670	245,591	289,934	46,13	30,11	n.a.
**Total:**			**532,368**	**962,974**	**100,00**	**100,00**	

Chromatogram

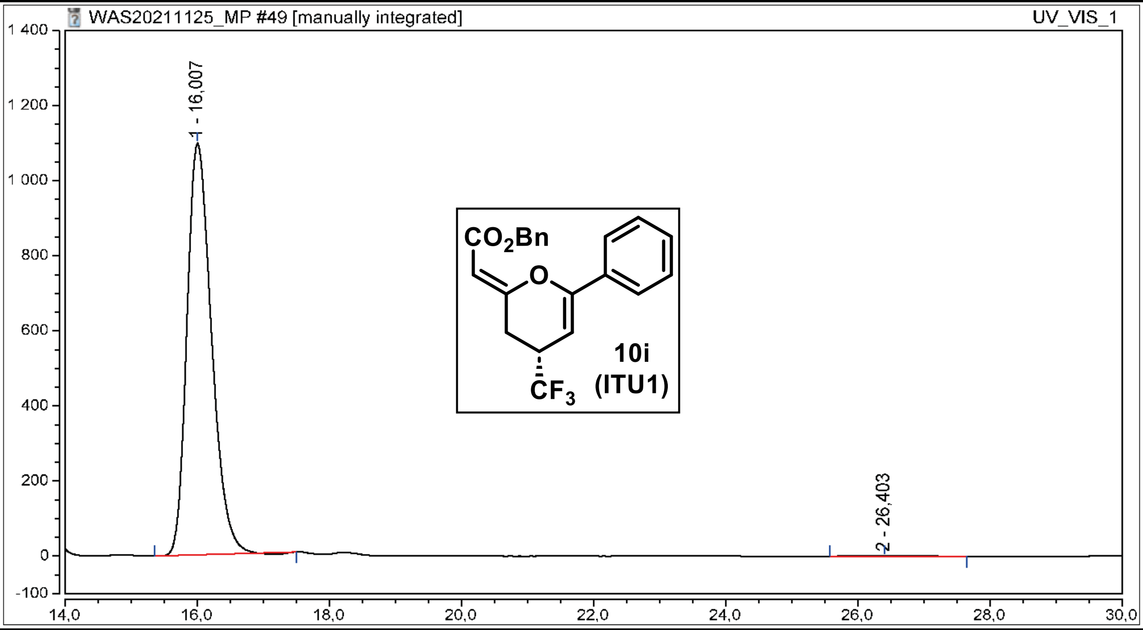

Integration Results

No.	Peak Name	Retention Time<br>min	Area<br>mAU*min	Height<br>mAU	Relative Area<br>%	Relative Height<br>%	Amount<br>n.a.
1		16,007	470,641	1096,786	99,79	99,92	n.a.
2		26,403	0,972	0,851	0,21	0,08	n.a.
**Total:**			**471,613**	**1097,636**	**100,00**	**100,00**	

Chromatogram

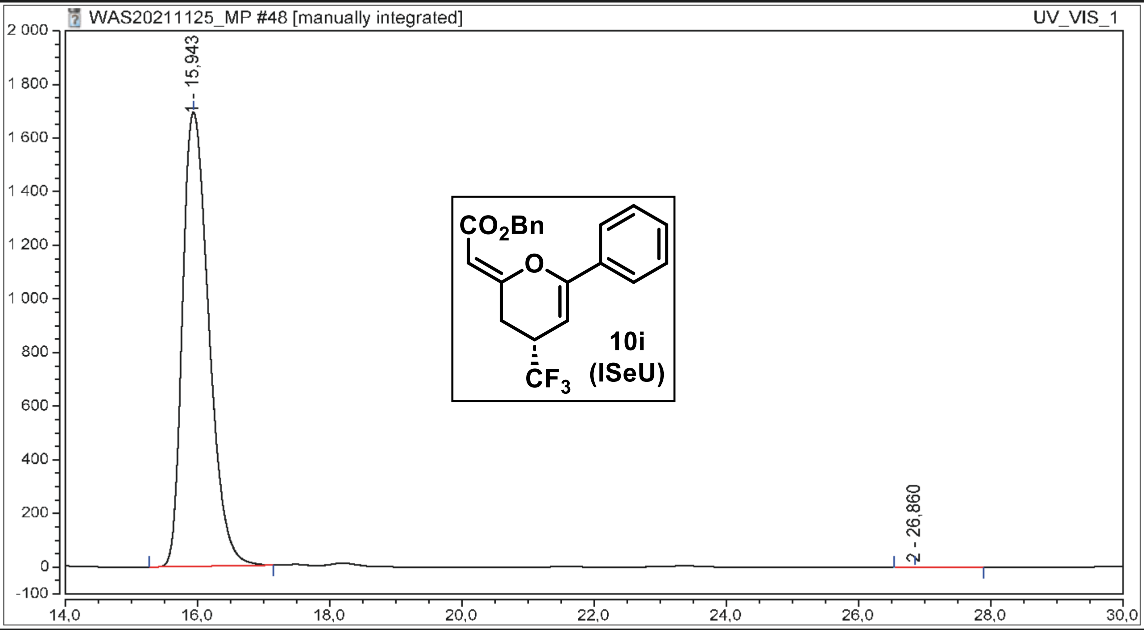

Integration Results

No.	Peak Name	Retention Time min	Area mAU*min	Height mAU	Relative Area %	Relative Height %	Amount n.a.
1		15,943	780,303	1695,545	99,89	99,93	n.a.
2		26,860	0,864	1,154	0,11	0,07	n.a.
**Total:**			**781,167**	**1696,698**	**100,00**	**100,00**	

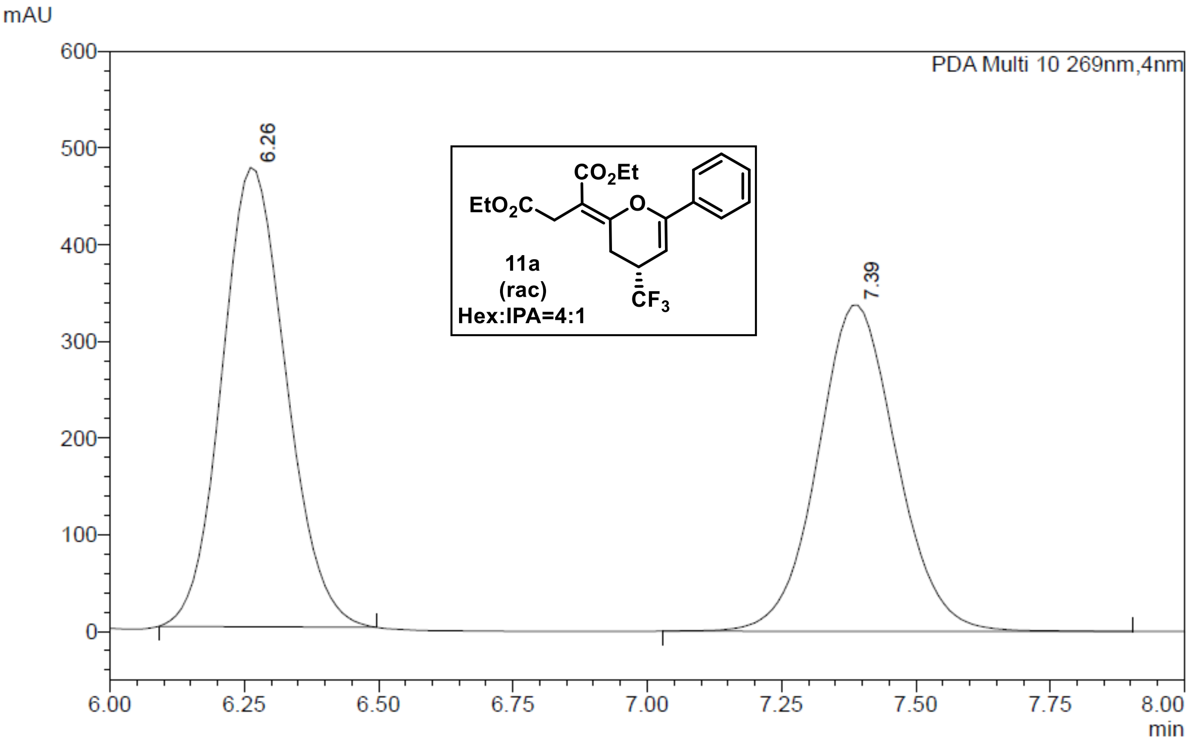

Peak Table

PDA Ch10 269nm

Peak#	Ret. Time	Area	Area%
1	6.26	3964415	53.37
2	7.39	3463342	46.63
Total		7427758	100.00

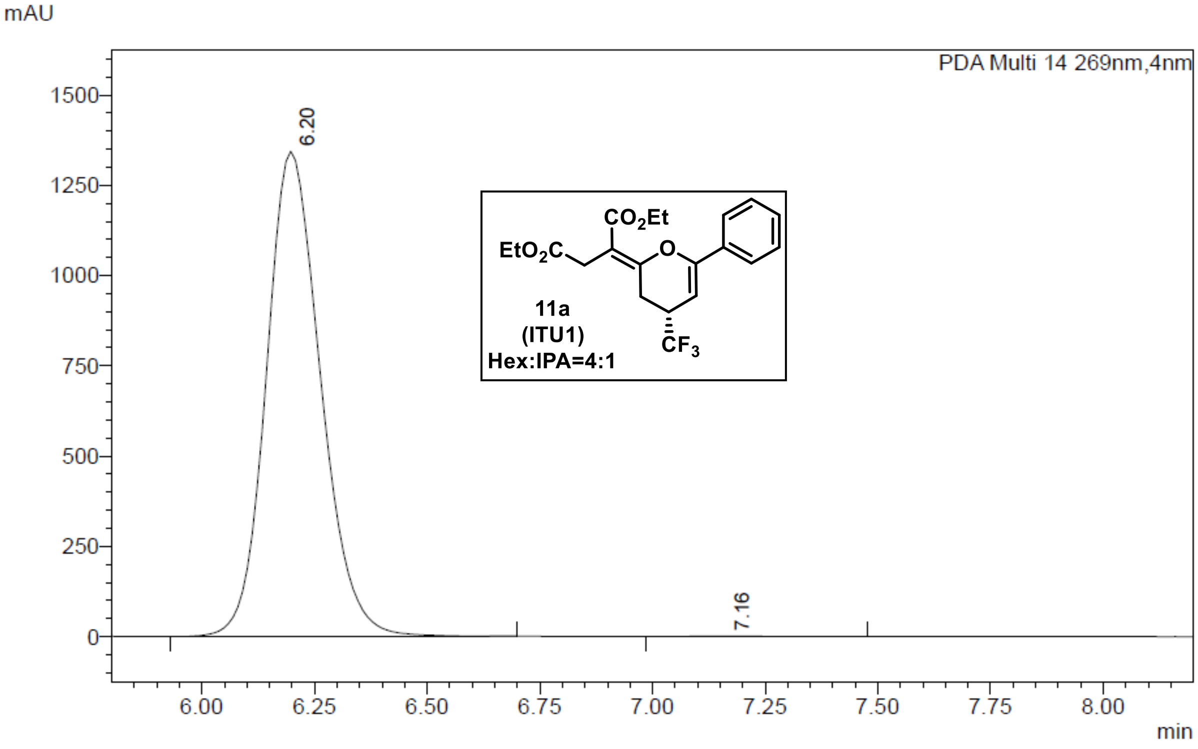

Peak Table

PDA Ch14 269nm

Peak#	Ret. Time	Area	Area%
1	6.20	11251854	99.90
2	7.16	11651	0.10
Total		11263506	100.00

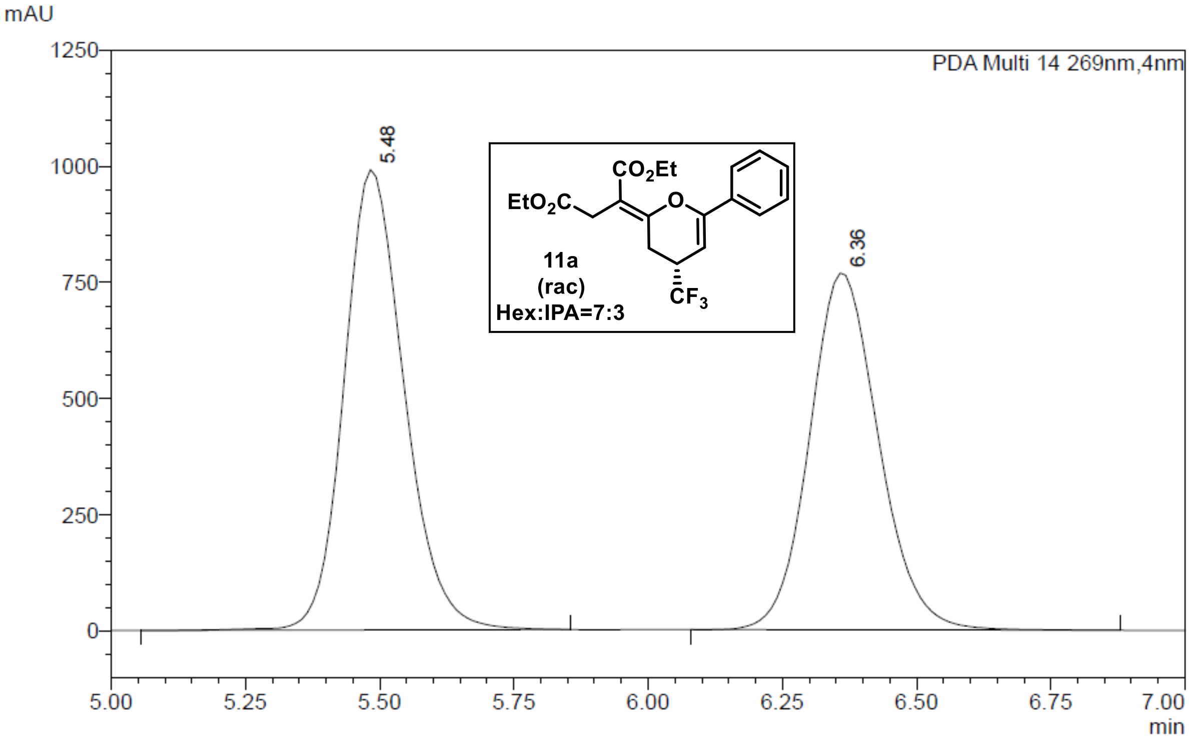

Peak Table

PDA Ch14 269nm

Peak#	Ret. Time	Area	Area%
1	5.48	7716116	52.82
2	6.36	6892454	47.18
Total		14608571	100.00

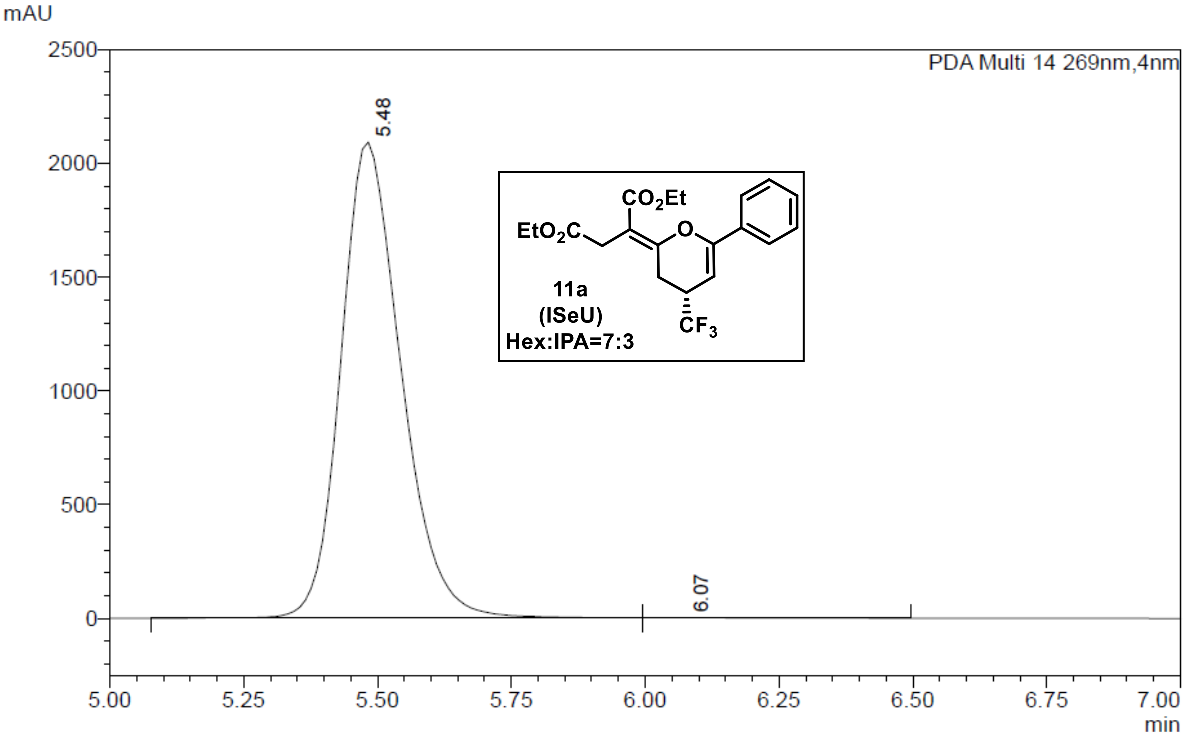

Peak Table

PDA Ch14 269nm

Peak#	Ret. Time	Area	Area%
1	5.48	16437095	99.67
2	6.07	54841	0.33
Total		16491936	100.00

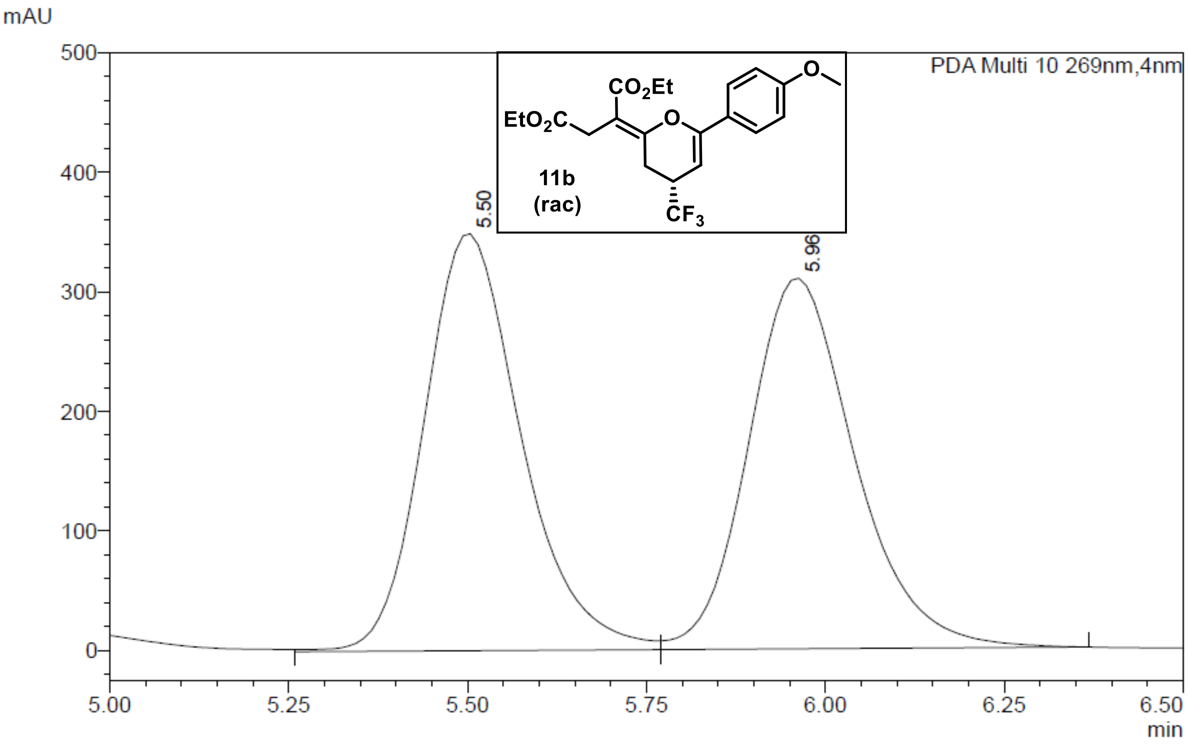

Peak Table

PDA Ch10 269nm

Peak#	Ret. Time	Area	Area%
1	5.50	3306717	50.74
2	5.96	3210343	49.26
Total		6517060	100.00

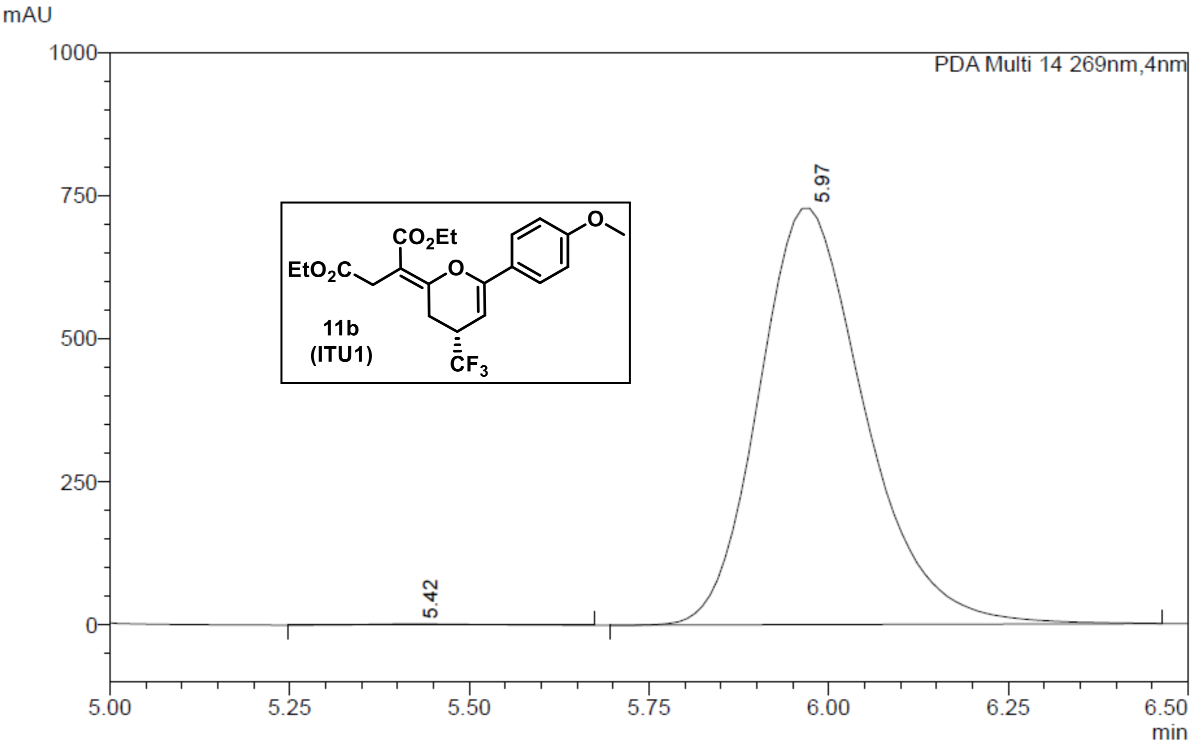

Peak Table

PDA Ch14 269nm

Peak#	Ret. Time	Area	Area%
1	5.42	19059	0.25
2	5.97	7462967	99.75
Total		7482026	100.00

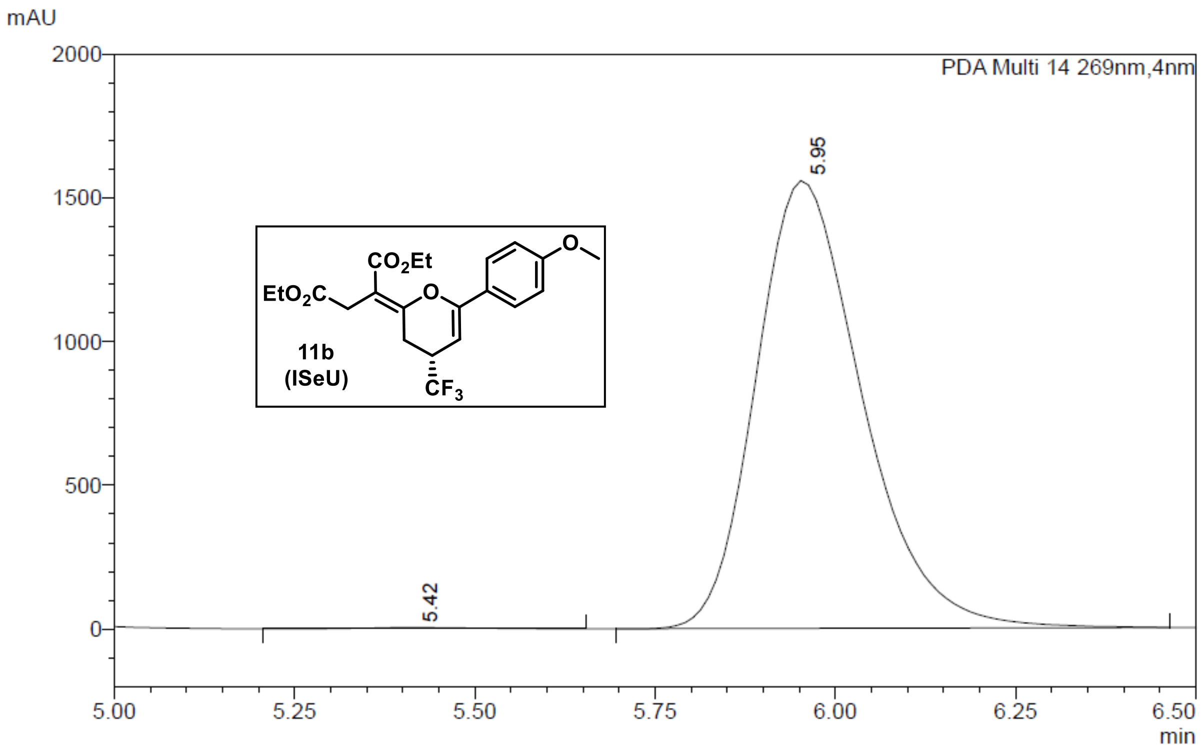

Peak Table

PDA Ch14 269nm

Peak#	Ret. Time	Area	Area%
1	5.42	46671	0.29
2	5.95	15986558	99.71
Total		16033229	100.00

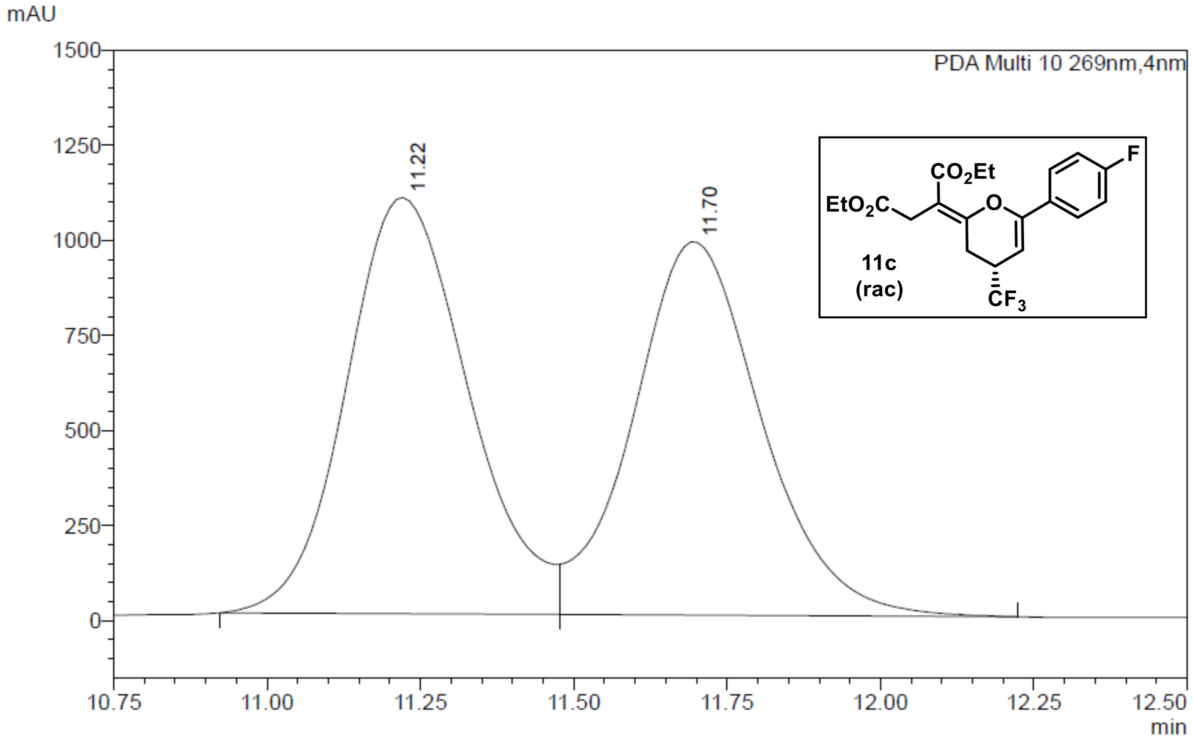

Peak Table

PDA Ch10 269nm

Peak#	Ret. Time	Area	Area%
1	11.22	15187069	51.51
2	11.70	14297243	48.49
Total		29484311	100.00

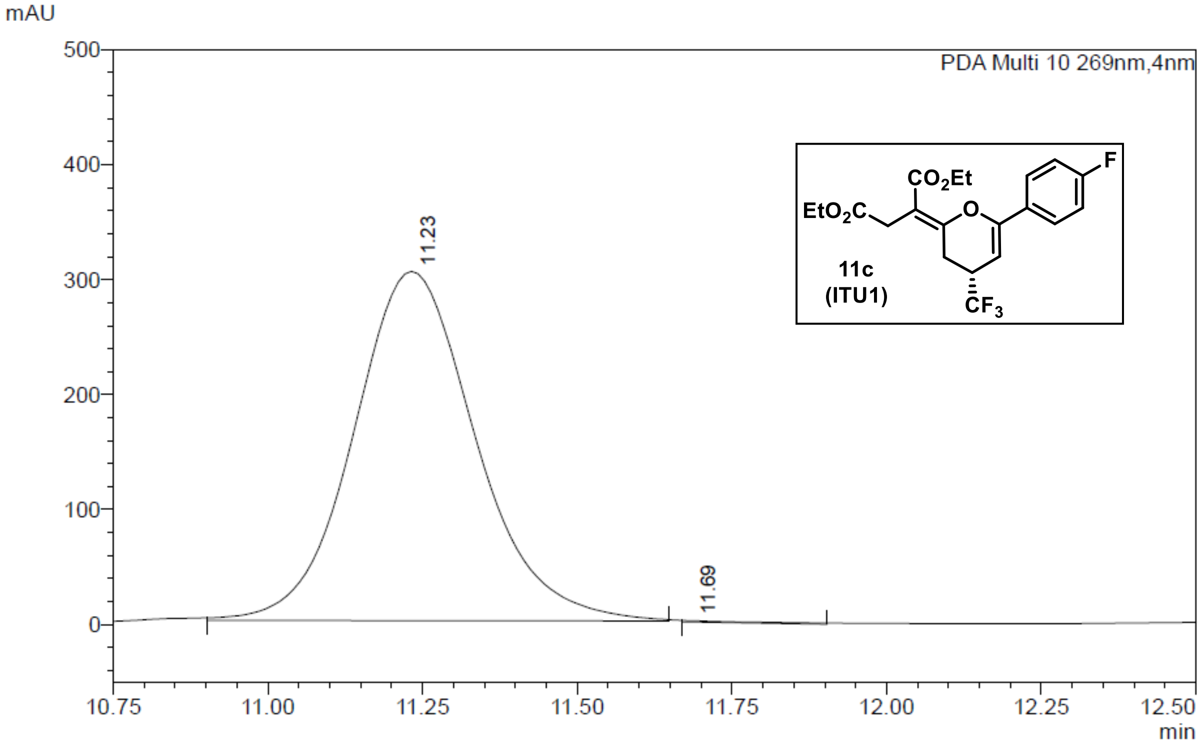

Peak Table

PDA Ch10 269nm

Peak#	Ret. Time	Area	Area%
1	11.23	4165093	99.72
2	11.69	11803	0.28
Total		4176896	100.00

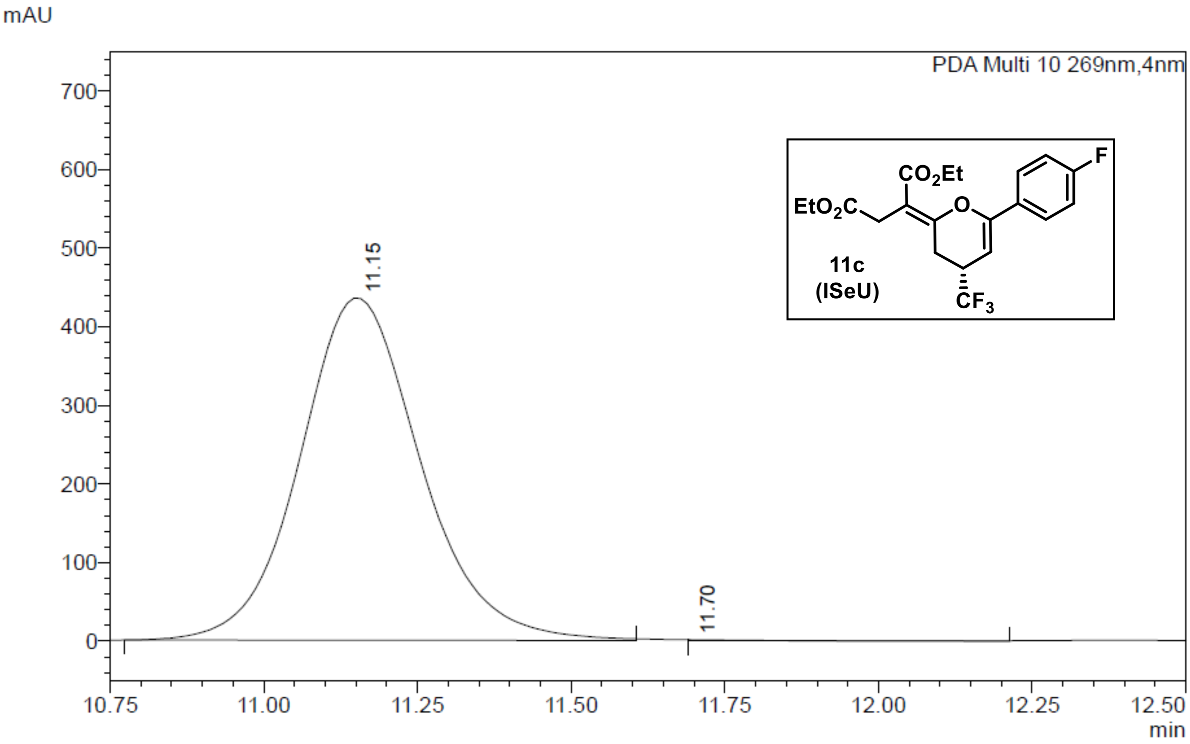

Peak Table

PDA Ch10 269nm

Peak#	Ret. Time	Area	Area%
1	11.15	5958702	99.86
2	11.70	8644	0.14
Total		5967346	100.00

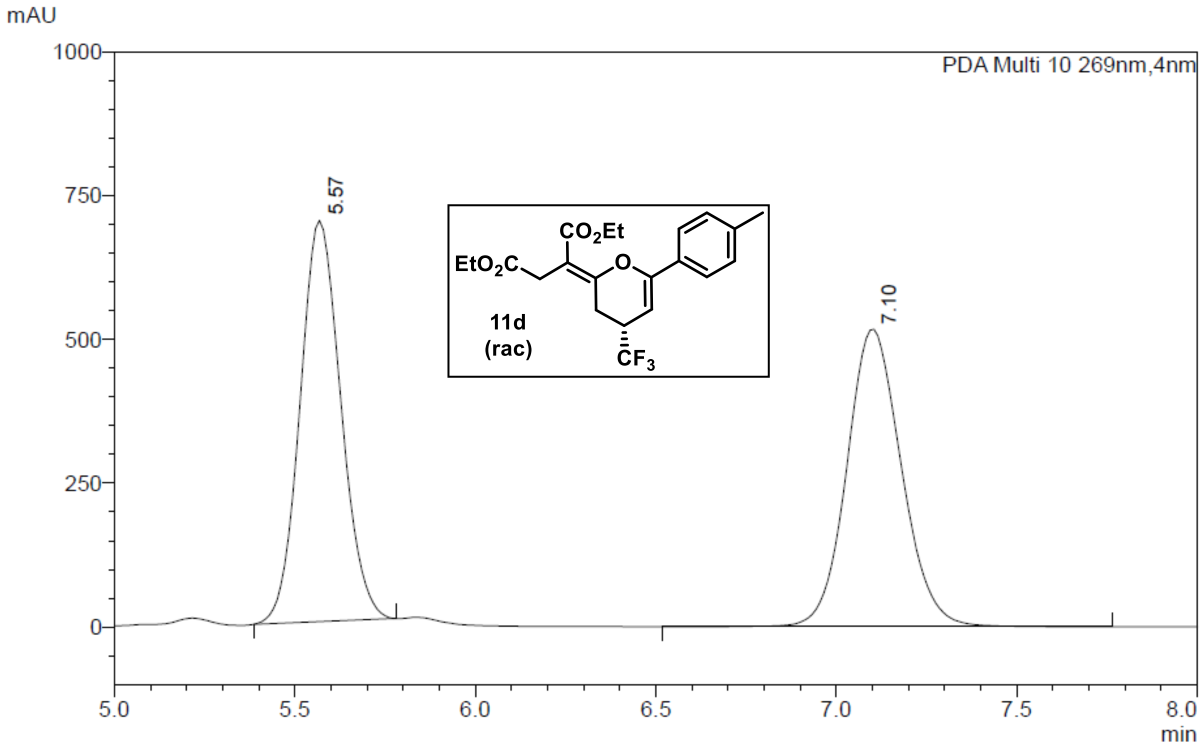

Peak Table

PDA Ch10 269nm

Peak#	Ret. Time	Area	Area%
1	5.57	5487568	50.17
2	7.10	5449769	49.83
Total		10937336	100.00

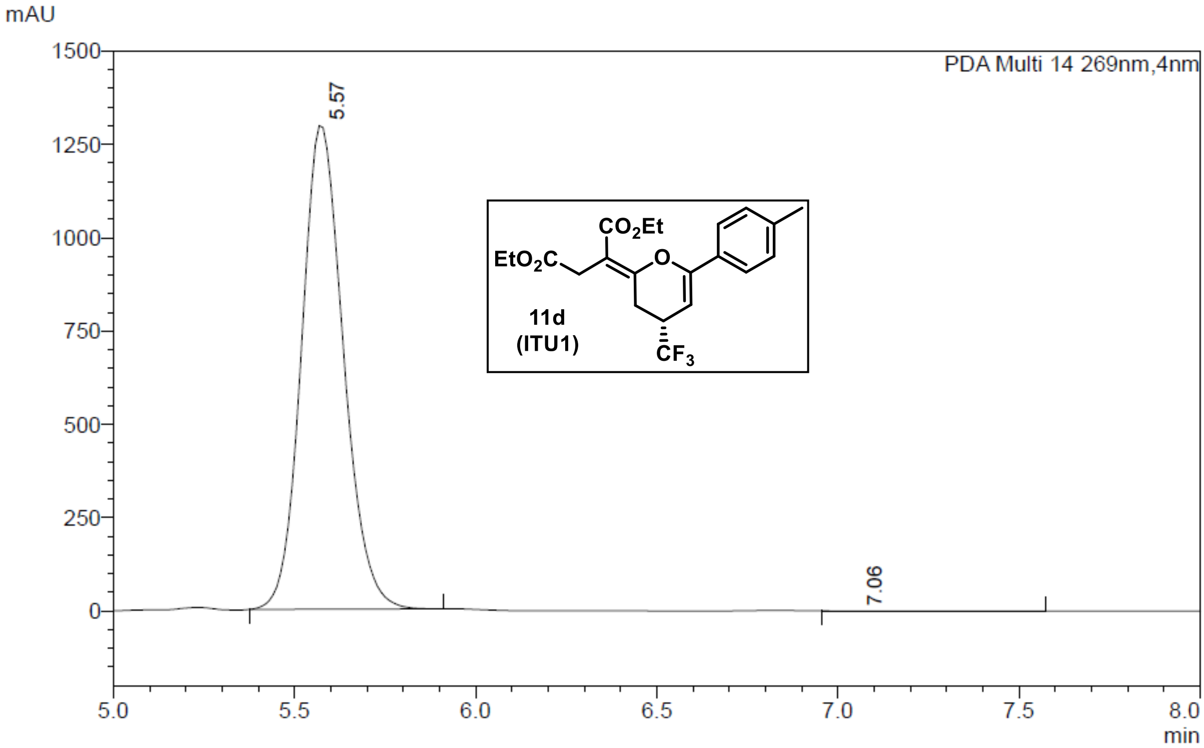

Peak Table

PDA Ch14 269nm

Peak#	Ret. Time	Area	Area%
1	5.57	10443315	99.93
2	7.06	6850	0.07
Total		10450164	100.00

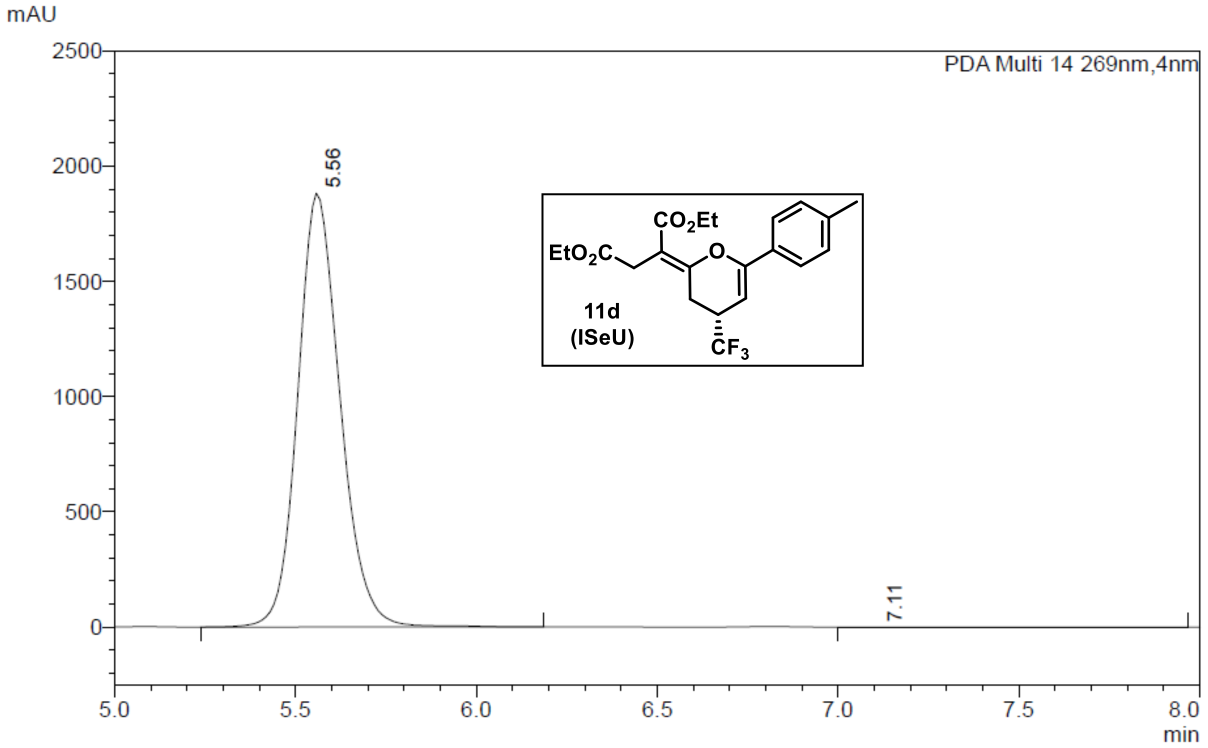

Peak Table

PDA Ch14 269nm

Peak#	Ret. Time	Area	Area%
1	5.56	15305783	99.95
2	7.11	7580	0.05
Total		15313363	100.00

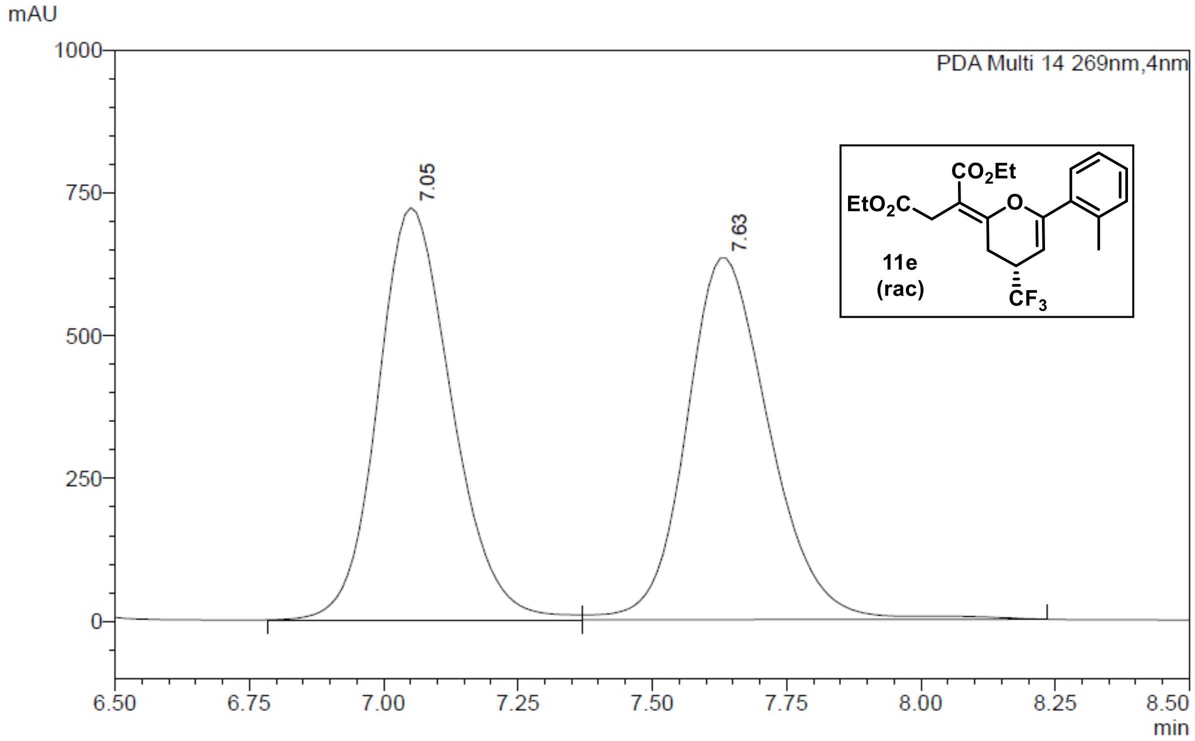

Peak Table

PDA Ch14 269nm

Peak#	Ret. Time	Area	Area%
1	7.05	6929428	50.29
2	7.63	6849719	49.71
Total		13779147	100.00

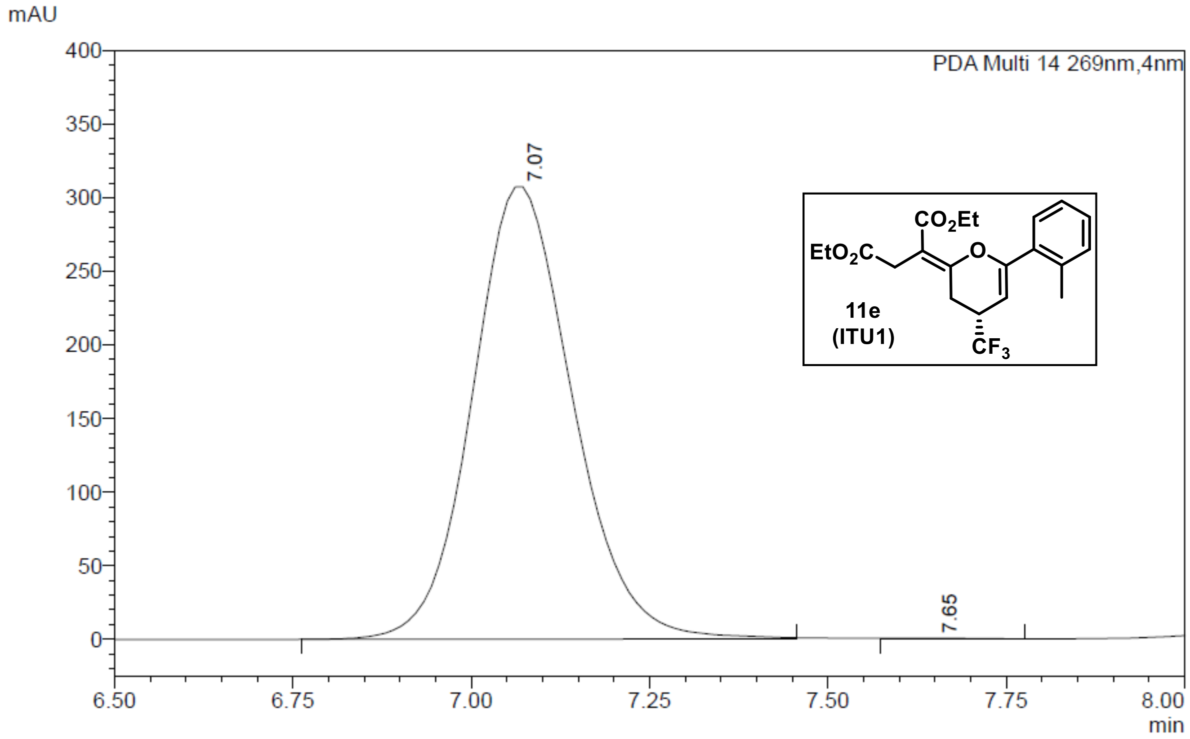

Peak Table

PDA Ch14 269nm

Peak#	Ret. Time	Area	Area%
1	7.07	3014876	99.90
2	7.65	3165	0.10
Total		3018041	100.00

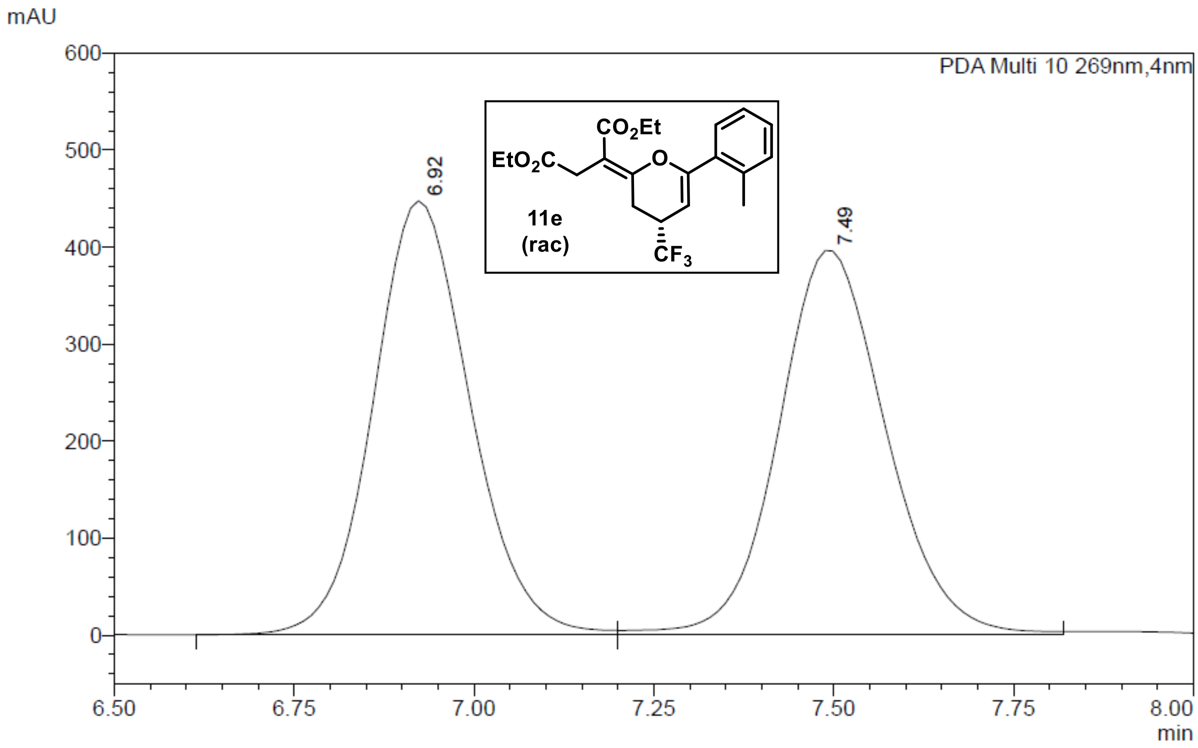

Peak Table

PDA Ch10 269nm

Peak#	Ret. Time	Area	Area%
1	6.92	4205926	50.41
2	7.49	4137130	49.59
Total		8343056	100.00

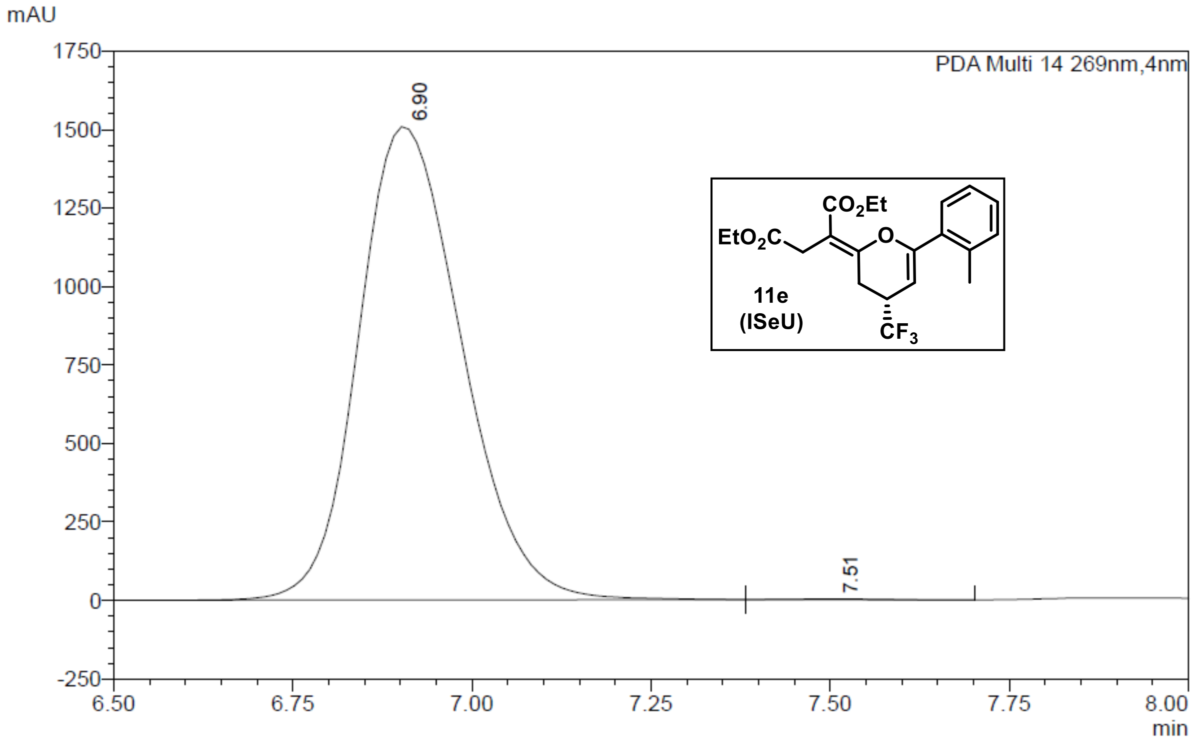

Peak Table

PDA Ch14 269nm

Peak#	Ret. Time	Area	Area%
1	6.90	15113724	99.89
2	7.51	17078	0.11
Total		15130803	100.00

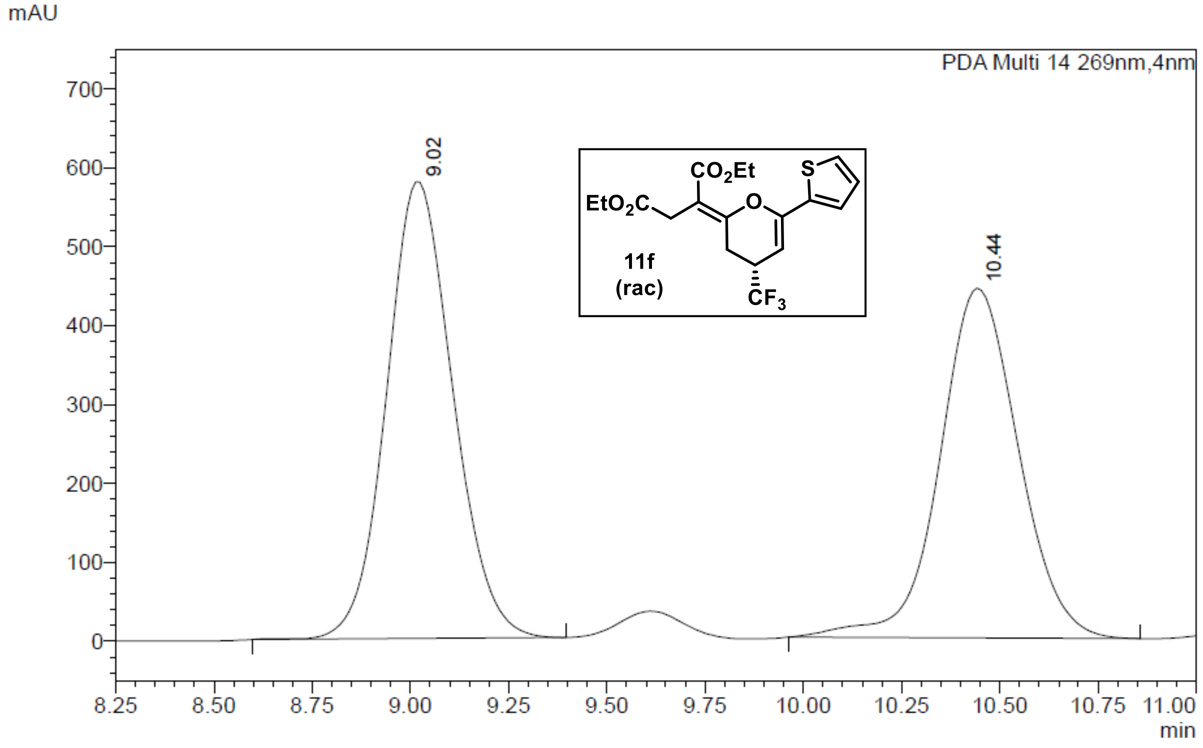

Peak Table

PDA Ch14 269nm

Peak#	Ret. Time	Area	Area%
1	9.02	6811952	52.44
2	10.44	6178360	47.56
Total		12990312	100.00

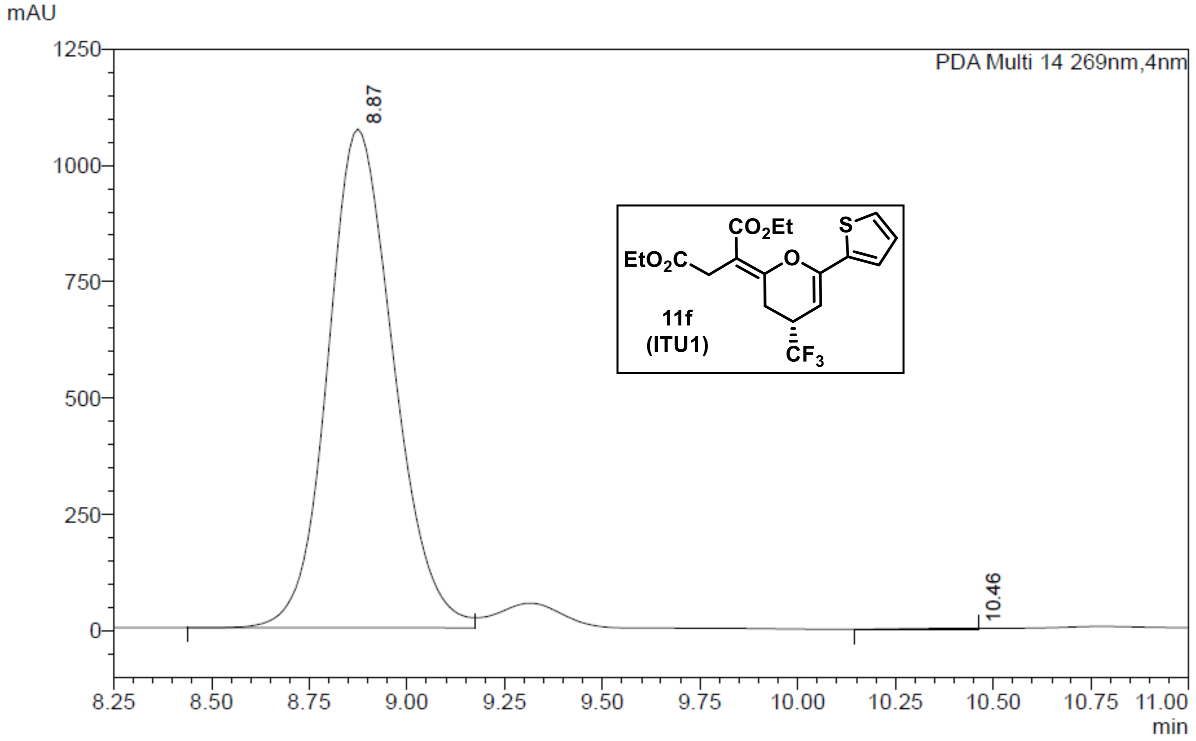

Peak Table

PDA Ch14 269nm

Peak#	Ret. Time	Area	Area%
1	8.87	12539317	99.80
2	10.46	25414	0.20
Total		12564731	100.00

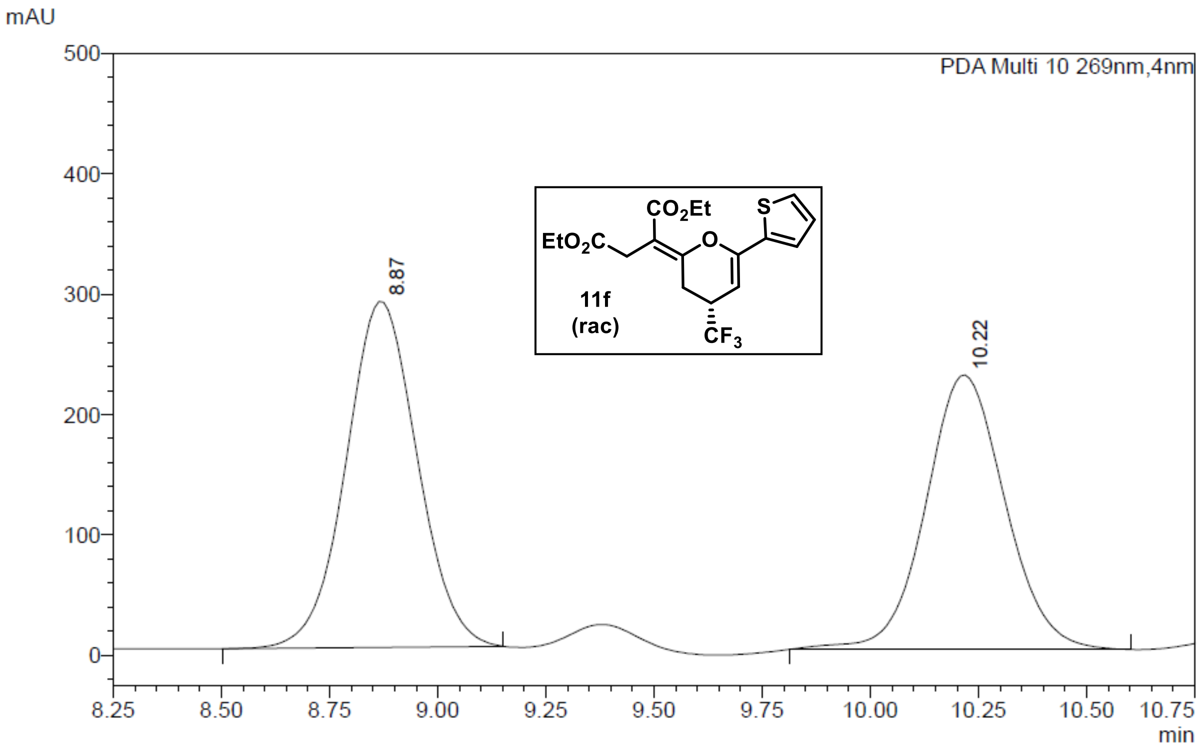

Peak Table

PDA Ch10 269nm

Peak#	Ret. Time	Area	Area%
1	8.87	3295074	53.10
2	10.22	2910889	46.90
Total		6205963	100.00

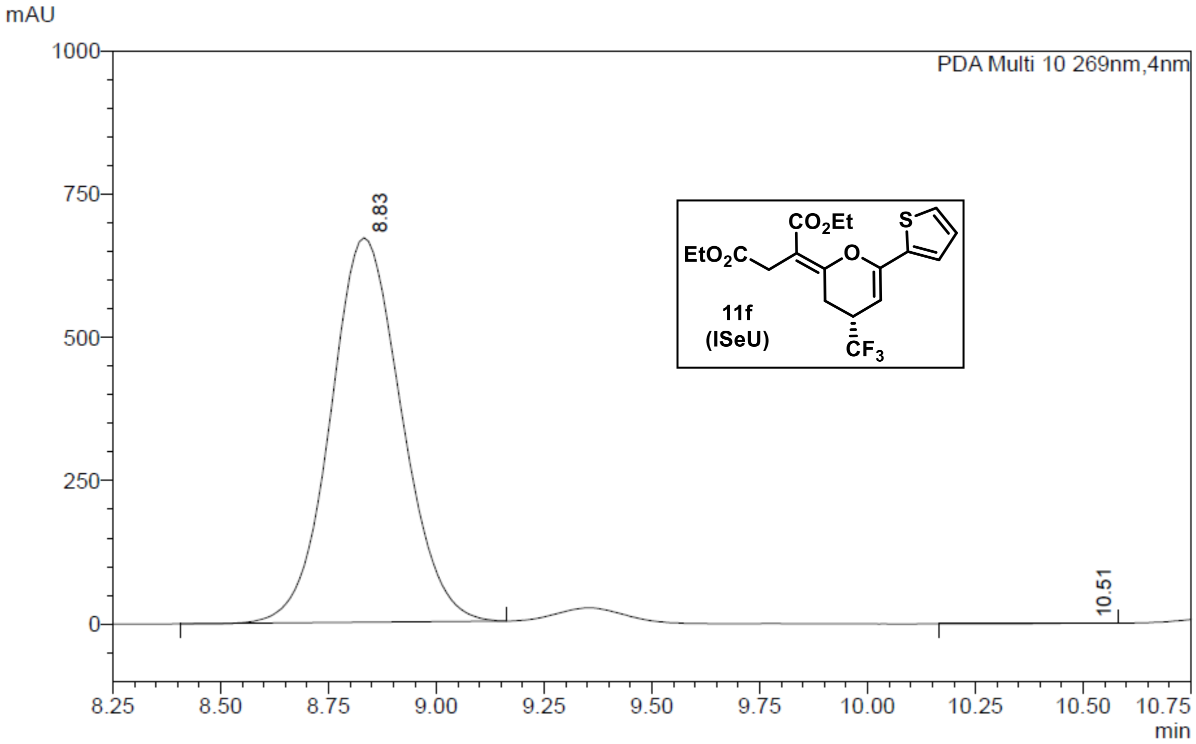

Peak Table

PDA Ch10 269nm

Peak#	Ret. Time	Area	Area%
1	8.83	7696097	99.81
2	10.51	14595	0.19
Total		7710693	100.00

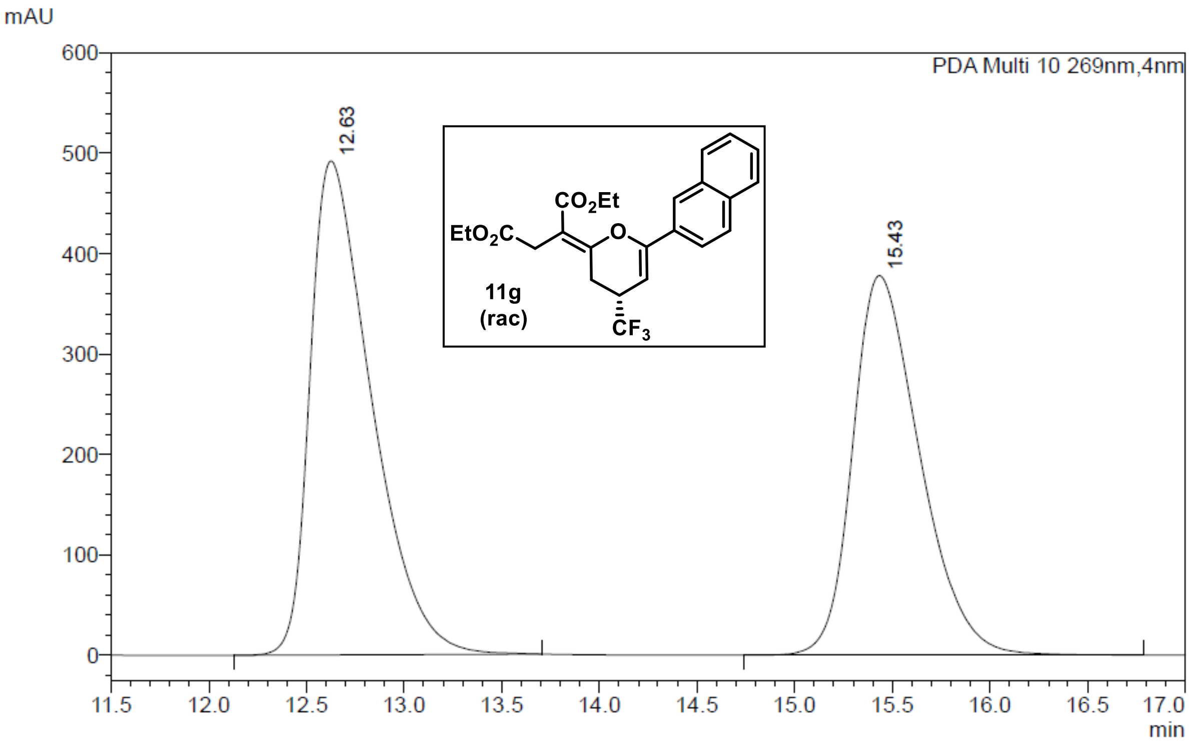

Peak Table

PDA Ch10 269nm

Peak#	Ret. Time	Area	Area%
1	12.63	10758078	54.36
2	15.43	9033262	45.64
Total		19791340	100.00

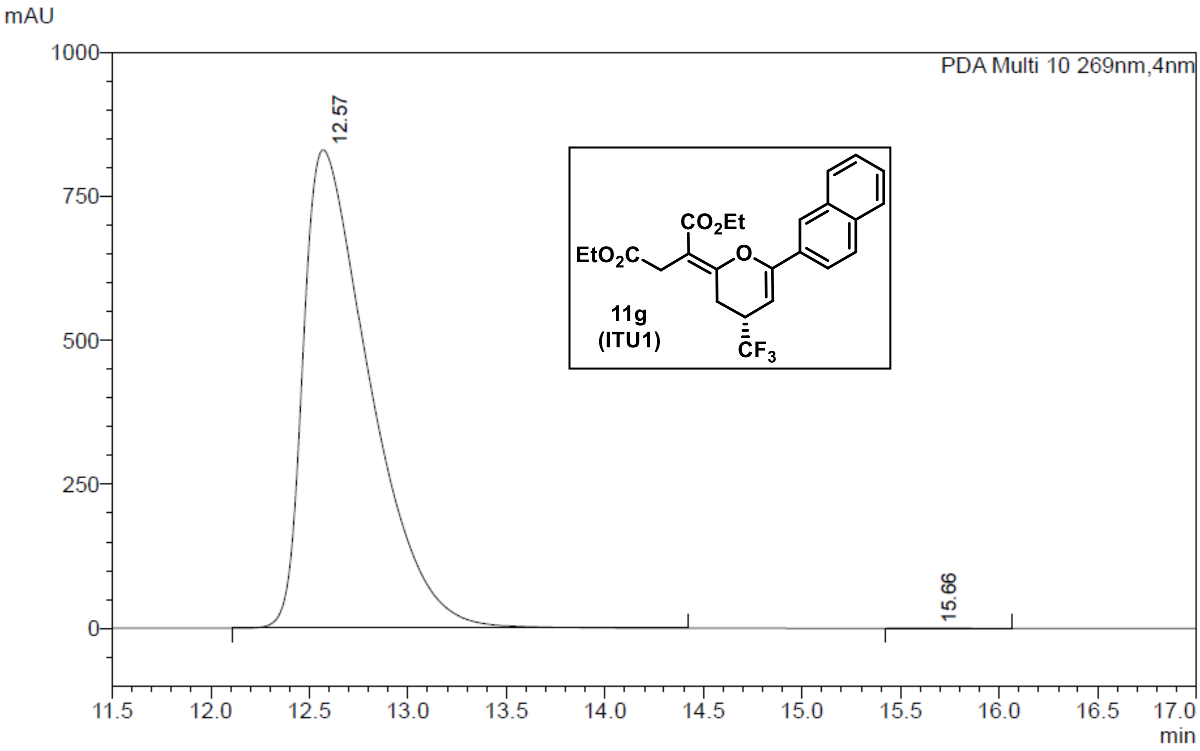

Peak Table

PDA Ch10 269nm

Peak#	Ret. Time	Area	Area%
1	12.57	19513716	99.98
2	15.66	3139	0.02
Total		19516854	100.00

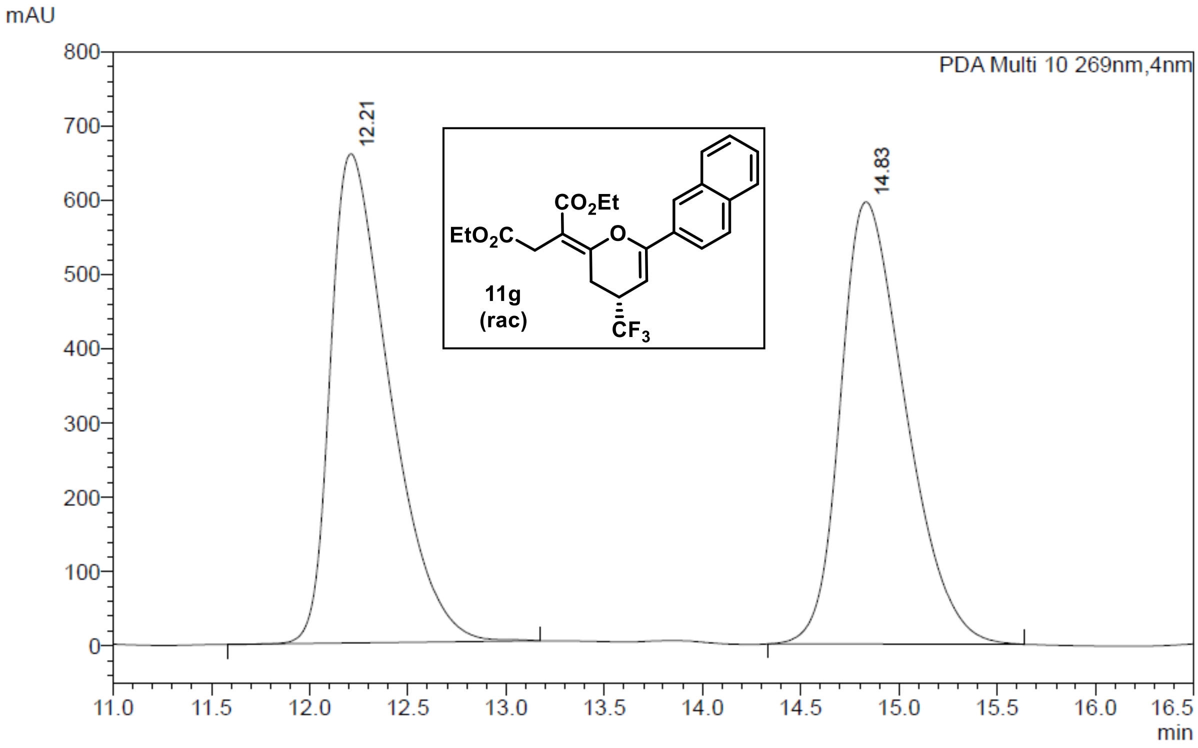

Peak Table

PDA Ch10 269nm

Peak#	Ret. Time	Area	Area%
1	12.21	13804084	50.11
2	14.83	13740862	49.89
Total		27544947	100.00

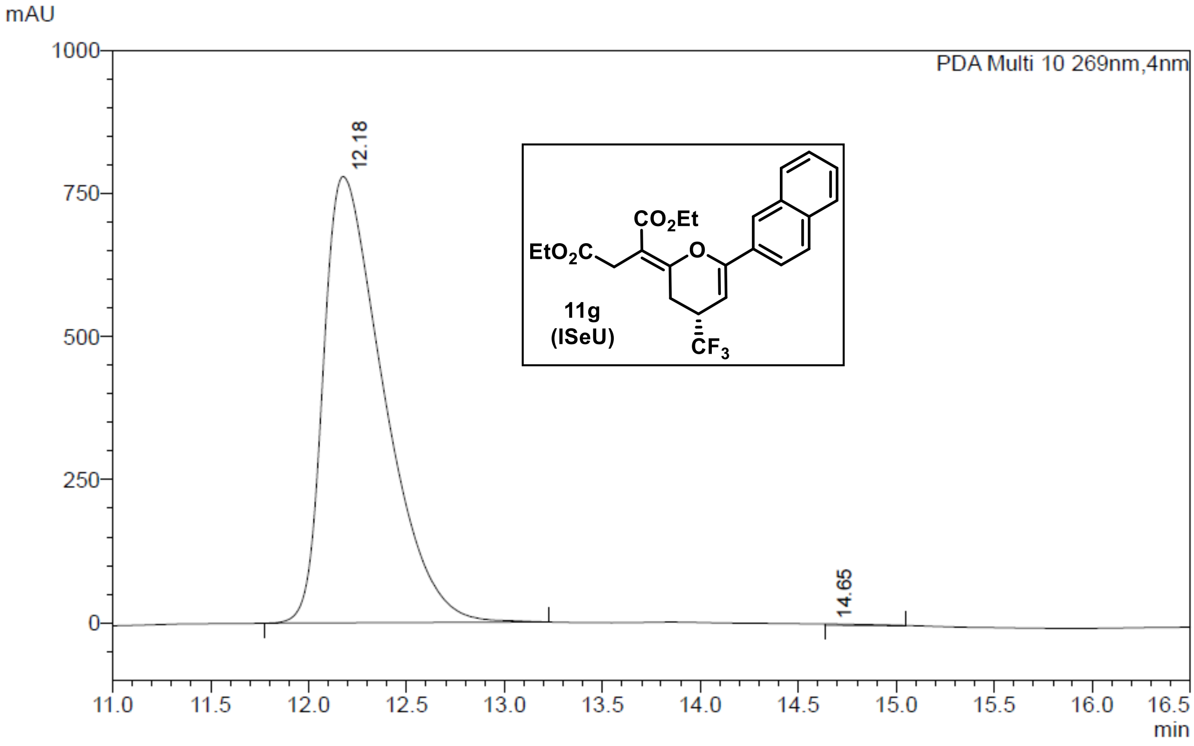

Peak Table

PDA Ch10 269nm

Peak#	Ret. Time	Area	Area%
1	12.18	16651785	99.76
2	14.65	40405	0.24
Total		16692191	100.00

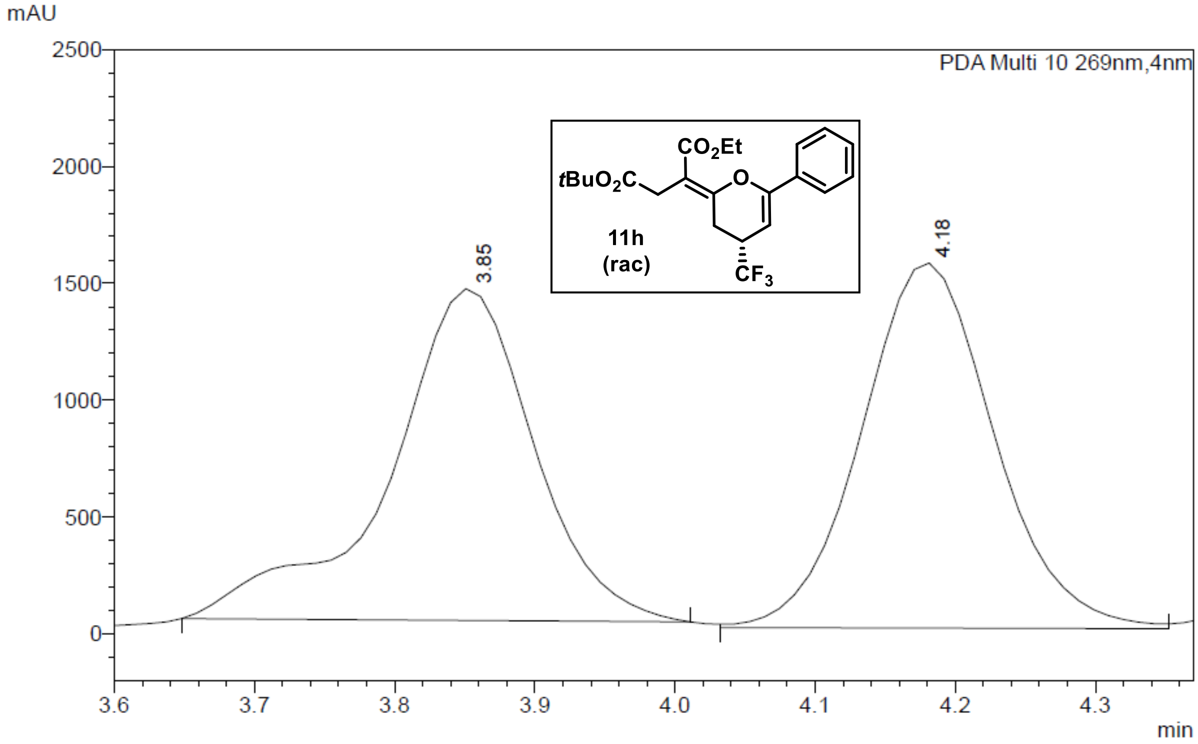

Peak Table

PDA Ch10 269nm

Peak#	Ret. Time	Area	Area%
1	3.85	10240524	49.93
2	4.18	10270878	50.07
Total		20511402	100.00

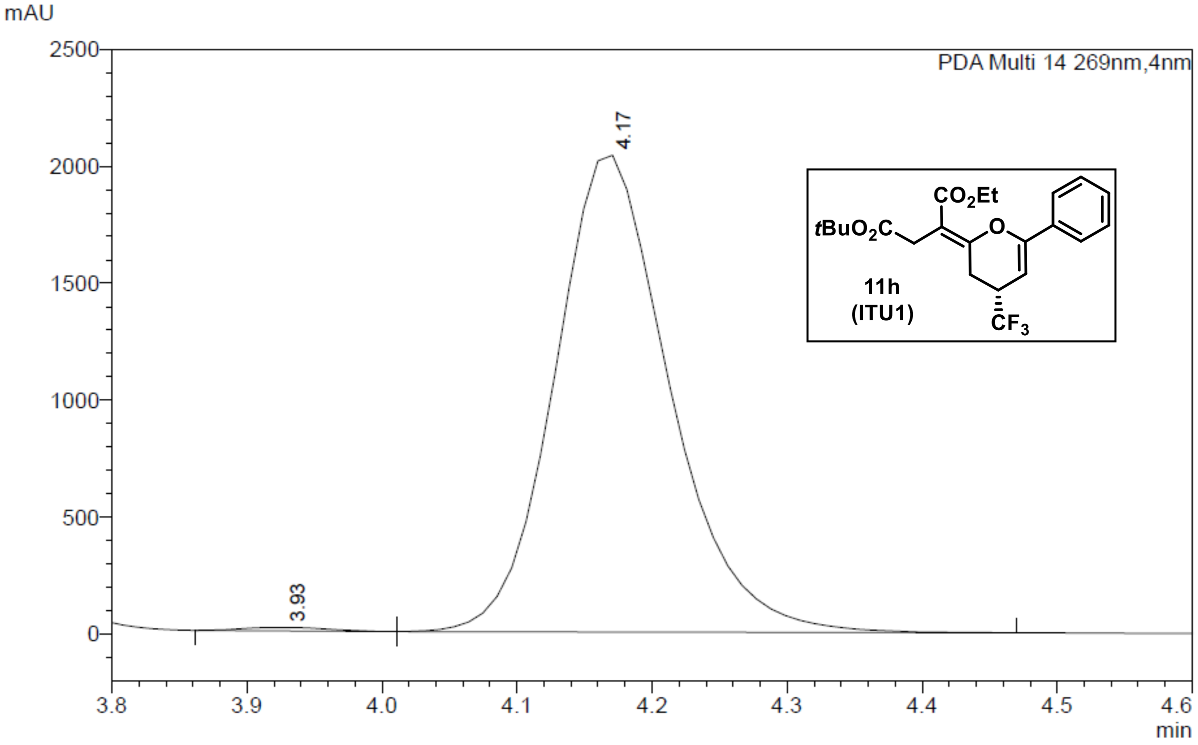

Peak Table

PDA Ch14 269nm

Peak#	Ret. Time	Area	Area%
1	3.93	62968	0.52
2	4.17	12095084	99.48
Total		12158052	100.00

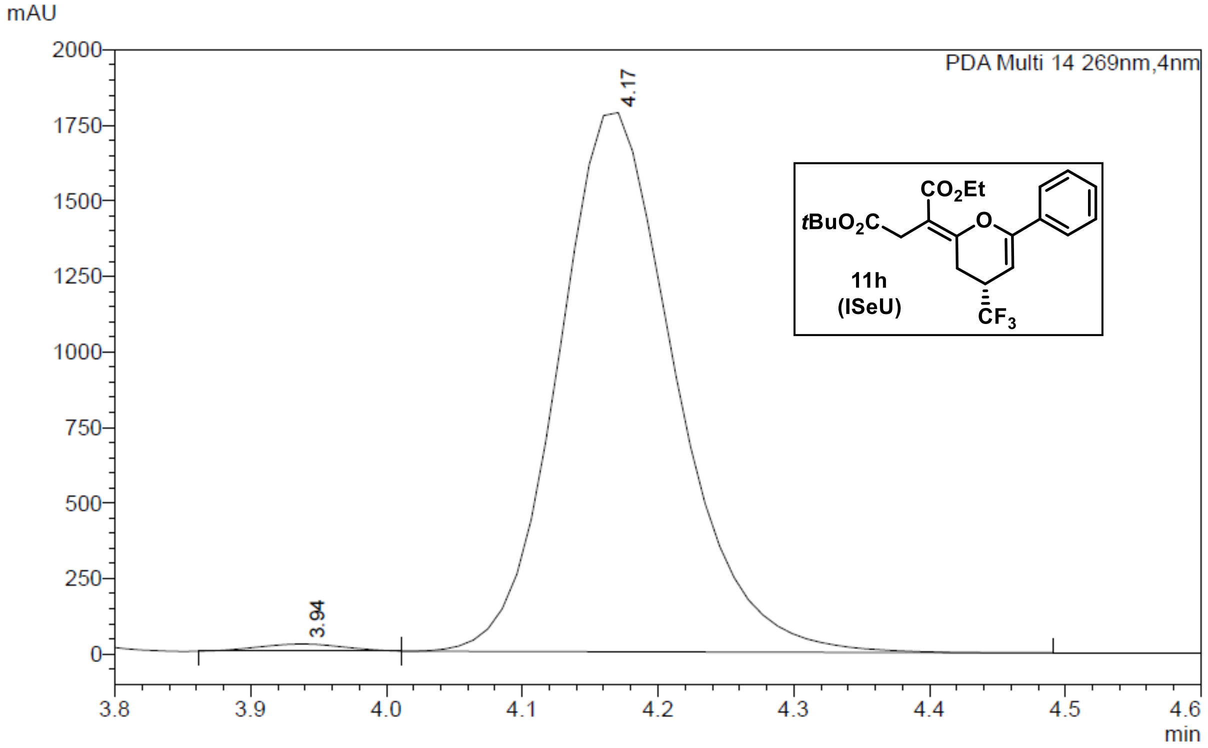

Peak Table

PDA Ch14 269nm

Peak#	Ret. Time	Area	Area%
1	3.94	104919	0.97
2	4.17	10707451	99.03
Total		10812370	100.00

Chromatogram

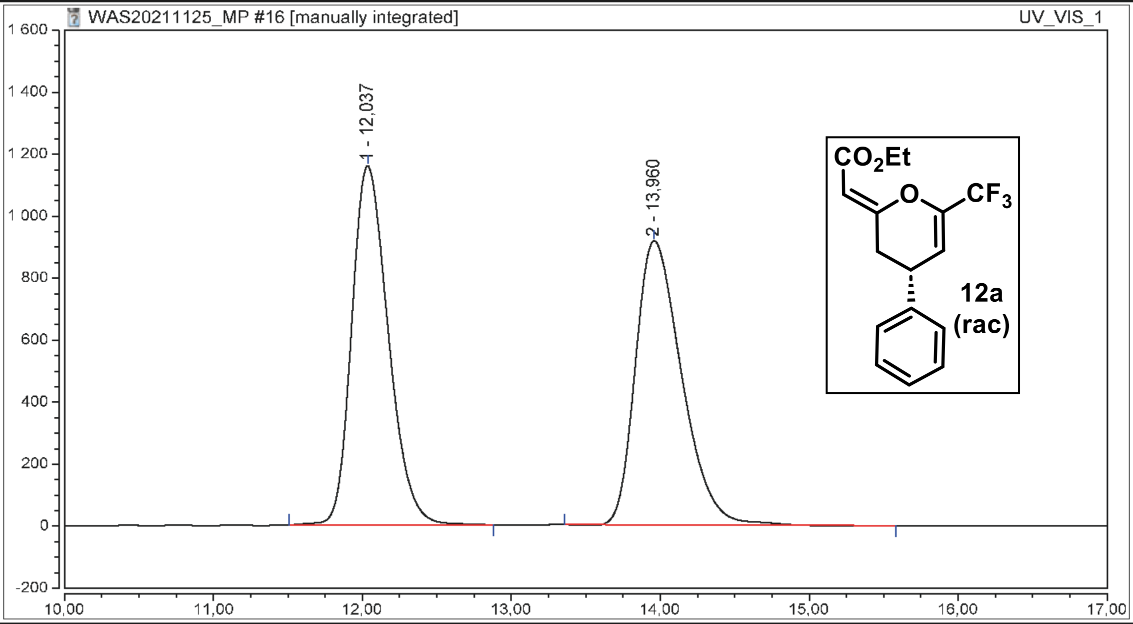

Integration Results

No.	Peak Name	Retention Time min	Area mAU*min	Height mAU	Relative Area %	Relative Height %	Amount n.a.
1		12,037	329,542	1159,193	50,12	55,84	n.a.
2		13,960	327,997	916,603	49,88	44,16	n.a.
**Total:**			**657,539**	**2075,796**	**100,00**	**100,00**	

Chromatogram

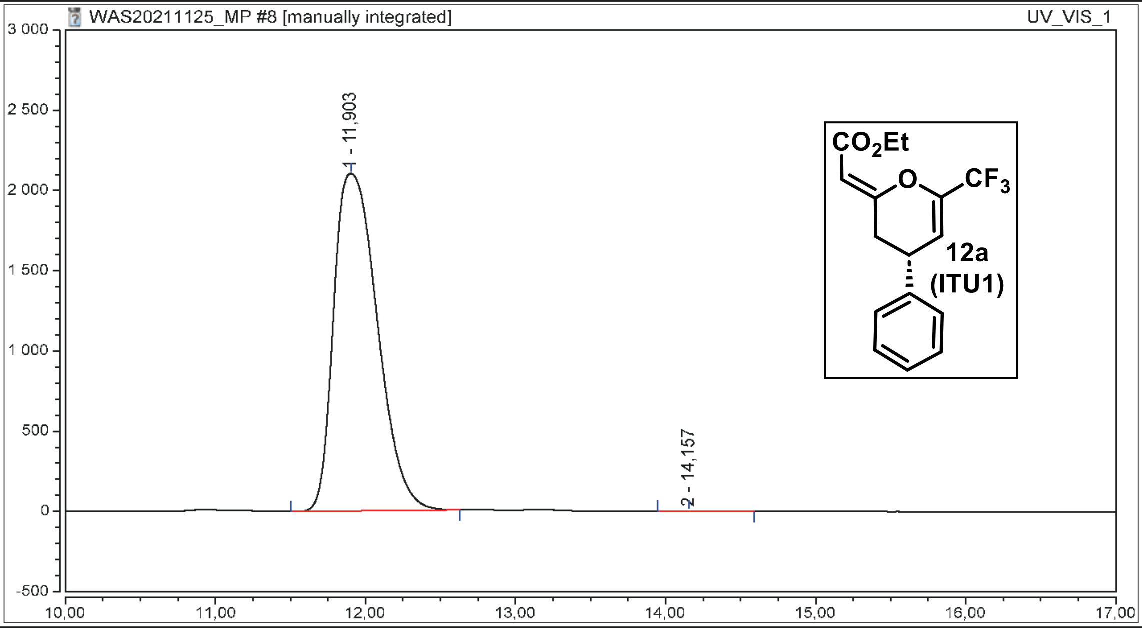

Integration Results

No.	Peak Name	Retention Time min	Area mAU*min	Height mAU	Relative Area %	Relative Height %	Amount n.a.
1		11,903	708,348	2102,868	99,89	99,89	n.a.
2		14,157	0,792	2,380	0,11	0,11	n.a.
**Total:**			**709,140**	**2105,248**	**100,00**	**100,00**	

Chromatogram

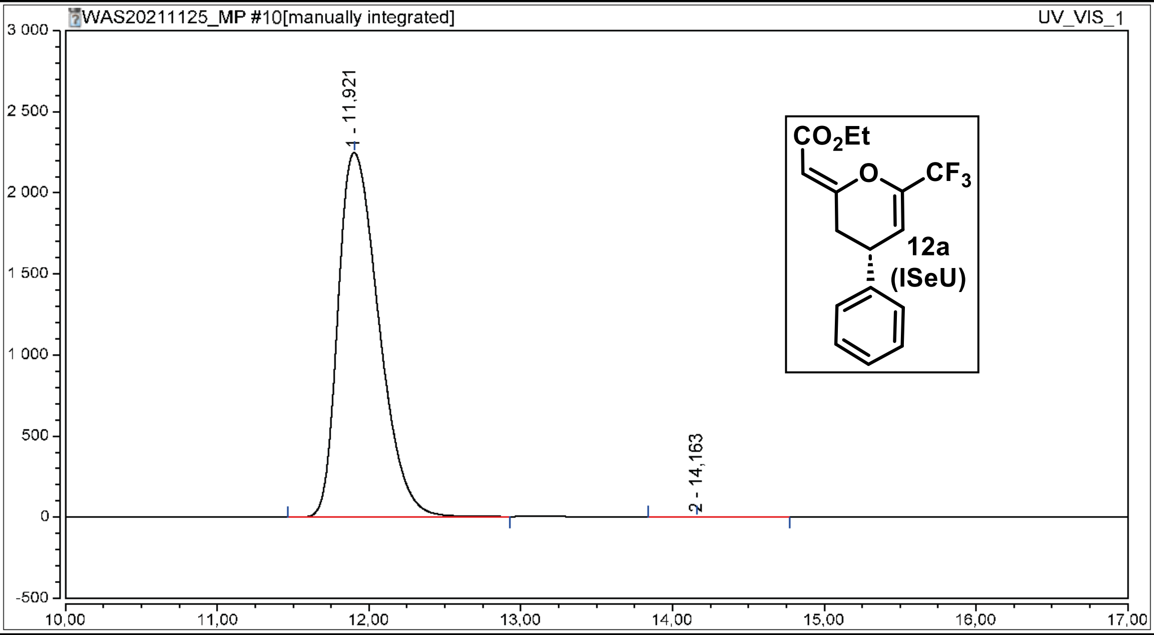

Integration Results

No.	Peak Name	Retention Time min	Area mAU*min	Height mAU	Relative Area %	Relative Height %	Amount n.a.
1		11,921	697,297	2246,661	99,92	99,91	n.a.
2		14,163	0,549	1,937	0,08	0,09	n.a.
**Total:**			**697,845**	**2248,598**	**100,00**	**100,00**	

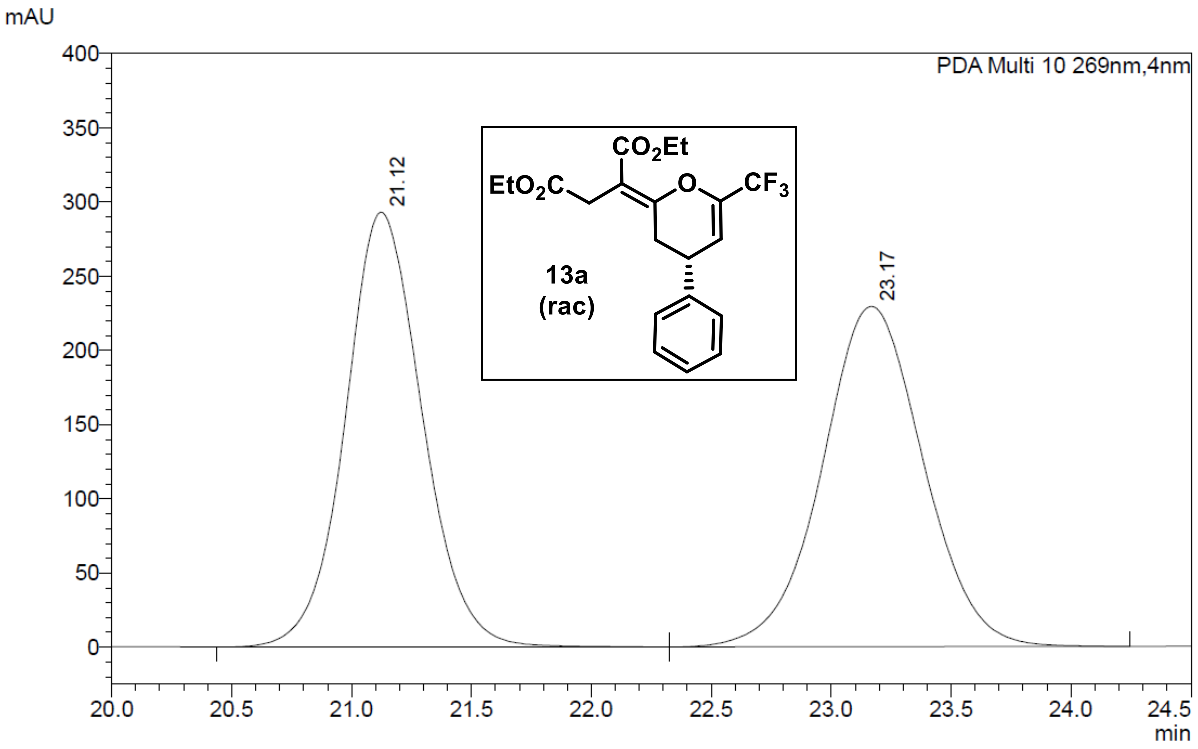

Peak Table

PDA Ch10 269nm

Peak#	Ret. Time	Area	Area%
1	21.12	6541539	49.28
2	23.17	6731765	50.72
Total		13273305	100.00

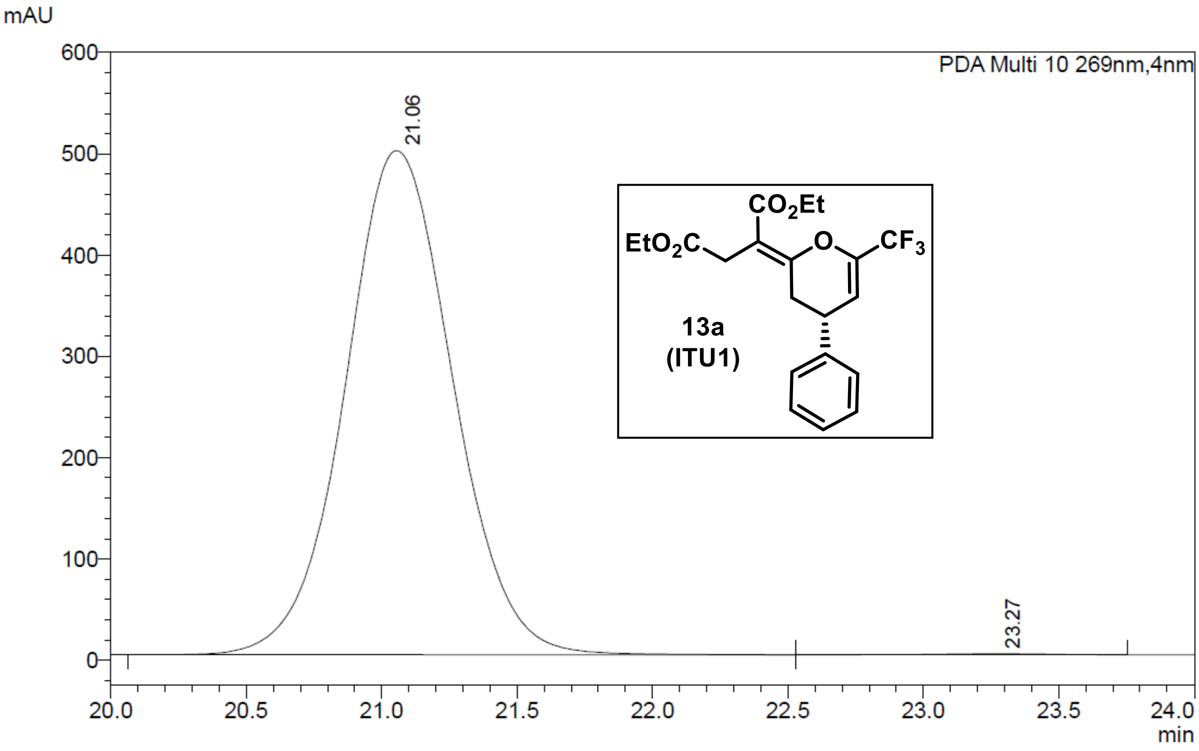

Peak Table

PDA Ch10 269nm

Peak#	Ret. Time	Area	Area%
1	21.06	13777637	99.77
2	23.27	31723	0.23
Total		13809360	100.00

Chromatogram

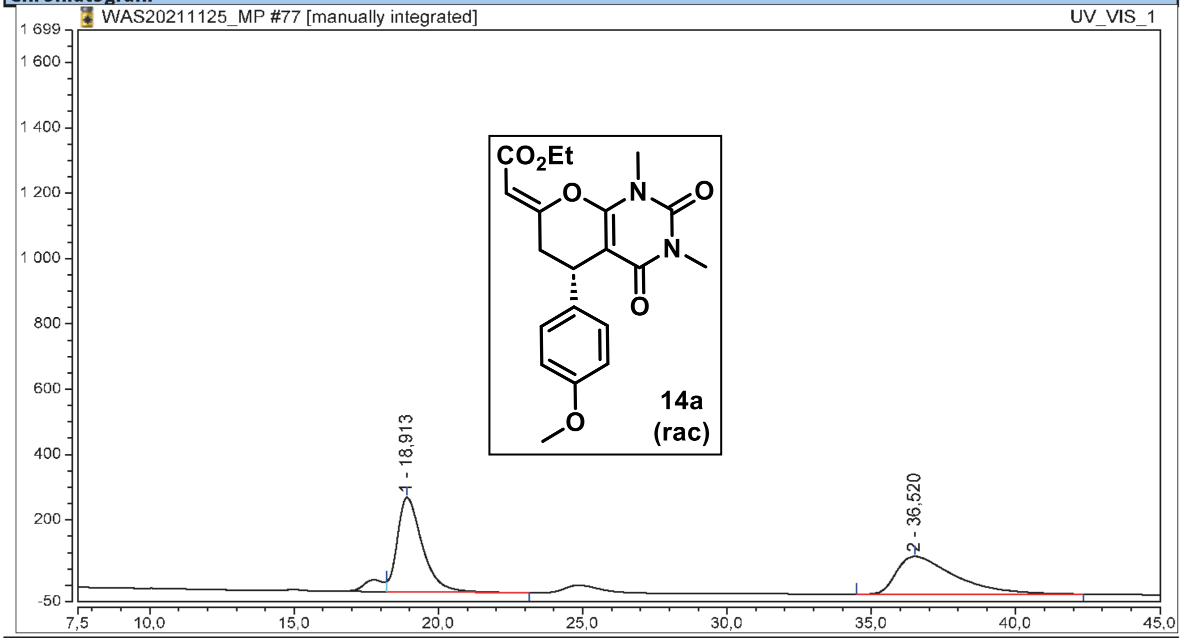

Integration Results

No.	Peak Name	Retention Time<br>min	Area<br>mAU*min	Height<br>mAU	Relative Area<br>%	Relative Height<br>%	Amount<br>n.a.
1		18,913	289,176	288,895	51,84	71,50	n.a.
2		36,520	268,604	115,181	48,16	28,50	n.a.
**Total:**			**557,779**	**404,076**	**100,00**	**100,00**	

Chromatogram

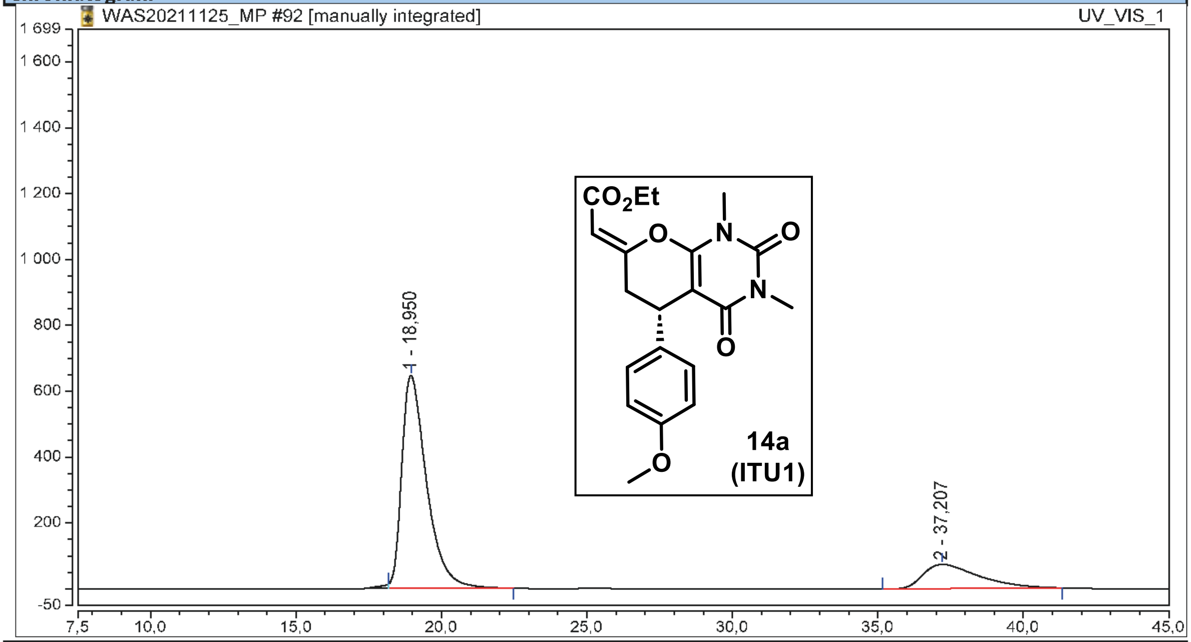

Integration Results

No.	Peak Name	Retention Time<br>min	Area<br>mAU*min	Height<br>mAU	Relative Area<br>%	Relative Height<br>%	Amount<br>n.a.
1		18,950	639,383	647,825	79,62	89,81	n.a.
2		37,207	163,700	73,524	20,38	10,19	n.a.
**Total:**			**803,083**	**721,348**	**100,00**	**100,00**	

Chromatogram

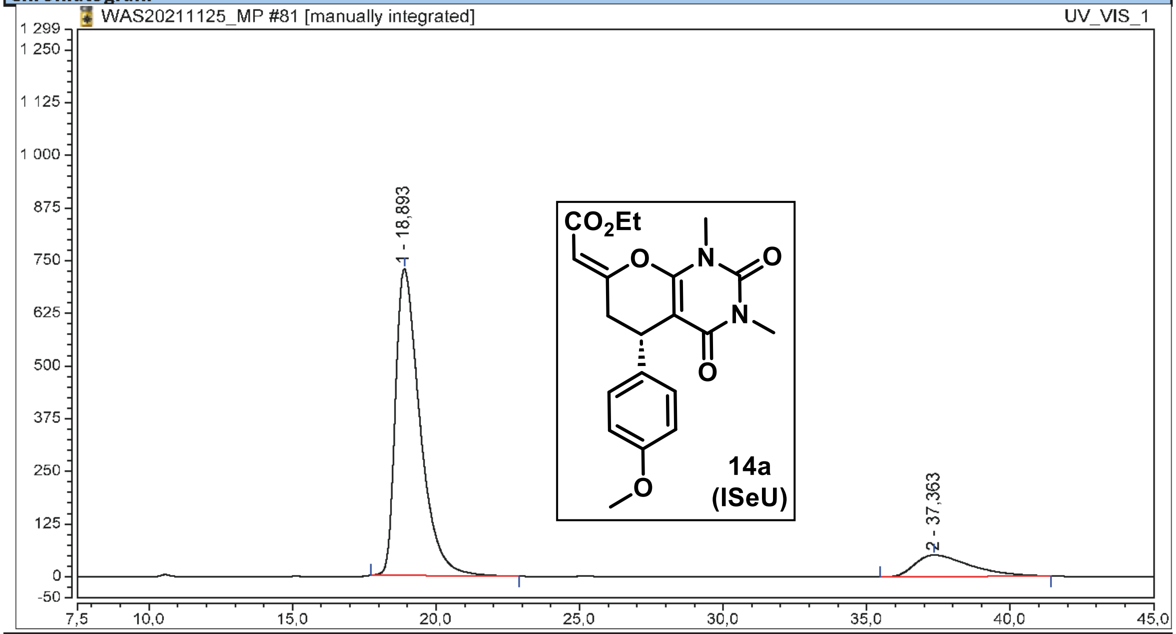

Integration Results

No.	Peak Name	Retention Time<br>min	Area<br>mAU*min	Height<br>mAU	Relative Area<br>%	Relative Height<br>%	Amount<br>n.a.
1		18,893	718,958	728,529	86,28	93,42	n.a.
2		37,363	114,318	51,281	13,72	6,58	n.a.
**Total:**			**833,276**	**779,810**	**100,00**	**100,00**	

Chromatogram

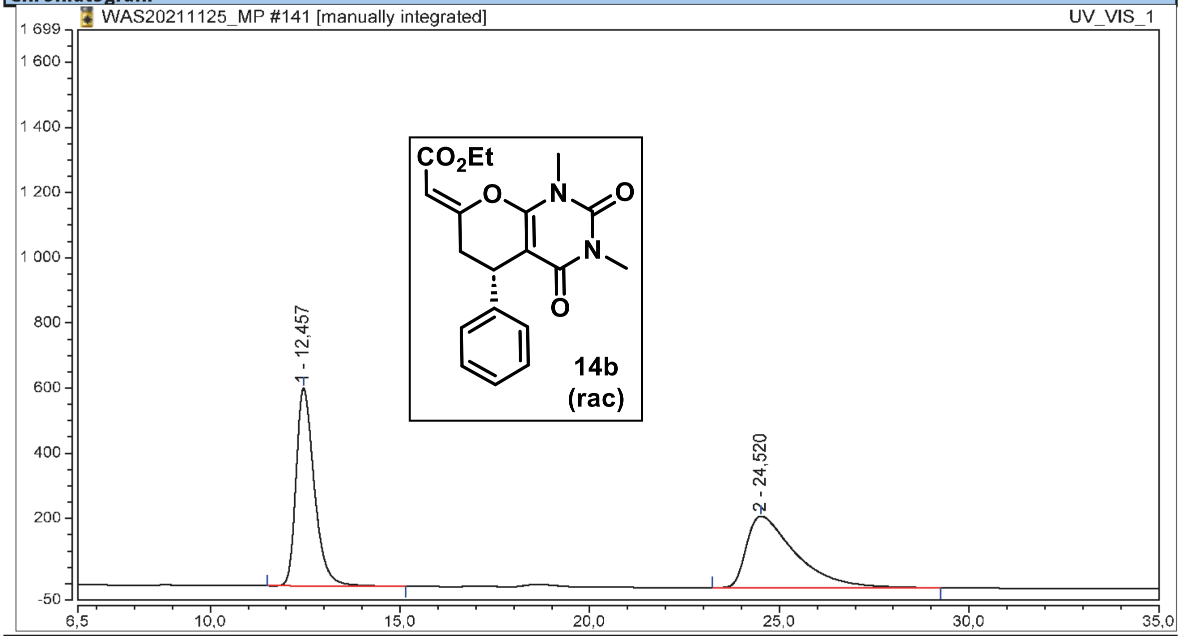

Integration Results

No.	Peak Name	Retention Time min	Area mAU*min	Height mAU	Relative Area %	Relative Height %	Amount n.a.
1		12,457	337,433	607,409	50,77	73,47	n.a.
2		24,520	327,198	219,307	49,23	26,53	n.a.
**Total:**			**664,632**	**826,716**	**100,00**	**100,00**	

Chromatogram

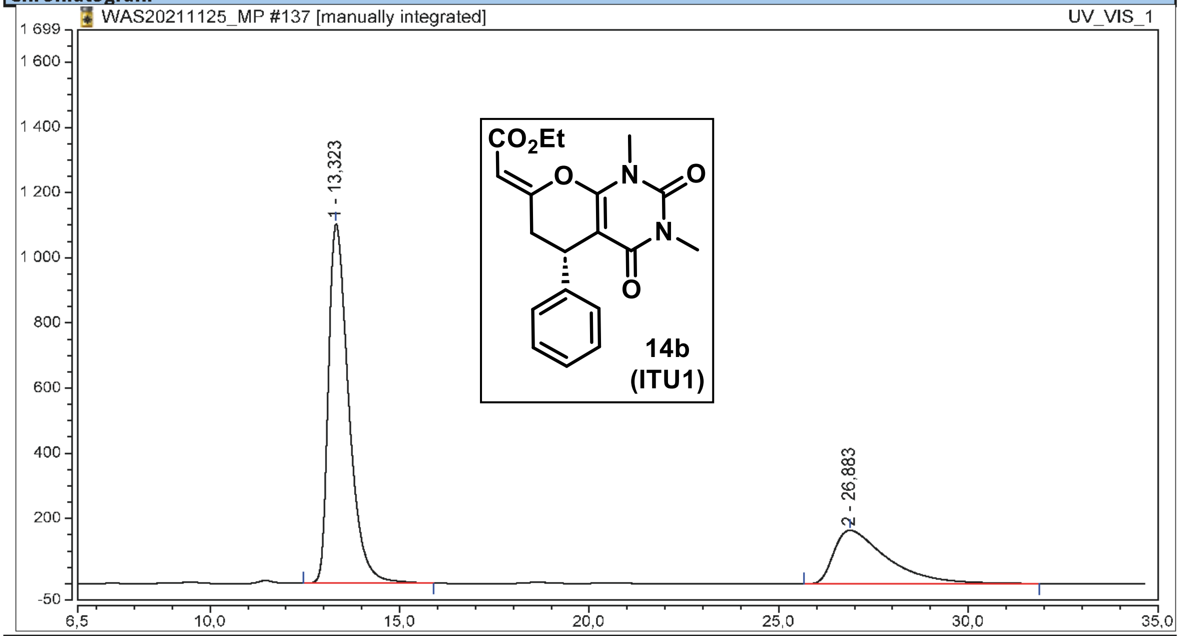

Integration Results

No.	Peak Name	Retention Time min	Area mAU*min	Height mAU	Relative Area %	Relative Height %	Amount n.a.
1		13,323	681,851	1104,138	71,87	87,08	n.a.
2		26,883	266,905	163,842	28,13	12,92	n.a.
**Total:**			**948,756**	**1267,980**	**100,00**	**100,00**	

Chromatogram

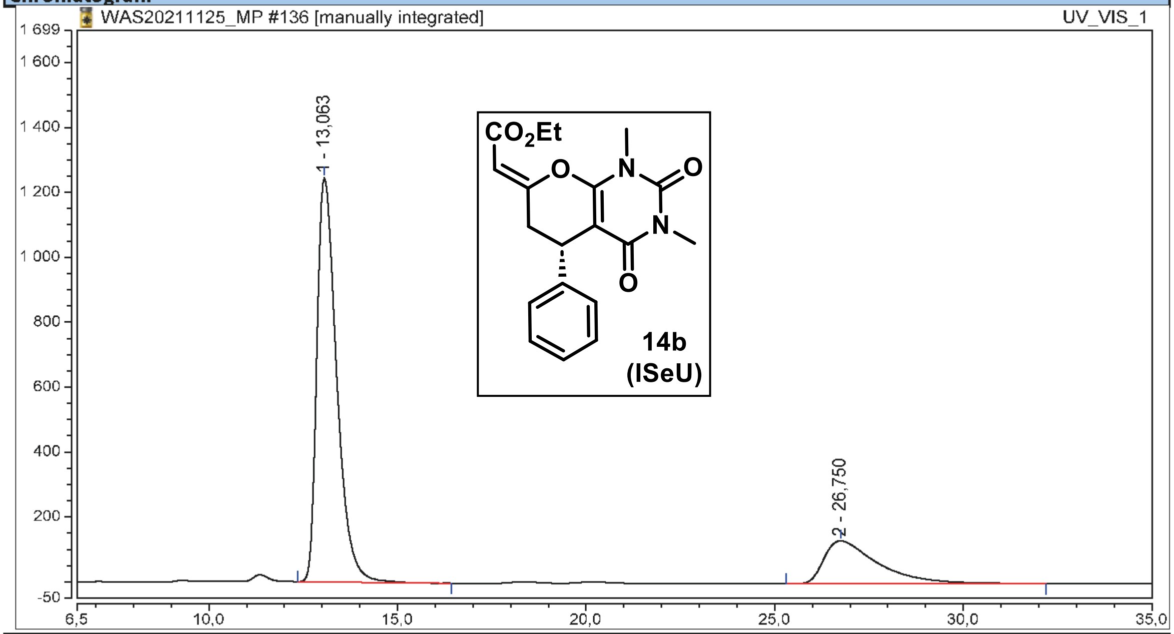

Integration Results

No.	Peak Name	Retention Time min	Area mAU*min	Height mAU	Relative Area %	Relative Height %	Amount n.a.
1		13,063	757,372	1246,408	77,63	90,42	n.a.
2		26,750	218,240	131,988	22,37	9,58	n.a.
**Total:**			**975,612**	**1378,396**	**100,00**	**100,00**	

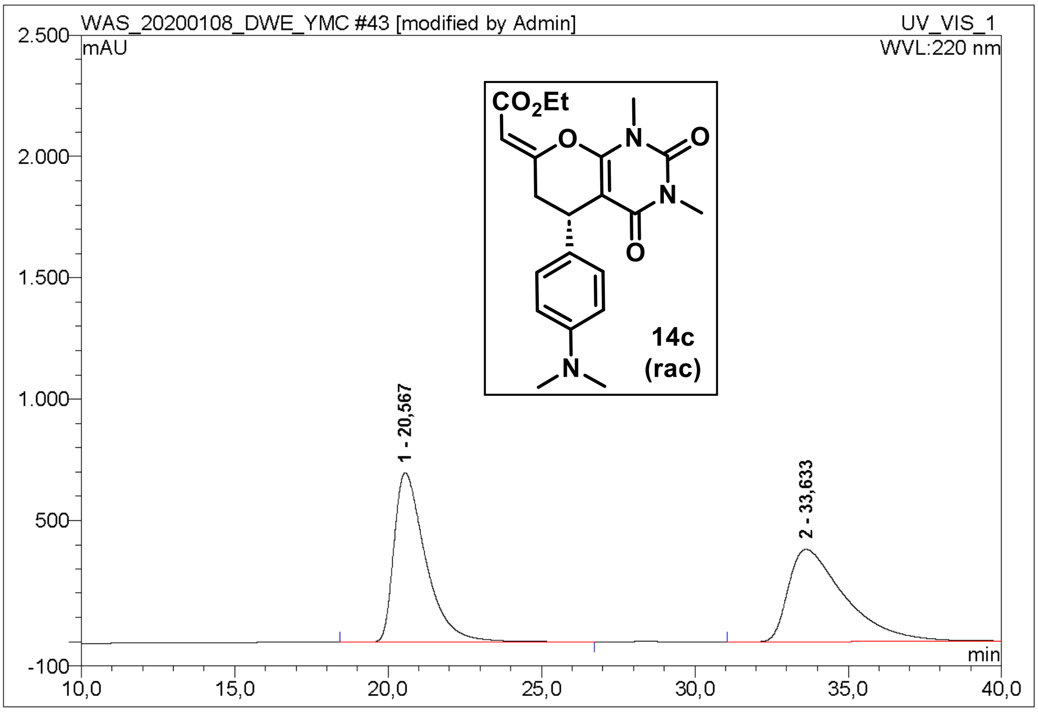

No.	Ret.Time min	Peak Name	Height mAU	Area mAU*min	Rel.Area %	Amount	Type
1	20,57	n.a.	697,957	837,357	50,68	n.a.	BMB*
2	33,63	n.a.	381,029	814,824	49,32	n.a.	BMB*
**Total:**			1078,986	1652,181	100,00	0,000	

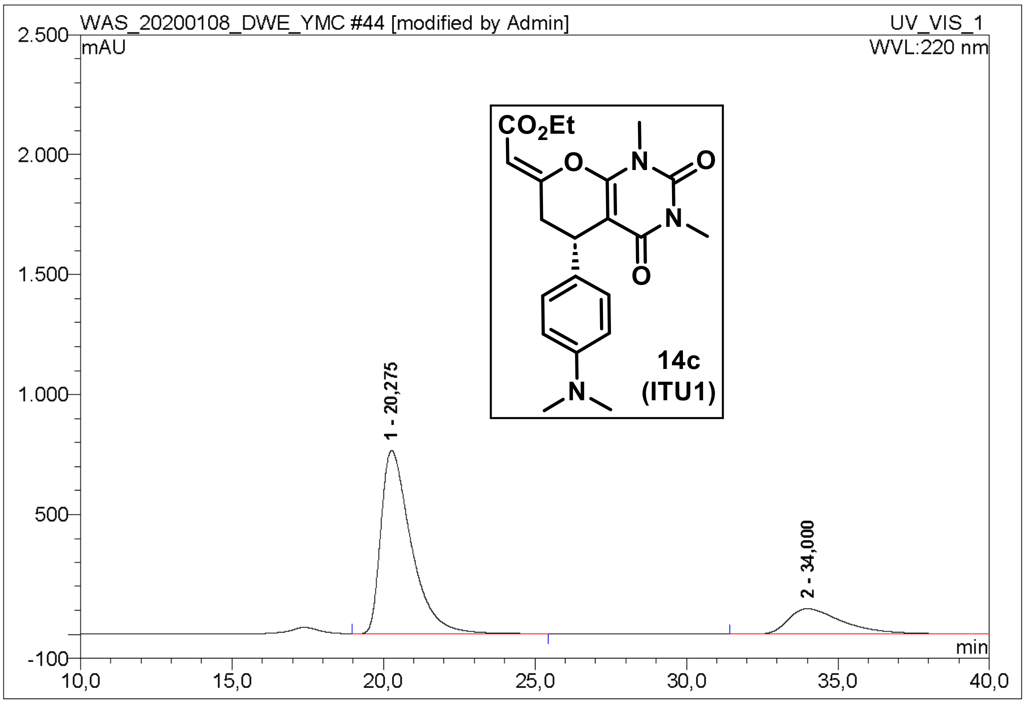

No.	Ret.Time min	Peak Name	Height mAU	Area mAU*min	Rel.Area %	Amount	Type
1	20,28	n.a.	765,592	888,239	79,84	n.a.	BMB*
2	34,00	n.a.	106,063	224,237	20,16	n.a.	BMB*
**Total:**			871,655	1112,476	100,00	0,000	

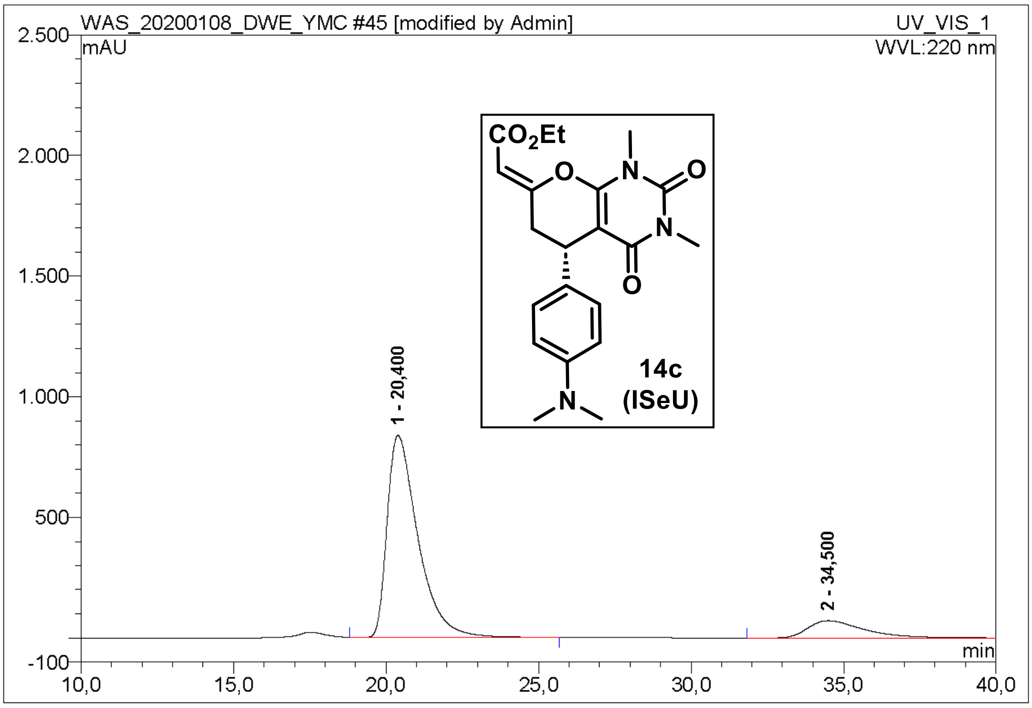

No.	Ret.Time min	Peak Name	Height mAU	Area mAU*min	Rel.Area %	Amount	Type
1	20,40	n.a.	838,233	983,394	86,38	n.a.	BMB*
2	34,50	n.a.	71,714	155,060	13,62	n.a.	BMB*
Total:			909,948	1138,454	100,00	0,000	

Chromatogram

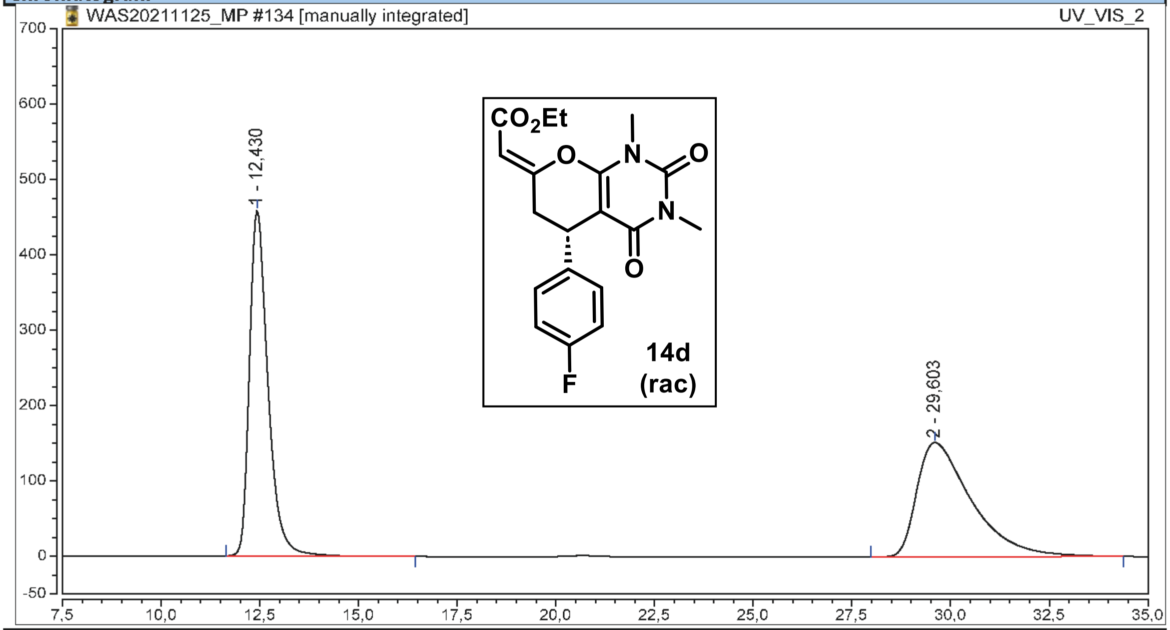

Integration Results

No.	Peak Name	Retention Time<br>min	Area<br>mAU*min	Height<br>mAU	Relative Area<br>%	Relative Height<br>%	Amount<br>n.a.
1		12,430	249,543	459,115	50,65	75,16	n.a.
2		29,603	243,166	151,733	49,35	24,84	n.a.
**Total:**			**492,709**	**610,848**	**100,00**	**100,00**	

Chromatogram

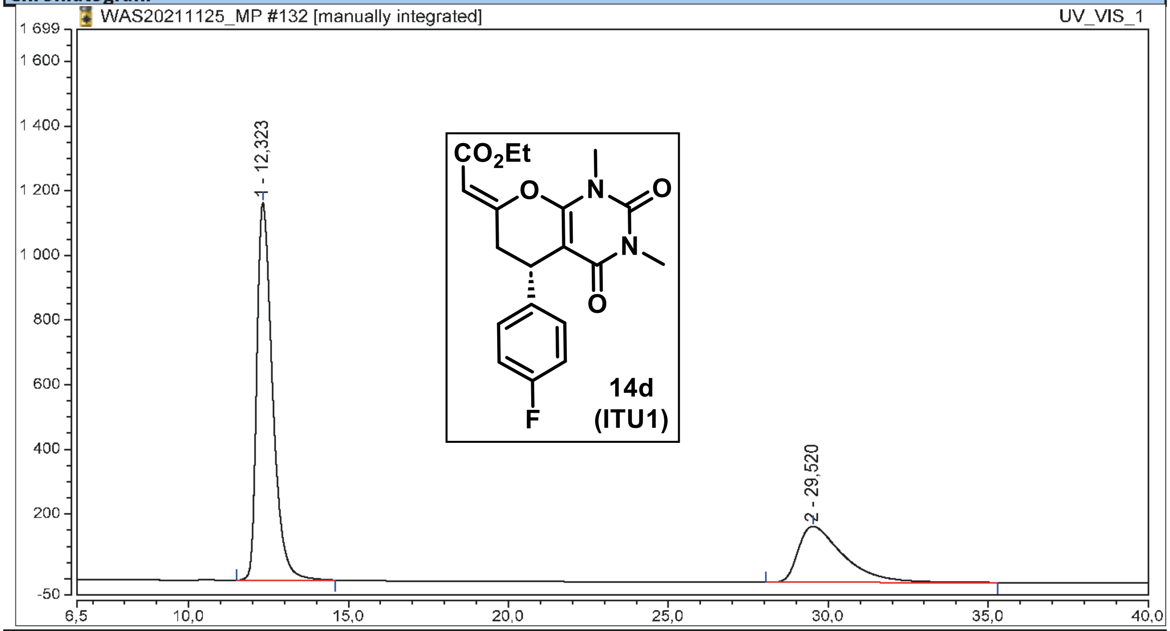

Integration Results

No.	Peak Name	Retention Time<br>min	Area<br>mAU*min	Height<br>mAU	Relative Area<br>%	Relative Height<br>%	Amount<br>n.a.
1		12,323	663,363	1168,552	70,24	87,09	n.a.
2		29,520	281,069	173,186	29,76	12,91	n.a.
**Total:**			**944,432**	**1341,738**	**100,00**	**100,00**	

Chromatogram

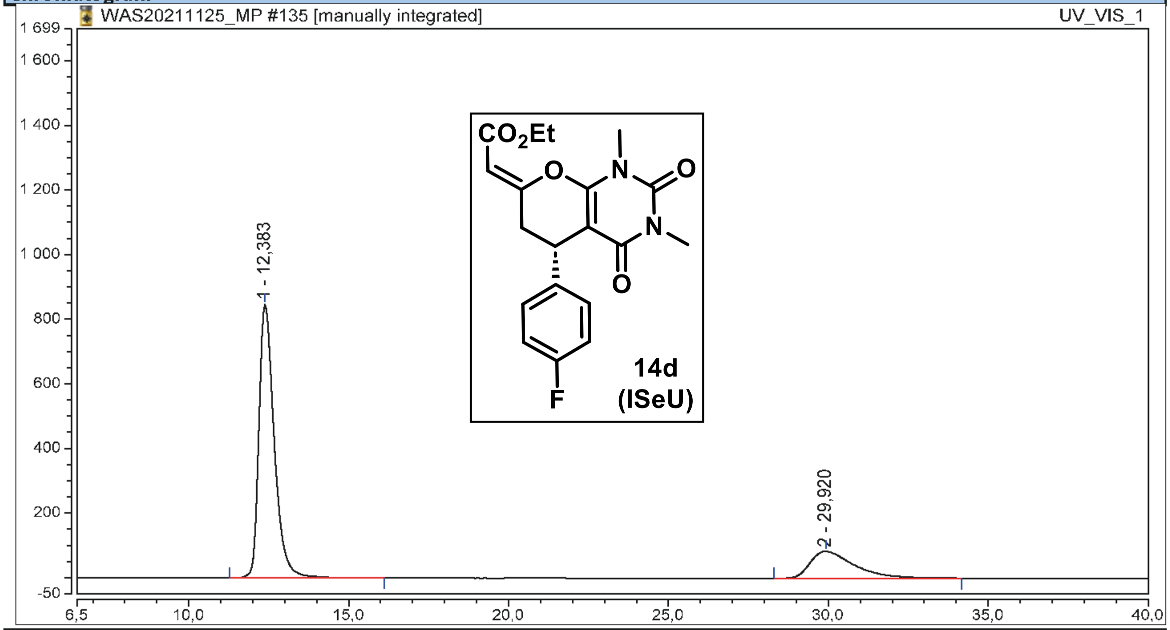

Integration Results

No.	Peak Name	Retention Time<br>min	Area<br>mAU*min	Height<br>mAU	Relative Area<br>%	Relative Height<br>%	Amount<br>n.a.
1		12,383	469,369	846,748	77,89	91,02	n.a.
2		29,920	133,265	83,512	22,11	8,98	n.a.
**Total:**			**602,634**	**930,261**	**100,00**	**100,00**	

Chromatogram

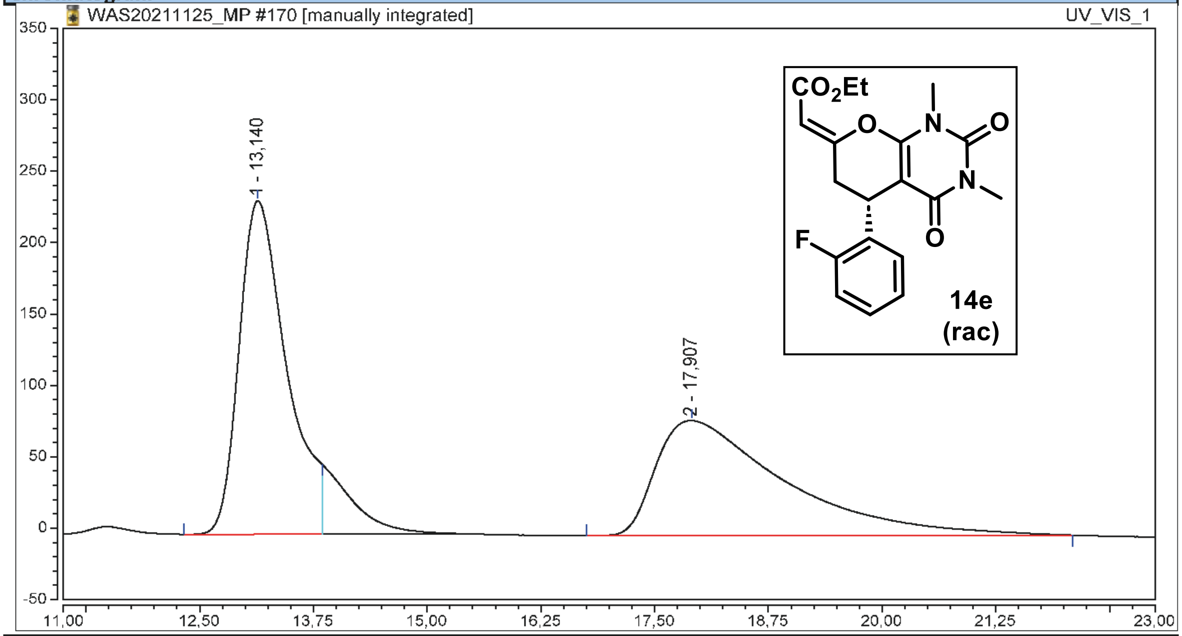

Integration Results

No.	Peak Name	Retention Time min	Area mAU*min	Height mAU	Relative Area %	Relative Height %	Amount n.a.
1		13,140	140,946	233,515	51,93	74,38	n.a.
2		17,907	130,494	80,413	48,07	25,62	n.a.
**Total:**			**271,440**	**313,927**	**100,00**	**100,00**	

Chromatogram

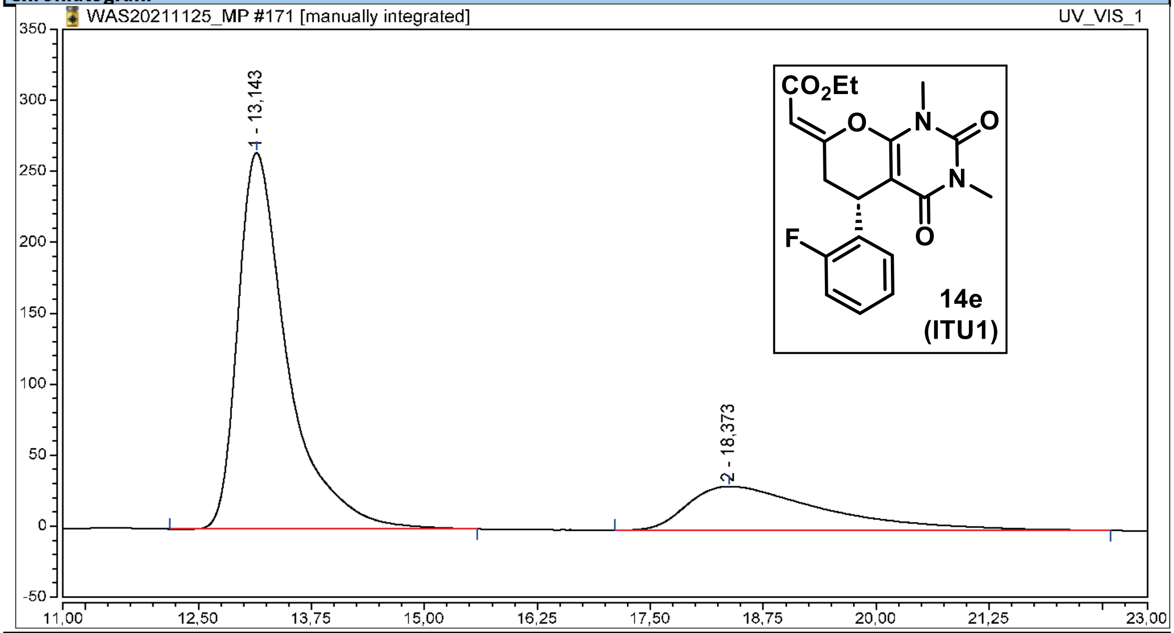

Integration Results

No.	Peak Name	Retention Time min	Area mAU*min	Height mAU	Relative Area %	Relative Height %	Amount n.a.
1		13,143	168,575	265,254	75,80	89,57	n.a.
2		18,373	53,825	30,881	24,20	10,43	n.a.
**Total:**			**222,400**	**296,135**	**100,00**	**100,00**	

Chromatogram

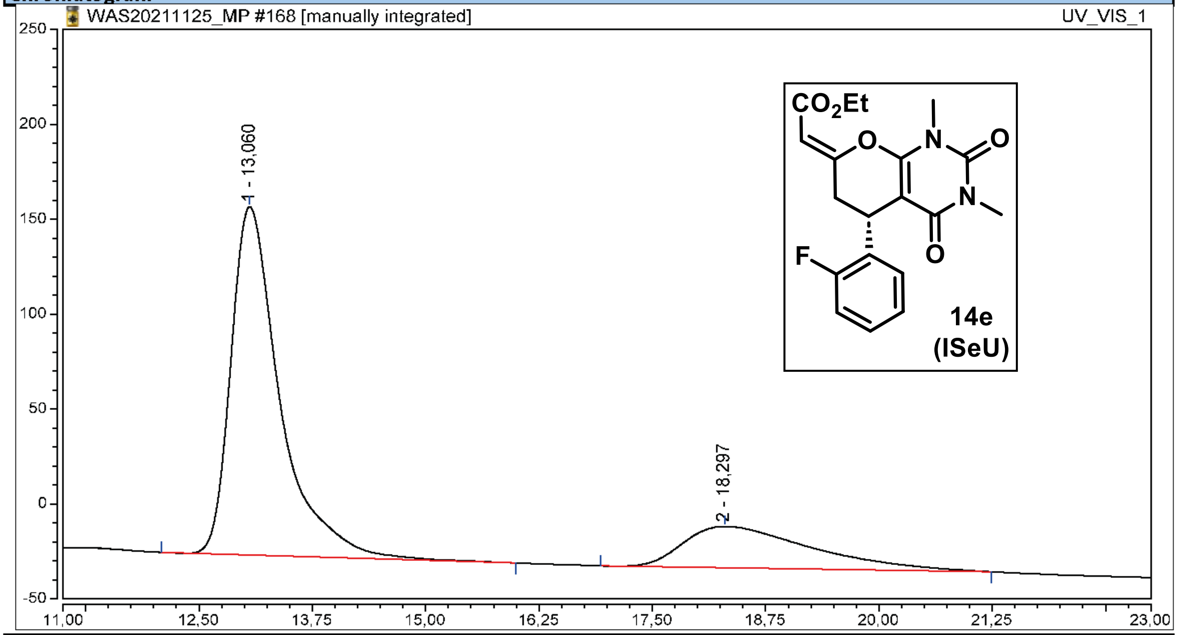

Integration Results

No.	Peak Name	Retention Time min	Area mAU*min	Height mAU	Relative Area %	Relative Height %	Amount n.a.
1		13,060	114,836	183,697	76,35	89,41	n.a.
2		18,297	35,572	21,752	23,65	10,59	n.a.
**Total:**			**150,408**	**205,449**	**100,00**	**100,00**	

Chromatogram

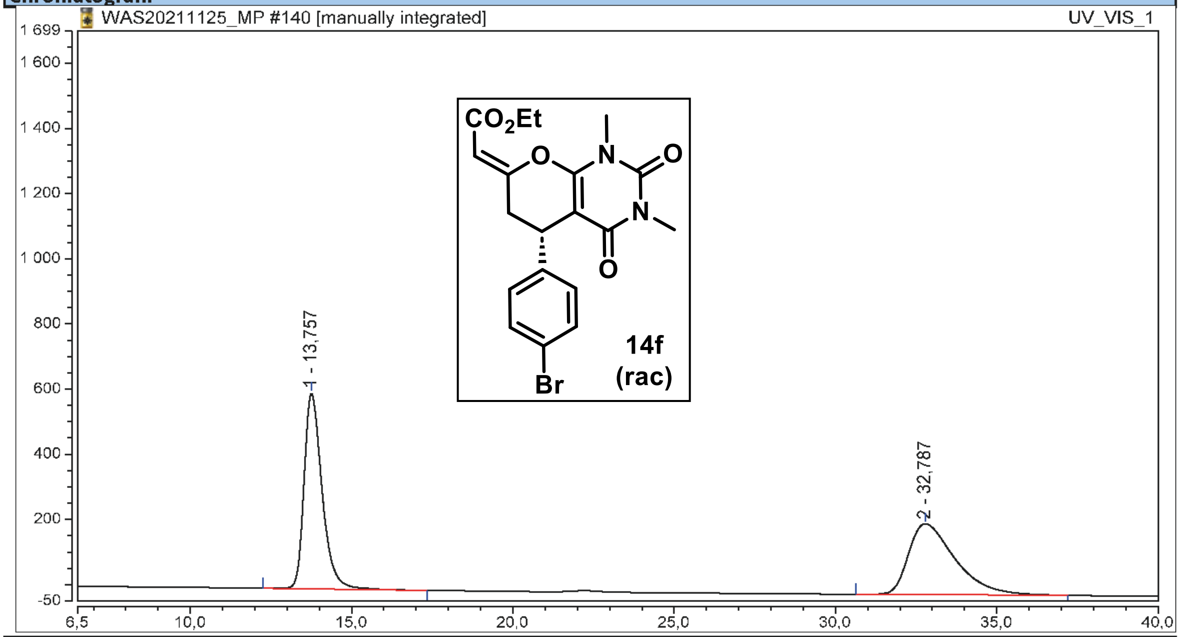

Integration Results

No.	Peak Name	Retention Time min	Area mAU*min	Height mAU	Relative Area %	Relative Height %	Amount n.a.
1		13,757	389,343	601,885	51,69	73,48	n.a.
2		32,787	363,914	217,233	48,31	26,52	n.a.
**Total:**			**753,257**	**819,118**	**100,00**	**100,00**	

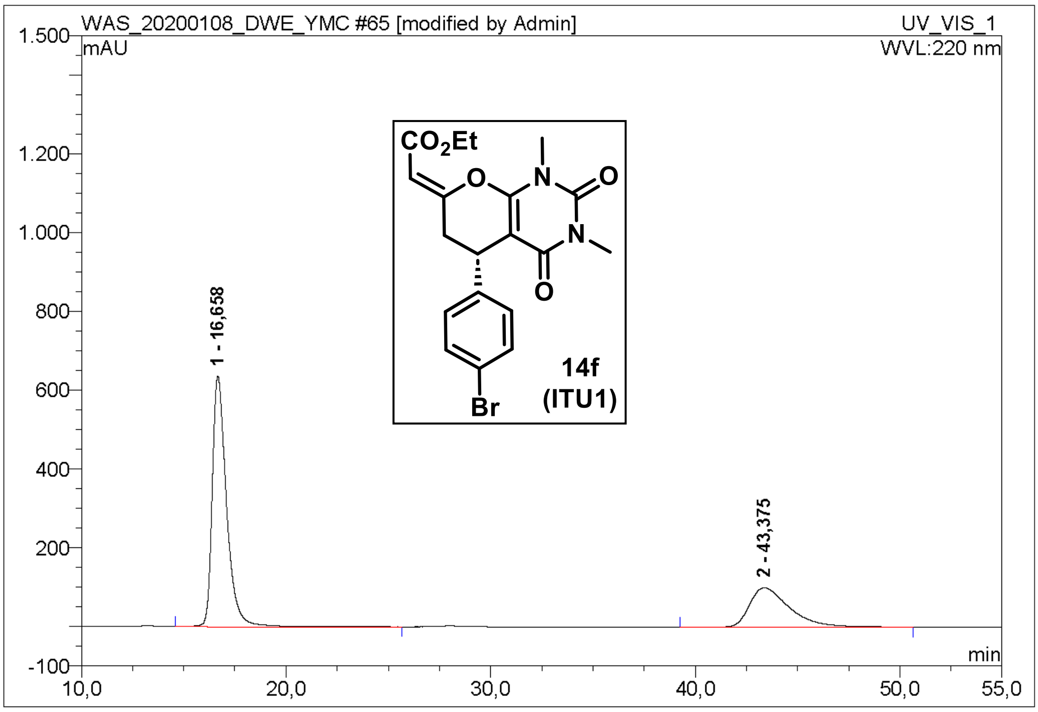

No.	Ret.Time min	Peak Name	Height mAU	Area mAU*min	Rel.Area %	Amount	Type
1	16,66	n.a.	635,957	515,943	69,60	n.a.	BMB*
2	43,38	n.a.	99,366	225,381	30,40	n.a.	BMB*
**Total:**			735,323	741,324	100,00	0,000	

Chromatogram

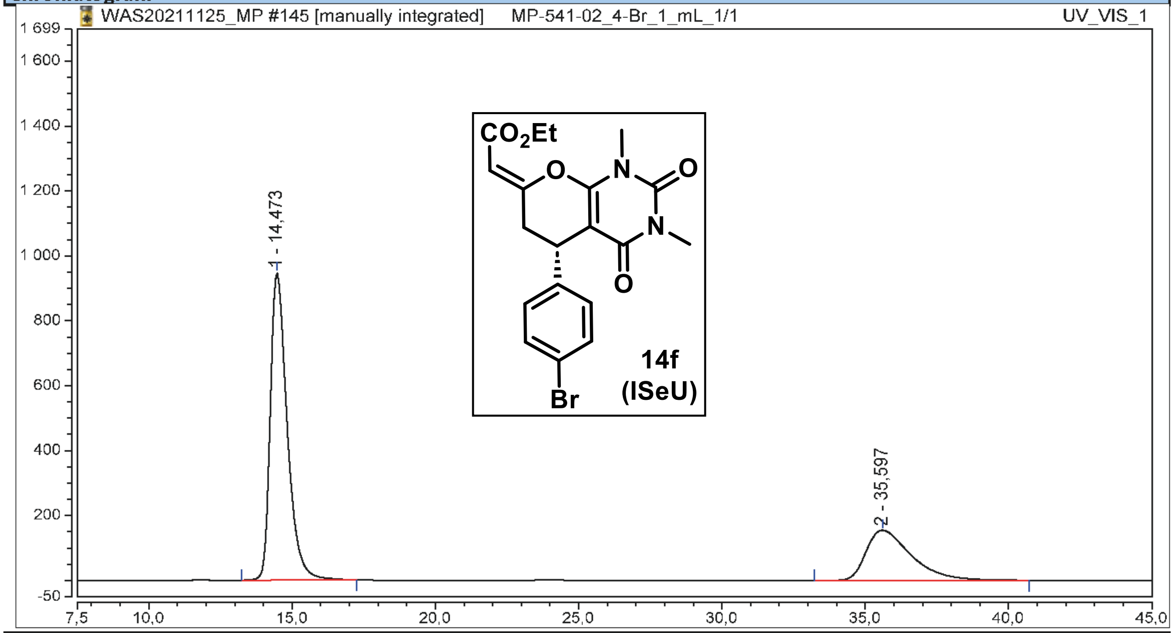

Integration Results

No.	Peak Name	Retention Time min	Area mAU*min	Height mAU	Relative Area %	Relative Height %	Amount n.a.
1		14,473	645,687	946,640	69,45	85,92	n.a.
2		35,597	284,049	155,154	30,55	14,08	n.a.
**Total:**			**929,737**	**1101,794**	**100,00**	**100,00**	

Chromatogram

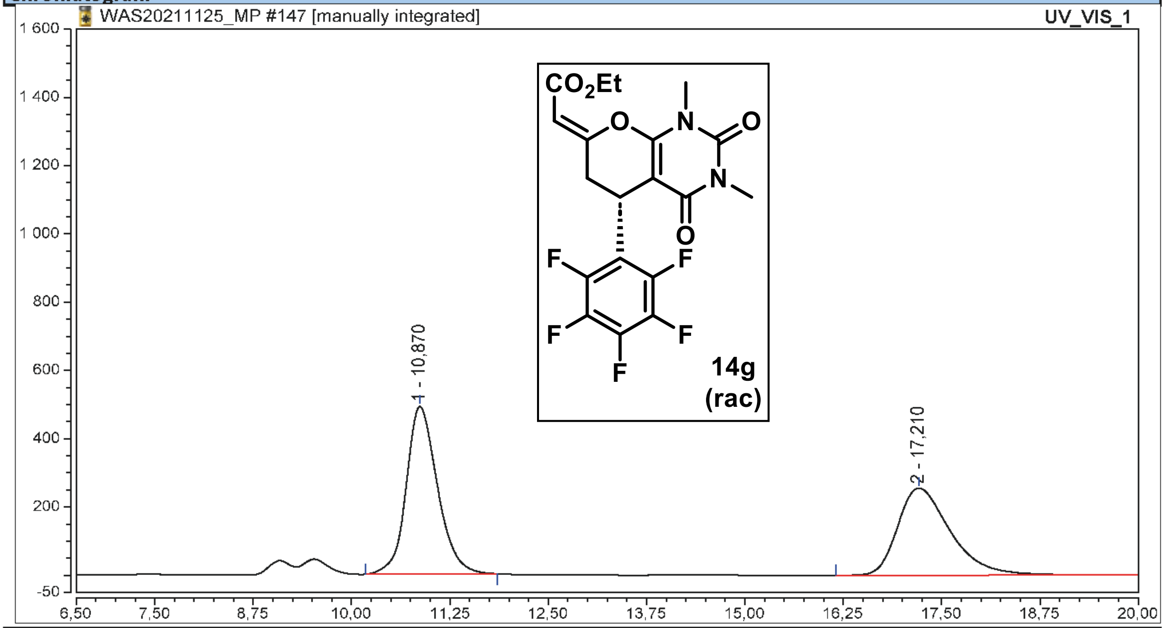

Integration Results

No.	Peak Name	Retention Time min	Area mAU*min	Height mAU	Relative Area %	Relative Height %	Amount n.a.
1		10,870	226,083	491,500	53,23	65,82	n.a.
2		17,210	198,668	255,183	46,77	34,18	n.a.
**Total:**			**424,752**	**746,682**	**100,00**	**100,00**	

Chromatogram

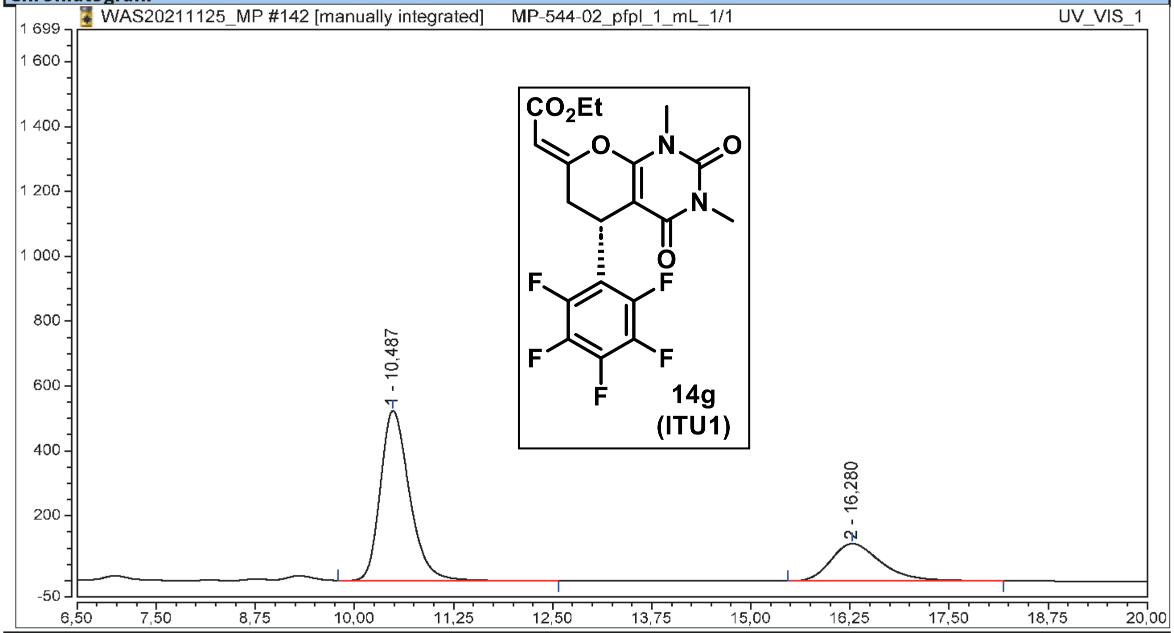

Integration Results

No.	Peak Name	Retention Time min	Area mAU*min	Height mAU	Relative Area %	Relative Height %	Amount n.a.
1		10,487	222,673	524,362	72,49	82,04	n.a.
2		16,280	84,503	114,754	27,51	17,96	n.a.
**Total:**			**307,176**	**639,115**	**100,00**	**100,00**	

Chromatogram

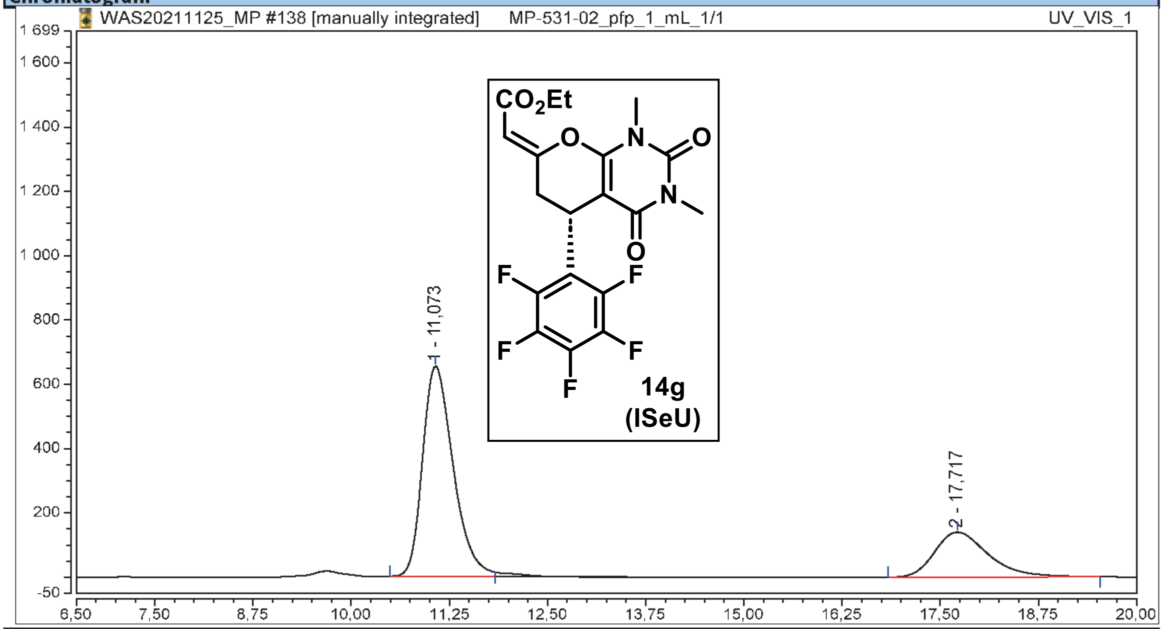

Integration Results

No.	Peak Name	Retention Time<br>min	Area<br>mAU*min	Height<br>mAU	Relative Area<br>%	Relative Height<br>%	Amount<br>n.a.
1		11,073	295,654	654,617	72,81	82,42	n.a.
2		17,717	110,407	139,627	27,19	17,58	n.a.
**Total:**			**406,061**	**794,244**	**100,00**	**100,00**	

Chromatogram

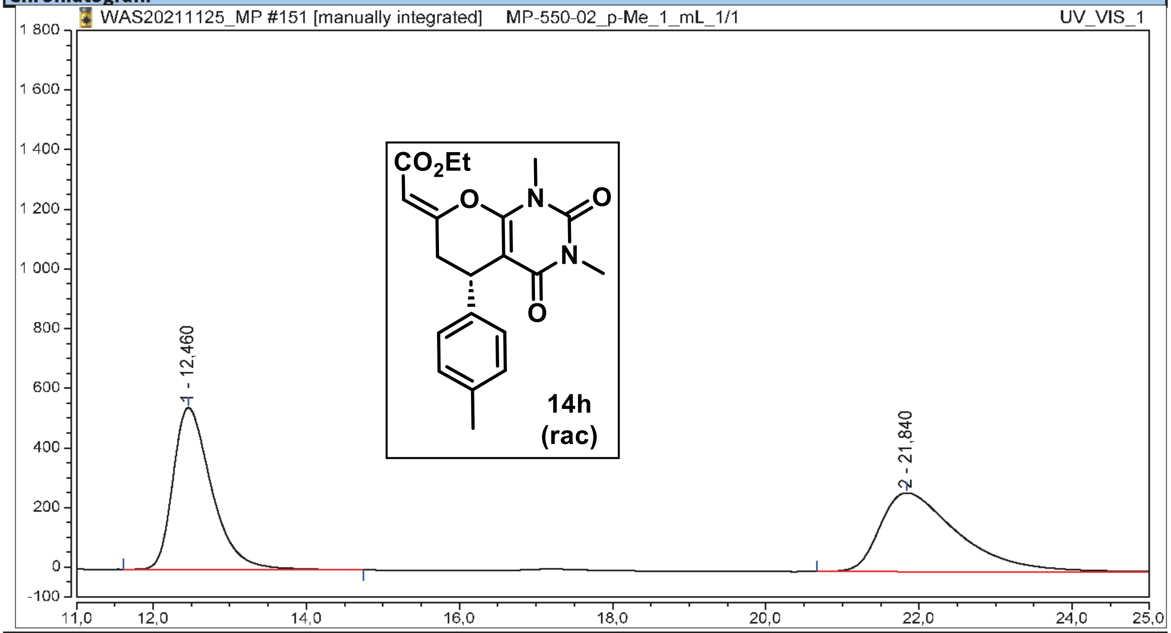

Integration Results

No.	Peak Name	Retention Time min	Area mAU*min	Height mAU	Relative Area %	Relative Height %	Amount n.a.
1		12,460	316,658	542,450	50,29	67,27	n.a.
2		21,840	313,064	263,957	49,71	32,73	n.a.
**Total:**			**629,722**	**806,407**	**100,00**	**100,00**	

Chromatogram

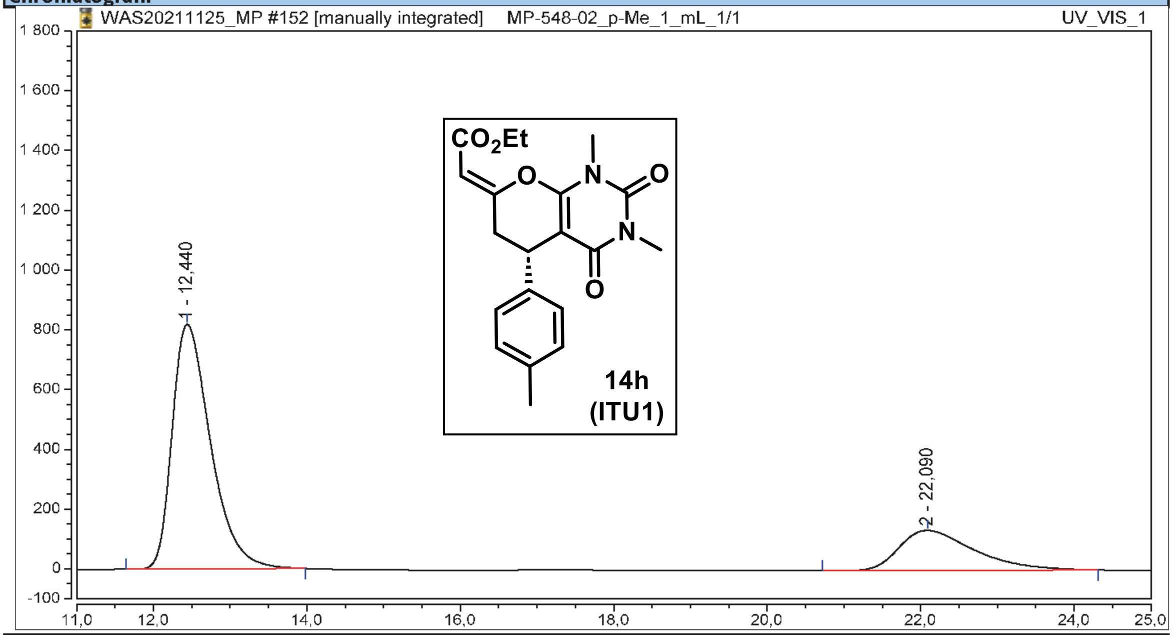

Integration Results

No.	Peak Name	Retention Time min	Area mAU*min	Height mAU	Relative Area %	Relative Height %	Amount n.a.
1		12,440	473,772	818,877	75,76	85,94	n.a.
2		22,090	151,581	133,950	24,24	14,06	n.a.
**Total:**			**625,353**	**952,827**	**100,00**	**100,00**	

Chromatogram

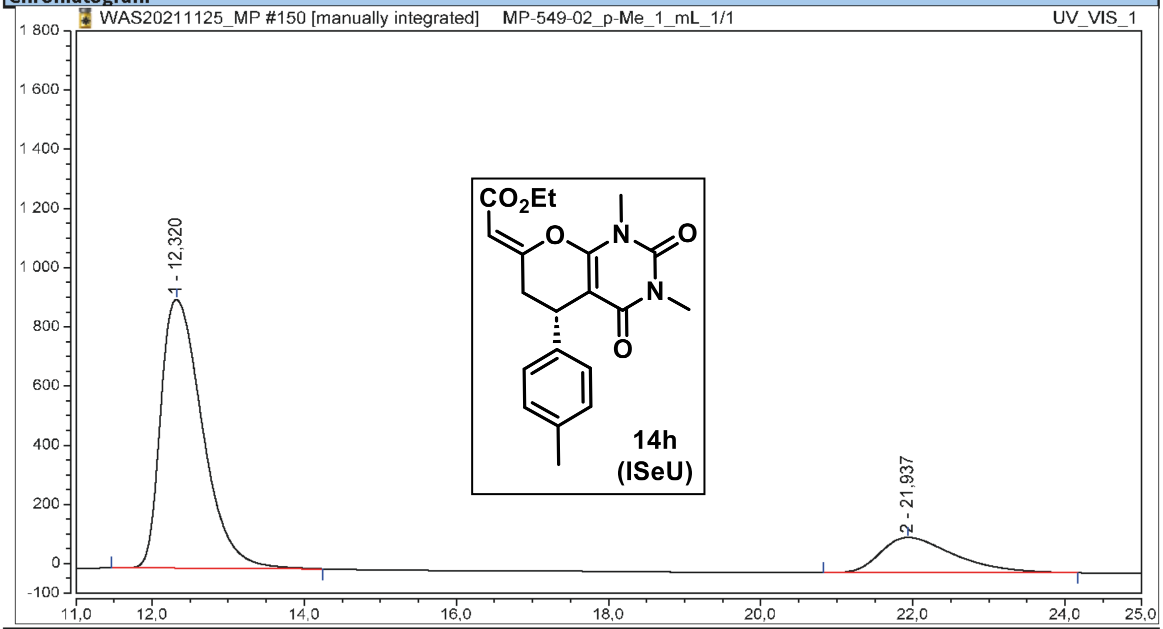

Integration Results

No.	Peak Name	Retention Time min	Area mAU*min	Height mAU	Relative Area %	Relative Height %	Amount n.a.
1		12,320	575,059	907,895	81,07	88,39	n.a.
2		21,937	134,261	119,226	18,93	11,61	n.a.
**Total:**			**709,320**	**1027,121**	**100,00**	**100,00**	

Chromatogram

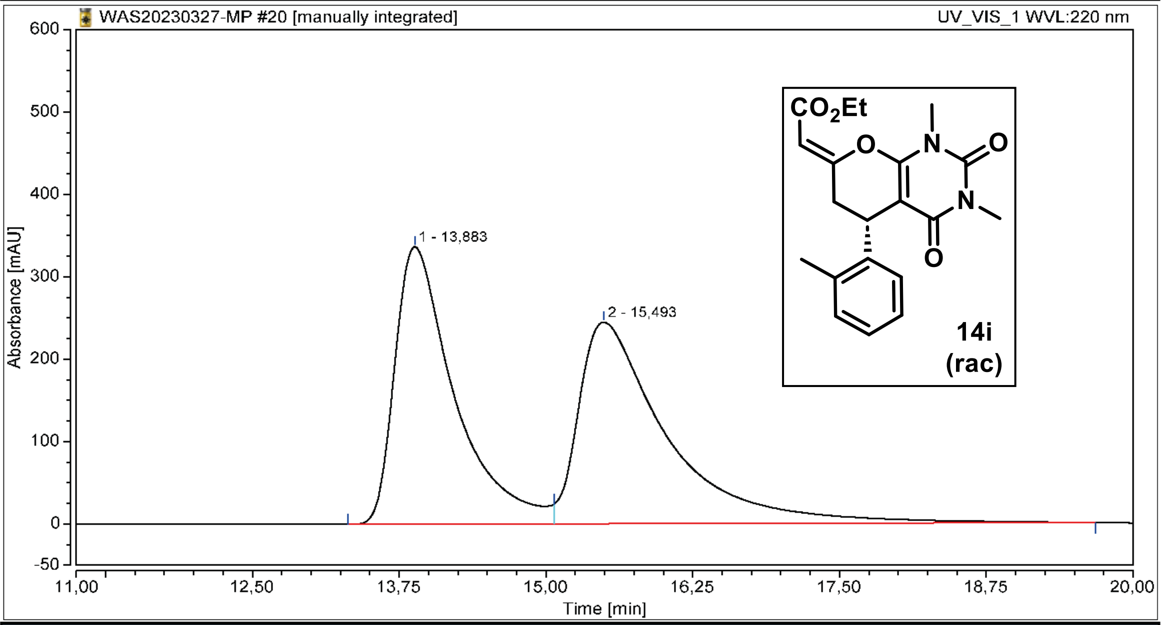

Integration Results

No.	Peak Name	Retention Time<br>min	Area<br>mAU*min	Height<br>mAU	Relative Area<br>%	Relative Height<br>%	Amount<br>n.a.
1		13,883	198,546	336,692	48,29	57,92	n.a.
2		15,493	212,576	244,587	51,71	42,08	n.a.
**Total:**			**411,121**	**581,279**	**100,00**	**100,00**	

Chromatogram

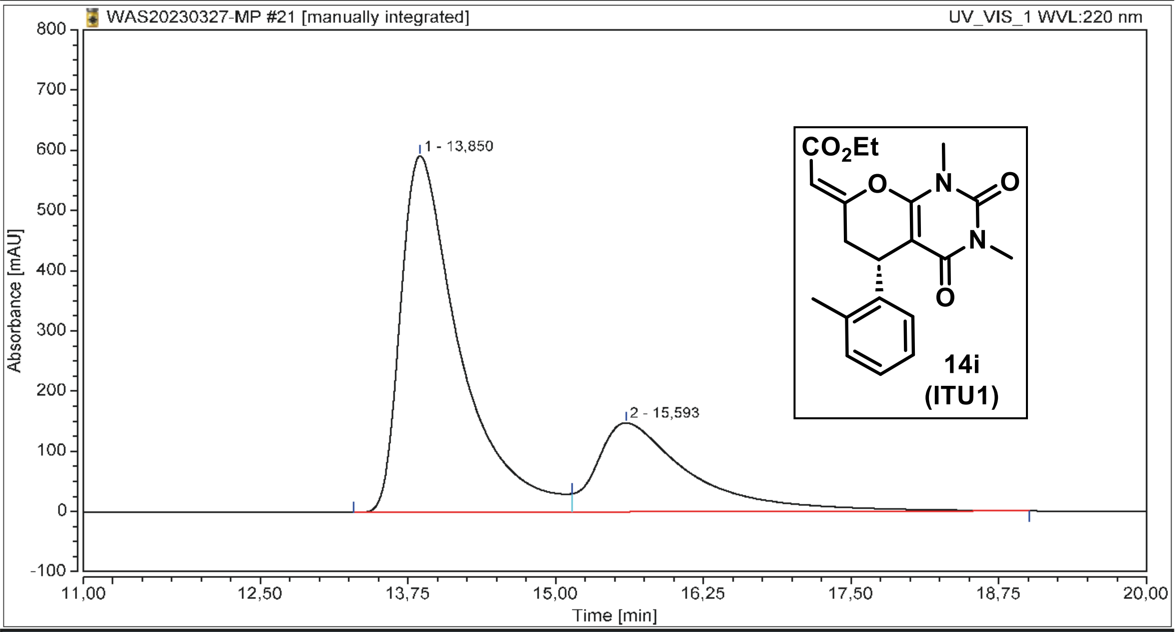

Integration Results

No.	Peak Name	Retention Time<br>min	Area<br>mAU*min	Height<br>mAU	Relative Area<br>%	Relative Height<br>%	Amount<br>n.a.
1		13,850	343,938	591,951	71,93	80,02	n.a.
2		15,593	134,236	147,762	28,07	19,98	n.a.
**Total:**			**478,174**	**739,714**	**100,00**	**100,00**	

Chromatogram

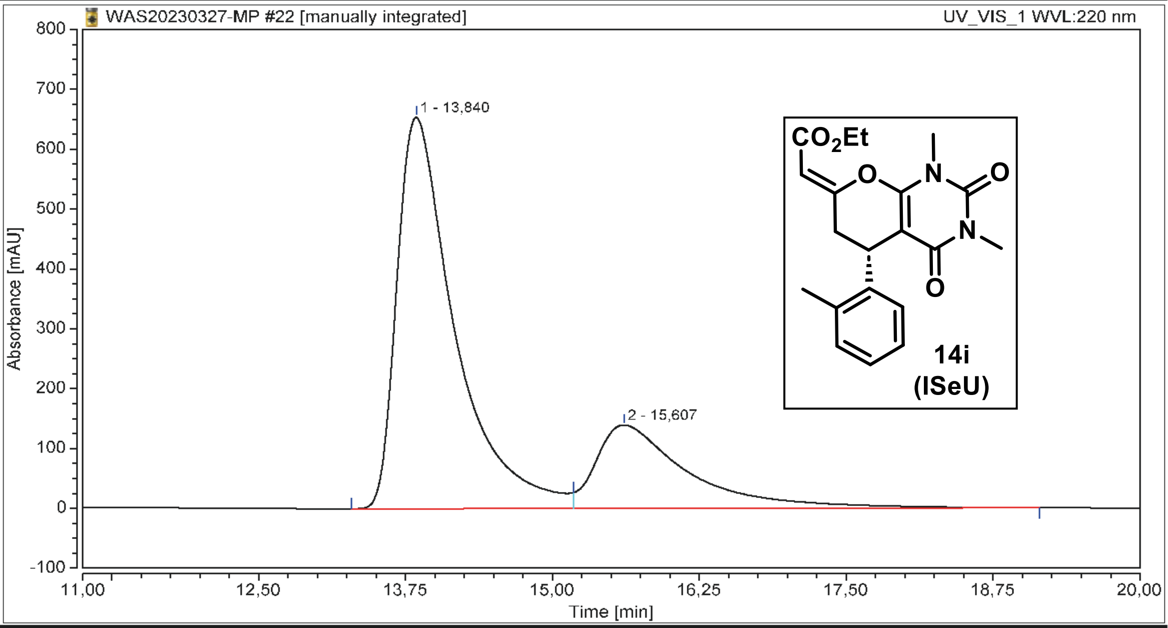

Integration Results

No.	Peak Name	Retention Time min	Area mAU*min	Height mAU	Relative Area %	Relative Height %	Amount n.a.
1		13,840	373,295	654,032	74,84	82,44	n.a.
2		15,607	125,497	139,335	25,16	17,56	n.a.
**Total:**			**498,792**	**793,367**	**100,00**	**100,00**	

Chromatogram

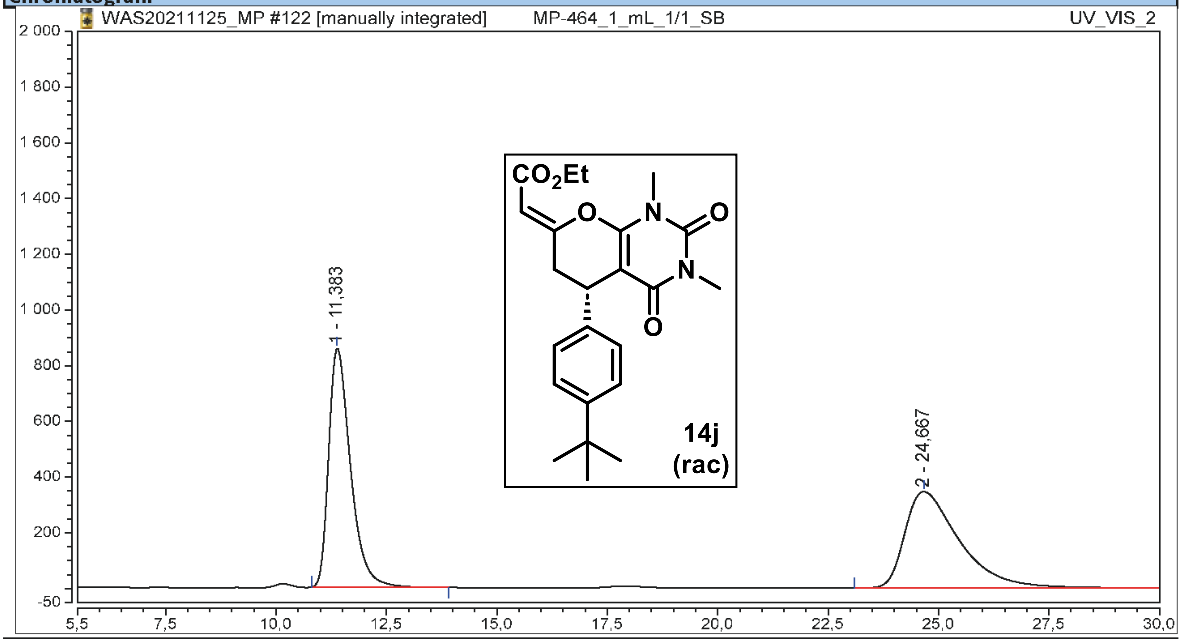

Integration Results

No.	Peak Name	Retention Time<br>min	Area<br>mAU*min	Height<br>mAU	Relative Area<br>%	Relative Height<br>%	Amount<br>n.a.
1		11,383	491,121	858,342	49,68	71,24	n.a.
2		24,667	497,402	346,491	50,32	28,76	n.a.
**Total:**			**988,523**	**1204,834**	**100,00**	**100,00**	

Chromatogram

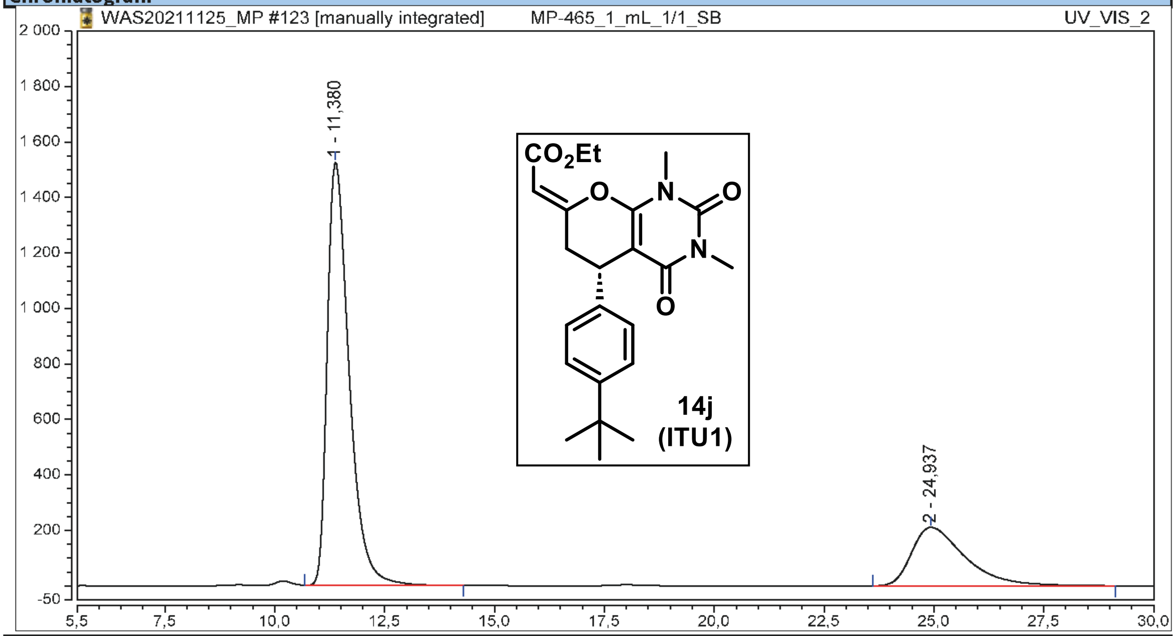

Integration Results

No.	Peak Name	Retention Time<br>min	Area<br>mAU*min	Height<br>mAU	Relative Area<br>%	Relative Height<br>%	Amount<br>n.a.
1		11,380	878,029	1525,959	74,66	87,85	n.a.
2		24,937	298,076	210,977	25,34	12,15	n.a.
**Total:**			**1176,105**	**1736,935**	**100,00**	**100,00**	

Chromatogram

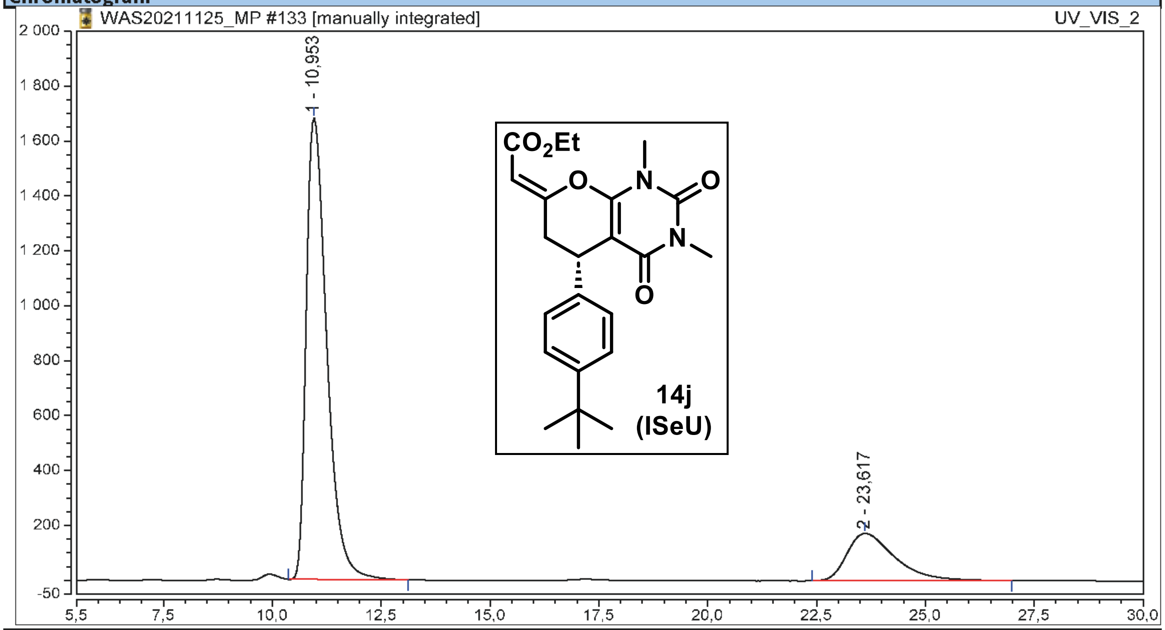

Integration Results

No.	Peak Name	Retention Time min	Area mAU*min	Height mAU	Relative Area %	Relative Height %	Amount n.a.
1		10,953	918,351	1681,763	80,69	90,70	n.a.
2		23,617	219,743	172,386	19,31	9,30	n.a.
**Total:**			**1138,094**	**1854,149**	**100,00**	**100,00**	

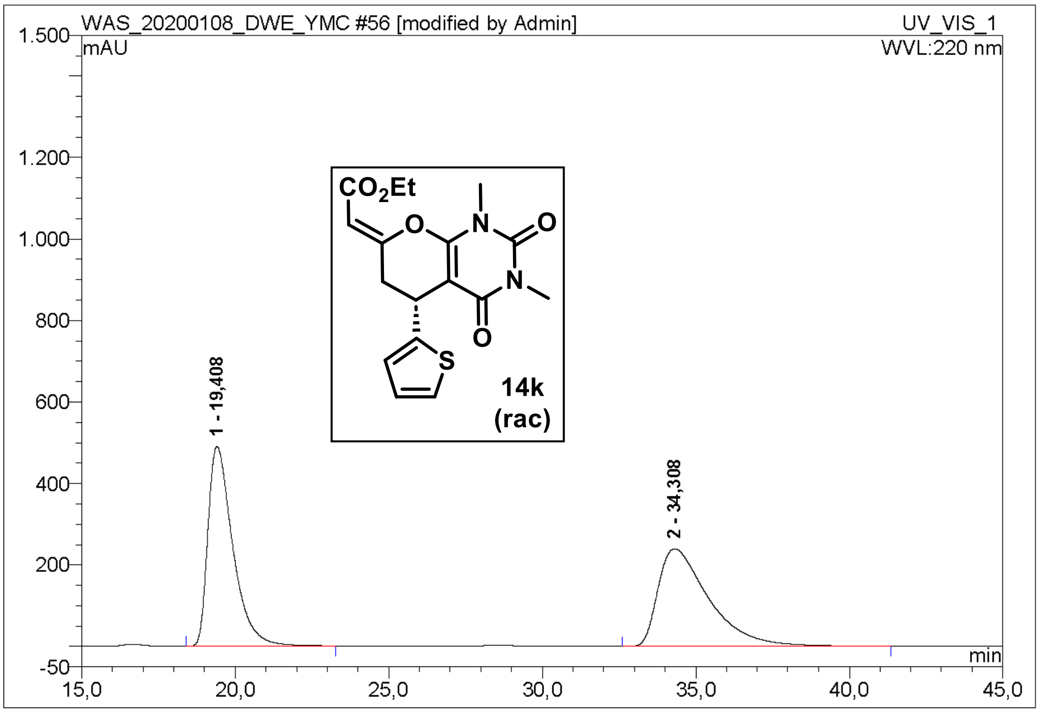

No.	Ret.Time min	Peak Name	Height mAU	Area mAU*min	Rel.Area %	Amount	Type
1	19,41	n.a.	490,592	462,055	50,24	n.a.	BMB*
2	34,31	n.a.	239,654	457,573	49,76	n.a.	BMB*
Total:			730,246	919,628	100,00	0,000	

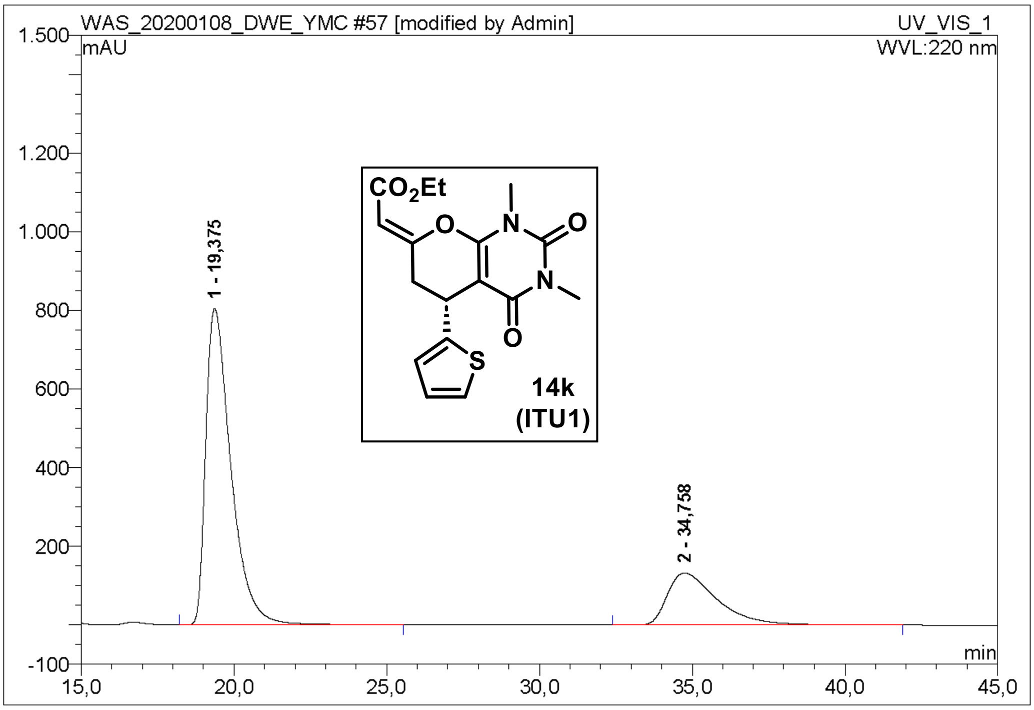

No.	Ret.Time min	Peak Name	Height mAU	Area mAU*min	Rel.Area %	Amount	Type
1	19,38	n.a.	805,030	769,410	75,48	n.a.	BMB*
2	34,76	n.a.	132,446	249,998	24,52	n.a.	BMB*
Total:			937,477	1019,409	100,00	0,000	

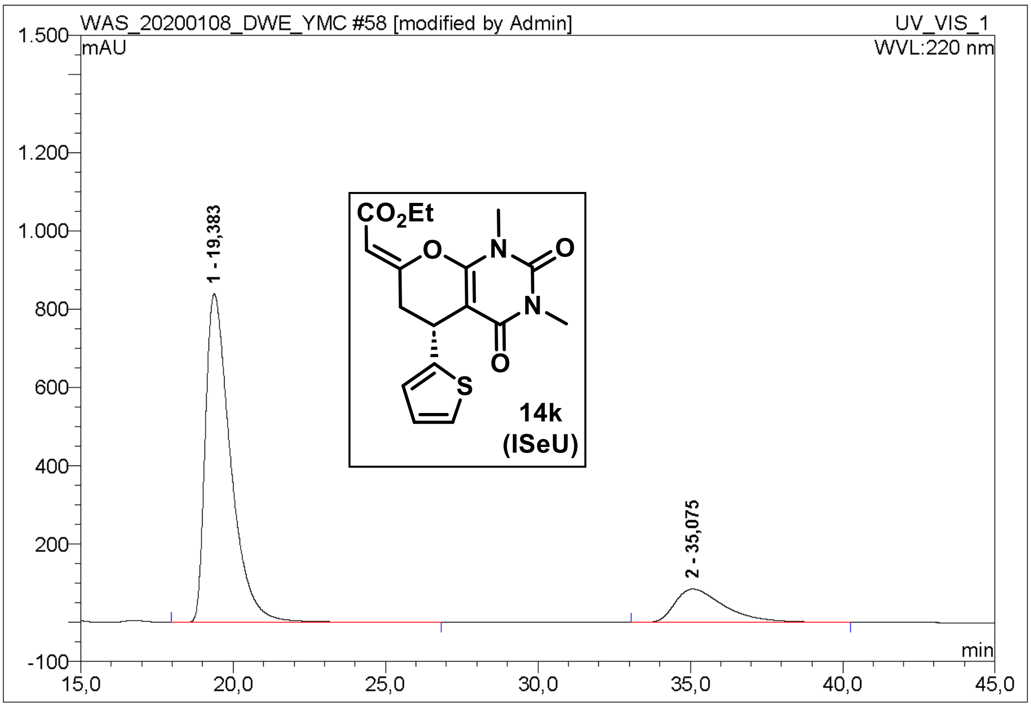

No.	Ret.Time min	Peak Name	Height mAU	Area mAU*min	Rel.Area %	Amount	Type
1	19,38	n.a.	839,369	805,889	83,26	n.a.	BMB*
2	35,08	n.a.	85,543	162,008	16,74	n.a.	BMB*
Total:			924,912	967,897	100,00	0,000	

Chromatogram

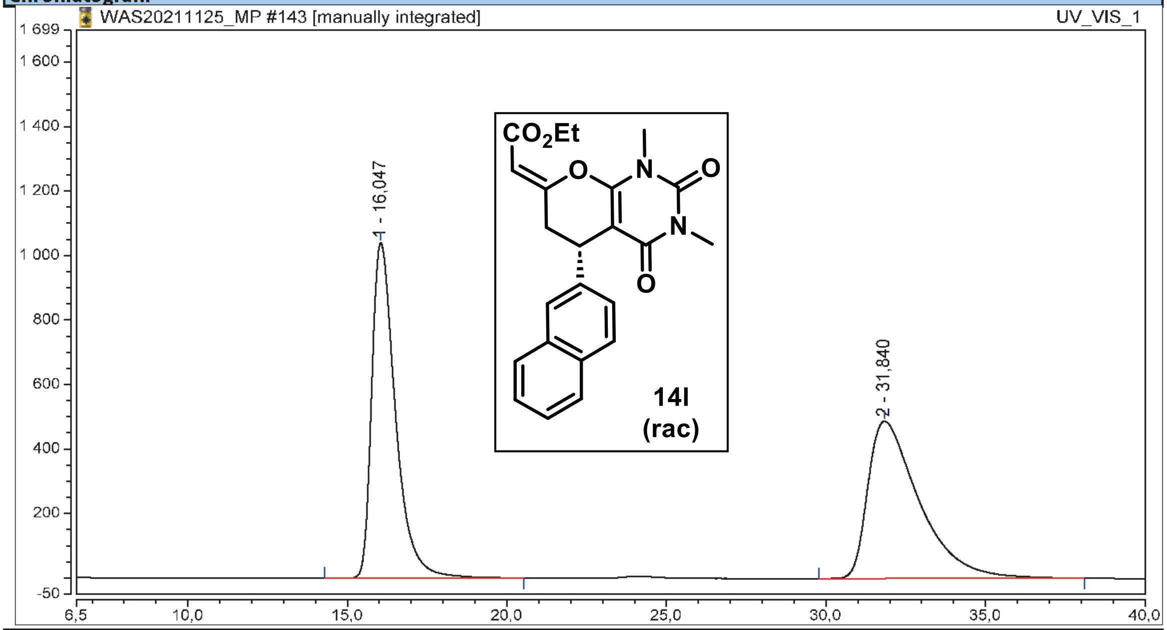

Integration Results

No.	Peak Name	Retention Time<br>min	Area<br>mAU*min	Height<br>mAU	Relative Area<br>%	Relative Height<br>%	Amount<br>n.a.
1		16,047	896,269	1041,040	49,62	68,10	n.a.
2		31,840	909,943	487,651	50,38	31,90	n.a.
**Total:**			**1806,212**	**1528,691**	**100,00**	**100,00**	

Chromatogram

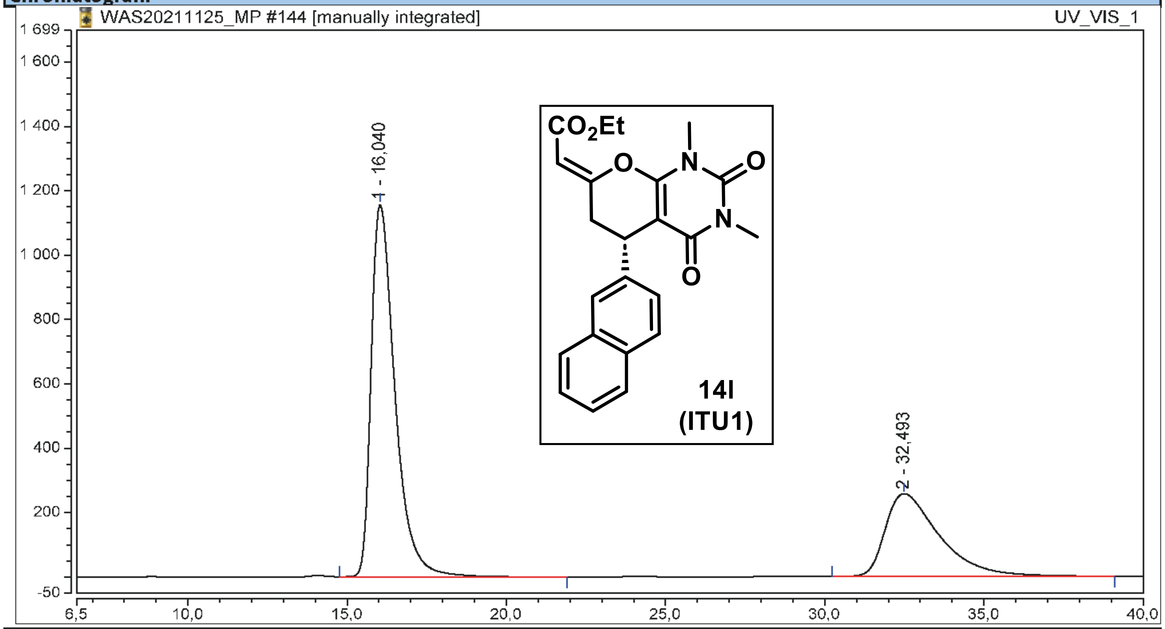

Integration Results

No.	Peak Name	Retention Time<br>min	Area<br>mAU*min	Height<br>mAU	Relative Area<br>%	Relative Height<br>%	Amount<br>n.a.
1		16,040	1004,762	1157,440	66,44	81,84	n.a.
2		32,493	507,450	256,886	33,56	18,16	n.a.
**Total:**			**1512,212**	**1414,327**	**100,00**	**100,00**	

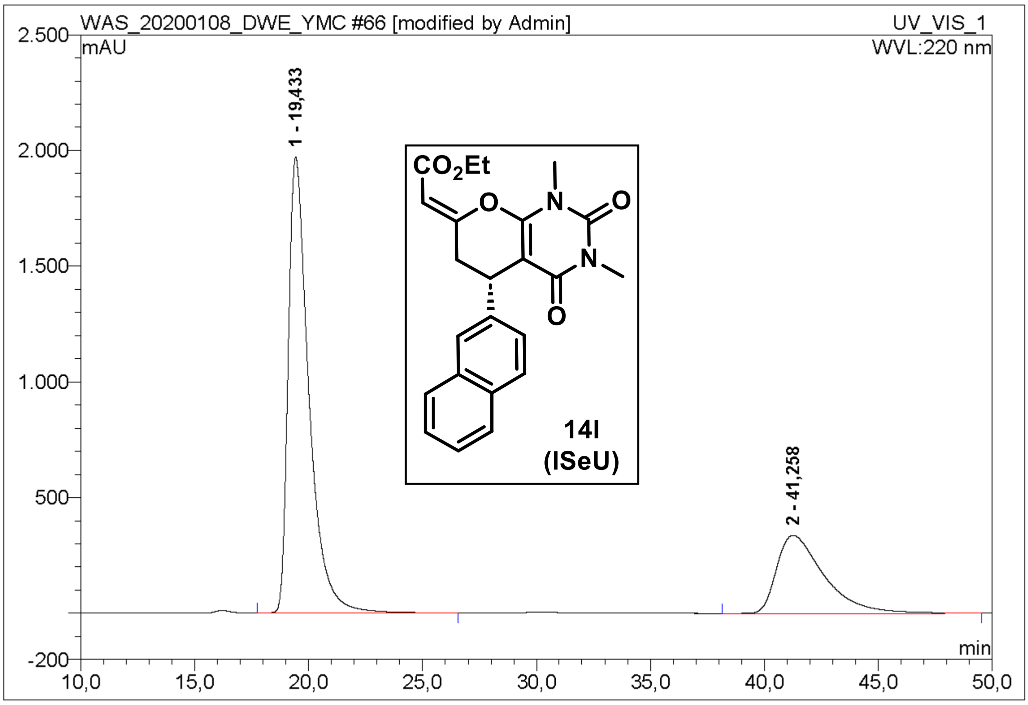

No.	Ret.Time min	Peak Name	Height mAU	Area mAU*min	Rel.Area %	Amount	Type
1	19,43	n.a.	1972,086	2073,070	71,91	n.a.	BMB*
2	41,26	n.a.	337,508	809,742	28,09	n.a.	BMB*
Total:			2309,594	2882,813	100,00	0,000	

Chromatogram

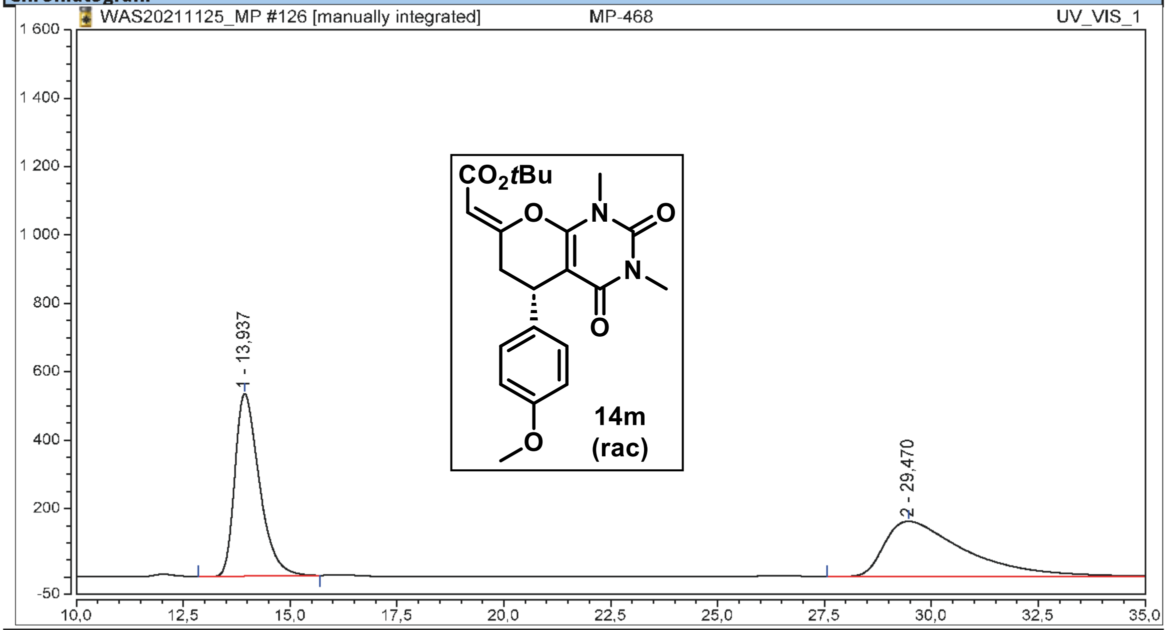

Integration Results

No.	Peak Name	Retention Time<br>min	Area<br>mAU*min	Height<br>mAU	Relative Area<br>%	Relative Height<br>%	Amount<br>n.a.
1		13,937	353,553	534,319	50,39	76,86	n.a.
2		29,470	348,123	160,827	49,61	23,14	n.a.
**Total:**			**701,676**	**695,146**	**100,00**	**100,00**	

Chromatogram

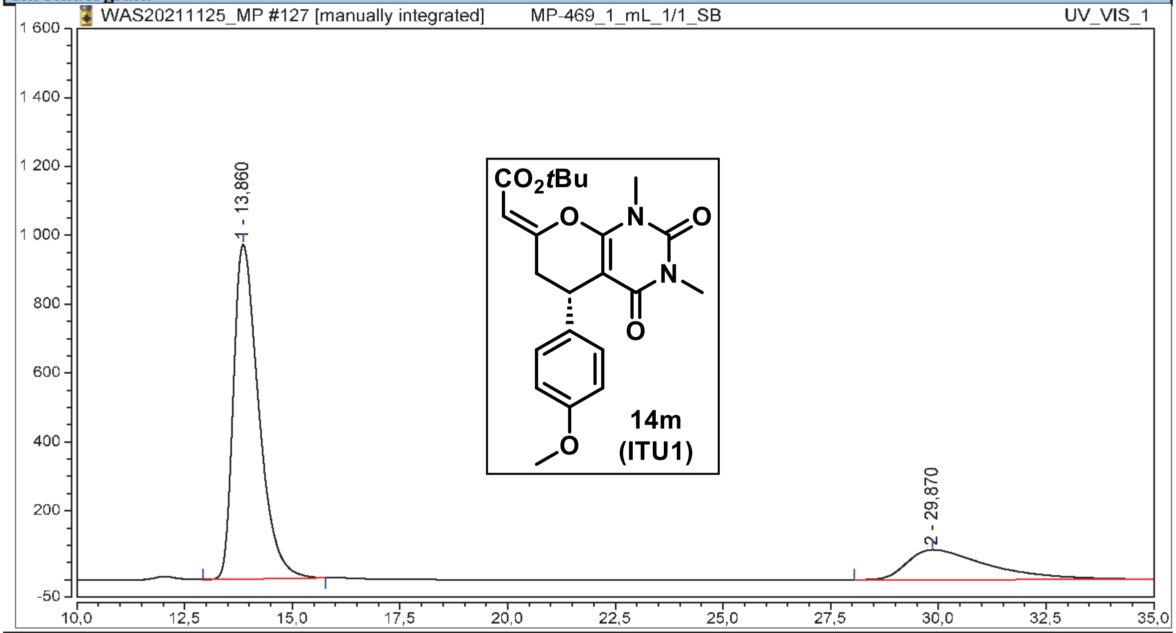

Integration Results

No.	Peak Name	Retention Time<br>min	Area<br>mAU*min	Height<br>mAU	Relative Area<br>%	Relative Height<br>%	Amount<br>n.a.
1		13,860	658,266	971,673	77,59	91,80	n.a.
2		29,870	190,147	86,747	22,41	8,20	n.a.
**Total:**			**848,413**	**1058,421**	**100,00**	**100,00**	

Chromatogram

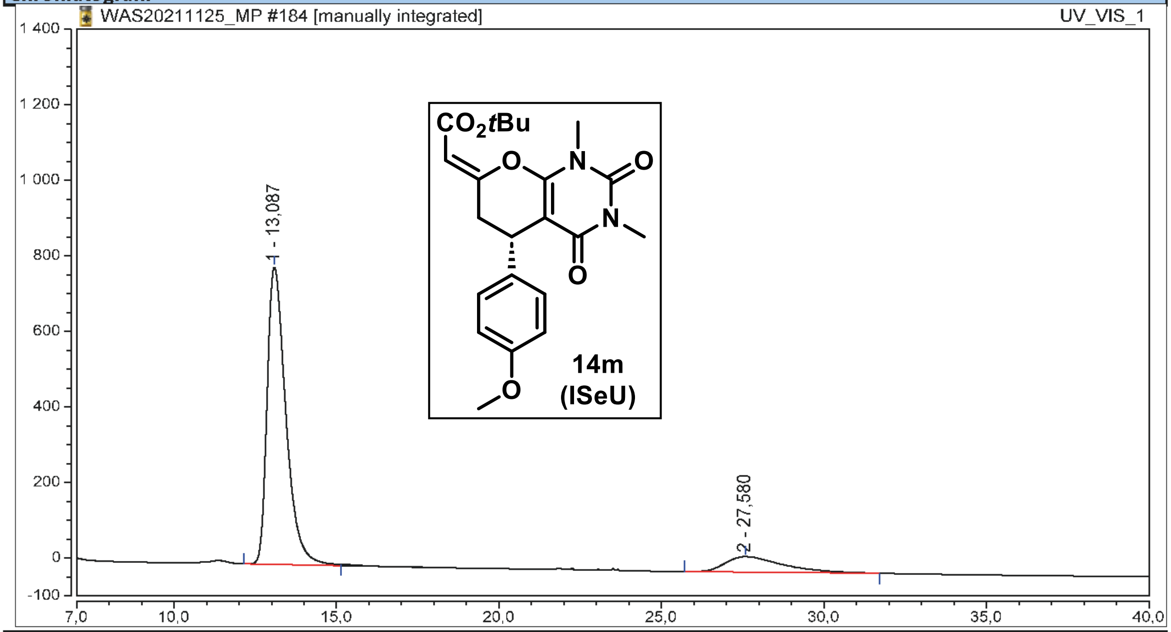

Integration Results

No.	Peak Name	Retention Time min	Area mAU*min	Height mAU	Relative Area %	Relative Height %	Amount n.a.
1		13,087	525,738	789,212	86,20	95,07	n.a.
2		27,580	84,147	40,917	13,80	4,93	n.a.
**Total:**			**609,885**	**830,129**	**100,00**	**100,00**	

Chromatogram

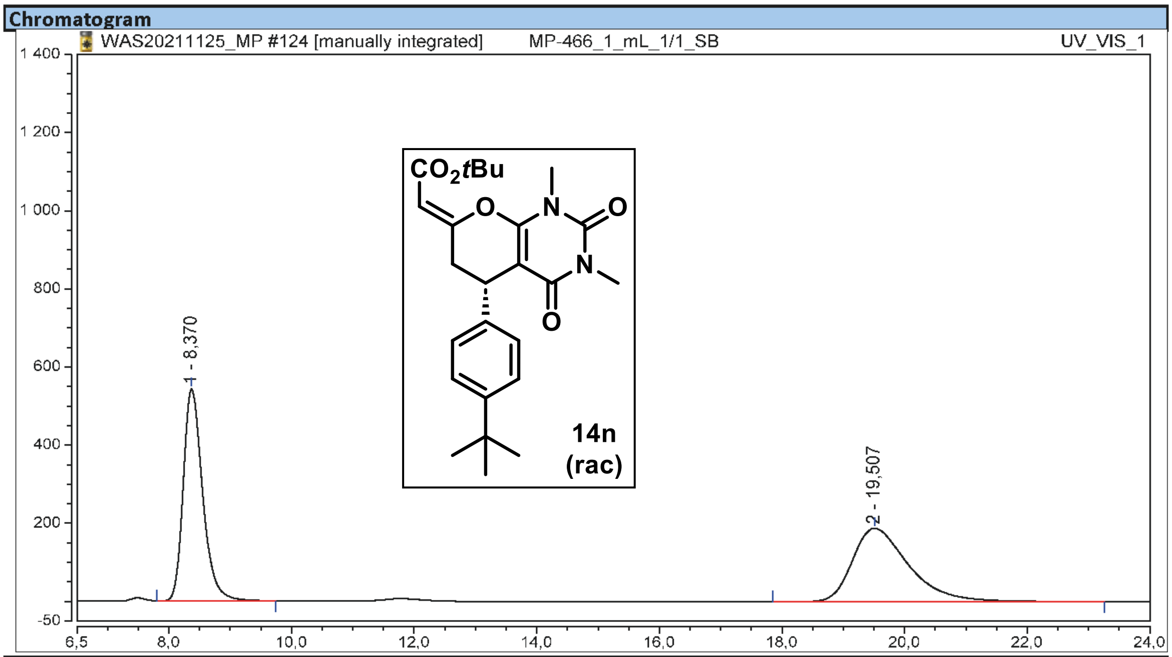

Integration Results

No.	Peak Name	Retention Time min	Area mAU*min	Height mAU	Relative Area %	Relative Height %	Amount n.a.
1		8,370	199,010	542,808	49,84	74,28	n.a.
2		19,507	200,321	187,956	50,16	25,72	n.a.
**Total:**			**399,331**	**730,764**	**100,00**	**100,00**	

Chromatogram

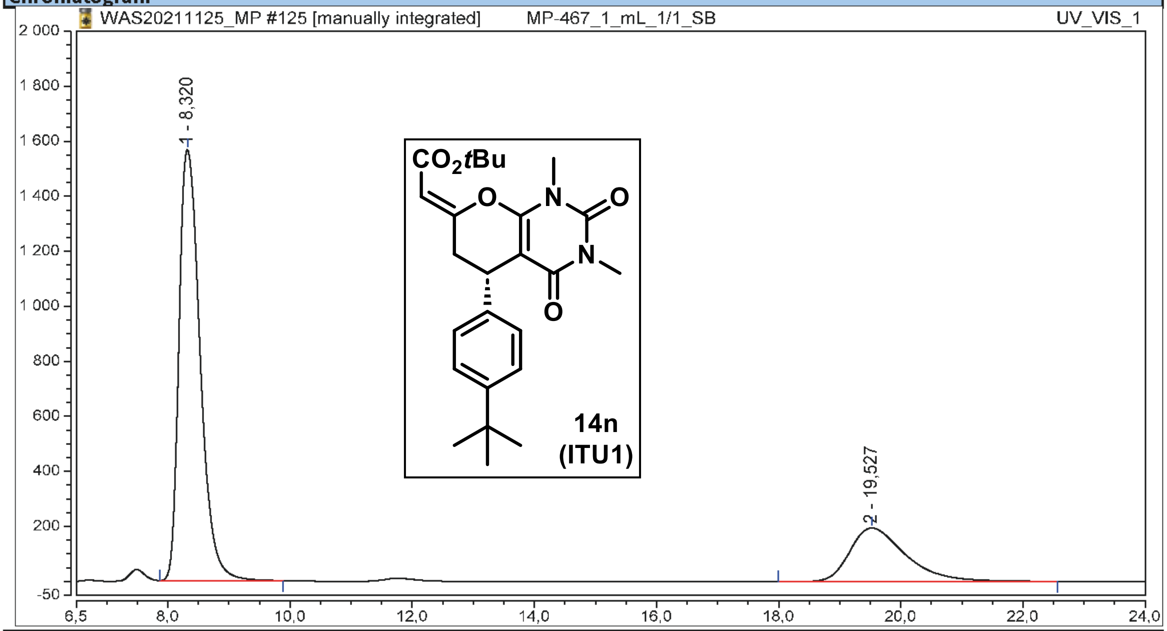

Integration Results

No.	Peak Name	Retention Time min	Area mAU*min	Height mAU	Relative Area %	Relative Height %	Amount n.a.
1		8,320	627,050	1568,205	75,23	88,95	n.a.
2		19,527	206,438	194,764	24,77	11,05	n.a.
**Total:**			**833,488**	**1762,969**	**100,00**	**100,00**	

Chromatogram

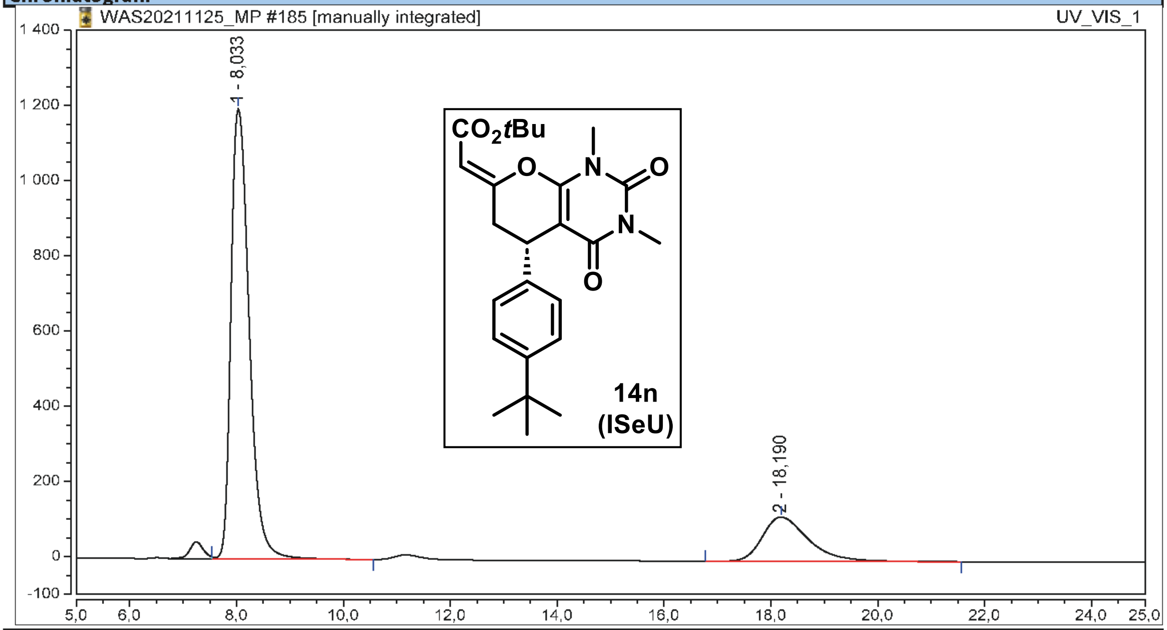

Integration Results

No.	Peak Name	Retention Time min	Area mAU*min	Height mAU	Relative Area %	Relative Height %	Amount n.a.
1		8,033	473,199	1198,591	80,10	91,06	n.a.
2		18,190	117,536	117,677	19,90	8,94	n.a.
**Total:**			**590,735**	**1316,268**	**100,00**	**100,00**	

Chromatogram

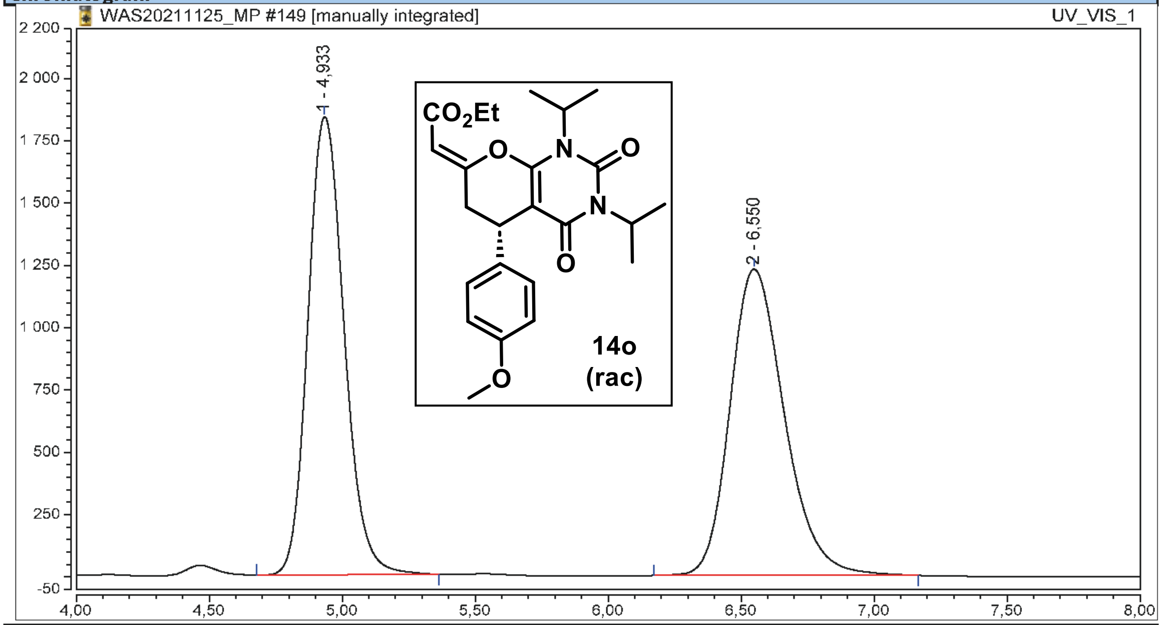

Integration Results

No.	Peak Name	Retention Time min	Area mAU*min	Height mAU	Relative Area %	Relative Height %	Amount n.a.
1		4,933	290,497	1838,790	49,90	59,93	n.a.
2		6,550	291,611	1229,517	50,10	40,07	n.a.
**Total:**			**582,109**	**3068,307**	**100,00**	**100,00**	

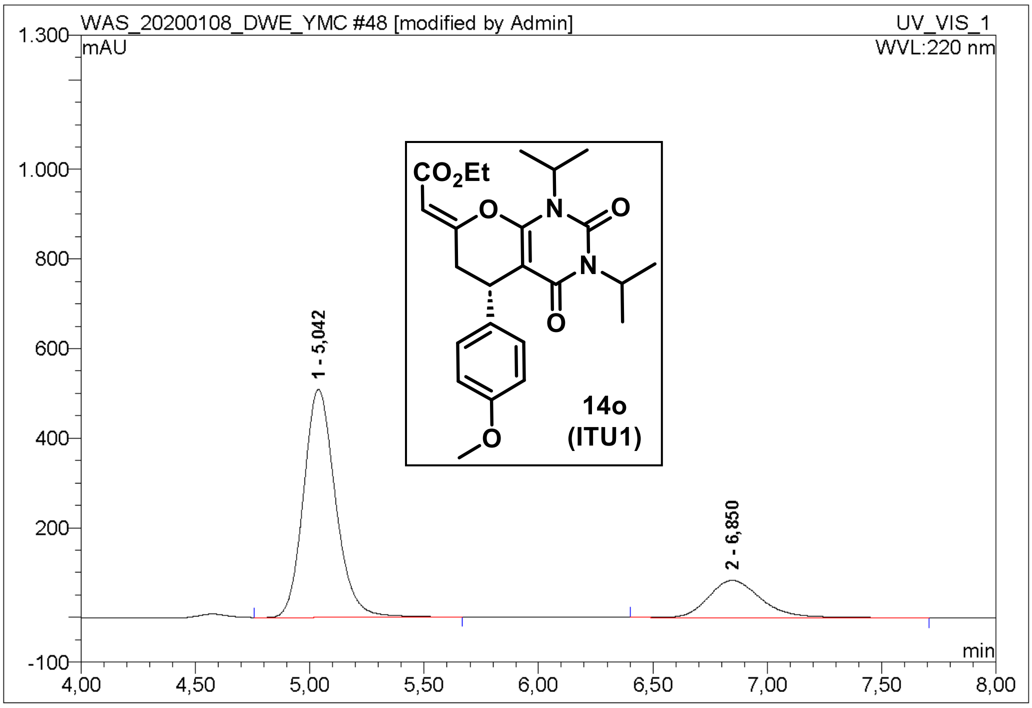

No.	Ret.Time min	Peak Name	Height mAU	Area mAU*min	Rel.Area %	Amount	Type
1	5,04	n.a.	508,066	81,725	78,98	n.a.	BMB*
2	6,85	n.a.	83,033	21,753	21,02	n.a.	BMB*
**Total:**			591,098	103,478	100,00	0,000	

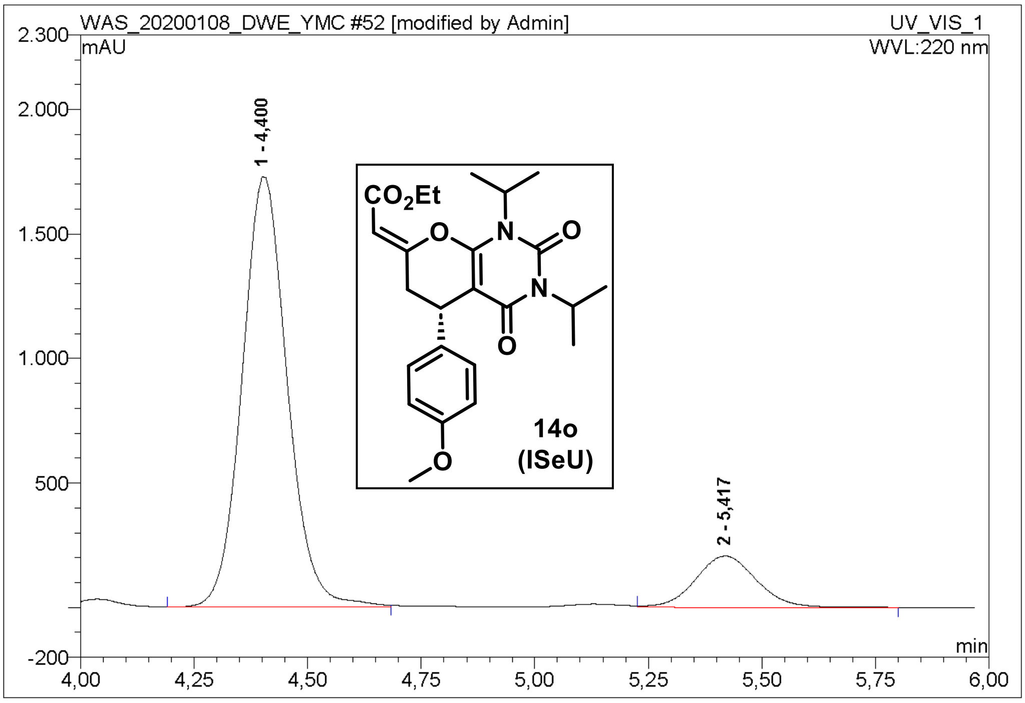

No.	Ret.Time min	Peak Name	Height mAU	Area mAU*min	Rel.Area %	Amount	Type
1	4,40	n.a.	1727,222	198,481	86,24	n.a.	BM *
2	5,42	n.a.	205,729	31,678	13,76	n.a.	MB*
Total:			1932,951	230,159	100,00	0,000	

Chromatogram

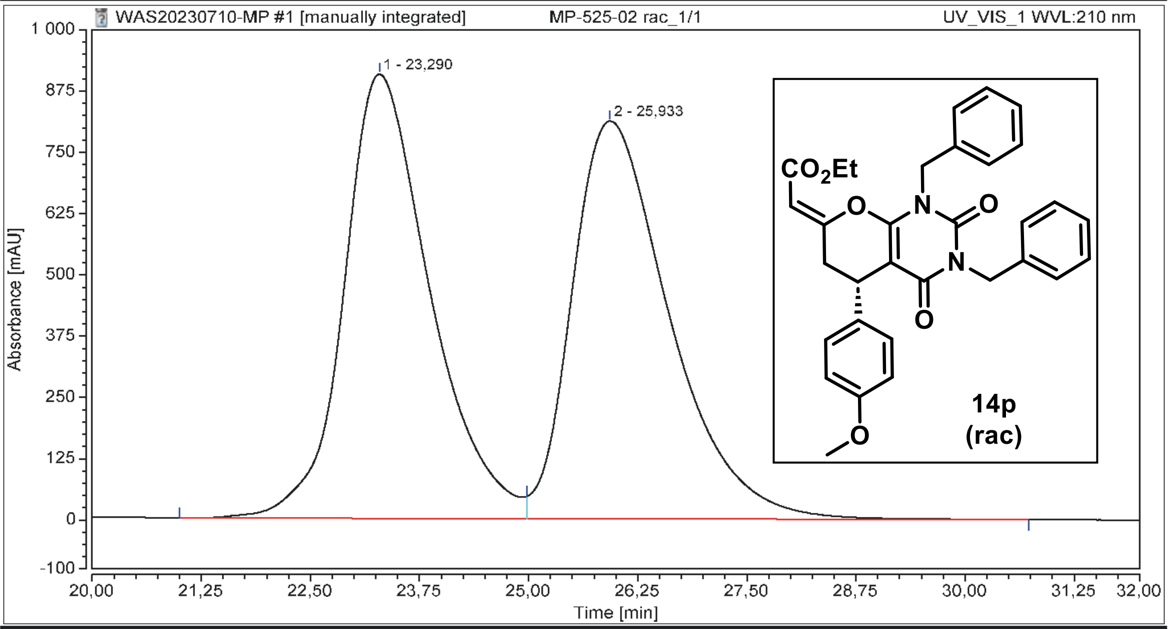

Integration Results

No.	Peak Name	Retention Time<br>min	Area<br>mAU*min	Height<br>mAU	Relative Area<br>%	Relative Height<br>%	Amount<br>n.a.
1		23,290	1035,080	905,983	49,76	52,75	n.a.
2		25,933	1045,175	811,453	50,24	47,25	n.a.
**Total:**			**2080,254**	**1717,436**	**100,00**	**100,00**	

Chromatogram

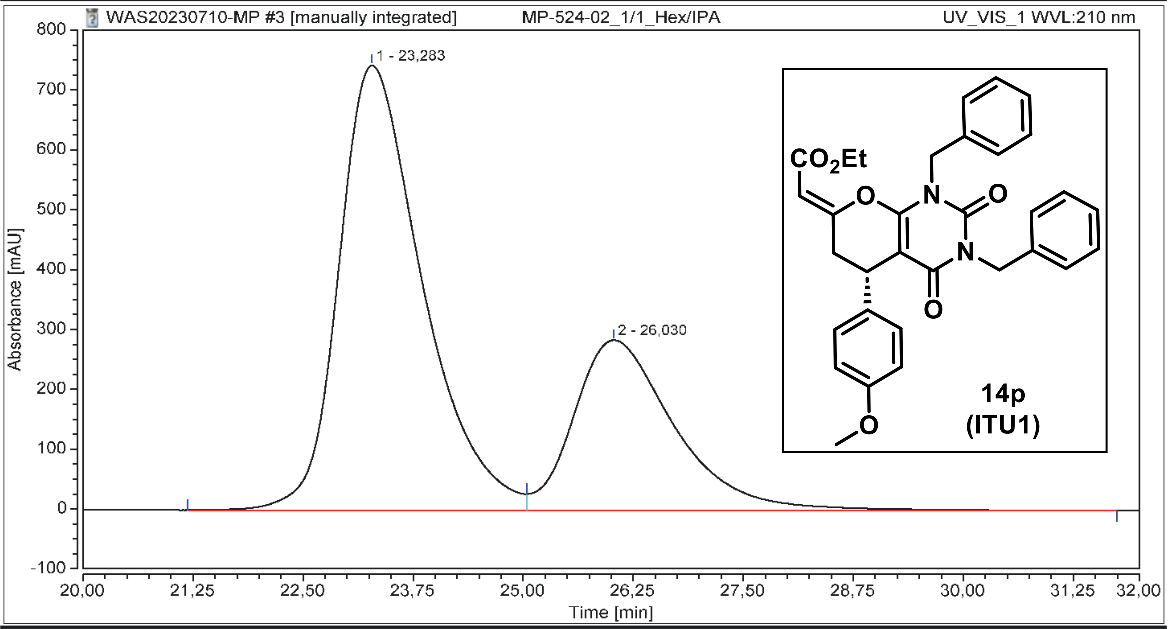

Integration Results

No.	Peak Name	Retention Time<br>min	Area<br>mAU*min	Height<br>mAU	Relative Area<br>%	Relative Height<br>%	Amount<br>n.a.
1		23,283	816,814	743,163	68,47	72,33	n.a.
2		26,030	376,150	284,241	31,53	27,67	n.a.
**Total:**			**1192,964**	**1027,404**	**100,00**	**100,00**	

Chromatogram

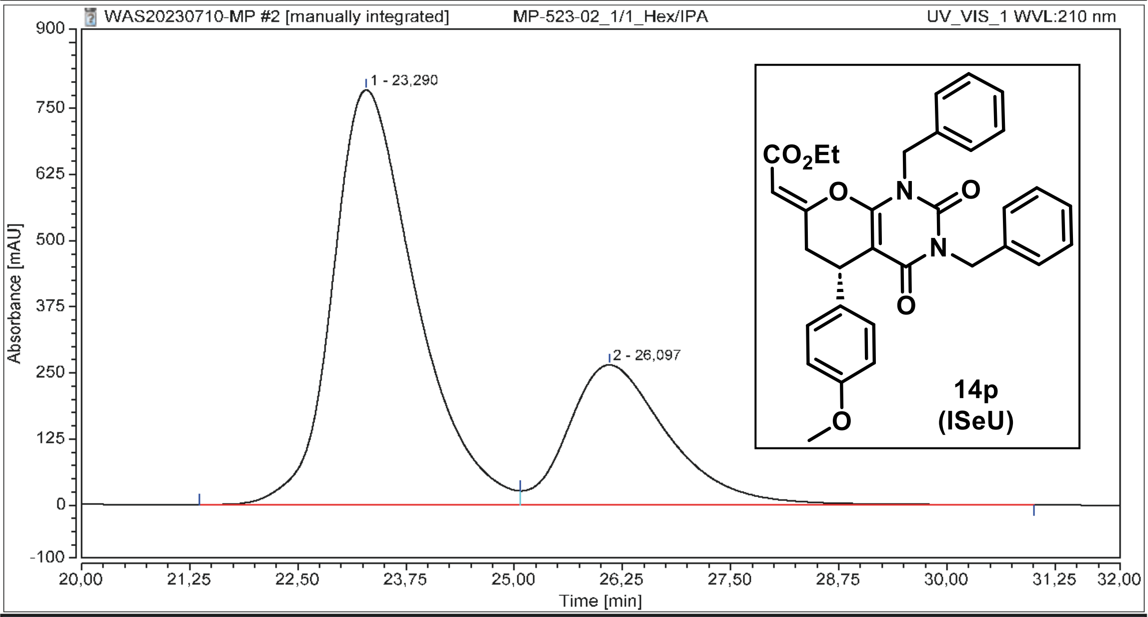

Integration Results

No.	Peak Name	Retention Time<br>min	Area<br>mAU*min	Height<br>mAU	Relative Area<br>%	Relative Height<br>%	Amount<br>n.a.
1		23,290	875,226	784,602	71,26	74,79	n.a.
2		26,097	352,922	264,529	28,74	25,21	n.a.
**Total:**			**1228,148**	**1049,130**	**100,00**	**100,00**	

Chromatogram

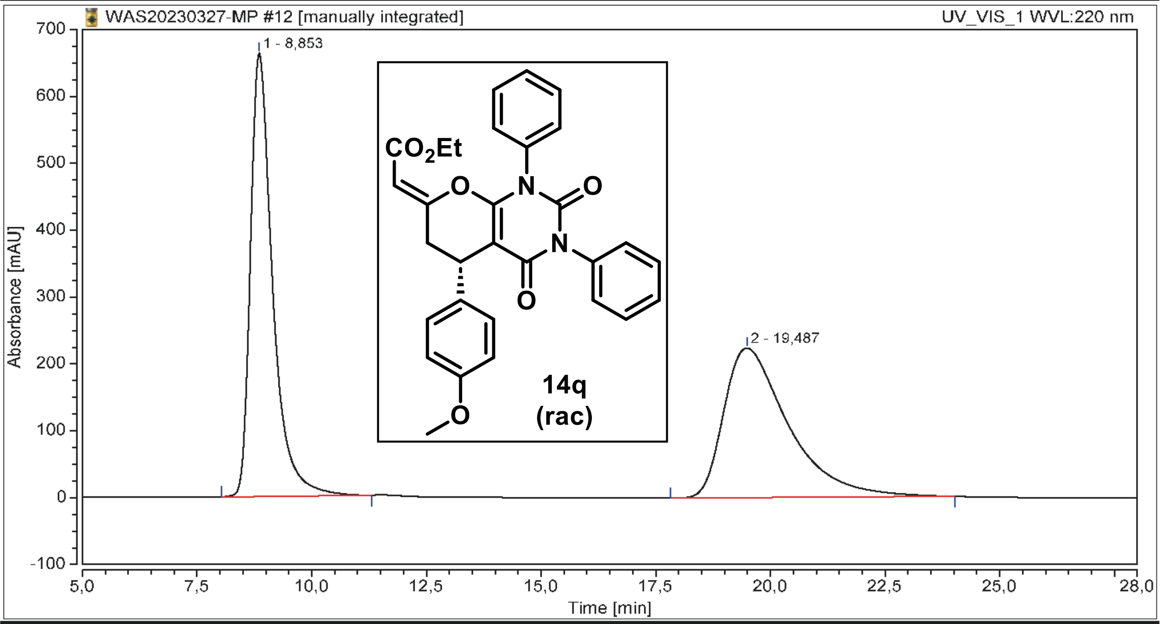

Integration Results

No.	Peak Name	Retention Time<br>min	Area<br>mAU*min	Height<br>mAU	Relative Area<br>%	Relative Height<br>%	Amount<br>n.a.
1		8,853	371,699	663,080	50,48	74,78	n.a.
2		19,487	364,603	223,675	49,52	25,22	n.a.
**Total:**			**736,303**	**886,755**	**100,00**	**100,00**	

Chromatogram

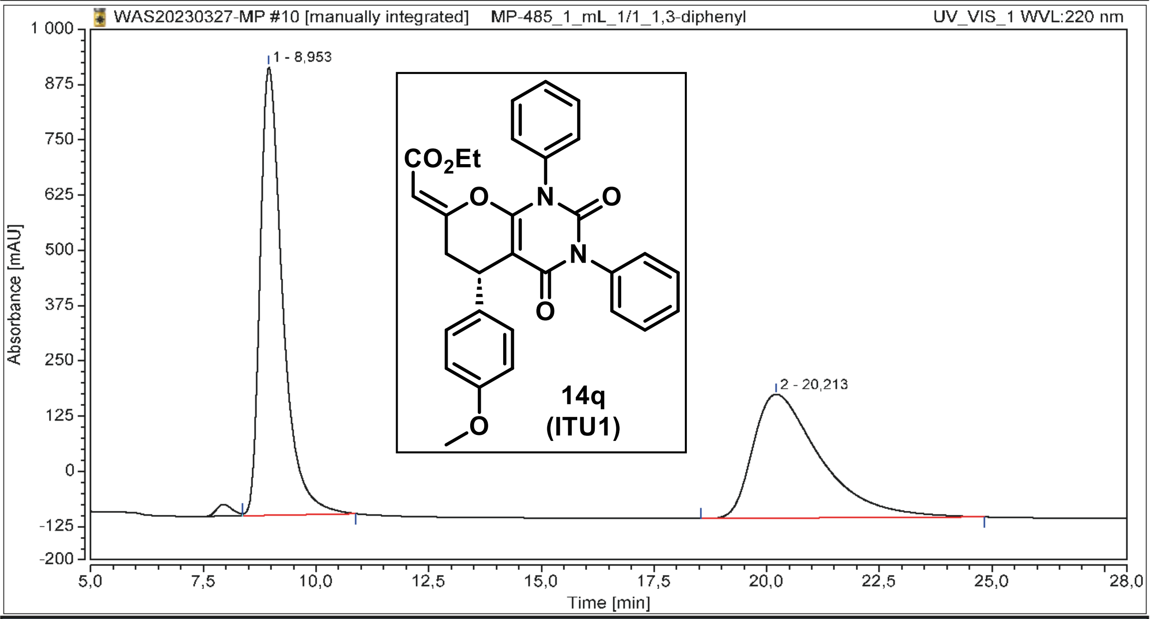

Integration Results

No.	Peak Name	Retention Time<br>min	Area<br>mAU*min	Height<br>mAU	Relative Area<br>%	Relative Height<br>%	Amount<br>n.a.
1		8,953	586,957	1014,738	54,75	78,37	n.a.
2		20,213	485,051	280,113	45,25	21,63	n.a.
**Total:**			**1072,007**	**1294,851**	**100,00**	**100,00**	

Chromatogram

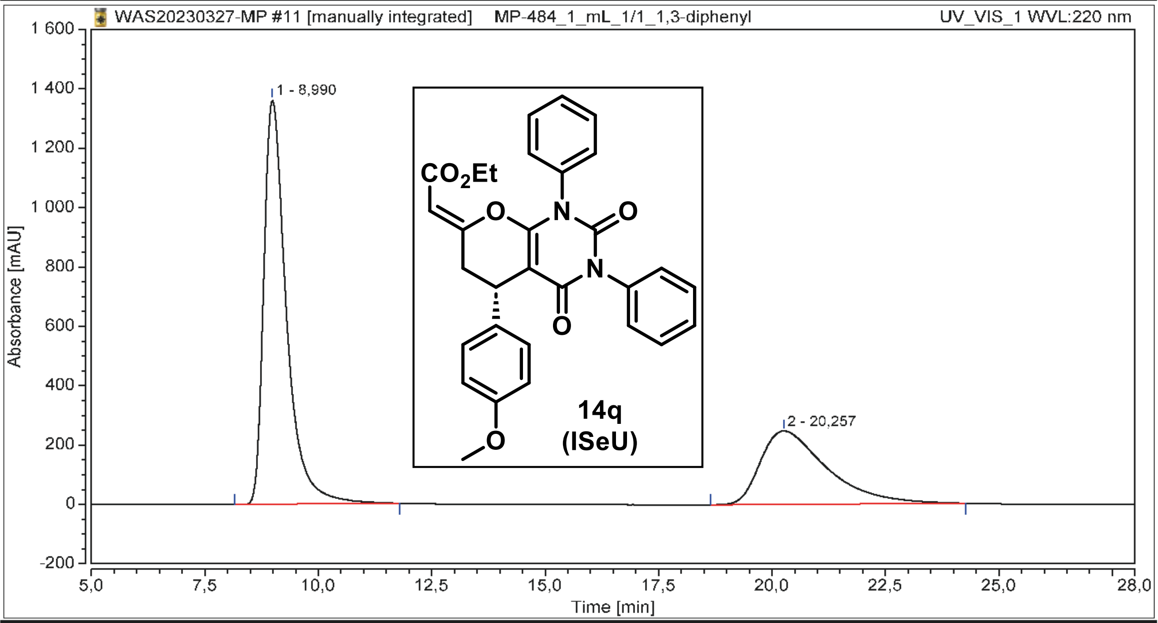

Integration Results

No.	Peak Name	Retention Time min	Area mAU*min	Height mAU	Relative Area %	Relative Height %	Amount n.a.
1		8,990	787,462	1362,752	65,22	84,64	n.a.
2		20,257	419,916	247,228	34,78	15,36	n.a.
**Total:**			**1207,377**	**1609,980**	**100,00**	**100,00**	

Chromatogram

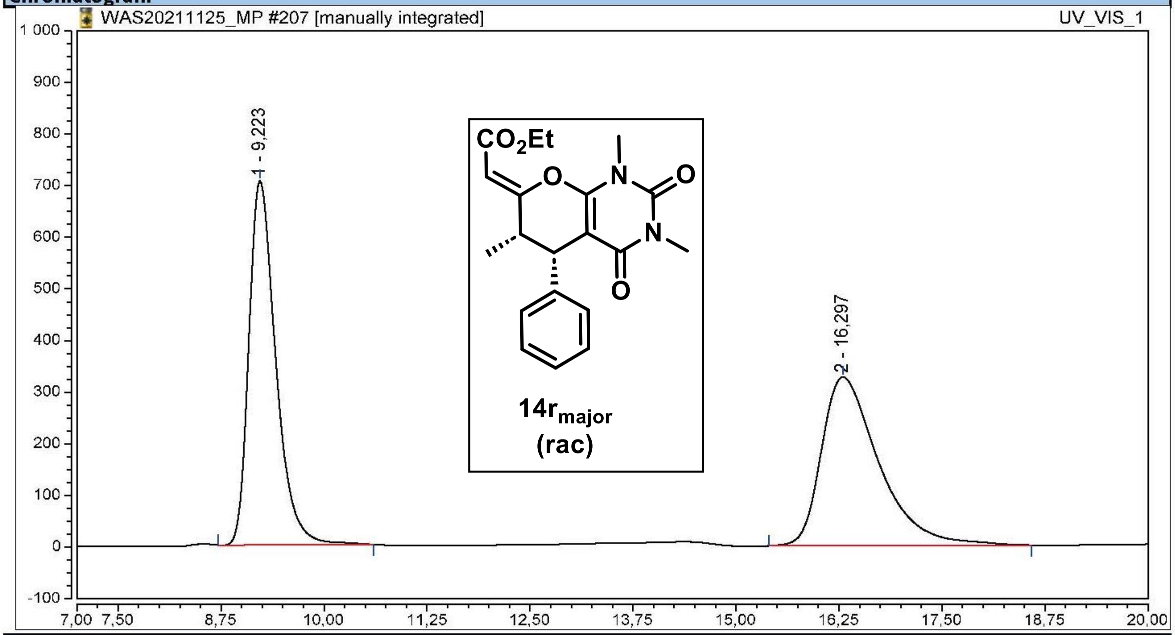

Integration Results

No.	Peak Name	Retention Time<br>min	Area<br>mAU*min	Height<br>mAU	Relative Area<br>%	Relative Height<br>%	Amount<br>n.a.
1		9,223	266,937	706,076	50,34	68,37	n.a.
2		16,297	263,283	326,627	49,66	31,63	n.a.
**Total:**			**530,220**	**1032,702**	**100,00**	**100,00**	

Chromatogram

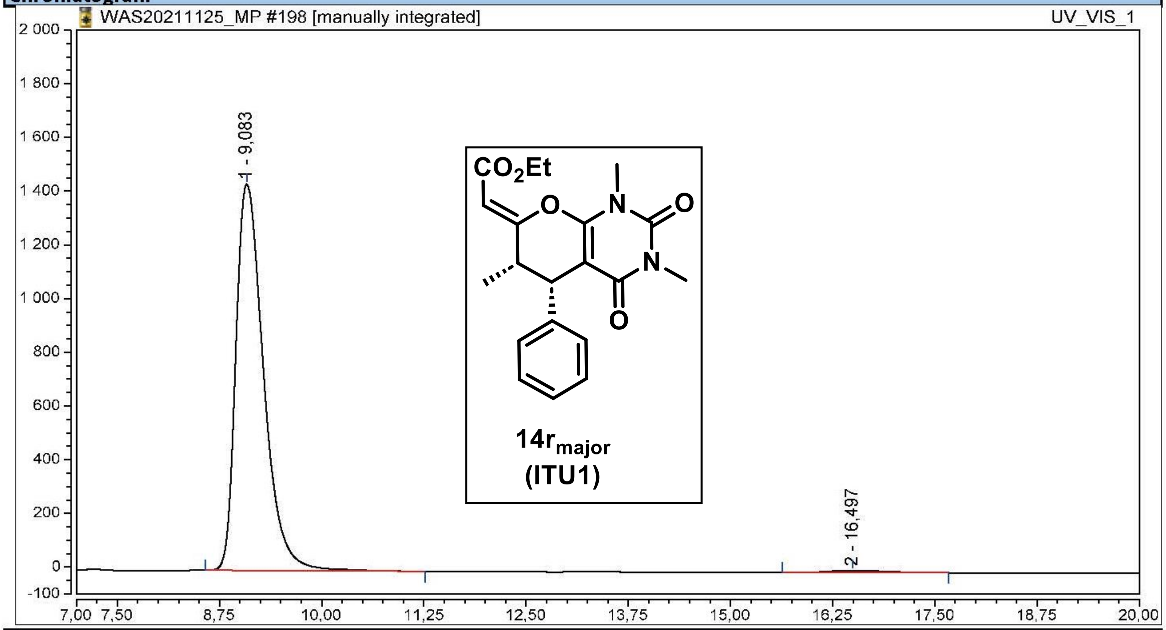

Integration Results

No.	Peak Name	Retention Time<br>min	Area<br>mAU*min	Height<br>mAU	Relative Area<br>%	Relative Height<br>%	Amount<br>n.a.
1		9,083	564,547	1439,757	98,79	99,41	n.a.
2		16,497	6,941	8,613	1,21	0,59	n.a.
**Total:**			**571,488**	**1448,369**	**100,00**	**100,00**	

Chromatogram

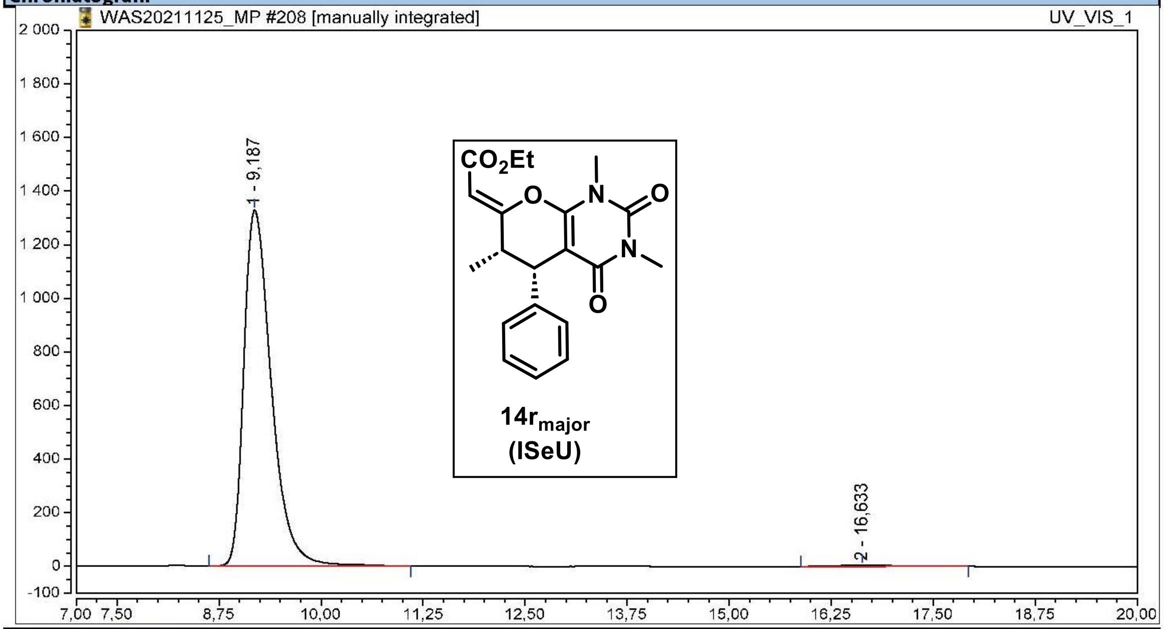

Integration Results

No.	Peak Name	Retention Time min	Area mAU*min	Height mAU	Relative Area %	Relative Height %	Amount n.a.
1		9,187	519,893	1329,073	99,23	99,64	n.a.
2		16,633	4,059	4,774	0,77	0,36	n.a.
**Total:**			**523,952**	**1333,847**	**100,00**	**100,00**	

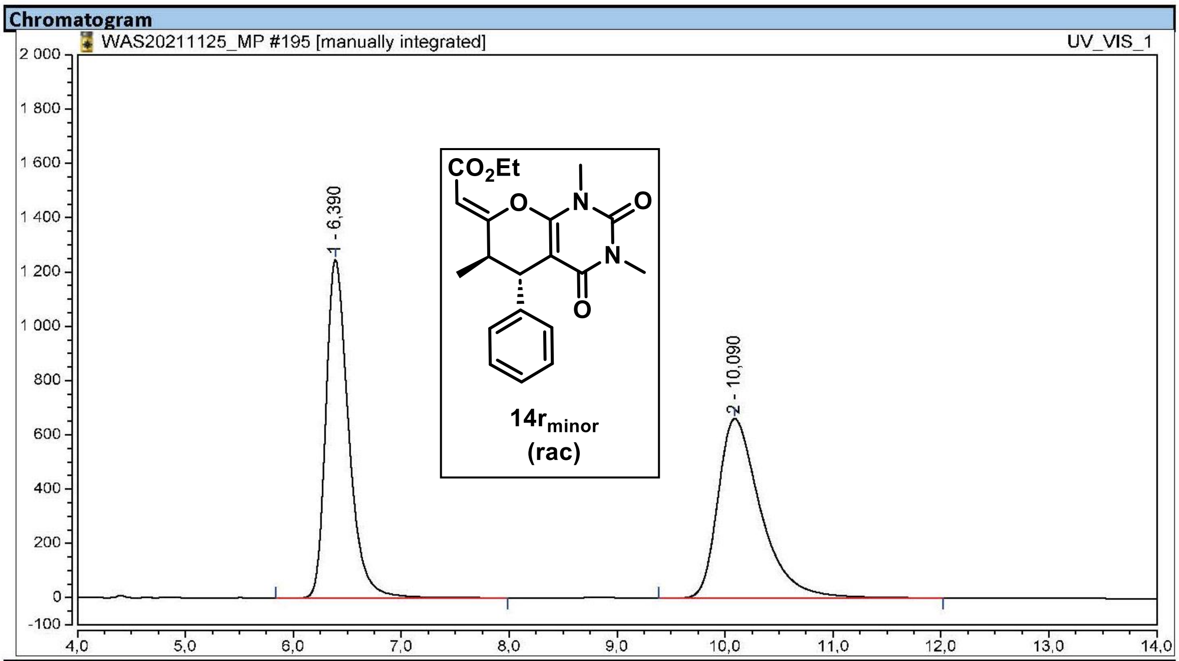

Integration Results

No.	Peak Name	Retention Time min	Area mAU*min	Height mAU	Relative Area %	Relative Height %	Amount n.a.
1		6,390	303,889	1249,412	50,02	65,39	n.a.
2		10,090	303,617	661,235	49,98	34,61	n.a.
**Total:**			**607,506**	**1910,647**	**100,00**	**100,00**	

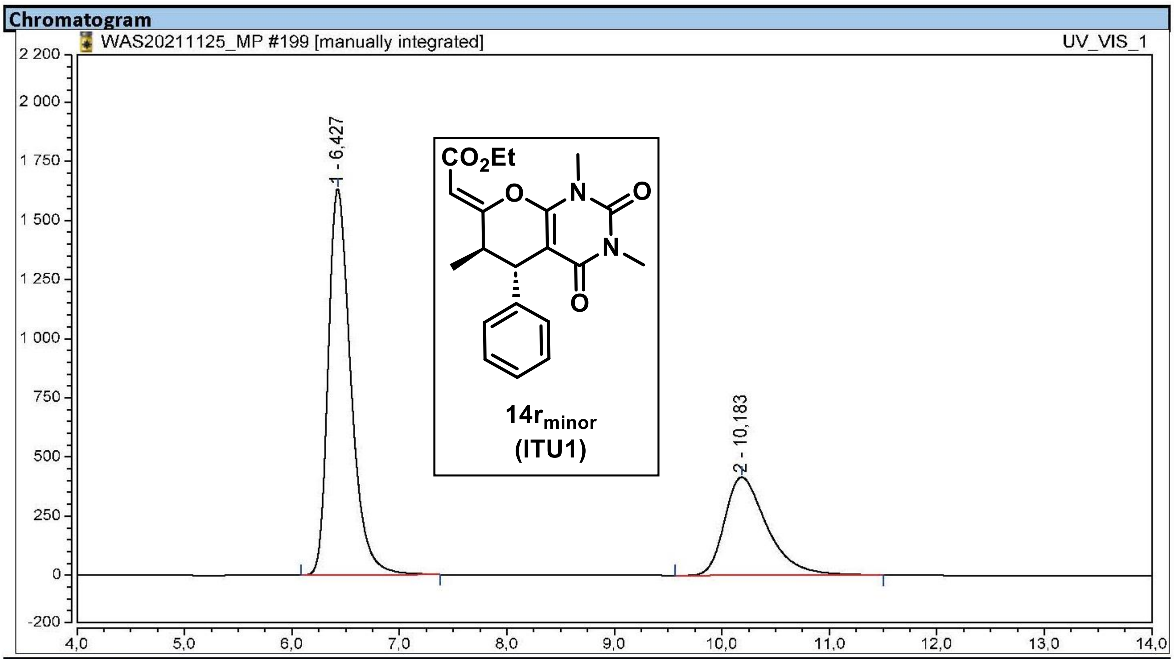

Integration Results

No.	Peak Name	Retention Time min	Area mAU*min	Height mAU	Relative Area %	Relative Height %	Amount n.a.
1		6,427	398,536	1633,338	67,72	79,72	n.a.
2		10,183	189,940	415,521	32,28	20,28	n.a.
**Total:**			**588,476**	**2048,859**	**100,00**	**100,00**	

Chromatogram

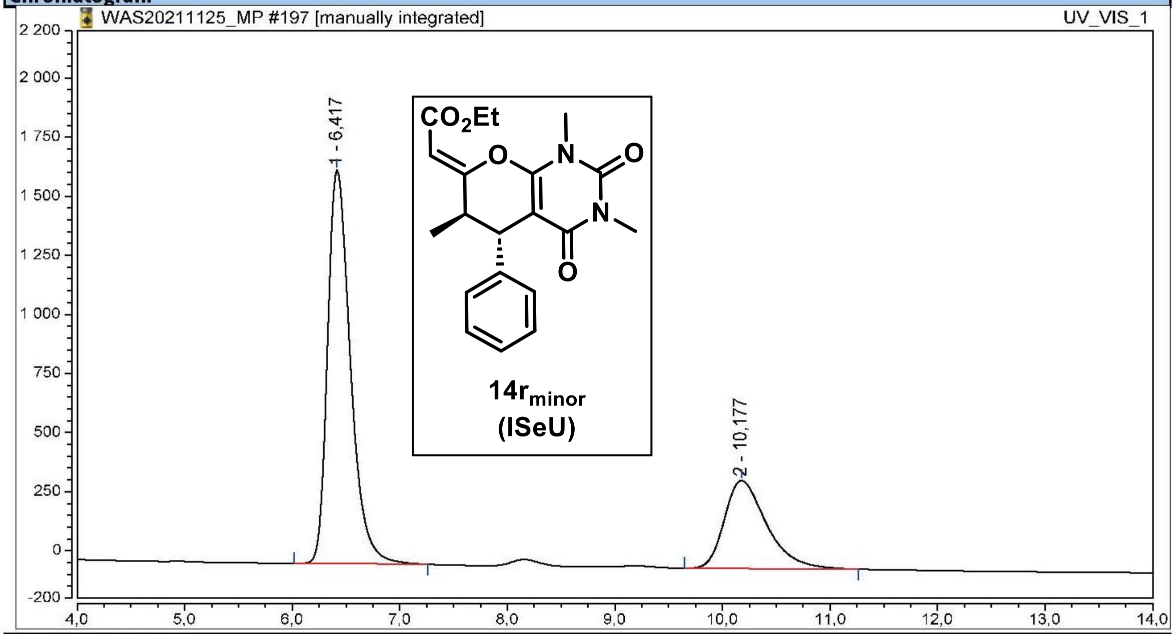

Integration Results

No.	Peak Name	Retention Time min	Area mAU*min	Height mAU	Relative Area %	Relative Height %	Amount n.a.
1		6,417	409,475	1666,092	70,72	81,73	n.a.
2		10,177	169,570	372,330	29,28	18,27	n.a.
**Total:**			**579,045**	**2038,422**	**100,00**	**100,00**	

Chromatogram

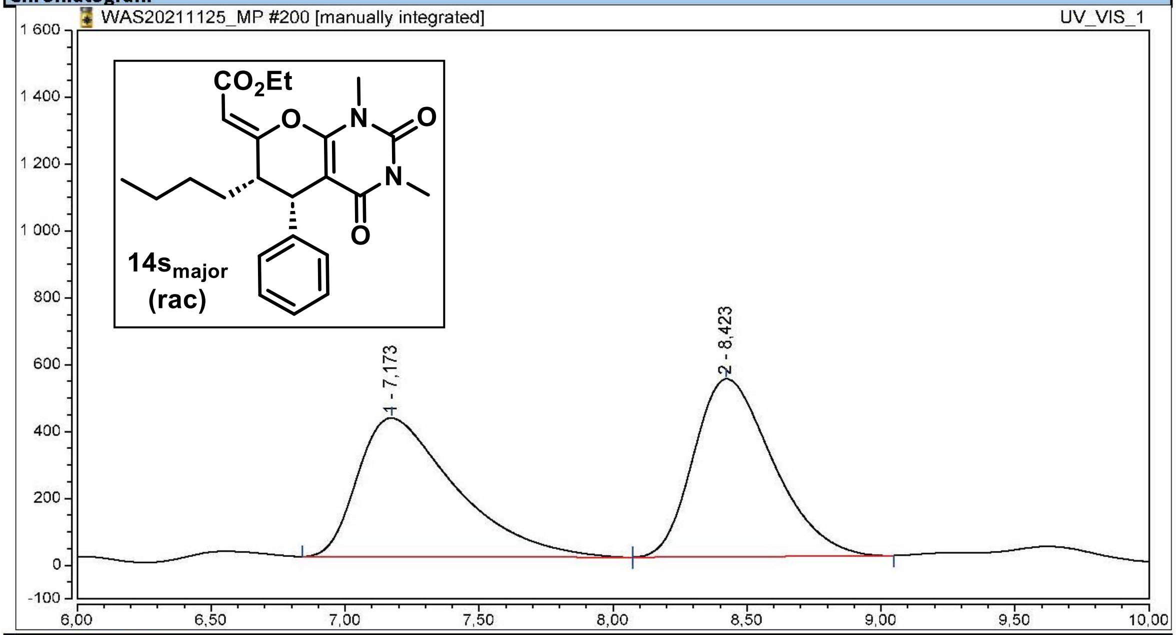

Integration Results

No.	Peak Name	Retention Time<br>min	Area<br>mAU*min	Height<br>mAU	Relative Area<br>%	Relative Height<br>%	Amount<br>n.a.
1		7,173	175,530	414,791	49,05	43,82	n.a.
2		8,423	182,302	531,788	50,95	56,18	n.a.
**Total:**			**357,831**	**946,579**	**100,00**	**100,00**	

Chromatogram

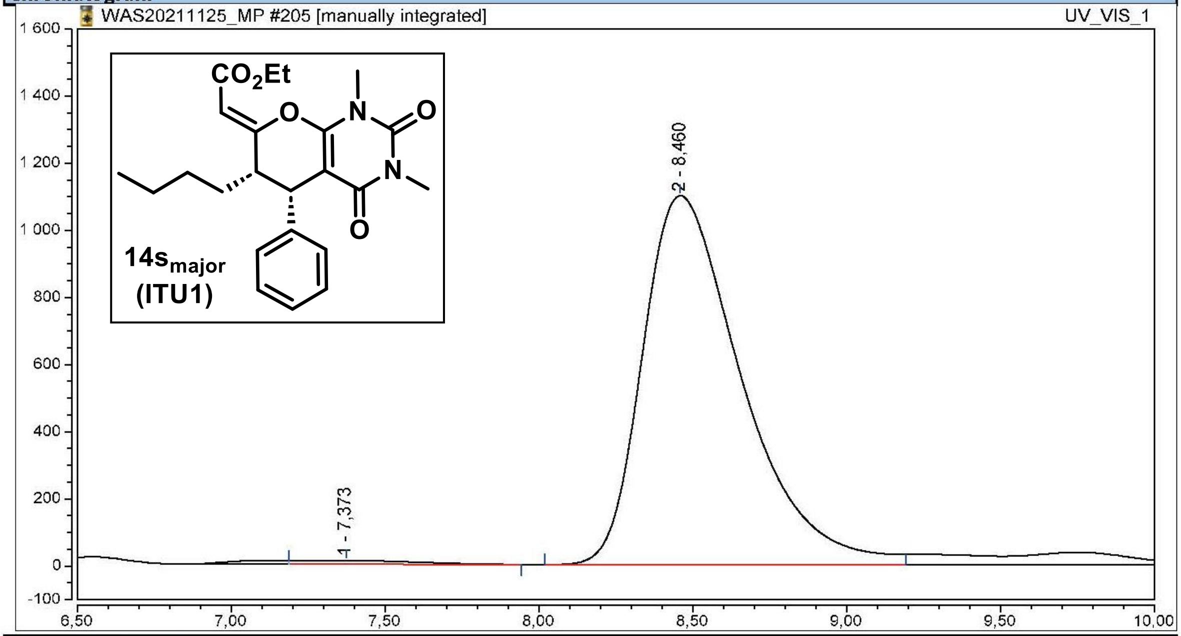

Integration Results

No.	Peak Name	Retention Time<br>min	Area<br>mAU*min	Height<br>mAU	Relative Area<br>%	Relative Height<br>%	Amount<br>n.a.
1		7,373	5,111	11,442	1,23	1,03	n.a.
2		8,460	410,531	1099,650	98,77	98,97	n.a.
**Total:**			**415,642**	**1111,092**	**100,00**	**100,00**	

Chromatogram

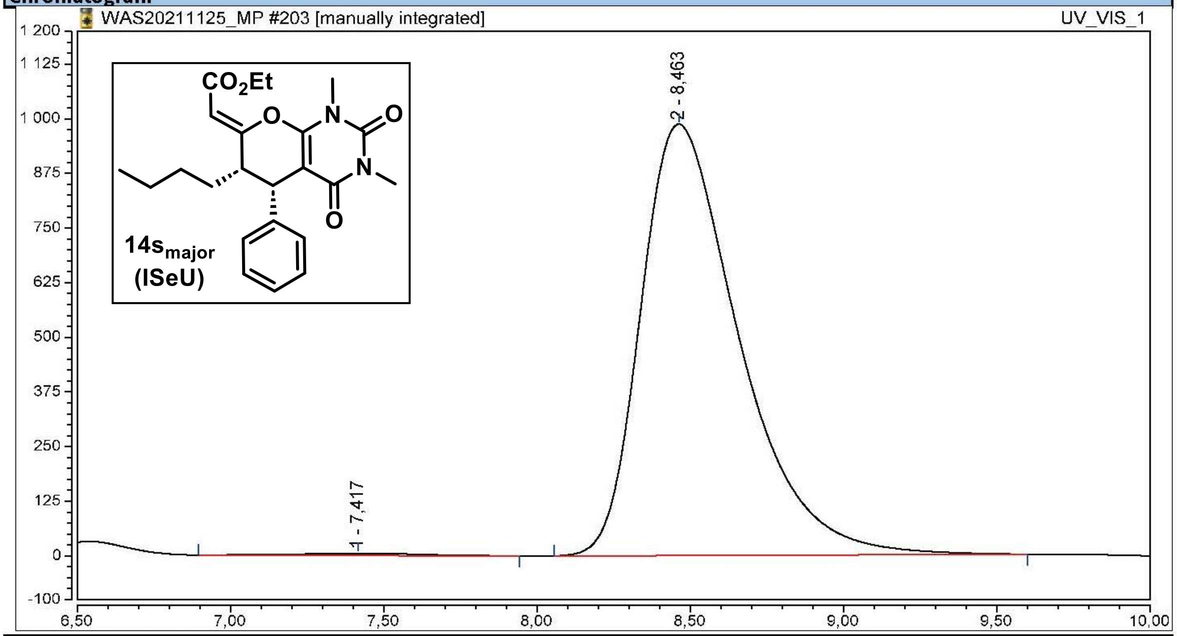

Integration Results

No.	Peak Name	Retention Time min	Area mAU*min	Height mAU	Relative Area %	Relative Height %	Amount n.a.
1		7,417	2,619	5,101	0,71	0,51	n.a.
2		8,463	366,274	986,696	99,29	99,49	n.a.
**Total:**			**368,894**	**991,797**	**100,00**	**100,00**	

Chromatogram

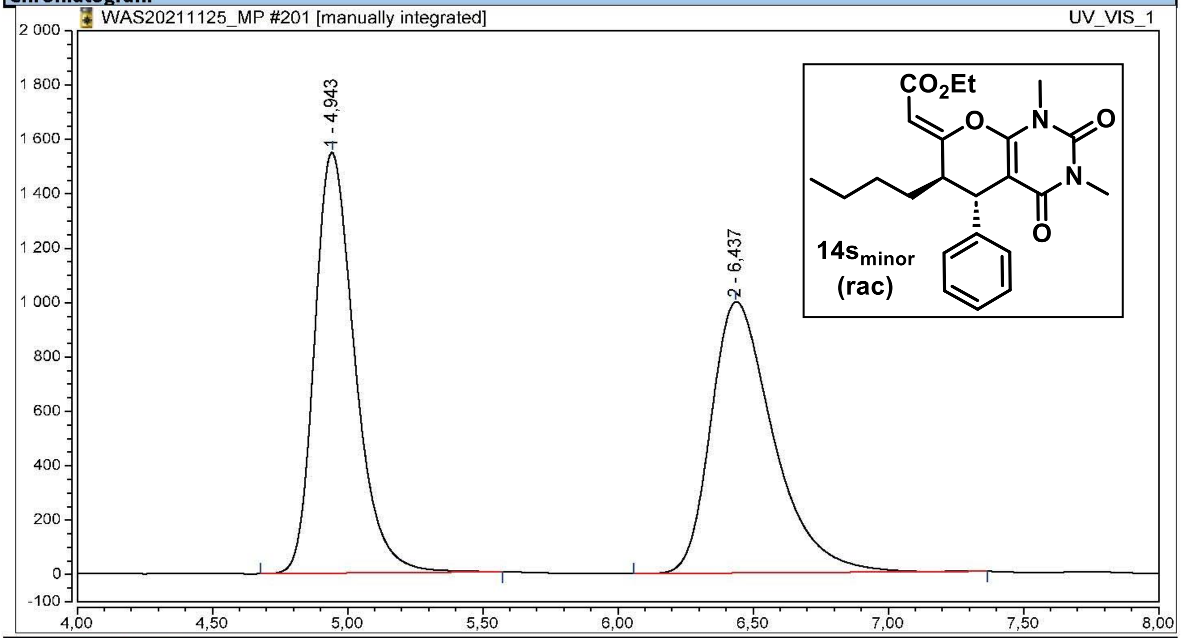

Integration Results

No.	Peak Name	Retention Time min	Area mAU*min	Height mAU	Relative Area %	Relative Height %	Amount n.a.
1		4,943	267,233	1548,453	50,01	60,81	n.a.
2		6,437	267,095	997,895	49,99	39,19	n.a.
**Total:**			**534,328**	**2546,348**	**100,00**	**100,00**	

Chromatogram

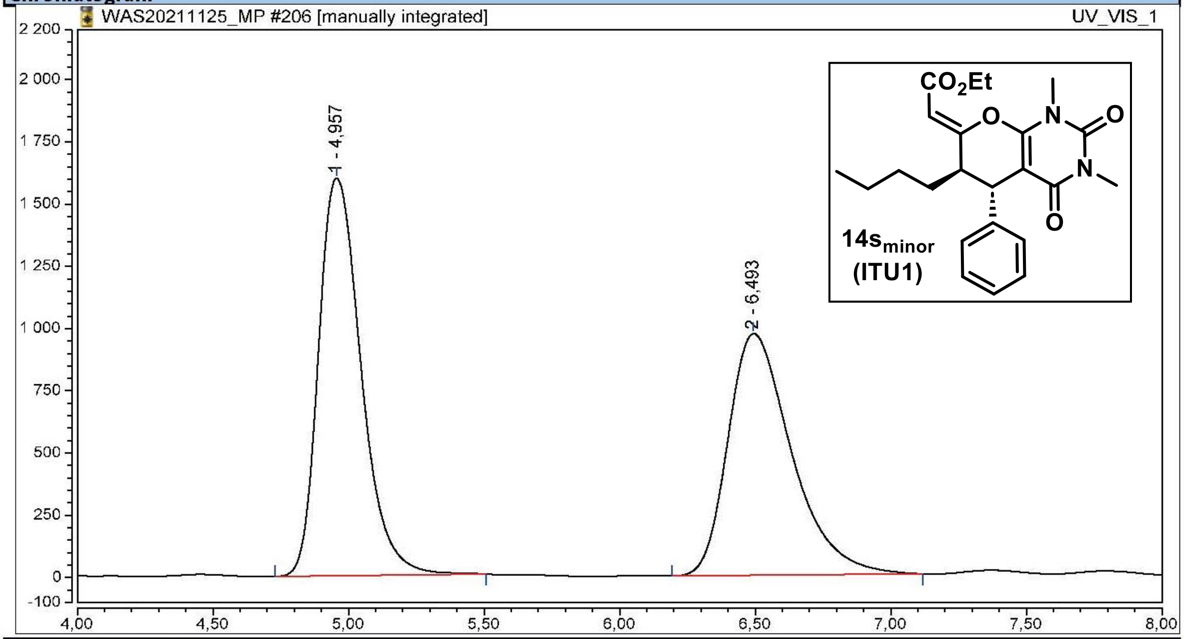

Integration Results

No.	Peak Name	Retention Time min	Area mAU*min	Height mAU	Relative Area %	Relative Height %	Amount n.a.
1		4,957	290,667	1598,240	52,43	62,21	n.a.
2		6,493	263,747	970,815	47,57	37,79	n.a.
**Total:**			**554,414**	**2569,055**	**100,00**	**100,00**	

Chromatogram

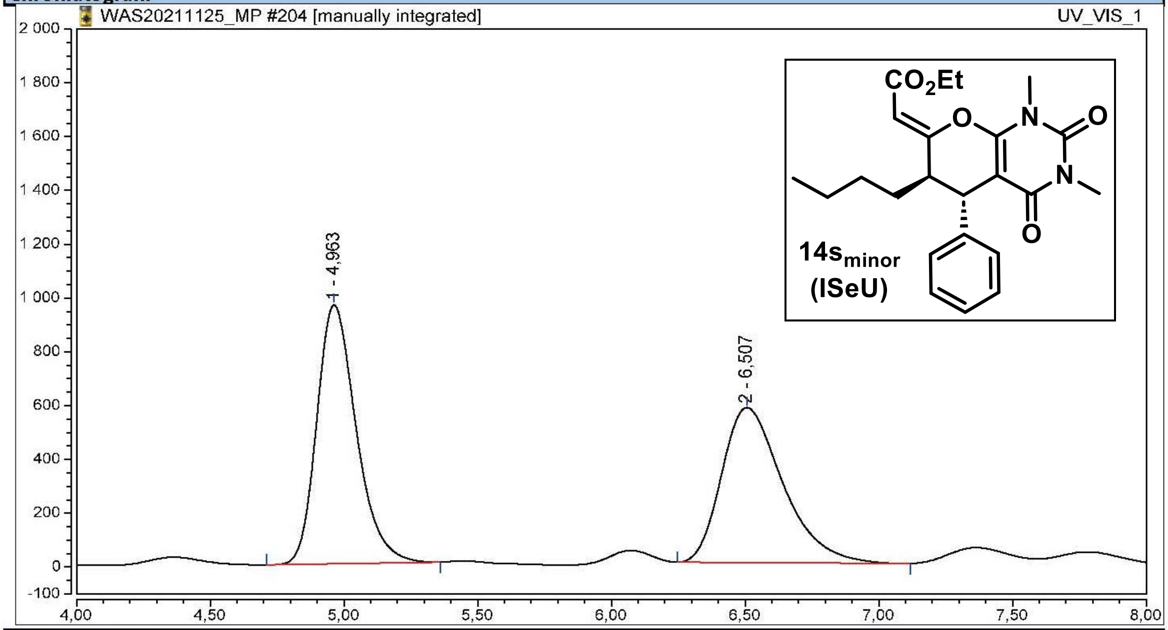

Integration Results

No.	Peak Name	Retention Time<br>min	Area<br>mAU*min	Height<br>mAU	Relative Area<br>%	Relative Height<br>%	Amount<br>n.a.
1		4,963	164,420	963,357	51,79	62,57	n.a.
2		6,507	153,055	576,404	48,21	37,43	n.a.
**Total:**			**317,475**	**1539,761**	**100,00**	**100,00**	

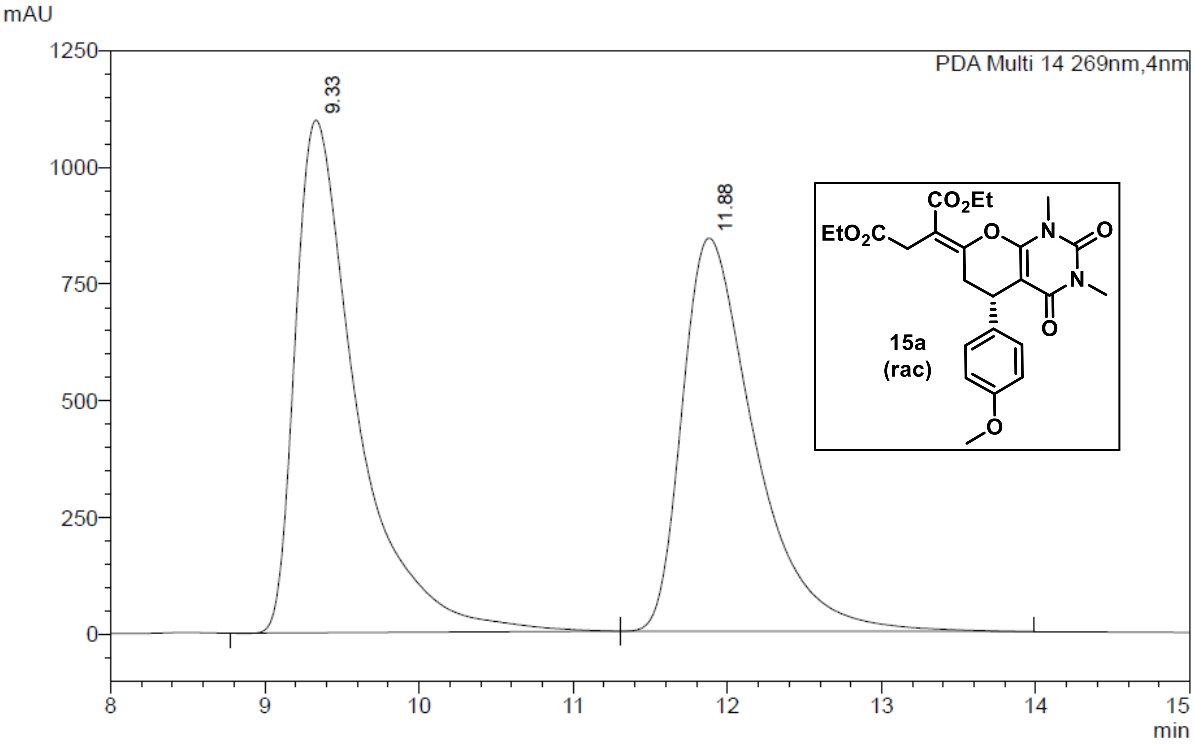

Peak Table

PDA Ch14 269nm

Peak#	Ret. Time	Area	Area%
1	9.33	30520080	52.50
2	11.88	27612013	47.50
Total		58132093	100.00

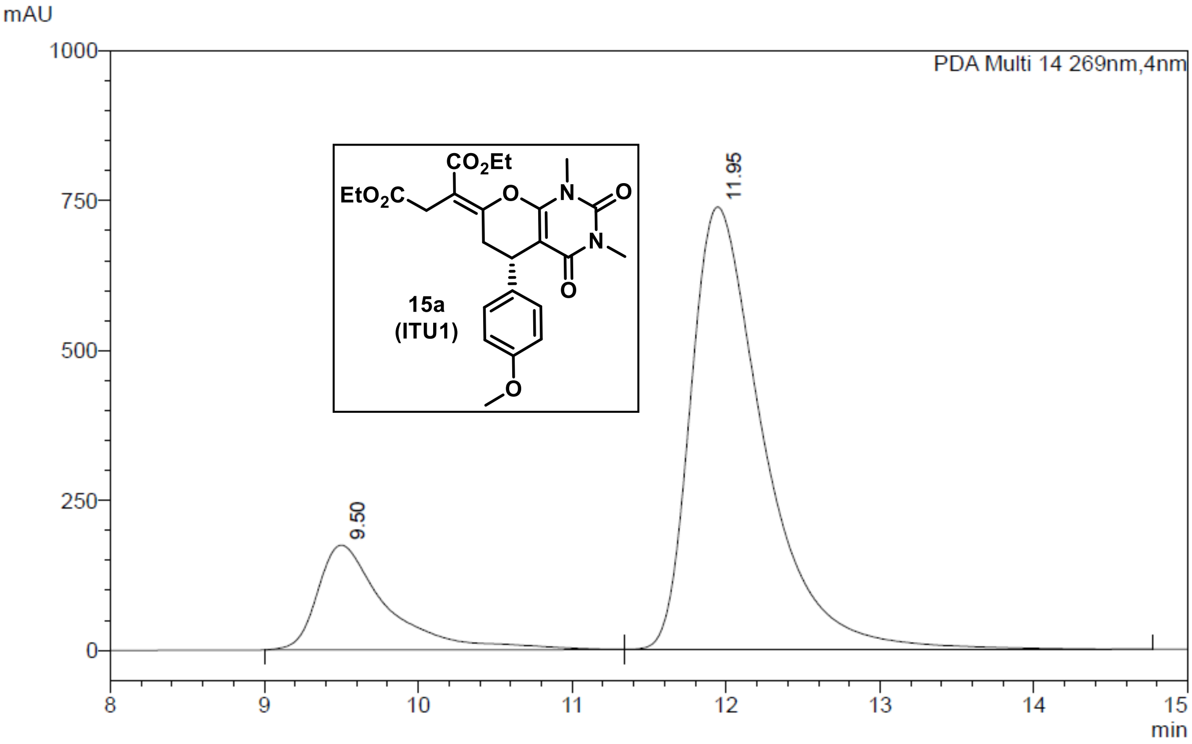

Peak Table

PDA Ch14 269nm

Peak#	Ret. Time	Area	Area%
1	9.50	5464545	18.35
2	11.95	24309092	81.65
Total		29773636	100.00

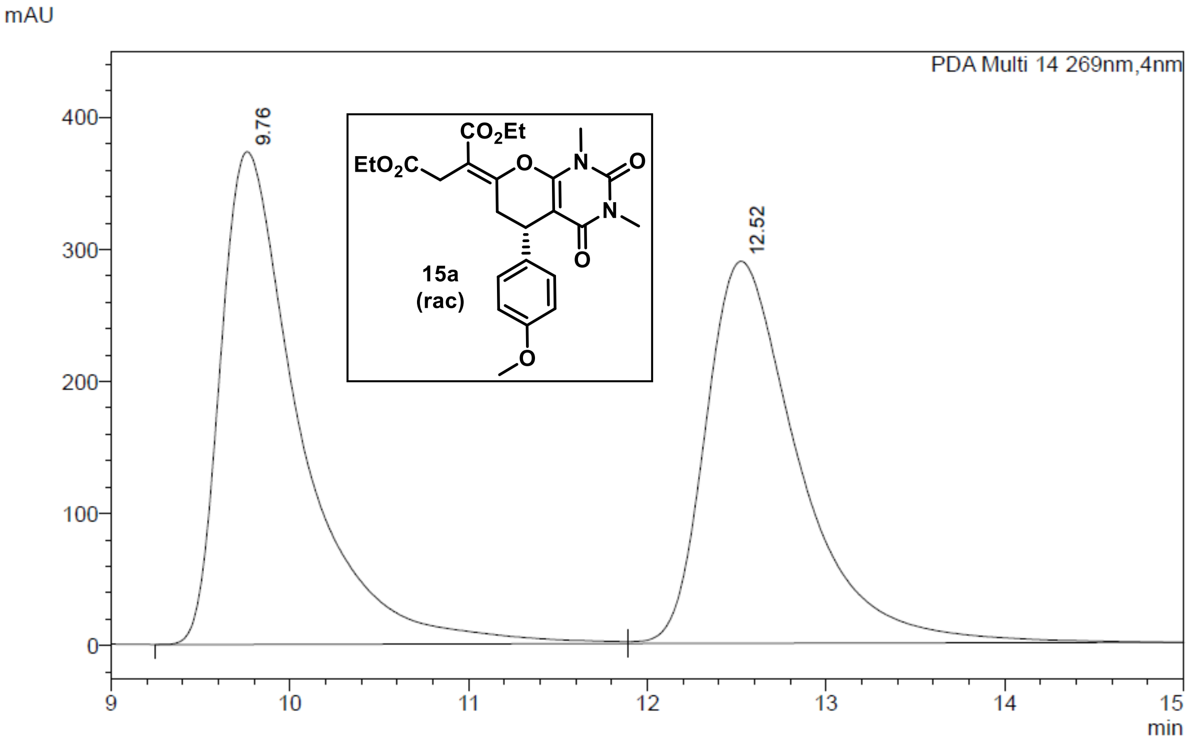

Peak Table

PDA Ch14 269nm

Peak#	Ret. Time	Area	Area%
1	9.76	11600038	52.36
2	12.52	10552800	47.64
Total		22152838	100.00

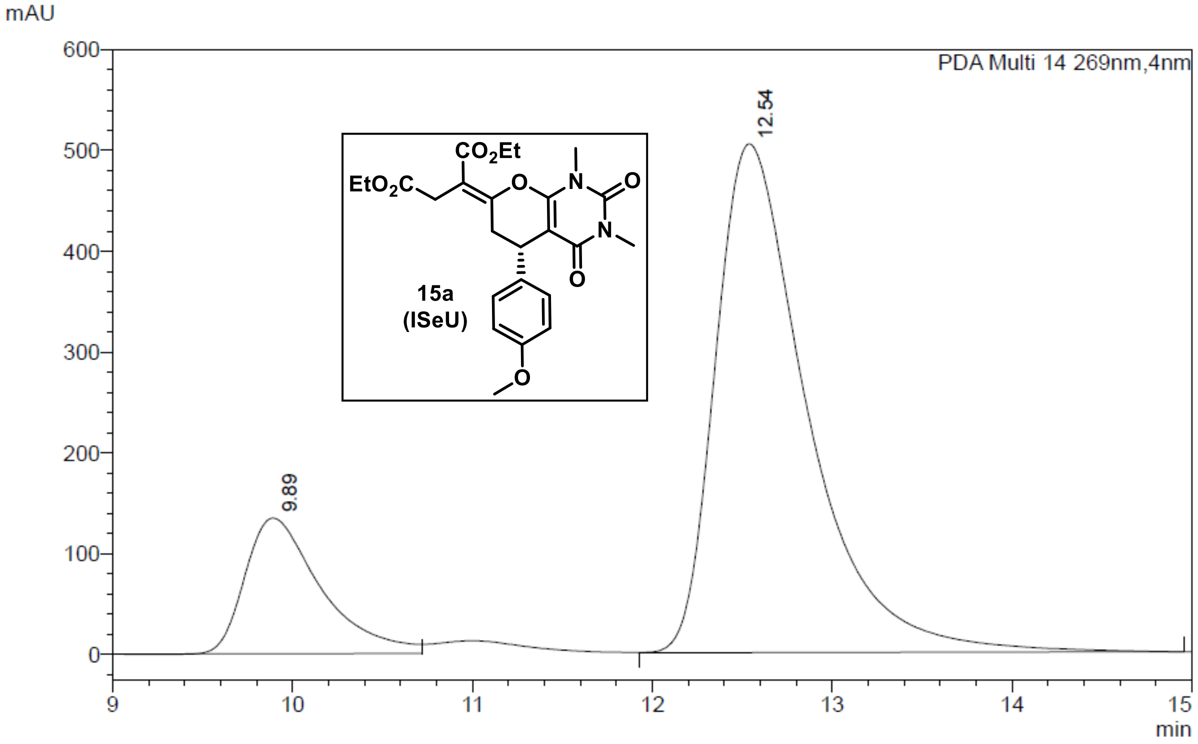

Peak Table

PDA Ch14 269nm

Peak#	Ret. Time	Area	Area%
1	9.89	3952687	18.03
2	12.54	17972438	81.97
Total		21925124	100.00

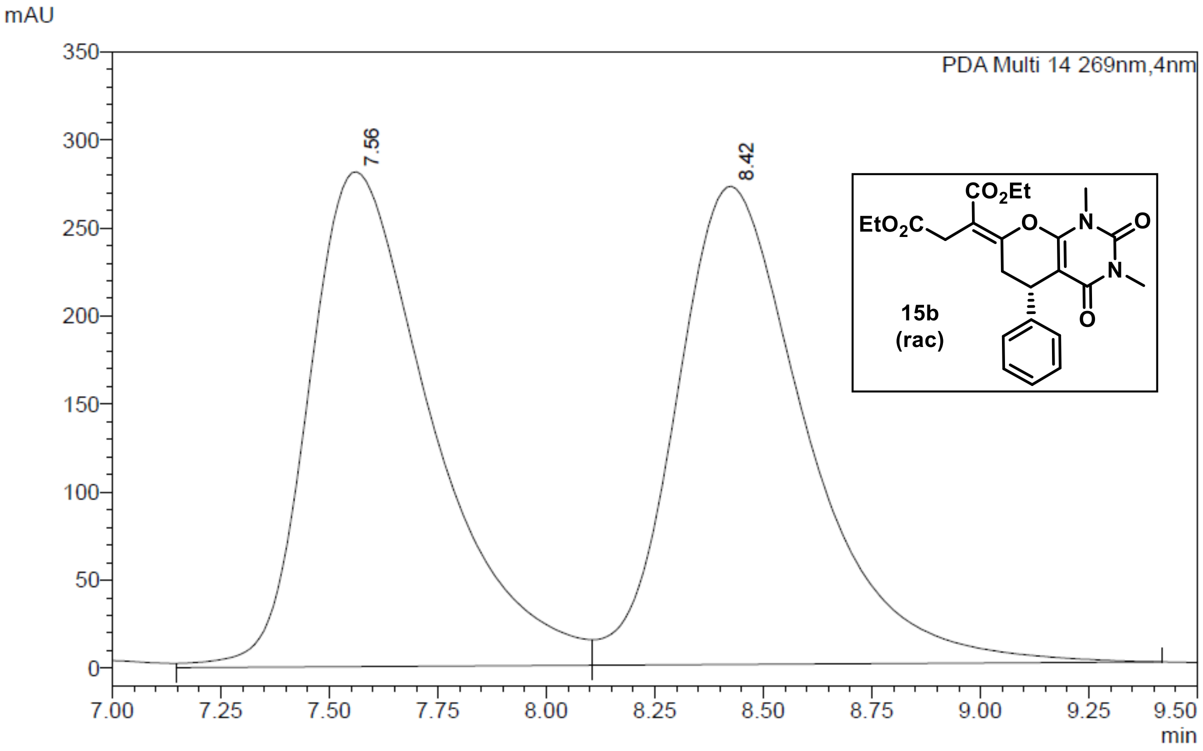

Peak Table

PDA Ch14 269nm

Peak#	Ret. Time	Area	Area%
1	7.56	5562548	49.39
2	8.42	5699827	50.61
Total		11262375	100.00

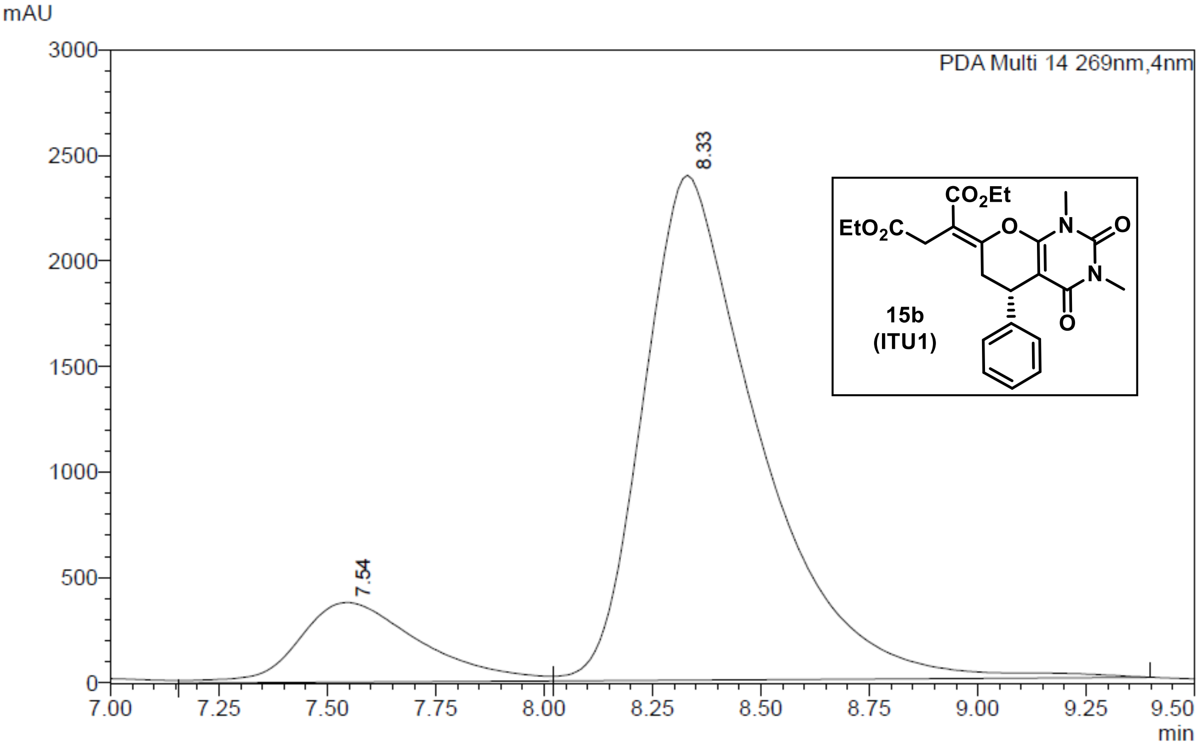

Peak Table

PDA Ch14 269nm

Peak#	Ret. Time	Area	Area%
1	7.54	7365952	14.19
2	8.33	44550710	85.81
Total		51916662	100.00

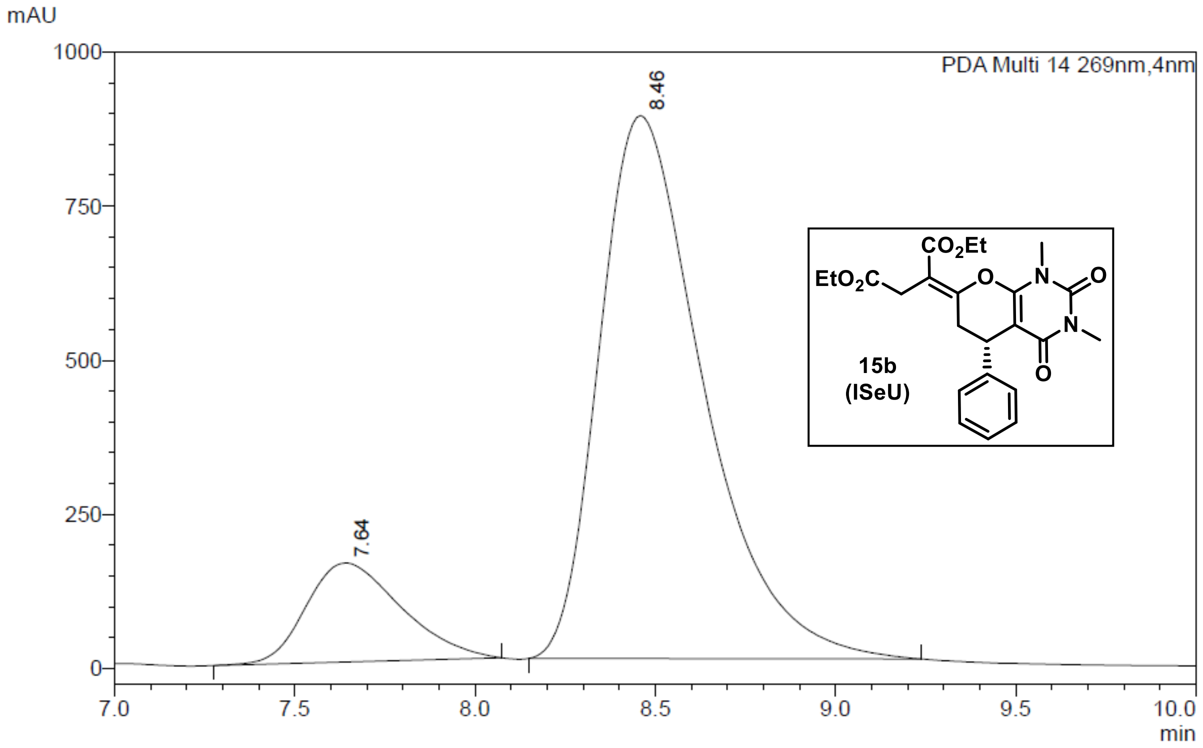

Peak Table

PDA Ch14 269nm

Peak#	Ret. Time	Area	Area%
1	7.64	2942231	14.26
2	8.46	17687722	85.74
Total		20629953	100.00

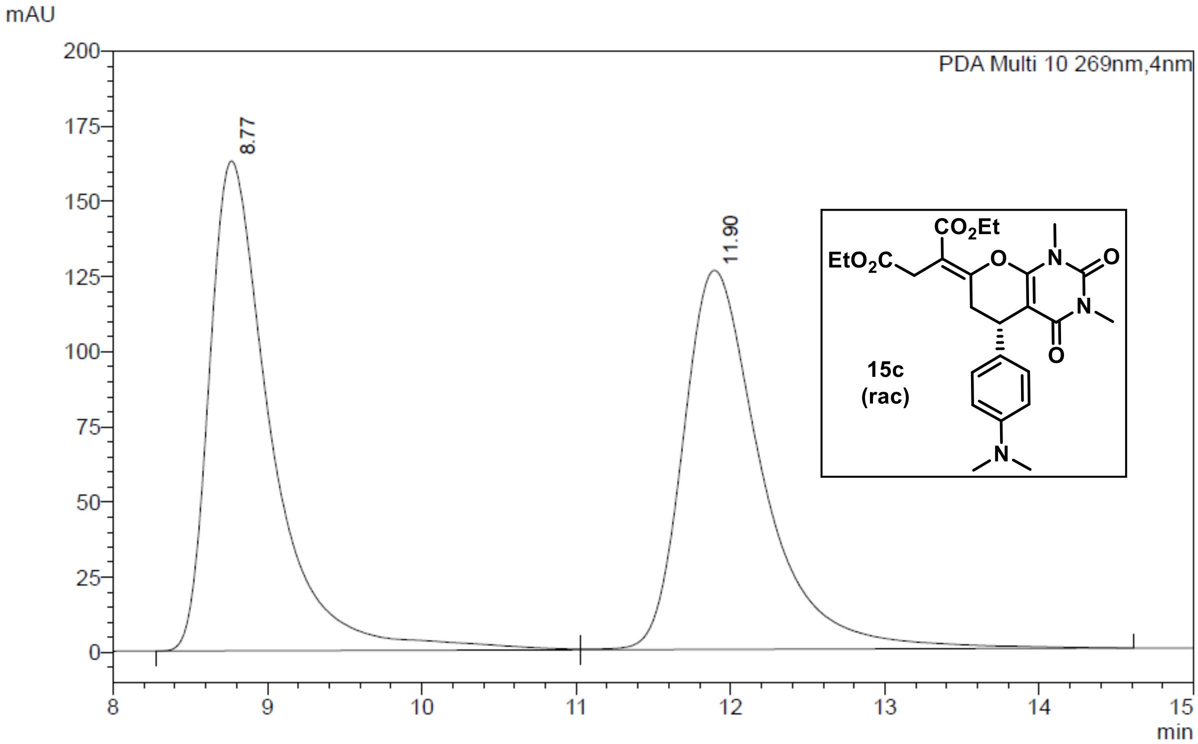

Peak Table

PDA Ch10 269nm

Peak#	Ret. Time	Area	Area%
1	8.77	4529702	50.24
2	11.90	4486805	49.76
Total		9016507	100.00

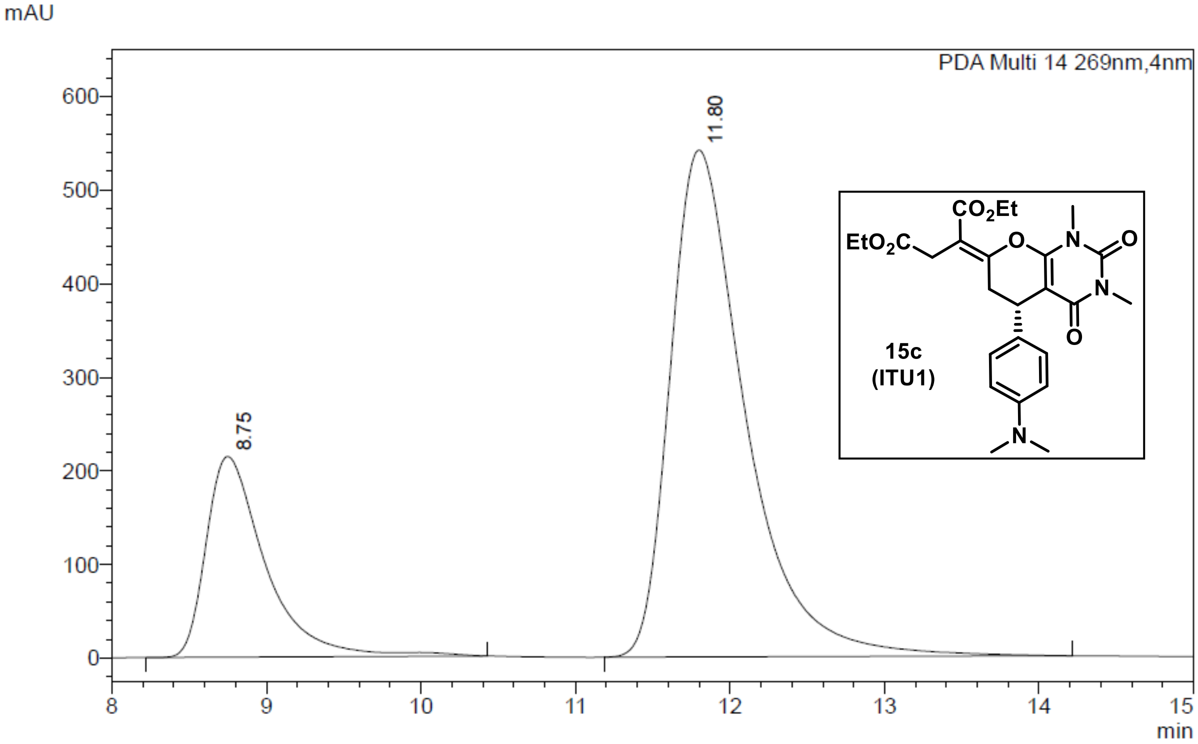

Peak Table

PDA Ch14 269nm

Peak#	Ret. Time	Area	Area%
1	8.75	5733720	23.79
2	11.80	18368845	76.21
Total		24102565	100.00

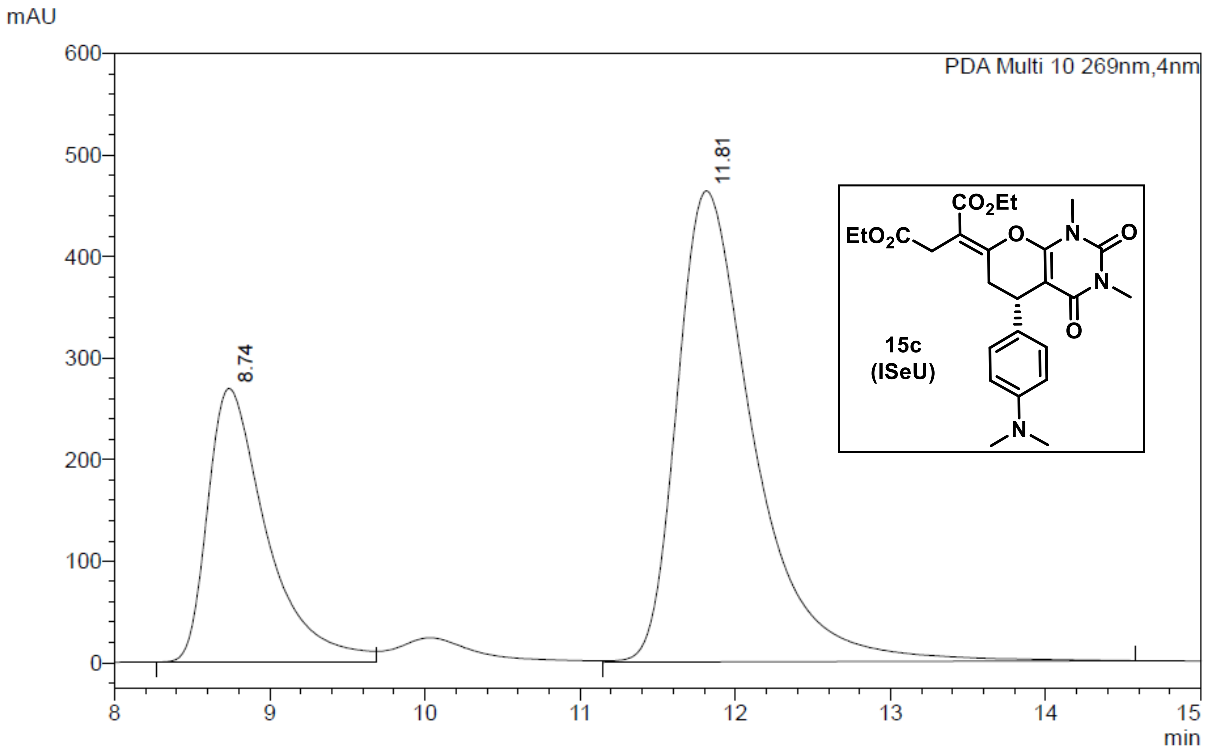

Peak Table

PDA Ch10 269nm

Peak#	Ret. Time	Area	Area%
1	8.74	7158145	30.97
2	11.81	15958467	69.03
Total		23116612	100.00

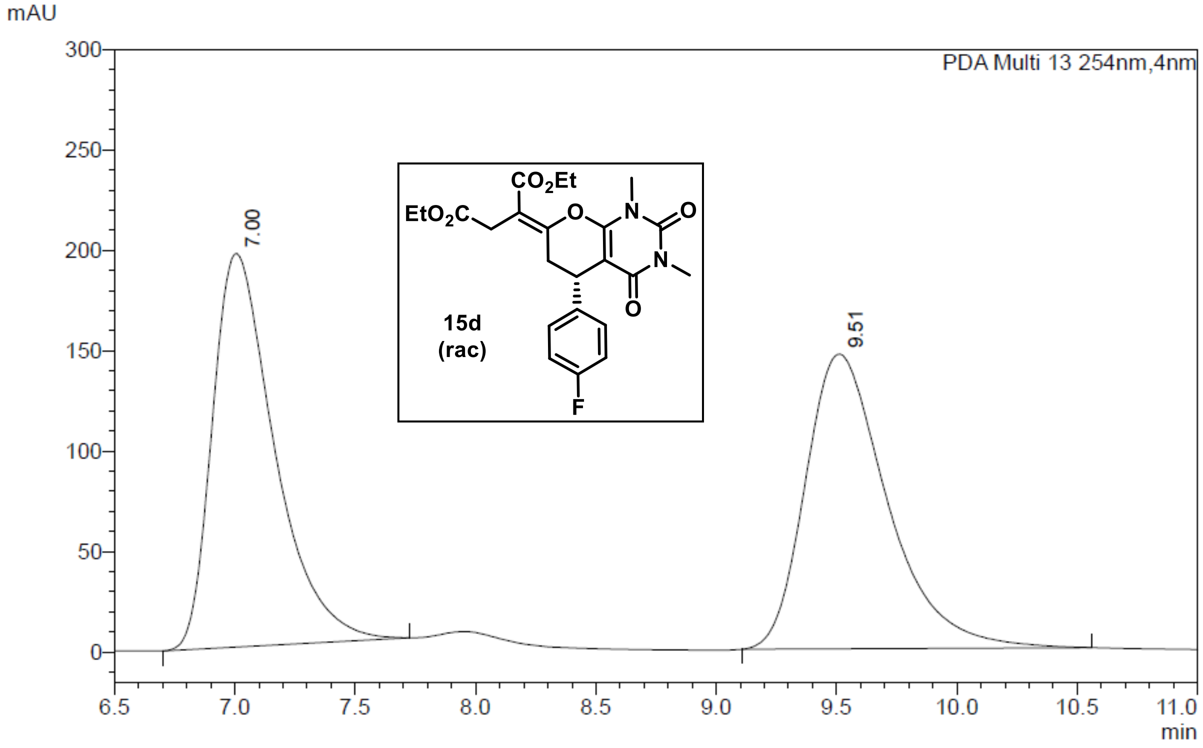

Peak Table

PDA Ch13 254nm

Peak#	Ret. Time	Area	Area%
1	7.00	3522850	50.28
2	9.51	3483448	49.72
Total		7006298	100.00

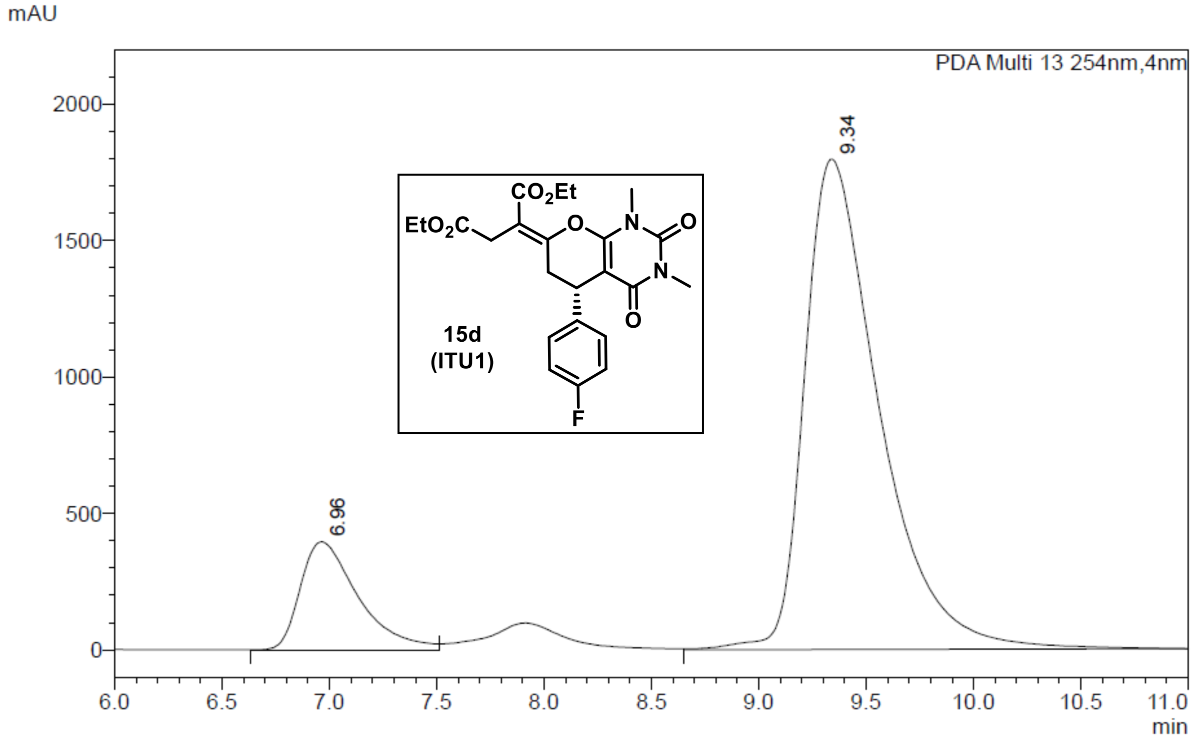

Peak Table

PDA Ch13 254nm

Peak#	Ret. Time	Area	Area%
1	6.96	7446130	14.60
2	9.34	43568355	85.40
Total		51014485	100.00

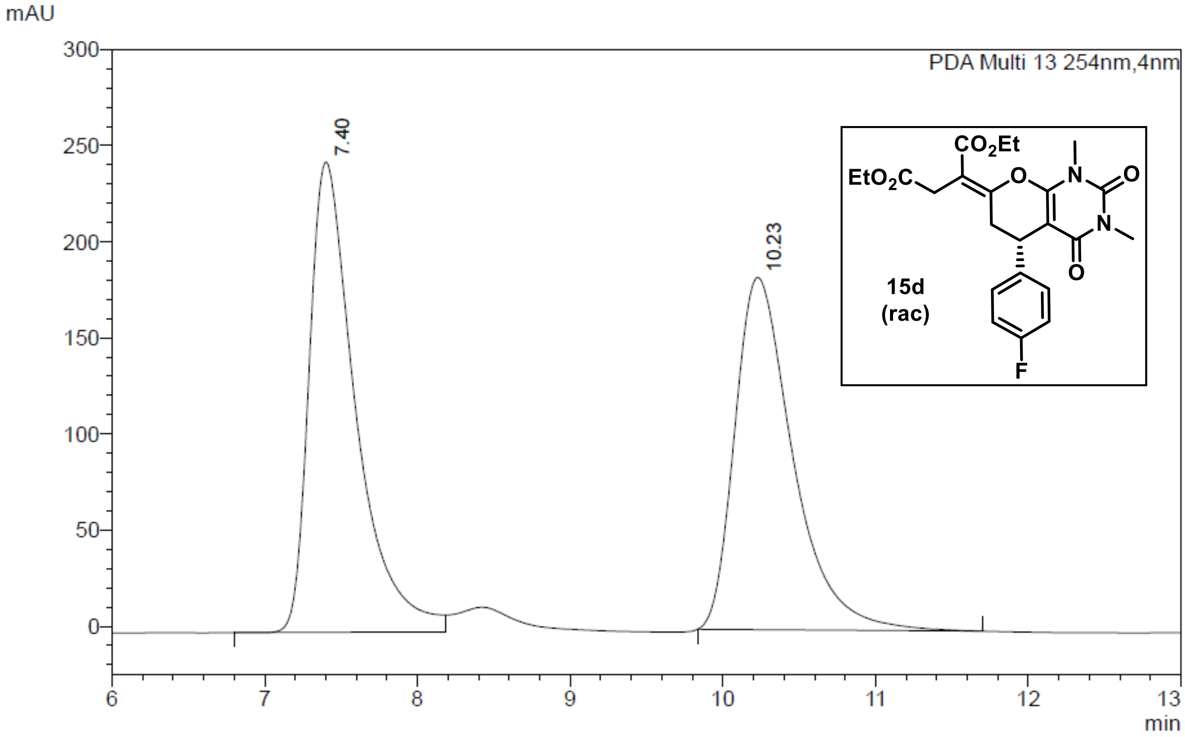

Peak Table

PDA Ch13 254nm

Peak#	Ret. Time	Area	Area%
1	7.40	5177042	51.59
2	10.23	4858480	48.41
Total		10035521	100.00

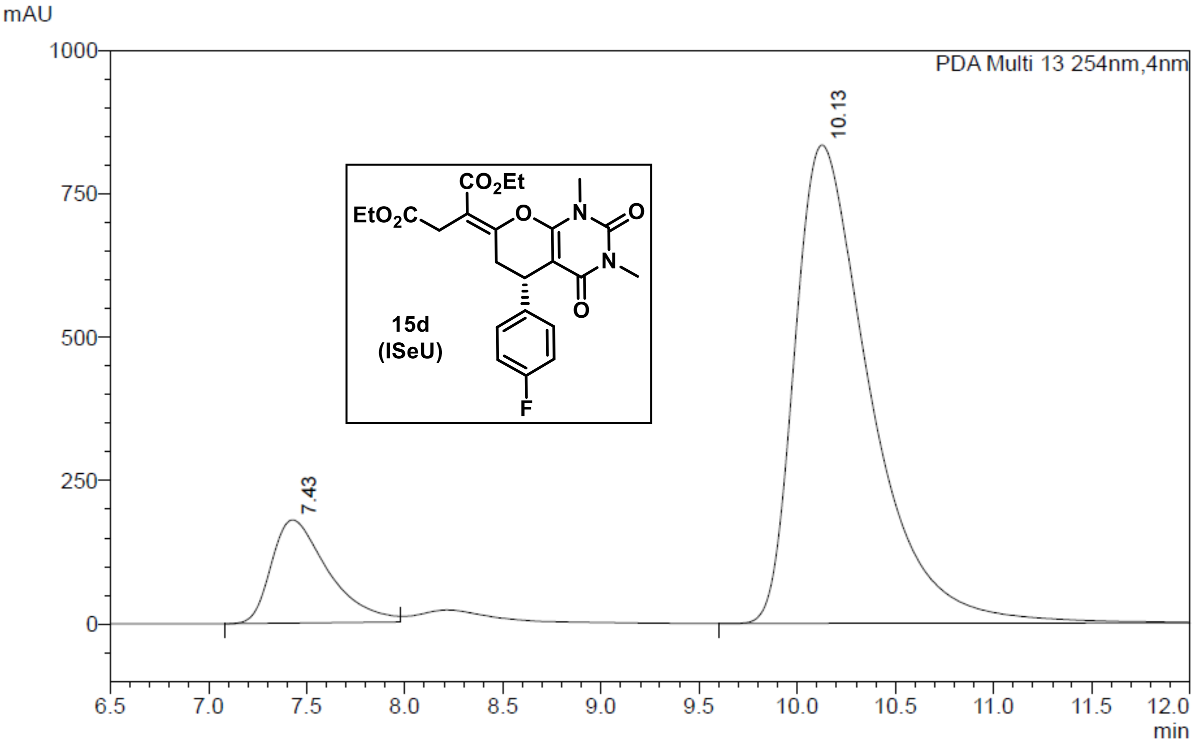

Peak Table

PDA Ch13 254nm

Peak#	Ret. Time	Area	Area%
1	7.43	3634487	14.02
2	10.13	22290051	85.98
Total		25924538	100.00

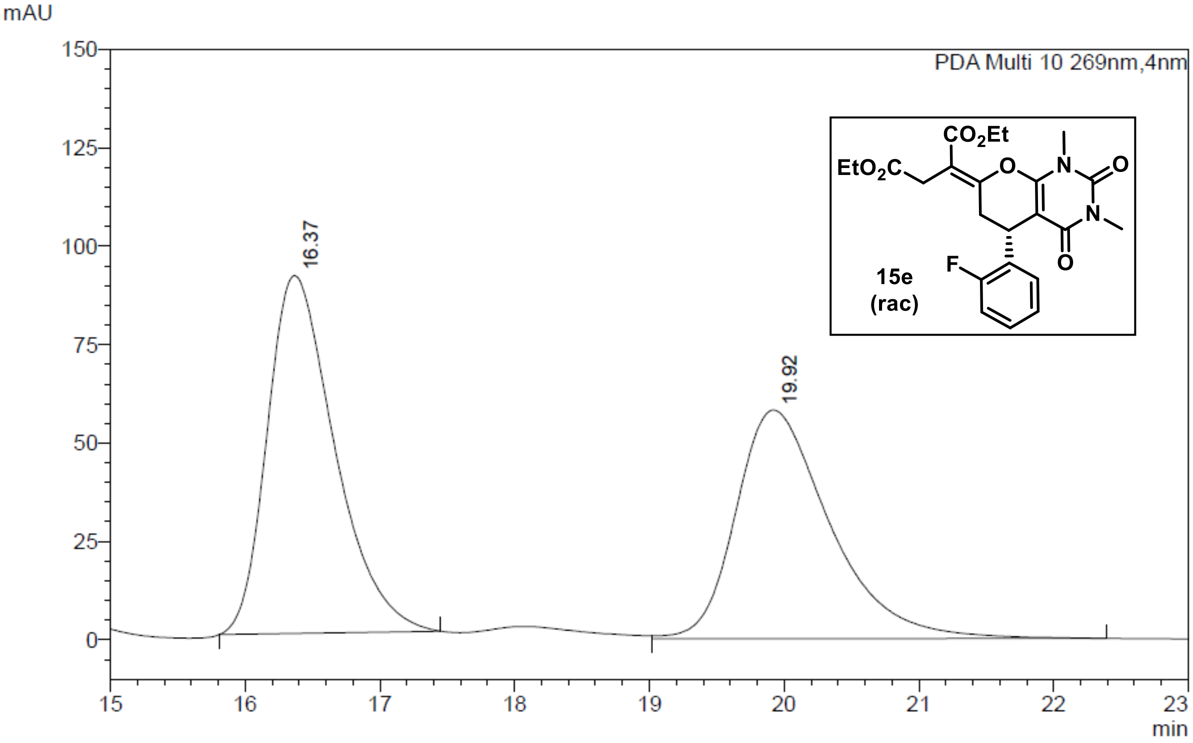

Peak Table

PDA Ch10 269nm

Peak#	Ret. Time	Area	Area%
1	16.37	3167324	52.80
2	19.92	2831951	47.20
Total		5999275	100.00

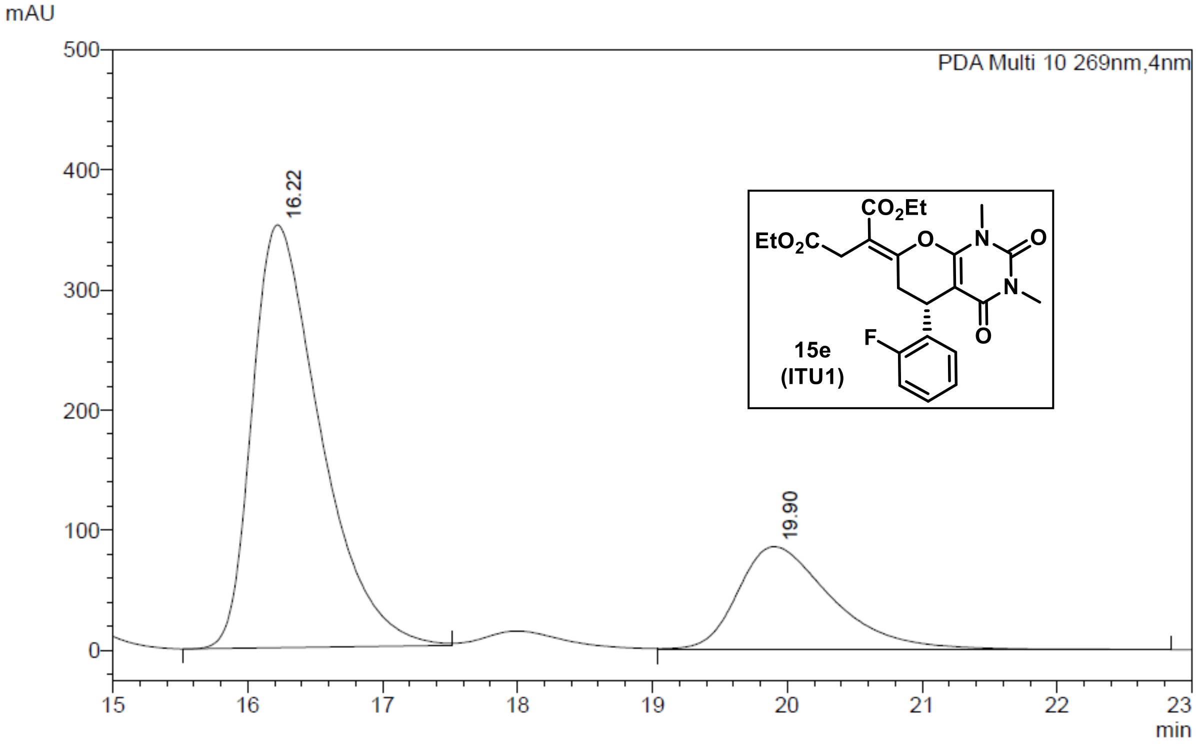

Peak Table

PDA Ch10 269nm

Peak#	Ret. Time	Area	Area%
1	16.22	12397640	74.61
2	19.90	4218401	25.39
Total		16616041	100.00

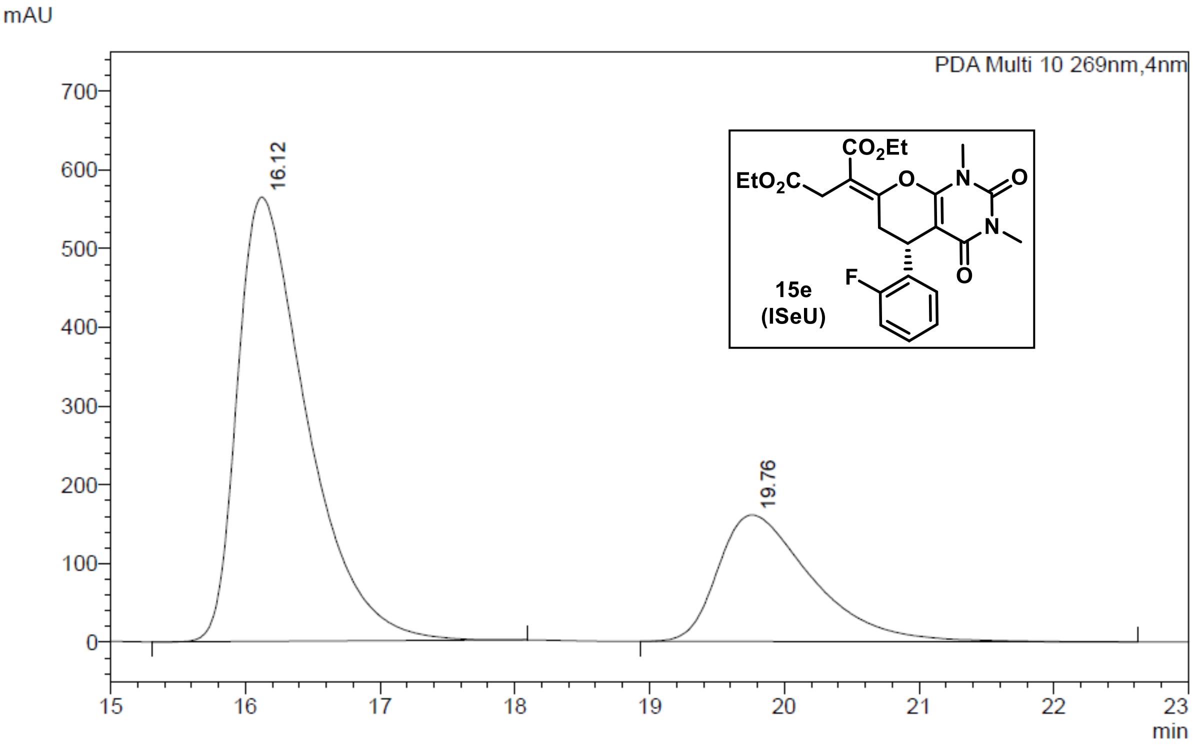

Peak Table

PDA Ch10 269nm

Peak#	Ret. Time	Area	Area%
1	16.12	20241059	72.14
2	19.76	7816266	27.86
Total		28057325	100.00

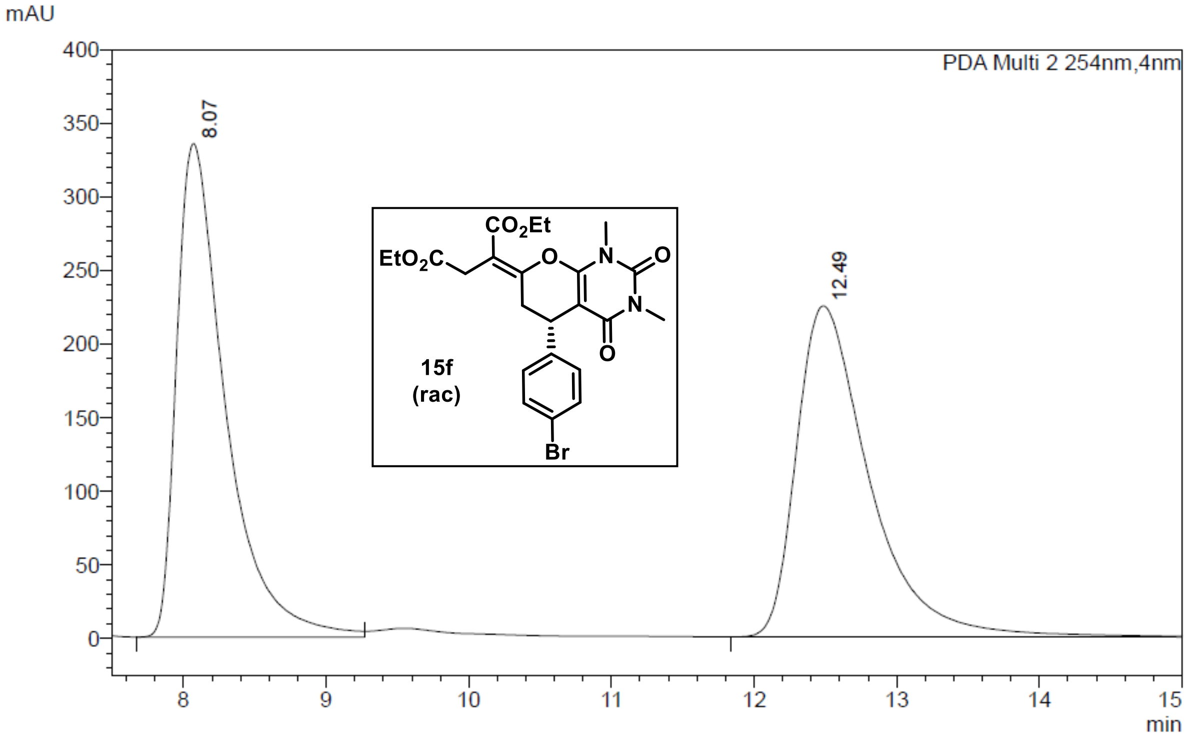

Peak Table

PDA Ch2 254nm

Peak#	Ret. Time	Area	Area%
1	8.07	7986849	49.94
2	12.49	8005597	50.06
Total		15992446	100.00

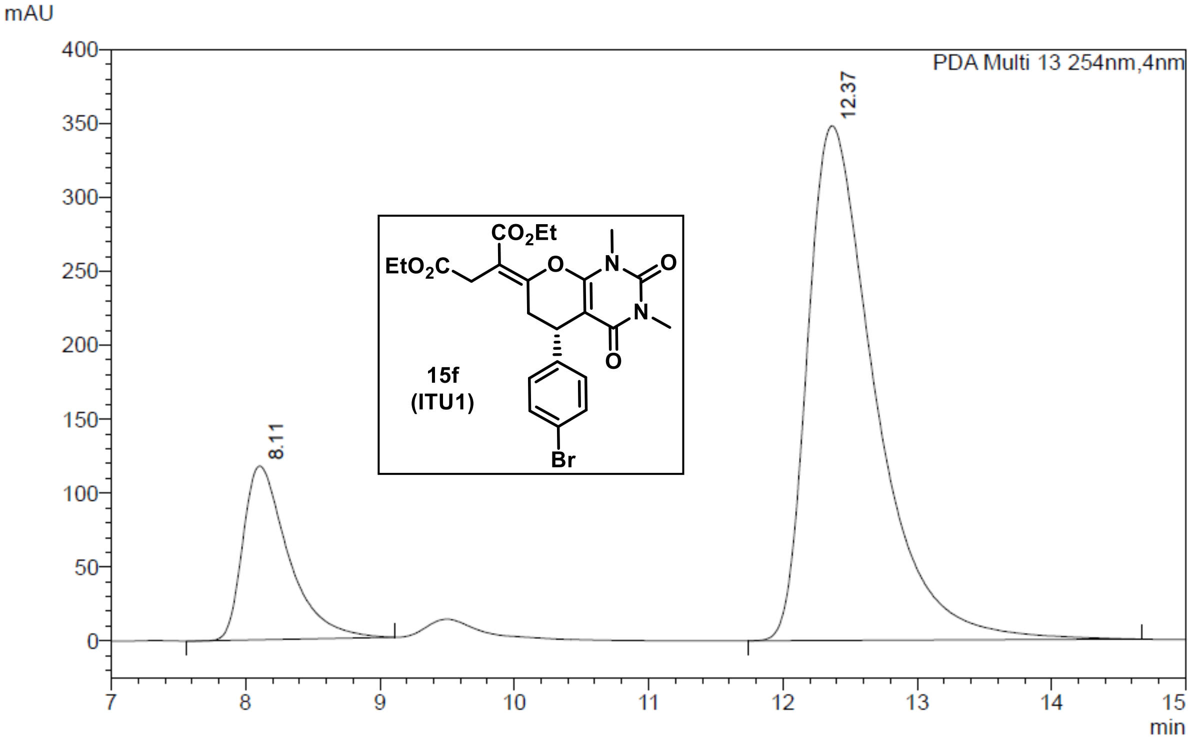

Peak Table

PDA Ch13 254nm

Peak#	Ret. Time	Area	Area%
1	8.11	2765486	18.51
2	12.37	12172884	81.49
Total		14938371	100.00

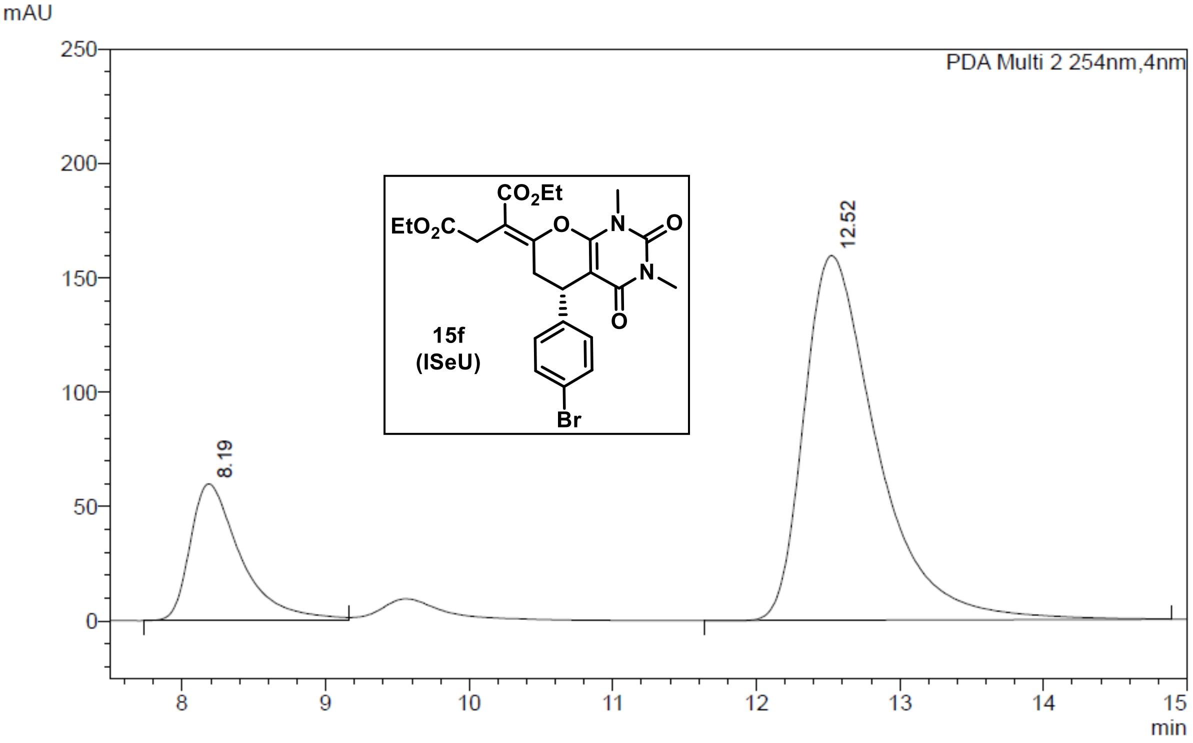

Peak Table

PDA Ch2 254nm

Peak#	Ret. Time	Area	Area%
1	8.19	1470775	20.67
2	12.52	5643160	79.33
Total		7113935	100.00

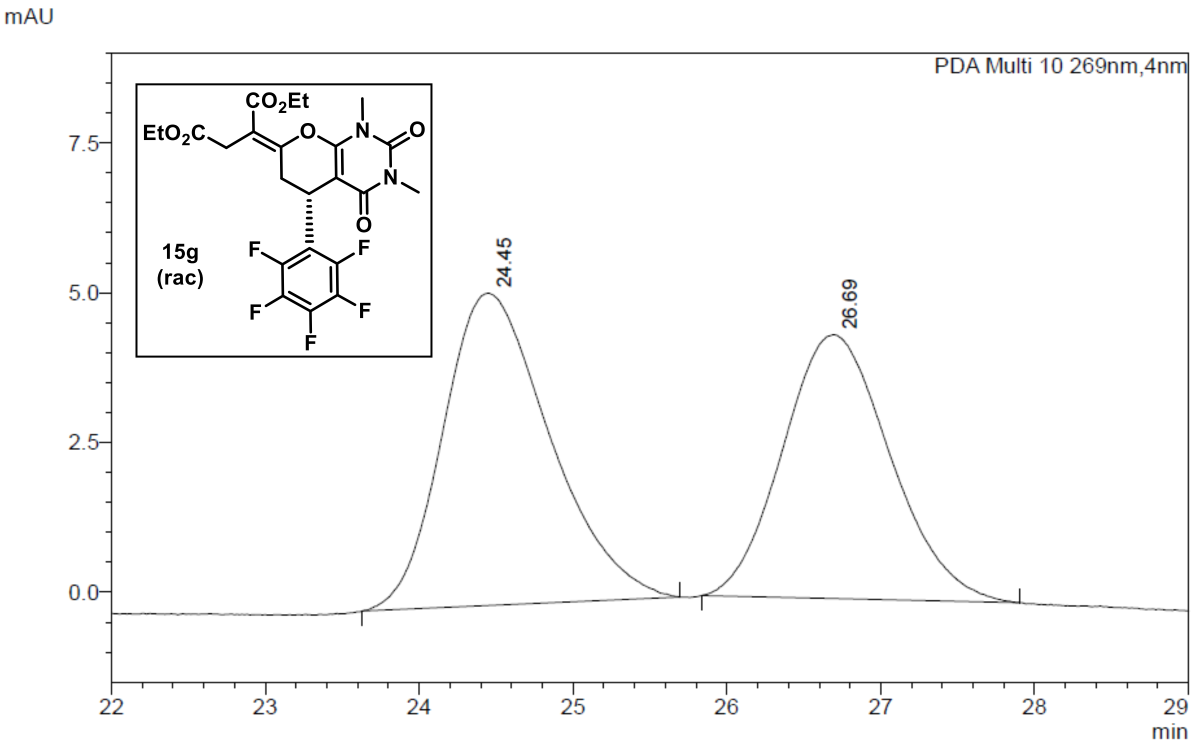

Peak Table

PDA Ch10 269nm

Peak#	Ret. Time	Area	Area%
1	24.45	251571	54.10
2	26.69	213473	45.90
Total		465044	100.00

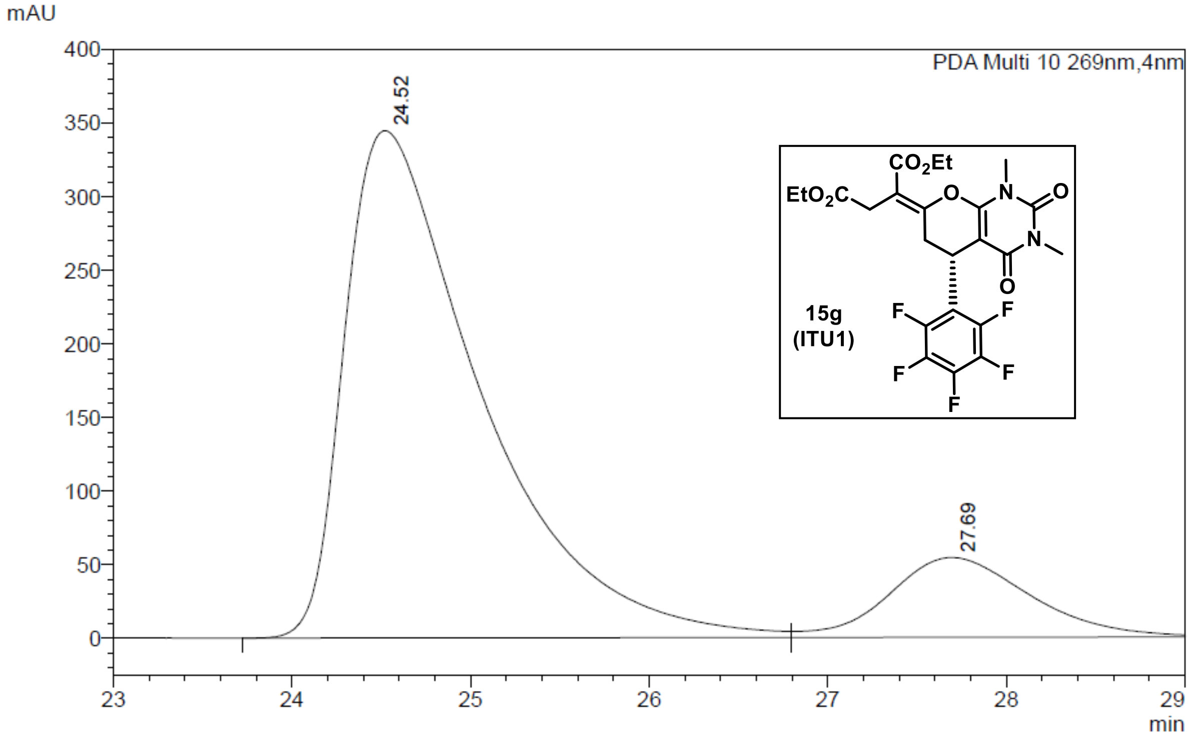

Peak Table

PDA Ch10 269nm

Peak#	Ret. Time	Area	Area%
1	24.52	18335657	86.01
2	27.69	2981188	13.99
Total		21316845	100.00

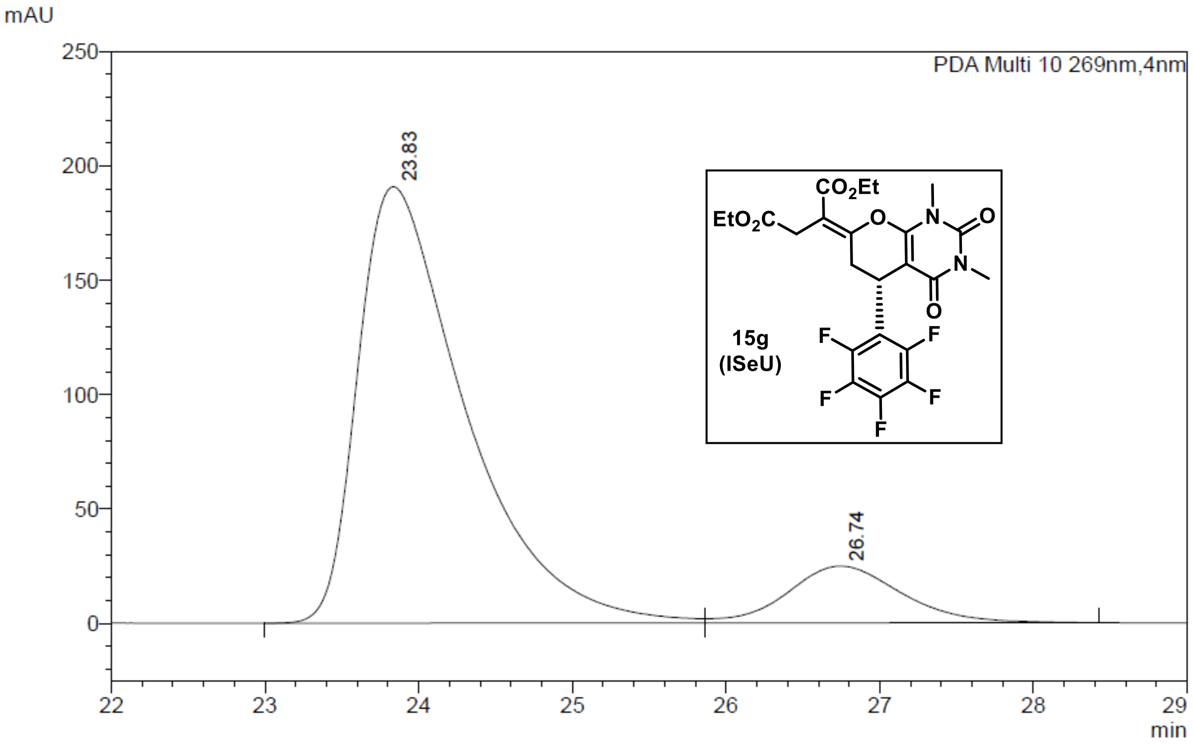

Peak Table

PDA Ch10 269nm

Peak#	Ret. Time	Area	Area%
1	23.83	9295710	87.91
2	26.74	1278909	12.09
Total		10574619	100.00

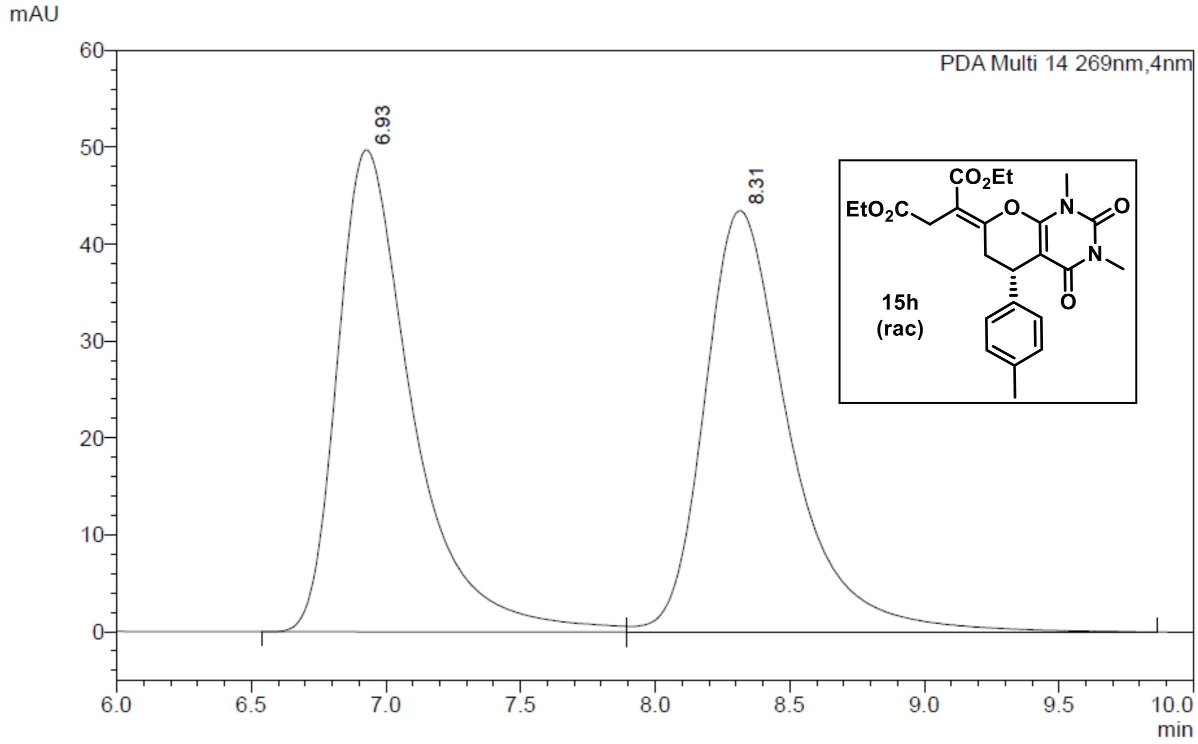

Peak Table

PDA Ch14 269nm

Peak#	Ret. Time	Area	Area%
1	6.93	965818	50.15
2	8.31	959945	49.85
Total		1925763	100.00

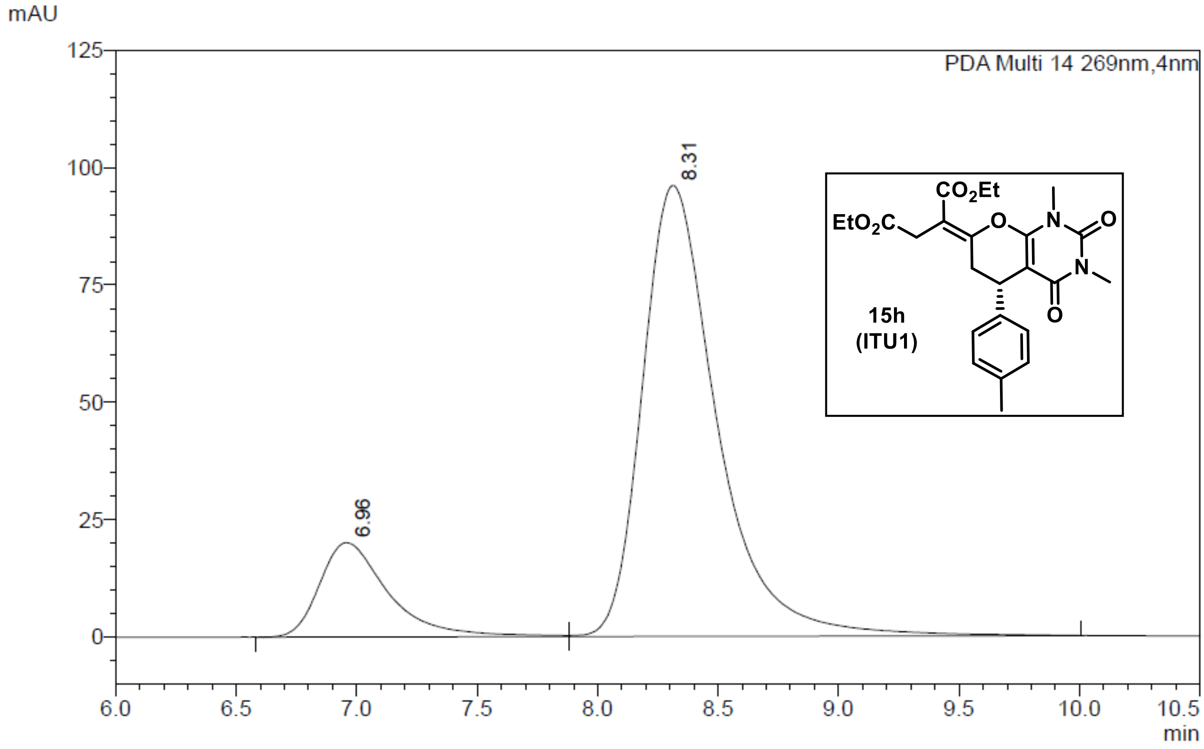

Peak Table

PDA Ch14 269nm

Peak#	Ret. Time	Area	Area%
1	6.96	398640	16.12
2	8.31	2074999	83.88
Total		2473638	100.00

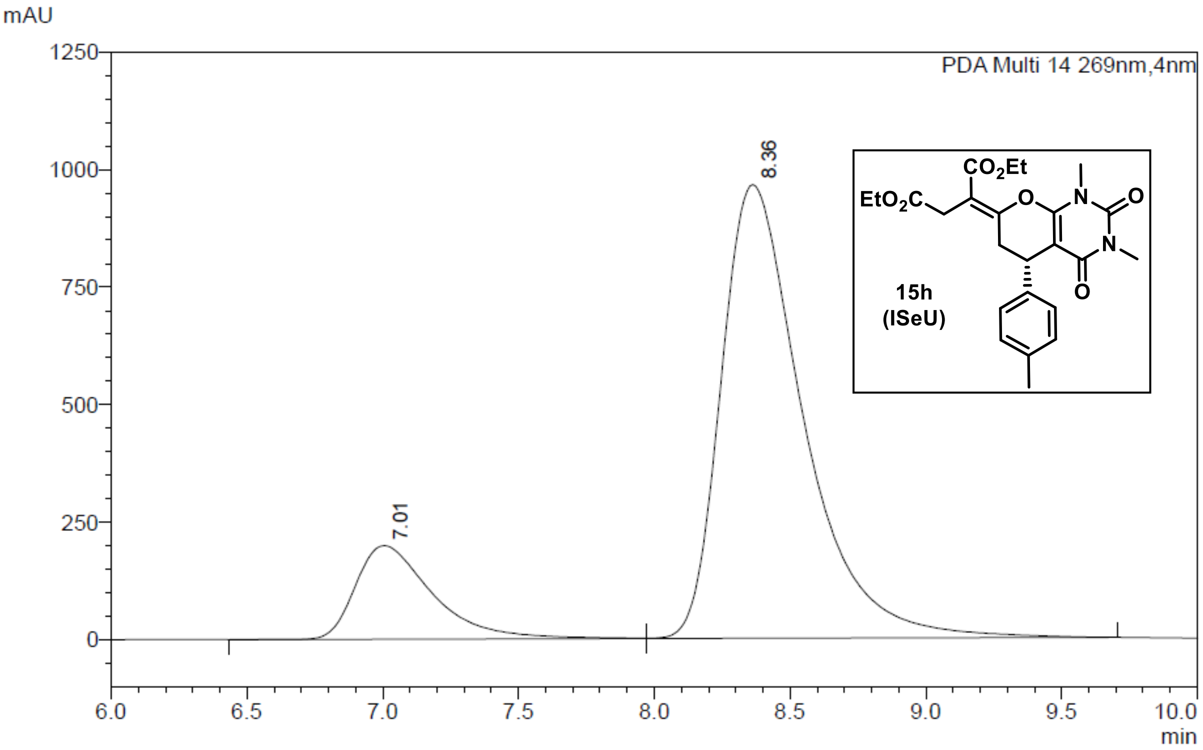

Peak Table

PDA Ch14 269nm

Peak#	Ret. Time	Area	Area%
1	7.01	3933177	16.18
2	8.36	20373793	83.82
Total		24306970	100.00

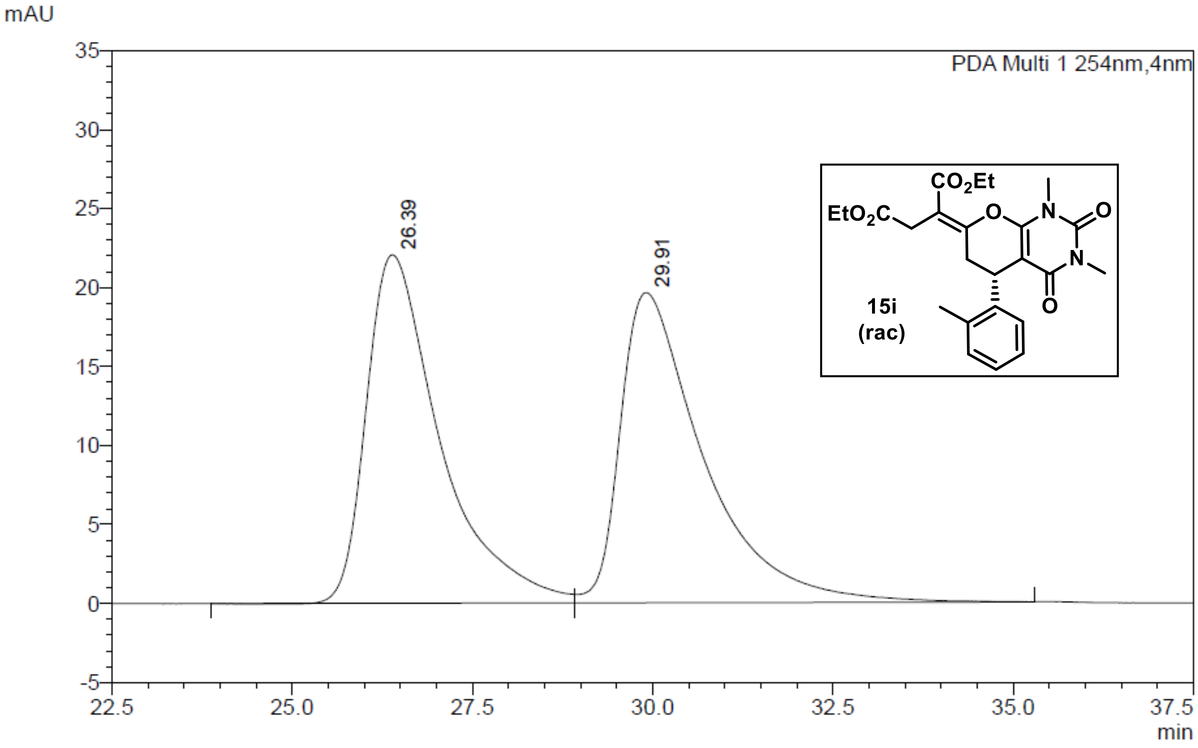

Peak Table

PDA Ch1 254nm

Peak#	Ret. Time	Area	Area%
1	26.39	1564865	49.85
2	29.91	1574111	50.15
Total		3138976	100.00

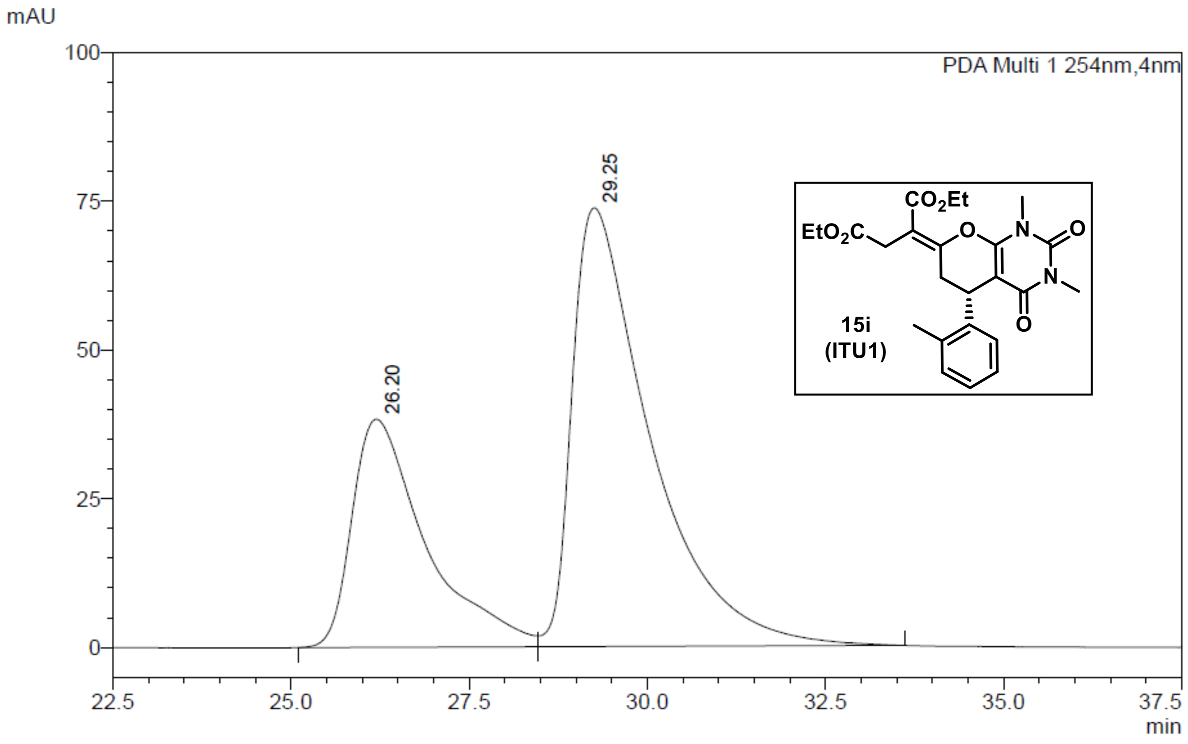

Peak Table

PDA Ch1 254nm

Peak#	Ret. Time	Area	Area%
1	26.20	2762098	32.93
2	29.25	5625160	67.07
Total		8387258	100.00

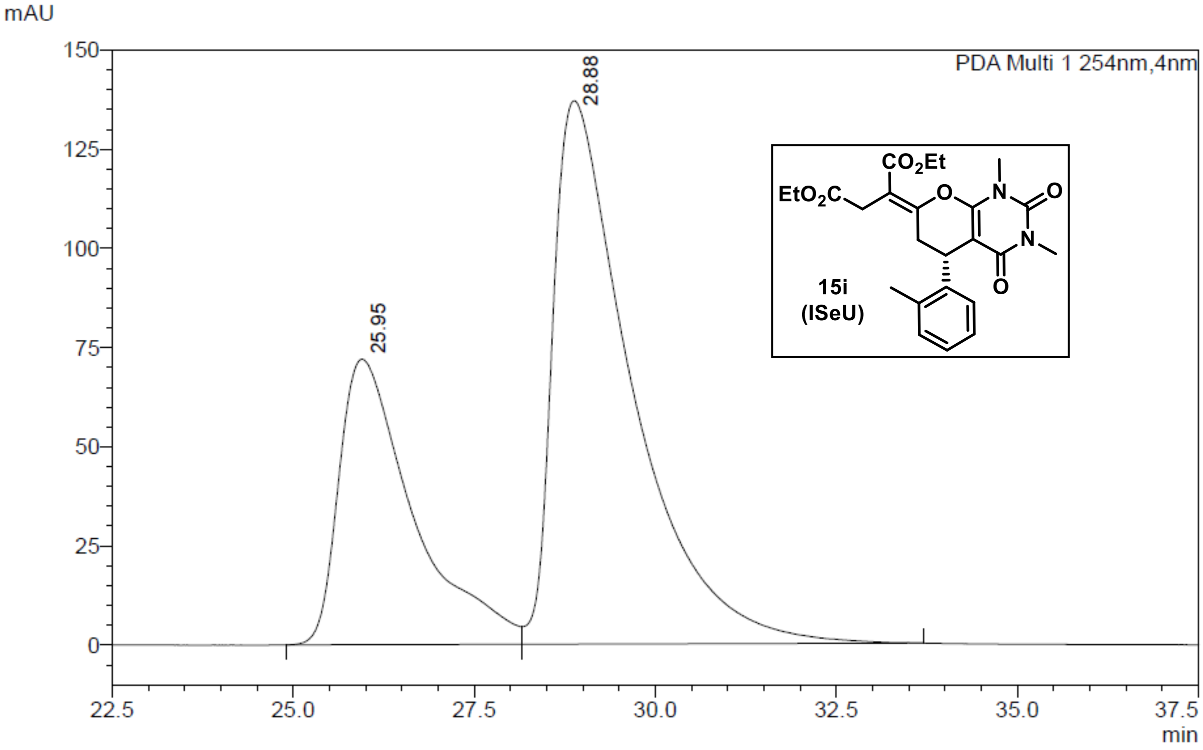

Peak Table

PDA Ch1 254nm

Peak#	Ret. Time	Area	Area%
1	25.95	5140419	32.96
2	28.88	10456308	67.04
Total		15596727	100.00

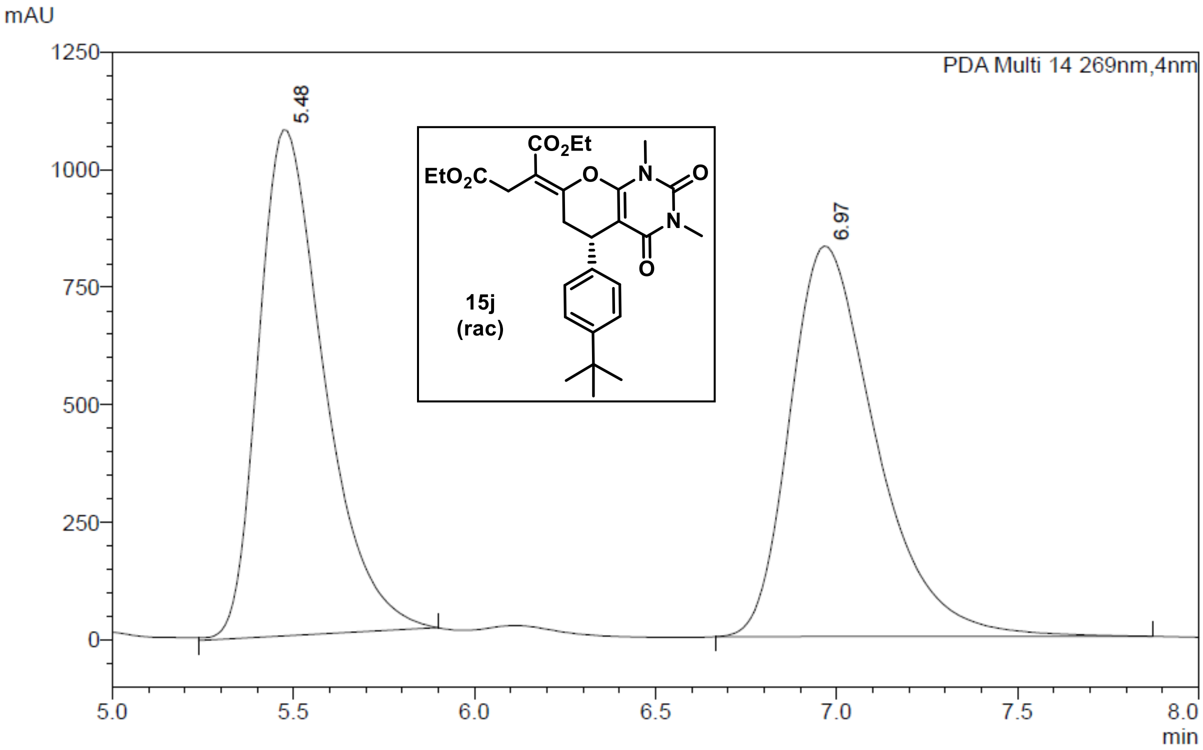

Peak Table

PDA Ch14 269nm

Peak#	Ret. Time	Area	Area%
1	5.48	13615216	49.65
2	6.97	13807882	50.35
Total		27423098	100.00

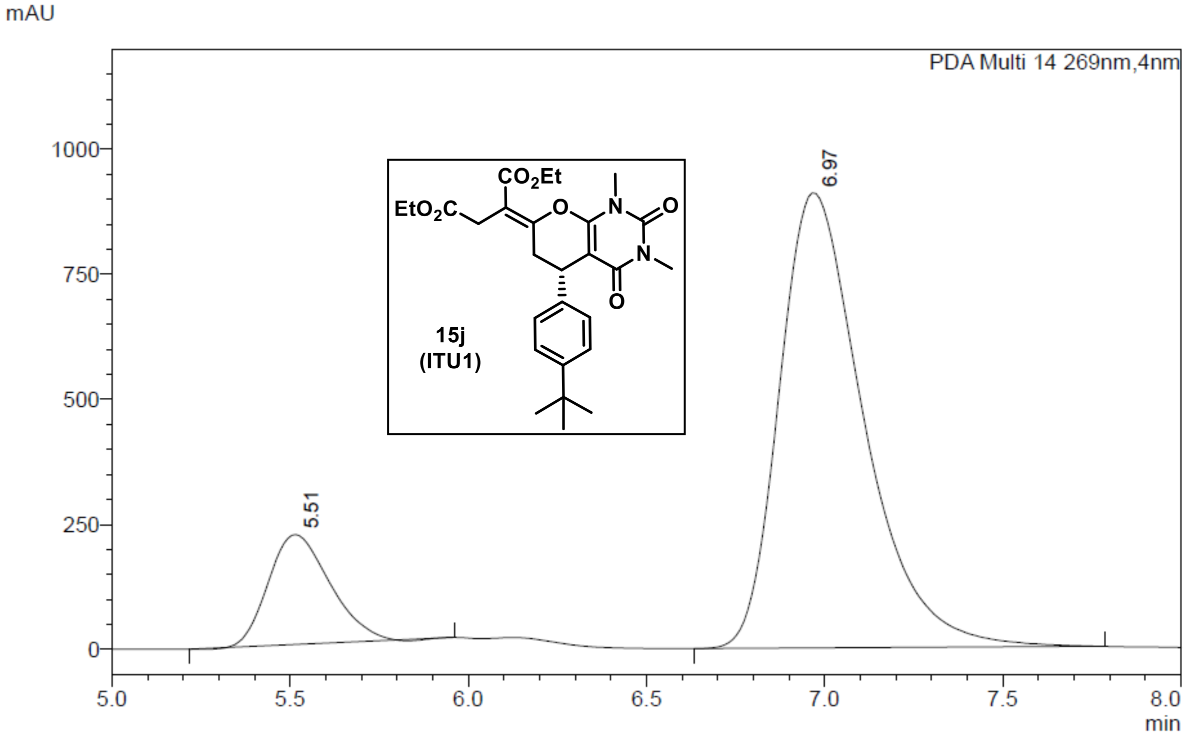

Peak Table

PDA Ch14 269nm

Peak#	Ret. Time	Area	Area%
1	5.51	2635608	14.94
2	6.97	15004000	85.06
Total		17639608	100.00

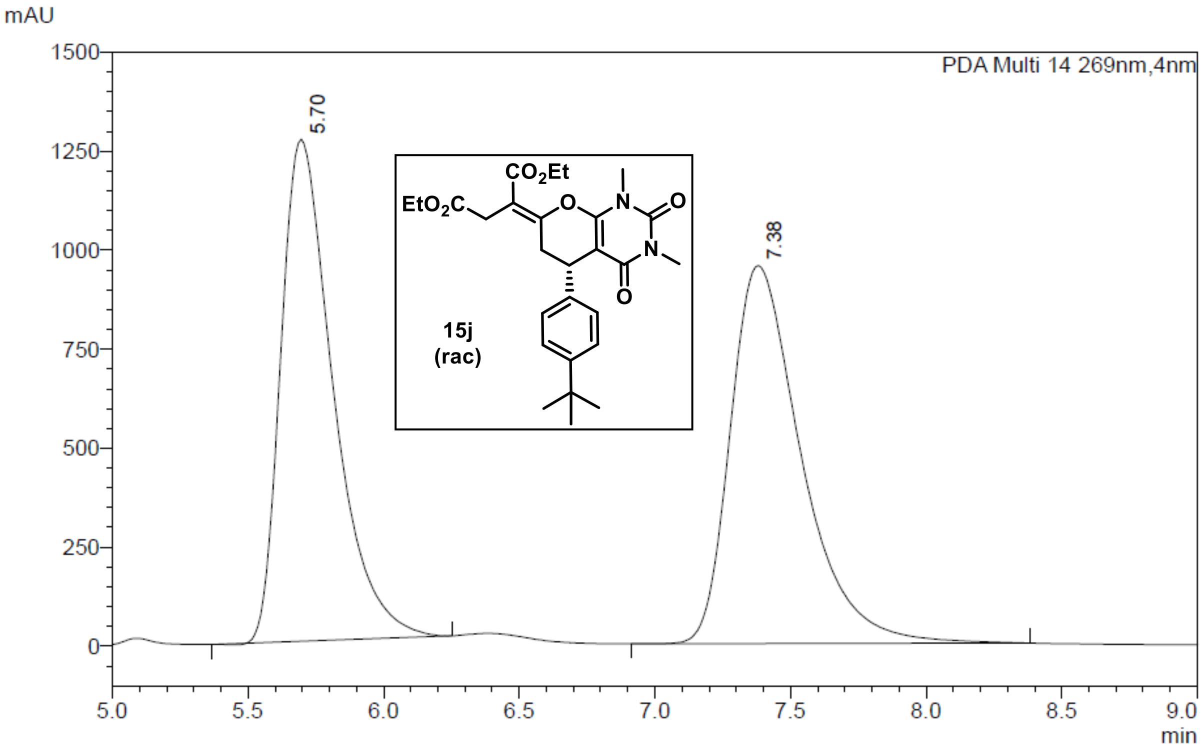

Peak Table

PDA Ch14 269nm

Peak#	Ret. Time	Area	Area%
1	5.70	16965319	49.45
2	7.38	17346086	50.55
Total		34311404	100.00

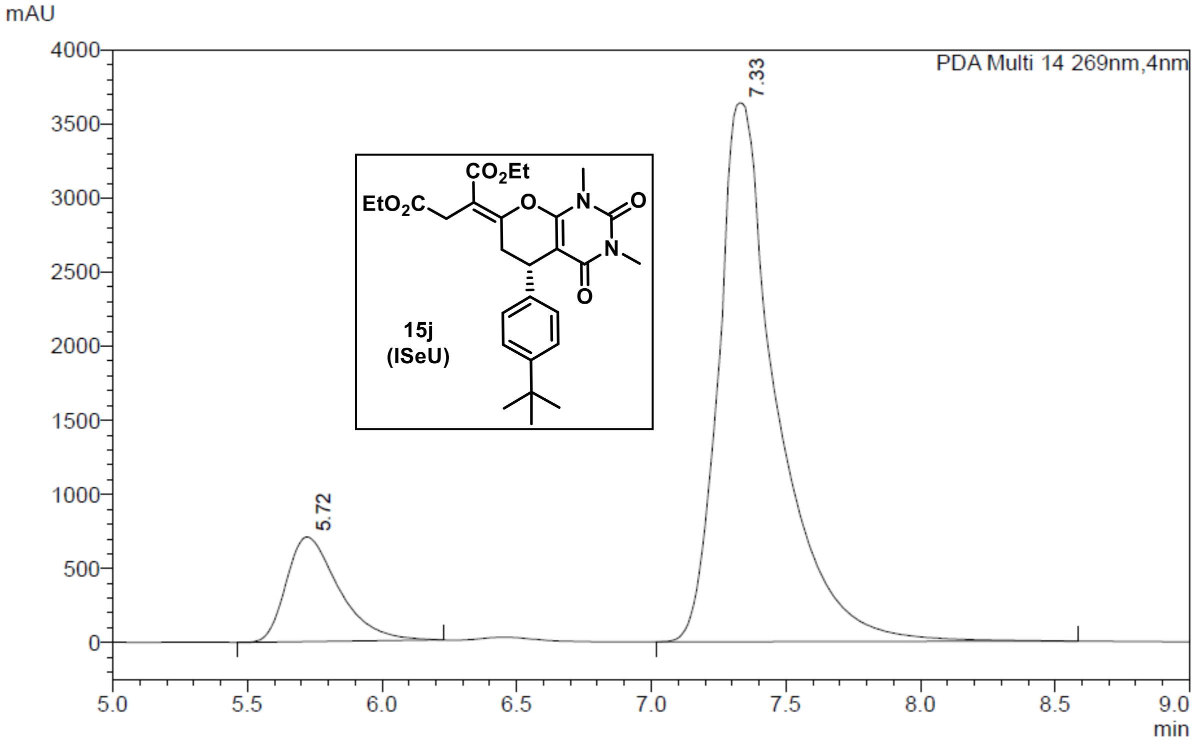

Peak Table

PDA Ch14 269nm

Peak#	Ret. Time	Area	Area%
1	5.72	9588605	14.88
2	7.33	54867827	85.12
Total		64456432	100.00

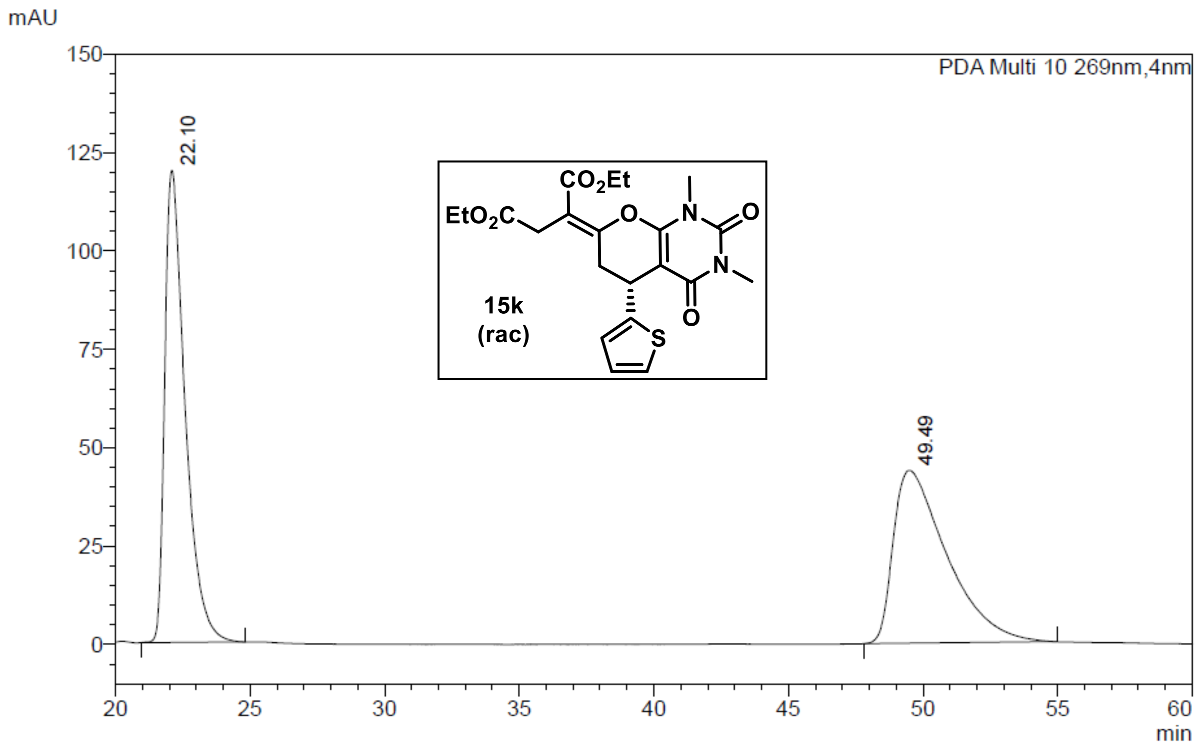

Peak Table

PDA Ch10 269nm

Peak#	Ret. Time	Area	Area%
1	22.10	6295333	50.88
2	49.49	6076961	49.12
Total		12372293	100.00

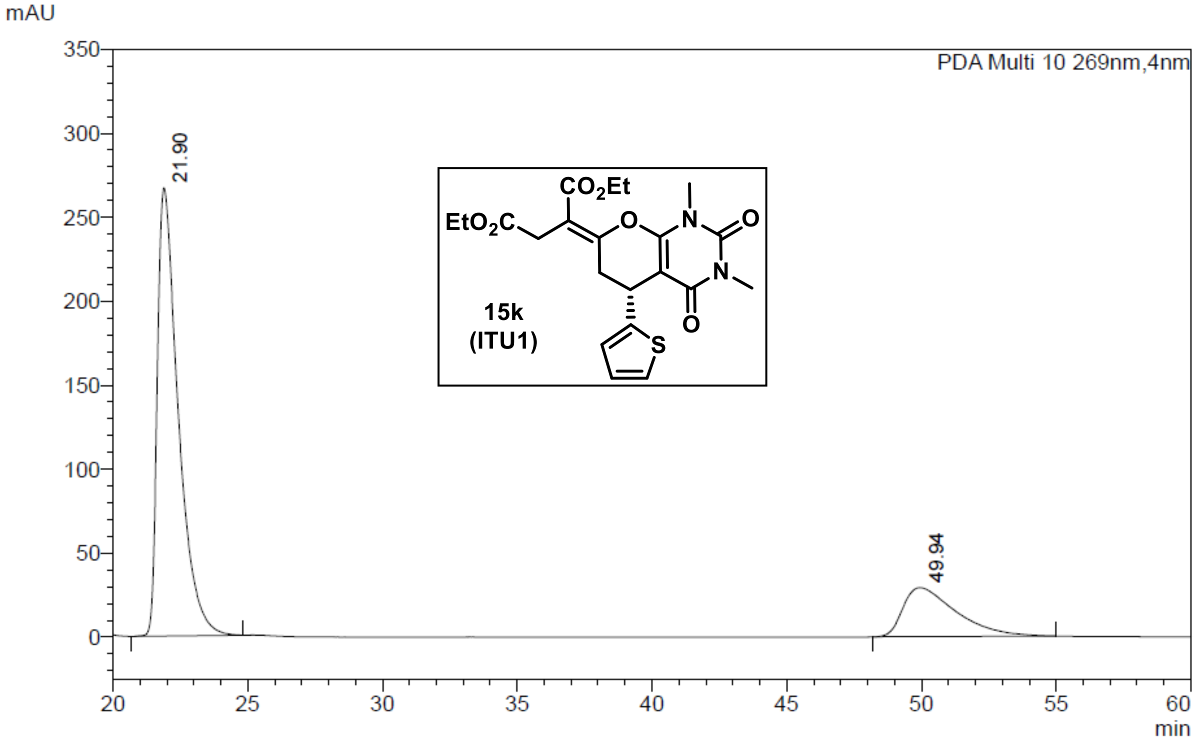

Peak Table

PDA Ch10 269nm

Peak#	Ret. Time	Area	Area%
1	21.90	14361175	78.32
2	49.94	3976317	21.68
Total		18337492	100.00

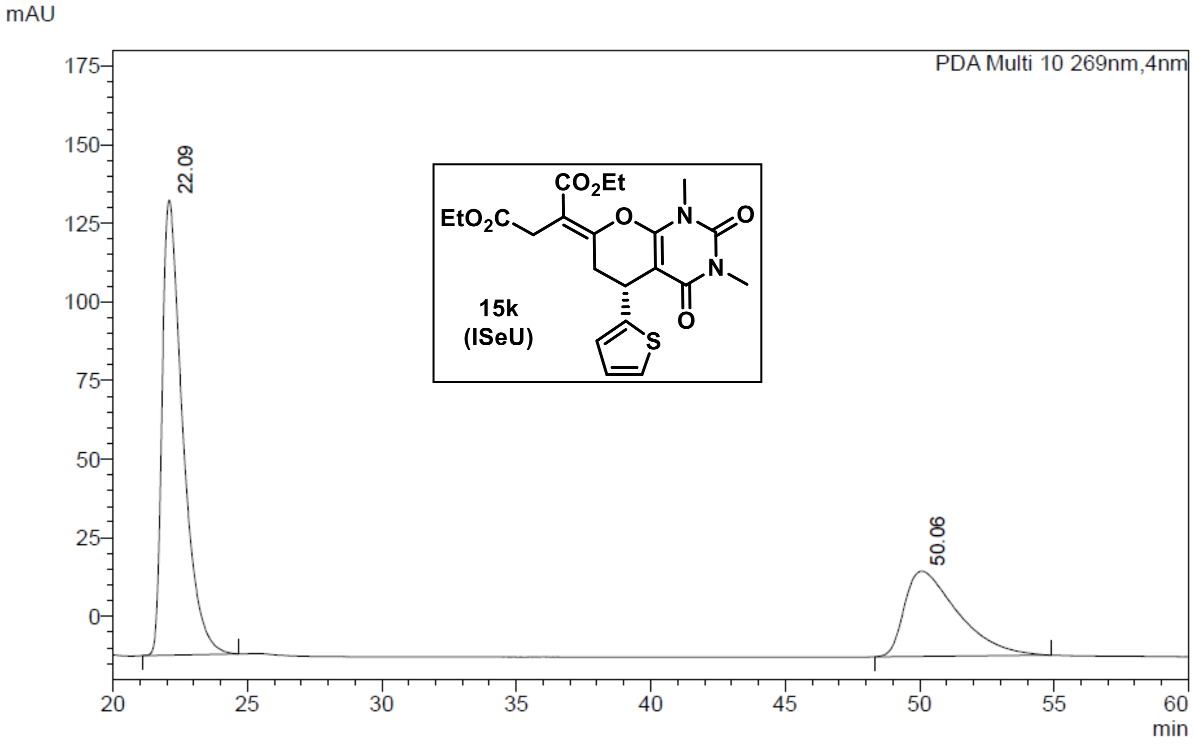

Peak Table

PDA Ch10 269nm

Peak#	Ret. Time	Area	Area%
1	22.09	7701601	67.35
2	50.06	3733669	32.65
Total		11435270	100.00

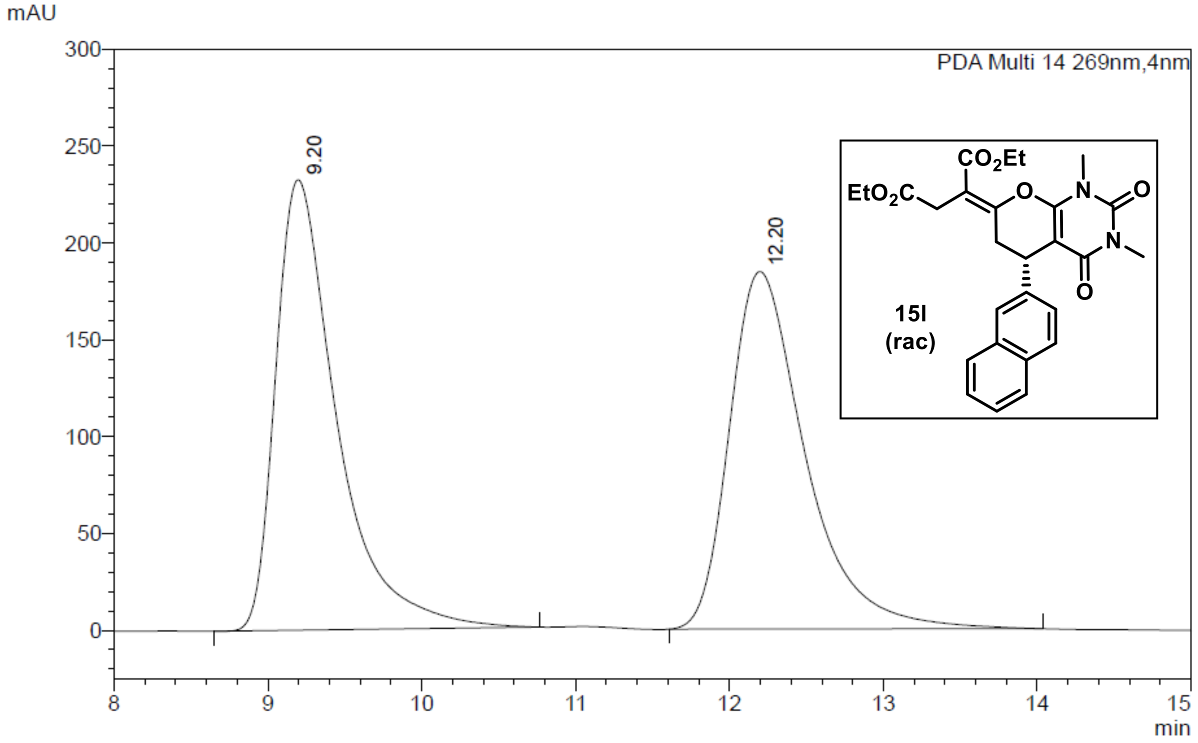

Peak Table

PDA Ch14 269nm

Peak#	Ret. Time	Area	Area%
1	9.20	6437208	50.37
2	12.20	6342308	49.63
Total		12779516	100.00

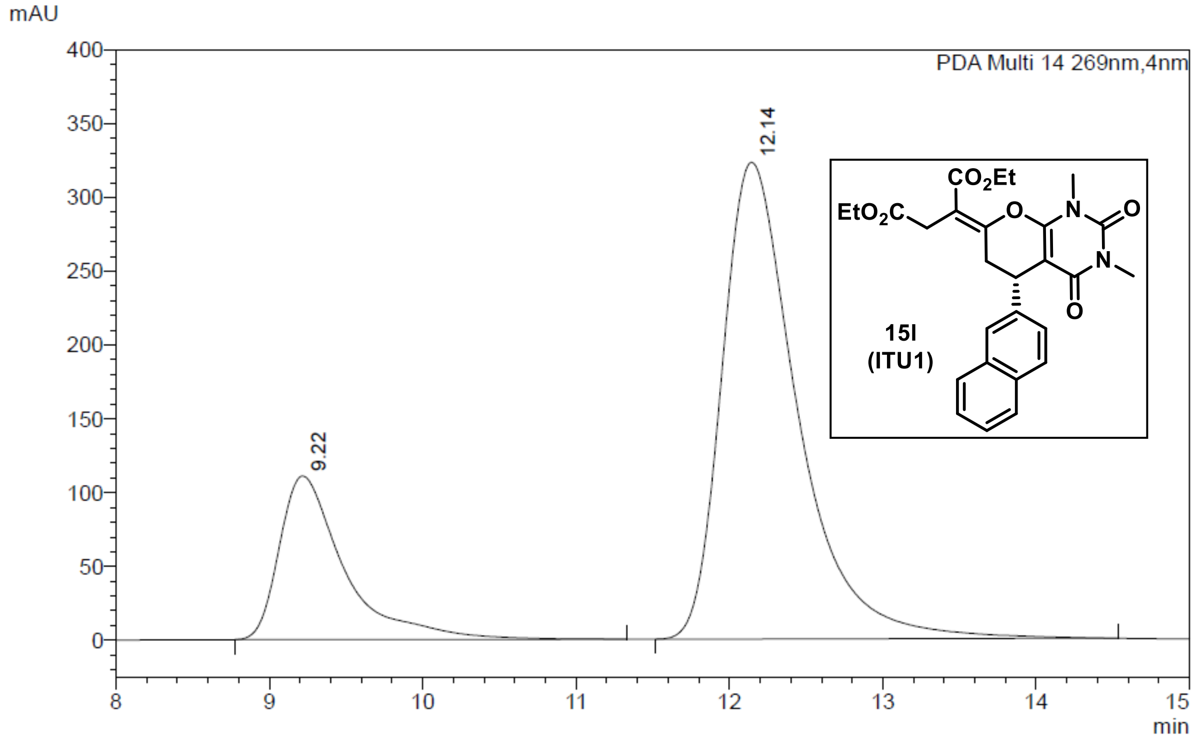

Peak Table

PDA Ch14 269nm

Peak#	Ret. Time	Area	Area%
1	9.22	3279953	22.81
2	12.14	11098815	77.19
Total		14378768	100.00

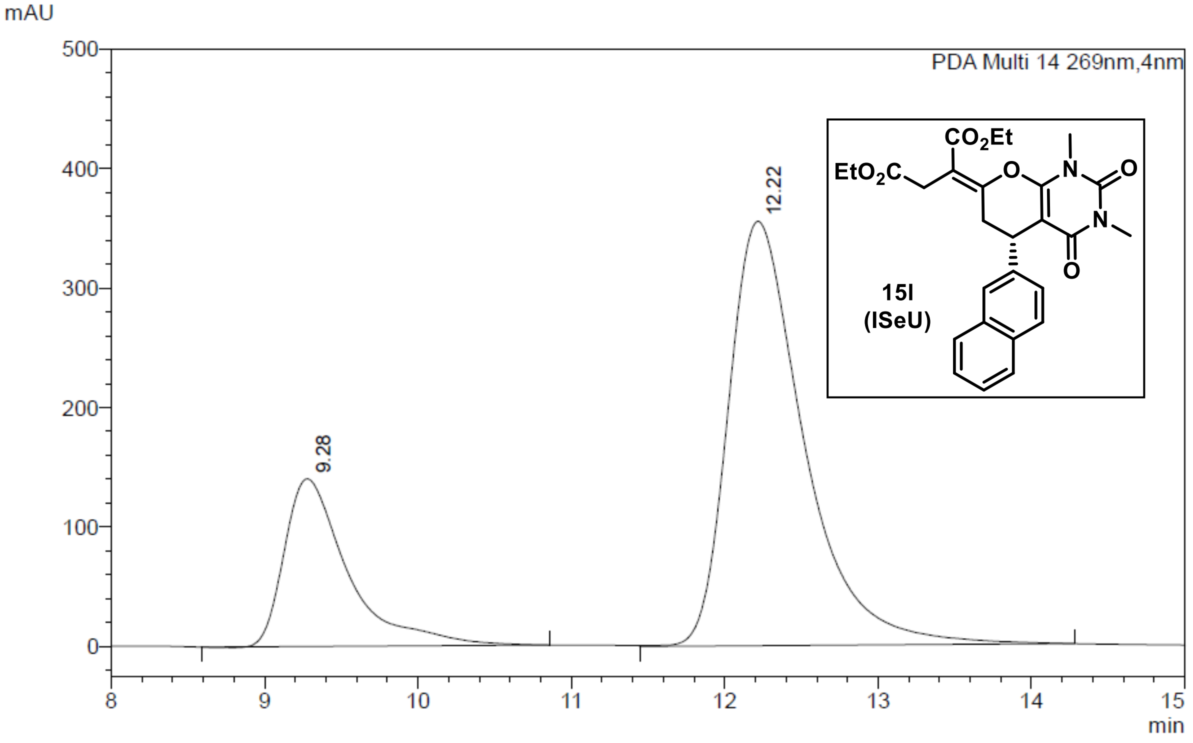

Peak Table

PDA Ch14 269nm

Peak#	Ret. Time	Area	Area%
1	9.28	4053938	24.94
2	12.22	12199369	75.06
Total		16253307	100.00

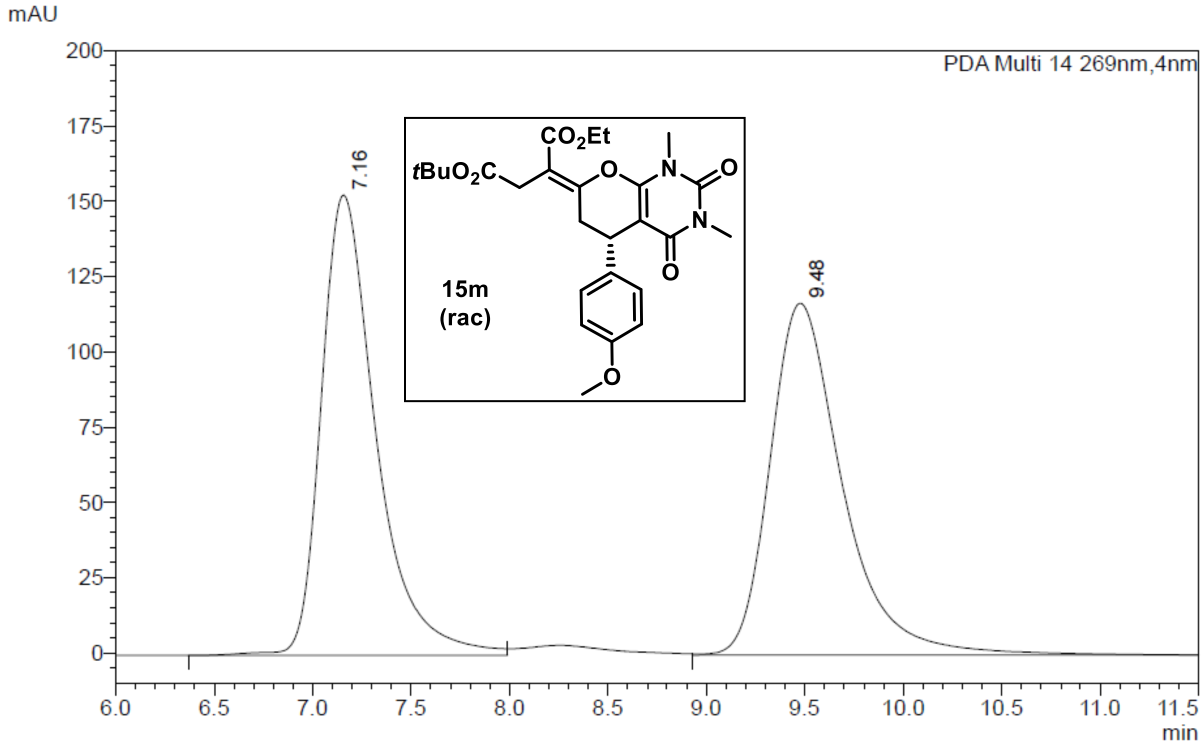

Peak Table

PDA Ch14 269nm

Peak#	Ret. Time	Area	Area%
1	7.16	2963898	49.69
2	9.48	3000867	50.31
Total		5964765	100.00

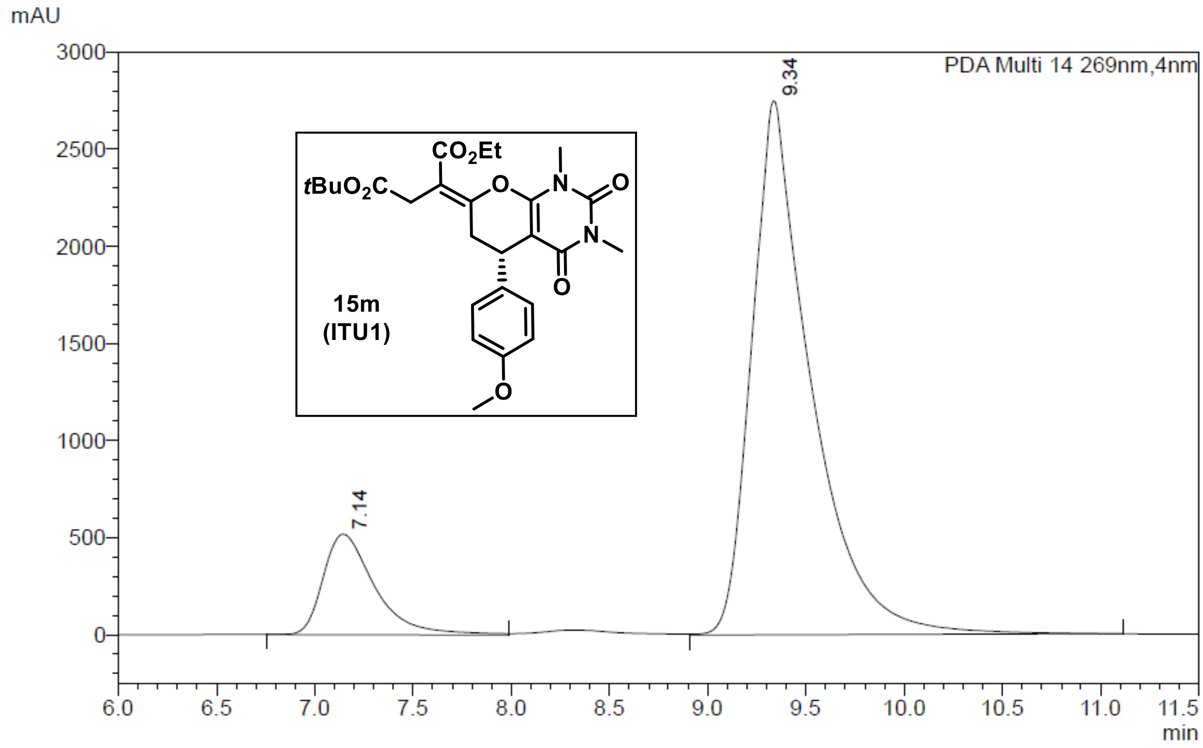

Peak Table

PDA Ch14 269nm

Peak#	Ret. Time	Area	Area%
1	7.14	9509081	14.13
2	9.34	57810265	85.87
Total		67319346	100.00

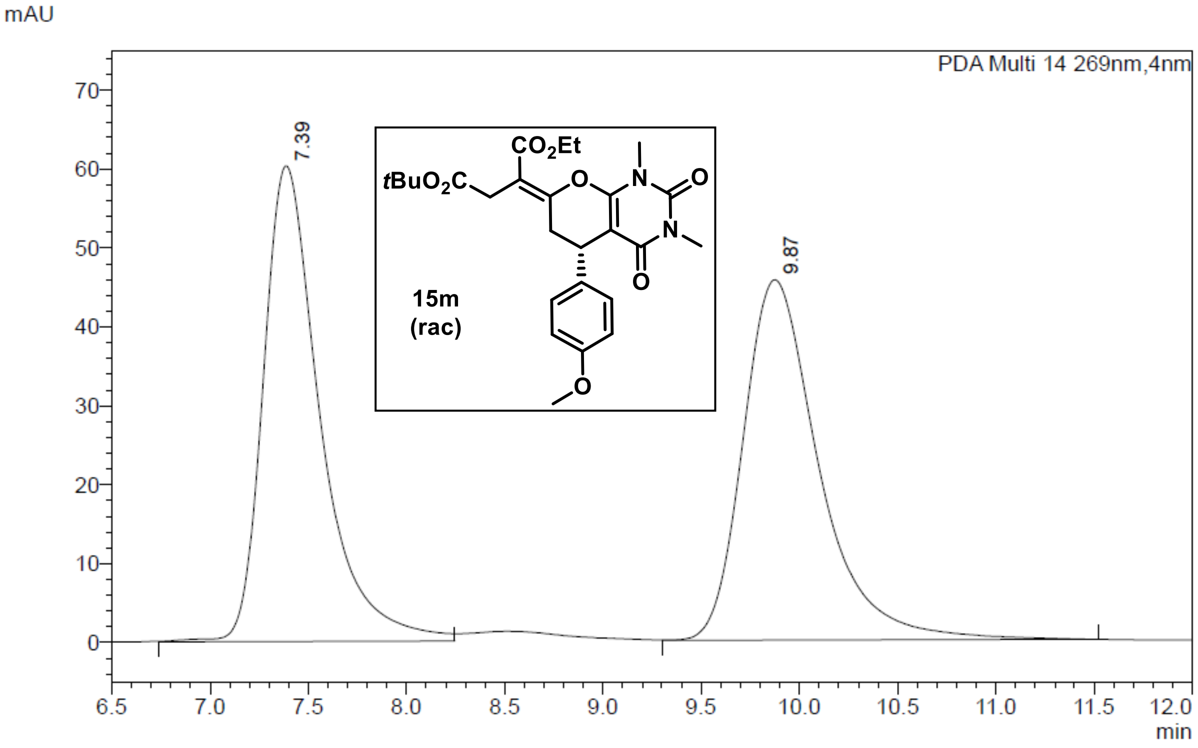

Peak Table

PDA Ch14 269nm

Peak#	Ret. Time	Area	Area%
1	7.39	1217022	49.81
2	9.87	1226489	50.19
Total		2443511	100.00

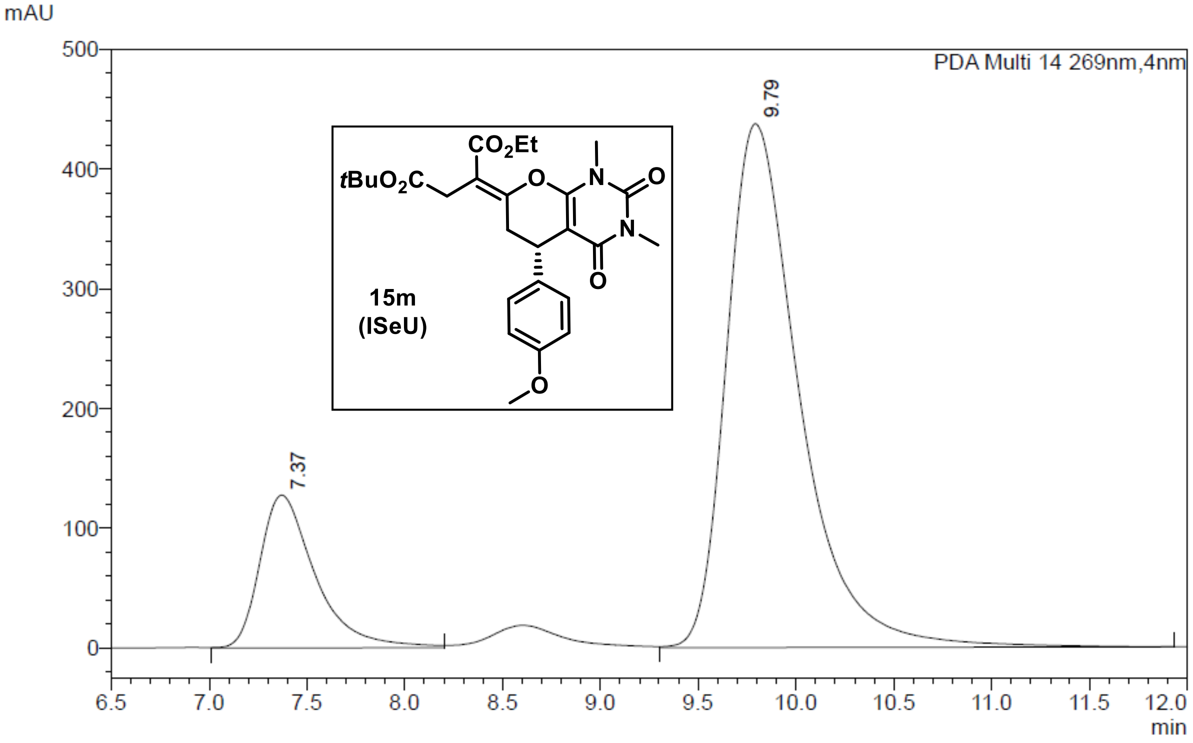

Peak Table

PDA Ch14 269nm

Peak#	Ret. Time	Area	Area%
1	7.37	2521915	18.19
2	9.79	11341462	81.81
Total		13863377	100.00

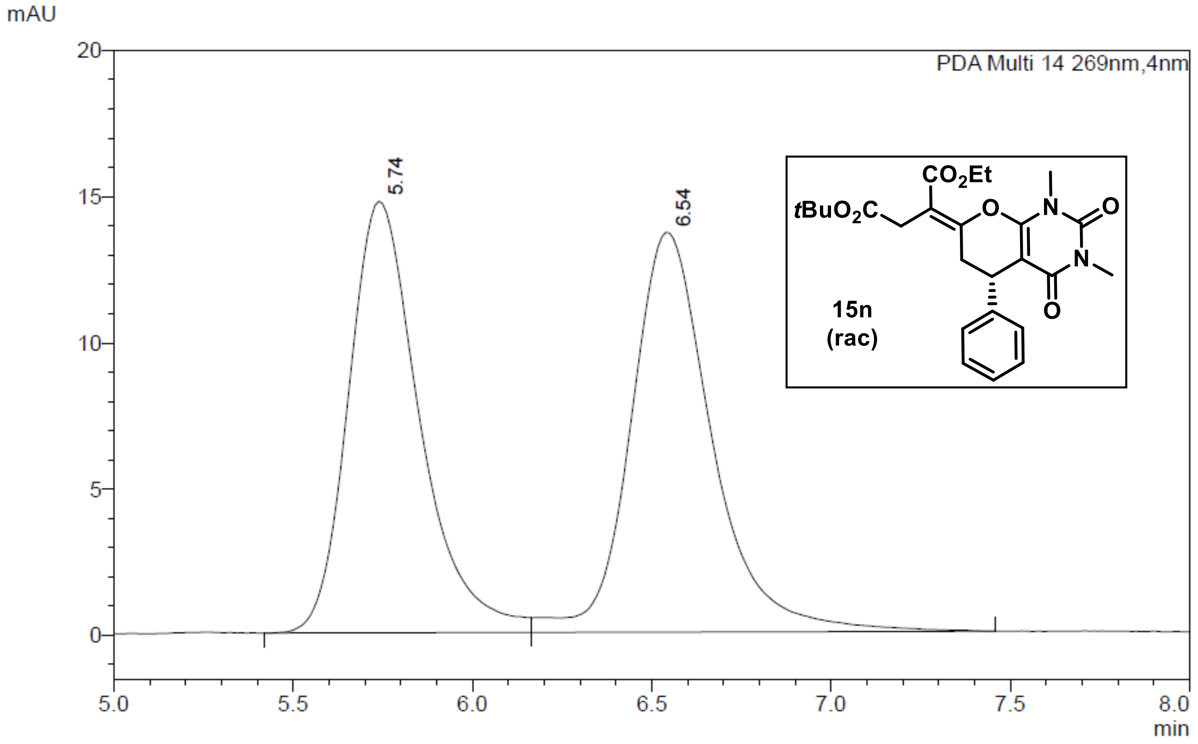

Peak Table

PDA Ch14 269nm

Peak#	Ret. Time	Area	Area%
1	5.74	204083	48.54
2	6.54	216352	51.46
Total		420435	100.00

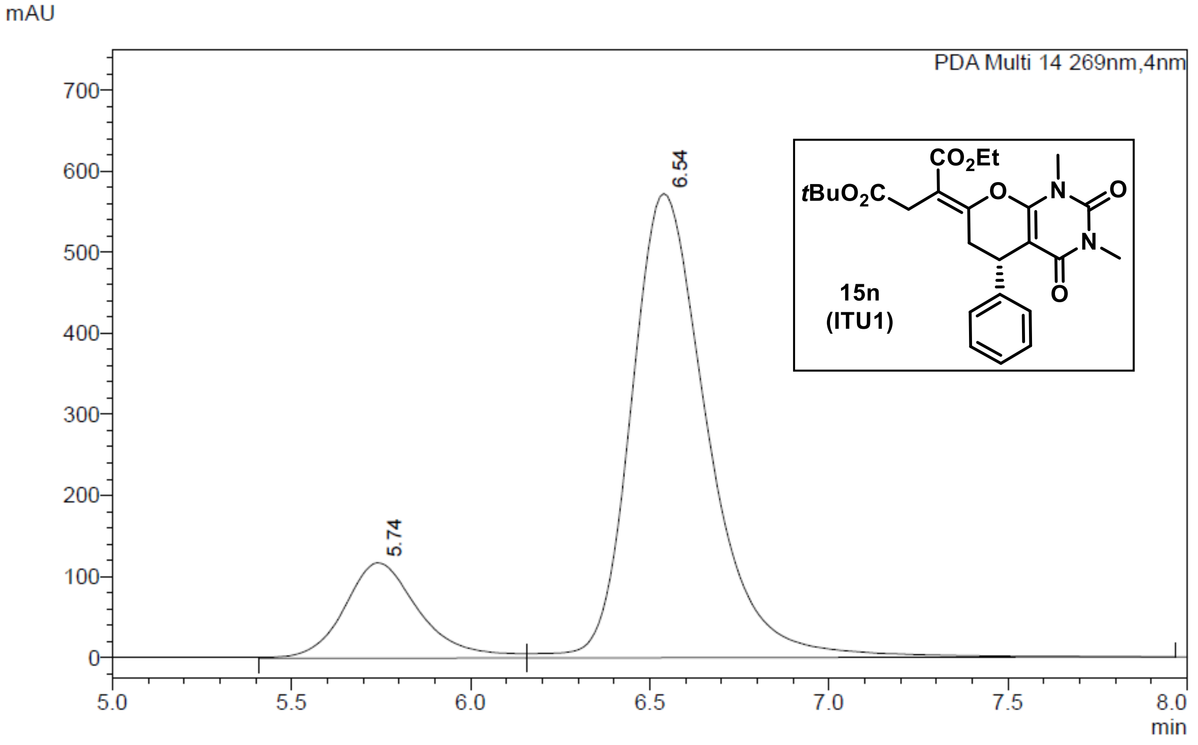

Peak Table

PDA Ch14 269nm

Peak#	Ret. Time	Area	Area%
1	5.74	1716944	16.78
2	6.54	8513926	83.22
Total		10230870	100.00

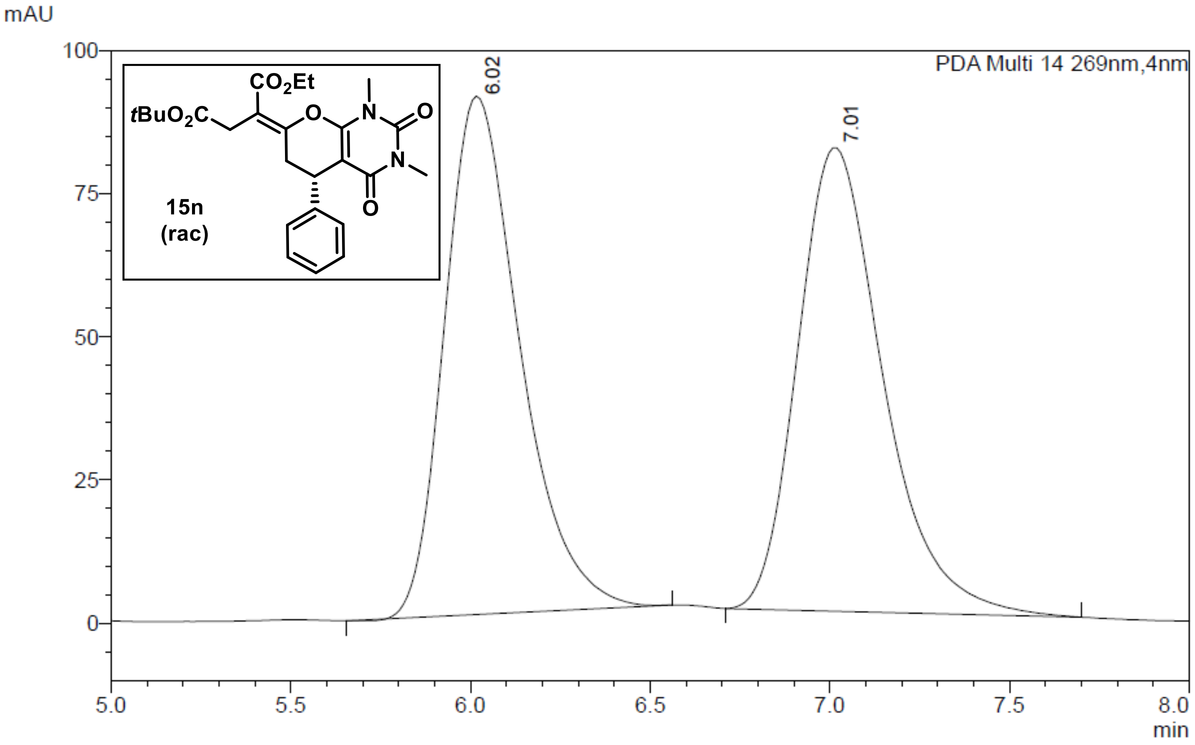

Peak Table

PDA Ch14 269nm

Peak#	Ret. Time	Area	Area%
1	6.02	1320673	49.67
2	7.01	1338200	50.33
Total		2658873	100.00

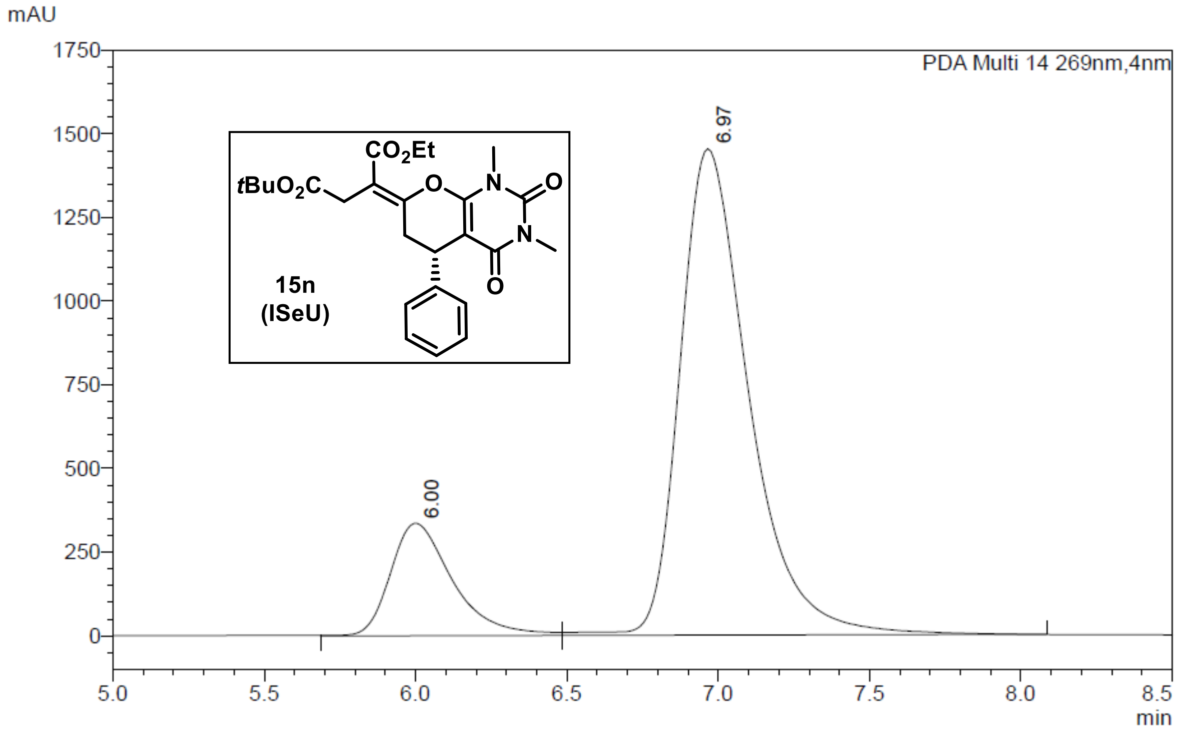

Peak Table

PDA Ch14 269nm

Peak#	Ret. Time	Area	Area%
1	6.00	4824277	17.29
2	6.97	23075683	82.71
Total		27899960	100.00

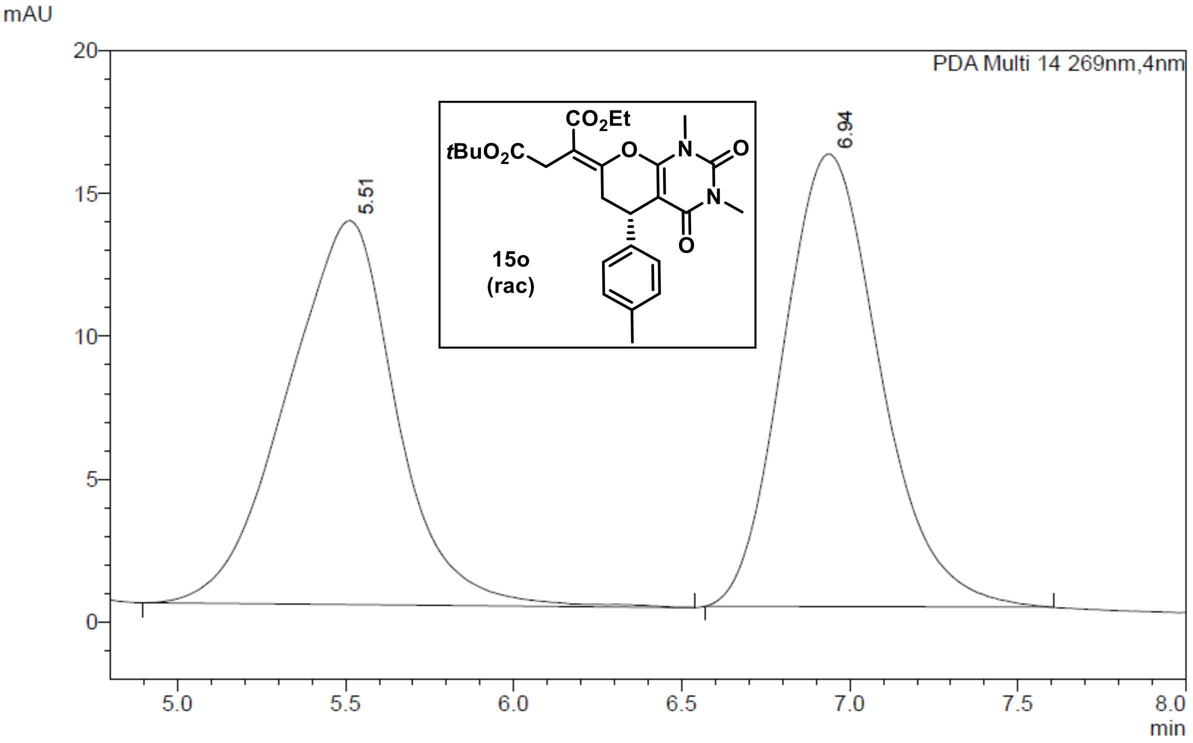

Peak Table

PDA Ch14 269nm

Peak#	Ret. Time	Area	Area%
1	5.51	306873	49.02
2	6.94	319159	50.98
Total		626032	100.00

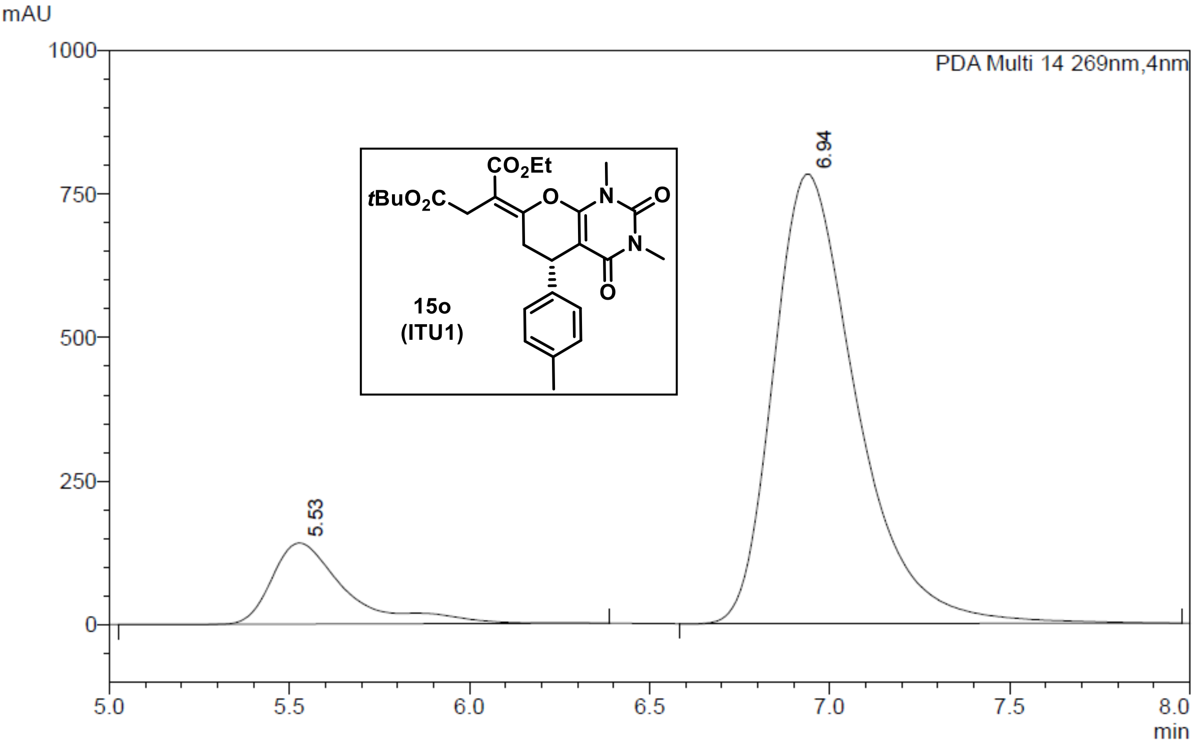

Peak Table

PDA Ch14 269nm

Peak#	Ret. Time	Area	Area%
1	5.53	2040757	13.95
2	6.94	12590279	86.05
Total		14631035	100.00

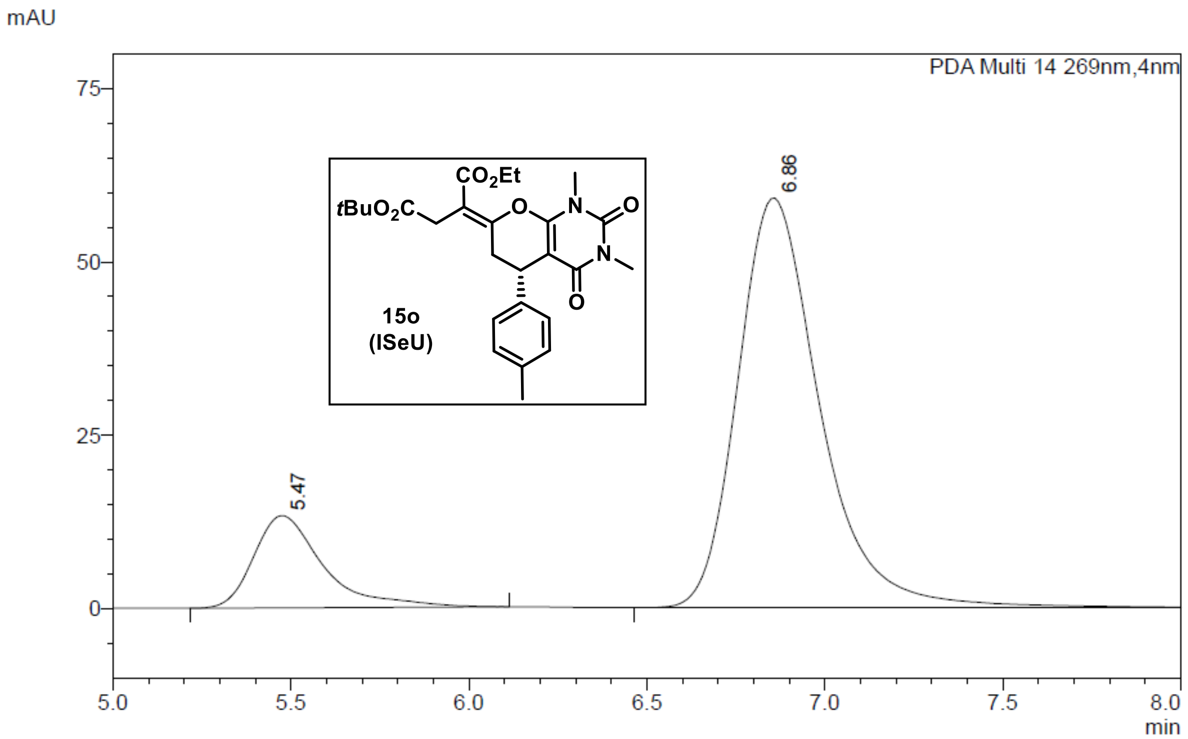

Peak Table

PDA Ch14 269nm

Peak#	Ret. Time	Area	Area%
1	5.47	180387	16.00
2	6.86	947091	84.00
Total		1127478	100.00

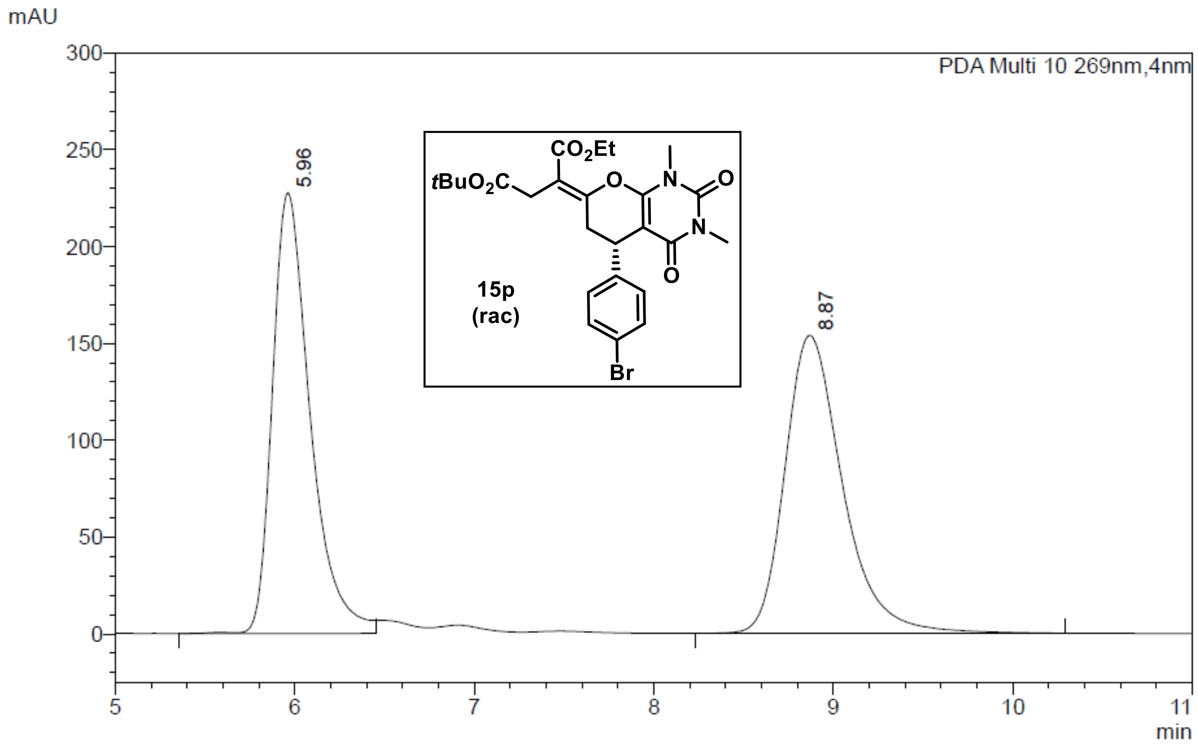

Peak Table

PDA Ch10 269nm

Peak#	Ret. Time	Area	Area%
1	5.96	3332606	49.25
2	8.87	3434534	50.75
Total		6767140	100.00

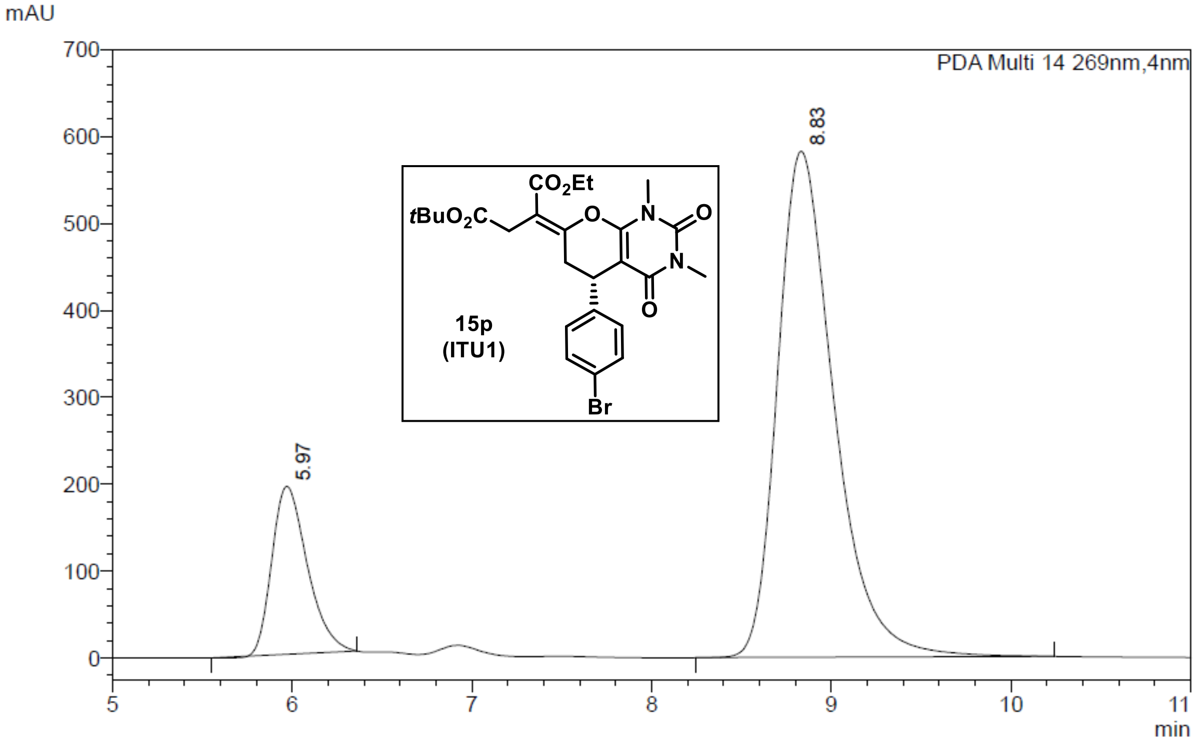

Peak Table

PDA Ch14 269nm

Peak#	Ret. Time	Area	Area%
1	5.97	2643309	17.17
2	8.83	12750800	82.83
Total		15394109	100.00

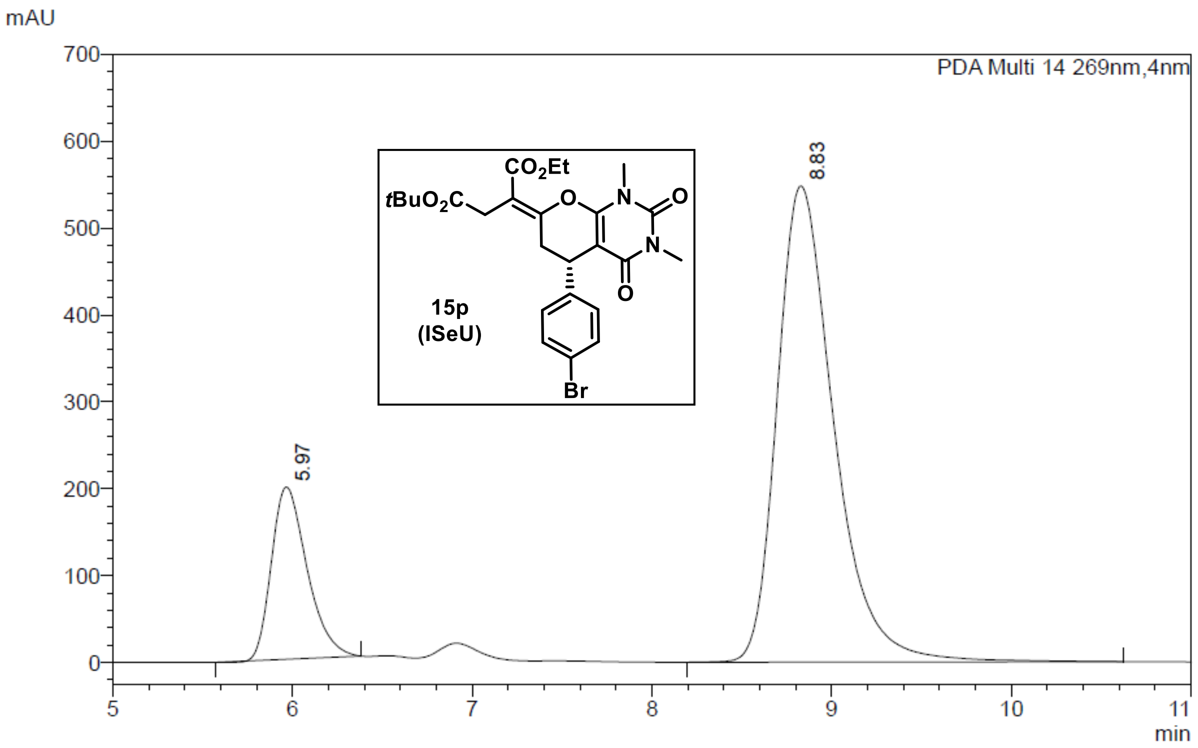

Peak Table

PDA Ch14 269nm

Peak#	Ret. Time	Area	Area%
1	5.97	2731107	18.49
2	8.83	12040184	81.51
Total		14771292	100.00

11.5 HPLC chromatograms of the product diversification

Chromatogram

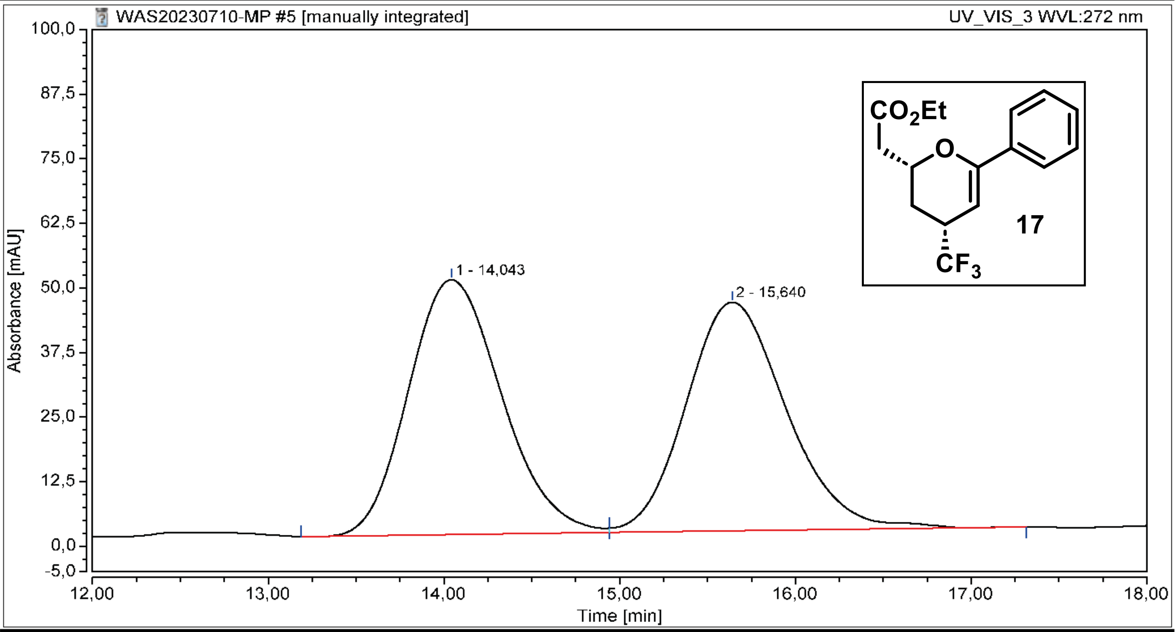

Integration Results

No.	Peak Name	Retention Time min	Area mAU*min	Height mAU	Relative Area %	Relative Height %	Amount n.a.
1		14,043	30,285	49,325	50,86	52,70	n.a.
2		15,640	29,258	44,265	49,14	47,30	n.a.
**Total:**			**59,543**	**93,590**	**100,00**	**100,00**	

Chromatogram

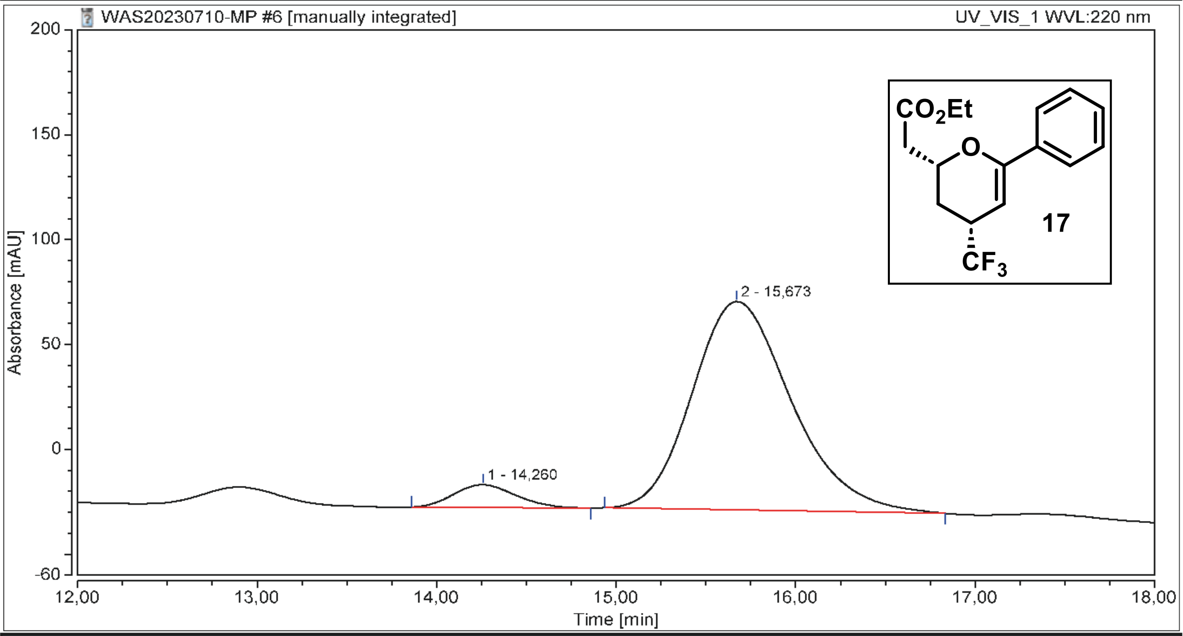

Integration Results

No.	Peak Name	Retention Time min	Area mAU*min	Height mAU	Relative Area %	Relative Height %	Amount n.a.
1		14,260	4,327	10,809	6,36	9,82	n.a.
2		15,673	63,707	99,297	93,64	90,18	n.a.
**Total:**			**68,033**	**110,107**	**100,00**	**100,00**	

Chromatogram

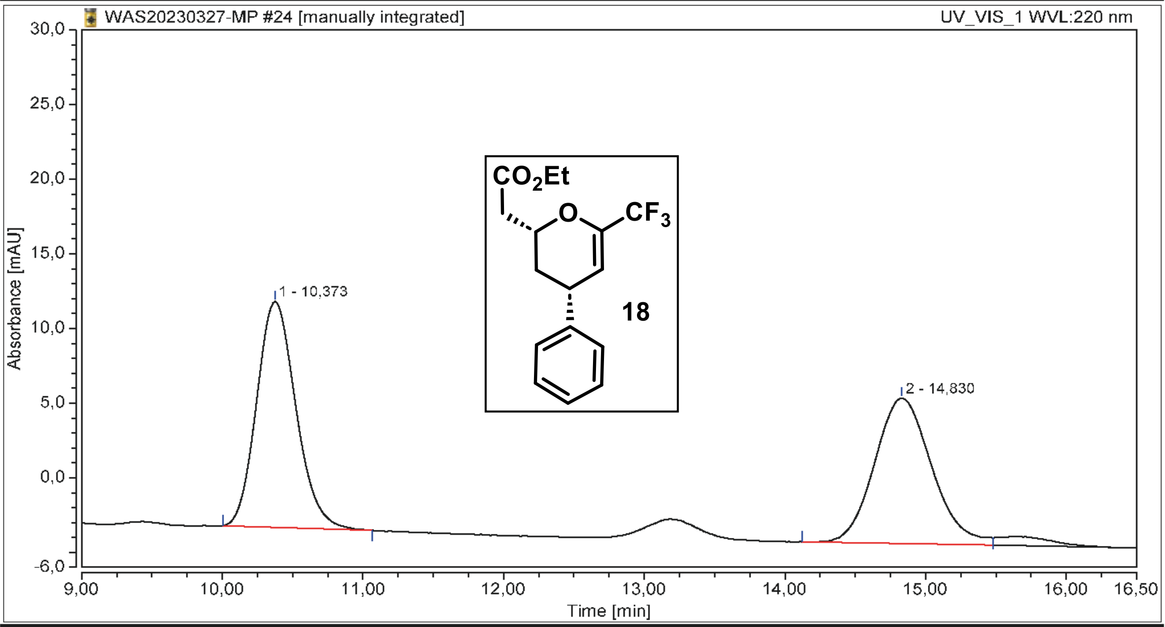

Integration Results

No.	Peak Name	Retention Time min	Area mAU*min	Height mAU	Relative Area %	Relative Height %	Amount n.a.
1		10,373	4,871	15,126	51,59	60,87	n.a.
2		14,830	4,571	9,723	48,41	39,13	n.a.
**Total:**			**9,442**	**24,849**	**100,00**	**100,00**	

Chromatogram

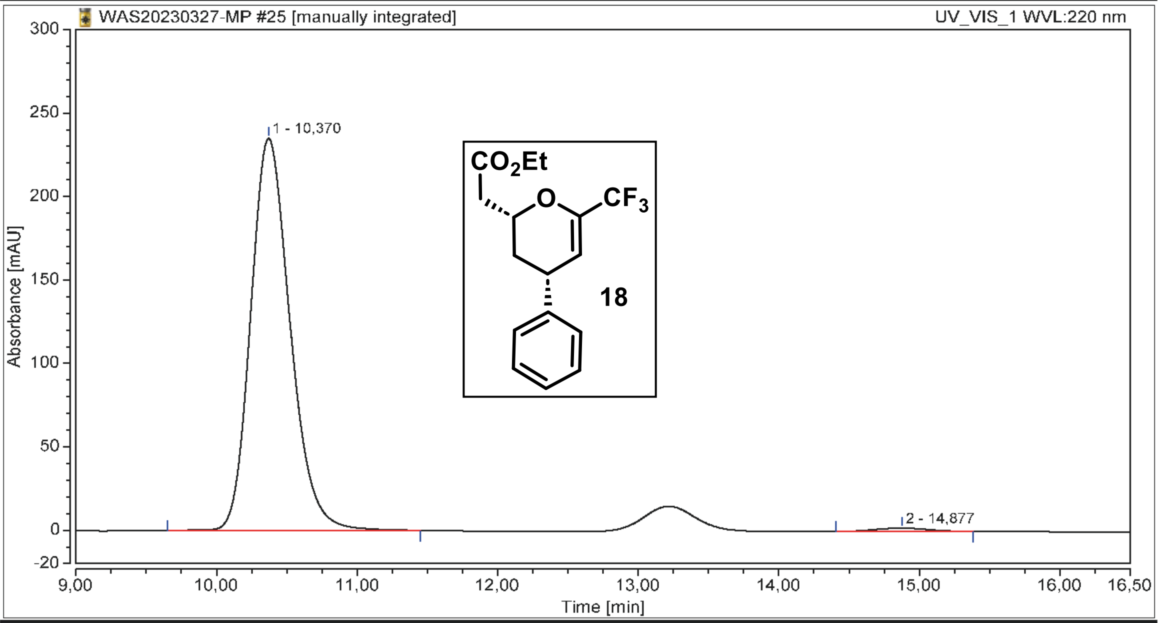

Integration Results

No.	Peak Name	Retention Time min	Area mAU*min	Height mAU	Relative Area %	Relative Height %	Amount n.a.
1		10,370	76,428	235,268	98,83	99,13	n.a.
2		14,877	0,904	2,073	1,17	0,87	n.a.
**Total:**			**77,331**	**237,342**	**100,00**	**100,00**	

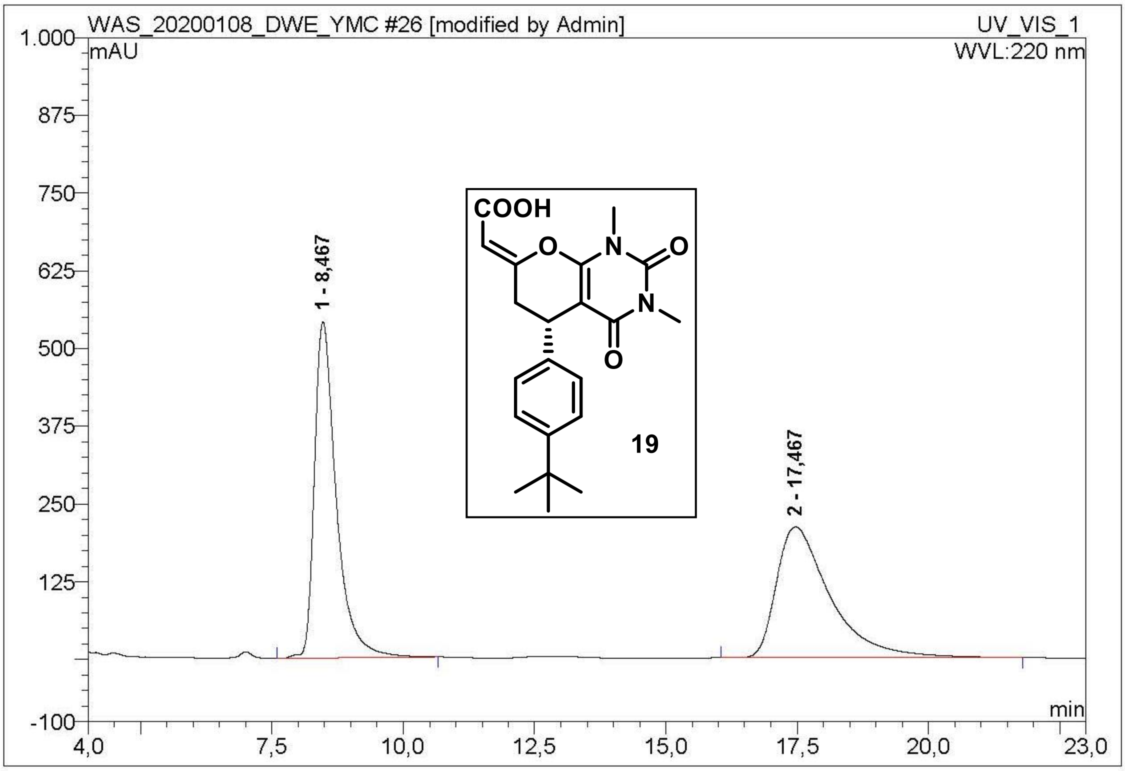

No.	Ret.Time min	Peak Name	Height mAU	Area mAU*min	Rel.Area %	Amount	Type
1	8,47	n.a.	541,303	260,398	51,10	n.a.	BMB*
2	17,47	n.a.	210,283	249,235	48,90	n.a.	BMB*
Total:			751,586	509,633	100,00	0,000	

Chromatogram

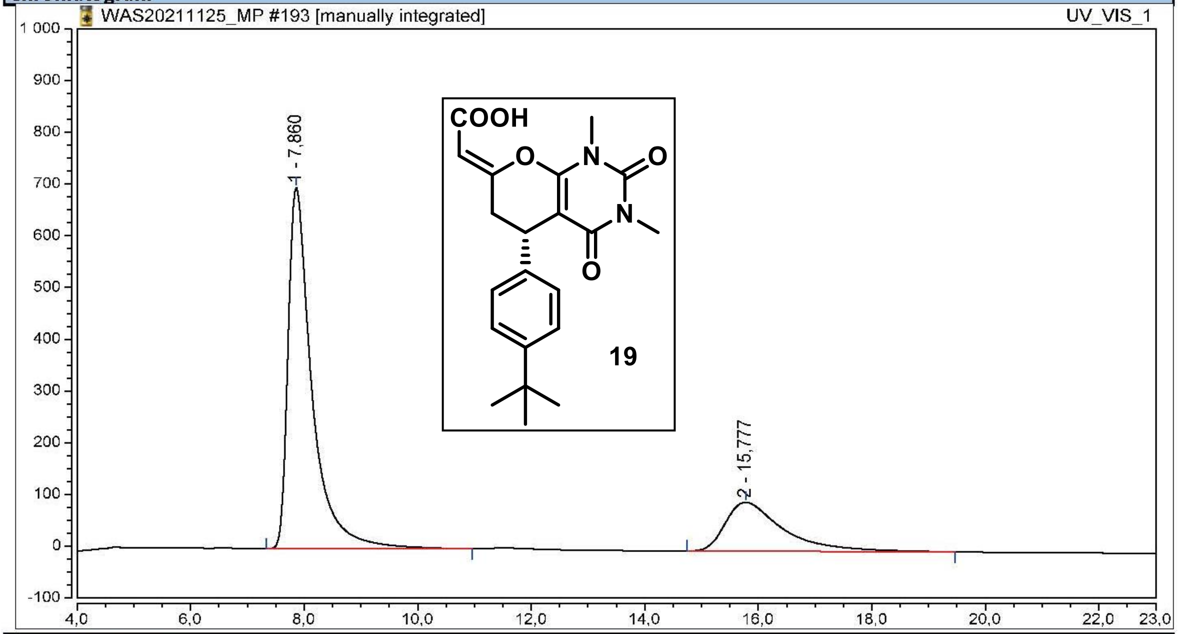

Integration Results

No.	Peak Name	Retention Time min	Area mAU*min	Height mAU	Relative Area %	Relative Height %	Amount n.a.
1		7,860	341,557	699,206	75,28	88,08	n.a.
2		15,777	112,133	94,603	24,72	11,92	n.a.
**Total:**			**453,691**	**793,809**	**100,00**	**100,00**	

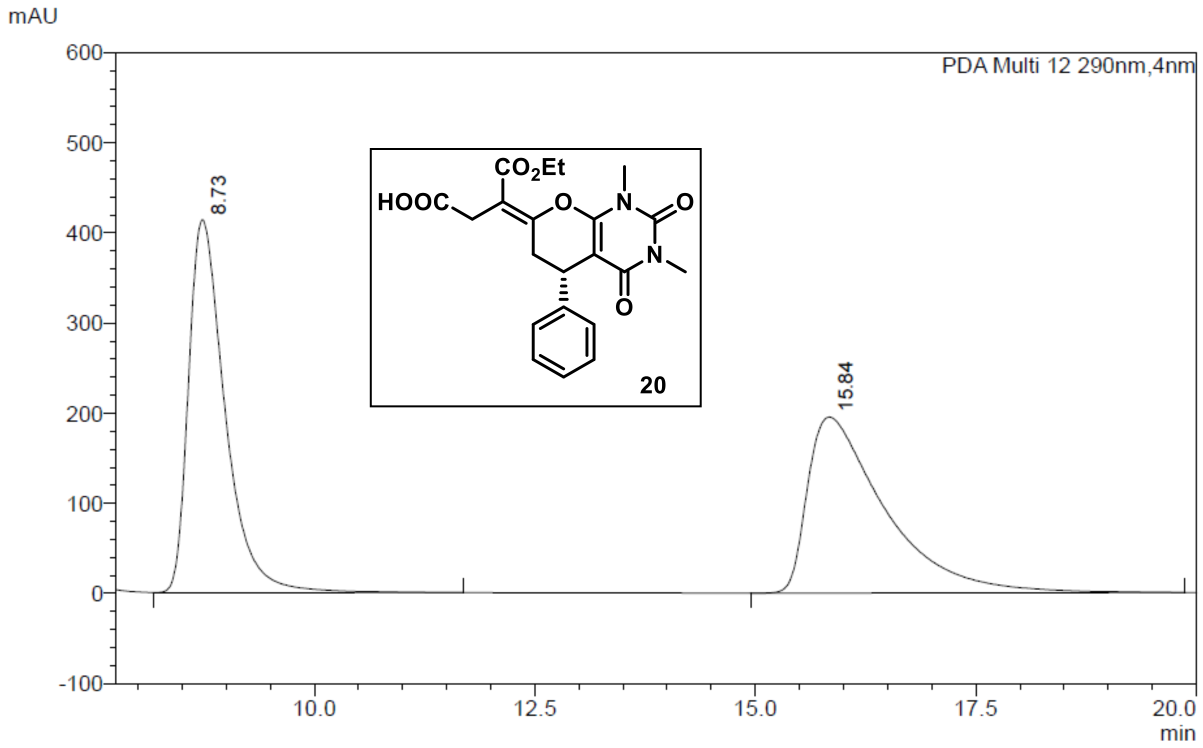

Peak Table

PDA Ch12 290nm

Peak#	Ret. Time	Area	Area%
1	8.73	12001921	49.98
2	15.84	12010753	50.02
Total		24012674	100.00

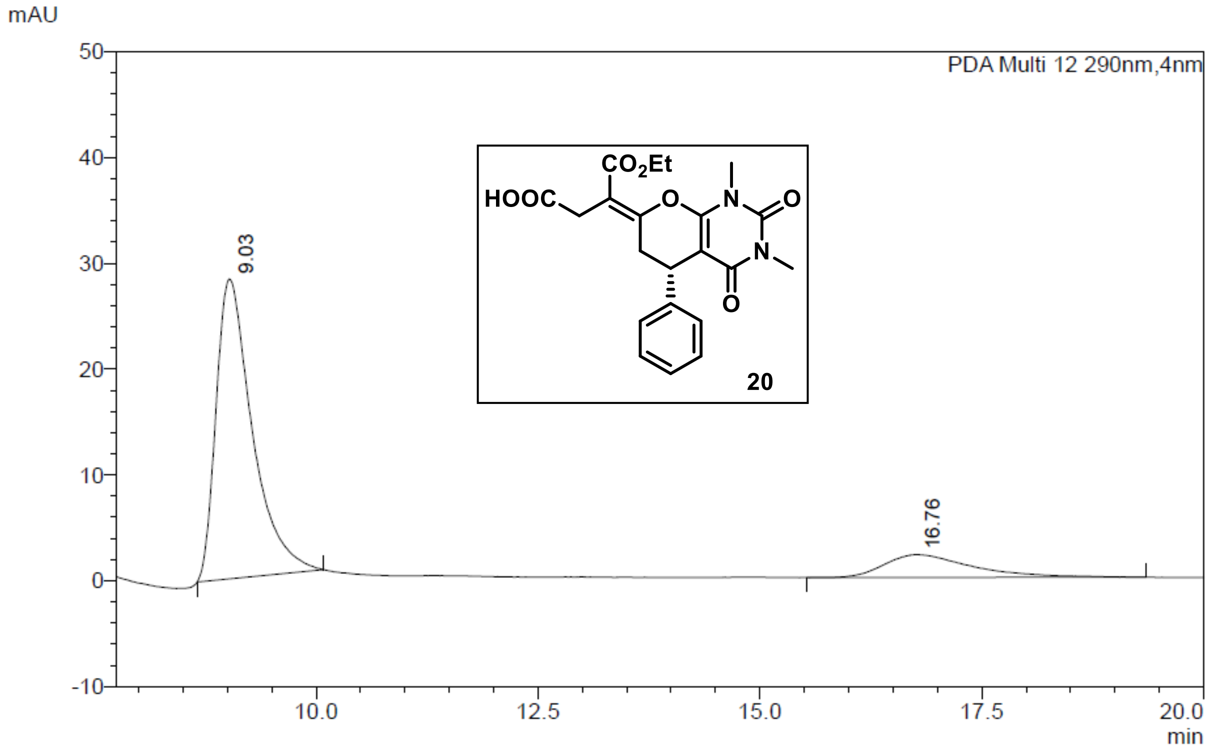

Peak Table

PDA Ch12 290nm

Peak#	Ret. Time	Area	Area%
1	9.03	803793	84.37
2	16.76	148935	15.63
Total		952728	100.00

Chromatogram

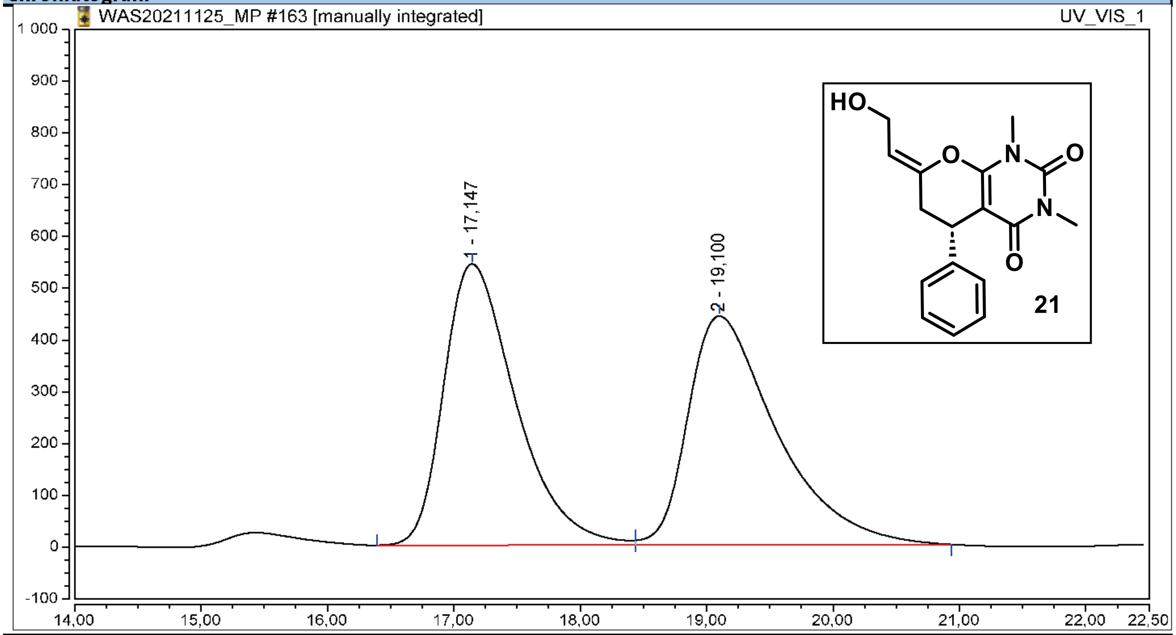

Integration Results

No.	Peak Name	Retention Time min	Area mAU*min	Height mAU	Relative Area %	Relative Height %	Amount n.a.
1		17,147	358,133	543,311	49,97	55,14	n.a.
2		19,100	358,526	442,015	50,03	44,86	n.a.
**Total:**			**716,658**	**985,326**	**100,00**	**100,00**	

Chromatogram

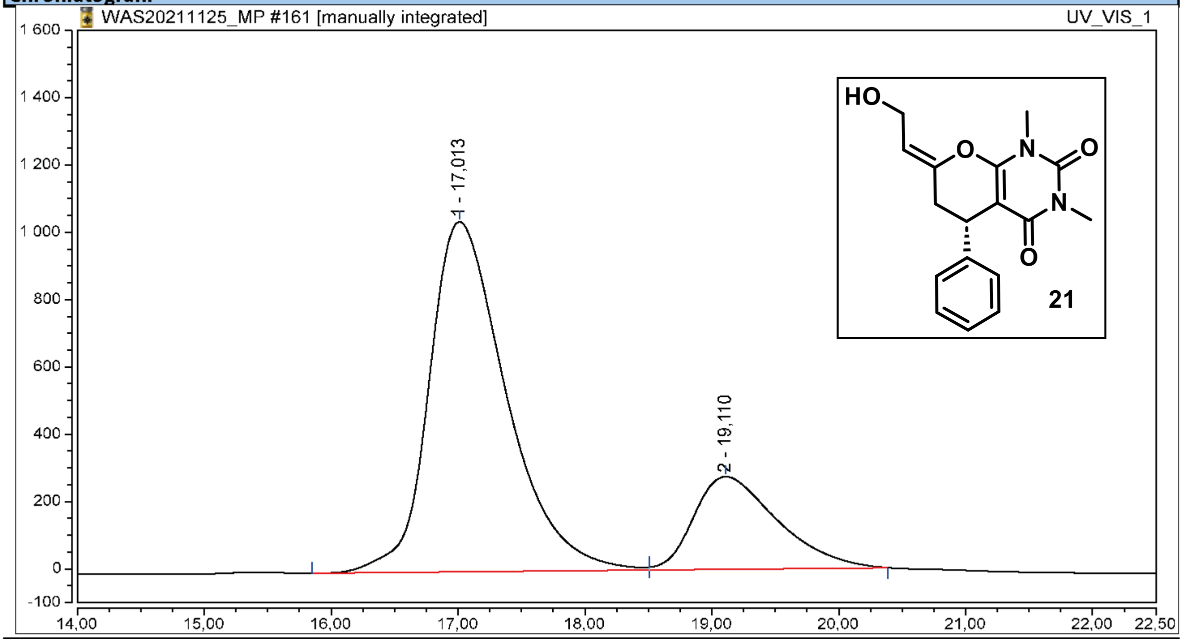

Integration Results

No.	Peak Name	Retention Time min	Area mAU*min	Height mAU	Relative Area %	Relative Height %	Amount n.a.
1		17,013	732,518	1040,215	77,77	79,08	n.a.
2		19,110	209,439	275,263	22,23	20,92	n.a.
**Total:**			**941,957**	**1315,478**	**100,00**	**100,00**	

Chromatogram

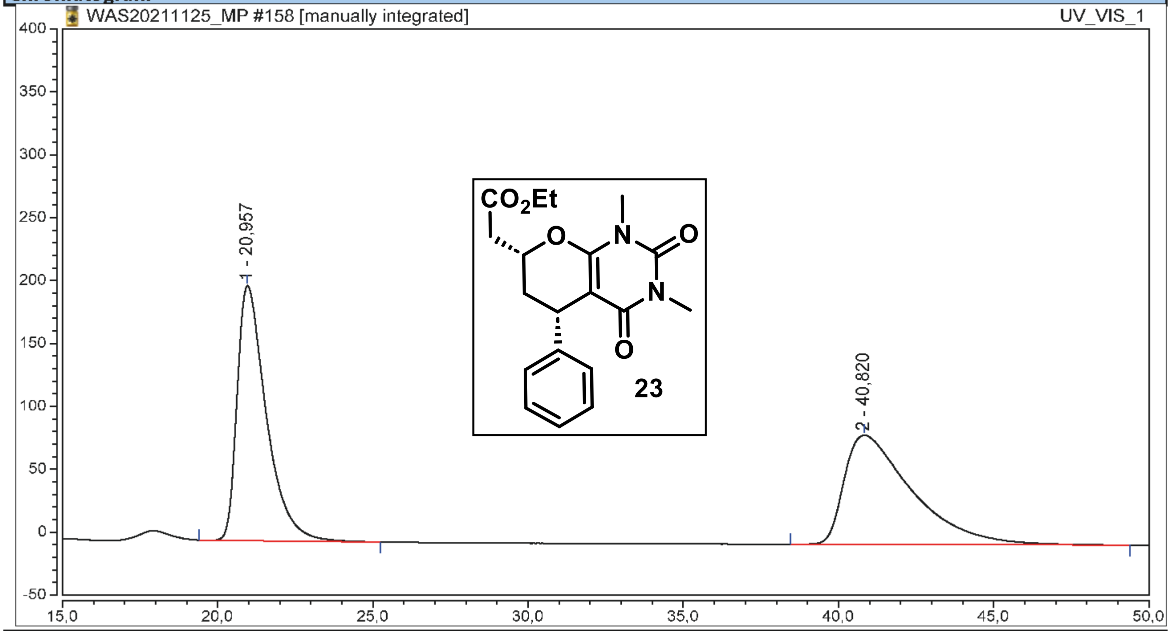

Integration Results

No.	Peak Name	Retention Time min	Area mAU*min	Height mAU	Relative Area %	Relative Height %	Amount n.a.
1		20,957	221,212	203,130	50,12	70,05	n.a.
2		40,820	220,191	86,857	49,88	29,95	n.a.
**Total:**			**441,404**	**289,987**	**100,00**	**100,00**	

Chromatogram

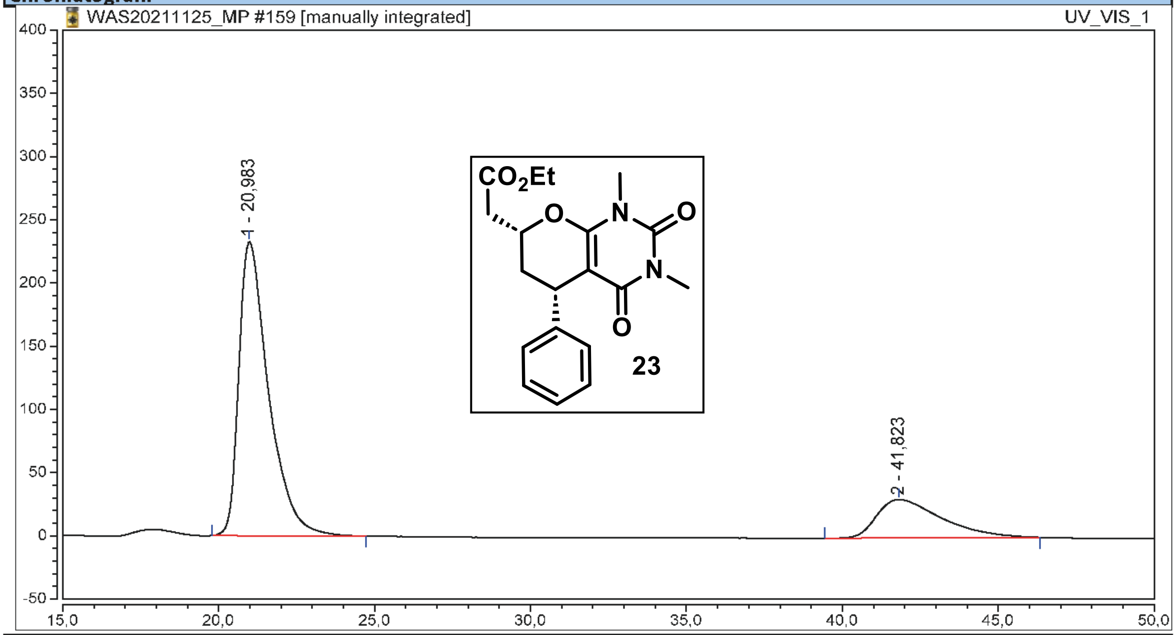

Integration Results

No.	Peak Name	Retention Time min	Area mAU*min	Height mAU	Relative Area %	Relative Height %	Amount n.a.
1		20,983	256,973	232,814	77,92	88,46	n.a.
2		41,823	72,832	30,386	22,08	11,54	n.a.
**Total:**			**329,805**	**263,201**	**100,00**	**100,00**	
